# The first identification of complete Eph-ephrin signalling in ctenophores and sponges reveals a role for neofunctionalization in the emergence of signalling domains

**DOI:** 10.1186/s12862-019-1418-z

**Published:** 2019-04-25

**Authors:** Arunkumar Krishnan, Bernard M. Degnan, Sandie M. Degnan

**Affiliations:** 1 School of Biological Sciences, The University of Queensland, Brisbane, Queensland Australia; 2 Present Address: National Center for Biotechnology Information, National Library of Medicine, National Institutes of Health, Bethesda, MD 20894 USA

**Keywords:** Multicellularity, Porifera, Ctenophora, Choanozoa, Receptor tyrosine kinase, Eph receptors, Ephrin ligands, Animal signalling pathways

## Abstract

**Background:**

Animals have a greater diversity of signalling pathways than their unicellular relatives, consistent with the evolution and expansion of these pathways occurring in parallel with the origin of animal multicellularity. However, the genomes of sponges and ctenophores – non-bilaterian basal animals – typically encode no, or far fewer, recognisable signalling ligands compared to bilaterians and cnidarians. For instance, the largest subclass of receptor tyrosine kinases (RTKs) in bilaterians, the Eph receptors (Ephs), are present in sponges and ctenophores, but their cognate ligands, the ephrins, have not yet been detected.

**Results:**

Here, we use an iterative HMM analysis to identify for the first time membrane-bound ephrins in sponges and ctenophores. We also expand the number of Eph-receptor subtypes identified in these animals and in cnidarians. Both sequence and structural analyses are consistent with the Eph ligand binding domain (LBD) and the ephrin receptor binding domain (RBD) having evolved via the co-option of ancient galactose-binding (discoidin-domain)-like and monodomain cupredoxin domains, respectively. Although we did not detect a complete Eph-ephrin signalling pathway in closely-related unicellular holozoans or in other non-metazoan eukaryotes, truncated proteins with Eph receptor LBDs and ephrin RBDs are present in some choanoflagellates. Together, these results indicate that Eph-ephrin signalling was present in the last common ancestor of extant metazoans, and perhaps even in the last common ancestor of animals and choanoflagellates. Either scenario pushes the origin of Eph-ephrin signalling back much earlier than previously reported.

**Conclusions:**

We propose that the Eph-LBD and ephrin-RBD, which were ancestrally localised in the cytosol, became linked to the extracellular parts of two cell surface proteins before the divergence of sponges and ctenophores from the rest of the animal kingdom. The ephrin-RBD lost the ancestral capacity to bind copper, and the Eph-LBD became linked to an ancient RTK. The identification of divergent ephrin ligands in sponges and ctenophores suggests that these ligands evolve faster than their cognate receptors. As this may be a general phenomena, we propose that the sequence-structure approach used in this study may be usefully applied to other signalling systems where no, or a small number of, ligands have been identified.

**Electronic supplementary material:**

The online version of this article (10.1186/s12862-019-1418-z) contains supplementary material, which is available to authorized users.

## Background

The origin of animal multicellularity and complexity appears to have required the evolution of elaborate internally-regulated intercellular signalling [1, 2]. Consistent with this premise, cell surface receptors and their interaction partners (ligands), which together constitute receptor-ligand signalling systems, have greatly expanded and diversified along the proximal stem leading to modern animals [3–7]. These signalling systems underlie both the development and the maintenance of animals, and are essential in the specification, differentiation, proliferation and movement of cells.

Although metazoans have a number of unique intercellular signalling systems, including Wnt and TGF-β (transforming growth factor-beta) pathways, they also use signalling pathways that are more ancient, such as the receptor tyrosine kinase (RTK) pathway [8–12]. These more ancient signalling systems often have expanded and diversified into metazoan-specific families. For instance, although closely-related unicellular holozoans have some of the largest and most diverse RTK families [5, 8, 11, 13, 14], these do not appear to be orthologous to the animal RTK families. In contrast, non-eumetazoan, early-branching or basal metazoan lineages (i.e. sponges and ctenophores) have clear orthologues of many bilaterian RTK families, including epidermal growth factor receptor (EGFR), Met, discoidin domain receptor (DDR) and Eph receptors [7, 15]. Intriguingly, however, the ligands of RTKs and other receptor families in these basal metazoans are currently either completely unknown or highly reduced in number compared to bilaterians and cnidarians [7, 11, 15], leaving a large gap in our understanding of the evolution of these signalling systems.

**Fig. 1:**
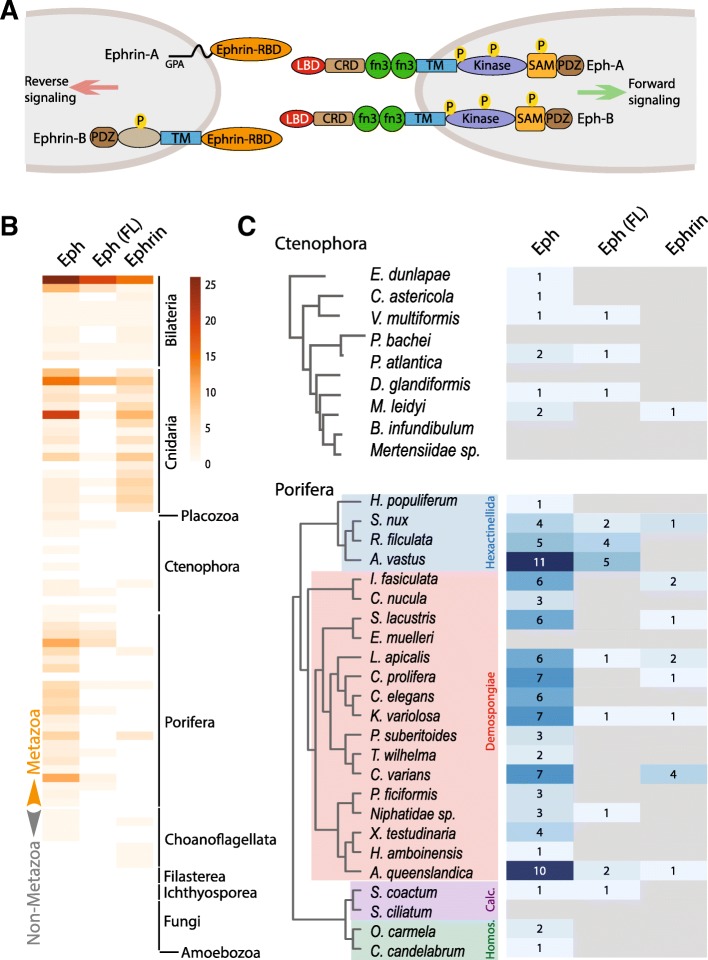
Presence and absence of Eph (receptor) and Ephrin (ligand) pairs in eukaryotes. **a** Schematic representation of ephrin ligand and Eph receptor protein domain architectures. Ephrin A and B ligands are depicted extending from a cell on the left, and Eph-A and -B receptors are extending from a cell on the right. A forward signal emanates from Eph receptors and a negative signal emanates from the ephrin ligands. CRD, Cysteine Rich Domain; fn3, fibronectin domain; GPA, GlycoPhosphatidylinositol (GPI)- linked Anchor; kinase, tyrosine kinase; LBD, Ligand Binding Domain; P, Phosophorylation sites; PDZ, Post synaptic density protein/Disc large tumor suppressor/Zonula occludens-1 domain; RBD, Receptor Binding Domain; SAM, Sterile Alpha Motif; TM, single-pass TransMembrane domain. **b** Heat map showing the number of Eph and ephrin genes detected in each of the sampled genomes and transcriptomes. Absolute counts of proteins used to plot the heap map are provided in Additional file 1: Table S2. **c** Number of genes detected in genomes and transcriptomes of ctenophore and sponge species. Homos., Homoscleromorpha; Calc., Calacarea. Shades of blue denote presence/counts, while grey boxes denote absence. Eph (FL), Full Length Ephrin receptor

Amongst the bilaterian RTK families identified in sponges and ctenophores, the Eph-receptors are of particular interest in the context of understanding the origins of metazoan intercellular communication. The Eph-receptors comprise the largest RTK subfamily in bilaterians and, unlike the other RTK families that bind secreted diffusible ligands, they interact with cell surface-associated ephrin ligands on neighbouring cells (Fig. 1a) [3, 16, 17]. Eph-ephrin complexes can act via diverse signalling modes that include bi-directional signalling in both the Eph- and ephrin ligand-bearing cells [3, 17–23]. In vertebrates, the ephrin ligand can exist as either a membrane-anchored (glycophosphatidylinositol (GPI)- linked) ephrin-A or a transmembrane type ephrin-B, and their receptor partners, based on binding preferences, are categorised as Eph-A and Eph-B receptors, respectively [3, 16, 17, 19, 24]. In vertebrates, Eph receptor ligand-binding preferences exhibit high promiscuity within their corresponding classes and a few interclass receptor-ligand pairs also exist (EphB2-ephrinA5 and EphA4-ephrinB2/B3 complexes) [14, 25–28].

Despite this promiscuity, all bilaterian Eph receptors have an identical domain architecture that includes an extracellular globular ligand binding domain (LBD), a cysteine rich region (containing sushi and EGF-like motifs), a variable number of fibronectin domains, a single-pass transmembrane domain that connects to intracellular kinase, and SAM (sterile alpha motif) and PDZ domains (Fig. 1a; [16, 17, 24, 29, 30]. The Eph-ephrin interaction is confined to the Eph’s extracellular LBD interacting with the extracellular receptor-binding domain (RBD) of the ephrin [3, 18, 24]. The Eph receptor LBD belongs to the galactose-binding (discoidin) domain-like superfamily (SCOP ID: 49785), and has eight major β-strands arranged in a compact β-barrel [31, 32]. The Ephrin-RBD is a member of the cupredoxin superfamily (SCOP ID: 49503) that predominantly binds copper, and functions in electron transfer between proteins [33, 34].

The Eph-ephrin receptor signalling pathway is amongst the best characterised of the RTK pathways, and has an array of roles in cell adhesion, differentiation, proliferation, migration, axon guidance and synapse formation [18, 20, 22, 23, 29]. Despite this, its evolutionary history remains obscure, typifying many metazoan signalling pathways for which the presence and role of receptors and, in particular, ligands in basal metazoans and non-metazoan holozoans are largely unknown or different from bilaterian systems [2, 35]. For instance, Eph receptors appear to predate the ephrin ligands, with receptors present in most metazoan lineages including sponges and ctenophores, but ligands detected only in cnidarians and bilaterians [1, 7]. So-called orphan receptors in these basal metazoans are likely to interact with highly divergent or different ligands [2], as yet undetermined. In addition, it is unclear whether Ephs and ephrins or similar proteins are present in the closest unicellular relatives of metazoans, the choanoflagellates.

In this study, we identify for the first time divergent ephrin ligands in sponges and ctenophores, placing the origin of this receptor-ligand system back at least to the beginning of the animal kingdom. Recovery of putative Eph and ephrins in choanoflagellates, although never together, raises the possibility that Eph-ephrin signalling may even have predated the Metazoa. Our analysis suggests that metazoan ephrins likely evolved from cupredoxins that lost the ability to bind copper. Metazoan Eph receptors appear to have evolved by the acquisition of the LBD via domain shuffling along the metazoan stem. The presence of the Eph-ephrin receptor-ligand system in the last common ancestor of metazoans suggests that it could have contributed to short distance cell-cell communication in the first animals.

## Results

### Identification of Eph receptors and ephrin ligands in non-bilaterian metazoans and putatively in choanoflagellates

To elucidate the origin of the Eph receptors and their membrane bound ligands, the ephrins, we undertook HMM (Hidden Markov Model) searches of a wide range of publicly available genomes and transcriptomes, and unpublished transcriptome datasets, with a greater focus towards non-bilaterian and non-metazoan genomes. This survey of 104 unikont species included in particular a comprehensive collection of non-bilaterian metazoans (24 sponges, 11 ctenophores and 17 cnidarians) and 28 unicellular holozoans (choanoflagellates, filastereans and ichthyosporeans), comprising all recently published transcriptomic and genomic datasets (Additional file 1: Table S1). Initial HMM searches using Pfam HMM models of canonical domains of the Eph receptors and ephrins recovered previously reported Eph receptors and ephrins from bilaterians, and also a small number of putative receptors and ligands in cnidarians.

Using iterative HMM searches based on these additional new sequences, we recovered Eph receptors from cnidarians, sponges and ctenophores; these were identified by the presence of their ligand-binding “Eph-LBD” domain, and numbered 85 (17 full-length), 100 (15 full-length), and 9 (3 full-length), respectively (Fig. 1b, c, Additional file 1: Table S2 and Table S3; Additional file 2). Across all analysed metazoans, we identified 244 receptors in total (Additional file 1: Table S3; Additional file 2), which included several fragmentary protein models that lack one or more domains. An extensive search for Eph-like receptors in choanoflagellate and filasterean genome/transcriptome datasets revealed that these unicellular holozoans do contain Eph-like receptors possessing fn3 (fibronectin type 3 repeats), a TM (trans-membrane) region and a Tyr-kinase domain with significant identity to bilaterian Eph receptors. A total of 14 proto Eph-like sequences were recovered, but most lack the Eph-LBD and thus cannot be considered as bona fide Eph receptors (see proto Eph-like sequences in Additional file 2). However, four of these proto Eph-like sequences (one each from the choanoflagellates *Salpingoeca urceolata*, *Salpingoeca rosetta*, *Hartaetosiga gracilis* and *Microstomoeca roanoka*) do contain a N-terminal domain that is not readily detectable by Pfam/CDD domain search engines. Manual inspection of an alignment with Eph-LBD of metazoan counterparts hinted at the presence of an Eph-LBD domain in these choanoflagellates, and thus each case was further investigated by searching against a database of HMM profiles constructed from individual Protein Databank (PDB) entries with the HHpred program. HHpred searches yielded significant similarity to the ephrin-binding (EPH-LBD) ectodomain of bona fide EPH receptors (See Additional file 1: supplementary notes). Reciprocally, a HMM search using a manually curated Eph-LBD HMM profile (built from a selection of metazoan EPH-LBDs from our survey) against all surveryed choanoflagellate datasets recovered four sequences with E-values ranging from 5.1e-13 to 4.5e-5 (Additional file 1: Supplementary notes). Taken together, these results indicate that choanoflagellates may contain Eph receptors with the ability to bind the ligand Ephrin via an Eph-LBD domain (see below for alignments and sequence conservations). Three of the four sequences that have a putative Eph-LBD domain also contain an intracellular Tyr-kinase domain; the fourth, which is found in *Salpingoeca urceolata*, has only the Eph-LBD and fn3 domains, but is missing the Tyr-kinase domain.

Regarding the Ephrin ligands, our comprehensive survey retrieved all of those previously known from bilaterians, as well as new ephrins from non-bilaterian metazoans (Fig. 1b; Additional file 1: Table S2 and Table S3; Additional file 2). Of particular note, our iterative HMM searches identified 14 novel ephrin ligands for the first time in sponges and ctenophores (Fig. 1c; Additional file 1, Table S3; Additional file 2); 13 of these were found across 8 demosponge and hexactinellid sponge species, and one in the ctenophore *Mnemiopsis leidyi* (Fig. 1c; Additional file 1: Table S3). These same HMM searches did not reveal any ephrin genes in the genome of the placozoan *Trichoplax adhaerens*. Strinkingly, akin to the scenario with the Eph-receptors, we were able to identify ephrin-like sequences that align well with metazoan counterparts in four choanoflagellate species: *Acanthoeca spectabilis*; *Salpingoeca urceolata*; *Stephanoeca diplocostata*; and *Salpingoeca infusionum*; see sequences in Additional file 2). These were further confirmed through HHpred searches against the PDB database, which recovered ephrins with high probability (Additional file 1: Supplementary notes). Of these, only the *Salpingoeca urceolata* transcriptome also contains an Eph-like receptor, but notably it is only a fragment that contains an N-terminal Eph-LBD but lacks the intracellular Tyr-kinase (Additional file 1: Table S3; Additional file 2).

Taken together, our iterative HMM search results retrieved Eph-ephrin receptor-ligand pairs in most metazoans, including sponges and ctenophores, suggesting that Eph-ephrin signalling was present in the last common ancestor to all contemporary animals. Our results also raise the possibility that the origin of Eph-ephrin signalling predated the divergence of metazoan and choanoflagellate lineages, as we recovered putative Eph-like receptors with intact ligand binding domains in some choanoflagellates, and ephrins in others. Notably, however, we found only a single instance where both an Eph-like receptor and an ephrin ligand was found in the same choanoflagellate (*Salpingoeca urceolata*), and in this case the receptor lacks the intracellular Tyr-kinase domain. Thus, based on currently available genome and transcriptome data, there is no evidence for functional Eph-ephrin signalling outside of the Metazoa, even though components are present in some choanoflagellates.

### Eph-ephrin ligand- and receptor-binding domains are conserved

**Fig. 2:**
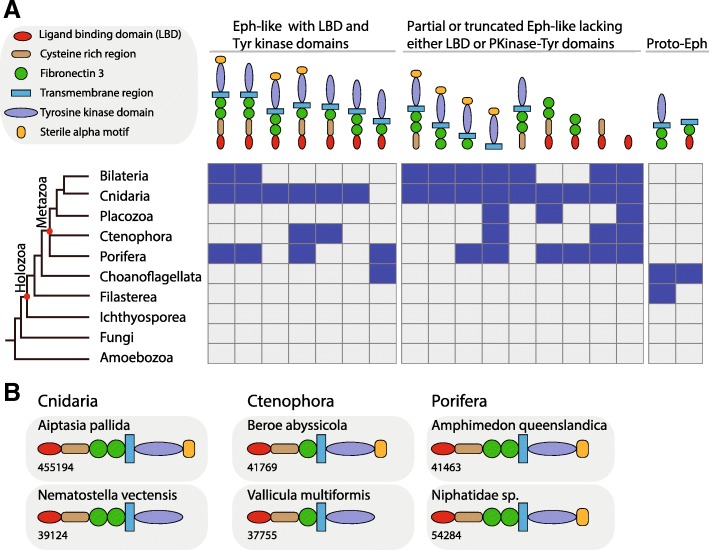
Conserved domain architectures of Eph receptors in different holozoan lineages. **a** Schematic representation showing the presence (filled blue boxes) or absence (filled grey boxes) of full-length, partial and truncated Eph-receptor-like domain architectures in metazoans and unicellular holozoans. **b** Representative domain architectures of full length Eph receptors in non-bilaterian metazoans. Sequence identifies (numbers) are provided below the schematic domain architecture, and the actual protein sequences can be found in the Additional file 2

We compared the domain architectures of all newly identified Eph and Eph-related receptors to the known bilaterian Ephs. We found that the general Eph receptor domain architecture that is present in bilaterians is conserved in cnidarians, ctenophores and sponges (Fig. 2a,b and Additional file 1: Figure S1). This architecture likely evolved before the emergence of metazoans, as putative Eph-like receptors in choanoflagellates also retain this general Eph ‘archetype’. Specifically, the four putative choanoflagellate receptors possess an N-terminal ligand binding domain (Eph-LBD), fn 3 repeats, a TM region, and often also an intracellular Tyr-kinase domain; the exception to this is that *Salpingoeca urceolata* lacks the Tyr-kinase domain). In general, variations of this highly-conserved domain organisation are limited to the number of fibronectin repeats, differences in the length of the cysteine rich regions and absence of the intracellular SAM domains (Fig. 2a,b; Additional file 1: Figure S1), although the latter might be attributable to gene models with truncated C-termini. We also find numerous partial gene models in basal metazoan transcriptomes that lack one or more domains of this general Eph receptor ‘archetype’ (Fig. 2a,b; Additional file 1: Figure S1).

To further investigate the ligand-receptor recognition and binding in the newly identified Ephs and ephrins, we analysed the sequence conservation of the Eph-LBD and ephrin-RBD domains. The interaction of Eph-LBD and ephrin-RBD is the first critical step for the initiation of Eph-ephrin signalling via the formation of heterodimers and tetrameric complexes [3, 18, 24]. We combined our sequence data with structural data of Eph-LB and ephrin-RB domains available from the PDB database (https://www.rcsb.org/) for structure-based sequence comparisons.

**Fig. 3:**
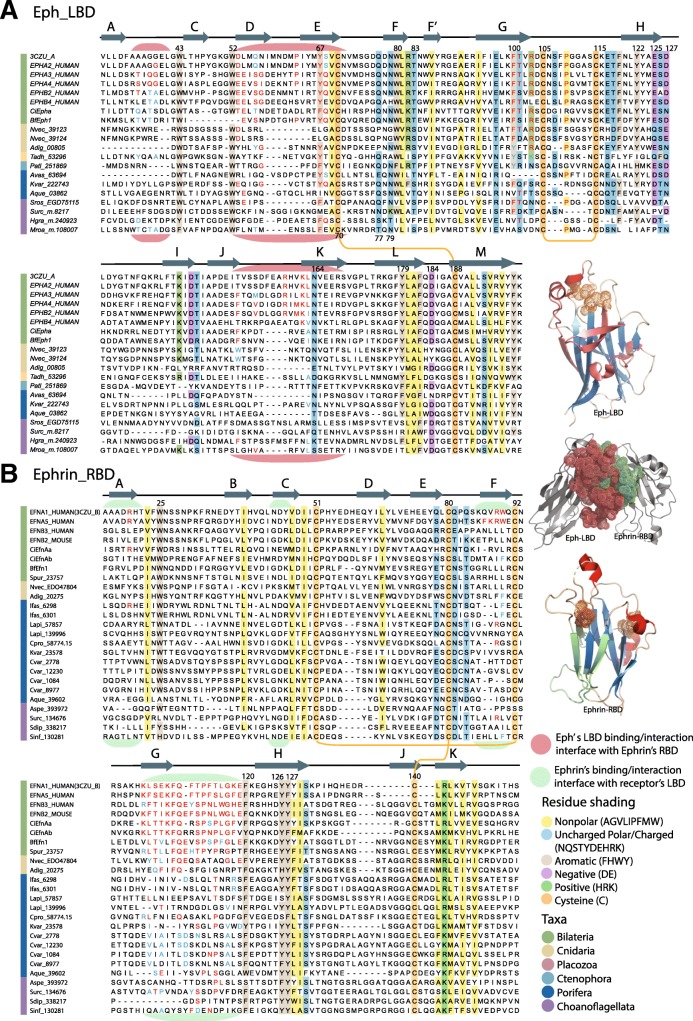
Characteristics of Eph LBD and Ephrin RBD domains. **a** Multiple sequence alignment of animal representatives of the Eph-receptor extracellular Ligand Binding Domain (LBD). Alignment depicts only the conserved blocks and residue number is not continuous. For complete alignment see Additional file 1: Figure S2. Secondary structure elements shown above the alignment are based on the LBD domain (Chain-A) of EphA2-EphrinA1 structural complex (3CZU) [30]. The “A-M” nomenclature shown above the alignment was adapted from [37] The three regions highlighted in pink (residues: 35–41; 52–70; 151–168) form the interaction interface between the Eph’s LBD and Ephrin’s RBD; the residues previously characterised to be involved in the interaction between these domains are shown in red. Such interface residues that are substituted with similar amino acid properties across alignment are marked in blue. **b** Multiple sequence alignment of the Ephrin ligand Receptor Binding Domain (RBD) in representatives of animals and choanoflagellates. Alignment depicts only the conserved blocks and residue number is not continuous. For complete alignment see Additional file 1: Figure S3. Secondary structure elements shown above the alignment are based on the RBD domain (Chain-B) of EphA2-EphrinA1 structural complex (3CZU). The “A-K” nomenclature shown above the alignment was adapted from [37]. The four regions highlighted in light green (residues: 19–21; 44–46; 89–90; 102–120) form the interaction interface between the Ephrin’s RBD and Eph’s LBD; residues previously characterised to be involved in the interaction between these domains are shown in red. Such interface residues that are substituted with similar amino acid properties across alignment are highlighted in blue. These interface residues were screened or identified based on all available vertebrate Eph-Ephrin structural complexes (see Additional file 1: Table S4 for comprehensive screen of interface residues and corresponding literature). The inset on the right (from top to bottom) shows structural renderings of: 1) EphA2-LBD (regions forming the interface with the Ephrin-A1-RBD are colored dark pink); 2) interaction interface regions of EphA2-LBD and Ephrin-A1-RBD (interface regions are shown as dots); Ephrin-A1-RBD (regions forming interface with EphA2-LBD are colored light green). See Additional file 1, Figure S2 and S3 for full-length alignments shown in panels A and B, respectively. For both the alignments, the colouring is based on 30% consensus threshold using the following scheme: nonpolar residues (AGVLIPFMW) shaded yellow, uncharged/charged polar (NQSTYDEHRK) shaded light blue, negatively charged (DE) shaded light purple, positively charged (HKR) shaded light green, aromatic (FHWY) residues shaded light brown and cysteines forming disulphide bridges shaded orange

Alignment of Eph-LBDs revealed a high degree of conservation, including invariant cysteine residues forming disulphide bridges (C105-C115 and C70-C188; residue numbers correspond to 3CZU_A human Eph-A2 LBD) and conserved blocks of beta sheets forming the antiparallel beta-sandwich jellyroll fold of the Eph ectodomains [36] (Fig. 3a; Additional file 1: Figure S2). In addition, several aromatic (W43, W52, Y67, W80, F100, Y122, Y179), polar (Q77, N79, T83, N164) and a few negatively charged residues (E125, D127, D184) around the interface loops are conserved. Strikingly, the choanoflagellate sequences share many of these conserved characteristics with the metazoan sequences, importantly including the cysteines forming disulphide bridges (Fig. 3a; Additional file 1: Figure S2). The loops connecting the beta sheets, such as the J-K and D-E (loop nomenclature was adapted from [37]) loops that protrude at the ligand binding interface region [30, 36, 37], as well as the long H-I loop known as the ‘class-specificity loop’, all are less conserved (Fig. 3a; Additional file 1: Figure S2). This is consistent with these regions being important for differential ligand class specificity and alternating between lock-and-key mode of binding to type A ephrin, and induced fit type mechanisms for type B ephrin [38, 39].

The ephrin ligands that we identified for the first time in sponges, ctenophores and choanoflagellates align well with the bilaterian counterparts (Fig. 3b; Additional file 1: Figure S3). Aside from the conserved cysteine residues forming disulphide bridges (C51-C92 and C80-C140; 3CZU_B; Fig. 3b) and conserved block of beta sheets, these newly identified ephrins also have fairly conserved length of functional loops, such as the G-H loops known to be crucial for receptor identification and interaction with Eph-LBD [37, 40, 41] (Fig. 3b). Of note, the ephrins contain four aromatic residues (W25, F120, Y126, Y127) in the proximity of the interface loops that are well conserved across all identified sequences, including those from choanoflagellates (Fig. 3b; Additional file 1: Figure S3). This high sequence conservation among all identified ephrins, including length of the connecting loops, is in agreement with the well-documented low degree of structural variance of ephrins [37, 40, 41].

Further, we surveyed residues crucial in stabilising interactions. These included the polar salt bridges, hydrogen bonds and the van der Waals contacts at the binding interface regions of Eph-LBDs and ephrin-RBDs, as previously published [28, 36, 37] or available in the PDB database (Additional file 1: Table S4). We find that the interface loops in both Eph-LBD and ephrin-RBD in sponges and ctenophores have generally undergone conservative substitutions and appear to have co-evolved; there are a few disruptive substitutions that were previously known to form salt bridges (Fig. 3a and b) [28, 36, 37].

### Lineage-specific expansions of Ephs and ephrins in cnidarians and sponges

**Fig. 4:**
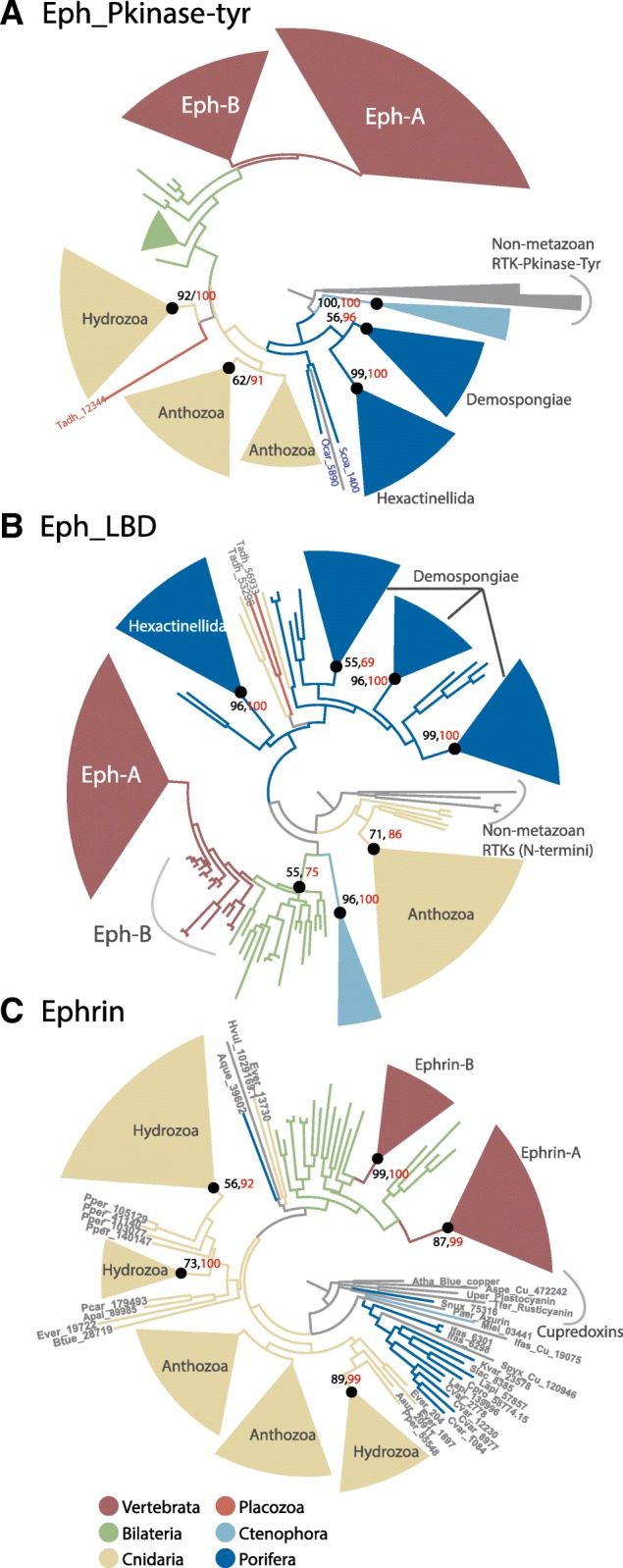
Phylogenetic reconstructions showing diversification and lineage-specific expansions of Eph-receptors and ligand ephrins. Phylogenetic tree topologies were inferred using Maximum Likelihood approach as implemented in RAxML and IQ-TREE softwares, and Bayesian approach as implemented in MrBayes software. Bootstrap values of > 50% as estimated using RAxML (Black) and IQ-Tree (Red) are marked on the key nodes and corresponding Bayesian posterior probability of threshold > 90% are shown as black dots. **a** Evolutionary relationships of metazoan Eph receptors, highlighting multiple expansions in cnidarians and sponges. The tree topology is inferred using the canonical intracellular Pkinase-Tyr domain. The Pkinase-Tyr domains of closely related non-metazoans RTKs were used as outgroup. **b** Evolutionary relationships of metazoan Eph receptors, highlighting multiple expansions in cnidarians and sponges. The tree topology is inferred using the extracellular ligand binding domain. N-terminal regions of closely related non-metazoan RTKs were used as outgroup. **c** Evolutionary relationships among metazoan ephrin ligands, highlighting multiple expansions in cnidarians. Closely related monodomain cupredoxins were used as outgroup. See Additional file 1, Figure S4, S5 and S6 for complete sequence labels and support values for the topologies shown in panels **a**, **b** and **c**, respectively. Raw tree files are provided in Additional file 3

We sought to reconstruct the origin and evolution of Eph receptors and ephrin ligands, and to define the origin of subtype diversification. Separate phylogenetic analyses of Tyr-kinase and Eph-LB domains in Eph receptors both support Eph-A and Eph-B subtype diversification occurring near the emergence of vertebrates [42], as the invertebrate bilaterians cluster together basally to the Eph-A/B subtype clade (Fig. 4a, b and Additional file 1: Figure S4 & S5). Our analyses also suggest that cnidarians and sponges have undergone unique lineage-specific duplications and divergences of Eph receptors. For instance, cnidarians have three distinct and well supported clades of Tyr-kinase Eph domains – two of which are anthozoan-specific and one of which is hydrozoan-specific – but only one large clade of Eph-LBDs (Fig. 4a, b and Additional file 1: Figure S4, S5). In contrast, demosponges have one clade of Tyr-kinase Eph domain and three distinct clades of Eph-LBDs, and hexactinellid sponges have one of each (Fig. 4a, b and Additional file 1: Figure S4, S5). The few Eph receptors identified in ctenophores comprise a single cluster of Tyr-kinase and Eph-LBD domains (Fig. 4a and b and Additional file 1: Figure S4 & S5).

Phylogenetic analysis of ephrin ligands reveals that the vertebrate ephrin-As and ephrin-Bs form separate clades, with invertebrate bilaterian ephrins basal to each (Fig. 4c and Additional file 1: Figure S6). This indicates that ephrin-A/B diversification predated the emergence of vertebrates, as previously proposed [42]. Invertebrate ephrin-A orthologues appear to be limited to urochordates (Fig. 4c and Additional file 1: Figure S6), consistent with ephrin-B being more evolutionarily ancient [43]. There appears to be two large lineage-specific expansions of ephrins in cnidarians, which encompass two and three anthozoan- and hydrozoan-distinct clades of ephrins, respectively (Fig. 4c and Additional file 1: Figure S6). We found that 55 of these cnidarian ephrins have a TM region at the C-terminal end, which is a characteristic of ephrin-B subtype (Additional file 1: Table S5). Although the presence of the C-terminal TM suggests this ephrin-B subtype was present in the bilaterian-cnidarian last common ancestor, the cnidarian ephrins are phylogenetically-distinct from the bilaterian ephrin-B subtypes (Fig. 4c and Additional file 1: Figure S6).

To examine this further, we analysed other non-bilaterian ephrins for the presence of TM regions at the C-termini, and glycosylphosphatidylinisotol (GPI) anchors that are characteristic of ephrin-A (see Fig. 1). We found that 64 of 114 non-bilaterian ephrins possessed a C-terminal TM, with nine predicted to possess also a C-terminally located lipid-anchoring site (Additional file 1: Table S5). Interestingly, seven sponge ephrins appear to have both a TM helix and a GPI anchoring site at their C-terminal end (Additional file 1: Table S5), thus possessing features of both ephrin-A or ephrin-B subtypes. The lone ctenophore ephrin – recovered from *Mnemiopsis leidyi* – is predicted to possess a GPI anchoring site by one of the utilised GPI-anchor predictors, albeit with low probability (Additional file 1: Table S5); there is no evidence for the presence of a TM helix. Given this is a fragmentary gene model and our inability to recover additional ephrins in ctenophores, it is currently unclear how ctenophore ephrins interact with the cell surface. The four putative ephrins that we identified in choanoflagellates all have a TM helix at the C-terminal end, but none have a C-terminally located lipid-anchoring site.

### Relationship of ephrins to monodomain cupredoxins and apicomplexan SAG1-related sequences (SRSs)

**Fig. 5:**
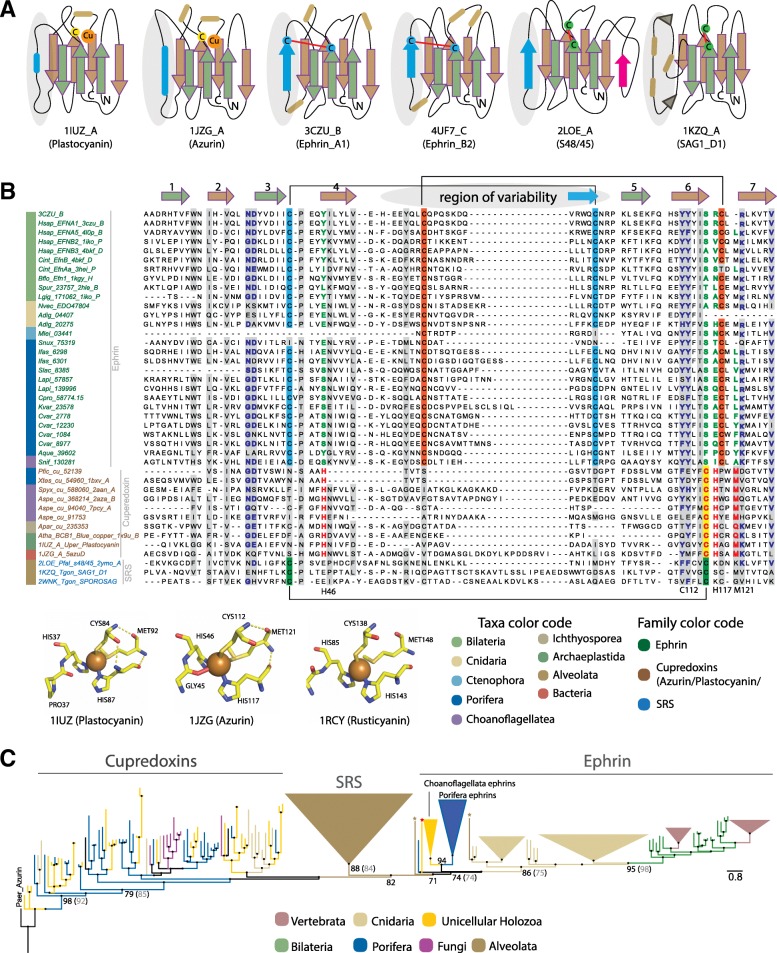
Evolutionary relationships of Ephrin, cupredoxin and SRS superfamilies. **a** Schematic rendering showing a common β-sandwich structural fold shared between ancient cupredoxins (first two from left), ephrins (middle two) and the closely related SRS superfamily (last two). Homologous β-sheets forming the β-sandwich fold across families are highlighted in green (front) and brown (rear). The region of variability extending after strand-4 are highlighted in blue. The insert between the canonical strands-1 and 2 is highlighted in pink for s48/45 domain. The conserved cysteine at the top of strand-3 forming different cross-sheet disulphide bridges in ephrins and SRS families, as well as the cysteine that chelates the copper metal in cupredoxins are shown; red line denotes disulphide bridges. For more details, see Additional file 1, Figure S7. **b** Structure based sequence alignment of ephrin, monodomain cupredoxin and SRS superfamilies from diverse taxa. The alignment illustrates the homologous β-strands forming the common β-sandwich structural core across families. The alignment includes structural representatives of ephrins (green text), monodomain cupredoxins (brown text) and SRS superfamily (blue text). Aligned columns with > 40% consensus are highlighted in grey. The strands are mapped on top of the alignment based on (3CZU_B) and coloured same as panel A, while numbers are marked only for strands homologous across families. Cysteines forming disulphide linkages for ephrins are highlighted in blue and orange background, while for the SRS family in green. Residues involved in chelating copper in cupredoxins are highlighted in red, while the non-conservative substitutions at the structurally equivalent positions possibly resulting in the loss of copper binding ability in ephrins are highlighted in green. Copper chelating residues and numbers marked at the bottom of the alignment corresponds to 1JZG (Azurin) from *Pseudomonas aeruginosa*. Residues involved in the chelation of copper in monodomain cupredoxins (plastocyanin, azurin, rusticyanin) are shown as ball and stick model in the inset at the bottom of panel B. To view the full-length alignment including the loops connecting the strands, and all disulphide linkages refer to Additional file 1: Figure S8. **c** Phylogenetic relationships between ephrins, monodomain cupredoxins and SRS superfamily. Tree topology was inferred using maximum-likelihood approach in the IQ-TREE software and the support values were estimated using ultrafast bootstrap (percentage from 1000 replicates). The topology was also tested using maximum-likelihood approach implemented in FastTree and support values are shown in parentheses for major nodes. Bacterial monodomain cupredoxins were used as outgroup. See Additional file 1: Figure S9 for more detail on phylogeny and complete sequence labels and support values. Raw tree files are provided in Additional file 3

As classified by the SCOP database, and examined previously [24, 44, 45] by fold prediction and HMM based HHpred programs, the ephrin receptor binding ectodomain constitutes a β-sandwich fold present in the large superfamily of copper binding cupredoxins (Fig. 5a; Additional file 1: Figure S7). This β-sandwich core shared by all these families is commonly referred as the cupredoxin or SRS fold, characterised by β-strands arranged into two β-sheets forming a Greek key β-barrel structure [34, 44, 45]. Unlike other cupredoxins, however, bilaterian ephrin receptor ectodomains lack a copper binding site and appear more structurally related to SRS superfamily of apicomplexan-specific proteins with a similar topology in the core β-strands [44].

To examine the relationship of ephrins to cupredoxins and the SRS superfamily, we performed sequence-structure-based comparisons and phylogenetic reconstructions. Using Pfam HMM models Copper-bind (PF00127), Cu_bind_like (PF02298) and Cupredoxin_1 (PF13473), we identified cupredoxin homologues in a diversity of organisms, including representative prokaryotes, non-metazoan eukaryotes and non-bilaterian metazoans. This survey recovered a range of cupredoxins, including the large multidomain enzymes comprising cupredoxin domain as one of their subunits. We restricted further analyses to the monodomain cupredoxins. Several of these were identified in sponges, ctenophores, unicellular holozoans and fungi (Additional file 1: Table S3). Representatives from these were aligned with bacterial monodomain cupredoxins, with ephrins identified in this study and with representative members of the SRS superfamily using a structure-based sequence alignment approach implemented in T-Coffee Expresso alignment server [46] (Fig. 5b; Additional file 1: Figure S8).

**Fig. 6:**
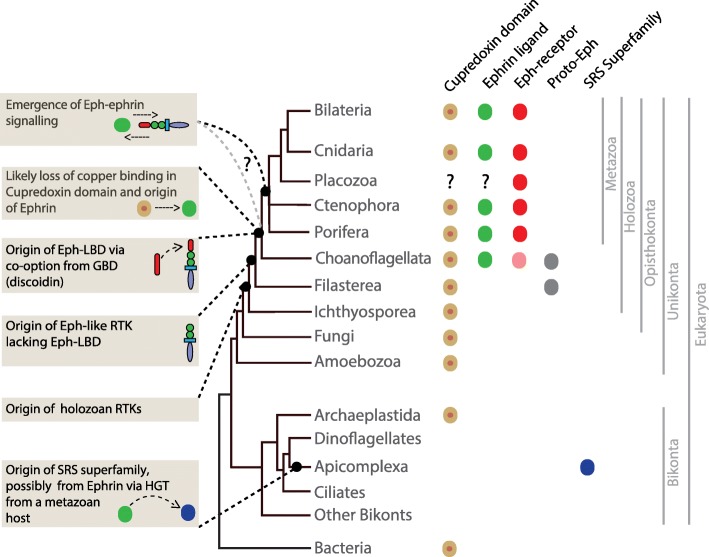
Reconstruction of the proposed evolution of Eph-ephrin receptor-ligand pairs. A simplified phylogenetic tree of the evolutionary relationship of relevant eukaryotes; not all eukaryotic lineages are shown. Prokaryotes are shown basal to eukaryotes. A coloured circle against a lineage name denotes the presence of a given gene family in that lineage; a question mark denotes uncertainty. An orange dot in the centre of brown circles representing monodomain cupredoxins denotes copper binding ability

This structure-informed alignment, along with the analysis of available crystal structures, enabled us to examine the structural conservation of ephrins in comparison with cupredoxins and SRSs, and specifically the structurally-equivalent residues of ephrins against the copper-binding active site of cupredoxins. This latter site is characterised by the thiolate sulfur of an invariant cysteine (C112) and the imidazole nitrogens of two conserved histidines (H46 and H117), accompanied by comparatively variable glycine (G45) and methionine (M121) (the numbers correspond to azurin (PDB ID: 1JZG) from *Pseudomonas aeruginosa*) [47] (Fig. 5a,b and Additional file 1: Figure S7 and S8). These analyses reveal that ephrins, cupredoxins and SRSs from disparate taxa have conserved blocks of β sheets that are likely required to maintain the structural fold. The ephrins have non-conservative substitutions at the amino acid positions in the copper binding active site of cupredoxins, which appear to underlie the loss of the copper binding ability (Fig. 5a,b and Additional file 1: Figure S7 and S8). Comparison of monodomain cupredoxin and ephrin-like sequences reveals the presence of copper binding active sites in the former, and their absence in the latter (Fig. 5b; Fig. 6).

A phylogenetic analysis comprised specifically of ephrins, apicomplexan SRSs and monodomain cupredoxins placed all ephrins from metazoans and choanoflagellates in a single clade, separate from both the monodomain cupredoxins and from a single apicomplexan SRS superfamily clade (Fig. 5c and Additional file 1: Figure S9). The SRSs are basal to the ephrin clade and the basal metazoan and unicellular holozoan monodomain cupredoxins largely clustered with their prokaryote counterparts (Fig. 5c and Additional file 1: Figure S9).

## Discussion

### Provenance of ephrin ligands and Eph receptors

The high diversity and disparity of cell-surface receptors in animals compared to their unicellular relatives suggests that the capacity for intercellular signalling expanded with the evolution of metazoan multicellularity [1, 2]. However, there has been a fundamental challenge in understanding the provenance of metazoan receptor-ligand systems. In particular, it is often difficult to identify ligand-receptor partners in basal metazoans such as sponges and ctenophores, and to determine how they relate to canonical ligand-receptor mediated signalling in bilaterians. The presence of orthologous receptor genes in sponges and ctenophores is consistent with signalling systems that are conserved in bilaterians and cnidarians also being functional in these phyla, which in turn is consistent with these pathways evolving before the diversification of basal animal lineages. However, the inability to often detect cognate ligands in sponges and ctenophore confounds reconstructions of ancestral states, and suggests that orthologous receptors in these taxa are binding highly divergent or analogous, potentially exogenous, ligands.

The RTKs typify this situation. They expanded before metazoan cladogenesis to give rise to a highly-diversified suite of metazoan-specific receptor subfamilies for which there are only a few matching ligands in sponges [7]. In this study, we sought to identify in non-bilaterians the ligands of the largest family of RTKs, the Eph receptors. This allowed us to reconstruct the evolution of this key signalling pathway and thus determine if the canonical bilaterian Eph-ephrin signalling system is a metazoan synapomorphy, or perhaps even predates the origin of the Metazoa.

Our comprehensive analysis of metazoan and non-metazoan genomes and transcriptomes identified the first ephrin ligands in sponges and in ctenophores, and found additional ephrins and Eph receptors in several eumetazoans. Eph receptors and ephrin ligand pairs forming an active signalling pathway are most likely a metazoan innovation, although non-metazoan holozoans do possess clear precursors of the signalling system (Fig. 6). For instance, the transcriptomes of some of the closely related unicellular holozoans analysed in this study do have Eph-like receptors, four of which include the Eph-LBD that is critical for binding the ephrin ligand (the choanoflagellate Eph-like receptors lack a cysteine rich region (containing sushi and EGF-like motifs), and intracellular SAM and PDZ domains (Fig. 2a). Similarly, we found evidence of ligand ephrins in unicellular holozoans. Importantly, however, we were unable to find any occurrence of full-length Eph-ephrin pairs in any of the unicellular holozoans. We did find both both an Eph-like receptor and a ephrin ligand in the choanoflagellate *Salpingoeca urceolata*, but the receptor lacks the critical intracellular Tyr-kinase domain and thus is likely non-functional. Because none of the non-metazoan holozoans possess a full-length Eph-ephrin receptor ligand pair, we cannot unambiguously trace the origin of the pathway to before the emergence of metazoans. As genome sequences of more non-metazoan holozoans become available, we look forward a final resolution of the possibility that a fully functional Eph-ephrin pathway predated the metazoans. In the meantime, based on the current data, we propose that the metazoan Eph receptor arose via domain shuffling that linked a proto-Eph-like RTK similar to that in extant unicellular holozoans with a more ancient galactose-binding domain (discoidin domain-like), which already had the capacity to bind ligands [48]. Consistent with previous studies [42], we found that EphA and EphB receptor subfamilies are restricted to vertebrates and likely arose from a single metazoan Eph receptor.

In contrast, the diversification of the ligand ephrin-A and ephrin-B subtypes appears to have occurred earlier, with ephrin-A and -B present in vertebrates and urochordates, and only ephrin-B present in cephalochordates and non-chordate bilaterians (this study; [42]. Cnidarians have had a uniquely large expansion of ephrins that is distinct from bilaterian ephrins and is characterised by a TM helix at the C-terminal end. We detected seven divergent ephrins in sponges that uniquely possess both a TM helix and a GPI anchoring site, and thus have diagnostic features in a single ligand that match those separating the bilaterian ephrin-A and ephrin-B types. As all non-bilaterian ephrins are basal to bilaterian ephrin-A and ephrin-B clades, it appears that these two clades evolved from an ancestral gene after diverging from a common ancestor with cnidarians. The ancestral metazoan ephrin may have possessed a TM helix and a GPI anchoring site, with each bilaterian ephrin clade maintaining one of these; cnidarians appear to have lost the GPI anchoring site.

### Lineage-specific ephrin and Eph evolution

Phylogenetic analyses of the LBD and the Tyr-kinase domain of Eph receptors indicate that this receptor family has undergone lineage-specific expansions and diversifications in non-bilaterian metazoans, namely cnidarians and sponges. There appears to be domain-specific selective pressures, as the extracellular Eph-LBDs have a different phylogenetic profile to the intracellular Tyr-kinase domains. For instance, the 13 Eph receptors identified in sponges separate into four major clusters when analysing the LBD, but only two when analysing the Tyr-kinase domain. This difference may reflect a capacity of the sponge Eph-LDB domain to interact with multiple, divergent ephrin ligands and possibly even with structurally analogous and closely related monodomain cupredoxins. In contrast to sponges, cnidarians have a single cluster of conserved Eph-LBD domains, but a large lineage-specific expansion of ephrins, suggesting a binding promiscuity; Eph-ephrin promiscuity is well documented in other animals [19, 25, 38]. It also suggests the cnidarians may form multimeric aggregation of ephrins to activate Eph signalling. In addition, cnidarians, sponges and ctenophores have a number of Eph-receptor domain architectures, including some that do not include a Tyr-kinase domain, that have not been found currently in bilaterians. Together, these observations are consistent with ephrin signalling being highly divergent in sponges, cnidarians and bilaterians; the limited ctenophore data prevents detailed inferences. These diverse Eph-like factors could include catalytically null Eph receptors that function in cell-cell adhesion rather than in signalling [49, 50].

In contrast to these differing evolutionary trajectories in bilaterians, cnidarians and sponges, the overall domain architecture of metazoan Eph receptors is highly conserved. This, together with the conserved structure scaffold (β-sandwich) of the ephrin-RBD and Eph-LBD domains, including the length of the interface loops crucial for receptor-ligand binding, suggests selective constraint on Eph-ephrin signalling throughout metazoan evolution. Nonetheless, it seems likely that structural features required for stable Eph-ephrin signalling were established in the last common ancestor to living metazoans.

### Ephrins likely evolved from monodomain cupredoxins

The ephrin receptor binding ectodomain (ephrin-RBD) is characterised by β-strands arranged into two β sheets forming a β-sandwich fold, which is also shared by the cupredoxin superfamily of copper binding proteins and apicomplexan-specific SAG1-related sequence (SRS) family [33, 34, 51]. The ephrin-RBD is most structurally similar to the monodomain cupredoxins, such as plastocyanin and azurin [24, 34, 52, 53], and to the apicomplexan SRS family, including SAG1 [44, 54]. Such relationships are also reflected in Dali and HHpred based structure-similarity searches. Unlike the ephrins and SRSs, which are restricted to metazoans and apicomplexans respectively, the cupredoxins exist in a wide range of organisms. They exist either as monodomain cupredoxins found predominantly, but not exclusively, in bacteria (amicyanin, azurin, pseudoazurin, rusticyanin), or as components of (i) galactose oxidase in fungi, (ii) hemocyanins, which are oxygen carriers in many arthropods and molluscs, or (iii) larger enzymes such as laccase in plants, fungi and insects, ascorbate oxidase in plants, ceruloplasmin in mammals [33, 34, 51]. Our sequence-structure and phylogenetic analyses are consistent with ephrin and SRS families either evolving independently from a monodomain cupredoxin ancestor, or SRS being derived from a horizontal gene transfer event from a metazoan. The latter hypothesis is supported by the restriction of the SRS family to apicomplexans, consistent with these parasitic alveolates having obtained the gene horizontally from a metazoan host [44].

We propose that prior to metazoan cladogenesis, an ancient monodomain cupredoxin underwent post-duplication neofunctionalization that included the loss of the copper binding site to give rise to the ephrin gene family. During this period, a functional monodomain cupredoxin was retained, as evidenced by its presence in non-bilaterian metazoans, but appears then to have been lost early in bilaterian evolution. Supporting this close relationship between ephrin and monodomain cupredoxins is the ability of partial peptide derivatives of a bacterial protein azurin to bind to EphB2 and EphA6 receptors [52]. In addition, the Ephrin-RBD required the acquisition of a mechanism to attach to the cell membrane either via TM helix or GPI-anchoring. Other cupredoxins have evolved similar abilities. For example, the bacterium *Neisseria gonorrhoeae* has a lipid-modified azurin cupredoxin with N-terminal lipid attachment to the outer membrane [55]. Similarity, other cupredoxin domains are part of large multidomain enzymes that are anchored to the membrane [56].

### Eph-receptor evolved by domain shuffling and externalising a galactose-binding domain

An essential feature in the evolution of the metazoan ephrin-Eph signalling system was the coupling of the Eph-receptor LBD to the internal Tyr-kinase domain. Unicellular holozoans do have several RTKs that resemble Eph receptors – they include fn3 domains and a Tyr-kinase domain that is similar to the Eph kinase domain, and in a few cases these domains are linked to an Eph receptor LBD-like domain. This suggests that the evolution of the bona fide metazoan Eph-receptors was the result of a domain shuffling event that first linked the LBD to the proto-Eph receptor before the divergence of metazoan and choanoflagellate lineages. However, a fully-functional Eph-receptor that also includes a cysteine rich region (containing sushi and EGF-like motifs), an intracellular SAM and a PDZ domain, appears to have evolved uniquely along the proximal metazoan stem. Both the SCOP database and the Pfam Clan classify Eph-LBD as part of the galactose-binding domain-like or carbohydrate-domain like superfamily, which contains a large number of beta-sandwich domains including the discoidin domain with the jelly roll-like topology [31, 32]. This superfamily includes the ancient discoidin domain (alternatively known as F5/8 type c domain) that is present in all domains of life and in diverse extracellular and membrane proteins, including blood coagulation factors, enzymes, receptors and proteins involved in cellular adhesion and migration [31, 32, 57–59], such as neuropilins, neurexins and receptor tyrosine kinase-discoidin domain receptor (DDR) [32, 48]. In Discoidin Domain Receptors (DDRs), the discoidin domain binds extracellular fibrillar collagen [48]. Similarly, the Eph-LBD appears to have evolved from a galactose-binding domain-like prior to, or after, being linked to a RTK similar to that present in extant unicellular holozoans; this appears to have occurred after the divergence of metazoan and choanoflagellate lineages.

A feature common to both the cupredoxin domain and the galactose-binding domain-like is that they are predominantly β-barrel proteins with Greek-key or jelly-roll barrel and such β-barrel domains. These are commonly present in the extracellular region of cell-surface proteins and both domains appear to have been recruited into such proteins at different times (e.g. [56] [31, 32]. The linking of ancient domains with membrane-bound external proteins appears to have increased in early metazoan evolution in concert with the origin of multicellularity [60]. We suggest that the co-option of these ancient domains from the cupredoxin and the galactose binding superfamilies of beta barrel proteins played a crucial role in the emergence of Eph-ephrin signalling early in animal evolution.

## Conclusions

We have shown here that the provenance of functional Eph-ephrin pairs can be traced back to the common ancestor of extant metazoans, much earlier than previously reported. Our identification of both Eph-receptors and ephrin ligands in functional pairings in sponges and ctenophores suggests this important RTK signalling pathway evolved along the metazoan stem after the divergence of animal and choanoflagellate lineages; the presence of Eph- and ephrin-like sequences in some choanflagellates leaves open the potential of an earlier origin of this signalling pathway. Both the ephrin-RBD and the Eph-receptor LBD appear to have evolved from ancient domains predominantly involved in electron transport and cell adhesion. We propose that the ephrin-RBD evolved from monodomain cupredoxin that lost the ability to bind copper and became linked to a TM helix and/or a GPI anchoring site, and that the Eph-LBD originated from a galactose-binding-like domain that paired with a proto-Eph RTK. Although Eph-receptors in sponge and ctenophores are highly similar to their bilaterian and cnidarian orthologues, the ephrin ligands differ markedly and had not been detected prior to this study. Their existence supports the parallel emergence of both Eph receptors and ephrin ligands prior to the diversification of crown metazoans, and is consistent with a crucial role for Eph-ephrin-mediated short distance cell-cell communication in the emergence and maintenance of metazoan multicellularity.

## Methods

### Taxon sampling and proteome/transcriptome datasets

To trace the evolutionary history of Eph-ephrin signalling system, we analysed complete genomes or transcriptomes of 104 unikont species, including 3 vertebrates, 1 jawless vertebrate, 9 invertebrate bilaterians, 17 cnidarians, 1 placozoan, 11 ctenophores, 24 poriferans, 28 unicellular holozoans, 9 fungi and 1 amoebozoan. For vertebrates, we downloaded manually curated human and mouse Eph and ephrin sequences from the SwissProt database; *Danio rerio* was subjected to sequence similarity search methods, similar to the rest of the analysed species. Overall our dataset included 31 genomes and 73 raw or assembled transcriptomes retrieved from the public databases; a number of unpublished transcriptomes were also included (Additional file 1: Table S1 and accompanying note). For unannotated transcriptomes, predicted protein sequences were generated by determining the longest open reading frame between stop codons for each sequence, using the program getorf available in the EMBOSS v6.5.7 software package [61].

### Identification of Eph-receptor and ephrin-ligands

All potential Eph-receptors and ephrin ligands were identified by HMMER searches against genome and transcriptome datasets using default parameters with an e-value cut-off of 0.01. We employed an iterative HMM search by building manually curated HMM profiles from sequences obtained from initial HMM searches, which used profile HMMs of Pfam domains found in canonical Eph receptors: Ephrin_lbd (PF01404); fn3 (PF00041); Pkinase_Tyr (PF07714); SAM_1 (PF00536); EphA2_TM (PF14575), a representative of Eph-TM regions and common domains that envelope cysteine rich regions [Ephrin_rec_like (PF07699), Sushi (PF00084), EGF (PF00008)]. Similarly, the Pfam domain Ephrin (PF00812), archetypal for ephrin-A and ephrin-B was used to search for ephrin orthologues. Novel putative Ephs and ephrins initially identified in non-bilaterian metazoans were aligned with representative full-length sequences from bilaterians to build new, distinct HMM profiles that were used in the second iteration. This iterative approach using domain specific HMM profiles was repeated for two rounds by including newly identified sequences to previous alignments. This approach maximised the chances of identifying distant homologues in basal metazoans and non-metazoans, and identified partial sequences, which may contain only one of these domains. After removal of redundant sequences using CD-HIT software, a final list of Ephs and ephrins were used in further sequence and phylogenetic analysis. For display purposes, we built heap maps using pheapmaps package in R. Absolute protein counts of Eph and ephrins, identified from each analysed species were plotted as heat maps (see Additional file 1: Table S3 for gene numbers).

### Retrieval of cupredoxins and SRS superfamily

Cupredoxin domains were identified by HMMER searches against all analysed taxa using Pfam HMM models such as Copper-bind (PF00127), Cu_bind_like (PF02298) and Cupredoxin_1 (PF13473). These HMMER searches retrieved several cupredoxin domain containing sequences including monodomain and multidomain cupredoxins, however, we restricted our analysis to monodomain cupredoxins, as mutlidomain cupredoxins contain the same domain in additional copies. Our strategy to search for cupredoxins in metazoans and closest unicellular relatives was to verify if non-bilaterian metazoans containing ephrins also encode genes for monodomain cupredoxins with copper binding centres. This allowed us to examine possible scenarios of the diversification of ephrins from cupredoxins during the eukaryotic evolution. We also retrieved the SRS superfamily members, which are specific to apicomplexans, to resolve the overall phylogenetic relationships with the closely related ephrins and cupredoxins. SRS superfamily members were recovered through BLASTP searches against the NCBI NR database using representatives (SRS3, SAG1, SRS8, SAG2A, SAG3) from several SRS families including S48/45 that constitutes the same SRS- structural fold. Such search strategy recovered the large expansions of SRS superfamily, and thus the overall dataset was clustered based on 40% identity threshold CD-HIT [62] clustering algorithm. Representatives from each cluster were utilised for further phylogenetic comparisons.

### Multiple sequence alignments and phylogenetic analysis

Multiple full-length sequence alignments were computed using MAFFT software (Version 7), utilising the E-INS-I algorithm, optimised for sequences with conserved motifs and carrying multiple domains, with BLOSUM62 as the scoring matrix and default parameters [63]. Domain envelope regions (start-stop sites) were defined for all domains in each sequence using a local Pfam search against the latest Pfam database version 30. Domain envelope regions obtained from Pfam searches were defined in a BED file, and exact domain regions were cut from full-length sequences using fastaFromBed as implemented in BEDTOOLS package [64]. Separate multiple sequence alignments were computed for each domain using MAFFT software (Version 7), utilising the L-INS-I algorithm, optimised for sequences containing one domain that could be aligned [63]. Such alignments were manually trimmed using Jalview and edited to remove badly aligned regions using a seed alignment as a guide. These alignemnts were then used to compute domain-specific phylogenetic trees.

To analyse the potential loss of copper binding sites in ephrins, we generated a structure-based sequence alignment using the T-Coffee Expresso alignment server [46]. With numerous structures available for Ephrin and other monodomain cupredoxins, Expresso automatically identified the closest homologues of the sequences within the PDB database and computed a structure-based sequence alignment. This ensured that structurally equivalent residues were aligned to unambiguously infer the potential loss of copper-binding sites in ephrins.

Maximum-likelihood phylogenetic trees were estimated by RAxML using PROTGAMMAAUTO to automatically determine the best protein substitution model for the given dataset [65]. Statistical support for the nodes were estimated using a rapid Bootstrap analysis (100 replicates) and search for best scoring ML tree was computed as implemented in RAxML. Maximum-likelihood phylogenetic trees were also computed using the edge-linked partition model as implemented in the IQ-TREE software [66]. ModelFinder [67] was used to indentify the best-fit model for accurate phylogenetic estimation for a given dataset, while the branch supports were obtained using the ultrafast bootstrap method (1000 replicates) [68]. Additionally, Shimodaira–Hasegawa approximate likelihood ratio test SH-aLRT branch test was computed as implemented in the IQ-TREE software [66]. Bayesian inference trees were calculated using MrBayes with two parallel runs for 300,000 generations, sampling every 100 generations [69, 70]. Diagnostics were calculated for every 1000 generations (diagnfreq = 1000) to analyse the convergence of the two independent runs starting from different random trees. Posterior probabilities were estimated using MCMC analysis and a Gamma shaped model was used to estimate the variation of evolutionary rates across sites. A stop rule was applied to terminate the MCMC generations when the standard deviation of split frequencies dropped below 0.01. For computing the overall topology showing the relationships of ephrin, monodomain cupredoxins and SRS superfamily from an array of diverse taxa (Fig. 5c and Additional file 1: Figure S9), we employed approximately maximum-likelihood method implemented in the FastTree program [71]. However, to improve the accuracy we increased the number of rounds of minimum­evolution SPR moves to four rounds (−spr 4) and used options “-mlacc 2” “-slownni” to make the maximum likelihood NNIs more exhaustive. This ML tree topology inferred from the FastTree program was also tested using the IQ-TREE software with options as mentioned above. The support values from three different tree building softwares are shown in the figures repoted in the manuscript. Raw tree files for all the trees reported in the manuscript are provided in the Additional file 3. All computed trees were rendered using the FigTree program (http://tree.bio.ed.ac.uk/software/figtree/).

### Prediction of TM regions and lipid anchoring in ephrins

To distinguish between membrane-anchored (GPI- linked) ephrin-A or a transmembrane type ephrin-B we predicted potential TM regions and GPI anchoring sites in all newly identified ephrins from non-bilaterian metazoans. We utilised Topcons single (a reliable approach that engage a consensus predictive methodology derived from outputs of five different TM prediction tools) [72] for predicting transmembrane regions, and PredGPI [73] and GPI-SOM [74] for predicting GPI anchor sites. A consensus output from Topcons single was considered indicative of the presence of TM helices and similarly sequences were considered to potentially GPI anchored if both PredGPI and GPI-SOM supports for the presence of GPI anchor with high specificity.

### Analysis of protein domain architectures

Inferred protein domain organisations were obtained using HMMER searches against Pfam database and Reverse Position-Specific BLAST searches against the pre-calculated Position-Specific Score Matrix (PSSMs) of the Conserved Domain Database (CDD) version 3.14. The cysteine rich regions of Eph-receptors, flanked between the Eph-LBD and the fibronectin domains are generally informed as either Sushi, EGF-like and other cysteine rich domains in Pfam and CDD searches. To be consistent, we uniformly denote the region between Eph-LBD and fibronectin as CRD (Cysteine rich domain). Also, as several of identified sequences lack one or more domains, we consider Eph-receptors as to be potentially full-length receptor, only if both the N-terminal ephrin-ligand binding domain, and C-terminal tyrosine kinase domain were present.

## Additional files

Additional file 1: **Figure S1.** Domain architectures of Eph receptors identified across all analyzed taxa. **Figure S2.** Multiple sequence alignment containing selected representatives of the Eph-receptor extracellular Ligand Binding Domain (LBD) animals and choanoflagellates. **Figure S3.** Multiple sequence alignment of the Ephrin ligand Receptor Binding Domain (RBD) containing representatives from animals and choanoflagellates. **Figure S4.** Evolutionary relationships of metazoan Eph receptors inferred using the canonical intracellular Pkinase-Tyr domain. **Figure S5.** Evolutionary relationships of metazoan Eph receptors inferred using the extracellular ligand binding domain (LBD). **Figure S6.** Evolutionary relationships among metazoan ephrin ligands, highlighting multiple expansions in the phylum Cnidaria. **Figure S7.** Cartoon rendering of representative structures of cupredoxins, ephrins and the SRS superfamily. **Figure S8.** Structure based sequence alignment of ephrins, monodomain cupredoxins and the SRS superfamily from diverse taxa. **Figure S9.** Phylogenetic relationships between ephrins, monodomain cupredoxins and SRS superfamily. **Table S1.** List of analyzed taxa and database sources. **Table S2.** Dataset utilized to plot the heat map shown in Fig. 1b. **Table S3**. Distribution of Eph, ephrin and monodomain cupredoxins. **Table S4.** Mapping of Eph-LBD and Ephrin-RBD interaction interface residues. **Table S5.** Predicted TM regions and lipid anchoring sites in ephrins from non-bilaterian metazoans. **Accompanying note.** Genome/transcriptome datasets used for sequence search. Eph-LBD and ephrin hits in choanoflagellates. (PDF 47231 kb)

Additional file 2: Complete FASTA sequences of all Eph receptors, ephrin ligands and cupredoxins identified new, or used for new analyses, in this study. (PDF 1079 kb)

Additional file 3: Raw tree files of all phylogenetic trees reported in the manuscript. (PDF 271 kb)

## Abbreviations

CDD: Conserved Domain Database
CRD: Cysteine Rich Domain
DDR: Discoidin Domain Receptor
EGFR: Epidermal Growth Factor Receptor
Eph: Ephrin receptor
fn3: fibronectin type 3 repeats
GPI: Glycosylphosphatidylinisotol
HMM: Hidden Markov Model
LBD: Ligand Binding Domain
PDB: Protein Databank
PSSM: Position-Specific Score Matrix
RBD: Receptor Binding Domain
RTK: Receptor Tyrosine Kinase
SAM: Sterile Alpha Motif
SH-aLRT: Shimodaira–Hasegawa approximate Likelihood Ratio Test
SRS: SAG1-Related Sequence
TGF-β: Transforming Growth Factor-beta
TM: trans-membrane
