# Supplementary material for: The first identification of complete Eph-ephrin signalling in ctenophores and sponges reveals a role for neofunctionalization in the emergence of signalling domains
Source: BMC Evol Biol. 2019 Apr 25;19:96. doi: 10.1186/s12862-019-1418-z (PMC6485061; doi:10.1186/s12862-019-1418-z)
Supplement: Supplementary file 1 — Figure S1. Domain architectures of Eph receptors identified across all analyzed taxa. Figure S2. Multiple sequence alignment containing selected representatives of the Eph-receptor extracellular Ligand Binding Domain (LBD) animals and choanoflagellates. Figure S3. Multiple sequence alignment of the Ephrin ligand Receptor Binding Domain (RBD) containing representatives from animals and choanoflagellates. Figure S4. Evolutionary relationships of metazoan Eph receptors inferred using the canonical intracellular Pkinase-Tyr domain. Figure S5. Evolutionary relationships of metazoan Eph receptors inferred using the extracellular ligand binding domain (LBD). Figure S6. Evolutionary relationships among metazoan ephrin ligands, highlighting multiple expansions in the phylum Cnidaria. Figure S7. Cartoon rendering of representative structures of cupredoxins, ephrins and the SRS superfamily. Figure S8. Structure based sequence alignment of ephrins, monodomain cupredoxins and the SRS superfamily from diverse taxa. Figure S9. Phylogenetic relationships between ephrins, monodomain cupredoxins and SRS superfamily. Table S1. List of analyzed taxa and database sources. Table S2. Dataset utilized to plot the heat map shown in Fig. 1b. Table S3. Distribution of Eph, ephrin and monodomain cupredoxins. Table S4. Mapping of Eph-LBD and Ephrin-RBD interaction interface residues. Table S5. Predicted TM regions and lipid anchoring sites in ephrins from non-bilaterian metazoans. Accompanying note. Genome/transcriptome datasets used for sequence search. Eph-LBD and ephrin hits in choanoflagellates. (PDF 47231 kb) [file 12862_2019_1418_MOESM1_ESM.pdf]

Contents of Additional file 1:

List of contents:

Fig. S1. Fig. S1. Domain architectures of Eph-like receptors identified across all analyzed taxa.

Fig. S2. Multiple sequence alignment containing selected representatives of the Eph-receptor extracellular Ligand Binding Domain (LBD) animals and choanoflagellates.

Fig. S3. Multiple sequence alignment of the Ephrin ligand Receptor Binding Domain (RBD) containing representatives from animals and choanoflagellates

Fig. S4. Evolutionary relationships of metazoan Eph receptors inferred using the canonical intracellular Pkinase-Tyr domain.

Fig. S5. Evolutionary relationships of metazoan Eph receptors inferred using the extracellular ligand binding domain (LBD).

Fig. S6. Evolutionary relationships among metazoan ephrin ligands, highlighting multiple expansions in the phylum Cnidaria.

Fig. S7. Cartoon rendering of representative structures of cupredoxins, ephrins and the SRS superfamily.

Fig. S8. Structure based sequence alignment of ephrins, monodomain cupredoxins and the SRS superfamily from diverse taxa.

Fig. S9. Phylogenetic relationships between ephrins, monodomain cupredoxins and SRS superfamily.

Table S1: List of analyzed taxa and database sources.

Table S2: Dataset utilized to plot the heat map shown in Figure 1B.

Table S3: Distribution of Eph, ephrin and monodomain cupredoxins.

Table S4: Mapping of Eph-LBD-ephrin-RBD interaction interface residues.

Table S5: Prediction of TM regions and lipid anchoring sites in ephrins.

Supplementary notes: 1) Genome/transcriptome datasets used for sequence search 2) Eph_LBD and Ephrin hits in choanoflagellates

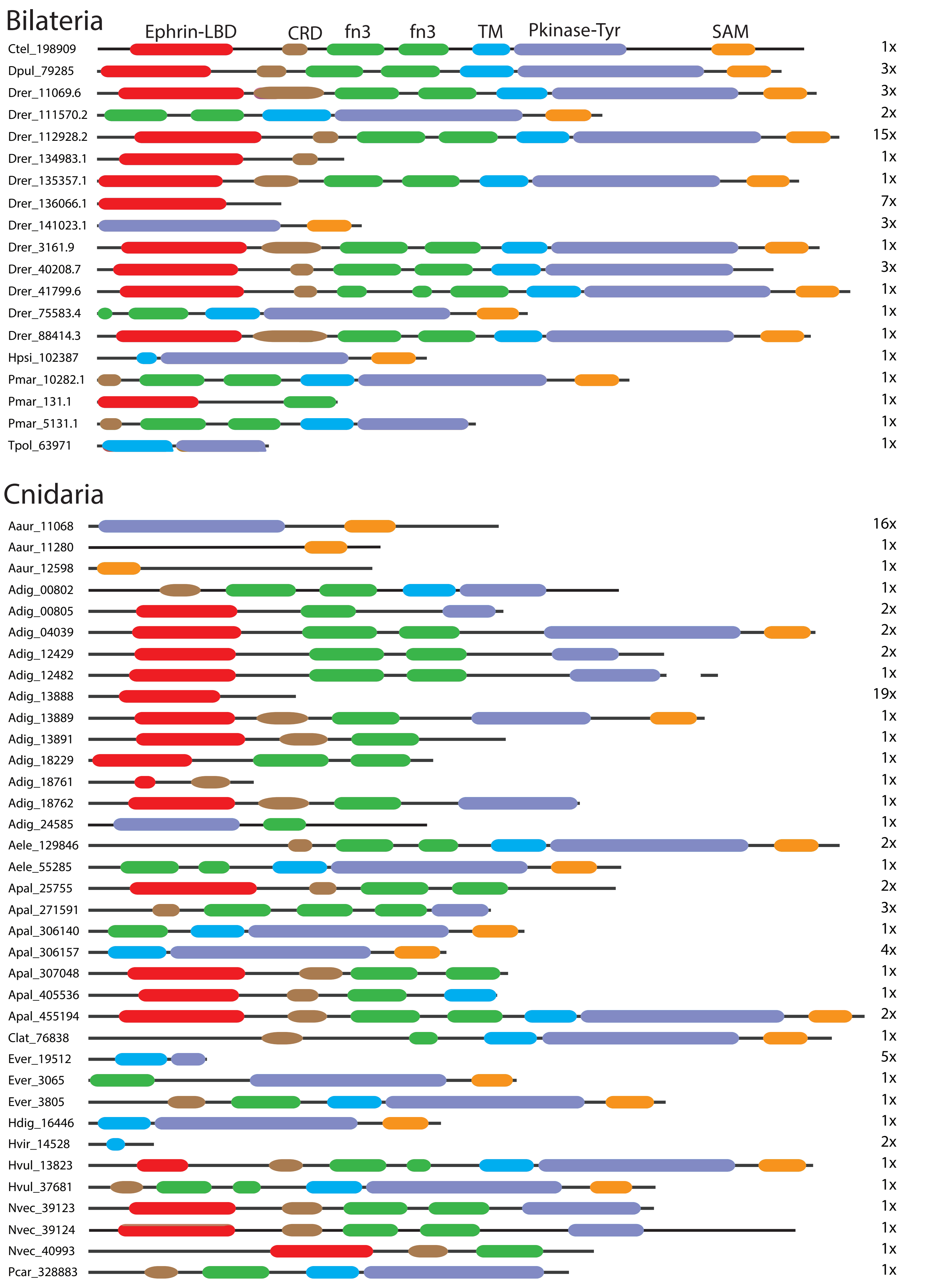
Bilateria
Ephrin-LBD
CRD
fn3
fn3
TM
Pkinase-Tyr
SAM
Ctel_198909 1x
Dpul_79285 3x
Drer_11069.6 3x
Drer_111570.2 2x
Drer_112928.2 15x
Drer_134983.1 1x
Drer_135357.1 1x
Drer_136066.1 7x
Drer_141023.1 3x
Drer_3161.9 1x
Drer_40208.7 3x
Drer_41799.6 1x
Drer_75583.4 1x
Drer_88414.3 1x
Hpsi_102387 1x
Pmar_10282.1 1x
Pmar_131.1 1x
Pmar_5131.1 1x
Tpol_63971 1x
Cnidaria
Aaur_11068 16x
Aaur_11280 1x
Aaur_12598 1x
Adig_00802 1x
Adig_00805 2x
Adig_04039 2x
Adig_12429 2x
Adig_12482 1x
Adig_13888 19x
Adig_13889 1x
Adig_13891 1x
Adig_18229 1x
Adig_18761 1x
Adig_18762 1x
Adig_24585 1x
Aele_129846 2x
Aele_55285 1x
Apal_25755 2x
Apal_271591 3x
Apal_306140 1x
Apal_306157 4x
Apal_307048 1x
Apal_405536 1x
Apal_455194 2x
Clat_76838 1x
Ever_19512 5x
Ever_3065 1x
Ever_3805 1x
Hdig_16446 1x
Hvir_14528 2x
Hvul_13823 1x
Hvul_37681 1x
Nvec_39123 1x
Nvec_39124 1x
Nvec_40993 1x
Pcar_328883 1x

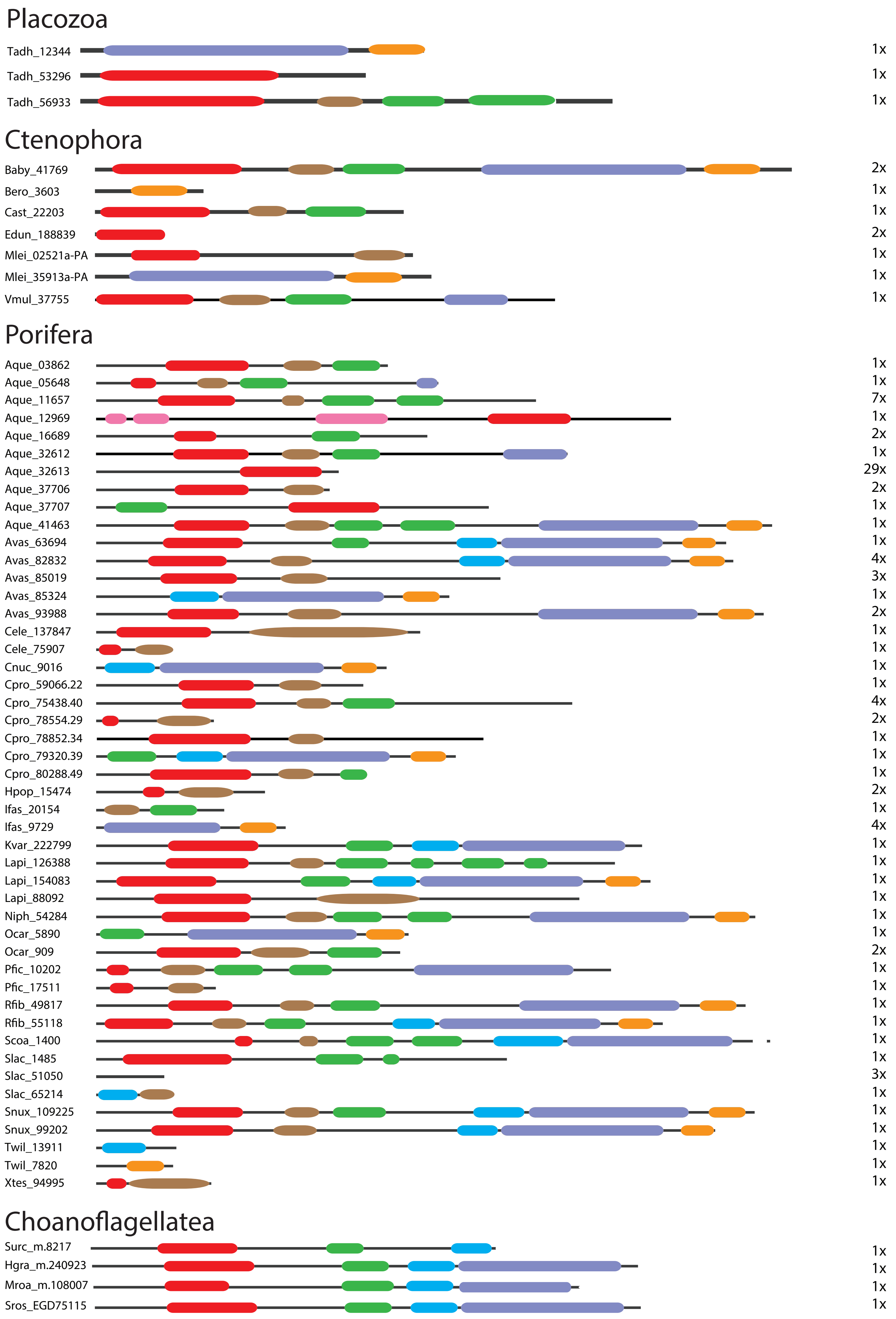

Fig. S1. Domain architectures of Eph-like receptors identified across all analyzed taxa. Inferred protein domain architectures were obtained using HMMER searches against Pfam database and Reverse Position-Specific BLAST searches against the pre-calculated Position-Specific Scoring Matrix (PSSMs) of the Conserved Domain Database (CDD) version 3.14. For choanoflagellate candidates, the domain architectures were estimated through HHpred searches sequence alignments performed in this study (See alignments in main figures and other supplementary figures). The cysteine rich regions of Eph-receptors, flanked between the Ephrin-LBD and the fibronectin domains are generally informed as either Sushi, EGF-like and other cysteine rich domains in Pfam and CDD searches. To be consistent we uniformly denote the region between Ephrin-LBD and fibronectin as CRD (Cysteine rich domain). Eph_LBD, CRD, fn3, TM, Pkinase-Tyr and SAM domains were highlighted in red, brown, green, blue, violet and orange, respectively. The numbers on the right denotes the number of occurrence of such domain architecture within a lineage.

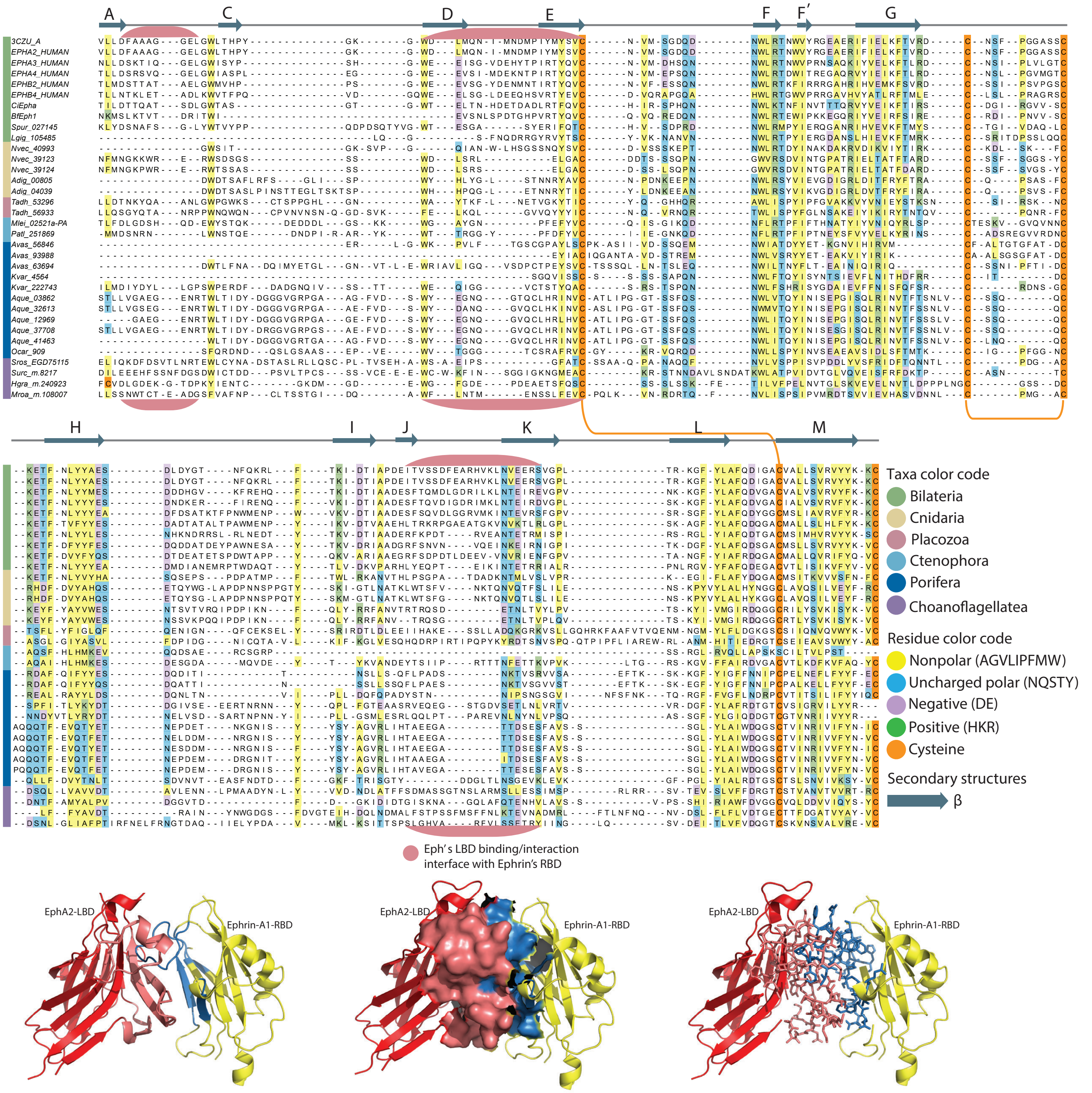

Fig. S2. Multiple sequence alignment of the Eph-receptor extracellular Ligand Binding Domain (LBD) in animals and choanoflagellates. The alignment includes representative sequences from all taxa in which the Eph receptors were identified. Secondary structure elements (only strands are shown) shown above the alignment is based on the LDB domain (Chain-A) of EphA2-EphrinA1 structural complex (3CZU). The coloring is based on 50% consensus threshold using the following scheme: nonpolar residues (AGVLIPFMW) shaded yellow, uncharged polar (NQSTY) shaded light blue, negatively charged (DE) shaded light purple, positively charged (HKR) shaded light green, and cysteines forming disulphide bridges shaded orange. The inset at the bottom show multiple structural rendering of EphA2-EphrinA1 structural complex (PDBID: 3CZU) in which the interaction interface regions of EphA2-LBD and Ephrin-A1-RBD are highlighted in pale red and blue, respectively.

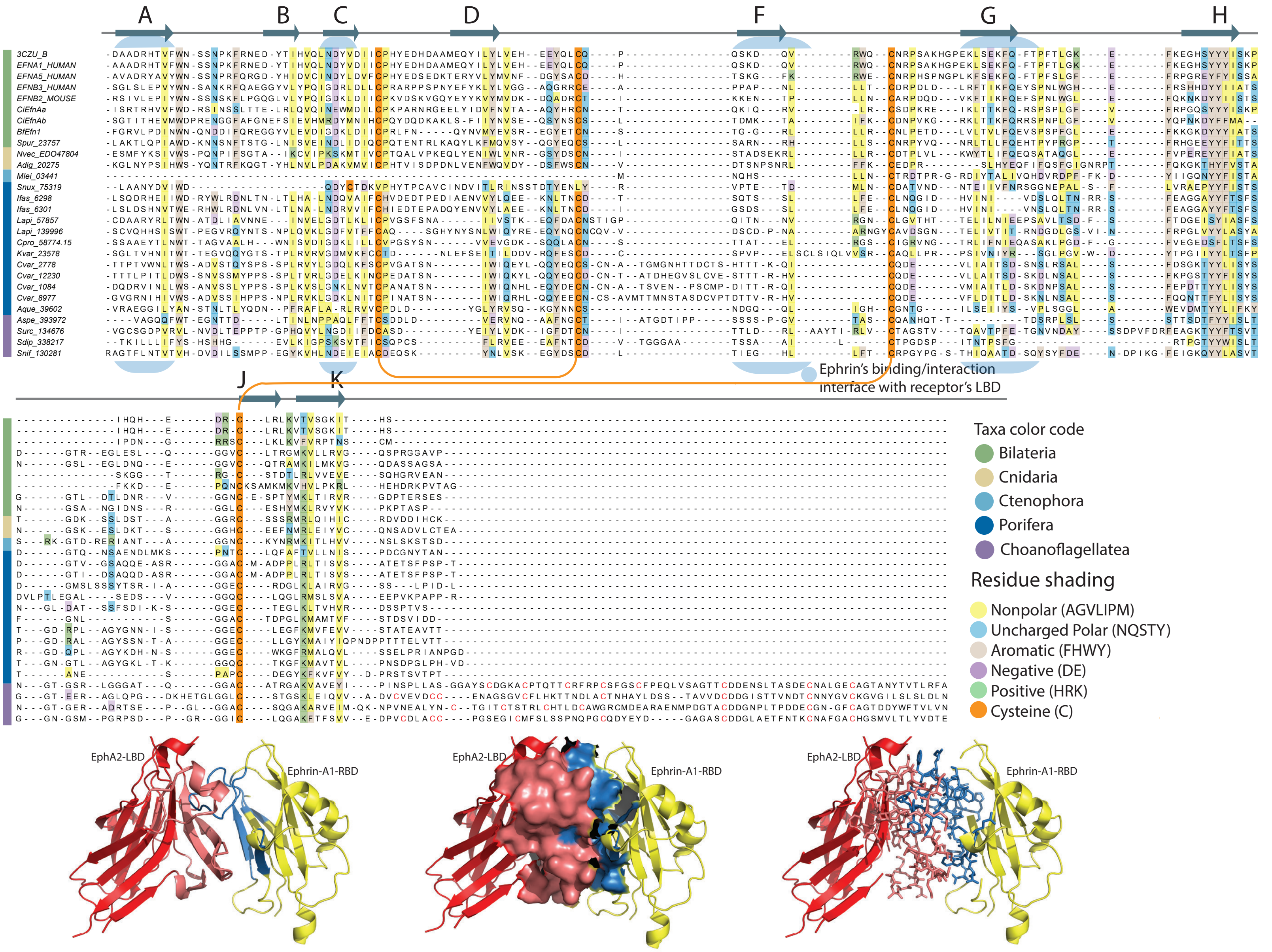

Fig. S3. Multiple sequence alignment of the Ephrin ligand's Receptor Binding Domain (RBD) containing representatives from animals and choanoflagellates.

Secondary structure elements shown above the alignment is based on the RBD domain (Chain-B) of EphA2-EphrinA1 structural complex (3CZU). The coloring is based on 50% consensus threshold using the following scheme: nonpolar residues (AGVLIPFMW) shaded yellow, uncharged polar (NQSTY) shaded light blue, negatively charged (DE) shaded light purple, positively charged (HKR) shaded light green, aromatic (FHWY) and shaded light brown, and cysteines forming disulphide bridges shaded blue. C-terminal ends of the sequences that usually contain the TM anchoring region were chopped off from the final figure, whereas the cysteine-rich region found in the choanoflagellate sequences, occuring prior to the TM helix are shown. The inset on the right show multiple structural rendering of EphA2-EphrinA1 structural complex (PDBID: 3CZU) in which the interaction interface regions of EphA2-LBD and Ephrin-A1-RBD are highlighted in pale red and blue, respectively.

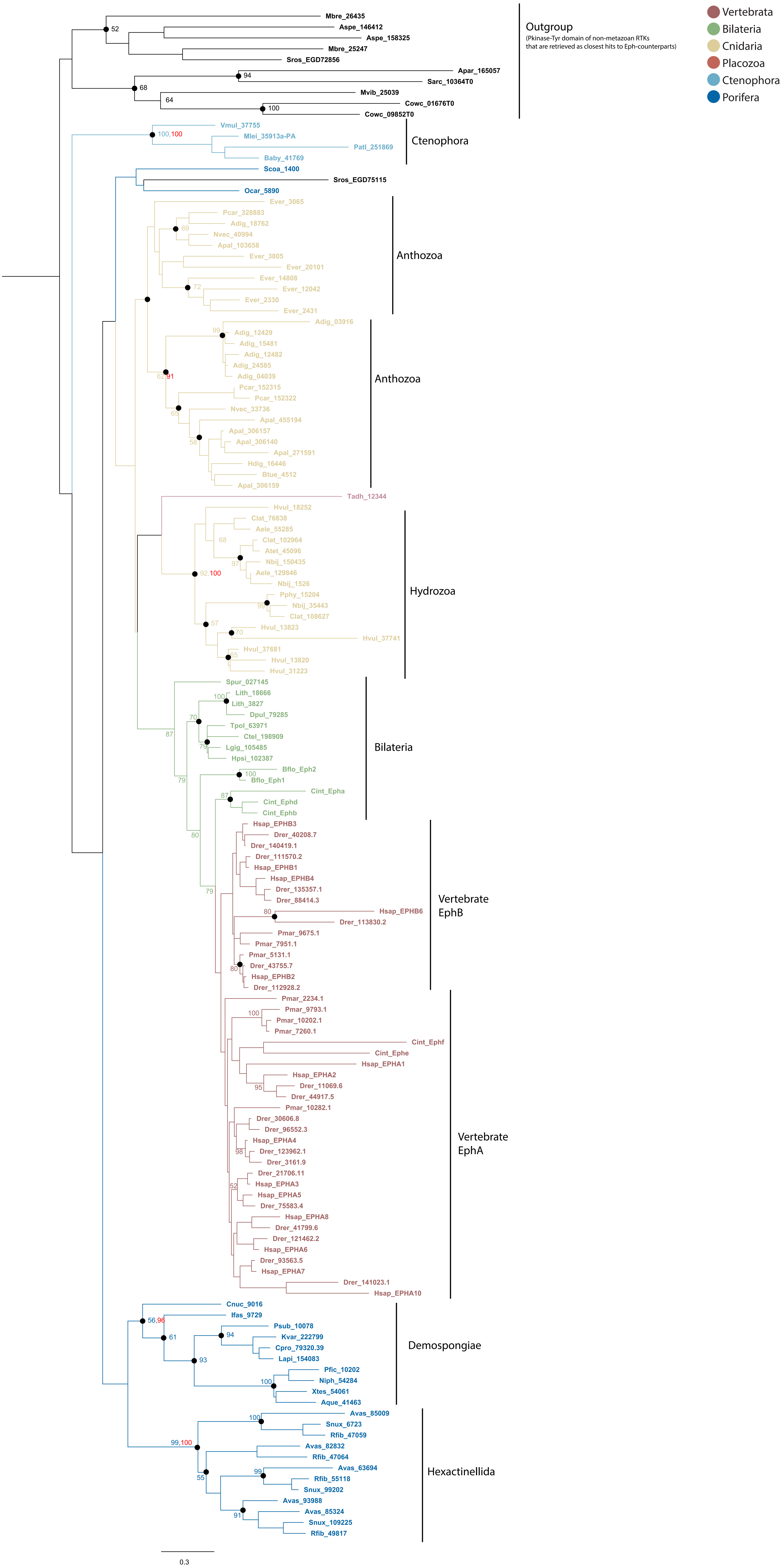

Fig. S4. Evolutionary relationships of metazoan Eph receptors inferred using the canonical intracellular Pkinase-Tyr domain. Pkinase-Tyr domains of closely related non-metazoans RTKs were used as outgroup and rooted on it. The tree topology was inferred using Maximum Likelihood approach using RaxML and the node support was also estimated using Bayesian approach. Bootstrap values of >50% are marked on the key nodes and corresponding Bayesian posterior probability of threshold >90% were shown as black dots. Red text denote branch support values (shown only for a few)) estimated using ultrafast bootstrap method (1000 replicates) as implemented in the IQ-TREE software. For complete confidence values at all internal nodes/branches refer to raw tree files provided in the Additional file 3.

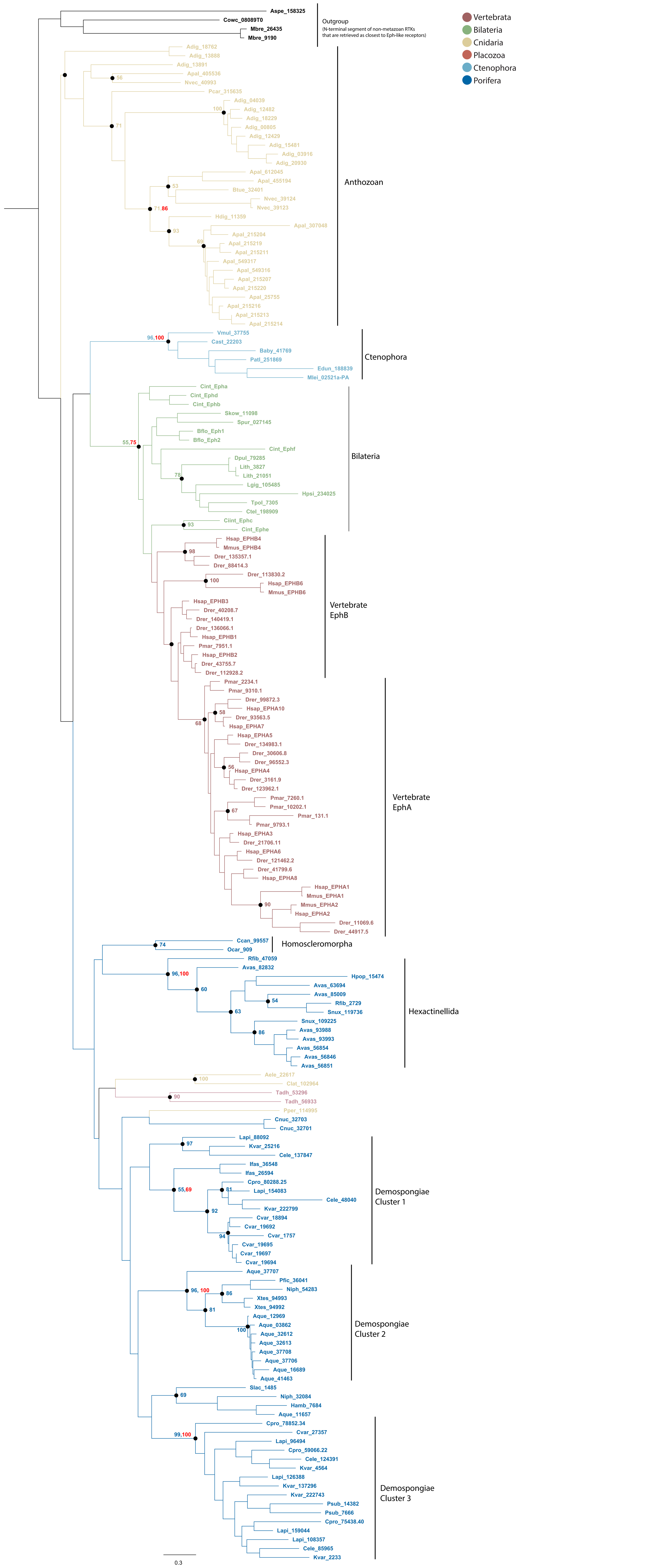

Fig. S5. Evolutionary relationships of metazoan Eph receptors inferred using the extracellular ligand binding domain (LBD). N-terminal regions of closely related non-metazoan RTKs were used as outgroup and rooted on it. The tree topology was inferred using Maximum Likelihood approach using RaxML and the node support was also estimated using Bayesian approach. Bootstrap values of >50% are marked on the key nodes and corresponding Bayesian posterior probability of threshold >90% were shown as black dots. Red text denote branch support values (shown only for a few) estimated using ultrafast bootstrap method (1000 replicates) as implemented in the IQ-TREE software. For complete confidence values at all internal nodes/branches refer to raw tree files provided in the Additional file 3.

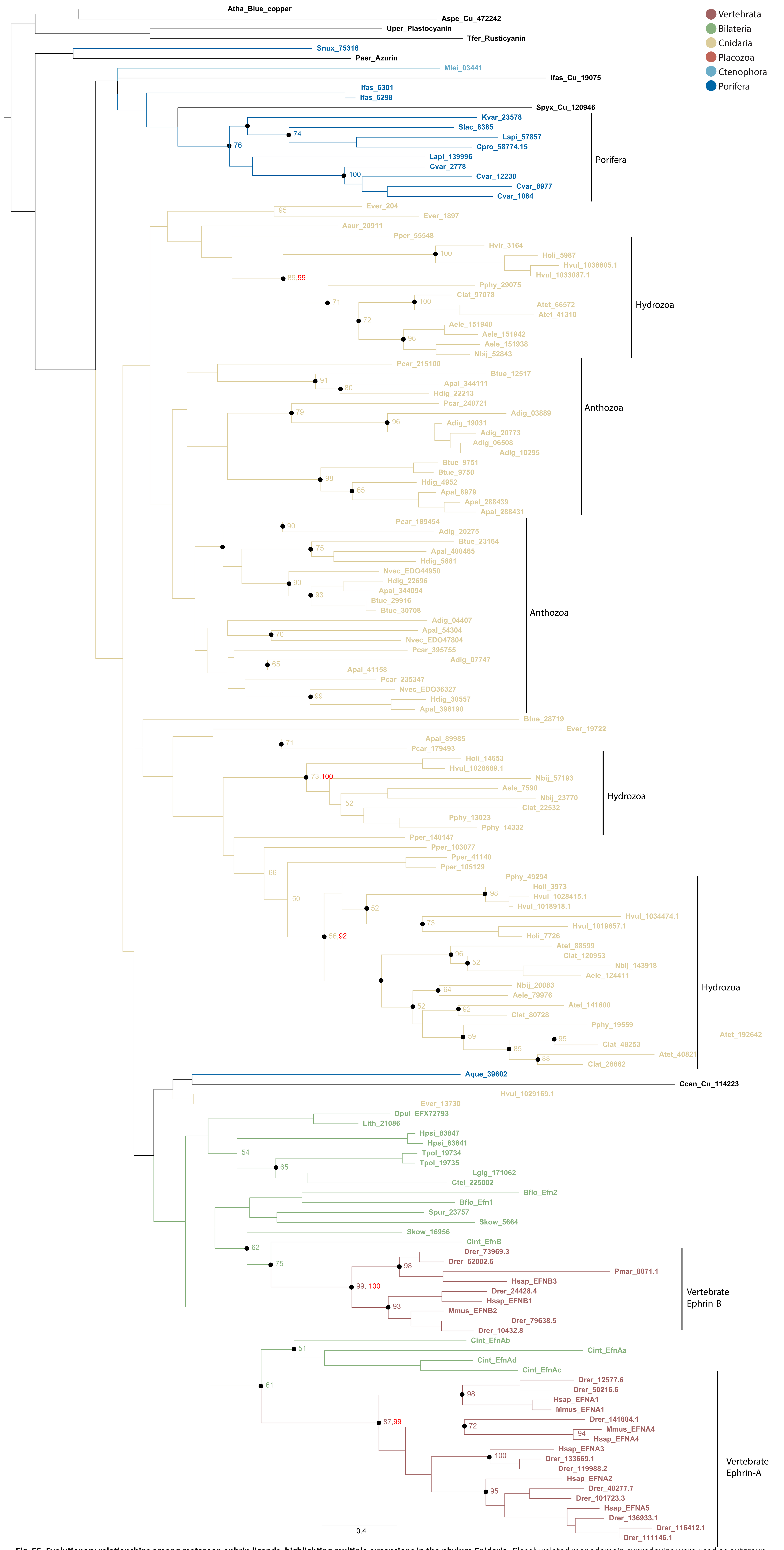

Fig. S6. Evolutionary relationships among metazoan ephrin ligands, highlighting multiple expansions in the phylum Cnidaria. Closely related monodomain cupredoxins were used as outgroup and rooted on it. The tree topology was inferred using Maximum Likelihood approach using RaxML and the node support was also estimated using Bayesian approach. Bootstrap values of >50% are marked on the key nodes and corresponding Bayesian posterior probability of threshold >90% were shown as black dots. Red text denote branch support values (shown only for a few) estimated using ultrafast bootstrap method (1000 replicates) as implemented in the IQ-TREE software. For complete confidence values at all internal nodes/branches refer to raw tree files provided in the Additional file 3.

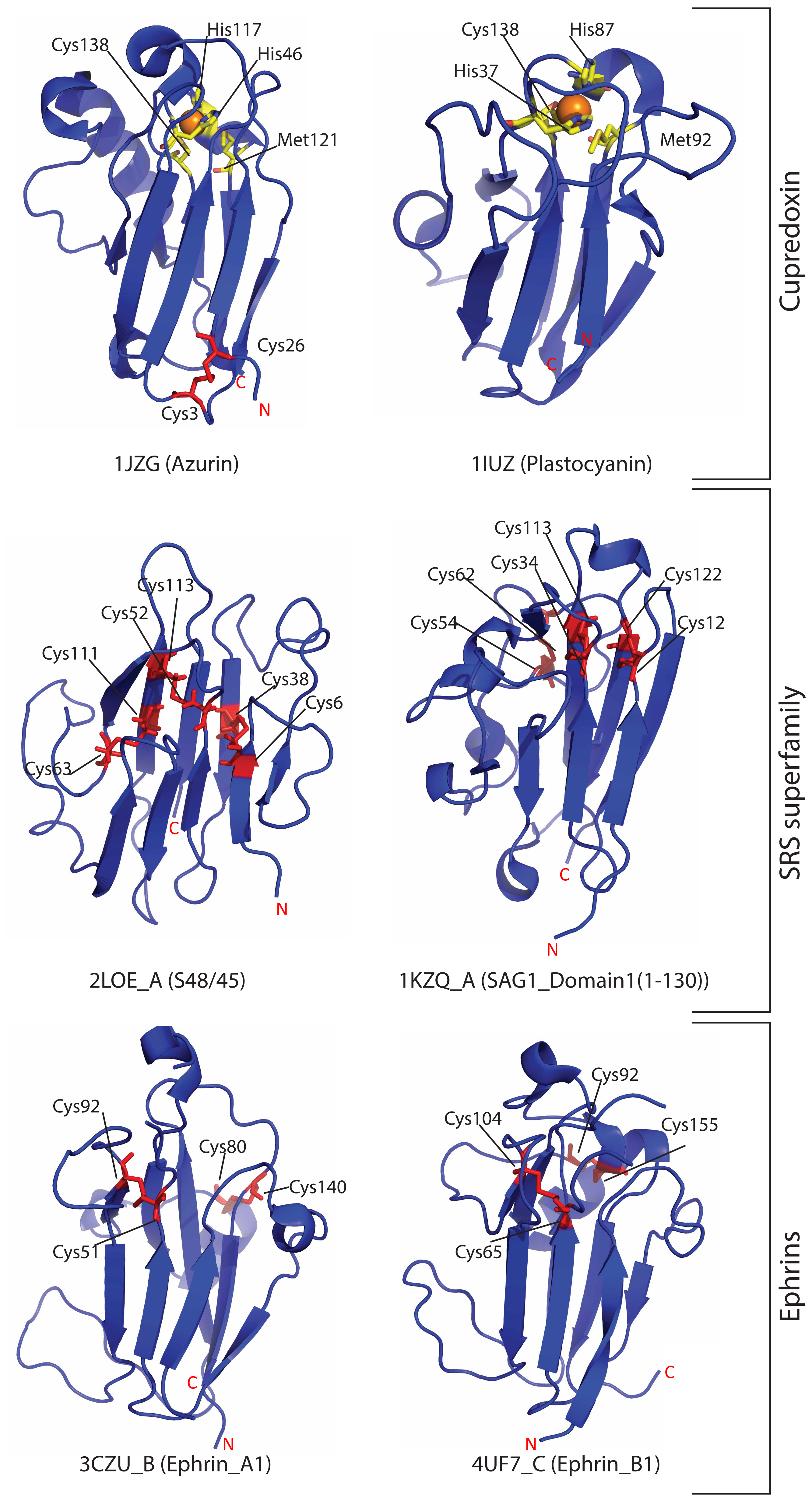

Fig. S7. Cartoon rendering of representative structures of cupredoxins, ephrins and the SRS superfamily. Residues involved in chelation of Copper (Orange Sphere) is highlighted in yellow. Cysteines forming disulphide bridges are highlighted in red in all structures. Cartoon rendering are shown approximately in the same orientation to show the common β-sandwich structural core shared across these families. The region of variability extending after strand 4 is on the left of all orientations.

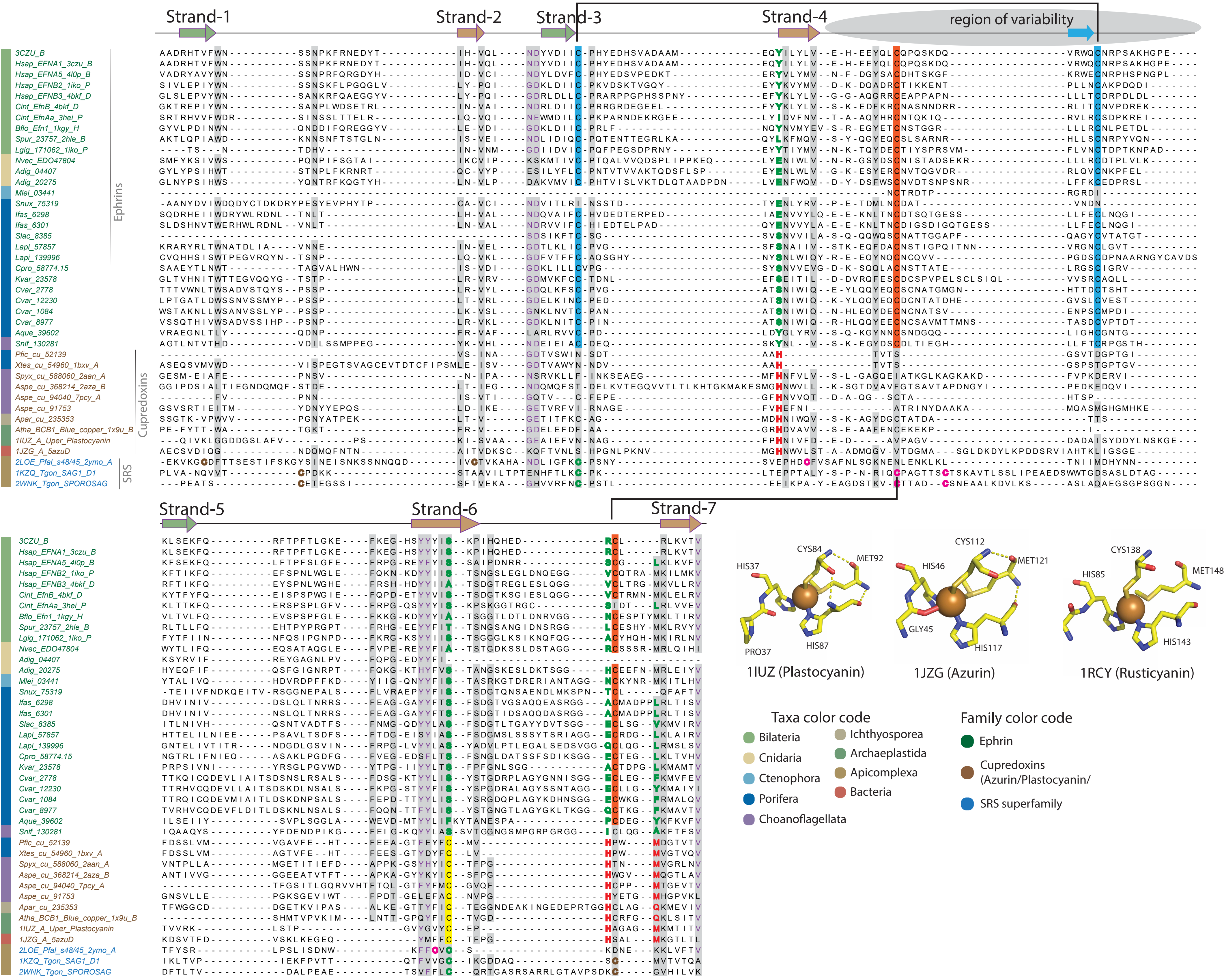

Fig. S8. Structure based sequence alignment of ephrins, monodomain cupredoxins and the SRS superfamily from diverse taxa. The alignment illustrates the homologous β-strands forming the common β-sandwich structural core across families. The alignment includes a few structural representatives of ephrins (3CZU_b; 4M4R_b; 2HLE_b; 4BKF_c), monodomain cupredoxins (azurin (1JZG_a) and from bacteria; plastocyanin (1IUZ_a) and blue copper protein (1X9U_b) from plants) and SRS superfamily (s48/45 domain (2LOE), SAG1-D1 (1KZQ) and SPOROSAG (2WNK) from apicomplexan). Aligned columns >40% consensus threshold are highlighted. The strands are mapped on top of the alignment based on (3CZU_B) and colored same as panel A in Fig.5, while numbers are marked only for strands homologous across families. Cysteines forming disulphide linkages for ephrins are highlighted in blue and orange background. The homologous SRS family domains have diversified disulphide bridges and cysteine partners involved in forming disulphide bridges are colored brown, green and blue. Cysteine at the top of penultimate strand and that form a dusulphide linkage with the cysteine at the top of strand-3 in the SRS family, is structurally equivalent to the cysteine that chelates the copper in cupredoxins. Residues involved in chelating copper in cupredoxins are highlighted in red/yellow, while the non-conservative substitutions at the structurally equivalent positions possibly resulting in the loss of copper binding ability in ephrins are highlighted in green. The inset at the right illustrates residues involved in chelation of copper (Orange Sphere).

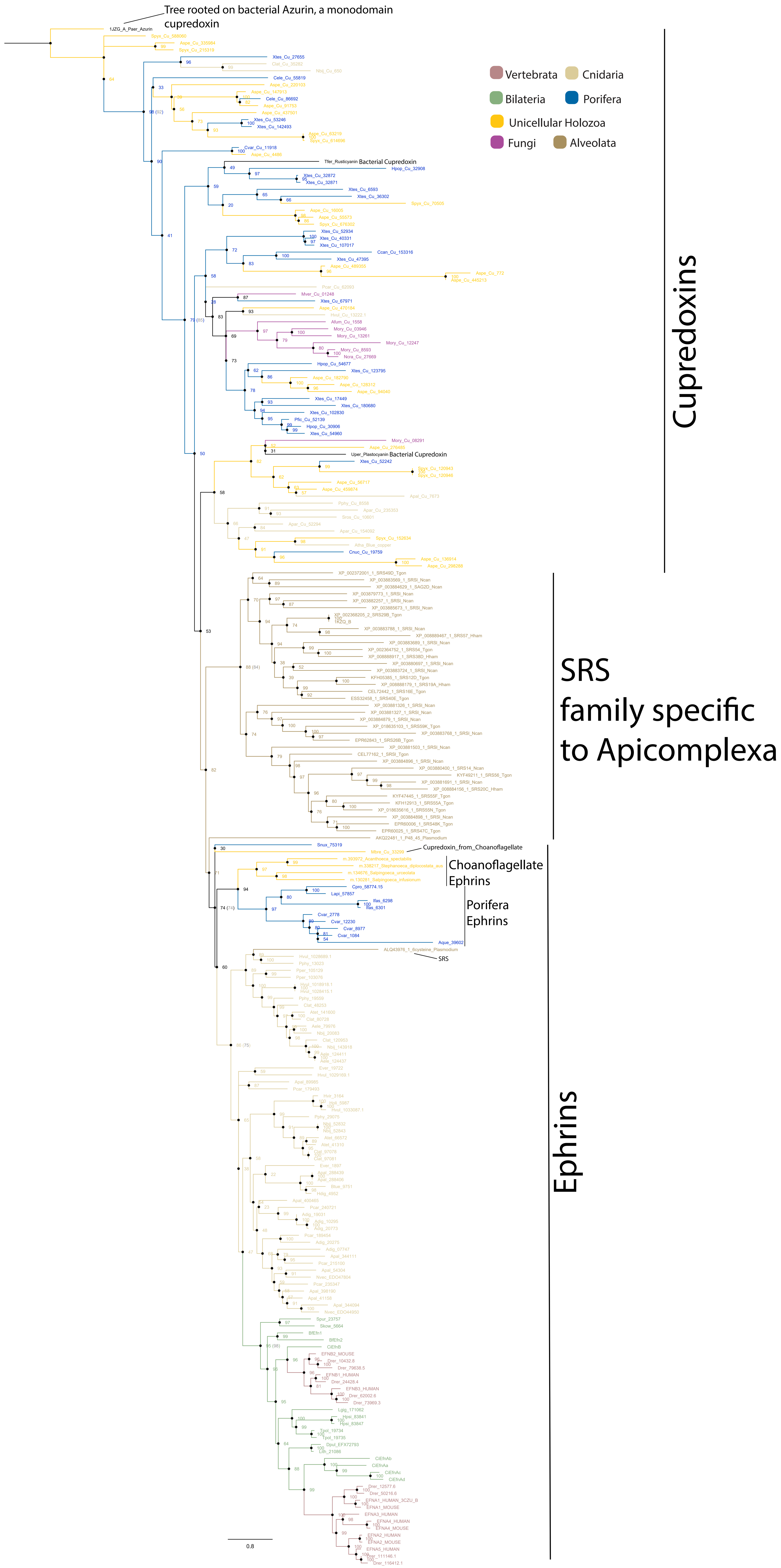

Fig. S9. Phylogenetic relationships between ephrins, monodomain cupredoxins and SRS superfamily. Tree topology was inferred using maximum-likelihood approach with ultrafast bootstrap method (1000 replicates) as implemented in the IQ-TREE software. Bacterial monodomain cupredoxins were used as outgroup and root. Percentage support values of 1000 replicates were shown for all nodes. Additionaly, the tree topology was tested maximum-likelihood method implemented in the FastTree program. Support values obtained using FastTree program are shown for important nodes (shown inside curved parentheses. For complete confidence values at all internal nodes/branches refer to raw tree files provided in the Additional file 3.

Table S1: List of analysed taxa and database sources

Taxonomic group	Species	Species Abbrev.	Dataset type	Source database	Accession
Amoebozoa	*D. discoideum*	*Ddis*	Whole genome	Ensembl Protists (v31)	
Fungi					
Blastocladiomycota	*A. macrogynus*	*Amac*	Whole genome	Ensembl Fungi (v31)	
Ascomycota	*A. fumigatus*	*Afum*	Whole genome	Ensembl Fungi (v31)	
	N. crassa	*Ncra*	Whole genome	Ensembl Fungi (v31)	
	S. cerevisiae	*Scer*	Whole genome	Ensembl Fungi (v31)	
	M. oryzae	*Mory*	Whole genome	Ensembl Fungi (v31)	
Basidiomycota	*U. maydis*	*Umay*	Whole genome	Ensembl Fungi (v31)	
Mucoromycota	*M. verticillata*	*Mver*	Whole genome	Ensembl Fungi (v31)	
	R. delemar	*Rdel*	Whole genome	Ensembl Fungi (v31)	
Chytridiomycota	*S. punctatus*	*Spun*	Whole genome	Ensembl Fungi (v31)	
Unicellular Holozoa					
Ichthyosporea	*S. arctica*	*Sarc*	Whole genome	Broad Institute	
	A. parasiticum	*Apar*	Transcriptome	Broad institute	
Filasterea	*M. vibrans*	*Mvib*	Transcriptome	NCBI SRA	SRR343051
	C. owczarzaki	*Cowc*	Whole Genome	Broad institute	
Choanoflagellatea	*S. rosetta*	*Sros*	Whole Genome	Ensembl protists (v31)	
	S. pyxidium	*Spyx*	Transcriptome	NCBI SRA	SRR1915749
	M. ovata	*Mova*	Transcriptome	dbEST	
	M. brevicollis	*Mbre*	Whole Genome	JGI	
	A. spectabilis	*Aspe*	Transcriptome	NCBI SRA	SRR1915695: SRR6344971
	D. grandis	*Dgra*	Transcriptome	NCBI SRA	SRR6344970
	S. punica	*Spun*	Transcriptome	NCBI SRA	SRR6344976
	S. urceolata	*Surc*	Transcriptome	NCBI SRA	SRR6344979
	H. gracilis	*Hgra*	Transcriptome	NCBI SRA	SRR6344977
	S. macrocollata	*Smac*	Transcriptome	NCBI SRA	SRR6344984
	S. diplocostata	*Sdip*	Transcriptome	NCBI SRA	SRR6344980; SRR6344982
	H. nana	*Hnan*	Transcriptome	NCBI SRA	SRR6344981
	S. kvevrii	*Skve*	Transcriptome	NCBI SRA	SRR6344978
	D. costata	*Dcos*	Transcriptome	NCBI SRA	SRR6344966
	C. perplexa	*Cper*	Transcriptome	NCBI SRA	SRR6344967
	S. infusionum	*Sinf*	Transcriptome	NCBI SRA	SRR6344968
	M. roanoka	*Mroa*	Transcriptome	NCBI SRA	SRR6344969
	S. parva	*Spar*	Transcriptome	NCBI SRA	SRR6344983
	H. balthica	*Hbal*	Transcriptome	NCBI SRA	SRR6344985
	S. dolichothecata	*Sdol*	Transcriptome	NCBI SRA	SRR6344972
	C. hollandica	*Chol*	Transcriptome	NCBI SRA	SRR6344973
	S. helianthica	*Shel*	Transcriptome	NCBI SRA	SRR6344974
	M. fluctuans	*Mflu*	Transcriptome	NCBI SRA	SRR6344975
Metazoa					
Porifera					
Hexactinellida	*H. populiferum*	*Hpop*	Transcriptome	NCBI SRA	SRR1916923
	S. nux	*Snux*	Transcriptome	NCBI SRA	SRR1916581
	R. fibulata	*Rfib*	Transcriptome	NCBI SRA	SRR1915835

	A. vastus	*Avas*	Transcriptome	Evolution & Research Archive	
Demospongiae	*I. fasciculata*	*Ifas*	Transcriptome	Dataverse Network	
	C. nucula	*Cnuc*	Transcriptome	Dataverse Network	
	S. lacustris	*Slac*	Transcriptome	NCBI SRA	SRR1168575
	E. muelleri	*Emue*	Transcriptome	NCBI SRA	SRR1041944
	L. apicalis	*Lapi*	Transcriptome	NCBI SRA	SRR1915755
	C. prolifera	*Cpro*	Transcriptome	Degnan Lab	
	C. elegans	*Cele*	Transcriptome	Dryad	doi: 10.5061/dryad.50dc6/3
	K. variolosa	*Kvar*	Transcriptome	NCBI SRA	SRR1916957
	P. suberitoides	*Psub*	Transcriptome	Dataverse Network	
	T. wilhelma	*Twil*	Transcriptome	NCBI SRA	ERR216193
	C. varians	*Cvar*	Transcriptome	NCBI SRA	SRR1391159
	P. ficiformis	*Pfic*	Transcriptome	Dataverse Network	
	Niphatidae indet	*Niph*	Transcriptome	Degnan Lab	
	X. testudinaria	*Xtes*	Transcriptome	NCBI SRA	SRR1738101
	H. amboinensis	*Hamb*	Transcriptome	NCBI SRA	SRR1619429
	A. queenslandica	*Aque*	Whole Genome	Aqu2.1 dataset http://amphimedon.qcloud.qcif.edu.au/downloads.html	
Calcarea	*S. coactum*	*Scoa*	Transcriptome	Dataverse Network	
	S. ciliatum	*Scil*	Whole Genome	Compagen database (T-PEP dataset)	SCIL_T-PEP_130802
Homoscleromorpha	*O. carmela*	*Ocar*	Whole Genome	Compagen database (T-PEP dataset)	OCAR_T-PEP_130911
	C. candelabrum	*Ccan*	Transcriptome	Dataverse Network	
Ctenophora					
	E. dunlapae	*Edun*	Transcriptome	NCBI SRA	SRR777663
	C. astericola	*Cast*	Transcriptome	NCBI SRA	SRR786490
	V. multiformis	*Vmul*	Transcriptome	NCBI SRA	SRR786489
	P. bachei	*Pbac*	Whole Genome	neurobase.rc.ufl.edu/pleurobrachia	
	Pleurobrachia sp	*Pleu*	Transcriptome	NCBI SRA	SRR789901
	D. glandiformis	*Dgla*	Transcriptome	NCBI SRA	SRR777788
	B. abyssicola	*Baby*	Transcriptome	NCBI SRA	SRR777787
	Beroe sp	*Bero*	Transcriptome	NCBI SRA	ERR216194
	M. leidyi	*Mlei*	Whole Genome	Ensembl Metazoa (v31)	
	B. infundibulum	*Binf*	Transcriptome	NCBI SRA	SRR786491
	Mertensiidae sp	*Mert*	Transcriptome	NCBI SRA	SRR786492
Placozoa					
	T. adhearens	*Tadh*	Whole Genome	Ensembl metazoa (v31)	
Cnidaria					
Anthozoa	*E. verrucosa*	*Ever*	Transcriptome	NCBI SRA	SRR1324944 SRR1324945
	A. digitifera	*Adig*	Whole Genome	OIST Marine genomics Unit	
	P. carnosa	*Pcar*	Transcriptome	NCBI SRA	SRR402974 SRR402975

	N. vectensis	*Nvec*	Whole Genome	Ensembl metazoa (v31)	
	B. tuediae	*Btue*	Transcriptome	NCBI SRA	SRR504347
	A. pallida	*Apal*	Transcriptome	NCBI SRA	SRR696721<br>SRR696732<br>SRR696745
	H. digitata	*Hdig*	Transcriptome	NCBI SRA	SRR504348
Scyphozoa	*A. aurita*	*Aaur*	Transcriptome	NCBI SRA	SRR040475<br>SRR040476<br>SRR040477<br>SRR040478<br>SRR040479
	P. periphylla	*Pper*	Transcriptome	NCBI SRA	SRR1915828
Hydrozoa	*H. viridissima*	*Hvir*	Transcriptome	NCBI SRA	SRR040470<br>SRR040471<br>SRR040472<br>SRR040473
	H. vulgaris	*Hvul*	Transcriptome	NCBI SRA	SRR2051265
	H. oligactis	*Holi*	Transcriptome	NCBI SRA	SRR040466<br>SRR040467<br>SRR040468<br>SRR040469
	A. tetragona	*Atet*	Transcriptome	NCBI SRA	SRR871525
	C. lathetica	*Clat*	Transcriptome	NCBI SRA	SRR871529
	N. bijuga	*Nbij*	Transcriptome	NCBI SRA	SRR871527
	A. elegans	*Aele*	Transcriptome	NCBI SRA	SRR871526
	P. physalis	*Pphy*	Transcriptome	NCBI SRA	SRR871528
Bilateria					
Protostomia	*Priapulus sp*	*Pria*	Transcriptome	NCBI SRA	SRR1611567
	Lithobius sp	*Lith*	Transcriptome	NCBI SRA	SRR1159752
	D. pulex	*Dpul*	Whole Genome	Ensembl (v85)	
	H. psittacea	*Hpsi*	Transcriptome	NCBI SRA	SRR1611556
	T. polymorphus	*Tpol*	Transcriptome	NCBI SRA	SRR1611583
	L. gigantea	*Lgig*	Whole Genome	Ensembl (v85)	
	C. teleta	*Ctel*	Whole Genome	Ensembl (v85)	
Deuterostomia	*S. purpuratus*	*Spur*	Whole Genome	Ensembl (v85)	
	S. kowalevskii	*Skow*	Whole Genome	Ensembl (v85)	
	P. marinus	*Pmar*	Whole Genome	Ensembl (v85)	
	D. rerio	*Drer*	Whole Genome	Ensembl (v85)	
	M. musculus	*Mmus*	Whole Genome	SwissProt	
	H. sapiens	*Hsap*	Whole Genome	SwissProt	

Note: Broad Taxonomic supergroups are coloured red, while the sub-taxonomic groups are coloured brown and further divisions are coloured blue.

Full names of species listed in the Table S1.

Bilateria: *Homo sapiens; Mus musculus; Danio rerio; Petromyzon marinus; Saccoglossus kowalevskii; Strongylocentrotus purpatus; Capitella teleta; Lottia gigantea; Tubulanus polymorphus; Hemithris psittacea; Daphnia pulex; Lithobius sp; Priapulus sp.*
Cnidaria: *Eunicella verrucosa; Acropora digitifera; Platygyra carnosa; Nematostella vectensis; Bolocera tuediae; Aiptasia pallida; Hormathia digitata; Aurelia aurita; Periphylla periphylla; Hydra viridissima; Hydra vulgaris; Hydra oligactis; Abylopsis tetragona; Craseoa lathetica; Nanomia bijuga; Agalma elegans; Physalia physalis.*
Placozoa: *Trichoplax adhearens.*
Ctenophora: *Beroe abyssicola; Beroe sp; Bolinopsis infundibulum; Coeloplana astericola;*

Dryodora glandiformis; Euplokamis dunlapae; Mertensiidae sp; Mnemiopsis leidyi; Pleurobrachia atlantica; Pleurobrachia bachei; Vallicula multiformis.
Porifera: *Hyalonema populiferum; Sympagella nux; Rossella _bulata; Aphrocallistes vastus; Ircinia fasciculata; Chondrilla nucula; Spongilla lacustris; Ephydatia muelleri; Latrunculia apicalis; Clathria prolifera; Crella elegans; Kirkpatrickia variolosa; Pseudospongosorites suberitoides; Tethya wilhelma; Cliona varians; Petrosia _ciformis; Niphatidae indet; Xestospongia testudinaria; Haliclona amboinensis; Amphimedon queenslandica; Sycon coactum; Sycon ciliatum; Oscarella carmela; Corticium candelabrum;*
Unicellular holozoa: *Acanthoeca spectabilis; Monosiga brevicollis; Monosiga ovata; Salpingoeca pyxidium; Salpingoeca rosetta; Capsaspora owczarzaki; Ministeria vibrans; Amoebidium parasiticum; Sphaeroforma arctica; Diaphanoeca grandis; Salpingoeca punica; Salpingoeca urceolata; Hartaetosiga gracilis; Salpingoeca macrocollata; Stephanoeca diplocostata; Stephanoeca diplocostata; Helgoeca nana; Salpingoeca kvevrii; Didymoeca costata; Choanoeca perplexa; Salpingoeca infusionum; Microstomoeca roanoka; Savillea parva; Hartaetosiga balthica; Salpingoeca dolichothecata; Codosiga hollandica; Salpingoeca helianthica; Mylnosiga fluctuans*
Fungi: *Allomyces macrogynus; Aspergillus fumigatus; Mortierella verticillata; Neurospora crassa; Rhizopus delemar; Saccharomyces cerevisiae; Spizellomyces punctatus; Magnaporthe oryzae; Ustilago maydis.*
Amoebozoa: *Dictyostelium discoideum.*

Table S2: Dataset utilized to plot the heat map shown in Figure 1B.

Species	Eph-receptor-like	Eph-receptor-like (FL)	Ephrin-like
Danio_rerio	26	19	15
Petromyzon_marinus	10	5	1
Saccoglossus_kowalevskii	1	0	2
Strongylocentrotus_purpatus	1	1	1
Capitella_teleta	1	1	1
Lottia_gigantea	1	1	1
Tubulanus_polymorphus	2	0	2
Hemithris_psittacea	2	0	2
Daphnia_pulex	1	1	1
Lithobius sp	3	1	1
Priapulus_sp	0	0	0
Eunicella verrucosa	9	0	4
Acropora digitifera	15	10	8
Platygyra carnosa	4	0	5
Nematostella vectensis	5	3	3
Bolocera tuediae	3	0	6
Aiptasia pallida	20	1	10
Hormathia digitata	2	0	6
Aurelia aurita	3	0	1
Periphylla periphylla	4	0	5
Hydra viridissima	1	0	1
Hydra vulgaris	7	1	8
Hydra oligactis	0	0	3
Abylopsis tetragona	1	0	5
Craseoa lathetica	3	1	7
Nanomia bijuga	3	0	6
Agalma elegans	3	1	7
Physalia physalis	2	0	5
Trichoplax adhearens	3	0	0
Beroe abyssicola	1	1	0
Beroe sp	1	0	0
Bolinopsis infundibulum	0	0	0
Coeloplana astericola	1	0	0
Dryodora glandiformis	0	0	0
Euplokamis dunlapae	1	0	0
Mertensiidae sp	0	0	0
Mnemiopsis leidyi	2	0	1
Pleurobrachia atlantica	2	1	0
Pleurobrachia bachei	0	0	0
Vallicula multiformis	1	1	0
Hyalonema populiferum	1	0	0
Sympagella nux	4	2	0
Rossella fibulata	5	4	0
Aphrocallistes vastus	11	5	0
Ircinia fasciculata	6	0	2

Chondrilla nucula	3	0	0
Spongilla lacustris	6	0	0
Ephydatia muelleri	0	0	0
Latrunculia apicalis	6	1	1
Clathria prolifera	7	0	0
Crella elegans	6	0	0
Kirkpatrickia variolosa	7	1	0
Pseudospongosorites suberitoides	3	0	0
Tethya wilhelma	2	0	0
Cliona varians	7	0	4
Petrosia ficiformis	3	0	0
Niphatidae sp	3	1	0
Xestospongia testudinaria	4	0	0
Haliclona amboinensis	1	0	0
Amphimedon queenslandica	11	2	0
Sycon coactum	1	1	0
Sycon ciliatum	0	0	0
Oscarella carmela	2	0	0
Corticium candelabrum	1	0	1
Acanthoeca spectabilis	0	0	1
Salpingoeca urceolata	1	0	1
Stephanoeca diplocostata(aus)	0	0	1
Salpingoeca infusionum	0	0	1
Salpingoeca rosetta	1	0	0
Capsaspora owczarzaki	0	0	0
Ministeria vibrans	0	0	0
Amoebidium parasiticum	0	0	0
Sphaeroforma arctica	0	0	0
Allomyces macrogynus	0	0	0
Aspergillus fumigatus	0	0	0
Mortierella verticillata	0	0	0
Neurospora crassa	0	0	0
Rhizopus delemar	0	0	0
Saccharomyces cerevisiae	0	0	0
Spizellomyces punctatus	0	0	0
Magnaporthe oryzae	0	0	0
Ustilago maydis	0	0	0
Dictyostelium discoideum	0	0	0

Table S3: Distribution of Eph, ephrin and monodomain cupredoxins across all analysed taxa

Taxonomic group	*Species*	*No of Eph*	*No of FL-Eph*	*No of Ephrins*	*No of monodomain cupredoxins*
**Amoebozoa**	*D. discoideum*	0	0	0	0
**Fungi**					
**Blastocladiomycota**	*A. macrogynus*	0	0	0	0
**Ascomycota**	*A. fumigatus*	0	0	0	2
	N. crassa	0	0	0	3
	S. cerevisiae	0	0	0	0
	M. oryzae	0	0	0	7
**Basidiomycota**	*U. maydis*	0	0	0	0
**Mucoromycota**	*M. verticillata*	0	0	0	3
	R. delemar	0	0	0	0
**Chytridiomycota**	*S. punctatus*	0	0	0	2
**Unicellular Holozoa**					
**Ichthyosporea**	*S. arctica*	0	0	0	0
	A. parasiticum	0	0	0	3
**Filasterea**	*M. vibrans*	0	0	0	1
	C. owczarzaki	0	0	0	0
**Choanoflagellatea**	*S. rosetta*	1	0	0	1
	S. pyxidium	0	0	0	20
	M. ovata	0	0	0	0
	M. brevicollis	0	0	0	1
	A. spectabilis	0	0	1	40
	D. grandis	0	0	0	0
	S. punica	0	0	0	0
	S. urceolata	1	0	1	4
	H. gracilis	1	0	0	0
	S. macrocollata	0	0	0	0
	S. diplocostata	0	0	1	0
	H. nana	0	0	0	10
	S. kvevrii	0	0	0	1
	D. costata	0	0	0	4
	C. perplexa	0	0	0	1
	S. infusionum	0	0	1	0
	M. roanoka	1	0	0	1
	S. parva	0	0	0	1
	H. balthica	0	0	0	1
	S. dolichothecata	0	0	0	2
	C. hollandica	0	0	0	8
	S. helianthica	0	0	0	0
	M. fluctuans	0	0	0	8
**Metazoa**					
**Porifera**					
Hexactinellida	*H. populiferum*	1	0	0	5
	S. nux	4	2	1	0
	R. fibulata	5	4	0	0

	A. vastus	11	5	0	1
Demospongiae	*I. fasciculata*	6	0	2	1
	C. nucula	3	0	0	2
	S. lacustris	6	0	1	0
	E. muelleri	0	0	0	0
	L. apicalis	6	1	2	0
	C. prolifera	7	0	1	1
	C. elegans	6	0	0	2
	K. variolosa	7	1	1	1
	P. suberitoides	3	0	0	0
	T. wilhelma	2	0	0	0
	C. varians	7	0	4	2
	P. ficiformis	3	0	0	3
	Niphatidae sp	3	1	0	1
	X. testudinaria	4	0	0	33
	H. amboinensis	1	0	0	1
	A. queenslandica	10	2	1	0
Calcarea	*S. coactum*	1	1	0	1
	S. ciliatum	0	0	0	1
Homoscleromorpha	*O. carmela*	2	0	0	1
	C. candelabrum	1	0	1	6
Ctenophora					
	E. dunlapae	1	0	0	0
	C. astericola	1	0	0	0
	V. multiformis	1	1	0	0
	P. bachei	0	0	0	0
	P. atlantica	2	1	0	3
	D. glandiformis	0	0	0	0
	B. abyssicola	1	1	0	0
	Beroe sp	1	0	0	0
	M. leidyi	2	0	1	0
	B. infundibulum	0	0	0	1
	Mertensiidae sp	0	0	0	0
Placozoa					
	T. adhearens	3	0	0	0
Cnidaria					
Anthozoa	*E. verrucosa*	9	0	4	0
	A. digitifera	15	10	8	0
	P. carnosa	4	0	6	2
	N. vectensis	5	3	3	0
	B. tuediae	3	0	7	0
	A. pallida	20	1	11	1
	H. digitata	2	0	5	0
Scyphozoa	*A. aurita*	3	0	1	0
	P. periphylla	4	0	6	0
Hydrozoa	*H. viridissima*	1	0	1	0
	H. vulgaris	7	1	9	1
	H. oligactis	0	0	4	0
	A. tetragona	1	0	7	1

	C. lathetica	3	1	7	1
	N. bijuga	3	0	6	1
	A. elegans	3	1	9	0
	P. physalis	2	0	5	1
Bilateria					
Protostomia	*Priapulus sp*	0	0	0	1
	Lithobius sp	3	1	1	0
	D. pulex	1	1	1	0
	H. psittacea	2	0	2	1
	T. polymorphus	2	0	2	0
	L. gigantea	1	1	1	0
	C. teleta	1	1	1	0
Deuterostomia	*S. purpuratus*	1	1	1	0
	S. kowalevskii	1	0	2	0
	P. marinus	10	5	1	0
	D. rerio	26	19	15	0

Table S4: Mapping of Eph-LBD-ephrin-RBD interaction interface residues

Structural complex	Species	PDB-IDs	Residual contacts at Eph-LBD/ephrin-RBD binding interface		Reference
			Eph's Ligand Binding Domain (LBD)	Ephrin's Receptor Binding Domain (RBD)	
**EphA2-ephrinA1**	Human	3HEI 3MBW 3CZU	E40, D53, L54, M55, Q56, N57, I58, M59, P63, Y65, M66, S68, C70, M73, T101, R103, F108, P109, T151, F156, R159, H160, V161, C188, A190	Y46, D86, V88, R89, W90, R94, H99, E102, K103, L104, S105, K107, Q109, R110, F111, T112, P113, F114, T115, L116, G117, E119	[1, 2]
**EphA2-ephrinA5**	Human	3MX0 2X11	E40, D53, L54, M55, Q56, N57, I58, M59, P63, Y65, M66, S68, C70, M73, T101, R103, F108, P109, T151, F156, R159, H160, V161, C188, A190	Y57, K98, R99, K117, Q119, L120, F121, T122, P123, F124, S125, L126, E129	[2, 3]
**EphA3-ephrinA5**	Human	4L0P	I38, L42, H50, E53, E54, I55, S56, G57, V58, Y62, P64, Q69, T102, R104, P110, I111, F152, L157, R160, I161, L162, K163, C189, V190, A191, V193	A27, V28, A29, Y57, F97, K98, R99, W100, R104, P108, N109, F114, S115, K117, Q119, L120, F121, T122, P123, F124, S125, L126, G127, E129	[4]
**EphA4-ephrinA2**	Human	2WO3	E55, S58, I59, M60, N64, P66, R68, Q71, V72, T104, R106, D107, L111, P112, F154, I159, D161, R162, I163, L166, C191, I192, A193, V195	Y68, F109, K110, R111, W112, E113, K125, F126, F133, T134, P135, F136, S137, L138, F140, E141	[5]
**EphA4-ephrinA5**	Human	4BKA 4BK5 4M4R	G41, E51, E55, S58, I59, M60, P66, I67, R68, Q71, V72, C73, T104, R106, P112, F154, I159, R162, I163, M164, C191, I192, A193, V195	A29, R31, Y57, F97, K98, R99, W100, P108, N109, K113, F114, S115, K117, Q119, F121, T122, P123, F124, S125, L126, F128, E129	[6, 7]
**EphA4-ephrinB2**	Human	2WO2 3GXU	S36, Q40, G41, E42, E55, E56, V57, S58, I59, M60, E62, N64, P66, R68, T69, Q71, C73, T104, R106, P112, F154, V157, D158, G160, D161, R162, M164, L166, I192, A193, V195	K57, E94, T96, P97, L98, K103, Q106, D107, I108, K09 F110, T111, K113, Q115, S118, P119, N120, L121, W122, G123, E141	[5, 8]
**EphA4-ephrinB3**	Human	4BKF	S36, Q40, G41, E42, E55, E56, V57, S58, I59, M60, E62, N64, P66, R68, T69, Q71, C73, T104, R106, P112, F154, V157, D158, G160, D161, R162, M164, L166, I192, A193, V195	R57, A97, N99, L100, L101, R106, L109, D110, L111, R112, F113, T114, I115, K116, Q118, S121, P122, N123, W124, G125, E128	[6]
**EphB2-ephrinB2**	Mouse	1KGY	T35, T36, T38, A39, E40, E52, E53, V54, S55, G56, Y57, D58, E59, M61, T63, R65, Q68, C70, S101, R103, S107, P109, F155, D159,	K60, D62, T99, P100, L101, R106, Q109, D110, V111, K112, F113, T114, K116, Q118, F120, S121, P112,	[9]

			L160, G161, G162, V164, K166, I167, C192, S194, I196	N123, L124, W125, G126, E128	
**EphB4-ephrinB2**	Human	2HLE	T27, A28, D29, E43, E44, L45, S46, G47, L48, E50, Q52, S54, R56, E59, C61, T93, L95, L100, P101, T147, K149, P151, G152, A155, G157, V159, C184, A186, L188	K57, D59, P97, L98, Q106, D107, I108, K109, F110, T111, K113, Q115, E116, F117, S118, P119, N120, L121, W122, G123, E125, Q127	[10]

Note: The residue numbers correspond to UniprotKB sequence from corresponding species.

Table S5: Predicted TM regions and lipid anchoring sites in ephrins from non-bilaterian metazoans and choanoflagellates

Accession	Species	TOPCONS (TM envelope regions)	Pred-GPI	GPI-SOM
**Cnidaria**				
Aele_124411	*A. elegans*	274-294		
Aele_124437	*A. elegans*	274-294		
Aele_151933	*A. elegans*	150-170		
Aele_151935	*A. elegans*	151-171		
Aele_151940	*A. elegans*	114-134		
Aele_151942	*A. elegans*	115-135		
Aele_79976	*A. elegans*	259-279		
Apal_288406	*A. pallida*	370-390		
Apal_288439	*A. pallida*	295-315		
Apal_344094	*A. pallida*	299-319		
Apal_344111	*A. pallida*	229-249		
Apal_398190	*A. pallida*	312-332		
Apal_400465	*A. pallida*	231-251		
Apal_54304	*A. pallida*	236-256		
Apal_89985	*A. pallida*	242-262		
Atet_141600	*A. tetragona*	431-451		
Atet_40821	*A. tetragona*	115-135		
Atet_41310	*A. tetragona*	289-309		
Atet_88599	*A. tetragona*	146-166		
Btue_9750	*B. tuediae*	298-318		
Clat_120953	*C. lathetica*	279-299		
Clat_28862	*C. lathetica*	256-276		
Clat_80728	*C. lathetica*	419-439		
Clat_97078	*C. lathetica*	224-244		
Clat_97081	*C. lathetica*	261-281		
Ever_13730	*E. verrucosa*	283-303		
Ever_1897	*E. verrucosa*	237-257		
Hdig_4952	*H. digitata*	273-293		
Holi_5987	*H. oligactis*	265-285		
Holi_7726	*H. oligactis*	218-238		
Hvir_3164	*H. viridissima*	301-321		
Hvul_1018918.1	*H. vulgaris*	303-323		
Hvul_1019657.1	*H. vulgaris*	220-240		
Hvul_1028415.1	*H. vulgaris*	360-380		
Hvul_1028689.1	*H. vulgaris*	348-368		
Hvul_1029169.1	*H. vulgaris*	215-235		
Hvul_1033087.1	*H. vulgaris*	271-291		
Hvul_1034474.1	*H. vulgaris*	111-131		
Hvul_1038805.1	*H. vulgaris*	216-236		
Nbij_52832	*N. bijuga*	229-249		
Nbij_52843	*N. bijuga*	271-291		
Nbij_57193	*N. bijuga*	181-201		
Nvec_EDO36327	*N. vectensis*	346-366		

Nvec_EDO44950	*N. vectensis*	521-541		
Nvec_EDO47804	*N. vectensis*	246-266		
Pcar_179493	*P. carnosa*	268-288		
Pcar_189454	*P. carnosa*	249-269		
Pcar_215100	*P. carnosa*	226-246		
Pcar_235347	*P. carnosa*	282-302		
Pcar_240721	*P. carnosa*	284-304		
Pper_103076	*P. carnosa*	558-578		
Pper_105129	*P. periphylla*	313-333		
Pper_41140	*P. periphylla*	336-356		
Pphy_13023	*P. physalis*	336-356		
Pphy_19559	*P. physalis*	277-297		
**Ctenophora**				
Mlei_03441	*M. leidyi*		140 (ASP)*	
**Porifera**				
Aque_39602	*A. queenslandica*	248-268	245 (SER)	245 (SER)
Cpro_58774.15	*C. prolifera*	209-229	205 (SER)	205 (SER)
Cvar_1084	*C. varians*	254-274	249 (GLY)	
Cvar_12230	*C. varians*	279-299	275 (TYR)	
Cvar_2778	*C. varians*	230-250	227 (SER)	227 (SER)
Cvar_8977	*C. varians*	219-239	211 (ASP)	210 (SER)
Ifas_6298	*I. fasciculata*		230 (SER)	230 (SER)
Ifas_6301	*I. fasciculata*		233 (SER)	233 (SER)
Kvar_23578	*K. variolosa*	192-212	185 (ASN)	185 (ASN)
Lapi_139996	*L. apicalis*	250-270		
Snux_75316	*S. nux*	234-254		
**Choanoflagellata**				
Aspe_393792	*A.spectabilis*	381-401		
Surc_ 134676	*S.urceolata*	395-415		
Sdip_338217	*S.diplocostata (aus)*	477-497		
Sinf_130281	*S.infusionum*	367-387		

Note: The blank cell indicates either TM or GPI anchoring site is not predicted by the prediction methods utilised. * indicates not a strong probability for the predicted GPI anchor site, while the rest shown are highly probable.

Supplementary note:

Genome/transcriptome datasets used for sequence search

Complete proteome datasets were downloaded from various public database resources including Ensembl (v85), Ensembl Metazoa (v31) (Ensembl Protists (31), Ensembl Fungi (31), Broad institute, JGI, and Compagen among others. Majority of publicly available transcriptomes of species for which genomes are not yet sequenced were downloaded from NCBI SRA database (see accession numbers in Table S1). Transcriptomes of eight other sponges sequenced in an earlier study [11] were download from publicly available resources such as Evolution & Research Archive (*Aphrocallistes vastus*) and Dataverse network (*Chondrilla nucula, Ircinia fasciculata, Petrosia ficiformis, Spongilla lacustris, and Pseudospongosorites suberitoides Sycon coactum and Corticium candelabrum*).
Translated gene models for genomes *Oscarella carmela* and *Sycon ciliatum* were downloaded from Compagen database. Previously assembled transcriptomes were directly subjected to ORF predictions, whereas the raw unassembled transcriptomes were assembled by current or former members of Degnan Lab (Kevin Kocot, S. Fernandez-Valverde, Laura Grice and Federico Gaiti) and datasets are stored in Degnan Lab server for further use. In-house datasets include transcriptomes of two demosponges, *Niphatidae indet* (F. Gaiti, K. Kocot, B. Degnan, unpublished data) *Clathria prolifera* (S. Fernandez-Valverde and B. Degnan, unpublished data). The sponge referred as Niphatidae indet has tentatively been identified as belonging to the demosponge family Niphatidae based on spicule morphology, and its transcriptome has been sequenced and assembled by Degnan Lab (F. Gaiti, K. Kocot, B. Degnan, unpublished data) as described in [12]. Similarly, another transcriptome *Clathria prolifera* was assembled by Degnan Lab (S. Fernandez-Valverde and B. Degnan, unpublished data) and RNA for this *Clathria prolifera* was kindly provided by Xavier Fernàndez-Busquets (Institute for Bioengineering of Catalonia (IBEC), Barcelona, Spain). Best open reading frame (ORF) predictions were generated using either TransDecoder or using getorf program from EMBOSS 6.5.7 software package, where longest ORF between stop codons for each assembled transcript were predicted. After ORF predictions, the transcriptome sequences were further utilized for HMM searches as described in the methods section of main text.

Eph_LBD and Ephrin hits in choanoflagellates

Using sensitive HHpred and HMM profile searches we were able to identify Eph_LBD and ephrins for the first time in choanoflagellates. While these are not readily detectable in Pfam and CDD searches, we used HHpred searches plus manually curated HMM profile searches to identify and verify these domains. The hits are described here. The N-terminal domains of the proto Eph-like receptors were searched against a database of HMM profiles constructed from individual Protein Databank (PDB) entries with the HHpred program. For four of those, HHpred searches yielded significant relationships with ephrin-binding (EPH_LBD) ectodomain of the EPH receptors. 1) m.8217_Salpingoeca urceolata sequence recovered PDB ID: 5JR2-chanin B corresponding to Eph LBD; probability: 100 %; e-value: 1.7e-40. 2) m.240923_Hartaetosiga gracilis: probability: 96.32%; E-value: 2.5e-3; PDB ID: 3MX0_C. 3) m.108007_Microstomoeca roanoka: probability: 96.7%; E-value: 8.2e-4; PDB ID: 5JR2_B. 4) EGD75115_ Salpingoeca rosetta: probability: 97.8%; E-value: 2e-6; PDB ID: 5JR2_B. Reciprocally, a HMM search using manually curated Eph_LBD HMM profile (built from a selection of metazoan EPH_LBD from our survey) against all surveyed choanoflagellate datasets recovered these four choanoflagellate sequecnes with E-values ranging from $5.1e^{-13}$ to $4.5e^{-5}$ (m.8217_Salpingoeca_urceolata $5.1e^{-13}$; m.108007_Microstomoeca_roanoka $8.8e^{-8}$; m.240923_Hartaetosiga_gracilis $5.4e^{-6}$; EGD75115_ Salpingoeca rosetta 4.5e-05). Similarly, our searches identified four sequences across four different choanoflagellate species (transcriptomes of *Acanthoeca spectabilis, Salpingoeca urceolata, Stephanoeca diplocostata, Salpingoeca infusionum*). HHpred predictions for these are: 1) m.393972_Acanthoeca_spectabilis: probability: 93.79%; E-value: 1.2e−1; PDB ID: 4UF7_C. 2) m.134676_Salpingoeca_urceolata: probability: 97.84 %; E-value: 9.3e−7; PDB ID: 4UF7_C. 3) m.338217_Stephanoeca_diplocostata_aus: probability: 95.6 %; E-value: 6.4e−3; PDB ID: 4UF7_C. 4) m.130281_ Salpingoeca infusionum: probability: 97.33 %; E-value: 9.8e−6; PDB ID: 4UF7_C.

References

1. Himanen JP, Goldgur Y, Miao H, Myshkin E, Guo H, Buck M, Nguyen M, Rajashankar KR, Wang B, Nikolov DB: **Ligand recognition by A-class Eph receptors: crystal structures of the EphA2 ligand-binding domain and the EphA2/ephrin-A1 complex**. *EMBO reports* 2009, **10**(7):722-728.
2. Himanen JP, Yermekbayeva L, Janes PW, Walker JR, Xu K, Atapattu L, Rajashankar KR, Mensinga A, Lackmann M, Nikolov DB *et al*: **Architecture of Eph receptor clusters**. *Proceedings of the National Academy of Sciences of the United States of America* 2010, **107**(24):10860-10865.
3. Seiradake E, Harlos K, Sutton G, Aricescu AR, Jones EY: **An extracellular steric seeding mechanism for Eph-ephrin signaling platform assembly**. *Nature structural & molecular biology* 2010, **17**(4):398-402.
4. Forse GJ, Uson ML, Nasertorabi F, Kolatkar A, Lamberto I, Pasquale EB, Kuhn P: **Distinctive Structure of the EphA3/Ephrin-A5 Complex Reveals a Dual Mode of Eph Receptor Interaction for Ephrin-A5**. *PloS one* 2015, **10**(5):e0127081.
5. Bowden TA, Aricescu AR, Nettleship JE, Siebold C, Rahman-Huq N, Owens RJ, Stuart DI, Jones EY: **Structural plasticity of eph receptor A4 facilitates cross-class ephrin signaling**. *Structure* 2009, **17**(10):1386-1397.
6. Seiradake E, Schaupp A, del Toro Ruiz D, Kaufmann R, Mitakidis N, Harlos K, Aricescu AR, Klein R, Jones EY: **Structurally encoded intraclass differences in EphA clusters drive distinct cell responses**. *Nature structural & molecular biology* 2013, **20**(8):958-964.
7. Xu K, Tzvetkova-Robev D, Xu Y, Goldgur Y, Chan YP, Himanen JP, Nikolov DB: **Insights into Eph receptor tyrosine kinase activation from crystal structures of the EphA4 ectodomain and its complex with ephrin-A5**. *Proceedings of the National Academy of Sciences of the United States of America* 2013, **110**(36):14634-14639.
8. Qin H, Noberini R, Huan X, Shi J, Pasquale EB, Song J: **Structural characterization of the EphA4-Ephrin-B2 complex reveals new features enabling Eph-ephrin binding promiscuity**. *The Journal of biological chemistry* 2010, **285**(1):644-654.
9. Himanen JP, Rajashankar KR, Lackmann M, Cowan CA, Henkemeyer M, Nikolov DB: **Crystal structure of an Eph receptor-ephrin complex**. *Nature* 2001, **414**(6866):933-938.
10. Chrencik JE, Brooun A, Kraus ML, Recht MI, Kolatkar AR, Han GW, Seifert JM, Widmer H, Auer M, Kuhn P: **Structural and biophysical characterization of the EphB4*ephrinB2 protein-protein interaction and receptor specificity**. *The Journal of biological chemistry* 2006, **281**(38):28185-28192.
11. Riesgo A, Farrar N, Windsor PJ, Giribet G, Leys SP: **The analysis of eight transcriptomes from all poriferan classes reveals surprising genetic complexity in sponges**. *Molecular biology and evolution* 2014, **31**(5):1102-1120.
12. Whelan NV, Kocot KM, Moroz LL, Halanych KM: **Error, signal, and the placement of Ctenophora sister to all other animals**. *Proceedings of the National Academy of Sciences of the United States of America* 2015, **112**(18):5773-5778.
