# Supplementary material for: The first identification of complete Eph-ephrin signalling in ctenophores and sponges reveals a role for neofunctionalization in the emergence of signalling domains
Source: BMC Evol Biol. 2019 Apr 25;19:96. doi: 10.1186/s12862-019-1418-z (PMC6485061; doi:10.1186/s12862-019-1418-z)
Supplement: Supplementary file 2 — Complete FASTA sequences of all Eph receptors, ephrin ligands and cupredoxins identified new, or used for new analyses, in this study. (PDF 1079 kb) [file 12862_2019_1418_MOESM2_ESM.pdf]

Ephrins downloaded from databases/previous studies (Mellott DO and Burke RD, BMC CELL BIOL 2008)

>Hsap_EFNA1
MEFLWAPLLGLCCSLAAADRHTVFWNSSNPKFRNEDYTIHVQLNDYVDIICPHYEDHSVA
DAAMEQYILYLVEHEEYQLCQPQSKDQVRWQCNRPSAKHGPEKLSEKFQRFTPFTLGKEF
KEGHSYYYISKPIHQHEDRCLRLKVTVSGKITHSPQAHDNPQEKRLAADDPEVRVLHSIG
HSAAPRLFPLAWTVLLLPLLLLQTP
>Hsap_EFNA2
MAPAQRPLLPLLLLLLPLPPPPFARAEDAARANSDRYAVYWNRSNPRFHAGAGDDGGGYT
VEVSINDYLDIYCPHYGAPLPPAERMEHYVLYMVNGEGHASCDHRQRGFKRWECNRPAAP
GGPLKFSEKFQLFTPFSLGFEFRPGHEYYYISATPPNAVDRPCLRLKVYVRPTNETLYEA
PEPIFTSNNSCSSPGGCRLFLSTIPVLWTLLGS
>Hsap_EFNA3
MAAAPLLLLLLLVPVPLLPLLAQGPGGALGNRHAVYWNSSNQHLRREGYTVQVNVNDYLD
IYCPHYNSSGVGPGAGPGPGGGAEQYVLYMVSRNGYRTCNASQGFKRWECNRPHAPHSPI
KFSEKFQRYSAFSLGYEFHAGHEYYYISTPTHNLHWKCLRMKVFVCCASTSHSGEKPVPT
LPQFTMGPNVKINVLEDFEGENPQVPKLEKSISGTSPKREHLPLAVGIAFFLMTFLAS
>Hsap_EFNA4
MRLLPLLRTVLWAAFLGSPLRGGSSLRHVVYWNSSNPRLLRGDAVVELGLNDYLDIVCPH
YEGPGPPEGPETFALYMVDWPGYESCQAEGPRAYKRWVCSLPFGHVQFSEKIQRFTPFSL
GFEFLPGETYYYISVPTPESSGQCLRLQVSVCCKERKSESAHPVGSPGESGTSGWRGGDT
PSPLCLLLLLLLLILRLLRIL
>Hsap_EFNA5
MLHVEMLTLVFLVLWMCVFSQDPGSKAVADRYAVYWNSSNPRFQRGDYHIDVCINDYLDV
FCPHYEDSVPEDKTERYVLYMVNFDGYSACDHTSKGFKRWECNRPHSPNGPLKFSEKFQL
FTPFSLGFEFRPGREYFYISSAIPDNGRRSCLKLKVFVRPTNSCMKTIGVHDRVFDVNDK
VENSLEPADDTVHESAEPSRGENAAQTPRIPSRLLAILLFLLAMLLTL
>Hsap_EFNB1
MARPGQRWLGKWLVAMVVWALCRLATPLAKNLEPVSWSSLNPKFLSGKGLVIYPKIGDKL
DIICPRAEAGRPYEYYKLYLVRPEQAAACSTVLDPNVLVTCNRPEQEIRFTIKFQEFSPN
YMGLEFKKHHDYYITSTSNGSLEGLENREGGVCRTRTMKIIMKVGQDPNAVTPEQLTTSR
PSKEADNTVKMATQAPGSRGSLGDSDGKHETVNQEEKSGPGASGGSSGDPDGFFNSKVAL
FAAVGAGCVIFLLIIIFLTVLLLKLRKRHRKHTQQRAAALSLSTLASPKGGSGTAGTEPS
DIIIPLRTTENNYCPHYEKVSGDYGHPVYIVQEMPPQSPANIYYKV
>Hsap_EFNB2
MAVRRDSVWKYCWGVLMVLCRTAISKSIVLEPIYWNSSNSKFLPGQGLVLYPQIGDKLDI
ICPKVDSKTVGQYEYYKVYMVDKDQADRCTIKKENTPLLNCAKPDQDIKFTIKFQEFSPN
LWGLEFQKNKDYYIISTSNGSLEGLDNQEGGVCQTRAMKILMKVGQDASSAGSTRNKDPT
RRPELEAGTNGRSSTTSPFVKPNPGSSTDGNSAGHSGNNILGSEVALFAGIASGCIIFIV
IIITLVVLLLKYRRRHRKHSPQHTTTLSLSTLATPKRSGNNNGSEPSDIIIPLRTADSVF
CPHYEKVSGDYGHPVYIVQEMPPQSPANIYYKV
>Hsap_EFNB3
MGPPHSGPGGVRVGALLLLGVLGLVSGLSLEPVYWNSANKRFQAEGGYVLYPQIGDRLDL
LCPRARPPGPHSSPNYEFYKLYLVGGAQGRRCEAPPAPNLLLTCDRPDLDLRFTIKFQEY
SPNLWGHEFRSHHDYYIIATSDGTREGLESLQGGVCLTRGMKVLLRVGQSPRGGAVPRKP
VSEMPMERDRGAAHSLEPGKENLPGDPTSNATSRGAEGPLPPPSMPAVAGAAGGLALLLL
GVAGAGGAMCWRRRRAKPSESRHPGPGSFGRGGSLGLGGGGGMGPREAEPGELGIALRGG
GAADPPFCPHYEKVSGDYGHPVYIVQDGPPQSPPNIYYKV
>Cint_EfnAa
MLIHQCVFLLTCLSLVKLVISRTRHVVFWDRSINSSLTTELRLQVQINEWMDILCPKPAR
NDEKRGEELYIDVFNVTAAQYHRCNTQKIQRVLRCSDPKREIKLTTKFQERSPSPLGFVF

RPGQSYYYISKPSKGGTRGCSTDTLRLVVEVESQHGRVEANKHTRFDPWLDQSEFGMKHN
KRHHHLKNAENELEKREPQEPVVLSQKNSAAPPDGGNSAVGIHSRRHDTRLLALITSSLL
MVLFTKWV
>Cint_EfnAb
MPLSKVNRMILSLFMCYLLFDGVRSKRFNSGTITHEVMWDPRENGGFAFGNEFSIEVHMR
DYMNIHCPQYDQDDKAKLSFIIYNVSEQSYNSCSLTDMKTAIFKCDNPVKGRKLTTKFQR
RSPNPLGFVYQPNKDYFFMAFKKDEPQNCKSAMKMKVHVLPKRLHEHDRKPVTAGTTRSS
TPRTLSSTTTTTTIATTTTTTPRTSQKPHIPRQSSTRNTPPTKTLKPGYVRGDGEGDGPG
GGVGRITCATTWMLVALVLTVLLQN
>Cint_EfnAc
MIRSLAICFSLLLFQGEGNAKRRHHVIYWNTVMNPGLLSTSEHTYHVKIGEFLDILCPQN
SLMGITDGLEEPETFNLYNVTQTDFEQCSHGDNKNFIFACDRPERENKLTIKFQTISPSP
LGFRFQYCSEYYFVAASRDPNKRTNGCNKNSMRLRIFVECRETRHHKHDKKTTTASTTTT
TTLPTTTITKVRTTTSRKPRIVLPNVEHSNTTKSIPDKKPSEDPETLRQIKNRRGNNSSS
CLSFSINTWTLTFFVYVITTWVMS
>Cint_EfnAd
MATQFTYYLFTLCLICVNSQATVDRHVLYWNSVMTPGLKKNDYVMQVKMGQFMDILCPQR
SLMGITGPKEPTTFDLYNVTEDNYQQCNTGGHKNFIFACDRPERENKLTIKFQTISPSPL
GFRFQYCSEYYFVAVPRNGRRGKQSGCHKQSTRLRISVGCHETTTTSTTTTTTTTTTTTT
TTTTPKPTTSTANPDYEFIKKLLASTKDTQSSRDETFVPKLKSLLIKSSEKNSPVVLASS
LDQTSDNKGNAAPSHTSVFSTTLLVMLTSWVLRSLLLV
>Cint_EfnB
MTTVKMDKLTIFVAALSIVIWGPESVTVEGKTREPIYWNSANPLWDSETRLINVAINDRL
DIICPRRGRDEGEELFYYKLYLVSKEDFKRCNASNNDRRRLITCNVPDREKKYTFYFQEI
SPSPWGIEFQPDETYYVISTSDGTKSGIESLQGGVCTRMNMKLELRVHPEDTPIVDNRVM
VDTTEHNLPKPHLPPKVTDRKPIIDPNPPTHYNPVVVPPGTPKNTPPQSINPVDPPVDPV
APTQSENSTSVGSNGLVIGVVLGACAVLFIVLLGFLGYKVYRRRRHMKKYQSPPMTPTRG
ATPLHQVTLLPISTPHGHHARIQSEHERHRTGTMSSHSERPPPSYNESFLDNGGPVVAV
>Bflo_Efn1
MALNKWTWFSRLKSYPAAHVFTFLFLTMLDRTEGYFFGRVLPDINWNQNDDIFQREGGYV
LEVDIGDKLDIICPRLFNQYNVMYEVSREGYETCNSTGGRLLLRCNLPETDLVLTVLFQE
VSPSPFGLEFKKGKDYYYIATSGGTLDTLDNRVGGNCESPTYMKLTIRVRGDPTERSEST
DGNYAGRGDGVTTLPSVAKSADRRVNSASQLRRGALDFVTLLLTALLATVFGTLRL
>Bflo_Efn2
MALQTLLPELAVQSLTSCTFLGTRLENHRKSLWPGKVFLRRLGQDRLKTVSAKTSQPQCK
ERFGVSRPDFLLCVLRPPHLMWSQDAQQGFSGGGYHLSPNVNDMVTVFCPHYEVGSTDLV
EYNVLYEVDEDGYNRCDAERGRPLLRCDRPYRSYPLSLTIYFQEFSPSPYGLEFDRGRDY
YYISTSTGEYGGLNATVGGNCVNGMKMKLSICCKSNTSDTSVANTLHLGVSWFCGLSVAA
LFFNMASLSPPSLSATETPNVVEEQTQRRSPPHIPSNRIPVAGFLAFRAAFKGLSRFPCH
RGKGSCDEGICHPELLQVLVIEPYPVKDRFRSSSYREDGTDGSEHCPYGGEKTLSLTGPD
LSSVMSALSYLAIQYHAAGPPHCLQHMQSTGSG

Ephrins identified in this study from our searches

Ephrins in bilateria

>Drer_10432.8 D. rerio
MGDSLWRYYFGVLVIACKVNLSRALILDSIYWNTTNTKFVPGQGLVLYPQIGDKMDIVCP
RVEGGSMEGVEYYKLYMVPLEQLKSCQVTKADTPLLNCVKPDQDVKFTLKFQEFSPNLWG
LEFFRGKDYYIISTSNGTMEGLDNQEGGVCKTKSMKIIMKVGQNPSDPISPKDYPTSYPP
KHPDLGGKDSKSNEVLKPDASPHGEDKGDGNKSSSVIGSEVALFACIASASVIVIIIIIM
LVFLLLKYRRRHRKHSPQHATTLSLSTLATPKRGGSGGNNNGSEPSDIIIPLRTADSVFC
PHYEKVSGDYGHPVYIVQEMPPQSPANIYYKV

>Drer_12577.6 D. rerio
MDFLWLLCVAVSVSAWYASAERHSVYWNSTNANFLWDDYTVDVRINDYLDIICPHYAHGE
IASQEAERYVLYMVELEDYENCKPHSFDQLRWECSRPFAPHAPEKFSEKFQRFTPFTLGK
EFRQGESYYYISKPLHHHGQECLRLKVDVVGPHGSKNKKKMVEKVEEIEGKMAAGGVHNP
SNRLPADDPIAMIPVVQRSVGSSGVSVAPISSFVTLLSVFICLVLHQAS
>Drer_24428.4 D. rerio
MAGSDGSWKYYLWILTAMCRYALPAAKSLESVVWNSQNPKFVSGKGLVIYPEIGDKLDII
CPKGDMGRPYEFYKLYLVKKEQAESCSTILDPNVLVTCNKPEKDIKFTIKFQEFSPNYMG
LEFKRFTNYYITSTSNGTQEGLENREGGVCSTRSMKIIMKVGQDPNAPDPDLPDLPDRPY
DNEIKDPTTSPSRKTERGRENEVDGNGSKMPGKDTRNQNNSPGSVEGIFGSKPALFAAIG
AGCVIFLLIIIILIVLLLKLRKRTRKHSQPRGGTALSLSTLATPKGAAQAGSEPSDIIIP
LRTTENNYCPHYEKVSGDYGHPVYIVQEMPPQSPANIYYKV
>Drer_40277.7 D. rerio
MELSLVVFTVVCWVSVWSDDRIISDRHAVYWNSSNSRFWQGEYTVAVSINDYLDVYCPYY
ESPQPHSRMERYILFMVNHDGYLTCEHRMRGFKRWECNRPQSPDGPLRFSEKFQLFTPFS
LGFEFRPGHEYYYISSPHPNHAGKPCLKLKVYVKPTSSGYESPEPFLTDQSQRCGADGPC
LAVLMLLLVFLLAGV
>Drer_50216.6 D. rerio
MDLVWLLYIAASICAVFASAERLTVFWNSTNSKFRWDDYAVEVRLNDYLDIVCPHYPLGE
VPSQDAERYVLYMVEREDYDTCRPQSYDQMRWECGHPFAPHAPEKFSEKFQRFTPFTLGK
EFRQGESYYYISKPLHHHGQECLRLRVDVIANDGSQEARVGQGTKVGTGGGAHTPSNRLP
ADDPVVALPEVQKSTRTNSAISLASWTVLTTLLPLLLLLLLQ
>Drer_62002.6 D. rerio
MGVNRRNSGFQMASRGCVNGLGILLIFLVDLLGITATNMEPIYWNSLNKRFSDDKGYVLY
PQIGDRLDLICPSSDPPGPRAPADYEYYKLYLVSSREQADRCEVTGAPNLLLTCDKPNSD
MRFTIKFQEYSPNLWGHEFKTNHDYFIIATSDGTRQGLESMRGGVCATQGMKVVLKVGQS
PYGLPAKSPKPDSAGRINNPNPGTGNSTHPQIPPRGSGGENGPLPASNIAVIAGAAGGSA
FLLLVTAVICVVCYRRRHAKHSESHHPPLSLSSLTSPKRGCGGGVGGGNNNGSEPSDIII
PLRTSDSAYCPHYEKVSGDYGHPVYIVQEMPPQSPANIYYKV
>Drer_73969.3 D. rerio
MGIQTRGAWVAMAFSDWVSALGFLLIFLMESLAVDATNMEPVYWNTLNRRFGDERGYILY
PQIGDRLDLVCPATSPAGPNSPPEYEFYKLYLVSTREQADRCEVMGAPNLLLTCDKPNSD
MRFTIKFQEYSPNLWGHEFKNLRDYYIIATSDGTKEGIDSMRGGVCVTRGMKLVLKVGQT
AYGLPPKPKPDPGRPNSKIPDTESGSKGGETSESGAVSASNIALISGGAGGAFILLIAIV
IAVVCYRRRKAKHSETHHPALTLSSLTPKRGSSGGTATGSSGGNNNGSEPSDIIIPLRTS
DSAYCPHYEKVSGDYGHPVYIVQEMPPQSPANIYYKV
>Drer_79638.5 D. rerio
MDATVWSYIFGLFVFACRVKLTRSTVLESIYWNTSNTKFVPGRGVVLYPQIGDKMDIVCP
RIKPGSTEQTNIEYFRVYLVPKEQLETCHVTKSDMLLLNCDKPDQDVKFTFKFQEFSPNL
WGLEFLRGKDYHIISTSNSTFEGLDNHHGGVCRSKSMKLVLRVGQSPTDSFSAKNHPTRN
PPKYPENKDQNTFSKENDVSQIDSMQNGESGGKSGESVGSAGSDVALFAGVASGAVIFIL
IIIALVALLHRRHQKHSAQCSGQLPLNTLPKRGSGASGGSNNNGSEPSDIIFPIRTSGSM
YCPHYEKVSGDYGHPVYIVQEMPPQNPANIYYKV
>Drer_101723.3 D. rerio
XFLNGEYTVEVAINDYLDIYCPHYEEHLPQERMERYILFMVNYDGYTTCNHHMKGFKRWE
CNRPISPNGPLKFSEKFQLFTPFSLGFEFRPGHEYYYISSPHPNLGGKPCLKLKVYVKPT
NDSLYESPEPFLTDDSSGGWILMPSLWLMLPLLLHLCSS
>Drer_111146.1 D. rerio
MPQVEMFYLLFSSLWMSVLGLDPASKVVADRYAVFWNRTNPRFYRGDYHIDVCINDYLDV
YCPHYMDTVPEERTERYVLYMVNYDGYSSCDHTAKGFKRWECNRPHSPNGPLKFSEKFQL
FTPFSLGFEFRPGREYYYISSTLAENGRRSCLRLKVFVRPTNNCDKSHDRVDKFDDRSIN
SIENNNDGTSHESAEPSRSDVNSAGRVQTVGPLLASVLVLLASLSSL
>Drer_116412.1 D. rerio

XFYLVPGFRLCFWSQVTICGQLPSKPITPEKPSIQRGKKHTKVWNHLRKSKWFYRGDYHI
DVCINDYLDVYCPHYMDTVPEERTERYVLYMVNYDGYSSCDHTAKGFKRWECNRPHSPNG
PLKFSEKFQLFTPFSLGFEFRPGREYYYISSTLAENGRRSCLRLKVFVRPTNNCDKSHDR
VDKFDDRSINSIENNNDGTSHESAEPSRSDVNSAGRVQTVGPLLASVLVLLASLSSL
>Drer_119988.2 D. rerio
MALAAVSLYLLALAWTNLRPVHGNNRHAVYWNSSNIHLRREGYTVQVSVNDYLDIYCPHY
NQSQRGSLERGVAEQYVLYMVSYRGYRTCDPQMGFKRWECNRPHAPHAPIKFSEKFQRYS
AFSLGYEFNVGHEYYYISTPIHHHGHSCLRLRVYVCCSTDDDSSPPDYTVRPKLHDIDEF
NPEIPKLEKSVSGSSPSGDRLLITVATLLLAALLVS
>Drer_133669.1 D. rerio
MALYALCLFLLTLTCTNFALVTAARHAVHWNSSNILLRKEGYTLQVNVNDYLDIYCPHYN
SSQRGIAEQYVLYMVSYRGYRTCDPQLGFKRWECNRPHAPHAPIKFSEKFQRYSAFSLGY
EFHVGQEYYYISTPTHHHGRSCLRLRVYVCCSTASDSDDEPQPTEPDYTLRPNIKIDDLD
DYDNPEVPKLEKSISGSSPSRDRLLLTVASLLLIALSVS
>Drer_136933.1 D. rerio
WSVLFCRFQRGDYHIDVCINDYLDVYCPHYEDSVPEERTERYVLYMVNYDGYSTCDHTAK
GFKRWECNRPHSPNGPLKFSEKFQLFTPFSLGFEFRPGREYYYISSMITETGRRSCLKLK
VFVRPPNGCEKTIGVHDRVFVDDKVDNALEPRDDTSHEAEPSRSDVSTSGLRHQTSRPLL
ALLLLCISLYLLL
>Drer_141804.1 D. rerio
MNTPRCSGVCAMMRLLALFIHVLSTTAKRHVVYWNSTNTRLTGDDYSVQVNLSDYLDILC
PHYPGDHPSGASVETLALYLVAESQFRGCVDTKEAIKRWECNQPFAPFGPVRFSEKIQRF
TPFSLGFEFLPGHHYYYSSLSTDDGPPLPCMKLRVTVCCPPTTAGSSRQEATETPSHSTT
VQNMTLTQLFLLLIPFLLSYFL
>Pmar_8071.1 P. marinus
FPGSHGVILYPQMGDRIDLICPVGLGPGPAEFHRLYLVSRDSSVRCEAPPGARNSRLVLA
CDRPARALKYTLKVQRVSPNYRGLEFAPKREYHIILATSDGTWEGLSGRVGGACVTHGMR
LVLRVG
>Spur_23757 S. purpuratus
MALPKAKSGIIFFVNAVLLLWSVTLAKTLQPIAWDKNNSNFTSTGLNISVEINDLIDIQC
PQTENTTEGRLKAQYLKFMQVSYQGYEQCSLSARNRHLLSCNRPYVQNRLTLLFQEHTPY
PRGPTFRHGEEYYFITTSNGSANGIDNSRGGLCESHYMKLRVYVKPKPTASPTTGGPPTT
PSNSSTPPDISRAATSMTSRTTTRATTTLRTTSLPPTTTRRTTRYTTGGKIKSTEEGNNI
PDNMTRGAANRRHVSTTLLLASLLVAVVRWLHP
>Skow_16956 S. kowalevskii
MYTAVMALQWWLAFLIMIEPCVSAAAAEQLEPIVWSVGNPIFRRGLRYIVQVRIEDTVDI
ICPEKPKDNLGDSPSEYYRIYLVSKSDYEKCDTTTSRLLLNCDQPSVETKFTFLFTQLSP
VPNDFIFQPCQDYHLISTSNATEAGDEGMENKMGGVCRSNNMRIIIRVSCTVPTYSQILP
>Skow_5664 S. kowalevskii
MILSLIVILLAICGHLVCTKRLDPIVWDSNTFPNGGQALQVNMWDEVAIVCPREENTTEG
RERAQYNVIYMVPKLGYDECRINLPETRVLHRCDEPFQENRYALLFQDYSPVPGDFVFEH
GREYYYISVSNDRIDGLNKVSGGNCQYKHTKLIIKLCCGNAPTRVPAVTFSSSTKESNVI
PDKTTLFEDGSEDYESKEHNSVHDNKDDPKNDLLSSSSSSMSNLIRTSCFINLLSFSIVR
LFV*
>Ctel_225002 C. teleta
MRFINHSLRFKASNNDHVIEVNLYDDIDFVCPHFEDDDSGNYEEYYLIHQVSKREYDDCN
LYSPDTAIMIVNCSSPGKKKRFTILFEPFQSIPNVPEYHPGGRYYFISTSTGGREGIANP
HQGACVYKNMKLTIQVCCESTTPPPPANSTLAIPSTDSTPHPSVVPGNANTSSDLSTTTP
KITSTPPPSPPSTTLKDPKDPQGSSNPKFKTTVTPYRSKPPDRTKDPDDEKRENEIDDPE
HHKSINRASDSRVPQTLLCASLTFSAVLRYLLGR
>Lgig_171062 L. gigantea
MDIITQIFATSNTDHVINVNMGDIIDVICPQFPEGSDPRNYEYYTIYMVNKTQYDECTIY
SPRSVMFLVNCTDPTKNPADFYTFIINNFQSIPGNPDFKRGKSYYIISTSGGGLKSIKNQ

FQGACYHQHMKIRLNVCCEANDTGTENQNTGSNNQGSNAQDGRTGSPSHGSGDSGTSKPA
TNNKTPTTVTPSTTTRHTTTNGKTPSTDEPKTDPNKTPPEKNNIVDNKIIHANGPKNVGL
ISDNGNGSGVANSFSVLLVTFTVLLSVSCCS
>Tpol_19734 T. polymorphus
MDTVPWWCANLLVMFLSSFVGGLDLPPIYWNSTNEMFKSDNIDHEIHVTLGDKIDIICPY
YKEAGYETSDMEYYVIYQVTKRGYDDCSAFSDTSNKHVMRKVVTCDGKTTRMTILIKDFS
PIPNSLEFHQGKTYYYISTSTGTKEGLEKKYQGACYDKNMRMKFKVCCDDKLPSGPVSET
TGKSTQPTYTTSKWQRKTTKKFIPPKYDKDKQKDIINKDKNQNEVLPNNPSDLKRVSQGA
IAVTFTPIFTLFCSLLVTITKFILINR
>Tpol_19735 T. polymorphus
MDTVPWWCANLLVMFLSSFVGGLDLPPIYWNSTNEMFKSDNIDHEIHVTLGDKIDIICPY
YKEAGYETSDMEYYVIYQVPKEQYYDCSINSPSKIQIIFCKKPLRRRRYTIIFSQYSAIP
NAPEFRKGQSYYYISTSTGTKEGLEKKYQGACYDKNMRMKFKVCCDDKLPSGPVSETTGK
STQPTYTTSKWQRKTTKKFIPPKYDKDKQKDIINKDKNQNEVLPNNPSDLKRVSQGAIAV
TFTPIFTLFCSLLVTITKFILINR
>Hpsi_83841 H. psittacea
MKTSDIINDYTMPWFVTNLNIYVVLIISFCVQSFQQQPAPSTPLPVTYHVYWNSSNQIFN
LNNNHHIINVRVQDAVNIYCPYYAASPNGPEPTVTTSGLEYYVIYQVAQHQYRSCDLNPT
DAGNNNIKRLIVNCSDPSGNRNSIATIFIEAFSSIPGKDSFEAGNSYYFITTSSGEANGL
SQDHHGACKKNNMKMRIDVCCPPDSRTAECENHEVQFTCSTSQTTPSQPATDRPLDRTTP
RPTVTTHRTLATSGRTPTLISDKPTIKTETDPTVVGTGEPHTSSTDKTHIHKLTVYFVSM
VTLLFLIRQKQ*
>Hpsi_83847 H. psittacea
MKTSDIINDYTMPWFVTNLNIYVVLIISFCVQSFQQQPAPSTPLPVTYHVYWNSSNQIFN
LNNNHHIINVRVQDAVNIYCPYYAASPNGPEPTVTTSGLEYYVIYQVSKNEYDSCNISGS
KQIANCSTPRSSTIPTTLLIQSFSPTPGATQYQKGNSYYYITTSSGEANGLSQDHHGACK
KNNMKMRIDVCCPPDSRTAECENHEVQFTCSTSQTTPSQPATDRPLDRTTPRPTVTTHRT
LATSGRTPTLISDKPTIKTETDPTVVGTGEPHTSSTDKTHIHKLTVYFVSMVTLLFLIRQ
KQ*
>Dpul_EFX72793 D. pulex
MNKEREKEEGLTTFKGATRQIPIFNVHSTSNLACVAYMVLSVFRIDNTDHIIDVNKNNIP
FEYDQVNIICPVYTPGTHEDDAEKYIIYNVSKEEYDTCRITNPNPRIIAVCDKPYKLMYF
TITFRSFTPQPGGLEFRPGQDYYFISTSSRDDLHRRIGGRCSSHNMKVVFKVCCKAEDLD
KAGTTAGRPALPSTTTSTTMSTISTSVAVMNSTTTQAPVVPTRIITTQVMLPSVTSPRMP
PHWKDVEIQSFSVRTTTKKSDDFDKLSNEVVKNEELTSKSSGARRQVVFSPLSTLIAAAL
VTLVLFRVR
>Lith_21086 Lithobius sp
DALPLDVSCVRLDDIYWNSSNPIFRIDNTDHIIDVNKNNIAFEYDQVNIICPVYPPNTDQ
EEAEKYIIYNVSKEEYDTCTITNPQPRIIAVCDKPHSLMYFTITFRSFTPQPGGLEFRPG
QDYYFISTSSQTDLHSRYGGRCLTHNMKIIFKVCCDPSKVTPTPRTTTTTSANAVGPMAP
PTPRRRTTTTTTTTSTRPPTTAMAKTTSIKGGNSGSSSSEIHPDKHNKKTESKDKHPNET
RVKNEELVNNTASTWPTRAHIHWVLFTSILTLMSASLLAR*

Additional bilaterian Ephrins that are included only in the supplementary tree and not elsewhere (Supp_tree_additional_Ephrins_complete_mining_tree_file): See additional file 3 for tree file.

>SPU_023757-tr
MALPKAKSGIIFFVNAVLLLWSVTLAKTLQPIAWDKNNSNFTSTGLNISVEINDLIDIQCPQTENTTEGRLKAQYLKFMQVSYQGYEQCSLSA
RNRHLLSCNRPYVQNRLTLLFQEHTPYPRGPTFRHGEEYYFITTSNGSANGIDNSRGGLCESHYMKLRVYVKPKPTASPTTGGPPTTPSNSST
PPDISRAATSMTSRTTTRATTTLRTTSLPPTTTRRTTRYTTGGKIKSTEEGNNIPDNMTRGAANRRHVSTTLLLASLLVAVVRWLHP
>LotgiP171062

MDIITQIFATSNTDHVINVNMGDIIDVICPQFPEGSDPRNYEYYTIYMVNKTQYDECTIYSPRSVMFLVNCTDPTKNPADFYTFIINNFQSIPG
NPDFKRGKSYYIISTSGGGLKSIKNQFQGACYHQHMKIRLNVCCEANDTGTENQNTGSNNQGSNAQDGRTGSPSHGSGDSGTSKPATNN
KTPTTVTPSTTTRHTTTNGKTPSTDEPKTDPNKTPPEKNNIVDNKIIHANGPKNVGLISDNGNGSGVANSFSVLLVTFTVLLSVSCCS
>EDO47804
VTLLVLMAANLSLVESMFYKSIVWSPQNPIFSGTAIKCVIPKSKMTIVCPTQALVVQDSPLIPPKEQLYENIWLVNRGSYDSCNISTADSEKRL
LLRCDTPLVLKWYTLIFQEQSATAQGLEFVPEREYYFIATSTGDKSSLDSTAGGRCSSSRMRLQIHICRDVDDIHCKSEAQCQALLNKEESTVA
PATTTQYHPTDDNFTEGYSRNNLTSTPAPCIGKPDSHGRRDFFTEMKYQFAVGFLTAVCLVFIVSTAYLGYRLYRKTKESPRMCSENGRFSM
RASTRTTSDGADSIDGAEHELLRDSSHC
>Bm17311
INLIILLLLDIISDYYHIVEARRLPDIYWNSTNQMFDISNTDHVMSVKLLDRVTIICPLPTTNPQISYEYTKLYAVSQNGYENCQLLNERLIGVCQ
TPRQQSSISIVFRDFSPLPGALEFKPGHSYYFITTSDGTKKGIDRRSGGLCATEHMKIKFEIQSDRDDNNLNDYFEPSSAHFRHRLQNSAEVST
PLLYIIHTSDSSSAEGYAISDDDNDDDEGGGAACNYQLNLSLTAITLIILYFITIA
>EJD76147.1
INLLITLVLDMFSDHRTADARRLPDIYWNSTNPMFDISNTDHVMSVKLLDRVTIVCPQPTINPQISYEYTKLYAVSQSGYENCQLLDERLIGV
CQTPKQQSSISIVFRDFSPLPGALEFKPGYSYYFITTSDGTEKGIDRRSGGLCASEHMKIKFEIQSDRGDNLNDYFEPSSAHFRHRLQNSAEVS
TPLLYIIHTSDSSAEGYAAAEDEGDAACNYQLNISLTVLALIFLYFTTIA
>CBG07443
MYVLLGVLAFTFPAYCRDYHDVIWNSRFFPQHLKHSPMNVRLGDQLTIICPKSFHRGMHYEYAKLYWVGKQDFDQCTHNTYYTKLMGVC
ANETETTAIKMIFQKYNPIPNGIDFQIGEIYYIVSTSSGHLEDISQPTGGLCWQENMKLAIRIVGEELPRMKYEVHDYQSLVSSSTSSPLSSTM
NSLVILFIASKF
>DPOGS203864-PA
TFLVLVFLIESVMGNYAKSFNIHWNTTNSIFRIDNTDHVFDLNKGNAQFEYDQVNVMCPVYAPGTFEEDTEKYIIYNVSKEEYDTCRITNPN
PRIIAICDKPHKLMYFTITFRPFTPQPGGLEFLPGKDYYFISTSSKDDLHRRIGGRCLSHNMKLVFRVCCKPEDQSPVPPPQPTQPTPPPPPTT
PTTTSTTTTIKPVTKKTHKYDKTPNEVVKSEELSYSRGAALASSIALALAASAVLAPLAR
>C43F9.8
MQIATFILLSLFPFIGWARKIPDINWISSNPIFDVSNTDHVISVHIGDRVSIRCPKSDETGKYEYSYIYMVSDEEYDHCFLSKPRLVGACDNQTI
NASINIVFRSFTPTPGGFEFQPGKNYFLISTSDGTLEGIDRKKDGLCTAKQMKIKFEVGQDRRGIENPKFAARTLKKDRDAEHSTPVMYVVH
DESDIDDDDDGSNACSYFVSIVVLLASRYLL
>CBG10748
MPPYFLLLLPLLFLIETHAKRLPQIYWNSTNPLVERYAAIGDTLDIVCPFFDENSDERTEQSIIYRVTEEEYENCEHRSNAKELGRCTQPYQEEK
LKVAFRLMSPNPSGLDYRPGVTYYFISTSTGSRKGLYNEQGGLCASHNLKMVIHITDRNGDIGIQHHRHHHHKKTTTTTTTTTTVPPKTVPA
TQEDMDSSAEKLWEQFYEKVMPIDNKWPEVTRGERVTLYQGDKKDEYEPAVPADGLDFEIHEIGDVESLYSSTSRLQYLLLLPAVLMLRLL
>CBN29472
MRLSISLLLLSIFSIHPGSTRKIPDINWISSNPIFDVSNTDHVISVHIGDRVSIKCPKSDETGKYEYSYIYMVSDEEYDHCFLAKPRLVGACDNQTI
NASINIVFRSFTPTPGGFEFQPGKNYFLISTSDGTQEGIDRKKDGLCSTKQMKIKFEVEPDRRGSENPKFAARTLKKDRDASEQSTPVMYVV
HDGNDIDDEKIKVKAFVGFPSPTSSIFDWVPFHPFGLKQSTPLIRLKVRAYNRVVYTKKDTLLKEVEDVKEYKKQQEKQFEQNHPTSLEEPEI
NVNEPDKMYEKGEEPKIFLFPKQGETVSKIGGEMGKVEELAHENEDSTTEKPKDDDFQIMYDDELKEYVLKRRPPKMDRNGVIDSWNGG
DSKERGDDGKREKVVDEEWREEEDEDDEARRILESEEDGIEGSTFKNRTVAGGTLKKKKNHTEVTLEEEEMNLDGLWCLL
>CBG04418
MRLPILPLLLTLFPIFGWTRKIPDINWISSNPIFDVSNTDHVISVHIGDRVSIKCPKSEPNGKYEYSYIYMVSDEEYDHCFLSKPRLVGACDNQT
VDASINIVFRSFTPTPGGFEFQPGKNYFLISTSDGTQEGIDRKKDGLCTTKQMKIKFEVEKDRRGIQNPKFAARTLKKDRDAEQSTPVMYVV
HDGNDIDDDDDGSNACSYLVSIVFLLASRYFL
>Y37E11AR.6a
MHPPIKIQTILLFILTTVHCSAKRLPQIYWNSTNPLVERYAAIGDTLDIVCPFFDENSDELTEQSIIYRVTEEEYENCERRSKAKELGRCTQPYQE
EKLKVAFRLMSPNPSGLDYRPGVTYYFISTSTGSRKGLYNEQGGLCASHNLKMVIHITDRNGDIGPHHHRHHHKKTTTTTTTSTSTSTPKTIP
PVVEMDSSAEKLWEQFYEKVMPIDNTWPEITRGERVTLYQGNKKDEYEQVPAEVVDFEIHEIGDVESLYSSSGRLRYVLLLPALLLLRIF
>CBN21225
MLPNISSLLLLLLFTISTVAKRLPQIYWNSTNPLVERYAAIGDTLDIICPYFDENSDELTEQSIIYRVTEEEYENCERRSNAKELGRCTQPYQEEKL
KVAFRLMSPNPSGLDYRPGVTYYFISTSTGSRKGLYNEQGGLCASHNLKMVIHITDRNGDIGIQHHRHHHHKKTTTTSTTTTTEKPSKLQEL
DASAEKLWEQFYEKVLPIDNNWPEVTRGERVTLYQGNKKDEYEQVPAEVVDFEIHEIGDVESLYASSPRLPILLLIPAVFLLRLL
>TC008794_001

QPSSVLVNGLILLTFSLHSFVTATNPSSAKFYTIHWNSSNPIFRIDNTDNVIDVNRYNLKFEYDQVNLICPVYVPGTRDEEMEKYIIYNVSKDEY
DTCRITNPNPRIIAVCDKPYKLMYFTITFRPFTPQPGGLEFLPGHDYYFISTSTSDDLHRRIGGRCTTHNMKIVFKVWGPAQTSPPPAPATVS
RWWPVTSPRSTSTSSTTTTTSTPPPVTTTKKSKTYGGKHPNEVGKSEELTLGGGAAVGPVQVLVVCAVAVLLAMR
>CRE10731
MPPQSLTLLNYILHLLLLSQIPISTAKRLPQIYWNSTNPLVERYAAIGDTLDIICPFFDKDSDDRTEQSIIYRVTEEEYENCQRKSSAKELGRCTQ
PFQEEKLKVAFRLMSPNPSGLDYRPGVTYYFISTSTGSRKGLYNDQGGLCASHNLKMVIHITDRNGDIGIQHHRHHHHKKTTTTSTTTTVAP
ETTDEETDSEKLWEQFYEKVMPIDNQWPEVTRGERVTLYQGNKKDEYEQVPAEVVDFEIHEIGDVESLYSSSGQQQYLLLLPAVLLLRLL
>ACYPI002895-PA
LTLVLLICFETVLLSTVSDCTKSFTVHWNTSNSIFRIDNTDHIIDVNKGNQPFEYDQVNIVCPVYAPGTHESDAEKYIIYNVSKEEYDMCRITNP
NPRIIAYCDKPYKLMYVTITFRSFTPQPGGLEFQPGQDYYFISTSSKDDIHRRIGGRCSTNNMKVVFKVCCRPEDQQNNQNLAPRPTQNAG
NPQRLPFPGSTHGYPARPPSVYASSTQQPSLAGKQPPFGATGPSVNNNKPSHKKSSEYDKHPNEVVKNEELTYNGAGAIAASMSAKVLAIL
VLVAIAAVDRLILYR
>EDO44950
NLRASQEHGYRGLCEIYPPLIWSPLNPAFRPVPFGTENDTVYSMNVLPMSKLYIICPNQVLNPTKVQLNTPKELLYENLWIVDKNSFDSCQV
NTLLRRNKQLMECTTPLALKYYPLVFQDFSATADVLEFERGKDYFFIATSNGQQSSLQSTRNGRCQTHNMKLQVHVCHSATDPVCNPTEP
PTTLPTTTQATTTPQPTTQSTPTTLTTTTAATTTTTPSTSTPTTQPTTLPKVVQAAPPETPSPRQPEPRTKEDVDVRDSITGPRAGASKGTRCP
RDPEDTTWLILVAVMGAIIAVSISCNIYILCCKKQSSSHQPRSDDKRSSAAHALLRENSRIDKV
>F15A2.5
MSSSWALFLLLFVAPLVSCRNYHDVMWNSKQFPVAFKTTPIKVDLGDQLTIICPKAYGMTYEYAKLYWVGETEWSQCWLHEPVWLGVCA
TENYTTEVKLIFRQTNPIPDGMDFQVGKTYYIISTSTGDIEGINQAVGGLCKYHHMKLAISVVGYEKQSHSKSEITEKNFAHGIGYEIHEVGQL
VSSGHQNFTLLTTTSLLFCTMFLSGVLF
>SRAE_X000008200
YNILLFIAFNILLTSDSLFVDGKKLTDLLWDPQNEIFNNNNNIKPVIQAHIMDRIKINCPKARENDRKYKYSKLYIVSKEGYEKCSLINSKIIGTCK
NPNLTSSINIVFREVSPLVGAFVFKPGEDYYLISTSTGTLDGIDNTEGGLCLTDNMKIKFEIQEKDQKLSNENSQTNIENKYIEKDESNSMTAGL
LQSFNDDTAIVYKIHNADVEYNDDELYGYINSTKNISLNYNIFMIYLFFFIISYIFFLI
>TC004127_001
LTVVLLVCMETVFLCTQSTCSKTFQVHWNTSDSIFHIDNTDHIIDVNKGNAQFEYDQVNIICPVYTPGTYGDDLEKYIIYNVSKEEYETCRITN
PNPRIIAICDKPYKLMYFTITFRPFTPQPGGLEFLPGKDYYFISTSSKDDLHRRIGGRCSSNNMKIVFKVCCAPDQSQANFTTKLTSTSTTTLG
WPDIHRPMVEATSSSTTQWAPRTDAPPTRKPTKKMNEYEKHPNEVMKNEELTYSKGVWLSAGPPSFLVVIACIAVIRWR
>CapteP225002
MRFINHSLRFKASNNDHVIEVNLYDDIDFVCPHFEDDDSGNYEEYYLIHQVSKREYDDCNLYSPDTAIMIVNCSSPGKKKRFTILFEPFQSIPN
VPEYHPGGRYYFISTSTGGREGIANPHQGACVYKNMKLTIQVCCESTTPPPPANSTLAIPSTDSTPHPSVVPGNANTSSDLSTTTPKITSTPPP
SPPSTTLKDPKDPQGSSNPKFKTTVTPYRSKPPDRTKDPDDEKRENEIDDPEHHKSINRASDSRVPQTLLCASLTFSAVLRYLLGR
>RPRC001731-PA
IFILEVKCFSLTRFCITHRFRIDNTDHIIDVNKGNIPFEYDQVNIICPTYTPGTHEEDAEKYIIYNVSKEEYDTCRITNPNPRIIAICDKPYKLMYFTI
TFRSFTPQPGGLEFQPGQDYYFISTSSKDDLHRRIGGRCTTNNMKVVFKVCCRPEDSHPNSSKSVSSEESTVVPGTLSGIVTRAPGHSPVQT
FAPPAVHTTPTQPTIIRSTPKKTKVYEKHPNEVVKNEELTYSTAPTLCFVRINLIIMELRLHSPVGAEDVVYHWPLTSGRGSDKHDSALEIVETI
RWVCEDFPELKLPLENNILSDFDTKRYGCISVQNEV
>EDO36327
MNVAAMSKVKIVCPNPTSIVDKVQDDTPKEFLYENLWIVTEESYDKCNTTLSPRNKILLKCDTPLSLKYYTVAFQQFSATAQGLEFALGQEYY
FIATSDGTKSSLDKTSGGNCIKSNMRMKFYICKSPQDPRCLPPTTAPPTTPSGCPTVPSTTQTPLPPKTNPTTEPTESNEVPLLPTENSHTKSS
LQSDDPKPTLRPSMAPAVTSIRPQVTKYSSQTEKEAIVREEPCSHDDDPRCLPPTTAPPTTPSGCPTVPSTTQTPLPPKTNPTTEPTESNEVPL
LPTENSHTKSSLQSDDPKPTLRPSMAPAVTSIRPQVTKYSSQTEKEAIVREEPCSHDHGIRPSWSIIIPLLIIIVLLIATNVFFVYQLKQIYTAKDS
NICESLDRRSERSKFTLASIGLGRRKDEASPVNV
>CRE22399
MNPGVLYSAIWLSHFIVSAETRAYHDVIWNSRFFNENFKFTKMHVRLGDQLTIVCPESYHQGMNYEYAKLYWVSEKEWEQCYTDRSQPF
GVCGDEDRTETIKLNFRSRNPIPGGMEFEVGKTYYLISTSTGEAYGINNVIGGLCTEHQMRIAIEIIGGQVPVQQMRYEVHEYQSLSYTAPISS
WLSLLCLASFVLYYYV
>AGAP009426-PA
LTVALVIALQLVALQPTVLHCAKTFYMHWNTTNSIFRIDNTDHIIDVNKGNAQFEYDQVHIICPVYEPGTFDNETEKYIIYNVSKVEYETCRIT
NHDPRIIAICDKPNKLMFFTITFRPFTPQPGGLEFLPGNDYYFISTSSKDDLHRRIGGRCTTDNMKVVFKVCCADDQQQQQQHHGHLPS
NVAGLVGCTATTTTTTSTSKPTKKTKEYDKHPNEVVKNEELTYNNAAS
>Mdes004544-RA:cds

LTVVLLIFLETVLLSTVSNCAKTFFMHWNTSNSIFRIDNTDHIIDVNKGNLAFEYDQVHIICPVYEPGTFDNETEKYIIYNVSKVEYETCRITNAN
PRIIAICDKPQKLMYFTITFRPFTPQPGGLEFLPGNDYYFISTSSKDDLHRRIGGRCSTNNMKVVFKVCCASETDSQNATQSSLDGVSKNSSPT
RQPSNDRSGTIITGNSTDGSSDGRIPPLTVNTNRKSTMTWLSRDNDGSGGYRHNEYDKHPNEVVKNEELTLNHNHGTVMRPYRSIRYST
WFPFCWIPQDFVMLMCIIILVFANYYDYLHTMVQSYIPNLRRTTMLSATTNDDSDGNRMFLDYLKSDAIPGNPMQRSNYNRYKATSEFVE
LRAFKRNNINGIDIK
>EFV61082
MPIYAISFHSPSESASLEAELMDSLEIICPLYNDTVTLDQAEYSIIYMVSEYGFENCLLDNERPVGQCYSPYTETAIKLIFREFTPIPNGLEFEPGQ
TYYFITTSDGSMPGIHKRQGGLCSSANMKFRVRIKPTSNSFRQAIKEARPVVNSELKPDREKSTPATVHPQSMEENSKWAMNSNDVIEDAF
HHSLPLQAETDQTENNNFVIPEVSIYQLNSRNEMEKPWIHHAPRPPLILYEIHEVVNQDSPNSLFAYSHSTAAKSSTLLVFTFFTLTILIVHFGQ
SCC
>EKC32286
MYIKWSVLMFAMMSDILFSQSNSEINVNLDDSIDFRCPFYPSGTSSSSYKYYLVYIVTMDEYSQCRLDTPVRGTLVINCSEPNTEQYFTILIRR
FQSVPGMIDFPPGKYYFMTTSTGYLEGINNKENGACRSNNMRLVMTVRTPESQTPAPTQAPQNSSGPVPTTTTSTTTTTTTPKPVTPSPST
PTPTPSTSTIKPSTTTISTRIEIQTSLEEEKLLKRFYLLDLGVEVVNQEVEGRYPLHFAADYGQAQVIEYLVSKGAKINQTDKHNITPLLAAVWEG
HTSSVEVLLKLGADKSVKSPDGQSLVDCAEKEDVKALLK
>CRE10194
VDFLLTTCSLILLLGSFPHTSADEHVIYWNSTNPMFRNRLPFIEVRLGDVVRFVCPDNEGRSNGEYLTIYEVTEFARDECALESNKREVVRCGV
ETNAEKLIRSHQLPVLNPKQPIPPKNVAQFIRPVNPIPNGKEYQPGSTYYFITTSNGKASGIDQKMYGLCESHNMRLSMKVSPSQPSASTIKP
KAPTRRQEDFVTKSSAEMMGGQEDEDSENDNAHLLPRDLEIATNPKFRRPSQFEQAAVSAGVQDQQLLKVIQMAKEGKTGTFENDRQA
EAHKSAEKEAWHPVSVQYVADLMNTAYKNADERVTYQRDPDFLIHEEDFSANSLEYSSSSRSLRSFIAICVAVVFLF
>PHUM089410-PA
MRFRIDNTDHIIDVNKGNIPFEYDQVNIICPVYTNGTHEEDTEKYIIYNVSKEEYDTCRITNPNPRIIAICDKPYKLMYFTITFRSFTPQPGGLEF
QPGQDYYFISTSSKDDIHRRIGGRCSTHNMKVVFKVCCPPQDVHNQTSTNRAPSTVAAVSTNLQSTTSSSTTTHWHSLSPPRPTSYKDFDL
NRNYKSSTKKTVDSTNDQNEVGKKGKLTNNRSNRCFSSNTILIIFISILRIFLR
>EFV61089
LEPSTRRQRWRLLSSSSTVQTLFRQSEQLKPRSTLVSDDRSFVSTKAMEPFVVQLHDKVDLICPQYKRETLPNLLEFSIIYMVDKVGYESCSIN
STARLVGLCTVPYKKQVVTVVFRRFTPNPGGLEFVPNRRYYLITTSTGKMEGMQNTDRGLCYSKNMRLQFDVKEENYQPVESKNKLDINR
ASTKPFIIWDQPNHRAGENARFTAELNFAHNNPAGNDVYWSNSRFTAPKHRANAYRPARLTERLSICMIKINKTTTAIRKPASALVACKAFI
YTFLP
>KZS17355
MCCCCCCCCRGEKKENFSAVVDEGFRIDNTDHIIDVNKNNIPFEYDQVNIICPVYTPGTHEDDAEKYIIYNVSKEEYDTCRITNPNPRIIAVCD
KPYKLMYFTITFRSFTPQPGGLEFRPGQDYYFISTSSKDDLHRRIGGRCSSHNMKVVFKVCCKAEDLDKGTTAGRPALSSTTTSTTMSTISTSV
AVMNSTTTQAPVVPTRIITTQLLLPSVTSPRMPPHWKDVEIQSFSVRTTTKKSDDFDKLSNEVVKNEELTSKSSGARRQVVHNPVSALIAAG
LVSLILFRVR
>FBpp0405637
VTLLTIICIETVLLSTMSSCAKTFYMHWNTSNSIFRIDNTDHIIDVNKGNLAFEFDQVHIICPVYEPGAFENETEKYIIYNVSKVEYETCRITNAD
PRVIAICDKPQKLMFFTITFRPFTPQPGGLEFLPGNDYYFISTSSKDDLYRRIGGRCSTNNMKVVFKVCCAAEEKNKTTAPTTLIENTGGAGT
GMGTGTGSVDNTAINVDVNVNGVNTIGVNTANAGNTGNIHLKPINGLGTSINTNIDQFNRIPIQPNIIGNNIGQSGVGGGAGGVILSPGH
GSINMLPPGRGGIHLTYPGHHHIQTGIRINNVPTNVQQNQNNPNMHSHPPPPHPHHKQQAGNNNLNANGNDDHHGYDKHPNEVVK
NEELTYNSGTQQSHLCKWNWNWIIAIITIIYR
>CBG01610
LDFLLAICLILLRSSTFADEHTVHWNSTNSMFRNRHPSIEVRLGDVVRFVCPDNEGRKNGEYLTVYEVSEFAMGECALESNSREVIKCGVDT
NTEKIIRTHQLPIGESREPPKNVAQFIRSVNPIPNGKEYQPGQTYYYITTSSGKPGGIGQQMYGLCVSKNMRLSMKVLSSQPTPSPSSKPARS
RTDARRQEDFITKSSAELMGGQEDEDSENDNAHLLPRDLEIATNPKFRRPSQFDQAAASAGVLDGQFLKVVQMAKEGKTGTFENDREVQ
KSAEKDAWDPINRHYVADLMNSAYKNANDRVVYQREPDFLIHEEDISTNSLGYSSSSSSSLPTFLIVFLIAVNLLF
>ISCW018996-PA
CVVVVFCSFRIDNTDHIIDVNKGNIPFEYDQVNIICPVYRTGTREEDVEQYIIYNVSKEEYDSCRITNPMPRIIAVCDRPHQLMYFTITFRSFTP
QPGGLEFRPGQDYYFISTSTGDVGGLHRRIGGRCATHNMKVIFKVCCDPSK
>KNC32890
FTLLTIICLETILLSTMSSCAKTFYMHWNTSNSIFRIDNTDHIIDVNKGNLAFEFDQVHIICPVYEPGTFDNETEKYIIYNVSKVEYETCRITNADP
RVIAICDKPQKLMFFTITFRPFTPQPGGLEFLPGNDYYFISTSSKDDLYRRIGGRCSTNNMKVVFKVCCASEEKNKTTTIATGGSAGGSAGSTS
TTAVGSALPTDSAGNSGEDITNMHSNQNNQQQQSNHPHHGNNINTIGINGINTGMLPNGVMNTGLGGMGNNQHNGLQLNPIMNSP
SGNVATSINTNIDQFNRIPLQPNSINVLSNNIGGGMGMGNMGPPGMSGNNGLGLGSHGGSGILLTPGHNGINMIASGGGAGGLPGIHQ

YPGHGYHGQTGIRINNVPSTHHTNLKNMQNNNNDNDHHHPYDKHPNEVVKNEELTYNRAAASMTTATALSLIFVLTLALQICYWMIPILS
KLSGFYSSYVLGVLTIGYILGELGHYLIGVTSKQTAMELDYGDISCQQNNSMFLRTEVPTQCADIKNETSCVASEVNGTAYCEWNYNGLGIEY
QILAGPTFILIFTIAGVFMGIAADKYNRVKMLTVCTLVFAVAIILQGTVKEYWQLALLRMIMAAGESGCNPLATGIMSDIFPEDKRALVMAIF
NWGIYGGYGIAFPVGRYITQANFFGMGWRVCYLGAGILAILIAALTGTTLKEPERKVIGDNEMRTKDGKKANLWSVLKNPAMIMLMIAAS
IRHSGGMTFAYNADLYYNTYFPDTDLGWWLFAVTIGIGSVGVVVGGVVSDKIVAKMGIRSRAFVLAISQLIATLPAFGSVYFDPLWAMITL
GGSYFFAEMWFGIVFAIVVEIVPLQVRSSTIGVFLFVMNNIGGNLPIFVDPVAKAIGYRGAIMIFYAGMYGISSILFLITCFMLEGKPQASAEE
KAENGLEVPARNIQGHDNSVFSNDEPPAYPNGKSNTALPNHLQMYNNNTNSHKPQIQESSRL
>FBpp0122928
VTLLALICMETILLSTMSSCAKTFYMHWNTSNSIFRIDNTDHIIDVNKGNLAFEFDQVHIICPVYEPGTFDNETEKYIIYNVSKVEYETCRITNA
DPRVIAICDKPQKLMFFTITFRPFTPQPGGLEFLPGNDYYFISTSSKDDLYRRIGGRCSTNNMKVVFKVCCASEEKTKNTTTSKSVGITDTSNV
SSYDVGHNYLNGKNIGLGSNIGISGVSSGIVPPFSINPPNGNSDVNSNSIGPSINTNLDKFNRIPIQPNIIGNNIGTNSNGEVNVASNAGILPA
PGHGSINMLQPGRGGINIPYPGHHHIQTGIRINNVPTHHSYPPHKGNPNSNSNDDHHYEKHPNEVVKNEELTYNSGSSSRKCRIYSIWIRLF
SWLSLTLPSFCCMAAHWISSPVALSIAAVLGMHYIAGKAIITPQLYRSENILCTPSCINNFRVSSTATNKYVC
>AAEL004488-PA
LTLGLIVCLQLLMMSTVLHCAKTFYMHWNTTNSIFRIDNTDHIIDVNKGNSAFEYDQVHIICPVYEPGTFDNETEKYIIYNVSKVEYETCRITN
HDPRIIAICDKPNKLMFFTITFRPFTPQPGGLEFLPGNDYYFISTSSKDDLHRRIGGRCSTNNMKVVFKVCCADEQHQHPNITSSNNNIPSST
AIDGGSSPNGNSAVSGSSGVSVNTNASGSMDGFDPSTRLLPSSTVNSSQGNIVQSTVNWPVWNGGQQRPVPSTPSVNNGGNGRHQH
HYTPSPPRTAINNYNNYGGNSGSGSGAHSNSNNYNINNPPSNAHQNNKPTKKTNEYDKHPNEVVKNEELTYNNGAATRIATSSRGLLGLV
LLAICSHLLLSSSRLCVTGLPNALPLTR
>FBpp0182251
VTLLTLICIETVLLSTMSSCAKTFYMHWNTSNSIFRIDNTDHIIDVNKGNLAFEFDQVHIICPVYEPGTFENETEKYIIYNVSKVEYETCRITNAD
PRVIAICDKPQKLMFFTITFRPFTPQPGGLEFLPGNDYYFISTSSKDDLYRRIGGRCSTNNMKVVFKVCCATEEKNKTTQTTTSVAVSGADPG
GGNSVNIAAHDDSHGHVHGHNTGPGNTIGISGVNGGLSGGSPSVGIATNPNNGPMNGLGTSINTNIDQFNRIPIQPNIMGNNVGSSGV
GGGIILSPGHGHGSINMLPPGRGGVNIAYPGHHHIQTGIRINNVPTQHSYPSHKGNVNGNSNGNDDHHHYDKHPNEVVKNEELTYNSGS
AITARNYFNVWIWLCSWLPLPSSLAVCMSAHWINSSLVSIFAILAMHQVIRIALPTQRCRPENKAFSPSSKNCQVTNTKSNR
>FBpp0163267
ATLLTIICIETVLLSTMSSCAKTFYMHWNTSNSIFRIDNTDHIIDVNKGNLAFEFDQVHIICPVYEPGAFDNETEKYIIYNVSKVEYETCRITNAD
PRVIAICDKPQKLMFFTITFRPFTPQPGGLEFLPGNDYYFISTSSKDDLYRRIGGRCSTNNMKVVFKVCCAPEDKNKTTEITTMLGNVPAVDS
GGTSNIHKGVDNAAVNIDPNRNMNSPNGIHTIGVGVGSGSAAGGGGQMKPVNGAGTSINTNIDQFNRIPIQPNLIAGGGGAVGTGVAG
GSGIMHSPGHGSINMLPPGRGGIHMPYPNHHHIQTGIRINNVPTQQNNQHPHHKGNLNNMNSNDDHHNYDKHPNEVVKNEELTYNS
GSGQATLNRPIDVYLWSWSWLPGGTITTTILIAAIFYTFHYLFWTLNNTMARRIETLGIYR
>FBpp0394582
VTLLTIICIETVLLSTMSSCAKTFYMHWNTSNSIFRIDNTDHIIDVNKGNLAFEFDQVHIICPVYEPGAFENETEKYIIYNVSKVEYETCRITNAD
PRVIAICDKPQKLMFFTITFRPFTPQPGGLEFLPGNDYYFISTSSKDDLYRRIGGRCSTNNMKVVFKVCCAAEDKNKTTETTLLGSVPAESGN
GVDNAGLNVDQNLNANANHGHGHNGVNTISTNTGFIPGGSAVGSGSGGSGGGVQLKPISGMMGTSINTNIDQFNRIPIQPNVMGNNI
GAAGGGASGSSGTGGIMLSPGHGSINMLPPGRGGVHMNYPGHHHIQTGIRINNVPTQPNNQHPHHKGNMNVNSNDDHHNYDKHPN
EVVKNEELTYNSGSGQATRSHIWIWTWLAGGAATQGLTSMHAYGINLTLLLAIIVITFQYLFWAPAAYTMRRRPEPLGINYR
>FBpp0088144
VTLLTLICMETVLLSTMSSCAKTFYMHWNTSNSIFRIDNTDHIIDVNKGNLAFEFDQVHIICPVYEPGTFENETEKYIIYNVSKVEYETCRITNA
DPRVIAICDKPQKLMFFTITFRPFTPQPGGLEFLPGNDYYFISTSSKDDLYRRIGGRCSTNNMKVVFKVCCAPEDNNKTTALSNSKSVTDTGG
AINVNIANNDESHVNSHGNNIAIGTNIGINGGQIIGGPQSAGIPINPLSGNNNINGIPTTINSNIDQFNRIPIQPNIIGNHVGTNAVGTGIVGG
GGIILTPGHAHGNINMLQPGRGGINGAYPGHHHIQTGIRINNVPTQHNYPSHKGNANSNINGNDDHHHYNKHPNEVVKNEELTYNSGA
ATSDGNIFALWIWILSIFPLLSIQSCHLSSYWISASFLVSTIAILGIHYLIQITLQTTVQRYSPGMVEITATSMNGMFDQNAGTIEYDR
>FBpp0242833
VTLLTLICIETVLLSTMSSCAKTFYMHWNTSNSIFRIDNTDHIIDVNKGNLAFEFDQVHIICPVYEPGTFENETEKYIIYNVSKVEYETCRITNAD
PRVIAICDKPQKLMFFTITFRPFTPQPGGLEFLPGNDYYFISTSSKDDLYRRIGGRCSTNNMKVVFKVCCAAEEKNKTIASTTTSVAVAGSDG
GVGGGGGGNVISNAATNDDHNPKHSHGHHNIGGNTIGISGVNTGLISGGSGGNGAGGAGMPINANNGLGMGMGTSINTNIDQFNRIP
IQPNIMGNNIGSSGVGPGPGLGVGGGIILSPGHGHGSINMLPPGRGGVNVAYPGHHHTQTGIRINNVPTQQNYPPHKGNGGNSNANG
NDDQHHYDKHPNEVVKNEELTYNSGSSRTSGHCIGQSATALLIILVTLAIQRLIIIHPASNIRPTHRPYTISPPLLLPSPAGINASR
>FBpp0363337
VTLLTLICMETVLLSTMSSCAKTFYMHWNTSNSIFRIDNTDHIIDVNKGNLAFEFDQVHIICPVYEPGTFENETEKYIIYNVSKVEYETCRITNA
DPRVIAICDKPQKLMFFTITFRPFTPQPGGLEFLPGNDYYFISTSSKDDLYRRIGGRCSTNNMKVVFKVCCGPENKNKTKAISNSKSGTDTRG
AINVNLANNDDSHVNSQGNNIAIGTNIGINGGVIIGGPQSPGIPINPLSGNSNLNGIPTTINSNIDQFNRIPIQPNIIGNHVGTNAVGPAIVG

GGGIILTPGHGHGNINMLQPGRGGINGAYPGHHHIQTGIRINNVPTQHSYPSHKGNANSNINGNDDHHHYNKHPNEVVKNEELTYNSGT
ATSDGLFFALWTWIFSTLSLSSIQSCHLRAYWINSSFLFSIFAILGIHYFVQSTFQTTLRYRPEMVKITATTKNGLFCQKADLFKYDR
>F56A11.3
FEFLITTFLLLGLAAADEHIVYWNSTNSLFRNRQPTIEVRMGDVVRFVCPDNEEGRNDGEYLIVYEVTEFAMDDCALESHSREVIRCAPEGT
AEKVLRTQQLSGGRREDWKKQKVPPKNVAQLIRQLNPIPNGKEYQPGQTYYYMTTSTGKANGTNHRMYGLCESQNMRLSMKVSASQP
HPTRRAPTRRQEDFVTTASAELMGGQEDEDSDNDNAHLLPRDLEGSTNPKFRRPSQLETAGVENQQFMKVVQMAQAGKTGTFENEKE
AIAQKSSEKDGWHPVNVQYVADLMNNAYQNADERISYQRDFEIHEENDLAVKSLEYSSSSTSLSTNFAILLAVIYVLY
>GSADVT00010381001
FKELYLFLWIYLFLINMVNTYHTFVIHWNRTTFMPYQDGSLVFEVRLGDVMDLICPFYDEQQSYMTSELEQYDIYRVSENEYQSCNINQYNP
EPITRRIITCNSPYDVMKYTLNFRSYLPIPNGFEFRPNQTYYFLSTSSSHSQCNKLKIFVHDYRK
>tetur15g03520.1
MWFHHFLKSTVSKEEYDSCRITNSNPRIIAICDKPHQLKYFTITFRSFTPQPGGLEFKPGQDYYFISTSTGKREGLDRRLGGRCSTHHMKVIFK
VCCSTNDNNVLNDKSDANNIKNNSNINSTINNNQSFSSTTVDSSSLSSSELSIESSSSSSIASSSLSSADLHSDDRSNANGLYLNANINNINSLV
STSSPPSTSTTLGSLVTSTFPASPEHWWRPHFTRIQDLKWRSKGPGNVVKETNHHHLTVGHDSNSFTNPNANNSQNNSSNDTLRPIAWTI
FAIACFIFIIFWALWCKVRRNNVMLPMSK
>EJD76078.1
MDSMEIVCPTYSMHDNIDKYEQLIVHQVSDLSFMSCELDSRSQAFLICDSPLATTSISHKIVFRQFSPLPNGFEYQPGRSYYLITTSNGSTEG
MNNTRLGLCVTANMRLRIDVRPLSDDSYPSSEEGMDMKSEVLESLEPKRTGEEDWSRDRRIKPPTADNAKTEQDIDMLNNESQKFAEKH
GFDLMRLEYVRQLAIDGVEGDFPFRELENDSMRVSMGSPETKLASGPGRPSFSSTLSDRETLHSTSRQAQRWISKEIQSRFGASRDVFPDQ
NTSGIESAKDYVVDDVSGGGAIFGRLEAKYEGNTNYFCDYKRGLGVHTLWVHTISDNPKSRSRQVGCTQYSAFPEMLHYYNTD
>HelroP194703
LLTPKMYQLNVLRVQREMSSLKIRQLNLIIFIIISFQMTYLTFKWTERGGHTMEVRLFDQVDFICPYYDDNLKSDQKRFERYIIYQVDEKSYTT
CHADGAVLILNCSSPFVKKRFTLLFETFLAIPNVPEFVAGGKYFFLSTPADANKNKSGHNSKEACRENMKIIVTVCCSTTTNTATITTTASTAVII
TTASAAATITTATSTTTTFDFASNNAMYNKFKRTNASSLLLASYGKVLNQELLINHAIPALHPLHHPPTSLNPPLNCSSSDPFLNI
>Bm11673
ITLLRFFIVPLMTSATIYNISWISTNPLFSSGRNNIPLFMENLKVNDRIRFSCSTNGTEQTAIHRVNERGALNCRGPAKFINSLIGTCMKANDSII
IRLREIPMLPNEMSYKPDHDYFFITTSTGDKDGLENDEGGLCRTENMRLKIRVESKWTRISQKIPEFTAKSGPVMMIPVNSGQYAVPVVYIP
VAQSSETNEGDKKRPMFPYHLYFANGQFQATNSNLEDLGSQAEKFDAGDRPKIFKHVSFAINSNGYIAPKFLKEKEKDLDFEIDYMDSATR
RLYEKFGNVENTIIISSCIAIANVLI
>GSADVT00033567001
INELVLVLIFLFIFLIKQPIHSVILRSLYWNHTTFRPYQDDSLIFEVRLGDVMDLICPFYDEQESYMLNDFEQYDIYRVTKNEYDLCSIDQFNLDP
TTRIIITCNSPYEVMKYTFNFRSYLPIPNGLEFHSNQTYYLLSTSTLSSSHQQCYKLKINVHDYQRTLASSSTVPSLNEKERHSPIISHKSNPRIGS
SSSSSIDKAIEYNEHHHLFSKIVKAERHSDPYSIKWITKVPSSIKDFEDKTYRITNSISSSPSNTLSLALSSSSSINLSVSFINILILTLFSI
>HelroP169042
MPTLYVRFKKSTNLPSVEIMMGDYIEIICPIYSNTTDASVMEVYVLRWVSEEEYRGCYVKNPNSKIFQCDTPLKRNKFTLAILPNPSVPGQMT
FKEDTRYYMTSTSTGRSEGLLNKEGGVCAERNMKLIFYVPKLHFNIASSAVSIDGKEDNSA
>CBN08149
MGSSYVFGYMNGYDTNHESFRNEHPTIEVRMGDVVRFVCPDNADRDEGEYLIVYEVTEFSMTDCALESTATEIIKCGVDTKKAPEPKNVA
HLIRQLNPLPNGKEYITGQTYYYMTTSSGKANGLNHRMVGLCGTRNMRIAMKVLPSQPTPAPYVKPMTPTRRQEDFVTKSSAEMTDGQE
DEDSENDNAHLLPRDLDVASNPKFRRPSQFEQAAASAGVHDKQLFKVIQMAKEGKTGTFENEKIANSHSSLERDQWNPVNVQYVTDLM
NTAFKNAEDRISYQRDDDFMIHEENDIAMKTLGYSSSVSSIPTLFVILLVALLLF
>EFV61087
FYLHYPDVKVVSISPPVTAEICSRFTANSAGLIVYADIRDSVDIHCPKYTDTVTQDKAEHSIIYMVSKFGYDNCILDKERIVGQCSSPYVDSIIKLT
LREFTPMPNATSDGSAEGMHERSDGLCSFNNLKMLVHIRRGREDNHRSLKKQTNIHHPQWLLKYRDSLPGSATTNSPGSQMSNDMDNA
RQKTSPNQDVSKAEQTRRTSRPVDDTVGSYRVDVPLSNRKQLEDFVYPNTILRRQPETESIILYEIHELSNGELEALGLSSSSNAKLKGSIRCTI
VGAVSVLLLSMNW
>SRAE_X000209500
NYQEYIAEKIYDIKNDENGNYFNFQWYSKNPKLQEAINENYLETENVLDKENYLTIKINASTIFRFICPESKNYEKSDVKINLGKHQTDYFNEYT
KIYAVSKRSFIKCELTESSKLIGECINNNKKKHFDFITARHATELLRDFYFITTSTGNIDGFHNKKDGLCKTKNLRLHLRVLRESTFDEISEYEKDYE
KQKIKLQNYNSNIRQSEEFNHDIIDFRQTDKKGTMYLKKDYNTNLYKKLEEYNNHHKNLNEKQFDKNDNEVESIPPSDDDDVITSEEYDLLF
NEQSDVLSDIDKKNIQLFKEKQFKKFETQRQVEDTINDSKLYENFRFHKNSYVLPDNNDYDSDEYFGNKKNKHINHSKKEKHFITMERILND
DNITKYAQLTSTYDRVRLFDSPFAYLVINKEVNRYCSYCLQPPIYGKKLMRCGACEFACYCNKECQKLAWKTHRAECRRLKAVFPNLPLTEVL
FLSRIIDKVLFLERHGDHFGWERYRKFSDIMSHKEDILNDKLKIEHFKKLVKKMEIYRKEEMIPEDKFFDIYCRTAINSHSIHTNAGSEVGIALDL

GVSIYDHSCRPNCSLVFDGFKVYIRPLTQSANPYDPKSAFISYIDVGRSRYRRQEDLKTKWYFDCKCERCIDPKDDILTSLKCKNMDCDEPIIT
HELGEVKDIECPKCKKICDSEYVKKGQELMKSLPSSIDPTLKVDEIQKYLDEVNNILHNKNIYVSRLTTALLHMNGTLQGNIDFVQKQVYENY
KLCFPTMDRHNGFQLLHIVKSLIEQDKRNEAIPYAFDAMTIFEVCFGMQHPYYLQTLALWTFLEKKIDKTNEELFSLMNFESNAAIDISKYIG
DIKLNPNDAMKMASQNQEQIQP
>SRAE_X000065200
MKILYQIFSYIFLFPLLTISVKIHNLPLKFKDGILTDISRLVMKVGDKLEVHCPEKMHFTLYKVSSSMGEACVIPAHSTQIVAICKPGITGKIIIRSST
VGRMMKATFTEGEDGYIISVNSGLDEKIKANSDENQYGGLCLKSNLKIPFSIVTEEVPKEDIINVDENINTKIENFINNPVTRLPGSRINVPSEE
RNPIVPRQSSGRIPSDRFKYSSINEDGKFTNANKEAKVERYMVDKVKLYLDVKAAIDNNPNLKSKTKVAFSKVENYFRGLIQFTEKEIRDLIELF
ELHQDSDYDIGYEELTRDSSGSGTTIIIVSLLAAFMFLTGLTIIVIIKHLK
>Smp_129000.1:pep
MSYTFSSNVTMFRVKEGDNIVFVCPSDVRRSQKLFWTMSKETYDECGAPSSSQTVKLLDCSQSPKNTEFILKVAQFSEIPYSPHFRENDFVY
FLGQHDLCSIYNLRIAVKLVPDKSVNNNEELSTSTQKINNLVDDKDKSPNTVTSDTYKLNPHRTETLSAWTRYRFLLIPGSLGFLTLIGIQAVIC
ALWHPKKSSLCRKCCSSHIVNKSNDGTNDTNEMSTTPIQMLEKSFTRKPFENHQCQSIQTSTLITQSTLNDQYNKGDFINNSKRNSDIVNIL
HDTYTLDKAMNSFYTSINTDPQTTKIKDKNQMNLSELIKCLE
>Smp_133090.1:pep
IELIMILIKLIIAEYKDHIIYWDASNSIFEENPDELYVNEGDNLIFICSTNRTHVRNLYWRKRFIVSDFLRYNPFLFIKNNIRLSFKLAKNSVNDKN
NSLVSDVLMFPISKSQLFDQNKCKLN
>PPA18651
AALHSQQWKQWMDGTKSSPPASPPKRKYGQYALQLLHWSTNLTTFFELNDHNITIDLYTEDLAIVCQTEDVDSVVRLIPAAMADSCAFEG
GVVAHCPNYLRVPLLYADIRGIGEYVLTSFSDGTAEGKHATSGGFCKEGLKIPVAIVGRSRDWSRFREKEEKSTTIRPREVFHSMPDEDSSSV
HFFSSVSVTISMAMLLFFVLL
>tetur15g03500.1
MPWIIVLLTLIEFSSAKLASIGIYWNASNPIFRIDNTDHIIDVNRGNNPFEYDQVNIICPTYTEGTNEEAETYIIYNPNVFSSILASLTVA
>Smp_133080.1:pep
MAFKEGQNEIYVNEGDNLIFICPPNRTFSQSLYWTNDSRVTIKCNRTLSIKVIKLLDCFGDNYATEFILKISKFHEISSLPVFHHELPIHFVAQSVI
CQNSNFRLSVRLASTHKTSSTDSVSNGSAIYIKKSNLTNHYEKSTTSNKSSYFHIKRKIAKFISNVSSTSHLNYEFTNSEQTTWKEYRFLILPATLA
FFTLIGMQIVFCSFWLPISVINKFFKHFRPSKHLHKLKSQSNQKVKQNESNSLKVCKENSKIPNQSLCWGIPTQTSMNSCATHSMKEFKYDK
TVNYINEGDNLLFICPLNRTFPENLH
>CRE14007
MSKYFNKELENLAYGKCVEADVNCEVFVRQTISFCYREFGEAYCYELVPSLKPTTTTESTTTTTTTTTTTTTSVPTTQKSNTGMLIGMSIGVIIL
LLVIVICWVRCWLKRRRARRNQLTIADGGETGANGGKKRTGKGTTTGTTKTGKTGKTKKTKGTTKTGKWTTTGHTKTGKTKSGFFKKNKK
TKTGSGSGI
>ADAC006258-PA
LTIALVIALQLLALQPTVLHCAKTFYMHWNTTNSIFRIDNTDHIIDVNKGNAQFEYDQVHIICPVYEPGTFDNETEKYIIYNVSKVEYETCRITN
HDPRIIAICDKPNKLMFFTITFRPFTPQPGGLEFLPGNDYYFISTSSKDDLHRRIGGRCTTDNMKVVFKVCCGDDQQSHHGHLPSNGTVIGG
GGHGGVDGGTAIGAGGPGGAANANNTISIDAFDPNSRLLPPAINTSQGNIVQNTVNWPVWGGNDGQGGGLNNANNNNGHPQQPH
QPQHLPPSHGGGIGGHPGGRPGVAVPPNGRHNHHYTASPPRTSINVPGGNGNGGGAYGNGNINNHNYNINSPPSNAHPGSKPTKKTK
EYDKQHPNEVVKNEELTYNNGAGGVGARSRHLVTLLARERLQQEKQEEEVEEEPRKEQKQRDRGTERWCTQAMHLILVVLSFRFQGSRR
FLVALHYANRLQNSPFQQRCSVNSATTKVTRRSSQKIEKRTVHDLKKKDVSWESVHEEQQDGGEGCERTDKPHTDSLWTGVGSQEE
>AGLA008721-RA
LRGLILLTFSYHWFALATLPSTAKFYTIHWNSSNPIFRIDNTDNIIDVNRNNVKFDYDQVNLICPVYMPGTRDEEMEKYIIYNVSKDEYETCRI
TNPNPRIIAVCDKPYKLMYFTITFRPFTPQPGGLEFLPDITSRHNVRHYLQFSGCLRPVGIKSKDVFHKRDNLTQTTCDSLN
>BIMP16439-PA
MVARSVGSYAKRAIASGDSTTRDGISVLFKQSGRRKNCDKRETECDRQRDPRSNLYTGSLVLYAYNREHFKPVEIYARKTYLSARVSKEEYET
CRITNPNPRVIAVCNNPYKTMYFTITFRPFTPQPEGLEFLPGHDYYFISTSSKDDLHKRIGGRCTSNNMKVVFKVCCRNEADTSSSSATSRNN
SVHTICCG
>Bm2726
MDSMEVVCPTYNMHNNTNNYEQLIVHQVSDLSFMSCELDSRSQAFLICDSPLATTSTSHVIVFHQFSPLPNGFEYQPGRSYYLITTSNGSAE
GINNTRLGLCVTANMRLRIDVRPLRDNSYLNSEEGMDTKSTALESSEAERTEKEEWSRSRRVKPPISDSVKIGQDIDRLNNESQKFAKKHGL
DLTRLEYVRQLAMDGVEGDFLFRELENDSVKTSLDSSETTLDSGSGRPSFSSTLTDRGEVPHSTSRQAQRWISKEIQSRFNASWDIFPDQNR
SGMESADYVVDDMSSGGAAIFEQLEAKYVIIIALLLCSSGR
>CJA07765b

MQISILLLLSLLTSLGWTRKIPDINWISTNPIFDVSNTDHVISVRIGDRVSIKCPKSDGAGKYEYSYIYMVSDEEYDHCFLAKPRLVGACDNQTI
NVKNLTLLSAIQFYFQASINIVFRSFTPTPGGFEFQPGHNYFLISTSDGTQEGIDRKKDGLCTTKQMKIKFEVESDRRGIESPKFAARTFKKDRD
VEQSTPVLYLVHDGSDIDDDDGGSNACSYLVSISALLIYRYLL
>CJA14032
MGDVVRFVCPDNDGRKPGEYLTVHEVSEFAMEDCALESGSREIIRCGPDGTIEKRVRNQGRDGEQQRRKIAPKNVAQLIRHLNPIPNGKE
YQVGSTYYYITTSTGKAEGIDHRMFGLCESQNMRLSLKVAAAAHHVRPMAPTRRQEDFVAKNSAEMMGQEDEDSENDSAHLLPRDLDV
AVNPKFRRPSQLEQAAASAGVQDQQFLKVVQMAKEGRTGTFENEKKLEEEAKKSAEKDGWHPVNVQYVADMMNNAYQNVDASLSYQ
REPDFEIHEDSDLSVKSLEYSSSTSMISTFFSILTALLFALI
>CJA19195
MHPVPISETILLLLLLFFCTPTSTKRLPQIYWNSTNPLVERYAAIGDTLDIVCPFFEENTEQLTEESIIYRVSEEEYENCERRSNSKELGRCTQPHQ
QEKLKVAFRLMSPNPSGLDYRPGVTYYFISTSTGSRKGLYNEQGGLCASHNLKMVIHITDKNGDIGPHHHRHHHKKTTTTTTESPVKPEPLL
TSSQIATLDTSSEKLWEQFYEKVLPIENTWPEISTRGERVTLYQGSKKDEYEQVPAEVVDFEIHEIGDVESLYSSSGSSRSQGLFMLLLPATLL
WLL
>CPIJ001112-PA
LTLGLIVCLQLLMMSTVLHCAKTFYMHWNTTNSIFRIDNTDHIIDVNKGNSAFEYDQVHIICPVYEPGTFDNETEKYIIYNVSKVEYETCRITN
HDPRIIAICDKPNKLMFFTITFRPFTPQPGGLEFLPGNDYYFISTSSKDDLHRRIGGRCSTNNMKVVFKVCCNDDQHPHPNVTSSVNSIPSST
AIDGPSAGGGDGQNAASGSRGVSINTNASGGSLDGTSVDPSTRLLPSSTVNSSQGNIVQSTVNWPVWNGGQQRPVPSTPAINGNGRHQ
HHYTPSPPRTAVNNYNNNNYGGSGSGSGAHSNSNNYNINNPPSNAHQNNKPTKKTNEYDKHPNEVVKNEELTYNNAAATRTRGMLGL
VALLVCATQLLLVVHSSSSSSWSSWSTSSIEEEPFLEVVVAGYDDDDADGGNRKWENVRKSDNGLRVACTLGVKGSFAFSARRAEQEPERL
EWFGFEKLADDKARKSELTLKFELE
>EFO17917.2
VNEHAAVDCRGPAKFVDSLIGTCTKANDSIIIRLREIPMLPNEMSYKPNHDYFFITTSTGDKDGLENGEGGLCRTKNMRLKIRVKSKWTDVS
QKNPEFTAKNGPVMMIPVNSGQYAVPVVYVPVAASSEVNEGDRKKSVFPYQLYFGNGQFRITNSNIENLIPRA
>EFV49446
MVIREFTPNPSGLEFKPGRKYYFISTSTGSRAGLANRNGGLCSKNKMKMMFDVRRSDARTKIKTDEYFFITYHTPGMVGEDYQEDAEEHLL
VNEKEEEDIEEPIVEENVDWAIEPETTTPLLYIIHTRPYDEISASWYNRDKFGKMFYMDDENPILISNTLTRRAITKLALLYRCLLLQSYCSFTTG
NDIS
>EMLSAP00000001309
FICLAILLRTSSAGYGLTKEVIILYWNTTNPIFRIDNNDNIIDVGSSPMVYDQVNIVCPVYSPGSISEENTEQFVIYHVNKEEYDTCRIMKPYPRII
AKCNTPFKPGYFTISFRSFSPTPGALEFHPGKDYYFISTSSKDDIHRRLGGSCLSNNMKVVFKVTDKTATSTPVVKTSTPSINTERNYYAAYNST
SQRIDPFYVRDTYYNDDGKEELPKKQRRRKKPWKNSSQNSILNDQTSREASYVEKVNNLMKQEVIRSNDSCHPRYSLAFLSFLLFAISRIRXN
KSKICELTLEYKESFKKRQSNNTPSPKFFWFLFDVECYTSLFLPIYI
>EMLSAP00000004326
MFIHYLLILLGFYGSAYASKKVHYLHWNRANPMFHPRNNEHILNVNQGNLPWEYDQVNIICPVSKPGVHYPETHVIXSVSKAEYESCRITNP
KPRIVAICNDPYKVMYFTITFRSFTPTPGGLEFKPGADYYFISTSSREDLHRRVGGGCASHNMRMIFKVARNDHFQGEDSNNDESFTQSPLP
PHNKKKTXATAFSPPLFGRHRIMYDSRVPSTKGADDYIYYYNPRNLVNMEEKFKKKMKELDEMENEIWGLNEEALKLTSASSVWRISHILSL
LPFVTLLLLRRSS
>EMLSAP00000004327
ITLRVLLALTAFPVSSIGSKNIFYIHWNRANPMFRQDNRDHIVEVNKGNQQWEYDQVNLICPTSKPGTSLYPETHVIYSVSKEEYETCRITNP
NPRIVAVCNSPFRLLYFTLTFRSFTPIPGGLEFKPGHNYYFISTSSATDLHRRVGGGCASHNMRMIFKVAGEDGTSFPQDDYLPRKTNPLFES
FDNRRNNDVYGSSYYSSKKASEYSSRFLYAAPANYQPKEEILSNHIEEAQKFLSNDSSRLMGSLSYPSLLLAFTCMLYVSLY
>EMLSAP00000004329
HVFNMLLLRLSFLLFVISKRGSGKQVHEVHWNASNPMFRIDNTDNVIDVNESNGVFEFDXANIVCPYYQGTSSPQTSDEEKETYIIYNVSKE
EFDSCIILNPNPRVIAQCDHPDKLLYFTITFRSFTPTPGGLEFKPGNDYYFITTSSKGELRRKNGGRCNSHNMKVIFKIAPLEKRNIVKTDDAPL
EEKLSNNKLYPSLQSPKTRKMYKEDLYRRKNQKRRHEETMFKQEASRXPSSSPSIHGVFSWTLSRLLLPIITLSFLHLSLVS
>EMLSAP00000004379
TLFILIFIILPSEIHFVQSKELFNIYWNTSNPMFRKNLNQNVIDVNVGNHPWEYDQANIVCPTYGSGVREADIETFIIYNEEYESCRIRNPNPRII
ALCNSPYKAHYYTITFRSFTPTPGGLEFNPGQSYYFISTSSRNDLHRRVSGRCLSNNMKLTFKLAPLPSSAEDALKVPSINVPRITSSPSPSTKTD
YNYYYEYPLNDIKNEPRPEIFLQDKKLKSSVKQEASVMSNARRVSEIGSPYIITIHFALLIHTLT
>EMLSAP00000007655
LSFRILTLVSLFKICLSMRQNHVIHWNTSNPIFRIDNTDHILDVNHGNQPWEYDQVNIICPNYKTLRGTSSDTEDEEKYIIYNVSKEEYETCRIL
NPNPRIVAVCDKPHELLYFTISFRSFTPTPGGMEFKPGKDYYFISTSSPNDLKSKNGGRCSTHNMKIAFKVASRDHERSSSSPIESLSNKKNVY
PLSINTPRRRRPFTTRMPPTRILDNRFGKILPPIYEEAQFRHPNDVVKQEASRMEVITDSHPDPLSGSNKPYFFLHWVWY

>EMLSAP00000009373
LQYNLLFTLILFLLSPLSSDSTAIHNLYWNVSNPLFSESNEHLTLDVNGGNHPWEYDQVNLICPTYPPSSLPPLESHIIYSVSREEYETCRVTSPS
PKIVAXCDRPQEXLYFTITFRSFTPTPGGLEFQPGKDYYFVSTSSREDIQRRIHGYCSSFNMRIIIHVAENSKKLNSSSPEVDRETRQSDGVSDN
PIILQTVDQRKLPNVVRKEQRVSSGSGMWSIQTFSTLCLCLSIRLL
>ENN77760
MGLPKMQRTGHDYYFISTSTSDDLHRRIGGRCTTHNMKIVFKVWGPPAQTPTQPPTQPPPRPTWSSMTPPRPTPSTTTMTTSRSTVSTTK
KSKAYNKHPNEVVKSEELTLGGTSDASVLLSNAILICVMVLRALQSMR
>ENN77761
LRTVVVILFSLQAFVLATAAPSSAHFYTIHWNSSNPIFRIDNTDNIIDVNRDNLKFEYDQVNLICPVYTPGTRDDEMEKYIIYNVSKDEYETCRI
TNPNPRIIAVCDKPYKLMYFTITFRPFTPQPGGLEFLP
>HelroP177176
MAFVLLTMIMMMLVSENDYRNCIMYNEREALLVLNCSHPYSKRRYTILFESYPSIPNAPEYKTGESYYFITTSTGNHHGLNNVYHGACRQH
NMRIKIKVSDRLPNIFYDDEEDNGDEDDDDDDDDEDYNEEDDDNDEGDASYGGKRNKNDDDDPFGTTMKITTATVTVKSNVHNISRPPP
AKKVGEGGGRGQHKQKHQHQHQQRRMQITKAPTFIDNGYIFNFNDDDDDGGDRGDNNRVIKRPNSNYHTQLKCLTADSHFPERQGAT
>HelroP188210
VMNVLQPPLRSLDDRSNEAEYYIVYMQHKQQQLQSVSINNNNYKFNINININNNINNINYNINKTRCSINPSQFQHSRQNFVAFLEKVKQKD
YKNCVLSSRDAVHTIVNCSNPYRPQPKRFTMSFESISTIPNNPEFQAGSTYYFIG
>KFM67366
MYFTITFRSFTPQPGGLEFLPGHDYYFVSTSTGDLAGLHQRVGGRCATHNMKVIFKVCCSPSKGTSSSHQTSTVNVSVPASAAPNNLHKNT
SARPAENKSFDRSHLEPASVPPSTLTTAPRWVLPVPTSRMPPHYPRPYEVPIEDEMDKSPPSDKIKEAVEKMPKRDRAKNEELVNGAAPFA
HRWYHQSGAYYFVVACLLHHLLLGLLFR
>MESCA003657-PA
MFFTITFRPFTPQPGGLEFLPGNDYYFISTSSKDDLYRRIGGRCSTNNMKVVFKVCCAQEESNHLNYTTTITSPTKLEHDESLNKANRIETDKH
QHSSQSINTNISNVLRSTVSWSTPVISSTIPVRPNHYHPPFLSSPNPSYEQKEKTSVYPNKPTKKISEKNQPQ
>NV11883-PA
VTLVFLVCLQTVLLSTVSNCAKTISMYWNTTNSIFRIDNTDHIIDVNKNNAAFEYDQVNIICPVYQPGTYDDEAEKYIIYNVSKEEYETCRITN
QSPRVIAICDKPFKMMYFTITFRPFTPQPGGLEFHPGRDYYFISTSSKEDLHRRIGGRCTTHNMKVVFKVCCGNDADSSSYPATARNNSVAV
TSSTVSTSSSSTSTAVQNGNNAGMPAAIPPSVFKGSDRFYPSNVHHGHDNGHHQPGSGQDASPTLSHVPQVPMFPNYPHQQPQPPIYQ
GPPASQPPKTSITQKKKNKEYSDHPNEVVKNEELTYNGASSPTLRTLSQQQQLAWTLTLLCGVLLPYLLR
>OVOC4018
IMLLRFFFMPLVISGTIYNISWISTNPIFSESSNIPLFMEHLKMNDRIRFSCSTNGIEQTAIHRVNEHAAINCRGPARFVDSLVGTCMKANDSV
IIRLREIPMLPNEISYKPDHDYFFITTSTGEKNGFANSEGGLCLTKNMRLKIRVEAKWTKVNQKISEFTTKNGQVMMIPMNSGQYAVPVVYV
PVVSSSEVDEDDKKKLVFPYHLYFANGEFRASNSNLEDLRPRAELQFDANDQPENLENPAFVINSDGYAAAKPLKEKDADFEIEYMDSASD
RSHERFGIVDITIITNCIMIADILL
>OVOC4451
MDDLFTDRSLFPQDAEIGAEAAFIEVDAMDSMEIVCPTYSINDNVDKYEQLVVHQVSDLSFMSCELDSRSQAFLVCDSPLAATSISHKIVFR
QFSPLPNGFEYQPGRSYYLITTSNGSIEGMNNTRLGLCVTANMRLRIDVRPLSDHSYPSSEEGMNTRYELESSHEKEDWFRDHHVKPSTAD
NSKTEKDIDRLNNESQKFAEKHGLDLTRLEYVRQLAIDGVEGDFSFQELENDSLRNSMDSSKTTLASGPGRPRFSSTLSDHKAFYSTSRQAQ
RWISKEMQSRIDASRDLLPDQNTSGVDPAKDYVVDDISGAGAILGRLKTKYIIVNVLLLLWNSGR
>OVOC9135
INLLITVILDIFSNHRIVIDARRLPDIYWNSTNQIFDISNTDHVMSVKLLDRVTIICPQPTTINSRMPYEYTKLYAVSQIGYDNCQLLDERLIGVC
QTPGQQSSISIVFRDFSPLPGALEFKPGYAYYFITTSDGTEKGIDRRSGGLCASEHMKIKFEIQPDRGDNLNDYYEPSSAHFRHRLQKNELAEV
STPLLYIIHTSDSSAEGYSASEEEDGAACKCQLNMLLMVVTLIFLYFTVTL
>Ocbimv22026748m.p
MSLAQFCSIRVKIKVQQQQQLLLHKHAVFVNVYACCLFRPENQKLRVNLGDRVNIICPNYKSYTHESEMEYYSIYMVSKTEYEECVIYDPKN
AIRLLRCLKSNDTTLFTLHVREFQPFPFVPDFEAGKSYYIVTTSEGTYSGLDNQWGGACKHKGMRLKLDICCSSTTASPGGKSHTKSSRRHTT
VTPVSYPKQTTSTLRRVKMTTTPIISTTTTTTKLPISTTTVYRKTIHSYDTKIIEADFDKQSPGITKQNSEINATGPKNMGLINSSGFPRCSWTLL
FALIHVCQWVLLSR
>PHUM090120-PA
MALGFRPNVKSSTITGYSTTFIYSTSTSTSVLSVVLLVCLETVLLSTGSDCTKNFVVHWNTTNPMKVYIDLGDEEGKNNRKK
>PPA28315
MTYKPDNTYYFISTSTGSTEGMANEAGGLCTTHALKMKLHVVKRGGACHIHRKHIHHSHRSSSTTTTTQKATVVRDLPLESPFRLPSREERR
EEWILTPSIPVKKSREEDPLWDHFYQKVDLTPEREVRTRGERVALDTKASEYKLGDAYEALGQVLDGQNNV

>SMAR011358-PA
MYVTITFRSFTPQPGGLEFRPGQDYYFISTSSQDDLHGRHGGRCLSHHMRIIFKVCCDPSKVAPSPRTVSSSSTTALTTTTTRRRIITMPTTTR
STSTVAWTKTTTVLKNNGELQPEKERQGKKTDKKDKHPNETGVKNEELVNASRSIKKRCEIMIAVTSVNDTKCLINIQYNLCGPDTVGAQN
SVQSGQVSGRHRYFSMAGDWMGSAIVSGLDRCPVGTVSGPHRFGGFVGSEVDT
>TDAL010693-PA
FEFDQVHIICPVYEPGTYENETEKYIIYNVSKVEYETCRITNADPRVIAICDKPQKLMFFTITFRPFTPQPGATSSKDDLYRRIGGRCSTNNMKV
VFKVCCASEENNKTSTSFNHNPGLGGFNHSDHGNTLAGKIDVDLNSPTHINQPNVNNINNVHAGVGNIGNIPFKTISGPTATAINTNIDHF
NRIPMQPNGVNVISNNNGVGSLGNSGILLSPNHGTINMIPSSGNGVIPYQGQIGQTGIRINNIPPNHHPSIKNSNNNNDSRK
>XP_003394061.1
VTLVFFVCLQTILLSTVSNCAKTISMYWNTTNSIFRIDNTDHIIDVNKNNAAFEYDQVNIICPVYQPGTYDEDAEKYIIYNVSKEEYETCRITNP
NPRVIAVCNNPYKTMYFTITFRPFTPQPEGLEFLPGHDYYFISTSSKDDLHKRIGGRCTSNNMKVVFKVCCRNEADTSSSSATSRNNSVAVTS
STVPSSSSTSTAVLGGGAVGIPPPPPPSVVKNGGPDRFYPGGSIHHHHDHHQPATAAPTLPHVPPPAIYPVHPHQPPIHNGPPSSSPPKTSI
GQKKKNKEYSDHPNEVVKNEELTYNGASSSRVQDRYYQLVMVLTGSLFISAIMPQLLR
>XP_012055056.1
VTLVFLVCLQTVLLSTVSNCAKTISMYWNTTNSIFRIDNTDHIIDVNKNNAMFEYDQVNIICPVYPPDTYVDDDAEKYIIYNVSKEEYETCRIT
NPSPRVIAVCDKPRKTMYFTITFRPFTPQPGGLEFLPGHDYYFISTSSKDDLHRRIGGRCTSHNMKVVFKVCCSNEAETSASSATSRNNSVAV
TSSTVPSSSSTSTAVLGGSAGLPAPPIVYRGGDRFYPEISIDLDPHQPGTAAPTLPHVPSPAVYPVHPHQPQPPIHNGSPSSITPPKTSTGQKK
KNKEYSDHPNEVVKNEELTYNSASSYARTQVRYTSLILATGSLLMSALLQQLLR

Ephrins in Cnidarians

>Pphy_13023 P. physalis
LPSILWHPSNYLFGCSNLTIRVQVGDQINLVCPFVSSELRTFNTKSLIPTKATQEIQEKI
YFLTSEQDDKYRTCNSTGATLFHACDTPKVINIISETFYGKKVSPTTHQYLEGKTYYLIS
TSNRTKETIDNRIGGSCENQYGYFPLKLAIYVCTKQEVTSGSCNKCNTVSCYENGCGIWS
EWRPTNAFVWNGTKCMQKLARHCNSKYFTCEGESDKYEVTTNNSNCRFSCPTTSPLSSAI
LTSTSLRVPSSTLPSKSSMHESVITKTVYNTTVSIVPTTVVSKIPMTTVQISEKTVIVTV
TKSTCATDPCTAMSTMQPPTVLPIYSDINYDKKFYITVASAVITGLLTGVFVMHAFVSYN
EGKRIMTTIIQVEEYQPLSTENDTSKLENGSASNSQFNGYMEDVYTSIR*
>Pphy_19559 P. physalis
MNDNKWKVIFVIFIAWLGIVGSHSSYPSIIWHPMNPLFACPVPKINVRILDTVRFVFPSD
EYDFFMNSTNSKLLYENLYFLKTNRDQYEACNATGMQRLRNCGGTEKLDDCDIKNLLPSY
TVTFLESEISPEDFTFVKGETYYFIGTGFRTAENLKNLIGGSCNHVDNFGIYKLRLQIYV
CKDDDVDCHICKSEGCYYKECSMECPKSWTQMSYLDTSNQCKVLHIRYCTNRILGQTFPQ
INFTDILCGSIESGNGTKPRADKLQGSNPSNDGLVTGLAIIVVISFVVGIFIGVLGFKMV
NERQWLQKRKVNAMNFSDDPVRYYSQPSLQSNLPLEEKPPFIQNHSSS*
>Pphy_29075 P. physalis
MFVFLFDIFALSTLVTFFTYPVVVSSITSLILPSFFWDPRNQIFNKSGCKSSWKYPHLYI
DQESKMFIVCPFFRITHVIRNDYVPVSNLFENMYICNKEEFDTCKLGKDTDVTRLVMNCN
EPEVKLKYKVLTFSRLITSRDDFEFKSGETYYFIATSNGTKESLDNRSKGRCESHNMKLA
ITICPYGSNCHSKSNWTCEREDKDKNSDTKRTEKDVPYRPHVNTSITDNEKDVVRTDDTF
TGRQVSNKDNASFCEDKLCLVY
>Pphy_49294 P. physalis
KYHASLSDNSARGKIYENLYLLNTDENAFTSCDATSGKRLLTCDGTSPTSYDVKFEEFSS
SSDIPDFKQGETYYMIGTGFRTKDNIDNKVNGSCSKIDK
>Pphy_14332 P. physalis
AIQGIYEKIYLLRSDQLDNYGNCNSTGGKLYHVCDTPNVINILSETFFGRKVTPATHKYT
EGKTYYLLSSSNRTMENINNTLGGSCLGSDRYFPLKLAIYICTKEDVKMGKCNKCQTSTC
YENGCGQWNDWMASNIFVWNGTSCLQKFTRRCNSIYFECDGENEKYVDIADNSRCQFLCP
SPVFPPIK
>Aele_124411 A. elegans
MILLKHGINKSMIIFLAVCLAMVFARSSIYPSIYWNPWNPLFACEDPTINVRIGDTINFL
CPSDEYNFYIPFSHVNPVYENLYFLNDDEEKYKECNAIGARNLLRCDDKETSSKLYTISF

RDIQTSERSMKFDRGKNYYLIGTGFRKTGKLDNKVNGSCRDSGNPGQFKLRLKIHVCEND
DQCNICKSAGCYYEKCGANCSNWKSVSSETTECAVMEKRTCVYPYLNESARSEYRPRLNF
NRTKCEESRKVFVNKTKTEKGPFIAEQDGDIPIYKILVVVLGFNCLALLLFICIRSNYFK
GMSVKLRNMCFVIGGIGKKRNDDENHKLSIDDAEAHRDSRIDNNSIYQELLNGKINADID
MSKKIEKISDLCKDLLANASDEIEKSKTHLIIDLLHELQGKSIQNGDLKLEVDSGDSGID
DPDGRIRVGSDNLAYRNSTHSLPKKKSSMLSPPS*
>Aele_124437 A. elegans
MILLKHGINKSMIIFLAVCLAMVFARSSIYPSIYWNPWNPLFACEDPTINVRIGDTINFL
CPSDEYNFYIPFSHVNPVYENLYFLNDDEEKYKECNAIGARNLLRCDDKETSSKLYTISF
RDIQTSERSMKFDRGKNYYLIGTGFRKTGKLDNKVNGSCRDSGNPGQFKLRLKIHVCEND
DQCNICKSAGCYYEKCGANCSNWKSVSSETTECAVMEKRTCVYPYLNESARSEYRPRLNF
NRTKCEESRKVFVNKTKTEKGPFIAEQDGDIPIYKILVVVLGFNCLALLLFICIRSNYFK
GMSVKLRNMCFVIGGIGKKRNDDENHKLSIDGHCWDDEIERPRTEVVLDFFTDAEAHRDS
RIDNNSIYQELLNGKINADIDMSKKIEKISDLCKDLLANASDEIEKSKTHLIIDLLHELQ
GKSIQNGDLKLEVDSGDSGIDDPDGRIRVGSDNLAYRNSTHSLPKKKSSMLSPPS*
>Aele_79976 A. elegans
MSCKNIFILSLLWAGLTSGEYVQSIYPSIAWDPWNPLFACNEPKINVRIGDIINFLCPSD
ELKFFVSSSSIVVPHENLFFLGNDKDRYNACNATGMSITKKLLSCDDSQKVYTLSFRDIQ
TKPDSIAFTRGETYYMIGTGFRVKSNLTSTEGGSCRYTDSRGKYKLRLQIYVCKDDESDC
HICKSEGCYYKDCGSSCTPWISDGQVYRNGSKCFKLETRTCSDALIGSNQKENRYAADPC
SEYLKVTEDLSSKESVIAGLSIGVVIAFLVGIIFGFLIIKCYNKNHDKMVVEHKSGPVNT
ESTHINHGRFNKAFSFESLERFSNLVGVEKLENQKSPKQNEISPAEQQNPKSPKKRKRLK
ESEDEKNQNVFEKGESILI*
>Aele_151942 A. elegans
RLVMKCDNPLDTQKLKYKHFSFASTRSSMKEFEFQAGTTYYFIATSDGTQSGLKDLKDGR
CKTNNMRLAIHICEHGKNCHLTNRRCNKDGVKNPESLRSSNTAGSSAEKNLVLYIVLAGI
GGLLIGIILSAVIWTVRRTSQDGAFPSPYCCDKRSKLRLNEKVTQQPLC*
>Aele_151935 A. elegans
RLVMKCDNPLDTQKLKYKHFSFASTRSSMKEFEFQAGTTYYFIATSDGTQSGLKDLKDGR
CKTNNMRLAIHICEHGKNCHLTNRRCNKDGVKSKESSSIQTPKPSAREPVKIETTKPPTT
IRVVPSVSDPESLRSSNTAGSSAEKNLVLYIVLAGIGGLLIGIILSAVIWTVRRTSQDGA
FPSPYCCDKRSKLRLNEKVTQQPLC*
>Aele_151940 A. elegans
RLVMKCDNPLDTQKLKYKHFSFASTRSSMKEFEFQAGTTYYFIATSDGTQSGLKDLKDGR
CKTNNMRLAIHICEHGKNCHLTNRRCNKDGVKNPESLRSSNTAGSSAEKNLVLYIVLAGI
GGLLIGIILSAVIWTVRRTRSARRRNPNRDSGAPSTITEHIPVDDADEQKEDLDTFGNPV
KIETVNQKYFTSMDYRNSNEFRRLISTASTCSSNRYSDGERHCVTPIGGYK*
>Aele_151933 A. elegans
RLVMKCDNPLDTQKLKYKHFSFASTRSSMKEFEFQAGTTYYFIATSDGTQSGLKDLKDGR
CKTNNMRLAIHICEHGKNCHLTNRRCNKDGVKSKESSSIQTPKPSAREPVKIETTKPPTT
IRVVPSVSDPESLRSSNTAGSSAEKNLVLYIVLAGIGGLLIGIILSAVIWTVRRTRSARR
RNPNRDSGAPSTITEHIPVDDADEQKEDLDTFGNPVKIETVNQKYFTSMDYRNSNEFRRL
ISTASTCSSNRYSDGERHCVTPIGGYK*
>Aele_151938 A. elegans
MPSTLSSMPSMVTITLLLLYHQMGPFVVLSNKDIILPSFSWDPRNECFNSTLNGEGYAQI
RVDQDSQMYFICPSIAITYDVQTSYTPRKFMYENMYMCTKEEFDDCEIREINDKTRLVIK
CLYLSFCVSSGLSHFITN
>Aele_7590 A. elegans
VRMNDILNFICPYFDLSTQKSINTISLDFSNNHAIYESIYVTSEEGYQTCDAKNGTLFHK
CNTPSE
>Nbij_52832 N. bijuga
MASSLSSLPSMVTITLLLLYHQMGPFGVMSNEDLILPSFSWDPRNECFNSNQTAEGFAHI
RVDQDSQMYFLCPSSVITLDVQTSRPLRKNMYENMFMTTKDEFDSCEIKNINDKTRLVMK

CDEPLYKEKLKFKHFSFTSFRSSMKGYEFQAGTTYYFIATSDGSAKGLESRKAGRCNTDN
MRLAITICEHGKNCHRSNRRCTKDGGVKNPNALRSVKKADSSTENYAVYIALAAAGGLVI
GIFLSFMFLKLQKKASHFTSRNANRDSGAPSTVTEHLSVSESDDEKKVEQEDTFKCSVVE
VADNP
>Nbij_52843 N. bijuga
MASSLSSLPSMVTITLLLLYHQMGPFGVMSNEDLILPSFSWDPRNECFNSNQTAEGFAHI
RVDQDSQMYFLCPSSVITLDVQTSRPLRKNMYENMFMTTKDEFDSCEIKNINDKTRLVMK
CDEPLYKEKLKFKHFSFTSFRSSMKGYEFQAGTTYYFIATSDGSAKGLESRKAGRCNTDN
MRLAITICEHGKNCHRSNRRCTKDGGVKSKDSVIRQAVRASSAPKTVNTLLKTTNPPTTP
PHQPVVTSVSDPNALRSVKKADSSTENYAVYIALAAAGGLVIGIFLSFMFLKLQKKASHF
TSRNANRDSGAPSTVTEHLSVSESDDEKKVEQEDTFKCSVVEVADNP
>Nbij_143918 N. bijuga
MMTRRTRGLKQCLLAGVLLLLTGGNVFGATFPSIYWNPWNPLFSSEEPTINVRIGDTVNF
LCPSDGYHFYMPYSHVSSVYENLHFLNDNEEKYLTCNATGTRRLLRCDDKDSSLKIYTIS
FRDIQTSRDSIKFVRGKDYFLIGTGFRSTDRLDDQVNGSCLDSGDQEGQYKLRLRVHICA
DDDKCDVCQSDACYYEKNGAKCSGWKAHPLNDDCPAIEKRICTYPLISKFPRTEYRPRTK
FNKTKCEEMRNVIVDKNGTASSTKGTLIQNEEDYKLLFYIALPILGA
>Nbij_20083 N. bijuga
MFFNCILLTSFILVGITSGEDDHSTYPSIIWHPSNPVFACAEPKINVQIGDLVNLLCPTD
DFKFYSSIVYENLYFLGTNYEQYKTCNATGMKRLLTCDGKTHVHTLPLREIKTSIGAIAF
TKGETYYLIGTGFRVKENLGLTSNGSCRYTDKQGKYKLRLQLYVCKDSDTDCNICKSEAC
YYKDCGTSTCTPWLSDKQIYRKFSSTCYTYETRTCSNTLLSTSQSETRLVP
>Nbij_23770 N. bijuga
MELKTAFYWFVVISVADTLTTDGPKTLPSILWHPKNYLFACNENRNLTINVRLKDIIDFV
CPFLDEYETSIITESLPFLLEYYEHIYLVDEEGYVNCDTTNGIPFLICETPALLKIKM
>Nbij_57193 N. bijuga
FLKCETFALLKIKMEKFESIFKYNRMVNNTYNRKYEAGKDYYLITTSSGEKAGIGQKKGG
LCIPTADNYAMKLILHVCEDDSEACVCQHASCYEQQCGVWNKWRETQTFYWNGTTCLQKY
TRECNTTTFKCEGEWTKFVQAASKEECKLPCTTQRPVTPVTPNCTITPYSHVTKEKYNIT
VITAILTALITGLFVMHAFMCYYEGMRIEGRVYRESANYERLSSASCADSISKKDGGSIY
SNGTVQKNAADDVDYNDDRSRKITNATTEEDAGSFRERQQSHNGSQRSDKRSVRSEQQQQ
EERISNASDRVSESNTRRSFASDREQRQSNASCREPRGSQASRSISGHRSHANPAYDDNE
DIHNGRERSDKEEDAVRKESKKDFANYGQPIYTSIN*
>Clat_120953 C. lathetica
MVRSIYIIRDILLGSVNIAIVLAEFSTYPSIYWNPWNPIFACDDPSINVRIGDTINFLCP
SDEYKFYIPPSTDVNMIYENLYFLNDKKEKYEACNATDERRLLRCDDKQATAKYYTISFR
DVPTSRHSIEFERGKSYYLIGTGFRSLNQLDNLVNGSCRRSGSEEQFRLRLKINVCEKDD
CNICRSEGCYYEKCKSKRSEWVHWSDPVTSNGKCVKLLRRECTYPYLDNTNRTEYKEINL
NISKCVKELPSSAPRTEPDVSKAIALNEALNEELQKYKIIVYILLSLLMVMLIAFCSIRC
HLSKKVNNCIKRDIETCPANRGNREIVEIVEIVDADTKDGPGEPNLAYNESLLSKRGSKK
SKIKKRDEKNPDF*
>Clat_97078 C. lathetica
MFILNARRPTVIITWFLYWLTTSPPLVVTVILPSFSWDPRNECFKDSKDGASMHVDQESQ
LYIICPSVAITERVQTLITDQKNMYENMYMCTKDEFDKCEVGPERTAGKTRLVMTCDKPL
DPQKLTYQSFSFQQFQSGEHGYAFEAGQTYYFIATSNGTKAGLENRKNGRCRENNMKLAI
AICAQGENCHQKNKECIKDGSKEHSGAQKSSKGHDSAPSIKYMIIVGVGSFVVGVILCLI
VITIYRKACSKKKSPTHKTKNRDSNMTEHTTVGMEEGEEQQDDTISRAARKAEAETRESL
NQKYFSDERYRNSNEFKRLISTTSATSSNRYSDSERYSVTPMGVYAH*
>Clat_97081 C. lathetica
MFILNARRPTVIITWFLYWLTTSPPLVVTVILPSFSWDPRNECFKDSKDGASMHVDQESQ
LYIICPSVAITERVQTLITDQKNMYENMYMCTKDEFDKCEVGPERTAGKTRLVMTCDKPL
DPQKLTYQSFSFQQFQSGEHGYAFEAGQTYYFIATSNGTKAGLENRKNGRCRENNMKLAI
AICAQGENCHQKNKECIKDGSKERKIALTSLANITTERPTRVVPTLAKYIPQIDPQTVIL

DSGAQKSSKGHDSAPSIKYMIIVGVGSFVVGVILCLIVITIYRKACSKKKSPTHKTKNRD
SNMTEHTTVGMEEGEEQQDDTISRAARKAEAETRESLNQKYFSDERYRNSNEFKRLISTT
SATSSNRYSDSERYSVTPMGVYAH*
>Clat_48253 C. lathetica
MYVLREMLQIQLIYIGVFIISLACLASGNLQYPTIVWHPMNPLFACENAKINVRIGDQIN
LQCPTEQFNFFSPASVVQVLYENLFFVGNDLYRYDTCNATGTKKLLDCRDGKNSYFSIWF
QDTAISQDQLLFVKGDTYYLIGTGFRSLDNIDNTINGSCSTTDKLGKYKLKLQIYVCKDD
EECNICKSAGCYYKECAWQCRHWRNMRYDSGQGCKVMRIRNCTNEILGQSREETDFTDIS
CPTPPSTTSSATTAPTTTIGP
>Clat_80728 v
MYSKQIFLLSFMWLSLTNGESTYPSIVWNPWNPLFACDEPKIHVRIGDIVNFVCPSDEFT
YYVPSSSVTLPYENLYFLGSNKEQYEACNATGMTKLLNCNGIDPKHKTLTFRDSPIARNS
ILFAKGETYYFIGTGFRTKDNLENSVNGSCMITDKRGVYKLRLQVYVCTDDDAGCHICTT
AGCYYKDCGTTKCSNWTSDGIVYKNSSHCFKVETRSCTNILIGDNNNQTRYADAHCSSES
DICSDWVSTGNLYYDATSLQCFSMETRTCNSTVSGENYNENKYTNVSCPNILKTCPNVTT
RYLSSNKCDVLPSRYCHVTFGGKVKETNYTPCAAHISFLIEPSMTLYNTSAMVSSSKIES
AVPFINSTNITLNNTNSTVNVTSNKVWSGSSIKMTSARLEPEYGRQLGRDISEKDTIIHG
LAVAVALAFIIGVTFGALTVKYMNNKKCTVKVENKVQPGITTFSNSGFEGTYDNSGKDDV
*
>Clat_28862 C. lathetica
CHLFTRTQAIRKMFKLHFKVICLTNILLYYSALANLRYPSIVWHPMNPLFGCPEPKINAK
IDDIVNLQCPTEEFNFFEPSSHVGIIYENLYFLGTDKERYNTCNATGTQRLLDCRDGKNS
IYTMWFLDVPIREDSLTFTKGKTYYFIGTGFRTKEKLNHTVNGSCSKTDIRGKYRLKFQI
YICKDDDTKCNICKSSGCYFKDCFWQCGNWSYGPGSTTVETRQCMNDILGETKQEINFVG
QQRYSDTSETKGLVGALVGAMVLTFIVGIVLGIFCYKRRFIGTCFKQHSFNPKVQSTDP*
>Clat_22532 C. lathetica
MCHKTSKDMSVIIWKALVLYVAARLPLQVAGAAVNGDNRMVPMVLPYILWHPDNYLFACN
EHKNLTIRIINNDIINFVCPYLVKRPDKTINTKGYPSSQKKVKIYEKISRLDNFEDYRTC
NSSRATTFYTCDKPGKINIKQHKFINWGEDKWVEGRTYYLISTSERTINSLDQKVNGS
>Atet_66572 A. tetragona
HIVTDIKLKNKRKKTMEMFGFICNVLRRRKSGVWCATWFLYWFAVFTLPTSVFNVILPSF
SWDSRNECFSSATSSGVELSVDQESQLYILCPSVAITDHVQSVVADQQQMYENMYLVTKE
EFESCTLDDPPRNDGRTRLVMECNNPLDTQRLKYQSFSFQQFQSGEFGYAFEAGKTYYFI
ATSNGTKIGLSNRSGGRCRKNNMKMSIHICAQGKGCHLQNKPCSQQKKESESKSTSVMGS
LELQPTAITNPTSKQTSPPTTSAPCNNIINNNNNSSNNNNNN
>Atet_141600 A. tetragona
MCNQLAVWCVALATWGVTLVDLTSTTSTHPSIVWNPWNPMFACDEPKINVRIGDIVNFVC
PSDELTFYVPSTSITLVYENLYFLGTRRDLYNACDATGMTQLLRCNGDQPKHTTVTFRDF
AMNKRSITFERGKTYYFIGTGFRTKENLDRRTNGSCKLTDEQGKYRLRLQVHVCADDDAG
CHICKTTACYYKNCGRITCSSWFSIGPNSQNITNCFQIQTRTCKNDLTEHEWHETTHANV
LCSYSNNTSTNCTQWSSTGNLYYDTNAMQCYAVETRGCNNLSTSDKISYSENRYKGVTCP
QVLRLCSNVTSTFLLQGECNPSGSQYCRLVLGGAIPSRTYTPCRLSGKVQAVQPTKSVTG
SLGTSSHYGPVSFIDVMSESSIYASPSVVSVTSSRGCTGSALMPDTFIDNEMEYQRSYGD
GKDSSLKDRLILGLAVSVALAFIIGVMLGSFTVKYLENRHRSAKVQDKEIVVPGTVATFT
NTGYDDTQGSFDNLGSEV*
>Atet_41310 A. tetragona
MSGGGGGRVLWCCSWLLCWFSIFNSVFCLILPSFSWDPRNLCFSESVDGAHLSVDQESQL
YILCPTVAIIEHVEENISDQKNMYENMFLCTKEEFDRCQLDEERGDGRTRLVISCDDPLD
TGKLTYQSFRFQQFQSGEHGYAFEVGRTYYFIATSNGTKEGLFNREGGRCRNDNMKLSIS
ICAQGEQCHLRNKPCTEPSVEAGNDKVVAAAAASIDFEMSMETTPQKPTAFVHTTAAAMN
TTMVPTIHSPCSPQTPTFRHPSVVAAPQVSQAPYVHEKSPISSSSSKKTMVLVGVGAFVG
GIVFSLLSITVYNKSCNGGSSSCCFRSRKSVSSNGVTQLMA*
>Atet_88598 A. tetragona

MDVLFCLFILTSHSVVMAEFSTYPSIYWNPWNPIFACEDPTINVRIGDTINFLCPSDEYK
FYVPPSTDVNIIHENLWFLGDKDQENYAKCDARGARQLLRCDGQKANYYTISFRDVVTSA
RAIEFEKGKTYYLIGTGYRTLDKLDDDV
>Atet_192642 A. tetragona
ICLILLIYFPTYLLPTLLADEMKNVQLPTVVWHPMNPVFSCNGNTMMRVRIGDVLNIQCP
TSQLKFYSFEENILQTYNENLFFLGNDTVRYNTCNATGIQPEQKLLTCKNDQNTAFTIQ
>Atet_40821 A. tetragona
LDVERLPKALTFVKGKTYYFIGTGFRTKENLNQTVNGSCSRTDEAGKYKLKFQVYICKDD
EDCDVCKSTGCYYKHCYCQWSAWSNSGAFKRTRTCRDEIVGEMHQDQKVDGGRISLALVF
VAAGTIVLYLIIGFICRKYNIPSQIQEAAERTKLNCKESV*
>Atet_88599 A. tetragona
TSARAIEFEKGKTYYLIGTGYRTLDKLDDDVGGSCNHTGSDGQFRLRLKIRVCENDKDCD
ICKTAGCYYKNCRVSCSPWVTHGYIVGEKGDCLKMEKRQCHYPYFDNNNRTEYRETKHNA
SKCSPGVGRQTGDKRPLCDNSSEIYKVLFVISVAILFVVLLILLCYKRCCIGEKENTYDA
EYPKDVQALNGCNNPVGKTNPGYRNSTKMSIHLPKMPSFRNNSNKSPTAKHPPQHPL*
>Holi_5987 H. oligactis
LKKMLIKITLLLWMQAVLIDTLQLPTFLWDPRNKIFSGDLCEGEYAKISVNYEATIYFTC
PHEALCSDVIEGELNNVKSMYENMYLVSKEEFENCEVNDKRQPILLCDNPEDNSLIKFTS
FYIFDVKSSLRTQFKDNTTYYFVATSNGKKNGLRNLKGGRCKESNMRLAMYVRGQQDNSP
INEHMCYEPKKVINLVKTDSPITTTESLMESLQYKTESSYKNTEFSDLSTEPINTKTEAI
PRVLPLKDSKNKQERDYDTKSHLWLDAIYVFVGFVVGIFLTLFIIKVKKYWMEKSFSQQK
AVCPEVFKEKDLKQQIETTNPDNREIQSQLLFNDNQAYRHSTEGNPLIEKLPCSNENNCV
ILMVK*
>Holi_3973 H. oligactis
MSKKDRIKVMQIVLILGCWSYLVHADSVILPTIYWDPINPLFACETTIQVEYDDVLTFKC
RDSSTPYSQSIDDTDILYESAYFLGTDQYMYNTCNATGAKEKILNCNIGTDSNSRTITFS
KYATSGKLKFTEGETYYIIGTGFRVQPNIGKSDKWKL*
>Holi_14653 H. oligactis
MKFDTLITLLIWRSYFHLKTYAAVPKNAVLPSLMWDPRNYLFSCENLTLKVRLGDKINLI
CPKAELNTLLLEQAPNAKDLFEKIYLLNEDQHVNYETCNTSSLLNFNWFCNKEFSHLLVF
EDSPLDPKTKPKFE
>Holi_7726 H. oligactis
FLGTNERMFNECNAAGSKQLLYCSYKNDSKSTSKLFFKKDSGDPDIITFEAGKTYYIIGT
GFRDAKNLDQLINGSCNTVEGGGIKRYRLRLKIYICTYDEIKLNKCGECTYAGCYFNNCE
NCTEWITDRNIEEKANNSSLCFSLESQICINPFLSSKVETRYRLVNTSCSTPTATPLIES
TSFSSLHPNVTSIFTEPSAPTAARQASLTAKSGGGTTYIVVASLVALVIGIGIGALGFSK
IHKKNCVAAESFNKICYEKKLAVPNSDHCFVNCNVIAK*
>Hvul_1033087.1 H. vulgaris
KNLKFLKMIIKITLLLWVQAVLIDALQLPTFLWDPRNKIFAGDLCEGKYAKISVNYEATI
YFTCPHEALCSDVIEGDSNNAKSMYENMYIVSKEEFENCDVKEXRQPVLLCDSPEDTSVI
KFTSFYIFDVKSSLRIPFKDNTTYYFVATSDGKKNGITKLKGGRCKENNMRLAMYVREQQ
DNSPINEHMCYEPKKVVSLLKTDSPITTTASVLESFQLRPEAFLKTTQFSSPNTEPSGVK
TEFSIENTYKKPVHEQIKQNPSTDSKSHFWYDVLYGGIGFIVGIFITIFIIKVKKSWTEK
SLSQTKAVCPEIYKEKDNKTEIINPNNQTQLLFSDNQAYRHSTEGNPLIEKLPFSNENDC
VILVSK*
>Hvul_1028689.1 H. vulgaris
LITVLIWRCYFHSETFGAILPSLLWDPRNYLFSCENLTLKVRIDDRINLICPKAELNTLQ
LEQAPNVVDLFEKVYLLNEDQFLNYETCNTSFLPSSNFAWLCDKKEFLSHLIVFDDNPLD
PKSKPKFVAGSSYYLISTSEQTESKLNQNIGGSCRGNPLKKLFTLKLKIHVCKKNEEKKL
CEVCQTVSCYEKGCGKWLPSSDLTISNHIWNGSHCLRKLPRKCISPYFQCDGLPYSYEVE
NNKEACKLECESSNTSSSIAYDKSTITSSSTLLYSVSSCSVTTFTEFITSLVISTAYISS
VSYSTIKTTVQVTVTKTETSPCSQSATVTQPARAEKVYVDIEYDKKFYITIIATIVGAIF
VGIFVAHACVTYFTSKRAKKQNIYYINEKDELVDTEETKIRRVSVT*

>Hvul_1018918.1 H. vulgaris
MTNLFNIQKDCVRNMQIALILGCWSLLVHAESVILPTIYWDPINPLFACENTIQVEYDDV
LNFKCRDSSIPYSQSIDDTDILYENAYFLGTNQYMYDTCNATDASEQILNCNGGTNSIDS
YRMVFSKYSPSGKSTFKDGETYYIIGTGFRFKPNLANLINGSCNYVYKSGQYKLRLKIYV
CSPEETCKKCNSDACYFKDCQVDCTSWETNYLQLIKSNNSCVSLQTRNCTNQLIGTVSIE
KREIPITCPTLSTFTVPTLSTSNVPNLSTSTVSTPSTSTVTQSLSSRAASLINQNNSQMK
LTYIMIFTIVFVFLFGISLGIAFHKKLYNRKFDTSPSNWILGNGIQNHDHCNGVNGDKIN
*
>Hvul_1028415.1 H. vulgaris
MTNLFNIQKDCVRNMQIALILGCWSLLVHAESVILPTIYWDPINPLFACENTIQVEYDDV
LNFKCRDSSIPYSQSIDDTDILYENAYFLGTNQYMYDTCNATDASEQILNCNGGTNSIDS
YRMVFSKYSPSGKSTFKDGETYYIIGTGFRFKPNLANLINGSCNYVYKSGQYKLRLKIYV
CSPEETCKKCNSDACYFKDCQVDCTSWETNYLQLIKSNNSCVSLQTRNCTNQLIGTVSIE
KREIPITCPTLSTFTVPTLSTSNVPNLSTSTVSTPSTSTVTQSLSSRDFNXSCTLWQTDY
LKIITSSDKNCSYLRTRRCKNQENTRVELEEIPTICPNTTSFPIVAXVVGENNNKQTLTY
IAIFVSILAFLFGIFFGVFIXKCFILKXECXDYQTKDCGMQNSAYTGSTDFEKGNEINYI
EKINNSHV
>Hvul_1038805.1 H. vulgaris
MKLQFILLVHMKHFVVMLLKGTQTMRNLCTRICILFLKKNLKTVNVKENRQPVLLCDSPE
DTSVIKFTSFYIFDVKSSLRIPFKDNTTYYFVATSDGKKKGITNLKGGRCKENNMRLAMY
VREQQDNSPINEHMCYEPKKVVSLLKTDSPITTTASVLESFQLRPEAFLKTTQFSSPNTE
PSGVKTEFSIENTYKKPVHEQIKQNPSTDSKSHFWYDVLYGGIGFIVGIFITIFIIKVKK
SWTEKSLSQTKAVCPEIYKEK
>Hvul_1002089.1 H. vulgaris
MLFNCQLFFGILEIKCSFAGDLCEGKYAKISVNYEATIYFTCPHEALCSDVIEGDSNNAK
SMYENMYIVSKEEFENCDVKENRQPVLLCDSPEDTSVIKFTSFYIFDVKSSLRIPFKDNT
TY
>Hvul_1034474.1 H. vulgaris
MWYIYYALVSLTTVNSIVLPTIYWDPRNPIFSCEVTIRARLYDTLTLACPTGDLPYQNLL
LDNNQIKENAYFLGTNERKFNECDATGSNRLLYCSYTKDKKPTYIFFLRSILVILQYYFF
TPGKLTILLVLGFEMQKILIS*
>Hvul_1029169.1 H. vulgaris
IKIKIKKKMVSLWFKLYFLILFLFGSNNGFTLSSILWDPSNPIFNAAKCSKDSTKFCAIM
YDEINLLCGNQYLNTYPIAYMSKNFFYNVYSTTNITEFENRDATNLEPLHKCHPKWDVKE
EKLYFDHFKYIFKQNVGQTVYFFTTSDGTKKSLDYTVGLTETKHMQFSVSLRSEENLECR
HRVNLCLQDNSGNPTAIPLRSEQENTFAHSVKHPMVLLLLFGLLVFGVVIGMVASIYFTQ
FFRKKKGSVSTSSSGDDYCANTSNLLSKPAGQKC*
>Hvul_1019657.1 H. vulgaris
MLTFLVLMSESSMNAMQQAQIDCFIVVIQKMKNQPIYFFLRKYSGDSAVLSFEPGKTHYI
IGTGFRDAKNLDQLINGSCNTVEGGDVKRYHLRLKIYICTNEEIISNKCGECRYAGCYFK
NCENCSEWKIDRNVEQKTNTSRLCFLLESQTCSNPFLSSNDERRYRLVNVSCSTPTPTTT
PLIESTSVSSLHYNTTSVFTEPTVVAVQKPPAENAGTTTYVVLVVSSLVALVIGIGIGAL
GFKKI
>Hvir_3164 H. viridissima
TRGRKKHILHFSHCVLSSLIFQTGRKKIFKKMQLKVTFLLWVQAVLADALQLPTFIWDPR
NKIFHGDVCAGQYAKISVNYEATIYFTCPHEALSNDILAGDFTKKKSMYENMFLVSKEEF
DNCEIEEKSKEKRQPVLKCDTPEDTSQIKFVSFYIFDVKSSLRYQFKVNTTYYFIATSNG
EKSGLENLKGGRCKENNMKLAMHVRAQNDDSPRNDHMCFEPKKVSNIETESPTTTRDATN
IYYTSESSTLSGEQMIQPPVAAASTNSNGKTANSNSINSNSIHSNSINSNSMNNKNLKET
IFLLSIGFFSGIIVMTVFYLAVKKWNERCSKRNDVKKMVSNPNQEIQNQLFIDNLAYRRS
TEVEHPLIERSALSTEKNPNIHLNV*
>Pper_105129 P. periphylla
MFQLLLFYFAIIGGKLGVGLHLPTIVWDPRNPMFACDDPQISVKLLDAVNFLCPNKGVIE

GGIKNPAKTENLYENAFILKPDQETQFHECNATGATQILDCNTPNTLKYNTLSFQPVSAS
SFTFQKGKTYYVIGTGYRTVSQLASRINGSCSKTDPSDGPFKLRLKLRVCSDDEVNLYKN
CSICESSGCYSKGCLSCGNWVQPADEEPYRKGSNCYTFHQRNCSNHELSVYYSEQKEVGV
TCPTTQTLTTKELTTKLQTITTIAKTLQSTISSTKRQSTMFTVSTSEVTTTKTKAPPKSS
TLKTSDDKYSTSFYFVAACVCVIFFIIGNVSGYFIMRWWERKKKKV
>Pper_103076 P. periphylla
MQQGNKNIVLPLALLLLARYSVTSLLPAVIWDPRNPLFHCPSKANQEINVRIHDSLNLVC
PHKSINIGGQENFVKTEDLYETAYLLQPNQENDFNNCISSGGRELLKCKTPNTFSYTTLT
FKPISGSGDATFVPGKTYYLIGTGNHTVRTIDNKVGGSCRGTDGTYKLKLKIKVCLDQDS
SCGICSTTACYTKGCGNYTSWNNTGLYRNLSTIASDSVNGHLKPLSQSASDNNCVQLRTR
SCDNDDDGCIGSNIDLVDTTCPKVNVSADIKDCGTWNRVGDTFRNSTDGCFVIKSRECWI
GTIEKVNTTEWKVEQTICPTVPVPPTKKPVACEDWTKSADIYYNASNYCFTNYIRECRLT
RDAFNYTQRELRRSICPTTLPITTQSTTMKTTPATKTLVCSDWYNSNDTYWNSSHACFSK
QVRNCTVAGNMTQVQLSDIACPTTPSTSAPPSTTLTTSNTSPTSKVHTTKKKGVGGGFTE
WSKWTMCVDFGCERLNKTRNRTCTNPPPSNGGLNCVGNLTESNKCPPKCYKKPIVKQDTP
VNSELSGNDNAVKCKVAMPIVAFFIGLLIGGFAVFSYIHFQEKRKK
>Pper_55548 P. periphylla
MAMVELEASYSGLNRCQLLGLILLFEFKMLSAFILPPFFWSPRNPYFHENKCGDHAHWYV
NSGYYLNVICPSAALPPKHLPDHIPRYKLFENMFIVSKEEFDSCTLDQKKHPRSRALMYC
DKSQTEDLQYRTLHFKPVRAWSGGLAFPVNKTYYFIGTGNGTYRGVKNTAGGRCKTDNMK
LAITVCGWDCSKCHDFCCPNSKNLTTTVATTIAKRTTIATSKPSPSTKENITTATQQLTQ
KVTEQNEGMLKTKKKK
>Pper_103077 P. periphylla
SDQENQFNNCNSSGGRELLKCQTPNVFSYTTLTFKLISGSGDATFVPGKTYYLIGTGNHT
VRTIDNKVGGSCRGTDGTYKLKLKIKVCLDQDSSCGICSTTACYTKGCGNYTSWNDTGLY
RNLSTIVSDSADGDLNLKPLSQSASENNCVQLRSRSCDNEDDGCIGSGVSLFNTTCPNVK
VLPDIKDCSTWQSVGDIYRNSTNGCFLIKSRECWIGT
>Pper_140147 P. periphylla
GDTIHLVCPNKATNLVVMDNVPSSYTLYENMHITGNKTTYLNCNATDTDVKLLEKCSQPN
TLQYKTLTFRSM
>Pper_41140 P. periphylla
PFGRDIQNIIISLVDLTNNSTDIGNMFGLLLFCLVGMGGKLGFCYVLPTVVWDPRNPMFS
CDDPQIVVKLLDSVNFVCPNKAMNEGGVKDPAKTENLYENAFILK
>Aaur_20911 A. aurita
FLPNNTYYFINTSNGTKASLKNKEGGRCKTHNMKLKVTVCDPAQGCSKCKDMCCEKPPTT
RPPTTTPTTTPTTVTTTKITTTEQLTVPAINDGNGGSGEETMVRTKEDTNATTITSRPRT
TQIHTTIETVLASTQKTKIESNKIS
>Hdig_22696 H. digitata
MAFRLNLKLLVVALWIFMECTLRSVSEIYVPLHWTPLNPFFKSSTSSASTSVLKMNVLPK
SQLNIICPNTVLNPVKMHQSPDKNNMYENLWIVDKMSFDSCSVNTSIKANRLLLQCNTPL
KFKHYPMVFFEISPSVQFLEFQKGKDYYFISTSDGTKKSLKNTRKGHCKTAKMKFIIHVC
LNEQDPLCKDKPATTTTIKPTTIPTTVPLHN
>Hdig_4952 H. digitata
MAVAMSLQSYEIFLLFFLLTWMLASSDIYKSVMWTTNNPYFQNSNTSELKSECFGGTLQM
KVRLKSVLPIVCPFEFIIMQKLPSRPSTGVLYENIWIVYDKESFDTCNVNLTARWGTPQE
IIDGEALQNKKLYDCNTPDTLQHYMYSIDFKEKSPVMPGVYFMATKTYYFLATSDGTPGS
INKTSGGHCNNDGDNKIRMKFQVYVCGTENDPKCNNSAEIGNFTCLRPDKPSPTSTISPS
ISTSQATVGPSPNPPGERTSFDELRKSRDCWRIAALFFVILFLILLLMFIYICYKLKKQQ
AVPVSPIDEPPYKENDKKDLIIPNDFSQTNGGYNEVDGSQTPKPEKTYTDPSAQPSPDEG
VEMTPVDIGVPQGKAVQDKPNIHAQTHVNIIGITQSNGNNNASLINKNNADQVHVRNGEV
GNKKNKDLTPKKKREAKDNLPPLKIPPRPVTGSPRTESSLISTGNNQEIEFHL*
>Hdig_30557 H. digitata
IFQKPEKTRVINVISMSRINIICPNPAMNVDVVQDNTPKEWLYENLWIVSKNSYDHCNAS

SPKTDRLLLRCDTPLQLKYHTLIFLQYSVTTQGLEFPVGKEYYFIATSDGTKASLDDRAG
GNCQRGSMKMKIYVCKGSGDPRCMPVPTPQKPQSNTDNA
>Hdig_5881 H. digitata
MNGNMRLNSFSSLLTILMSIVWTGQCDLYTSIQWDPLNPIFKASQEHDKTTAVLQLNVRM
SSKMNIICPNDATNLVKLKDTPRKDSLYENLWIVNKKAFDSCKVNSSIPGNRLLFSCDNP
DKLKYRLVRFVDASPVSGDPEFKFGMHYYFIFNIKWLREFPE*
>Hdig_22213 H. digitata
MKTAKTTRTSWVIFFKSRWILFIVTILTWSPVTSEYLESIVWSSLNPKFHFSKDDFNNNK
AFFTKPVSPQSNLVLVCPNVALILASISKQPTDLYENIWMVNRDDFYTCTINTLSRDNRL
LHLCNTPDKLNYYNVNITKVYSTLDYKPKEEESRSIYLIATSNGSKESLNQTSGGHCINK
KHGISLRIKITICTSKSSDPSCQIPLSSTSNVTSTCHNKTLQVTTSKI
>Apal_398190 A. pallida
MFWTETIIMVPSMLTGWLILEVILLVKPSVSLVYPSVQWTPRNPIFQKPENSRVINVQSM
SRINIVCPNTATNVDIVQDNTPKEWLYENLWIVSKNSYDNCNASSPKTDRLLLRCDTPLK
LKYYTIFFLQYSATIQGLEFPVGKEYYFIATSDGTKASLGNSAGGNCARGSMKIKFYVCK
GPRDPRCQPPTTSKKPQSNNIITYLPTTAKQPSKHFKTKQKPVETNSIPVKVVPSPTTQK
YSTTTTTKNEALQSDDPRMTLNPTLSSQGSTTVVLPETTTVDPEASIEHLLNRELNKEPC
THDHGTQPSWNVVIPLLVVIFILVIMNSFVICRLKQNNKSCLAEDNLNNVSTSEKRYSFK
NNNRKASSPSSSPVMV*
>Apal_400465 A. pallida
MDDIHFNKLSSFLISTILSTLLWTAEGTLYKSIHWDPLNPIFKTTNKSALQFNVTMKSQM
NIICPNVPTNLIDRDDAIQKDNLYENLWFVDKHSFESCHVNTTNKHNLLLYKCDDPIKLK
YRTLTFKAISPIKGEPEYEKGKEYYFLATSNGSKVSIDNQRSGHCKTHNMRFKIYVCSGK
NDQRCKTHESDEAKTTLSTPTQVINDKENFEGEKSGKRAACFLKSGDKIWFITIGVIIGI
LLMLLVGCLAFTWIKRRRSSYGDQIDGGKMPLNGKVGSNRV*
>Apal_41158 A. pallida
MWNTLLKFALLTSLLEPTLNTVIYPHLQWDPRNPFFKGSRSLNVETMSRMNIVCPNPALV
PAKLQNTIPKEQLYENFWIVDKSSYDACAVDTSKKTNRLVMECNKPLEINYYTIVFRQFS
ASANGLEFTPGKEYYFIATSDGKEVSLTKTSGGHCASHNMKMSVYVCAGSSDAKCQEESA
SPSTAAPFYCPTTTESTTTAPTTVAPTNPQCADSSTLLSIYNNTKATPMISNQTSLIPGL
VTALQRLEEKLVTMETKLDNCTSKSSSCSEPATSAPTTTSAPTTTTAPTTTSAPTTPPIP
ESCSAAYAAGSRTNGVYKINPGGLGAFDVYCDQTTDGGGWT
>Apal_344094 A. pallida
MASIRFDLKLLGLVLWVILECTLRSLSEIYVPLHWTHLNPLFKTSKSSSSPSILRQNVLP
KSRLNIICPNAALNPVKMHQSPAKDHLYENLWVVDKQSFDTCRVNTSLKANRLLLQCDTP
LIFKHYPLVFLEFSPAKQFMEFQKGKDYYFISTSDGTKNSLKSTKGGHCKTASMKFIIHV
CLNDQDPLCQDKPPTTTAPPTTQPTTTTTTKPTTKSTRIPTTQEPTTTVSPTTTPLTEQT
IRTLKQYEITPETMQNADVTKRDQQTNAGTEKTVNKSPDMETAKGERSAADIARDATWFI
LMAVMGALLVVSISCNLYTICIRNKAQRRSETVDDKRSSAVHTLLRDNSRVDKV*
>Apal_288439 A. pallida
MANLLLQISFLLTPCTLVSSDIYKSVMWTTNNPYFQNHNKTGNSLKSFCYGGELEMSVKL
KDVLPIICPFENLIMQTMESQPSSGVLYENLWIVYDFQSYNSCHINSNATFGGGSKKLKN
KLLYKCDSPDSLSNYFYRIDFKEESPVSKDFYFEPNTTYYFLATSDGGKSSLDKTSGGHC
NNDGDNEIRMKFRVYVCGSNEHYKDPKCADSKTVGTFRCIRPESPTSPPTTSSSVLSTSI
SPSPTISTTVVTSSDKKCQKETNDTPTKATDPSVSLECRIGEDPDLVKSRDYWRIAAILF
VLLFIAAVVAIVIICYRLKKKYSVPCIYSKQISVAESCHSQNLSPQNTVDKKGLVNKSYD
NMDEIDGPKKTMNKENTLQTPAVEMKKQPPLSNGNLHNGSVNTDKHDGGIETNGIQRKDT
GENKTFQMKTPSKLPPIPTKQTDVEFQV*
>Apal_288406 A. pallida
MANLLLQISFLLTPCTLVSSDIYKSVMWTTNNPYFQNHNKTGNSLKSFCYGGELEMSVKL
KDVLPIICPFENLIMQTMESQPSSGVLYENLWIVYDFQSYNSCHINSNATFGGGSKKLKN
KLLYKCDSPDSLSNYFYRIDFKEESPVSKDFYFEPNTTYYFLATSDGGKSSLDKTSGGHC
NNDGDNEIRMKFRVYVCGSNEHYDDPKCADSKTVGTFRCIRPESPPTTSSSVLSTSISPS

PTISTTVVTSSDKSELSVCSCPSMTYRWVTSTIVVNVTWNGSLTAITPLATKTRNNTVVL
ISSIAPNTTINASITSSHNLPGNTSNVNPSCECQKETNDTPTKATDPSVSLECRIGEDPD
LVKSRDYWRIAAILFVLLFIAAVVAIVIICYRLKKKYSVPCIYSKQISVAESCHSQNLSP
QNTVDKKGLVNKSYDNMDEIDGPKKTMNKENTLQTPAVEMKKQPPLSNGNLHNGSVNTDK
HDGGIETNGIQRKDTGENKTFQMKTPSKLPPIPTKQTDVEFQV*
>Apal_54304 A. pallida
MDFVYIVIFLAFCRVANSTIYRSIIWSPENPVFKETKLRCVLPRSMQYFLCPSLSLIVTD
REQQPKKENLYENIWLVDQHSYKTCKVGNTSKLLKKCSTPLTLSRFDILFLDMHPLSGGL
EFKFGKSYYIIATSNGTQKSLENKEGGHCRSHSMKMQFYVCKDDQDQYCRSEENCRHLLD
TPSTKRPTERTVEYSSTVTTKSCNLTHAQVKPTTKETETPDTSHYVASIEVFYRLGLAIL
GLVCVVFIASTLYLAIRLYKFTKLHSDCKKYTAQLTRPCNGSTAQSPNDSEVVLITCTTP
ENT*
>Apal_89985 A. pallida
MGFLRHYFQNLKGSPRTVFIKDHINFICPNNVIYVDKSNTDLDKDMLYANIYMVDKEGYD
NCNATGGKKIEVCDDPLKVKHAKVTIFTIDNLPNSQLPFFKAGHLHYFIDTSDGSQKNVS
NLIGGKCRTENMKMKMYVCKDKSDKACPYGNDVTNGGWSSWGQCSQSGTQTRQCNNPTPY
NGGFGCQGDDKRPCLPSTTAATTTTTTEPNHTNNDTITPKKEKRTGGVADTLGTLSVGEA
VAIFLAVFVFGVLFGVLLGFLLARTTFAHRHLNNVLRRKTSNSADQFNRNSIVSTTKLRP
FSTVSNDTTLTGVTGFTSYNDIYDASTS*
>Apal_344111 A. pallida
MNVLTLHPKPCFSLVFISLWCRVLGEYHESIYWSPLNNIFHFSKNDFKNNKAFHSKFVSR
HSNIIIICPNIATVLSKRKDKQPADNLYENIWMVNEKDFYSCKVDTAVKENRLLHRCSQP
GKLSFYNINFANVHYSKHANFSSIYLLATSNGTKGSLNNTSEGHCKNEEEGISLRIKFSV
CDASLPNNSCKESLTSSTNKSKGSLTCHNDTVSTCTSRTTVESIHHWEVLAICLGVVLGL
SLVINIIAVLYVCVWRKRAQGPSKTKAEEPMKSLIVEPHKSKHGRVSAAL*
>Apal_288431 A. pallida
MANLLLQISFLLTPCTLVSSDIYKSVMWTTNNPYFQNHNKKDNSLQSFCYGGELEMSVRL
KDVLPIICPFENLIMQTMESQPSSGVLYENLWIVYDEESYKTCNVNLTATFGGGREKLKN
KLLYKCDTPDKLTNYFYRIDFKEDSPVSKYFYFEPNTTYFFLGKLVIMPLDNELQVLPFQ
RRISI*
>Apal_8979 A. pallida
SLHKVCRLYFFFTPWTLVSSAIYKSVMWTTNNPYFQNHNKSDNNLKSFCYGGELEMSVKL
KDVLPIICPFEILIMQTMESQPSSGVLYENLWIVY
>Btue_29916 B. tuediae
IYVPLNWNPLNPKFQPRTVNSSYYRMNVLPMSKLNIICPNNVLNTVKLQRSPAKDHLYEN
FWIVDKKSFDSCEVNTSIPSNRLLYTCDEPLKLKYYLLVFLDFSPSIHSLEFVKGKDYYF
ISTSDGTKSTLKSTKDGHCKTHGMKMVLHVCLNEQDPLCKAKPVTTPRPTTTYP
>Btue_9751 B. tuediae
MSKLEYETVFVFLAVLSGLVTPDIYKSVMWTTNNPLFLNSTGLSRCYGEDLEMRVHLKSS
LPIVCPFEFIIMQTLPSRPATGVLYENFWIVYDRESFENCDINMNYTSPDNEKNKKLHDC
DTPDTLKSFTYTIDFKIRSPVNTGTAFVGGRYYYFLATSDGTDGSLNNTKGGHCNDTNIR
MKFKVYICKENDEKCQNISIGELLCPSDFISKPSTSSCPSPSLCPSPSLCPSPSLCPSPS
LCPSPSLCPSPSLCPSPSLNPSPSL
>Btue_9750 B. tuediae
MSKLEYETVFVFLAVLSGLVTPDIYKSVMWTTNNPLFLNSTGLSRCYGEDLEMRVHLKSS
LPIVCPFEFIIMQTLPSRPATGVLYENFWIVYDRESFENCDINMNYTSPDNEKNKKLHDC
DTPDTLKSFTYTIDFKIRSPVNTGTAFVGGRYYYFLATSDGTDGSLNNTKSGHCNDTNIR
MKFKVYICKENDVMCENIPIGEFLCPNDIAALSTTHPPTSQPSTTATTGNIISTTGNIIS
TTDTKLPTTTTSTESLPTTTSAASPSTTDAGKTSNGANNPSQRKSNEILDELSMWKTAAV
VLFACVCGLLLLIGAYCYIKRHQEDQSKKNKVTPPVKSPASCDIEILQMNFKLSCNNSSY
CEDSEDSEDGKKSHD
>Btue_23164 B. tuediae
MKQNSVVGFLFLTILMDIVLLGDCKTYKPLQWSPLNPLFDTSVYRINVSMYSRLNIICPN

PANNPVTIELDLLKERMFENIWSVNKESFETCVVNTKNKKNNLFFKCIHPLKLMFHTMIF
LPTTNIKGQTEFEAGKEHYFIATSNGSQNSLENTAMGHCHTKNMRFIIYVCEHKNDPLCI
KNGPDGPDDEDDKLERPSTETTHP
>Btue_30708 B. tuediae
MNVLPMSKLNIICPNNVLNTVKLQRSPAKDHLYENFWIVDKKSFDSCEVNTSIPSNRLLY
TCEEPLKLKYYLLVFLDISPSIHSLEFDRGKDYYFIYPLCKAKPVTTPRPTTTYPTTTAN
PTTTPP
>Btue_12517 B. tuediae
FYTHIVSPGSQINIICPNIATILRNFTQYPLRDSLYENLWIVGKEEFYSCKMNSSKTSSR
LVHVCDRPDTLHYYTVHFIPQNTLLSSSHPGKSRSLYLLESKEKEIERRI*
>Btue_28719 B. tuediae
MAARTLSWIIMAAFLGTLFAFGSSKRIDIDWNPRTSSQLKSNKVVFARIGDTLGITCPFD
KEESMWMVDYRDYVTCNASCDSYTYAAVCNTPNAVTIKELDVWDRDVHPEKKVTFCLAQC
TSLLVRLKERVTRYTPA*
>Nvec_EDO36327 N. vectensis
MNVAAMSKVKIVCPNPTSIVDKVQDDTPKEFLYENLWIVTEESYDKCNTTLSPRNKILLK
CDTPLSLKYYTVAFQQFSATAQGLEFALGQEYYFIATSDGTKSSLDKTSGGNCIKSNMRM
KFYICKSPQDPRCLPPTTAPPTTPSGCPTVPSTTQTPLPPKTNPTTEPTESNEVPLLPTE
NSHTKSSLQSDDPKPTLRPSMAPAVTSIRPQVTKYSSQTEKEAIVREEPCSHDDDPRCLP
PTTAPPTTPSGCPTVPSTTQTPLPPKTNPTTEPTESNEVPLLPTENSHTKSSLQSDDPKP
TLRPSMAPAVTSIRPQVTKYSSQTEKEAIVREEPCSHDHGIRPSWSIIIPLLIIIVLLIA
TNVFFVYQLKQIYTAKDSNICESLDRRSERSKFTLASIGLGRRKDEASPVNV
>Nvec_EDO44950 N. vectensis
MASFKEVRDLLLLEHSNGLISDDELLFFMGEYQSKNPEFTYDIYERFNLDDVEEPECKAY
FRFEKNNIPVLAETLGLPDFFKCTQRTVAGKIEGLCLVLRRMAYPCRLGDLIPVFGRPVQ
ELSMIANCVLEEIYDLHAHQYQSKNPEFTYDIYERFNLDDMEEPECKAYFRFEKNDIPVL
AETLGLPDFFKCTQRTVAANERGCIELNDVRGYSNLRASQANERGCIDLNDVRGYSNLRA
SQEHGYRGLCEIYPPLIWSPLNPAFRPVPFGTENDTVYSMNVLPMSKLYIICPNQVLNPT
KVQLNTPKELLYENLWIVDKNSFDSCQVNTLLRRNKQLMECTTPLALKYYPLVFQDFSAT
ADVLEFERGKDYFFIATSNGQQSSLQSTRNGRCQTHNMKLQVHVCHSATDPVCNPTEPPT
TLPTTTQATTTPQPTTQSTPTTLTTTTAATTTTTPSTSTPTTQPTTLPKVVQAAPPETPS
PRQPEPRTKEDVDVRDSITGPRAGASKGTRCPRDPEDTTWLILVAVMGAIIAVSISCNIY
ILCCKKQSSSHQPRSDDKRSSAAHALLRENSRIDKV
>Nvec_EDO47804 N. vectensis
MDLYLYRIVTLLVLMAANLSLVESMFYKSIVWSPQNPIFSGTAIKCVIPKSKMTIVCPTQ
ALVVQDSPLIPPKEQLYENIWLVNRGSYDSCNISTADSEKRLLLRCDTPLVLKWYTLIFQ
EQSATAQGLEFVPEREYYFIATSTGDKSSLDSTAGGRCSSSRMRLQIHICRDVDDIHCKS
EAQCQALLNKEESTVAPATTTQYHPTDDNFTEGYSRNNLTSTPAPCIGKPDSHGRRDFFT
EMKYQFAVGFLTAVCLVFIVSTAYLGYRLYRKTKESPRMCSENGRFSMRASTRTTSDGAD
SIDGAEHELLRDSSHC
>Pcar_189454 P. carnosa
MGRENCVVLALVYVYLELGALVSGAIIYPSVQWSFQNPRFKGGTFRLNVLPLSKINIICP
HMATSLLTTNDSNSNSHYENFWIVDSSSYHSCNVNTSITNNKILFKCDKPLQLNFEQLVF
QSVGMDNSAVFKKGKTYYFMSTSTGSQDSLDNKKNGHCLTNNMKMQIYICNGTQDVYCSD
VKPTTLPTMVHVGSMRIKGTAKPSATRIETTQLTSSQWQSPTELNSTQTLRGPKVDYLEK
GKEAAVMSVTVWIVIVVMLGAILAVSVGINFHFWRKQKQFGSKKPPYDRGNSLSPPLMDH
V*
>Pcar_235347 P. carnosa
MMYREPYSVLLLAQIVMFFQVLECAVYPSIHWSPQNPLFDNENSILYVLPMSKIHIVCPN
PTTIMKKVKANKSRGKLYQNLWIVSRDSYERCDTDYTPGSRLLLRCIYPLRLKYYTLVFQ
RYSALSSQVFIPGKEYYFIATSDGSQASVDSRSGGNCVNKNMKLRIFVCKDAQDPRCNRK
LTTTATEATTTASTTQSSASSTATNSTVLHVTNSTTTTQAPTEKTTWSTTSSTSESTSTA
VTWTTRAVTWTTQLRASLSSVVPVRSMQRSPLNAVSDLNWTIVIPLMACLLLSLMVNIIL

FCRLKKRKHEYSLEEKPGQQITEGVVVKSSEKQQIEPGVDRTWLLCRRSADVTEV*
>Pcar_240721 P. carnosa
LCVLVFFVNLNCIPASETYKSIIWDPENPLFSVGDASNCPYKYKRLKIHSASKIKFICPN
VATVLQTTSDEVSKTNKYENLWLLYNKESFDKCDTTLDINVTEPAIRCDEPTQLQFHAIT
FQRFAAYEDEPAFKKGHYYYIIATSNGKLTNINDTSGGHCDDTAKNVSMKIEVYVCNNTE
GEDEMCKEGVGELTCPYDTSSSSSESTPTPSTPHVTILSTPSEATATQIPTLTSVTTTST
AWATSSSTASATVNETNNTEKQQGEMGSRKGDSSEDSKTAWMVTALIFMAICIAIVTGLI
WYYFFCYGKKKTCQYETRKVQPSPQYGRKNPAFKADNDGLSEHETGSLPDKETGVVIYNK
EVFYDKQDGGERDKKNPPV*
>Pcar_179493 P. carnosa
MALASLHCSGLTVVFCFCLLLSGSVAEILPSILWHPENQLFKNLGDATRKVMAFDKLSII
CPNLVEYPITRNTSFSKDLLYQNVYMVDENGFNNCNATGGLSILNCDDPTKYSHASIVFQ
PSTADINDPRFKQGKAYYFINTASGLLSSLTNTQGGYCKQGMKMKIYVCNGSTDPNCLEG
NKVIHGGWCDWSECLDGHQTRLCNSPAQANGGLPCVGPDRRLCTTKPSQTFPCSTTNSET
ATEPGLPSKDNRTGDNLSDNELQINKGVFVAICLIILMVGIAVGGVLCLLVYRKQRRNHK
KKVKITRVPSALSGTSRPNSIVSTLSTVSTVSESAPIVPPRS*
>Pcar_215100 P. carnosa
MCHNKVFWFLLLVCHFRSVWNVEYYESVQWTPQNPKFQFSKDDIQDGKAFCKLSVIPTSQ
MIVICPHQTTYLENEQQAIPNEQRYEAVWYVDKQSFHDCKVGKHEGNINAVWLRCDTPDK
LKFNTLVFQRFSTDSLVFPPGSEHYFIGTSDGRKDSLNSTSGGHCDDVLNGVSMKMMIYV
CANETDPACNEKTEPPTTEITESTTGQDPATQAARKSASSDIWHLLTIILGISIAVSLMI
HSLCLVVVCRRRTKKKPIDKAMLRPETDKIGVENIQLNKNNSHSV*
>Pcar_395755 P. carnosa
GQQTSLDHTSGGRCSTHNMRMKIYICNDKNDPKCKDNPVSTVQRTQTITVCPSPTQSIQA
SSIPTQPQYCSATPDKEQVSELLNHTRLIPEIFNHTS
>Adig_03889 A. digitifera
MFKLLTCGKIFGFLRTKQHLMSVMCRSFQLLKGQKIGTSDDNPTGLVFLSLLFLEHAAGR
DDRTFDEGRTYYVMATSDGTQSSINDLSGGHCNDTSNNVDMRIEVYVCNKTGDRICDSTN
VGFFFRQWWMVIAITVSCIAVIVIVIFTVYCCSGKKKCPVVFPTAKETRKPGLGMSNSGY
VTDTMTRRSVYEAANQPSSQKFNHVTIETTSFN*
>Adig_04407 A. digitifera
MAISHQLKYALYGAMILIFGANGYLYPSIHWTSTNPLFKRNRTQCVYPESILYFLCPNTV
TVVAKTQDSFLSPEYENLWLVGKESYERCNVTQGVDRRLQVCNKPFELKSYRVIFREYGA
GNLPVFQPGEDYYFI
>Adig_06508 A. digitifera
MKENIWLLHNKTAFDECDVSQVQVTQKTDFFICDDPTRLVFLSLLFLEHAAGMNDRTFVG
GRTYYLIATSHGTQSSVNDLSGGHCNHKSKNVYMKIEVYVCNKTGDRICNSRNVGILTCP
PPATTAPYTSPNTTTLTHGTELGINITKSVIRDKSETVRTLPTTEAKLRTVIIAEEKITA
TTKPGSTKTAPKRGKRSSENDEVVEMCKSM*
>Adig_07747 A. digitifera
MTCLSQVGFVLVPFLASCIRVQGFLYYPSIQWDHRNTIFQTNYSISVLRRSKLIIVCPNP
ATIPVLVRDSTPMEEMFENLWIVDKPSYDACAVDTSQPQNRIAMNCDTPLELRYYTVVFQ
RFSANPDGLEFEPGKNYYFIAGNCGNSFSSANGKRFSTVDQDNDNSPKSCAQKFKSGWWH
GRCHQSSLNGLYFLGSSSNPYHFASGITWYTWKGYSYSLSKSEMKIRIW*
>Adig_10295 A. digitifera
MKENIWLLHNKTAFDECDVSQVQVTQKTDFFVCDDPTRLVFLSWLFLEHAAGMNDRTFVG
GRTYYLIATSHGTQSSVNNLSGGHCNDTSKNVHMKIEIYVCNKTGDRICNSRNVGILTCP
PPVTTAPYTSPSTSTLTHATELGINITESVVADKNETKSEIPRTETKLTGTIITRENTTE
AARPGSTKNETKRGKCICENDEVVEVCKTRNLWMIIAICVSCIAGVLLTLNVIVICCYCF
GPKGTDHFKTRCHACEKHADSHCAAEFKADGKNCGPGPRQLKVRLNSQIYLVCPNLPTVL
QQRGHDVQTSNMKENIWLLHNKTAFDECDVSQVQVTQKTDFFVCNDPTRLVFLSLLFLEH
AAGMNDRTFVGGRTFYLIATSHGTQSSVNDLSGGHCNDTSKNVHMKIEVYVCNKTGDRIC
NSRNVGILTCPPPVTTAPYTSPSTSTLTRGTELGINITESVVADKNETKSKIPRTETKPT

GTIITGKNTTETVITDKSETVRKLPTTVAELRTAIIAGEKVTKTTRSGSTKTAANRGKRI
GENDEVAEMCKTRYLWMIIAICVSCIAGVLLTLIVIVICCYCFGPKRKDHFKTPCHSSEK
RADCYYAAELDIYDCEDLGKGAQGVTAPQRERKYKCVESPPILTTLL*
>Adig_19031 A. digitifera
MHRIFRVKPTNIMKTLYYFIIFGLASVYSRDIYPSINWDPRNPIFQADGKNCGPGPRQLK
VRLNSQIYLVCPNLPTVLQTRGDEVQTSNMKENIWLLHNKTAFDECDVSQVQDSQNTDFF
MCDDPTRLVFLSLLFLEHAAGKNARTFVGGRTYYLIDRICNSRNVGILTCPSPATAAPYT
SPNTTTLTHGTELGINITESVIRDKSETVRMLPTVTTEAELRTGTVIITGEKITERTKPG
STKNEAKRGKLISENEVVERRKTRNLWMTIAICVSCIAGILLTVIVILVVLNYWLPIRKY
TLAQSSTTPHKRQHSSRDFLMV*
>Adig_20275 A. digitifera
MSIILETKFNDEGRYRRPFLSKLRLDVCQRMATNGRQRYFAIVVAIIHDLVKGLNYPSIH
WSYQNTRFKQGTYHLNVLPDAKVMVICPHTVISLVKTDLQTAADPDNLVENFWQVDYDSF
WSCNVDTSNPSNRLFFKCEDPRSLHYEQFIFQSFGIGNRPTFQKGKTHYFVSTANGSKES
LDKTSGGHCEEFNMRLEIYVCQNSADVLCTEAQSTAPLTTVPTVSFSGTDVPGVSLTPDG
KPQQNKGTLNVFERNVWLKIAIIPLGFLLLISVFVNIFLIVKIRNRKCSTPEMRDSNKHL
SNYSGST*
>Adig_20773 A. digitifera
MKRFKADGKNCGPGPRQLKVRLNSQIYLVCPNLPTVLQQRGHDVQTSNMKENIWLLHNKT
AFDECDVSQVQVTQKTDFFICEDPTRLVFLSLLFLEHAAGMNDRTFVGGRTYYLIATSHG
TQSSVNDLSGGHCNHTSKNVYMKIEVYVCNKTGETFNQRANLTTLDRICNSRNVGILTCP
PPATAAPYTSPDITTLTQTEQRVNITESVVTYKSETLSSTKLPRTESKPTGTTRTGEKTT
ETTRARSTKNETKRGKRIAENEMRKTLNLWRTIAICVSCIAGVLLTAIVILVVRNSWLPF
RKYTLAQCSITQNKTQHSSQESKTVGIQVRH*
>Ever_204 E. verrucosa
FPHDVVSYYQYPSVYWAPKNCRFNLNKPPNTGYNFTVKPFGKLYFVCPNIALWNDMSSTG
PQLSMMYENLMLVDKEGYDTCTVNTSRNPRLNRAILKCDGDASKLKYMQETFSRQRADPH
RMKYNKGQTYYFISTSNGSKESLDNLSGGHCKTHNMKLKITVCGSQDRCTFRMPKCPFTN
VFYKPTTATTRQARTSQTTVPENTATVATIATTETTA
>Ever_1897 E. verrucosa
MLRQALSCSFFALIFFPRVAYLTFYPTVFWTPNNCRFNLNKPNGKGYFIKVKSMSNMVFM
CPHFSIWAGKSDTSPVRTMMHENILLVDKESHKSCSIKNSTRAKRLLTCDNDPMDSHVKY
TNEKFASTQAYADKQCYKKGHHYYFISTSDGSVESLNNTEGGHCKTHHMKLHIYVCNKTE
PCEFEMPECPKKVEKGNNRTPSTHNCSDDCKDCKDSVTTRAQTAKQDWFTDNRHPTIYAI
MGLVIVILFVIILYLLCENRKYRKSGATIDEKNKDSCDKVLI*
>Ever_13730 E. verrucosa
VKTRKVNNDDEIRIWCPKYYTQGIKTTYQLHQSKLYFALYKVQTKEEYDTCDARKGKRLL
YCNDPSGKSTKDTEERQIVFLSNPISNLMDPYPPGETYYFIGTSGGMEKDIDKKVGGKCS
SHNMKFAIYVCREDENCSVDVLNGGWSLWKVNEETGLCERNCSNPAPQNGGFPCTGPKQM
DCSKIINGRKAKPTTTRESTETTTTAVKESEKSTKTSLTSRQQKVSTTTVTDKMIHKEQP
TSTKSGPTESTTDSTMSDKDTLDNKPEPEAHNSDDVTLSMKGAVLTFVGLFILGCIVGIV
QVLVILHVRSKRSGKQPEPQSPVNHV*
>Ever_19722 E. verrucosa
LFWDTNNPILQRRFWSEVVQVKIHDRITFICPYEIIKIRGSETEQGVYIDGLYQNLYVKE
VPADITNFDGACDTSENSTAIFIMNCDKPKEFYHKNIPINLNQAHNDLPKFKYGKDYMFF
ATSDGSKESLQSRNNTSKNCTMVFRIHFNEKKEDTPFPTCPLFERRSVCEKPSIPKEKKQ
NGSKEEKQKEIVECKNITPQRLNITTTTYNEVTVTWQPPSNISCNITNYSICYRNNLNEP
CSLEVLVPGKKHSVTLNGLAADHKYI

Ephrins in Ctenophores

>Mlei_03441 M. leidyi
MNQHSLLNCTRDTPRGRDIYTALIVQHDVRDPFFKDYIIGQNYYFISTASRKGTDRERIA

NTAGGNCKYNRMKITLHVVNSLSKSTSDISENGASSVPDPGGPAPPDEQDPGKVHASSIP
NETDVPRQNNNYTSFEEGTGVQLDSSAPDNGSTMARNDRVLAILLLTLIYILNS

Ephrins in sponges

>Aque_39602 A. queenslandica
MERRSSSALFLVSVLLYLQGVRAEGGILYANSTNLTLYQDNPFRVAFLARLRVVCPDLDY
GLYRVSQKGYNNCSNDGQQLIGHCGNTGILSEIIYSVPLSGGLAWEVDMTYYLIFKYTAN
ESPAPCDEGYKFKVFVYDPRSTSVTPTPTAMPMSSSSSSSSSSVVISSSSSSILSQLLTP
SLSQSRIHTVSPSSTETTVTGPTHEPSSSSTSEIETTEVRATSTGLINVNPTPPTVQPSV
STTVSGATSTTASLTLALSLLALLLILLL*
>Snux_75319 S. nux
MMFSRFIFVLYYASVCLAANYDVIWDQDYCTDKDRYPESYEVPHYTPCAVCINDVITLRI
NSSTDTYENLYRVPTETDMLNCDATVNDNTEIIVFNDKQEITVRSGGNEPALSFLVRAEP
YYFISTSDGTQNSAENDLMKSPNTCLQFAFTVLLNISPDCGNYTANCEFKSVFTHPPSSL
KCRKISPTPTTATTTPKRNPIPSSHHPEVFVYVFIGLTSFAIIFFIVCGAITLSLFWFDT
IPIQRGKTRQYKIPMTPI*
>Cvar_2778 C. varians
MMSFVISFVVSVLFALLEGSHMVSLSRATTTPTVWNLTWSADVSTQYSPSSPLRVYLGDQ
LKFSCPVGATSNIWIQEYLQQYEQCSCNATGMGNHTTDCTSHTTTKQICQDEVLIAITSD
SNSLRSALSFDSGKTYYLISYSTGDRPLAGYGNNISGGECLEGFKMVFEVVSTATEAVTT
TTMAPRVIEATETSMDSQLPTMDSSQPGLVNENNGDPFSVAAEGLASKAIATTITGVYLT
LLMTSIVLALSCM*
>Cvar_8977 C. varians
MVNLFIFVVVALISALWNSWWVSSQTSLSSIHSLGVGRNIHIVWSADVSSIHPPSNPLRV
KLGDKLNITCPINATSNIWIQRHLQQYEECNCSAVMTTMNSTASDCVPTDTTVRHVCQDE
VFLDITLDSKNLNSALSFQQNTTFYLISYSTGNGTLAGYGKLTKGGQCTKGFKMAVTVLP
NSDPGLPHVDVNSTVDLLMKNRTDTVGLESDGGTINGAVVNLTAGLTLSFLCFISLLAIT
*
>Cvar_1084 C. varians
LGSNFIFCKQIINQSSNSKMHATFLTVVTLSLLTMSEIWSTAKTAPVIEKPDQDRVINLL
WSANVSSLYPPSSPLKVSLGNKLNVTCPANATSNIWIQNHLEQYDECNCNATSVENPSCM
PDITTRQICQDEVMIAITLDSKDNPSALSFEPDTTYYLISYSRGDQPLAGYKDHNSGGEC
WKGFRMALQVLSSELPRIANPGDDTSAPKSTVPTPESEEPTYSGTPPSLVNEVSQFSKSP
NSGNLVPSGALAVFTGPTKLISVISVSLFSLVILL*
>Cvar_12230 C. varians
LAQADNLLARAPMSLGSQLRLQRSMYLTITPLMNPRVVMGTLLLMLDFFNTYSLPTGATV
MTSMSTTVVEQSTTTLPITLDWSSNVSSMYPPSSPLTVRLGDELKINCPEDATSNIWIQK
YLQQYEQCDCNTATDHEGVSNYSDTTDLCVESTTTRHVCQDEVLLAITSDSKDLNSALSF
KPGSTYYFISYSPGDRALAGYSSNTAGGECLLGYKMAIYIQPNDPPTTTELVTTSTTMVT
TTGPLTTSATTKVRVASTEPALQDTPFTGDVRTTTSGTVTLFNLNYLTIIGLLSLWCVSQ
*
>Kvar_23578 K. variolosa
MMDACRGLVLVSLGLTVTLTSGLTVHNITWTTEGVQQYGTSTPLRVRVGDMVKFCCTDNL
EFSEITILDDVRQFESCDCSPVPELSCLSIQLVVSRCAQLLPRPSIVNIYRSGLPGVWDY
TPGIIYYLTSFPFGNLSGGACTDPGLKMAMTVFSTDSVIDDESPATPVQTTHRHLVDVTN
AGLRNDSHPLVPRLHYILTLMITQLIAVTLATRKS*
>Cpro_58774.15 C. prolifera
LTMYCGCSVSLAVSLLSLISVSSAAEYTLNWTTAGVAALHWNISVDIGDKLILLCVPGSY
SNVVEVGDKSQQLACNSTTATELRGSCIGRVNGTRLIFNIEQASAKLPGDFGFVEGEDSF
LTSFSNGLDATSSFSDIKSGGECTEGLKLTVHVRDSSPTVSKPATTTETNSVDQTTDSLP
PSSSTTETKPVDFTEFELSPEGLISGSSPLHRTSTILLLTCTVLLAFMLRSHS

>Lapi_57857	L. apicalis
RPSAGECIRYFVIMESERFLAQVKWCLALALALLSLGLKRCDAARYRLTWNATDLIAVNN
EINVELGDTLKLICPVGSFSNAIIVSTKEQFDACNSTIGPQITNNVRGNCLGVTHTTELI
LNIEEPSAVLTSDFSFTDGEISYIASFSDGMSLSSSYTSRIAGGECRDGLKLAIRVGSSL
PIDLEAPTPVDDPTLTPTVIGGGLNSSSSATSLTSSITSITSITSVAMLIHLSNCN*
>Lapi_139996	L. apicalis
MASLSSYATSCSKVASQALVLCLCLVPLCSCVQHHSISWTPEGVRQYNTSNPLQVKLGDF
VTFFCAQSGHYNYSNLWIQYREEQYNQCNCQVVPGDSCDPNAARNGYCAVDSGNTELIVT
ITRNDGDLGSVINFRPGLVYYLASYADVLPTLEGALSEDSVGGQCLQGLRMSLSVAEEPV
KPAPPRSTSQGTTPGTEISEPVPTPTAGGMSSGSPTTDTTGSETEMTTTTAPQLEFLKAR
KVLDWHIIVMVVFGALIVNLLVVIIASSVLVHRRRRRRTISKVDLGTCEEKIPDSPTPLY
PNQVFDDPLN*
>Slac_8385	S. lacustris
VVNVSDSIKFTCSGSVSNVVILASQQQWQSCNATTGGAPFQAGYCVTATGTITLNIVHQS
NTVADTFSFNMGQDYYLASFSDGTLTGAYYDVTSGGECLSGVKMVIRVPGSSPTSSSSSS
>Ifas_6298	I. fasciculata
MAFGLFITLLLLAFASEKVLSQDRHEIIWDRYWLRDNLTNLTLHVALNDQVAIFCHVDED
TERPEDIAENVVYLQEEKNLTNCDTSQTGESSLLFECLNQGIDHVINIVDSLQLTNRRSF
EAGGAYYFTSFSDGTVGSAQQEASRGGACMADPPLRLTISVSATETSFPSPTMTVSEMST
TIISSTMDNVMPTPTVMSTSTSTSSSTTTNPTSEPSELPTPPSVTRDALSGAKLLSPIHS
VLLLVPLFTCIMIL*
>Ifas_6301	I. fasciculata
MAFGLFITLLLLAFASEKVLSLDSHNVTWERHWLRDNLVNLTLNVALNDRVVIFCHIEDT
ELPADQYENVVYLAEEKNLTNCDIGSDIGQTGESSLLFECLNQGIDHVINIVNSLQLTNR
RSFEAGGAYYFTSFSDGTIDSAQQDASRGGACMADPPLRLTISVSATETSFPSPTMTVSE
MSTTSISSTMDNVMLTPTVMSTSTSTSSSTTTNPTSEPSELPTPPSVTRDALSGAKLLSP
THSVLLLVPLFTCIMIL*

Ephrin like in Chonanoflagellates.

>m.393972_Acanthoeca_spectabilis
MRSRPSVGWLAVAGATVSVVAGQQFWTEGNTTDLTINLLNLRVWPWGATYRMRWDAVNPPAQLFFTCSDDLDVERVNQAAFNGCTI
ATGSGGDTIPPSSSSPGVTASCQANHQTTTDSRPLSLSSTTSDTFYYISLTNGTGSRLGGGATQGGACATRGAKVAVEYIPINSPLLASGGAY
SCDGKACPTQTTCRFRPCSFGSCFPEQLVSAGTTCDDENSLTASDECNALGECAGTANYTVTLRFATNTTALIPASTVPLQPFQLAGVVSNL
TGGMPLTLANAFQFSVVEEFAATHVDVQFSVVDFRNYNGGSQSLAAEMNRVVGRTVLYRNAQRVITAVIAVSDTPTQSPTVQVATGAPT
AAGQANAAAGAGGVVGTLEDNYIYIAAGAGVFVLIAILVVVRSRKKRDGPTGPIAPRSPPALNPTYQPEEDVNQIIDRTSIRVVAGQTRPRA
DTGDTA
>m.134676_Salpingoeca_urceolata
NNNNNNKRSQCMFRVRWLRQSPGGHRVKATQQHDNNNSILTMGRVLLAVALCVLAVLDVGCSGDPVRVLNVDLTEPPTPGPHQVYLN
GDIIFDCASDYEIYLVDKIGFDTCNITTLDRLAAYTIRLVCTAGSTVTQAVTPFETGNVNDAYSSDPVFDRFEAGKTYYFTSVTGGTEERAGLQ
PGDKHETGLGGLCSTGSKLEIQVVADVCVEVDCCENAGSGVCFLHKTTNDLACTNHAYLDSSTAVVDCDDGISTTVNDTCNNYGVCKGVG
ILSLSLDLNFGPAPGSLTSSGPGAVTLTDQQLASFVTQTANKLGVSSDTLNNVRFTVTDSGDLLAKLDVTQLAERSDANVTALETTAITIPTSD
GLGTIDLTVESVNSQGSTGGATNKSSGWQSWQTAVLVIGIIAVAAVGLVAHLKSSGGKNNRTVPDIPSYDQEHEVGVLHLNPVSRSTPYVE
TRDSASC
>m.338217_Stephanoeca_diplocostata_aus
LSLWDFYLWCPSRVLNQELTKILLLIFYSHSHHGEVLKIRSRDILIPCRRSCTRCPAVALQQAVELLILFLTCLYSPCLSCMIFFFFHSCCSVSVCR
WCQFALIKHFLWICLPGTYRMWDNNAGPSKSVTFICSQPCSFLRVEEEAFNTCDVTGGGAATSSAAILCTPGDSPITNTPSFGRTPGTYYWI
SLTNGTGERADRTSEPGLGGACSQGAKARVEIMQKNPVNEALYNCTGITCTSTRLCHTLDCAWGRCMDEARAENMPDGTACDDGNPLT
PDDECGNGFCAGTDDYWFTVLVNATTPLSTGAMSLSQEDGVANSIQTCSGVSGFTYVSDVISSPLLATVNFSLPTYREFGANALDDSLLQC
VGTSLSFRGSTCSVEDIPFVSETETPAPTQAPTTAAPTDTPTSSAPTQVPTDRPTIQPTVTGGTFFPTTASPTKPGDTLAPSTLSPTTVAQGLP

SSSNKESSFTSSVYFYGAAGAAGLVVLVVLICCCCCRKGRSKNSSPVDPRRNTTSDGRLPMHENPMYGSNKTYETETPAIESGTSERISFHAG
RTGASADVDVGPSAPPPPPPAVSSTDEVRAAWGSMW
>m.130281_ Salpingoeca_infusionum
MRKPPFLQASTNTSVHSRSNHHSMQVLVLLLATLVAVTRAGTFLNTVTVHDVDILSSMPPEGYKVHLNDEIEIACDEQSKYNLVSKEGYDSC
DLTIEGHLLFRSIITCRPGYPGSTHIQAATDSQYSYFDENDPIKGFEIGKQYYLASVTGGNGSMPGRPSDPGRGGICLQGAKFTFSVVEDPVC
DLACCPGSEGICMFSLSSPNQPGCQDYEYDGAGASCDDGLAETFNTKCNAFGACHGSMVLTLYVDTEVDSETLAKFGTLKADNVTVELLSS
TRVSIINSINSTASLPLEALSSVQASLGSFLEISVQVEVWGRAHITQIRNALNGRHFLASGAVYRWYTEEPQATTASGSTAGLSSSQVIGLGAG
LGTVVLVVVIVGVVVACRKSNSSPSAVKPSGSGAFTGDYAFDSKNVVQPMYTNPLAPATFDESVA

Eph receptors downloaded from databases/previous studies (Mellott DO and Burke RD, BMC CELL BIOL 2008)

>Hsap_EPHA1 NP_005223.3 ephrin receptor EphA1 [Homo sapi
ens]
MERRWPLGLGLVLLLCAPLPPGARAKEVTLMDTSKAQGELGWLLDPPKDGWSEQQQILNG
TPLYMYQDCPMQGRRDTDHWLRSNWIYRGEEASRVHVELQFTVRDCKSFPGGAGPLGCKE
TFNLLYMESDQDVGIQLRRPLFQKVTTVAADQSFTIRDLASGSVKLNVERCSLGRLTRRG
LYLAFHNPGACVALVSVRVFYQRCPETLNGLAQFPDTLPGPAGLVEVAGTCLPHARASPR
PSGAPRMHCSPDGEWLVPVGRCHCEPGYEEGGSGEACVACPSGSYRMDMDTPHCLTCPQQ
STAESEGATICTCESGHYRAPGEGPQVACTGPPSAPRNLSFSASGTQLSLRWEPPADTGG
RQDVRYSVRCSQCQGTAQDGGPCQPCGVGVHFSPGARGLTTPAVHVNGLEPYANYTFNVE
AQNGVSGLGSSGHASTSVSISMGHAESLSGLSLRLVKKEPRQLELTWAGSRPRSPGANLT
YELHVLNQDEERYQMVLEPRVLLTELQPDTTYIVRVRMLTPLGPGPFSPDHEFRTSPPVS
RGLTGGEIVAVIFGLLLGAALLLGILVFRSRRAQRQRQQRQRDRATDVDREDKLWLKPYV
DLQAYEDPAQGALDFTRELDPAWLMVDTVIGEGEFGEVYRGTLRLPSQDCKTVAIKTLKD
TSPGGQWWNFLREATIMGQFSHPHILHLEGVVTKRKPIMIITEFMENGALDAFLREREDQ
LVPGQLVAMLQGIASGMNYLSNHNYVHRDLAARNILVNQNLCCKVSDFGLTRLLDDFDGT
YETQGGKIPIRWTAPEAIAHRIFTTASDVWSFGIVMWEVLSFGDKPYGEMSNQEVMKSIE
DGYRLPPPVDCPAPLYELMKNCWAYDRARRPHFQKLQAHLEQLLANPHSLRTIANFDPRM
TLRLPSLSGSDGIPYRTVSEWLESIRMKRYILHFHSAGLDTMECVLELTAEDLTQMGITL
PGHQKRILCSIQGFKD
>Hsap_EPHA2 NP_004422.2 ephrin type-A receptor 2 isoform
1 precursor [Homo sapiens]
MELQAARACFALLWGCALAAAAAAQGKEVVLLDFAAAGGELGWLTHPYGKGWDLMQNIMN
DMPIYMYSVCNVMSGDQDNWLRTNWVYRGEAERIFIELKFTVRDCNSFPGGASSCKETFN
LYYAESDLDYGTNFQKRLFTKIDTIAPDEITVSSDFEARHVKLNVEERSVGPLTRKGFYL
AFQDIGACVALLSVRVYYKKCPELLQGLAHFPETIAGSDAPSLATVAGTCVDHAVVPPGG
EEPRMHCAVDGEWLVPIGQCLCQAGYEKVEDACQACSPGFFKFEASESPCLECPEHTLPS
PEGATSCECEEGFFRAPQDPASMPCTRPPSAPHYLTAVGMGAKVELRWTPPQDSGGREDI
VYSVTCEQCWPESGECGPCEASVRYSEPPHGLTRTSVTVSDLEPHMNYTFTVEARNGVSG
LVTSRSFRTASVSINQTEPPKVRLEGRSTTSLSVSWSIPPPQQSRVWKYEVTYRKKGDSN
SYNVRRTEGFSVTLDDLAPDTTYLVQVQALTQEGQGAGSKVHEFQTLSPEGSGNLAVIGG
VAVGVVLLLVLAGVGFFIHRRRKNQRARQSPEDVYFSKSEQLKPLKTYVDPHTYEDPNQA
VLKFTTEIHPSCVTRQKVIGAGEFGEVYKGMLKTSSGKKEVPVAIKTLKAGYTEKQRVDF
LGEAGIMGQFSHHNIIRLEGVISKYKPMMIITEYMENGALDKFLREKDGEFSVLQLVGML
RGIAAGMKYLANMNYVHRDLAARNILVNSNLVCKVSDFGLSRVLEDDPEATYTTSGGKIP
IRWTAPEAISYRKFTSASDVWSFGIVMWEVMTYGERPYWELSNHEVMKAINDGFRLPTPM
DCPSAIYQLMMQCWQQERARRPKFADIVSILDKLIRAPDSLKTLADFDPRVSIRLPSTSG
SEGVPFRTVSEWLESIKMQQYTEHFMAAGYTAIEKVVQMTNDDIKRIGVRLPGHQKRIAY
SLLGLKDQVNTVGIPI
>Hsap_EPHA3 NP_005224.2 ephrin type-A receptor 3 isoform
a precursor [Homo sapiens]
MDCQLSILLLLSCSVLDSFGELIPQPSNEVNLLDSKTIQGELGWISYPSHGWEEISGVDE

HYTPIRTYQVCNVMDHSQNNWLRTNWVPRNSAQKIYVELKFTLRDCNSIPLVLGTCKETF
NLYYMESDDDHGVKFREHQFTKIDTIAADESFTQMDLGDRILKLNTEIREVGPVNKKGFY
LAFQDVGACVALVSVRVYFKKCPFTVKNLAMFPDTVPMDSQSLVEVRGSCVNNSKEEDPP
RMYCSTEGEWLVPIGKCSCNAGYEERGFMCQACRPGFYKALDGNMKCAKCPPHSSTQEDG
SMNCRCENNYFRADKDPPSMACTRPPSSPRNVISNINETSVILDWSWPLDTGGRKDVTFN
IICKKCGWNIKQCEPCSPNVRFLPRQFGLTNTTVTVTDLLAHTNYTFEIDAVNGVSELSS
PPRQFAAVSITTNQAAPSPVLTIKKDRTSRNSISLSWQEPEHPNGIILDYEVKYYEKQEQ
ETSYTILRARGTNVTISSLKPDTIYVFQIRARTAAGYGTNSRKFEFETSPDSFSISGESS
QVVMIAISAAVAIILLTVVIYVLIGRFCGYKSKHGADEKRLHFGNGHLKLPGLRTYVDPH
TYEDPTQAVHEFAKELDATNISIDKVVGAGEFGEVCSGRLKLPSKKEISVAIKTLKVGYT
EKQRRDFLGEASIMGQFDHPNIIRLEGVVTKSKPVMIVTEYMENGSLDSFLRKHDAQFTV
IQLVGMLRGIASGMKYLSDMGYVHRDLAARNILINSNLVCKVSDFGLSRVLEDDPEAAYT
TRGGKIPIRWTSPEAIAYRKFTSASDVWSYGIVLWEVMSYGERPYWEMSNQDVIKAVDEG
YRLPPPMDCPAALYQLMLDCWQKDRNNRPKFEQIVSILDKLIRNPGSLKIITSAAARPSN
LLLDQSNVDITTFRTTGDWLNGVWTAHCKEIFTGVEYSSCDTIAKISTDDMKKVGVTVVG
PQKKIISSIKALETQSKNGPVPV
>Hsap_EPHA4   NP_004429.1 ephrin type-A receptor 4 isoform
a precursor [Homo sapiens]
MAGIFYFALFSCLFGICDAVTGSRVYPANEVTLLDSRSVQGELGWIASPLEGGWEEVSIM
DEKNTPIRTYQVCNVMEPSQNNWLRTDWITREGAQRVYIEIKFTLRDCNSLPGVMGTCKE
TFNLYYYESDNDKERFIRENQFVKIDTIAADESFTQVDIGDRIMKLNTEIRDVGPLSKKG
FYLAFQDVGACIALVSVRVFYKKCPLTVRNLAQFPDTITGADTSSLVEVRGSCVNNSEEK
DVPKMYCGADGEWLVPIGNCLCNAGHEERSGECQACKIGYYKALSTDATCAKCPPHSYSV
WEGATSCTCDRGFFRADNDAASMPCTRPPSAPLNLISNVNETSVNLEWSSPQNTGGRQDI
SYNVVCKKCGAGDPSKCRPCGSGVHYTPQQNGLKTTKVSITDLLAHTNYTFEIWAVNGVS
KYNPNPDQSVSVTVTTNQAAPSSIALVQAKEVTRYSVALAWLEPDRPNGVILEYEVKYYE
KDQNERSYRIVRTAARNTDIKGLNPLTSYVFHVRARTAAGYGDFSEPLEVTTNTVPSRII
GDGANSTVLLVSVSGSVVLVVILIAAFVISRRRSKYSKAKQEADEEKHLNQGVRTYVDPF
TYEDPNQAVREFAKEIDASCIKIEKVIGVGEFGEVCSGRLKVPGKREICVAIKTLKAGYT
DKQRRDFLSEASIMGQFDHPNIIHLEGVVTKCKPVMIITEYMENGSLDAFLRKNDGRFTV
IQLVGMLRGIGSGMKYLSDMSYVHRDLAARNILVNSNLVCKVSDFGMSRVLEDDPEAAYT
TRGGKIPIRWTAPEAIAYRKFTSASDVWSYGIVMWEVMSYGERPYWDMSNQDVIKAIEEG
YRLPPPMDCPIALHQLMLDCWQKERSDRPKFGQIVNMLDKLIRNPNSLKRTGTESSRPNT
ALLDPSSPEFSAVVSVGDWLQAIKMDRYKDNFTAAGYTTLEAVVHVNQEDLARIGITAIT
HQNKILSSVQAMRTQMQQMHGRMVPV
>Hsap_EPHA5   NP_004430.3 ephrin receptor EphA5 isoform a
[Homo sapiens]
MRGSGPRGAGHRRPPSGGGDTPITPASLAGCYSAPRRAPLWTCLLLCAALRTLLASPSNE
VNLLDSRTVMGDLGWIAFPKNGWEEIGEVDENYAPIHTYQVCKVMEQNQNNWLLTSWISN
EGASRIFIELKFTLRDCNSLPGGLGTCKETFNMYYFESDDQNGRNIKENQYIKIDTIAAD
ESFTELDLGDRVMKLNTEVRDVGPLSKKGFYLAFQDVGACIALVSVRVYYKKCPSVVRHL
AVFPDTITGADSSQLLEVSGSCVNHSVTDEPPKMHCSAEGEWLVPIGKCMCKAGYEEKNG
TCQVCRPGFFKASPHIQSCGKCPPHSYTHEEASTSCVCEKDYFRRESDPPTMACTRPPSA
PRNAISNVNETSVFLEWIPPADTGGRKDVSYYIACKKCNSHAGVCEECGGHVRYLPRQSG
LKNTSVMMVDLLAHTNYTFEIEAVNGVSDLSPGARQYVSVNVTTNQAAPSPVTNVKKGKI
AKNSISLSWQEPDRPNGIILEYEIKYFEKDQETSYTIIKSKETTITAEGLKPASVYVFQI
RARTAAGYGVFSRRFEFETTPVFAASSDQSQIPVIAVSVTVGVILLAVVIGVLLSGSCCE
CGCGRASSLCAVAHPSLIWRCGYSKAKQDPEEEKMHFHNGHIKLPGVRTYIDPHTYEDPN
QAVHEFAKEIEASCITIERVIGAGEFGEVCSGRLKLPGKRELPVAIKTLKVGYTEKQRRD
FLGEASIMGQFDHPNIIHLEGVVTKSKPVMIVTEYMENGSLDTFLKKNDGQFTVIQLVGM
LRGISAGMKYLSDMGYVHRDLAARNILINSNLVCKVSDFGLSRVLEDDPEAAYTTRGGKI
PIRWTAPEAIAFRKFTSASDVWSYGIVMWEVVSYGERPYWEMTNQDVIKAVEEGYRLPSP
MDCPAALYQLMLDCWQKERNSRPKFDEIVNMLDKLIRNPSSLKTLVNASCRVSNLLAEHS

PLGSGAYRSVGEWLEAIKMGRYTEIFMENGYSSMDAVAQVTLEDLRRLGVTLVGHQKKIM
NSLQEMKVQLVNGMVPL
>Hsap_EPHA6 sp|Q9UF33.3|EPHA6_HUMAN RecName: Full=Ephrin typ
e-A receptor 6; AltName: Full=EPH homology kinase 2; Short=E
HK-2; AltName: Full=EPH-like kinase 12; Short=EK12; Flags: P
recursor
MGGCEVREFLLQFGFFLPLLTAWPGDCSHVSNNQVVLLDTTTVLGELGWKTYPLNGWDAI
TEMDEHNRPIHTYQVCNVMEPNQNNWLRTNWISRDAAQKIYVEMKFTLRDCNSIPWVLGT
CKETFNLFYMESDESHGIKFKPNQYTKIDTIAADESFTQMDLGDRILKLNTEIREVGPIE
RKGFYLAFQDIGACIALVSVRVFYKKCPFTVRNLAMFPDTIPRVDSSSLVEVRGSCVKSA
EERDTPKLYCGADGDWLVPLGRCICSTGYEEIEGSCHACRPGFYKAFAGNTKCSKCPPHS
LTYMEATSVCQCEKGYFRAEKDPPSMACTRPPSAPRNVVFNINETALILEWSPPSDTGGR
KDLTYSVICKKCGLDTSQCEDCGGGLRFIPRHTGLINNSVIVLDFVSHVNYTFEIEAMNG
VSELSFSPKPFTAITVTTDQDAPSLIGVVRKDWASQNSIALSWQAPAFSNGAILDYEIKY
YEKEHEQLTYSSTRSKAPSVIITGLKPATKYVFHIRVRTATGYSGYSQKFEFETGDETSD
MAAEQGQILVIATAAVGGFTLLVILTLFFLITGRCQWYIKAKMKSEEKRRNHLQNGHLRF
PGIKTYIDPDTYEDPSLAVHEFAKEIDPSRIRIERVIGAGEFGEVCSGRLKTPGKREIPV
AIKTLKGGHMDRQRRDFLREASIMGQFDHPNIIRLEGVVTKRSFPAIGVEAFCPSFLRAG
FLNSIQAPHPVPGGGSLPPRIPAGRPVMIVVEYMENGSLDSFLRKHDGHFTVIQLVGMLR
GIASGMKYLSDMGYVHRDLAARNILVNSNLVCKVSDFGLSRVLEDDPEAAYTTTGGKIPI
RWTAPEAIAYRKFSSASDAWSYGIVMWEVMSYGERPYWEMSNQDVILSIEEGYRLPAPMG
CPASLHQLMLHCWQKERNHRPKFTDIVSFLDKLIRNPSALHTLVEDILVMPESPGEVPEY
PLFVTVGDWLDSIKMGQYKNNFVAAGFTTFDLISRMSIDDIRRIGVILIGHQRRIVSSIQ
TLRLHMMHIQEKGFHV
>Hsap_EPHA7   NP_004431.1 ephrin type-A receptor 7 isoform
1 precursor [Homo sapiens]
MVFQTRYPSWIILCYIWLLRFAHTGEAQAAKEVLLLDSKAQQTELEWISSPPNGWEEISG
LDENYTPIRTYQVCQVMEPNQNNWLRTNWISKGNAQRIFVELKFTLRDCNSLPGVLGTCK
ETFNLYYYETDYDTGRNIRENLYVKIDTIAADESFTQGDLGERKMKLNTEVREIGPLSKK
GFYLAFQDVGACIALVSVKVYYKKCWSIIENLAIFPDTVTGSEFSSLVEVRGTCVSSAEE
EAENAPRMHCSAEGEWLVPIGKCICKAGYQQKGDTCEPCGRGFYKSSSQDLQCSRCPTHS
FSDKEGSSRCECEDGYYRAPSDPPYVACTRPPSAPQNLIFNINQTTVSLEWSPPADNGGR
NDVTYRILCKRCSWEQGECVPCGSNIGYMPQQTGLEDNYVTVMDLLAHANYTFEVEAVNG
VSDLSRSQRLFAAVSITTGQAAPSQVSGVMKERVLQRSVELSWQEPEHPNGVITEYEIKY
YEKDQRERTYSTVKTKSTSASINNLKPGTVYVFQIRAFTAAGYGNYSPRLDVATLEEATG
KMFEATAVSSEQNPVIIAVVAVAGTIILVFMVFGFIIGRRHCGYSKADQEGDEELYFHF
KFPGTKTYIDPETYEDPNRAVHQFAKELDASCIKIERVIGAGEFGEVCSGRLKLPGKRDV
AVAIKTLKVGYTEKQRRDFLCEASIMGQFDHPNVVHLEGVVTRGKPVMIVIEFMENGALD
AFLRKHDGQFTVIQLVGMLRGIAAGMRYLADMGYVHRDLAARNILVNSNLVCKVSDFGLS
RVIEDDPEAVYTTTGGKIPVRWTAPEAIQYRKFTSASDVWSYGIVMWEVMSYGERPYWDM
SNQDVIKAIEEGYRLPAPMDCPAGLHQLMLDCWQKERAERPKFEQIVGILDKMIRNPNSL
KTPLGTCSRPISPLLDQNTPDFTTFCSVGEWLQAIKMERYKDNFTAAGYNSLESVARMTI
EDVMSLGITLVGHQKKIMSSIQTMRAQMLHLHGTGIQV
>Hsap_EPHA8   NP_065387.1 ephrin type-A receptor 8 isoform
1 precursor [Homo sapiens]
MAPARGRLPPALWVVTAAAAAATCVSAARGEVNLLDTSTIHGDWGWLTYPAHGWDSINEV
DESFQPIHTYQVCNVMSPNQNNWLRTSWVPRDGARRVYAEIKFTLRDCNSMPGVLGTCKE
TFNLYYLESDRDLGASTQESQFLKIDTIAADESFTGADLGVRRLKLNTEVRSVGPLSKRG
FYLAFQDIGACLAILSLRIYYKKCPAMVRNLAAFSEAVTGADSSSLVEVRGQCVRHSEER
DTPKMYCSAEGEWLVPIGKCVCSAGYEERRDACVACELGFYKSAPGDQLCARCPPHSHSA
APAAQACHCDLSYYRAALDPPSSACTRPPSAPVNLISSVNGTSVTLEWAPPLDPGGRSDI
TYNAVCRRCPWALSRCEACGSGTRFVPQQTSLVQASLLVANLLAHMNYSFWIEAVNGVSD
LSPEPRRAAVVNITTNQAAPSQVVVIRQERAGQTSVSLLWQEPEQPNGIILEYEIKYYEK

DKEMQSYSTLKAVTTRATVSGLKPGTRYVFQVRARTSAGCGRFSQAMEVETGKPRPRYDT
RTIVWICLTLITGLVVLLLLLICKKRHCGYSKAFQDSDEEKMHYQNGQAPPPVFLPLHHP
PGKLPEPQFYAEPHTYEEPGRAGRSFTREIEASRIHIEKIIGSGDSGEVCYGRLRVPGQR
DVPVAIKALKAGYTERQRRDFLSEASIMGQFDHPNIIRLEGVVTRGRLAMIVTEYMENGS
LDTFLRTHDGQFTIMQLVGMLRGVGAGMRYLSDLGYVHRDLAARNVLVDSNLVCKVSDFG
LSRVLEDDPDAAYTTTGGKIPIRWTAPEAIAFRTFSSASDVWSFGVVMWEVLAYGERPYW
NMTNRDVISSVEEGYRLPAPMGCPHALHQLMLDCWHKDRAQRPRFSQIVSVLDALIRSPE
SLRATATVSRCPPPAFVRSCFDLRGGSGGGGGLTVGDWLDSIRMGRYRDHFAAGGYSSLG
MVLRMNAQDVRALGITLMGHQKKILGSIQTMRAQLTSTQGPRRHL
>Hsap_EPHA10 sp|Q5JZY3.2|EPHAA_HUMAN RecName: Full=Ephrin
type-A receptor 10; Flags: Precursor
METCAGPHPLRLFLCRMQLCLALLLGPWRPGTAEEVILLDSKASQAELGWTALPSNGWEE
ISGVDEHDRPIRTYQVCNVLEPNQDNWLQTGWISRGRGQRIFVELQFTLRDCSSIPGAAG
TCKETFNVYYLETEADLGRGRPRLGGSRPRKIDTIAADESFTQGDLGERKMKLNTEVREI
GPLSRRGFHLAFQDVGACVALVSVRVYYKQCRATVRGLATFPATAAESAFSTLVEVAGTC
VAHSEGEPGSPPRMHCGADGEWLVPVGRCSCSAGFQERGDFCEACPPGFYKVSPRRPLCS
PCPEHSRALENASTFCVCQDSYARSPTDPPSASCTRPPSAPRDLQYSLSRSPLVLRLRWL
PPADSGGRSDVTYSLLCLRCGREGPAGACEPCGPRVAFLPRQAGLRERAATLLHLRPGAR
YTVRVAALNGVSGPAAAAGTTYAQVTVSTGPGAPWEEDEIRRDRVEPQSVSLSWREPIPA
GAPGANDTEYEIRYYEKGQSEQTYSMVKTGAPTVTVTNLKPATRYVFQIRAASPGPSWEA
QSFNPSIEVQTLGEAASGSRDQSPAIVVTVVTISALLVLGSVMSVLAIWRRPCSYGKGGG
DAHDEEELYFHFKVPTRRTFLDPQSCGDLLQAVHLFAKELDAKSVTLERSLGGGRFGELC
CGCLQLPGRQELLVAVHMLRDSASDSQRLGFLAEALTLGQFDHSHIVRLEGVVTRGSTLM
IVTEYMSHGALDGFLRRHEGQLVAGQLMGLLPGLASAMKYLSEMGYVHRGLAARHVLVSS
DLVCKISGFGRGPRDRSEAVYTTMSGRSPALWAAPETLQFGHFSSASDVWSFGIIMWEVM
AFGERPYWDMSGQDVIKAVEDGFRLPPPRNCPNLLHRLMLDCWQKDPGERPRFSQIHSIL
SKMVQDPEPPKCALTTCPRPPTPLADRAFSTFPSFGSVGAWLEALDLCRYKDSFAAAGYG
SLEAVAEMTAQDLVSLGISLAEHREALLSGISALQARVLQLQGQGVQV
>Hsap_EPHB1 NP_004432.1 ephrin type-B receptor 1 precurs
or [Homo sapiens]
MALDYLLLLLLASAVAAMEETLMDTRTATAELGWTANPASGWEEVSGYDENLNTIRTYQV
CNVFEPNQNNWLLTTFINRRGAHRIYTEMRFTVRDCSSLPNVPGSCKETFNLYYYETDSV
IATKKSAFWSEAPYLKVDTIAADESFSQVDFGGRLMKVNTEVRSFGPLTRNGFYLAFQDY
GACMSLLSVRVFFKKCPSIVQNFAVFPETMTGAESTSLVIARGTCIPNAEEVDVPIKLYC
NGDGEWMVPIGRCTCKPGYEPENSVACKACPAGTFKASQEAEGCSHCPSNSRSPAEASPI
CTCRTGYYRADFDPPEVACTSVPSGPRNVISIVNETSIILEWHPPRETGGRDDVTYNIIC
KKCRADRRSCSRCDDNVEFVPRQLGLTECRVSISSLWAHTPYTFDIQAINGVSSKSPFPP
QHVSVNITTNQAAPSTVPIMHQVSATMRSITLSWPQPEQPNGIILDYEIRYYEKEHNEFN
SSMARSQTNTARIDGLRPGMVYVVQVRARTVAGYGKFSGKMCFQTLTDDDYKSELREQLP
LIAGSAAAGVVFVVSLVAISIVCSRKRAYSKEAVYSDKLQHYSTGRGSPGMKIYIDPFTY
EDPNEAVREFAKEIDVSFVKIEEVIGAGEFGEVYKGRLKLPGKREIYVAIKTLKAGYSEK
QRRDFLSEASIMGQFDHPNIIRLEGVVTKSRPVMIITEFMENGALDSFLRQNDGQFTVIQ
LVGMLRGIAAGMKYLAEMNYVHRDLAARNILVNSNLVCKVSDFGLSRYLQDDTSDPTYTS
SLGGKIPVRWTAPEAIAYRKFTSASDVWSYGIVMWEVMSFGERPYWDMSNQDVINAIEQD
YRLPPPMDCPAALHQLMLDCWQKDRNSRPRFAEIVNTLDKMIRNPASLKTVATITAVPSQ
PLLDRSIPDFTAFTTVDDWLSAIKMVQYRDSFLTAGFTSLQLVTQMTSEDLLRIGITLAG
HQKKILNSIHSMRVQISQSPTAMA
>Hsap_EPHB2 NP_004433.2 ephrin type-B receptor 2 isoform
2 precursor [Homo sapiens]
MALRRLGAALLLLPLLAAVEETLMDSTTATAELGWMVHPPSGWEEVSGYDENMNTIRTYQ
VCNVFESSQNNWLRTKFIRRRGAHRIHVEMKFSVRDCSSIPSVPGSCKETFNLYYYEADF
DSATKTFPNWMENPWVKVDTIAADESFSQVDLGGRVMKINTEVRSFGPVSRSGFYLAFQD
YGGCMSLIAVRVFYRKCPRIIQNGAIFQETLSGAESTSLVAARGSCIANAEEVDVPIKLY

CNGDGEWLVPIGRCMCKAGFEAVENGTVCRGCPSGTFKANQGDEACTHCPINSRTTSEGA
TNCVCRNGYYRADLDPLDMPCTTIPSAPQAVISSVNETSLMLEWTPPRDSGGREDLVYNI
ICKSCGSGRGACTRCGDNVQYAPRQLGLTEPRIYISDLLAHTQYTFEIQAVNGVTDQSPF
SPQFASVNITTNQAAPSAVSIMHQVSRTVDSITLSWSQPDQPNGVILDYELQYYEKELSE
YNATAIKSPTNTVTVQGLKAGAIYVFQVRARTVAGYGRYSGKMYFQTMTEAEYQTSIQEK
LPLIIGSSAAGLVFLIAVVVIAIVCNRRRGFERADSEYTDKLQHYTSGHMTPGMKIYIDP
FTYEDPNEAVREFAKEIDISCVKIEQVIGAGEFGEVCSGHLKLPGKREIFVAIKTLKSGY
TEKQRRDFLSEASIMGQFDHPNVIHLEGVVTKSTPVMIITEFMENGSLDSFLRQNDGQFT
VIQLVGMLRGIAAGMKYLADMNYVHRDLAARNILVNSNLVCKVSDFGLSRFLEDDTSDPT
YTSALGGKIPIRWTAPEAIQYRKFTSASDVWSYGIVMWEVMSYGERPYWDMTNQDVINAI
EQDYRLPPPMDCPSALHQLMLDCWQKDRNHRPKFGQIVNTLDKMIRNPNSLKAMAPLSSG
INLPLLDRTIPDYTSFNTVDEWLEAIKMGQYKESFANAGFTSFDVVSQMMMEDILRVGVT
LAGHQKKILNSIQVMRAQMNQIQSVEV
>Hsap_EPHB3   NP_004434.2 ephrin type-B receptor 3 precurs
or [Homo sapiens]
MARARPPPPPSPPPGLLPLLPPLLLLPLLLLPAGCRALEETLMDTKWVTSELAWTSHPES
GWEEVSGYDEAMNPIRTYQVCNVRESSQNNWLRTGFIWRRDVQRVYVELKFTVRDCNSIP
NIPGSCKETFNLFYYEADSDVASASSPFWMENPYVKVDTIAPDESFSRLDAGRVNTKVRS
FGPLSKAGFYLAFQDQGACMSLISVRAFYKKCASTTAGFALFPETLTGAEPTSLVIAPGT
CIPNAVEVSVPLKLYCNGDGEWMVPVGACTCATGHEPAAKESQCRPCPPGSYKAKQGEGP
CLPCPPNSRTTSPAASICTCHNNFYRADSDSADSACTTVPSPPRGVISNVNETSLILEWS
EPRDLGGRDDLLYNVICKKCHGAGGASACSRCDDNVEFVPRQLGLTERRVHISHLLAHTR
YTFEVQAVNGVSGKSPLPPRYAAVNITTNQAAPSEVPTLRLHSSSGSSLTLSWAPPERPN
GVILDYEMKYFEKSEGIASTVTSQMNSVQLDGLRPDARYVVQVRARTVAGYGQYSRPAEF
ETTSERGSGAQQLQEQLPLIVGSATAGLVFVVAVVVIAIVCLRKQRHGSDSEYTEKLQQY
IAPGMKVYIDPFTYEDPNEAVREFAKEIDVSCVKIEEVIGAGEFGEVCRGRLKQPGRREV
FVAIKTLKVGYTERQRRDFLSEASIMGQFDHPNIIRLEGVVTKSRPVMILTEFMENCALD
SFLRLNDGQFTVIQLVGMLRGIAAGMKYLSEMNYVHRDLAARNILVNSNLVCKVSDFGLS
RFLEDDPSDPTYTSSLGGKIPIRWTAPEAIAYRKFTSASDVWSYGIVMWEVMSYGERPYW
DMSNQDVINAVEQDYRLPPPMDCPTALHQLMLDCWVRDRNLRPKFSQIVNTLDKLIRNAA
SLKVIASAQSGMSQPLLDRTVPDYTTFTTVGDWLDAIKMGRYKESFVSAGFASFDLVAQM
TAEDLLRIGVTLAGHQKKILSSIQDMRLQMNQTLPVQV
>Hsap_EPHB4   NP_004435.3 ephrin type-B receptor 4 precurs
or [Homo sapiens]
MELRVLLCWASLAAALEETLLNTKLETADLKWVTFPQVDGQWEELSGLDEEQHSVRTYEV
CDVQRAPGQAHWLRTGWVPRRGAVHVYATLRFTMLECLSLPRAGRSCKETFTVFYYESDA
DTATALTPAWMENPYIKVDTVAAEHLTRKRPGAEATGKVNVKTLRLGPLSKAGFYLAFQD
QGACMALLSLHLFYKKCAQLTVNLTRFPETVPRELVVPVAGSCVVDAVPAPGPSPSLYCR
EDGQWAEQPVTGCSCAPGFEAAEGNTKCRACAQGTFKPLSGEGSCQPCPANSHSNTIGSA
VCQCRVGYFRARTDPRGAPCTTPPSAPRSVVSRLNGSSLHLEWSAPLESGGREDLTYALR
CRECRPGGSCAPCGGDLTFDPGPRDLVEPWVVVRGLRPDFTYTFEVTALNGVSSLATGPV
PFEPVNVTTDREVPPAVSDIRVTRSSPSSLSLAWAVPRAPSGAVLDYEVKYHEKGAEGPS
SVRFLKTSENRAELRGLKRGASYLVQVRARSEAGYGPFGQEHHSQTQLDESEGWREQLAL
IAGTAVVGVVLVLVVIVVAVLCLRKQSNGREAEYSDKHGQYLIGHGTKVYIDPFTYEDPN
EAVREFAKEIDVSYVKIEEVIGAGEFGEVCRGRLKAPGKKESCVAIKTLKGGYTERQRRE
FLSEASIMGQFEHPNIIRLEGVVTNSMPVMILTEFMENGALDSFLRLNDGQFTVIQLVGM
LRGIASGMRYLAEMSYVHRDLAARNILVNSNLVCKVSDFGLSRFLEENSSDPTYTSSLGG
KIPIRWTAPEAIAFRKFTSASDAWSYGIVMWEVMSFGERPYWDMSNQDVINAIEQDYRLP
PPPDCPTSLHQLMLDCWQKDRNARPRFPQVVSALDKMIRNPASLKIVARENGGASHPLLD
QRQPHYSAFGSVGEWLRAIKMGRYEESFAAAGFGSFELVSQISAEDLLRIGVTLAGHQKK
ILASVQHMKSQAKPGTPGGTGGPAPQY
>Hsap_EPHB6   EAW51900.1 EPH receptor B6, isoform CRA_a [H
omo sapiens]

MATEGAAQLGNRVAGMVCSLWVLLLVSSVLALEEVLLDTTGETSEIGWLTYPPGGWDEVS
VLDDQRRLTRTFEACHVAGAPPGTGQDNWLQTHFVERRGAQRAHIRLHFSVRACSSLGVS
GGTCRETFTLYYRQAEEPDSPDSVSSWHLKRWTKVDTIAADESFPSSSSSSSSSSAAWA
VGPHGAGQRAGLQLNVKERSFGPLTQRGFYVAFQDTGACLALVAVRLFSYTCPAVLRSFA
SFPETQASGAGGASLVAAVGTCVAHAEPEEDGVGGQAGGSPPRLHCNGEGKWMVAVGGCR
CQPGYQPARGDKACQACPRGLYKSSAGNAPCSPCPARSHAPNPAAPVCPCLEGFYRASSD
PPEAPCTGPPSAPQELWFEVQGSALMLHWRLPRELGGRGDLLFNVVCKECEGRQEPASGG
GGTCHRCRDEVHFDPRQRGLTESRVLVGGLRAHVPYILEVQAVNGVSELSPDPPQAAAIN
VSTSHEVPSAVPVVHQVSRASNSITVSWPQPDQTNGNILDYQLRYYDQAEDESHSFTLTS
ETNTATVTQLSPGHIYGFQVRARTAAGHGPYGGKVYFQTLPQGELSSQLPERLSLVIGSI
LGALAFLLLAAITVLAVVFQRKRRGTGYTEQLQQYSSPGLGVKYYIDPSTYEDPCQAIRE
LAREVDPAYIKIEEVIGTGSFGEVRQGRLQPRGRREQTVAIQALWAGGAESLQMTFLGRA
AVLGQFQHPNILRLEGVVTKSRPLMVLTEFMELGPLDSFLRQREGQFSSLQLVAMQRGVA
AAMQYLSSFAFVHRSLSAHSVLVNSHLVCKVARLGHSPQGPSCLLRWAAPEVIAHGKHTT
SSDVWSFGILMWEVMSYGERPYWDMSEQEVLNAIEQEFRLPPPPGCPPGLHLLMLDTWQK
DRARRPHFDQLVAAFDKMIRKPDTLQAGGDPGERPSQALLTPVALDFPCLDSPQAWLSAI
GLECYQDNFSKFGLCTFSDVAQLSLEDLPALGITLAGHQKKLLHHIQLLQQHLRQQGSVE
V
>Cint_Epha	BAE06400.1 ephrin receptor [Ciona intestinal
is]
MDYLTVLIPLIIGLTNLQFISSEEVTILDTTQATSDLGWTASGTGWTELTNHDETDADLR
TFQVCHIRSPHQNNWLKTNFINVTTTQRVYVEIKFTIRSCDGIRGVVSCKETFNLYYLES
NHMNDRRSLRLNEDTYTKVDTIAADERFKPDTRVEANTETRMISPIRSRGLYIAFQDTGA
CMSIMHVRVYYKSCQETLRGLAIFPPTVSGPMMTSLVDVPGQCVPNSATMKNTKAPSLHC
NGEGEWLVPTGSCSCNPGYEPNNQLTECNQCRVGRFKHEVGNVPCQPCPARSFNVNFAST
MCMCISGYYRSPGDRPDAPCTRTPSKPSTLESSVNKTSVKLTWVEPRKNGGRTDITYSVT
CERCDATYTLCSRCGEGADFIPGRTRLTKGSLLVRDLAPHTDYKFKVYSFNGVSGVSGLR
PAFAEIRIRTNQAAPSAVQPVRVVMATSAEVLLQWSPPRYSSNPILDYQIQYSSIHGLFT
NVTTKTTYKLSNLSPSTSYIVQVRARSKVGYGLYSRASAFRTKPVKSSTAHYVLTNPHGA
SVDIDGGNDNMVHDTTVKPSSNHNRLVVAVCVACSFLVITLCALLILCGRRNRYAKAVES
DLEKYPGFINSSVDLLSTRTYVDPSTYEDPYNAVKEFAKEIDFKLVNIEQVIGRGEFGEV
CKGTFFNKCVAIKTLKVGYNTQERSDFLGEASIMGQFDHPNVIRLEGVVTKSRPLMIITE
FMENGSLDAFLRNQEGKIPSAQLTEMLLSIAQGMEYLSNMGYVHRDLAARNILVNSNLVC
KVSDFGLSRVLEQLEEDDVAMYTTRGGMIPIRWTSPEAITMRTFSTSSDVWSFGIVMWEV
MSFGERPYWEMSNQDVVQFVDSGYRLPPPCYCPRIIHSVMLECWKRDRTQRPNFSHLVTL
LDRFLLEADCLTDTINNRPFRDQDDCIPEMLDLSSLGQWLEQMHMLKYKPTMMRHGCVTA
EQIIRLTASGLAHMGFSDMNDVNIVLREADLLRQKIEEVGNVGVAV
>Cint_Ephb	BAE06403.1 ephrin receptor [Ciona intestinal
is]
MEQKYTRCFLCNKMIWIIIITGFLAERHVIAEEAILMDTRTAISDIGWAMSPGSDWTEAN
GPHPDGRDRRMLVVCQIKKPHQNNWARTPYIPVDGAERIYVEFEFTIRSCEDVPNVATCK
ETFNLYYYETDRDEATSTFPPWREGAYIKVDTIAADKRFKPGSAERNFETRDIGPLTKRG
VYLAIQDTGACMALMHVRVYYKYCAPTESNLANFGRTTAGPYMASLVEANGTCVDNSLAD
NDDRATFRCNSEGEWIVPKGSCLCDRGYGPDYTSTSCQECGRGFYKSSVGNGGCVRCPAY
SESQSDGSTECQCMPGYYRALSDDAAKPCSQPPSKPVNVLAIVNQTKITLTWERPFNDGG
RDDITYRIECQRCQVGFDHCAPCPQGVDFEPRQRDLVVTKVVVRDLSSYAYYRFKIFAIN
GVTWVAQLSPSEVEYAEVTVETNEAAPSMVLGVRVTDLSETSAILTWDTPAKVNGLIRSY
VVSLGLVYQGQVTDMRMFNSTSKRSMRLDGLKQNNMYAVKVRASTDAGFGPYSVPLKFSP
LESTTSSPNTNDHVGTIQLIIAVIGGCSIVIAIVLIVFCCRRKSHQNKSKLDMEKLPMFV
MGHLKNGSIGYMPGHKTYIDPTTYEDPARAVLDFTKEIDASYIGIEEVIGGGEFGEVCLG
RMRLPNSKKEHDVAIKTLKAGYSTQQKLDFLGEASIMGQFDHPNVIRLEGVVTKSQPHMI
ITEYMENGSLDTFLRKHDGEFTVIQLVGILRNIAAGMKYLSDMGYVHRDLAARNILVNAQ
LICKVSDFGLSRVLEESSDSTYTTRGGKIPIRWTAPEAINYRKFTSASDVWSYGIVMWEV

MSYGERPYWGMSNQDVIHAVGTGYRLPAPMDCPQVQHELMLDCWKKDRNERPKFSQVVAT
LDRMIREPEQLKVIAGHDKNRNGCLLNERGSLRRPSFQGPNGISETMTLLMDDRTRCTSL
LRHQVPSSLGVWLDGLGLGNYKENFVSAGYSSLDQVLQMSPSDLASLGIVETNQQNAVME
SISSIRYKTNPGRKPPQYRDERITGIPV
>Cint_Ephc NP_001071691.1 ephrin receptor precursor [Ci
ona intestinalis]
MFFLFIVFYCISIVTGEIHVLYNTKVATSDLDWALHPTYGAWEELSGLDVDGNTIRYHQV
CNTGMDEQDNWVRSPFIDAKSAQRIYMDIEFSVMKCEECRETFALYYYPSSSDTATTTFP
PWRENPYIKIDTLAAGERFDSETVGAEGINKKTLVIGPLSRRGFYIAAQDQGACMSIMSL
KLYYYHCEETTHNLAYFPNTISGGGIAELVSQSGKCVSNSVYANEVPKYRCNIYGEWQVP
TGSCQCRAGYEPNTQLTACTGCMVGKYKSTNGNTPCQVCPQHSVTHSTSASHCTCVAGHY
RAENDPISQACTRPPTKPRNVTHVQNKTSLLLSWVPPSTTGGRTDIYYSISCELCDSEHE
NCQPCNVDVQYRPSNHQHTTTTYMEVSNLNPFSCYKFKVSSSNGVSRVSVEPEQFELIKI
CTNAAAPSAVTGLKFIWIGEVSATLSWLPPTHSNKIVGYEVQLFQNNQRLKLTKIMEVST
PNVTINGLNPGWKYVVMVRACNNDGCGQFSEKLQLVTYDKGSEPESIVEGSTTWIGGVIG
GVLVIVIIIIVVMIKRRQTDKKKRKEIQARTKLNENTQQLNQSSFLQTAGRTYVDYRDPH
NGVKEIATEIDQTRIKIDSVIGRGEFGEVCRGKMLTGKTTTSVAVKRLKHGASLIDHTNF
LREACTMAQFKDPNIIQLKGVVTKSIPAMIITEFMEHGSLDKFLQARSGQPTVLQLLEML
RGIASGMKYLSSMKYVHRDLAARNILVNSQLVCKVSDFGLSRTLENDPQATYTTQGGKIA
LRWTAPESIRCRQFTSASDVWSYGIVMWEVMSYGEKPYWDMSNEVVTEVLEDGYRLPSPE
GCPTPVHSLMLKCWSYEPKRRPTLLEIIKTLDHFIKQPSSLQDDMEADASAPLLKPDSPN
SIQDVSTLDEWLDMVKLGRYRRSFHNNGINDLESLAHISESELDRLGIASPSHRTRLQGG
INTLRQHLVEVNEVQSSSNAPYDAHASTLPIAHGKPSNPVAV
>Cint_Ephd BAE06403.1 ephrin receptor [Ciona intestinal
is]
MEQKYTRCFLCNKMIWIIIITGFLAERHVIAEEAILMDTRTAISDIGWAMSPGSDWTEAN
GPHPDGRDRRMLVVCQIKKPHQNNWARTPYIPVDGAERIYVEFEFTIRSCEDVPNVATCK
ETFNLYYYETDRDEATSTFPPWREGAYIKVDTIAADKRFKPGSAERNFETRDIGPLTKRG
VYLAIQDTGACMALMHVRVYYKYCAPTESNLANFGRTTAGPYMASLVEANGTCVDNSLAD
NDDRATFRCNSEGEWIVPKGSCLCDRGYGPDYTSTSCQECGRGFYKSSVGNGGCVRCPAY
SESQSDGSTECQCMPGYYRALSDDAAKPCSQPPSKPVNVLAIVNQTKITLTWERPFNDGG
RDDITYRIECQRCQVGFDHCAPCPQGVDFEPRQRDLVVTKVVVRDLSSYAYYRFKIFAIN
GVTWVAQLSPSEVEYAEVTVETNEAAPSMVLGVRVTDLSETSAILTWDTPAKVNGLIRSY
VVSLGLVYQGQVTDMRMFNSTSKRSMRLDGLKQNNMYAVKVRASTDAGFGPYSVPLKFSP
LESTTSSPNTNDHVGTIQLIIAVIGGCSIVIAIVLIVFCCRRKSHQNKSKLDMEKLPMFV
MGHLKNGSIGYMPGHKTYIDPTTYEDPARAVLDFTKEIDASYIGIEEVIGGGEFGEVCLG
RMRLPNSKKEHDVAIKTLKAGYSTQQKLDFLGEASIMGQFDHPNVIRLEGVVTKSQPHMI
ITEYMENGSLDTFLRKHDGEFTVIQLVGILRNIAAGMKYLSDMGYVHRDLAARNILVNAQ
LICKVSDFGLSRVLEESSDSTYTTRGGKIPIRWTAPEAINYRKFTSASDVWSYGIVMWEV
MSYGERPYWGMSNQDVIHAVGTGYRLPAPMDCPQVQHELMLDCWKKDRNERPKFSQVVAT
LDRMIREPEQLKVIAGHDKNRNGCLLNERGSLRRPSFQGPNGISETMTLLMDDRTRCTSL
LRHQVPSSLGVWLDGLGLGNYKENFVSAGYSSLDQVLQMSPSDLASLGIVETNQQNAVME
SISSIRYKTNPGRKPPQYRDERITGIPV
>Cint_Ephe NP_001071691.1 ephrin receptor precursor [Ci
ona intestinalis]
MFFLFIVFYCISIVTGEIHVLYNTKVATSDLDWALHPTYGAWEELSGLDVDGNTIRYHQV
CNTGMDEQDNWVRSPFIDAKSAQRIYMDIEFSVMKCEECRETFALYYYPSSSDTATTTFP
PWRENPYIKIDTLAAGERFDSETVGAEGINKKTLVIGPLSRRGFYIAAQDQGACMSIMSL
KLYYYHCEETTHNLAYFPNTISGGGIAELVSQSGKCVSNSVYANEVPKYRCNIYGEWQVP
TGSCQCRAGYEPNTQLTACTGCMVGKYKSTNGNTPCQVCPQHSVTHSTSASHCTCVAGHY
RAENDPISQACTRPPTKPRNVTHVQNKTSLLLSWVPPSTTGGRTDIYYSISCELCDSEHE
NCQPCNVDVQYRPSNHQHTTTTYMEVSNLNPFSCYKFKVSSSNGVSRVSVEPEQFELIKI
CTNAAAPSAVTGLKFIWIGEVSATLSWLPPTHSNKIVGYEVQLFQNNQRLKLTKIMEVST

PNVTINGLNPGWKYVVMVRACNNDGCGQFSEKLQLVTYDKGSEPESIVEGSTTWIGGVIG
GVLVIVIIIIVVMIKRRQTDKKKRKEIQARTKLNENTQQLNQSSFLQTAGRTYVDYRDPH
NGVKEIATEIDQTRIKIDSVIGRGEFGEVCRGKMLTGKTTTSVAVKRLKHGASLIDHTNF
LREACTMAQFKDPNIIQLKGVVTKSIPAMIITEFMEHGSLDKFLQARSGQPTVLQLLEML
RGIASGMKYLSSMKYVHRDLAARNILVNSQLVCKVSDFGLSRTLENDPQATYTTQGGKIA
LRWTAPESIRCRQFTSASDVWSYGIVMWEVMSYGEKPYWDMSNEVVTEVLEDGYRLPSPE
GCPTPVHSLMLKCWSYEPKRRPTLLEIIKTLDHFIKQPSSLQDDMEADASAPLLKPDSPN
SIQDVSTLDEWLDMVKLGRYRRSFHNNGINDLESLAHISESELDRLGIASPSHRTRLQGG
INTLRQHLVEVNEVQSSSNAPYDAHASTLPIAHGKPSNPVAV
>Cint_Ephf NP_001071692.1 ephrin receptor like protein
precursor [Ciona intestinalis]
MVIPAKMFPVSSLLILFANFVHFVTSYQVEILNTRNATSDLIGWNMTTYVNNIEDFSPEV
KWDEVTFHQLNPSVRGFQICPDSQDTQSYKTNVQNWLRSPYMKLIGRSRLYVELNFWMRE
CENKDTICPETFKIYYAITSHQSEVPQLNESVFTKIDTVAAEDQFADSNSDWTEVLNTEI
VNIEIPQSIDISHAGVYVAIVDKGACIALVSVRVYSIVCPMEIQSLAIFPETLTGELDTS
LVKVNGSCVERSHPANSEVSSQPVNHCTSDGRWNVRTGACYCEAGYQPDHNMRTCQPCPR
NMYKPTSGNLNCTKCPSVSYTHHVGSISCVCSDGHLVSTSYLDGCGVCPQLPSPPLKLKV
IKSTTVPGAARLEWEKPQDFGNCQKIFYCVLCQVHSINHASTSLSKQNNNSSLFKQSDPT
QPSYTVDACDGVLYYPHQCSLKTNFVDMHNLTGHGLYTFQVVATNAVTHKHADSITGQDA
VRNKMLNPKVSNTSSAFVTFSIDKFHPSTPTNLHMIHSNYTSVTIQWNPPVYTSGDQISY
KIVWVENQARNKTAILQLKSTTLPTSHMLVNVTRAHLAQLNSGVNYYIAVQALSTAGQSN
YSSVLEVRTMSSDLTSKPTGKPNNSNDSLYVYLVAVVSVLLFGGFVFFFLWRRCRGTSGK
QKSKPCLDKPLQSSSEECDGFTVCTELSDQRTYVDPFSCGPIQTHKFSCETDPDLVSINK
VIGCGEFGEVYQGSLTLTDTGGKRKVIDVAIKQLHAQCQPNEQIDFLHEAKALERFQHPN
IVKLEAVVMASRPFMIVTELMVNGCLGKFLRHHRGAKRYFKPTVLVRMLHGVAKGMAYLS
KMCYVHRDLAARNVLINNELVCKVADFGLIRHIGEKKSTVNTKGGKISLRWTAPEAVAYQ
AYSQASDVWSFGVFAWEVITYGEKPYWSLTNQQVLQAINDGYRLPAPEDCPSVLHQLMLE
CWHRDKNTRPTFEQVVTRLDAMINQRGVLDTLASPASTGEDVLFFTDSPPHTPCPVPTVT
DGQSHHNYATCRIADNDSPLGNLIRGETPDPAEVTEPYVESNDLVDGSPSDDLLSHHSSY
EVSTM
>Bflo_Eph1
MAAGRTAQTSAFALWTIFVWIFTVEAAESGGALPSRSQRAASRLRSNPGNDEDSCPLGLR
NNSVPGNDENFCLPGWRSNSLLCQVNKMSLKTVTDRITWIEVSNLSPDTGHPVRTYQVCQ
VQREDQNNWLRTEWIPKKEGQRIYVEIRFSIRECRDIPNVVSCKETFDFYYFESDQDDAT
DEYPAWNESAYTKVDRIAADGRFSNVNVQEINKEIRNIGPITKDGFYLAFQDSGACMSLL
SVRVYYKVCPTVAHSLATFNKTVTGPEVTSLVQAAGSCVPNAEVEAQPTVQCTSDGKWTL
FAGGCRCLPGHEPESDGCSGGSEGCGKAARKIGPEINRTDEWDAPSQVEGVTQIGSTEMS
LSISWSPPRKPNGVILEYRVQFYPLRDGPTQTKFAKVDGQVTQATVEHLDMGEEYVLQVQ
ARTIAGYGQLSQPVTMRTDANGSQAPMTGDQPVMIAAIAAAGIFVLLVFVLVVIFVLYRR
RNRKPPSDMDKLEYSNGEVRLPGFVAPRVKTYIDPHTYEDPQHAVLEFAKELEPSCLTIE
QVVSPGEFGDVCKGRLKMGKGQYLDVAIKTLKAGSNDKMKGDFLTEASIMGQFTDPNVIK
LQGVITKSRPVMIVTEYMENGSLDTFLRKHDGRFQVPQLVGMIRGVASGMRYLAEMNYVH
RDLAARNVLVNSSLVCKVSDFGLSRVLEDDPDAAYTTQGGKIPVRWTAPEAIAYRKFTSA
SDVWSYGVLVWEVMSYGERPYWNWSNQDVIKSVEAGYRLPPPMDCPKAIYQLMSDCWQKE
RNARPKFTAIVASLDKLIRNPMVLKVLAQPRYLPEPMHHHSTSSDLYSSVPEWLDSLSLG
RYTENFLKAGFTNLDCVAKITLRELQSIGVTMIGHQKKIMNSAQMLRAQASPNANLRAPS
PIANGQPLPHMHGGGVSSLHGTPNMHHHNTRHITHALPV
>Bflo_Eph2
SQWIEVSNINSDGHPVRTYQVCQVRREDQNNWLRAEWIPKRQGRRIYVEIKFSIRECRDI
PNVVSCKETFDLYYFESDYPDATDDRPAWNDRTYTKVDRIAADGRFSHVNTEIINTEVRD
IGPIFKRGFYLAFQDSGACISLLSVRVYYKVCPTITEHYAIFNKTVTGPSITSLVQAEGQ
CIPNSEPVGRQPTVQCTGTGEWTLFTGSCRCLPGYEPQRTVAQRCDGKWRSFLAQGACKE
GTFKPNAGNYECMPCPLYSHAHRTGETRCTCDRGYYRSPRDKPTDACTGRFMPISSAPPS

KPRNIISTVNMTTVELQWETPVDNGGRNDLRYKIVCWRCHDITCVECRRAVQYRPAQVGL
NNTVVTIEGLMAYTKYRFEVHAENGVSSTSRVKDRFESITLKTNVTAPSQIQSIIQAAST
ETAMTIEWSVPAQPNGKILEYRIQFYRARDGPGMTAIIKVDGDVQSATVENLENSEEFAS
QVQARTIAGYGKLSEPITLRTSYTVAFSHTGAQAPLGSDQPVLIAAIAAAGGFVLVLSLF
ILVCVQHLQCHINIAIPVRLPGFVAPRVKTYIDPHTYEDPQHAVREFARELEPSCLTIEQ
VIGGGEFGDVCKGRLKVGKGQYLDVAIKTLKPGSTDKMKDDFLTEASIMGQFNDPNIIKL
QGVITKSQLTKHVFYIFPCLFQKHDGKFQVTQLVGMIRGVASGMRYLAEMNYVHQDLAAR
NVLINSQLVCKVSDFGLSRVLGDDPDAAYTTNVSHGGKIPIRWTSPEAIAFRKFTSASDV
WSYGVLVWEVMSYGERPYWNWSNQDVIKSVEAGYRLPPPMDCPQAIYQLMTDCWERERNA
RPEFLLVTYNGDGFQPKADCLLLYVRFRPMQPSFQPVLPTVPQWLDSMKMGRYRDNFTNG
GYSSMDLVMKMNIRDLQGIGVTMLAHQKKILNSIQNLHTQLSDCWP

Eph receptors identified in this study from our searches

Eph receptors in bilateria

>Drer_3161.9 D. rerio
MALMAGIIYALTSLFLGICAGLSAPRSPPANEVTLLDSRSVQGELAWVASPTEGGWEEVS
IMDEKNIPIRTYQVCNVMEPSQNNWLRTHWIQRGAAQRIYIEIKFTLRDCNSLPGVIGTC
KETFNLYYLESDSDNERYAHESRFSKIDTVAADESFTQVDIGDRIMKLNTEVRDVGVLSR
AGFYLAFQDVGACIALVSVHVFYKKCPLAVRNLAQFPDTVTGADTSSLVEVRGSCVDHSE
EQEVPKMYCGADGEWLVPIGNCLCNPGYEERIEQCQVCKVGYYRSLSSDVGCRKCPLHSY
SVKEGATSCDCDKGYYRSETDPAAMPCTRPPSAPHNLISNVNETSVLLDWSPPLSSGGRQ
DLTYNVVCKQCVRDTQRCSPCGDDVRYSPQRLSLRSTRVSVHQLQAHTNYTFQIWAINGV
SKHNPSPEQAVSVTLTTNQAAPSMVMSVQSKDITRHTLALFWDQPEKPNGVILEYEVKYY
EKDQNERSYRIVKTTSRNADIKDLTPLTSYVFHVRARTAAGYGDFSAPFEFSTNTVAAPV
VGGVMSSAVLLLLVAGCVVVLLLILIITFIFTKRRSKYSKTKQGEDKNVQQGVRIYVDPF
TYEDPNQAIREFAKEINTSCIKIEKVIGIGEFGEVCSGRLKLPGKREICVAIKTLKAGFT
EQQRLDFLSEASVIGQFDHPNIIHLEGVVTKCKPVMIITEYMENGSLDMFLRKNDGRFTV
IQLVGILRGIASGMKYLSDMSYVHRDLAARNILVNSNLVCKVSDFGMSRVLEDEPEGAYT
TRGGKIPIRWTAPEAITYRKFTSASDVWSYGIVMWEVMSYGERPYWDMSNQDVIKAIEEG
YRLPPPMECPLALHQLMLECWMRERADRPKFSQIVNMLDKLIRNPATLKRTAGDASRSHP
STINHAPSECSAPSLASVDDWLKLIGLEQYRENFNTAGYSSIESVIPMNHEDLAKMGISC
SAHQRKILSSVEDLLSGMHHRQDSKVPV
>Drer_11069.6 D. rerio
MDYRLTRVSFLFLYLFANVFTGIQSKEHVLLDMKAAGSELGWLTWPKEEGWEVVQTVVNG
SLLYTYSVCNVASDTEQDNWLRTTFIQRRPDVSRMSVELSFIVRDCSTFGGSSPSCRETF
GLFLGETDSDVGTNFRKSQFRKVATVAPDEVTADRGRGRADLRVNVEKRSIGTLSRRGFY
LAFQDLGGCVALLGLRVFYTTCPATMRSLAAFPEQVALGAEVEGTCVKDAVVLGEAAPRM
YCTAEGDWVVPVGQCVCRAGYQAVGDSCKACEPGFYKSTDSSQPCEVCPENTQRSGRGAL
LCPCMEGFYRAPTDLNSAPCSSLPSPPRDLVYTPLLTAGSLQLLWRPPADTGGRSDVTYS
VACERCEGGLCTLCGGRVRFEPAQTALRAPEVVVSELEPHVNYTFTVEAQNGVSQFSRKR
AMASITTVLHFTDGPRVLYLRVEDRTTSSLTLSWAVDHHVQNQPSPRYELMYRKKEDPGE
LDVTTYTVLVLEKNSVPINDLLPGTKYVFRVHTLTAEGHPSSHSAELEFETLPLAESRTQ
NSSMVVMGAIAGGGVMLLIVVVILLLHKRRLNSHVRRRVDGDYFSCPEKLLPLKTYIDPH
TYEDPCAAILKFASEIHPGHITKQKVIGAGEFGEVFRGSLKMPGRSEVAVAIKTLKPGYT
EKQRQDFLSEASIMGQFSHKNIIRLEGVVTKFKDAMIITEYMENGALDQYLRDHDGDFSS
YQLVGMLNGIAAGMKYLSDMNYVHRDLAARNVLVNSNLECKVSDFGLSRVLEDFPEGTYT
TTGGKIPIRWTAPEAIAYRKFTSASDVWSFGIVMWEVMSFGERPYWDMSNQEVMKSINDG
YRLPAPMGCPSAVNQLMLQCWMQDRSTRPRFVDIVNLLEKLLRNPESLTSIASIDPRVSI
RLPSTSGCDGAPFRSVDEWLESIKMAQYRETFALAGIKSFDQVLRLKIEDIGNIGVRLAG
HQKRIAYSILGLQDPVGALDLFAM

>Drer_21706.11 D. rerio
MDCKTLILFLFFFTLISSADFIIHPSNEVNLLDSKASQGELGWISSPSHGWEEISGVDEH
YTPIRTFQVCNVMEYSQNNWLRTNWIPRNSAQKIYVELKFTLRDCNSIPMVLTCKETFNL
HYLETEEDQGNKFREHQFSKIDTIAADESFTQMDLGDRILKLNTEVREVGPMTKKGFYLA
FQDVGACVALVSVRVYFKKCPFTVRNLAMFPDTVPTDSQSLVEVRGSCVNNSKEEDPPKM
YCSTEGEWLVPIGKCLCNIGYEERGVACQACRPGFYKASSGNVKCSKCPPHSYTHQEGSV
HCGCEKNYFRADEDPASMACSRPPSAPRNVISAINETSVILDWNWPADNGGRKDVIFNVV
CKRCWPGPRQCEPCRGAVRFLPRAFGLTNTTVTVADLLAHTNYTFEIEAQNGVSFQAPTI
RQYAAVTITTNQAAPSAVTVIRKDRTSRNSISLSWMEPERPNGIILDYEVKYYEKQEQET
SYTILRSKGTNVTLNGLKPDTTYLLQIRARTAAGYGSSSRKFEFETSPDSFSISSENSQV
VLIAISSAVAIILLTVVLYIVISRFCRYSKSKHPDEKRLPFGNGHLRLPGIKTYVDPHTY
EDPSQAVHEFAKELDASSIAIDKVVGAGEFGEVCSGRLKLPSKKEICVAIKTLKAGYTEK
QRRDFLSEASIMGQFDHPNIIRLEGVVTRSKPVMIVTEYMENGSLDGFLRKHDAQFTVIQ
LVGMLRGIASGMKYLSDMGYVHRDLAARNILVNSNLVCKVSDFGLSRVLEDDPEAAYTTR
GGKIPIRWTAPEAIAYRKFTSASDVWSYGIVLWEVMSYGERPYWEMSNQDVIKAVDEGYR
LPPPMECPATLYQLMLDCWQKDRNNRPKFEQIVSILDKLIRNPSSLKITASTASRPVNLL
LDRNSTDITSFHTMGDWLESARTLPCKDAFSGVSYSSCDTLAKTSAEDFKKVGVTIVGPQ
KKIVSSLTTLETHSKNGPVPV
>Drer_30606.8 D. rerio
MATLWIYPSLLFFLFPSLTPSQALRNYPENEVTLLDTMSALGDLGWEAYPAEGWEEISVM
DERNTPMRSYQVCNVMEASQNNWLRTRHIAREGAQRVYIEIKFTLRDCNSLPGVPGTCKE
TFNMYYYESNNPNLWFIKESQYIKIDTIAADESFTQTDVGDRVMKLNTEIRDINNLSKKG
FYLAFQDVGACIALVSLRVFYKKCPLAVLNLAQFPDTVTGGDSALVEVRGSCVNDSEEFE
APRMYCSGDGGWLVPIGRCVCKPGFEENKDYCQACRPGTYKASSMDAYCSKCPPHSFSHQ
EKASECSCEKGFYRADMDPRSMACTRPPSAPENPISTLNDTSVTLEWSPPRDSGGRGDVT
YSIHCRKCAGDGRICSPCSNNVHFVPRQFGLSIPKVLITDLLSNTNYTFSVEAVNGVSDL
SPTPKQLVTVNITTSQTVNVILKERKSKDSITLAWQGPDRPNGVIVEYEVIYYEKNQRDQ
NYTVLKTKGHMMTVEGLKPGTTYVFRVRARTDGGYGNYGGEIELETSHEDMLAIGDPNQS
TILAVSIAGGIVLLIFLVACFVVSGRHCGYIKAKQDPDEEKMQFQNGRVKLPGSRTYIHP
HTYEDPNQAVRDFAKEIDASTIRIERVIGAGEFGEVCSGRLKLPSKREIYVAIKSLKAGY
SEKQRRDFLSEASIMGQFDHPNIIRLEGVVTRCKPVMIITEYMENGSLDTFLKKHDGQFT
VIQLVGMMRGIASGMKYLSDMSYVHRDLAARNILVNSNLVCKVSDFGLSRVLEDDPEAAY
TTRGGKIPIRWTAPEAIAYRKFTSASDVWSYGIVMWEVISYGERPYWEMSNQDVIKAIDE
GYRLPAPMDCPVVLHQLMLDCWEKSRSERPKFGQIVNTLDKLIRNPNSLKELANSSVWED
PTTPEFSVSTVDEWLDAINMGQYKDNFANAGYVSLDSILYISMCELSKMDVTLVGHQKKI
LSAVQSLHAQGTHVQV
>Drer_40208.7 D. rerio
MTMDYSLLLYSLWVPVILAVEETLMDSRWATTELAWTTYPESGWEEVSGYDDHLSPIRTY
QVCNVLEPNQNNWLRTDFIPRRGVLRVYVELKFSVRDCGSIPNIPGSCKETFNLFYYESD
GDMATASSPPWRENPYVKVDTIAPDESFSLLESGIVNTKVRSFGPLSKAGFYLAFQDLGA
CMSLISARVFFKKCSTSIANFAVFPETATGAEATSLVIAAGACVPNAAEESVPLKLYCNG
DGEWMVPVGACTCMPGFEPAKKDTQCQACTPGTFKSKQGEGSCMPCPSNSRASSRASSVC
PCQSGYYRADSDLPDTACTTVPSAPLNVISSVNETSVSLEWSEPRDSGGRGDVVYNVVCK
KCLHDGGSCARCDDNVEVSPRRLGLAERQAAVRNLQAHTHYSFEIQAVNGVSPKSPSSPQ
YTTVNITTNQAAPSAVPTVHLMRATAGTLSLSWLPPEQPNGVILDYEIKYQERGESFSHT
VTAQHTSAKVEGLKAGTVYSVQVRARTVAGYGRYSNPVDFSTSLYDDPERSVQDLLPLIV
GSASAGFVVILAMIVIAVVCLRRQRTGSELEYTEKLQQYVSPGVKVYIDPFTYEDPNEAV
HEFAREIDISCVKIEEVIGAGEFGEVCRGRLKQAGRKETTVAIKTLKAGYTEHQRRDFLS
EASIMGQFDHPNVIHLEGVLTRSCPVLIVTEFMENGALDSFLRLNDGRFTVTQLVGMLRG
IAAGMKYLSDMNYVHRDLAARNVLVNSNLVCKVSDFGLSRFLDDNSSDPTYTSSLGGKIP
IRWTAPEAIAFRKFTSASDVWSYGIVMWEVMSFGERPYWDMSNQDVMNAVEQDYRLPPPM
DCPAVLHQLMLECWVKERNMRPRFGQIVSTLDKLLRNAASLKVLTSTHSGDLCRIGGTLP
GHQRKSIGDAQDIKQQMSQTLPIRV

>Drer_41799.6 D. rerio
MASDPGLLPLLVWTISMQVLGSRGTSNNEVNLLDTTKISGDWGWLTYPSHGWDAINEMDE
YFSPIHTYQVCNVMSPSQNNWLRTNWIQRDGARRIYIEMKFTLRDCNSMPGVLGTCKETF
NLYYYETDRDVGTSIWESQFSKIDTIAADESFTNVDLGVRRLKLNTEIRGVGPLGKRGFY
LAFQDIGACIAIVSVRVYYKRCTGMARNLAIFTDVVTGADSSSLVEVRGQCVDHAEERDT
PKMYCSAEGEWLVPIGRCVCSAGFEEHRDNCIACEVGFYKPVAGDQQCGKCPLHSHSETR
AALSCPCDANFYRSSTDPPSSPCTRPPSAPVNIISSVNGTSVSLEWVSPLDTGGRSDVIY
DVLCQKCDKDGLQCEDCRVGVGGGNGVVNSNVNGASGDTSGAAGGTVGALNGLVVARSGA
ALVRFIPQRTGLTEPWVTVLNLAAHANYTFRIMALNGVTHLSNEQPQSVMVNITTNQAAP
SEVMAVRQENASQNSVTLFWHEPAQPNGVILEYDIKYYEKDNEQQSYATLKSKNTSAKVS
GLKPGTSYIFQIRARTSAGCGRFSQSVEIQTGKAPPLRNTNVTIIWICMASVTGLVTFLA
VVICRKRQEERCGYSKAFQDSDEEKMHYQNGHVSFPEARFYIDPHTYEDPCQAVHEFARE
IEASRIKIEKIIGSGEFGEVCYGRMKLPGKRDVPVALKTLKAGYTEKQRRDFLAEASIMA
QFDHPNVIHLEGVVTRSKPVMIITEYMENGSLDSFLRRHDGQFTIIQLVGILRGIAAGMT
YLADLGYVHRDLAARNVLVNSNLVCKISDFGLSRVLEDDPDAAYTTSGGKIPIRWTAPEA
IAYRKFSSSSDVWSYGVVMWEVMSYGEQPYWNLTNRDVIKSVEEGYRLPAPMGCPGALHT
LMLDCWQKDRNERPKFCQIVTVLDKLIRTPETLKSVETLCRLNSPLMNRSIPDFTSCRSV
DEWLDTIKMGRYKDHFAAGGYRTLGHVMSMKQHDIQRLGVTLMGHQKKIMTSVQVMRAQV
LNQSVPSVHI
>Drer_43755.7 D. rerio
MMDILWILWTLPSVLAIEEILMDSTTATAELGWTVYPVSGTSDSSWEEVSGYDENMNTIR
TYQVCNVFDANQNNWVRTKYIRRRGAQRIHVEMKFSVRDCSSIPRVPGSCKETFNLYYYE
SDSDTATKTFPPWMENPWIKVDTIAADESFSQVDLGGRVMKINTEVRSFGPVSRNGFYLA
FQDYGGCMSLIAVRVFYRKCPRTIRNGAIFQETLSGAESTSLVAARGVCIPNAEEVDVPI
KLYCNGDGEWMVPIGRCMCKAGHEAVENGTVCRACPSGFFKTAQGDEKCLQCPINSRTTN
DGATNCVCRNGYYRTDSDPLEMPCTTVPSAPQNVISSVNETSLMLEWNPPRETGGRDDVV
YNIICKSCGGGRGGCTRCGDNVQFIPRQLGLTESRVFISDLLAHTQYTFEIQAVNGVSDQ
SPYSPQYTSVNITTNQAAPSTVSIMHQVSRTIDSITLSWSQPDQPNGVILDYELQYYEKD
QTEHNSSMVKSQTNTAVIRGLKPGGIYVFQVRARTVAGFGRYSGKMYFQTMTEEEYNTSI
QEKLPLIIGSAAAGLVFLIAVVVIIIVCNRRGFERADSEYTDKLQHYTSGHMSPGMKIYI
DPFTYEDPNEAVREFAKEIDISCVKIEQVIGAGEFGEVCSGNLKLPGKREMFVAIKTLKS
GYTEKQRRDFLSEASIMGQFDHPNVIHLEGVVTKSSPVMIITEFMENGSLDSFLRQNDGQ
FTVIQLVGMLRGIASGMKYLADMNYVHRDLAARNILVNSNLVCKVSDFGLSRFLEDDTSD
PTYTSALGGKIPIRWTAPEAIQYRKFTSASDVWSYGIVMWEVMSYGERPYWDMTNQDVIN
AIEQDYRLPPPMDCPSALHQLMLDCWQKDRNNRPKFSQIVNNLDKMIRNPNSLKAMTPLS
SGVHLPLLDRSVPDFSSFSSVDDWLDAIKMGQYKDNFANGNFTSFDLVSQMTMEDILRVG
VTLAGHQKKILNSVQMMRAQMNQIQSVEV
>Drer_44917.5 D. rerio
MDYRRIKRVLFVNVFMNFVFISLQKKEEVLLDMVASGAELGWLTSPVKDGWEIGQQAVNG
SLLYNYYVCNVEKGEQDNWLRTTFIQRNPAASRVFVELQFNVRDCNTFEAVPLNCKETFN
LYTSEADADIGTAFRKGLFRKVATIAPDEISDRGKMKMNTETKVVDNLSRKGFYLGFQDI
GACVAIYSVRVYYKTCPATVQSLAAFPETVAGGENQALREVAGSCVKNAISEGQPRIYCT
TDGEWVVPMSQCQCESGFEALEDTCQECQPGFFKPSVSSEACKPCPKNTEPSGPGATRCS
CNDGFYRAPEDPETSACFGLPSAPQSLLSSTAQMSFGRLQLSWKPPADNGGRSDITYSVV
CERCEGKTCQSCGEKVRFEPSNTELKETKVTVSELEPHLNYTFSVEARSGVSQFSHKRAV
SSINTTLHYTDPPKVTAMHLVESSPTSLSLQWSVSHRAKPVATRYELMFRKKENDEEKAS
GTVTTFTVLVLEKNSAQIKDLSPSTVYLFKVQALSSEGSPGSYSIEQEFSTLPPAGNTAV
ILGAAIGGAVMLFIVVVVLLLRKRRRNSHTRQGPEDTYFSSPDQLKPLKTYVDPHTYEDP
NTAVLKFASEIHPNHITKQKVIGAGEFGEVYRGILKAPSRKEVAVAIKTLKPGYTEKQRQ
DFLSEASIMGQFSHQNIIRLEGVVTKFKHAMIVTEYMENGALDKYLKDHDGEMSSFQLVG
MLRGIAAGMKYLSDMSYVHRDLAARNILVNSNLECKVSDFGLSRVLEDDPEGTYTTSGGK
IPIRWTAPEAIAYRKFTSASDVWSFGIVMWEVMAFGERPYWDMSNHEVMKAINEAFRLPA
PMDCPSAVYQLMLQCWQQDRSKRPRFGDIVSLLDKLLKSPDSLKPIADFDPRVSIRLPST

SGSDGSPFRSVSEWLESIKMSQYSENFSMAGVVSMEQVLQMKSEDIRNIGVRLPGHLKRI
AYSILGLKDQTSTLSVFAV
>Drer_75583.4 D. rerio
MVVDLLAHTNYTFEVEAMNGVSDLTGPPRQYVSLNVTTNQAAPSPITVVKKGKTTKNSIT
VSWQEPDRPNGIILEYEIKYFEKDQETSYTIIKSKETEVLVDGLKPSSAYIFQIRARTSA
GYGGFSRRFEFETSPYLAASSEQGQVPIIAVAVTVGIILIALVTGFLMSGRRCGYSKAKQ
DPDEEKMHFHNGHIKFPSVRTYIDPHTYEDPNQAVHEFAKEIDVCCITIERVIGAGEFGE
VCSGLLRLPGKREIPVAIKTLKAGYTEKQRRDFLGEASIMGQFEHPNIIHLKGVITKSKP
VMIITEYMENGSLDTFLKKNDGQFTVIQLVGMLRGIASGMRYLSEMGYVHRDLAARNILV
NSNLVCKVSDFGLSRVLEDDPEAAYTTRGGKIPIRWTAPEAIAYRKFTSASDVWSYGIVM
WEVMSYGERPYWEMSNQDVIKAVEENYRLPGPMDCPTALYHLMMDCWQKDRNSRPKFEEI
VSLLDKLIRNPSSLKGLVNPSNRVSNLLVEQGSPEGCVFRSVEEWLEAIKMGRYTELFME
SGYTSLQVVTQMTLEDLRRVGVTLAGHQKKILNSIQEMRVQMMNATMPI
>Drer_88414.3 D. rerio
MDRVCWIMALSWFWMVSTGLVSAEEEVLMNTKLETSDLRWTIYPSGDPEWEEMSGLDEEG
NSVRTFQVCPMDSSVSHWLRTRFIPRHGASQVYVEIRFTMMECSAMPASFRTCKETFNLY
YYQSDEDTASATHPAWMENPYSKVDTVAADFLLRRGGERKSNVKTVRVGPLSKKGFYLAF
QTQGACMALLSVRVFFKKCPAVSRAFSSFPETLPHSLVQQAEGVCVDNSAPTGQSTAPPT
MFCGEDGQWVGPPSSTCACKPGYEPVDSDRCRACGLGQYKASVGGSLCRVCPDNSNTHFA
GSSLCVCRPGYHRATSDLPDSACTKPPSAPRSIIYQINDTVVTLEWSEPLDRGGRSDLSY
SVECMHCRGSLCVQCADSITYRPGQMGVPGRRVIIRGLLPHTTYTFTVLAQNGVSAVSHT
SPASSSVNITTSRDVAVPVSGIRRIKASESSVSISWTVPPQTQHSIQDYQLRYSLKGQDD
GWQYVSSRSSSVVLNDLSRASQYQVQVRARTAAGYGHFSSAVSISTLPDDEESPSRLMLT
GVLVAIGLLILIAVVIVAVFCFRRSTRRRDPDPDKSGQFLMGQGIKVYIDPFTYEDPNEA
VREFAKEIDVSFVKIEEVIGAGEFGEVCRGRLKVPGKKENYVAIKTLKGGYTDKQRRDFL
SEASIMGQFQHPNIIHLEGVITASCPVMILTEYMENGALDSFLRLNDGQFTPIQLVGMLR
GIASGMKYLSEMSFVHRDLAARNILVNSNLVCKVSDFGLSRFLTENSSDPTYTSSLGGKI
PIRWTAPEAIAFRKFTSASDVWSYGIVMWEVMSFGERPYWDMSNQDVINAIEQDYRLPPP
PECPASLHQLMLDCWQKERSSRPRFCAIVSALDRLIRNPASLKITGRIPDGPSHPLLDQR
APPPLSHCSSVADWLRAIKMERYEDAFMQAGFTAIQHITHISTEDLLRIGVTLAGHQKKI
LSSVQTLRIHGGSLRY
>Drer_93563.5 D. rerio
MVCTSVSCVWIIFCLHLCFYVNTGIAQNTREVILLDSKAQQTELEWISSPPSGWEEISGL
DENYTPIRTYQVCQVMEPNQNNWLRTNWIEKGDAQRIFVELKFTLRDCNSLPGVVGSCKE
TFNLYYQETDVEVGRNIRESQYVKIDTIAADESFTQGDLGERKMKLNTEVRIIGPLSRRG
FYLAFQDVGACIALVSVKVYYKKCWSIIENLATFPDTVTGSEFSSLVEVEGTCVNDAEEE
ADNSPKMHCSAEGEWLVPIGKCICKAGFHQKGDACEPCGRGFYKSSSQDLQCSRCPAHSY
NDREGSWRCDCEDGYYRALSDPPSVSCTRPPSAPQNLLYNINHTTVSLEWTPPADTGGRN
DVTYRIICRRCTWEPEECFPCGSNVGYSPQQSGLTDTYVTITDLLAHANYTFEVEAVNGV
SDLSRTQRLFAAVSIATSQAGSTFFTNISVHFYTSADMFEVTAPSQVSEVIKERVQQHSV
QLSWQEPDQPNGVITEYEIKYYEKDQKDRIYSTVKSKSTSATVNNLKPSTAYVFQIRAFT
AAGYGIFGPRLEVTTKEESTGSATVISSEQNPVIIIAVVAVAGTIILVFMVFGFIIGRRH
CGYSKADQEGDEELYFQFKFPGTKTYIDPETYEDPNRAVHQFAKELDASCIKIERVIGAG
EFGEVCSGRLKLPGKRDVSVAIKTLKVGYTEKQRRDFLCEASIMGQFDHPNVVHLEGVVT
RGKPVMIVIEYMENGSLDAFLRKHDGQFTVIQLVGMLRGIAAGMRYLSDMGYVHRDLAAR
NILVNSNLVCKVSDFGLSRVIDDDPEAVYTTTGGKIPVRWTAMEAIQYRKFTSASDVWSY
GIVMWEVMSYGERPYWDMSNQDVIKAIEEGYRLPAPMDCPPGLHQLMLDCWQKDRADRPK
FDQIVGILDKMIRNPNTLKTPMGTCLRPISPLLDQNTPDFTSFRLVSEWLEAIKMERYMD
NFTAAGYSSLESVARMTIEDVMSLGISLVGHQKKIMSSIQTMRAQMLHLHGTGVQV
>Drer_96552.3 D. rerio
MALFRIYSFLAPFHILVLCQALRNYPDNEVTLLDSMSAPGDLGWEAYPSEGWEEISVMDE
RNIPMRTYQVCNVMEANQNNWLRTGLIQREGAQRVYVEIKFTLRDCNSLPGVPGTCKETF
NVYYHESNNAVAAPLRHIRENQYIKIDTIAADESFTQTDVGDRVMKLNTEVRDISGLSKR

GLYLAFQDLGACIALVSVRVFYKRCPLAVLNLARFPDTVTGGDSALVEVRGTCVEDAEEL
EGPRMFCSADGGWLVPIGRCVCRPGFEEVDGHCQPCRSGFYKASAMDAYCVKCPPHSYSH
QDKASECVCERGFYRAESDPRSMACTRPPSAPGNPISMVNETAVTLEWSPPRDSGGRGDV
SYSVHCRKCSGETGASERCVPCGSGAHFNPRQFGLTHPRVLVTELQPHTNYTFSVEALNG
VSDLSPSPRQLVSVNVTTSQTVSVILKERKGTDSVTLAWQGPEPVDGTVVEYEVTYYEKN
QQDQNYTVLKTKSNSMTVDGLKPGTTYIFRVRARTDGGYGNYKGEIELETSHEDMLAVGD
PNQQTILAISVAGGAVLLVLLVACFIVSGRRCGYIKAKQDPEEEKMQFQHGRVKLPETRT
YIHPHTYEDPNQAVRDFAKEIEVSNIRIERVIGAGEFGEVCSGRLRLPSKREIQVAIKSL
KAGYSEHQRRDFLSEASIMGQFDHPNIIRLEGVVTRCKPVMIVTEYMENGSLDTFLKKHD
GQFTVIQLLGMLRGIAAGMQYLSEMNYVHRDLAARNILVNGNLVCKVSDFGLSRVLEDDP
EAAYTTRGGKIPIRWTAPEAITYRKFTSASDVWSYGIVMWEVISYGERPYWEMSNQDVIK
AVDEGYRLPAPMDCPVVLHQLMLDCWEKNRSDRPKFGQIVNTLDRLIRNPSSLKQLANSA
VWEDPVTPEAAVNTVEDWLDLIKMGQYKEHFSSAGYVTLDSVLYVSSSELDKMGVELAGH
QKKILSSIQCLQAHHGTQVQV
>Drer_99872.3 D. rerio
VVLLNSKESQAELGWTSYPSNGWEEISGVDEKYKPIRTYQVCNVMEPSQNNWLQTGWIWR
QGGQRIFIELQFTLRDCNSIPGVSGSCKETFNLLYAESDWDLGRVSREDRYSKIDTIAAD
ESFTQGDLGERKMKLNTEVREIGHLNRKGFHLAFQDVGACVALVSVRVYYKRCLSTVQNL
AVFPDTVAEAAFSTLVEVRGACVNNSEVDTDSPPRMHCSAEGEWLVPIGKCSCSAGYEEG
HSSCEDRVSYLSMVDLLE
>Drer_111570.2 D. rerio
MPCTSVPSGPRNVISIVNETSVTLEWHSPRETGGREDVTYNIVCKKCQADRRSCSHCDDN
VEFQPRQLGLTESRVFISNLLAHTLYTFEIQAVNGVSNKSPYPAQHVSIDITTNQAAPSI
VPIMHQVSSTMKSITLSWPQPEQPNGIILDYELRYYEKELSEVNSTQMRSQTNTARVDGL
RPGTMYVMQVRARTVAGFGKYSSKMCFQTLTDDDYKSELREQLPLIAGSAAAGVVFIVSL
VAISIVCRRKRAYNKEAVYSDKLQHYSTGRELTLRPDLSNCHTPMGCPTLPGSPGMKIYI
DPFTYEDPNEAVREFAKEIDVSTVKIEEVIGAGEFGEVYKGRLKLPGKRELYVAIKTLKA
GYSEKQRRDFLSEASIMGQFDHPNIIRLEGVVTKSRPVMIVTEFMENGALDSFLRQNDGQ
FTVIQLVGMLRGIAAGMKYLSEMNYVHRDLAARNILVNSNLVCKVSDFGLSRYLQEDTSD
PTYTSSLGGKIPVRWTSPEAIAYRKFTSASDVWSYGIVMWEVMSFGERPYWDMSNQDVIN
AIEQDYRLPPPMDCPTALHQLMLDCWQKDRNARPRFTDIVNTLDKLIRNPTSLKAVASIP
TIPSQPLLDRSIPDFTTFSSVEDWLAAVKMSQYRDNFLNSGFTSLQLVTQMTSEDLLRIG
VTLAGHQKKILNSIQSMHLQMSQCQTGTALA
>Drer_112928.2 D. rerio
XCDHSVFTDGPPDKFSMNILGLYSQMPKFTAIMDIIRLLWIIRLVWAVEDVLMDSTTATA
ELGWTIYPSLGWEEVSGYDENMNTIRTYQVCNVFDANQNNWVRTKYIRRRGAQRIHVEMK
FSVRDCSSIPKVPGSCKETFNLYYFESDSDTATKVYPAWMENPWVKVDTIAADESFSQVD
LGGRVMKINTEVRSFGPVSRSGFYLAFQDYGACMSLIAVKVFYRKCPRIIRNGAIFQETL
SGAESTSLVSARGTCVPNGEEVDVPIKLYCNGDGEWLVPIGRCVCKAGHESVDNGTMCKA
CPFGSFKASQGDQQCLQCPINSRTSSEGATSCVCRNGYYRTDSDPLQMPCTTVPSAPQRV
ISSVNETSLRLEWDVPKESGGREDVVYNIICKSCGSGRGACTRCGDNVQFVPRQLGLTEP
RIHISDLLAHTQYTFEIQAVNGVSDQSPYSPQFTAVNITTNQAAPSAVSIMHQVSRAVDS
ITLSWSQPDQPNGVILDYELQYYEKNAVESNSSLLRSQTNTAVIRGLKSGTIYVFQVRAR
TVAGFGRFSGKMYFQTMTEEEYNSSLQEKLPLIIGSAAAGVVFLIAVVVLIIVCNRRGSD
RTDSEYTDKLQHYTSGHMSPGMKIYIDPFTYEDPNEAVREFAKEIDVTCVKIEQVIGAGE
FGEVCSGNLRLPGKREILVAIKTLKSGYTEKQRRDFLSEASIMGQFDHPNIIHLEGVVTK
STPVMIITEFMENGSLDSFLRQNDGQFTVIQLVGMLRGIAAGMKYLCDMNYVHRDLAARN
ILVNSNLVCKVSDFGLSRFLEDDTSDPTYTSALGGKIPIRWTAPEAIQYRKFTSSSDVWS
YGIVMWEVMSYGERPYWDMSNQDVINAIEQDYRLPPPMDCPSALHQLMLDCWQKDRNNRP
KFSQIVNTLDKMIRNPGSLKATTPLSSGVHLPLLDRSTPDFSSFSTVDEWLDAIKMSQYK
ENFANEDLTTFEAVSQMTMDDILRVGVTLAGHQKKILNSVQMMRAQMNQIQSVEV
>Drer_113830.2 D. rerio
XLCVALMEMWRIFLSVSLVVQHSSAEEVMLLDTSESTAELGWTTYPDTGWDEVSVLDDKG

RLMRTFEVCNVNQNPRLQDNWLATPFLYRQSAPRIFVTLRFSVRDCASLRSPSPTCRETL
TLYYKQADSQRELERTWAAEASSGETREGWVKIDTIAADKSFSRVEPSLPHQYKSENARR
INVKTRSFAPLTRKGFVLAIVDSGACVSLMGVSIFYRRCPATSRFLAFYPATPSGAEPTS
LVPVTGTCVPHSQSQGGTAPRMHCNTEGEWLVPVGGCTCDAGYEPNHNGSACLVCPIGSY
KAVAGSVSCTECPANSRTSSEGAKVCECRSGYYRAPADANTTACTSSPSAPLALSWEYES
SEGGVSLRWRPPADMGGRTDVWYGVVCRICPSATSTPPSMCSWCGETVTYSPSQNGLKQT
RVTLKNLLTRVTYLIQVQAMNEVSALSPFPPQFASINFTTSQSVPSEVPMLHQLSRVQDS
ITLSWPQPDRPNGDILEYQLRYYDKGSDEDSALSMYSETNTVTVTGLIPGSIYAFQIRAR
NERGFGPYSHTIYFSTLASEGQSKQIQNRLPLMIGSVMGGAAFLLVVIAIIIVFVFRSKR
RESPYSDRLQRYISNRGGVKYYVDPSTYEDPSEAVKEFAREIDPAHLKIEEVIGAAQFGE
VSRGRYRPIGRREALVAVKTLRWGVTEREKNVFLSEAGVLGQFDHPNVLKLEGVITRTPP
ERIITEFMENGPLDAFLRENEDQFSVLQLVGMVRGVGAGMRYLSERNFVHRDLAARNVLV
NSNLVCKVSDFGLSRLMRGLDHNMPTYTASLGSKIPVRWTAPEAFQHRKFSSASDVWSFG
ILMWEVMSYGERPYWDMSNQEVMKAVVDQYRLPAPNGCPPALHSLMLQCWQADRQDRPGF
DSLLSSLDRLIRHPASLKAEHSRPTQPLLSPTPTDLSSVNTVGDWLTVLKMDRYKDAFER
AQYHSLESVSMLTMQDVQALGVNLLGHQRKIVNAAKQLRTHLTQGHVEV
>Drer_121462.2 D. rerio
MTFQRFLFMFALPGLMLSDPGHPNQVVLLDTTAVLGELSWKTYPINGWDAITEMDELNRP
IHTFQVCHVMEPNQNNWLRTNWIPRQAAQKIYVELRFTLRDCNSIPWVSGTCKETFNLLY
LETDEPHGANAHFHPNDYAKIDTIAADESFTQTDLGDRVLRLNTEVREVTIARKGFYLAF
QDVGACIALVSVRVYYKKCPSTLRNLAAFPDTVPRVDSSSLVEVRGACVENAEERDTPKL
YCGADGDWLVPLGRCVCSIGYEESDGLCLACRPGFYKAFAGNIKCSKCPPHSSSHAEGSA
QCHCEKNYYRASKDPPTMACTRPPSPPRNLVFNINDTALFLEWTPPSDTGGRKDISYTAL
CLRCGADGQDCEPCNSNVRFVPKPTGLTSTSVVVQDFVAHANYTFQIEALNGVSGLGRSM
RQIANITVSTEQAGPSLVGVVRKDWASQTSVALSWQETEQPHAAILDYEIKYYEKEQEQL
SYSSTRTKSPSVIVTGLKPSTVYAFHVRARTLAGYSSYSPKFEFATGDEDSEEAVDQGQV
LVIVTATVGGFSLLVILTLFLLITGRCQWYIKSKIKSEDKKRTQYQNGHVPFPGLKTYID
PDTYEDPSQAVHEFAKEIDPSRIRIERVIGAGEFGEVCSGRLRIPGKKEIPVAIKTLKGG
YTERQRRDFLREASIMGQFDNPNIIHLEGVVTKSRPVMIVVEYMENGSLDSFLRKHDGHF
TVIQLVGMLRGIASGMMYLSDIGYVHRDLAARNILVDDNLVCKVSDFGLSRVLEDDPEAA
YTTTVLFCQGGKIPIRWTAPEAIAYRKFSSASDAWSYGIVMWEVMSYGERPYWEMSNQDV
ILSIEEGYRLPAPMGCPVALHQLMLLCWQKERSRRPHFNDVVSFLDKLIRNPSSLLTLVE
DVNSFPESPEDLPDYPLFISIGDWLDSIKMSQYKNNFIAAGYTTLDSISTMTIDDVRRIG
VSLIGHQRRIVSSIQALRLQFLHVQQSGFHENWTIYPEEPQ
>Drer_121980.1 D. rerio
VPSGPRNVISIVNETSVTLEWHSPRETGGREDVTYNIVCKKCQADRRSCSHCDDNVEFQP
RQLGLTESRVFISNLLAHTLYTFEIQAVNGVSNKSPYPAQHVSIDITTNQAAPSIVPIMH
QVSSTMKSITLSWPQPEQPNGIILDYELRYYEKELSEVNSTQMRSQTNTARVDGLRPGTM
YVMQVRARTVAGFGKYSSKMCFQTLTDDDYKSELREQLPLIAGSAAAGVVFIVSLVAISI
VCRRKRAYNKEAVYSDKLQHYSTGRGSPGMKIYIDPFTYEDPNEAVREFAKEIDVSTVKI
EEVIGAGEFGEVYKGRLKLPGKRELYVAIKTLKAGYSEKQRRDFLSEASIMGQFDHPNII
RLEGVVTKSRPVMIVTEFMENGALDSFLRQNDGQFTVIQLVGMLRGIAAGMKYLSEMNYV
HRDLAARNILVNSNLVCKVSDFGLSRYLQEDTSDPTYTSSLGGKIPVRWTSPEAIAYRKF
TSASDVWSYGIVMWEVMSFGERPYWDMSNQDVINAIEQDYRLPPPMDCPTALHQLMLDCW
QKDRNARPRFTDIVNTLDKLIRNPTSLKAVASIPTIPSQPLLDRSIPDFTTFSSVEDWLA
AVKMSQYRDNFLNSGFTSLQLVTQMTSEDLLRIGVTLAGHQKKILNSIQSMHLQMSQ
>Drer_123962.1 D. rerio
XNANSTGTDATSVRARSGGDAHEMTPMAALVFTATFALIIWIPTFALTARVYPPNEVTLL
DSRTVQGELKWVASPTEGGWEEVSIMDEKNTPIRTYQVCNVMEPSQNNWLRTDWIPRGGA
QRVYIEIKFTLRDCNSLPGVMGTCKETFNLYYYESNNDKERYIRENQFTKIDTIAADESF
TQVDIGDRIMKLNTEVRDVGILTRKGFYLAFQDVGACIALVSVRIFYKKCPLTVRNLAQF
PDTTTGADTSSLVEVRGSCVDNSEEREVPKMYCGADGEWLVPIGNCLCNPGYEEHEGTCQ
ACKIGNYRALATDGSCSKCPLHSYSVREGSTSCACDKGFFRSETDPASMPCTQPPSAPQH

LISNVNETSVNLEWTAPASSGGRQDLAYNVICKRCSSDGQRCQPCGNGIHFSPQQLGLRT
TRVSINDLQAHTNYTFEIWAVNGVSKQSPGPEQAVSVTVTTNQAAPSPVTTIQAKDITRH
AVSLAWQQPERPNGVILEYEVKYYEKDQNERSYRIMKTSSRSAEIKGLSPLTSYVFHVRA
RTAAGYGEFSAPFEFMTNSVPSPIIGDGANSTVLLVSVIGSVILLLIFIGVFIISRRRSK
YSKAKQDSDEERHLHPGVRIYVDPFTYEDPNQAVREFAKEIDASCIKIEKVIGIGEFGEV
CSGRLKMPGKREICVAIKTLKAGYTDKQRRDFLSEASIMGQFDHPNIIRLEGVVTKCKPV
MIITEYMENGSLDAFLRKNDGRFTVIQLVGILRGIASGMKYLSDMSYVHRDLAARNILVN
SNLVCKVSDFGMSRVLEEDPDAAYTTREITGTYQSQGGKIPIRWTAPEAITYRKFTSASD
VWSYGIVMWEVMSYGERPYWDMSNQDVIKAIEEGYRLPPPMDCPVSLHQLMLDCWQKERA
ERPKFSQIVNMLDKLIRNPNSLKRTGGEIARPNTTLLEPSSPEFTSTLGSVADWLQAINM
ERYRDNFTAAGYTTPEAVVHMTQEDMTRIGISTAAHQDKILNSAQGMLSQMQQMQDRMVP
V
>Drer_131364.1 D. rerio
MVCTSVSCVWIIFCLHLCFYVNTGIAQNTREVILLDSKAQQTELEWISSPPSGWEEISGL
DENYTPIRTYQVCQVMEPNQNNWLRTNWIEKGDAQRIFVELKFTLRDCNSLPGVVGSCKE
TFNLYYQETDVEVGRNIRESQYVKIDTIAADESFTQGDLGERKMKLNTEVRIIGPLSRRG
FYLAFQDVGACIALVSVKVYYKKCWSIIENLATFPDTVTGSEFSSLVEVEGTCVNDAEEE
ADNSPKMHCSAEGEWLVPIGKCICKAGFHQKGDACEPCGRGFYKSSSQDLQCSRCPAHSY
NDREGSWRCDCEDGYYRALSDPPSVSCTRPPSAPQNLLYNINHTTVSLEWTPPADTGGRN
DVTYRIICRRCTWEPEECFPCGSNVGYSPQQSGLTDTYVTITDLLAHANYTFEVEAVNGV
SDLSRTQRLFAAVSIATSQAAPSQVSEVIKERVQQHSVQLSWQEPDQPNGVITEYEIKYY
EKDQKDRIYSTVKSKSTSATVNNLKPSTAYVFQIRAFTAAGYGIFGPRLEVTTKEESTGS
ATVISSEQNPVIIIAVVAVAGTIILVFMVFGFIIGRRHCGYSKADQEGDEELYFQFKFPG
TKTYIDPETYEDPNRAVHQFAKELDASCIKIERVIGAGEFGEVCSGRLKLPGKRDVSVAI
KTLKVGYTEKQRRDFLCEASIMGQFDHPNVVHLEGVVTRGKPVMIVIEYMENGSLDAFLR
KHDGQFTVIQLVGMLRGIAAGMRYLSDMGYVHRDLAARNILVNSNLVCKVSDFGLSRVID
DDPEAVYTTTGGKIPVRWTAMEAIQYRKFTSASDVWSYGIVMWEVMSYGERPYWDMSNQD
VIKAIEEGYRLPAPMDCPPGLHQLMLDCWQKDRADRPKFDQIVGILDKMIRNPNTLKTPM
GTCLRPISPLLDQNTPDFTSFRLVSEWLEAIKMERYMDNFTAAGYSSLESVARMTIEDVM
SLGISLVGHQKKIMSSIQTMRAQMLHLHGTGVQV
>Drer_134275.1 D. rerio
MTPMAALVFTATFALIIWIPTFALTARVYPPNEVTLLDSRTVQGELKWVASPTEGGWEEV
SIMDEKNTPIRTYQVCNVMEPSQNNWLRTDWIPRGGAQRVYIEIKFTLRDCNSLPGVMGT
CKETFNLYYYESNNDKERYIRENQFTKIDTIAADESFTQVDIGDRIMKLNTEVRDVGILT
RKGFYLAFQDVGACIALVSVRIFYKKCPLTVRNLAQFPDTTTGADTSSLVEVRGSCVDNS
EEREVPKMYCGADGEWLVPIGNCLCNPGYEEHEGTCQACKIGNYRALATDGSCSKCPLHS
YSVREGSTSCACDKGFFRSETDPASMPCTQPPSAPQHLISNVNETSVNLEWTAPASSGGR
QDLAYNVICKRCSSDGQRCQPCGNGIHFSPQQLGLRTTRVSINDLQAHTNYTFEIWAVNG
VSKQSPGPEQAVSVTVTTNQAAPSPVTTIQAKDITRHAVSLAWQQPERPNGVILEYEVKY
YEKDQNERSYRIMKTSSRSAEIKGLSPLTSYVFHVRARTAAGYGEFSAPFEFMTNSVPSP
IIGDGANSTVLLVSVIGSVILLLIFIGVFIISRRQVTPSSAGSLGLRQCPLWCCYPFHCF
SSVTCPHTERRSKYSKAKQDSDEERHLHPGVRIYVDPFTYEDPNQAVREFAKEIDASCIK
IEKVIGIGEFGEVCSGRLKMPGKREICVAIKTLKAGYTDKQRRDFLSEASIMGQFDHPNI
IRLEGVVTKCKPVMIITEYMENGSLDAFLRKNDGRFTVIQLVGILRGIASGMKYLSDMSY
VHRDLAARNILVNSNLVCKVSDFGMSRVLEEDPDAAYTTREITGTYQSQGGKIPIRWTAP
EAITYRKFTSASDVWSYGIVMWEVMSYGERPYWDMSNQDVIKAIEEGYRLPPPMDCPVSL
HQLMLDCWQKERAERPKFSQIVNMLDKLIRNPNSLKRTGGEIARPNTTLLEPSSPEFTST
LGSVADWLQAINMERYRDNFTAAGYTTPEAVVHMTQEDMTRIGISTAAHQDKILNSAQGM
LSQMQQMQDRMVPV
>Drer_134983.1 D. rerio
MRSRWTYVAWICVFSLGAFRTFCIPANEVNLLDSRSVMGDLGWVAYPKNGWEEIGEVDEN
YAPIHTYQVCKVMEHNQNNWLQTNWILTQGAQRVFLELKFTLRDCNSLPGGLGTCKETFN
VYYYETNDEERRNIRESQYSKIDTIAADESFTELDLGDRVMKLNTEVRDLGPLTKKGFYL

AFQDLGACIALVSVRVFYKKCPFVVRNLALFPDTITGADSSQLLEVSGTCVNNSVADELP
RMHCSAEGEWLVPIGRCMCQAGHEEVNGSCQVCKPGFYRSVLESRSCSKCPPRSFSKVEA
SISCQCEQGYFRTQADPANMACTRKCHCLDKLKTKLFF
>Drer_135357.1 D. rerio
XLMNTKTETSDLKWTTHSRSKPEWEEVSGLDEENNSVRTYQICQADGSSSHWLRSKLIER
RGASQVYVELFFTMVECSSRNTHHRSCKETFNLYYYQSDTDDATATHPAWMENPYTKVDT
VAADFLLRKGGEKKVNVKTLRLGPLSKRGFYLAFQAQGACMALLSVRVFFKKCPALTRSL
SVFPETVPRSLVQEAVGQCVANAAQPGPSPRPPKMFCGEDGQWVDQPTTTCTCLPGFEAS
HGELECRACPVGLFKMGSGTGPCSVCPENSQTGETGSAACVCRSGFYRALSDSADTPCTR
PPSSPRSPVPQVNDTSLTLEWSEPLDSGGRSDLSYSVECRMCSTPGSPCTLCSDGVNYRP
SQTGIQGRRVSIWGLRPHTTYSFTVMALNGVSAQSQQGPAGETINITTSPNVPVLVSGLR
KSTATESSLTLYWNTPTQSHYRILQYQIRYCEKERGSEENSCHYMESNNNEVVLSDLRRA
TQYEVQVRARTFAGYGSFGKAILFRTLPDEDDSSSPLLVTGILIAMGMLLLIIVIGAAIY
CIRKQNNYKDPELSDKNGQYLMGQGVKVYIDPFTYEDPNEAVREFAKEIDVSCVKIEEVI
GAGEFGEVCRGRLRIPGKKENYVAIKTLKGGYTDKQRRDFLAEASIMGQFQHPNIIHLEG
IITASCPVMILTEFMENGALDSFLRLNDGQFTPIQLVGMLRGIASGMKYLSEMSYVHRDL
AARNILVNSNLVCKVSDFGLSRFLQENSSDPTYTSSLGGKIPIRWTAPEAIAFRKFTCAS
DVWSYGIVMWEVMSFGERPYWDMSNQDVINAIEQDYRLPPPPDCPTYLHQLMLDCWQKER
TARPRFANIVSALDKLIRNPASLKITAQEGAGPSHPLLDQRSPLTPSSCGTVGDWLRAIK
MERYEETFLQAGYTSMQLVTHINTEDLLRLGITLAGHQKKILSSIEALGIQNKAPGNVLY
>Drer_136066.1 D. rerio
SETLMDTRTATAELGWTAYPSSGWEEVSGYDENLNTIRTYQVCNVFESNQNNWLLTTFIA
RRGAQRIYVEMRFTVRDCSSIPRVPGSCKETFNLYYYETDSVIATKGTAFWMEAPYLKVD
TIAADESFSQVDFGGRLMKVNTEVRSFGPLSKNGFYLAFQDYGACMSLLSVRVFYKKCPS
VVQNFAIFPETMTGAESTSLVIARGMCIPNSEEVDVPIKLYCNGDGDWMVPIGSCTCKAG
FEPDNGNICRVL
>Drer_140419.1 D. rerio
MQWEEVSGYDDAMNPIRTYQVCNVRELNQNNWLRSDFIPRKDVLRVYVEMKFTVRDCNSI
PNIPGSCKETFNLFYYESDSDSATATSPFWMENPYVKVDTIAPDESFSMLESGRVNTKIR
SFGPLSKAGFYLAFQDLGACMSLISVRAFYKKCSTTIANFAVFPETATGAEATSLVIAPG
TCVPNALEVSVPLKLYCNGDGEWMVPVGSCTCMAGFEPAVKETQCQACSPGTFKSKQGEG
LCSPCPPNSRTSSGAASICSCRTGYYRADSDSPDSGCTTVPSAPRNVISSVNETSLILEW
SEPRDQGGREDLLYNVICKKCLPERGTCTRCDDNVDISPRHLGLTERHVTIRNLQAHTQY
SFEIQAVNGVASKSPYAPQFASVNITTNQAAPSAVPTVHLTGASANTMTLSWLPPEKPNG
IILDYEIKYHEKDQGEAIAHTMTAQHSYARIDGLKPGTPYVVQVRARTVAGYGRYSSPTD
FGTNHQADADKALQEQLPLIVGSLTAGLVFIIVVVVIAIVCLRKQRNGSESEYTEKLQQY
KSPIVTPGMKVYIDPFTYEDPNEAIREFAKEIDVSCVKIEEVIGAGEFGEVCRGRLKLPG
RREIIVAIKTLKAGYTERQRRDFLSEASIMGQFDHPNIIRLEGVVTKSRPVMIVTEFMEN
GALDSFLRLNDGQFTVIQLVGMLRGIAAGMKYLSDMNYVHRDLAARNILVNSNLVCKVSD
FGLSRFLEDDPTDPTYTSSLGGKIPIRWTAPEAIAYRKFTSASDVWSYGIVMWEVMSYGE
RPYWDMSNQDVINAVEQDYRLPPPMDCPTALHQLMLDCWVKERNLRPKFSQIVNTLDKLI
RNAASLKVVTSTHSGDLLRIGVTLAGHQKKILGSIQDMRLQMNQTLPVQV
>Drer_141023.1 D. rerio
AGDFGEVCRGCLKLPSKRDLPVAIKTLRAGCSEKQRRSFLSEAGILGQFDHANILRLEGV
ITRAALLFSLTVYNLLRNSLLLDSFLRKHEGQLTVLQLADLLSGVASGMKYLTEMGFLHR
RLAAHKVLVNSSLVCKVSGFRPLQDDKIEAVYSTLHGGKSLVLWTAPEAIQYRRYSSASD
VWSFGIVMWEVMSFGERPYWDMGNQDVIKAIEDGFRLPAPLNCPPSLHQLMLDCWQKERT
ERPTFTQIHSALSKSIRSPDNIGSSTLGRRTLGSSVSLAERTLPSFPSFSSVGEWLEAVD
MGRYKDNFTAAGYCYLESVARMTVQDVLSLGITCLEHQKQILSAIQTLRAQVIQMHGRGV
QV
>Pmar_131.1 P. marinus
WEEFSRIRDRNIPHCFYQVRNVMNQNQNWLRAHWIWRDGVQRAYVELKFTPDDCNSVQSV
ALTCKEMFNLLLQEQPPTRKPSTIAANQTFKHVDVADHMLKLNTEVRKTGPLSRKGFFVA

FHDMGACMALMSMRVYFKKCPAVVCAFASFPDKSSSLVEVSGLCVPNSVASDDPRMYCSK
DGEWLVPVGQHMCRPGYGANGGICTACPLSYYKKTPAIQKTPACCLHEASYYEAGSDAVN
MTYTRPQQGRAITWSVNETSALLEWSPPGRRRSPPVPHLRWQGQCALLCGGGVHFLPGHE
GLTVPGVTIVRLLAHTNYSFHIEALNRVS
>Pmar_2234.1 P. marinus
WEEISGYDEYYTPITTYQVCHVMEPSQNNWLRTNWISCGGAQRIYIELKFTLRDCSSIPG
VAGTCKETFNLHYYESDSDSSAHVDENQFFKIDTIAADESFTQVDLGGRIMKLNTEVRDV
GPLAKKGFHLAFQDVGACIALVSVRVYYKRCPRTVRGLAVFPDTVTGADSSSLIEVRGAC
VDNSAEKEAPRMFCSADGEWLVPIGRCVCGPGYEEVVVGACRACGIGFYKEIAGEQMCSK
CPPHSLSHRAGSYDCECDESYYRAKADPKSMACTRSTWSPSEGLTSVKDPTQTLEWSAPS
DTGGRRVLNYSVACRVCAPAEPLPASGDDGSSGAAGSPRCLPCGAEVRFAPRQSGVTERR
VTVTGLAAHTNYTFEVRATNGVSELTRAPPQSASIRLTMNPAAPGQILDVRQNRISKDAI
ALSWSEPKHPNSVILDYEITFSEKEQTEISYTTVKSKWNTITINGLKPSTIYTFQVRTRT
TAGYGQYSKPFHFQTSNEPLDISSDPSQSTIVWLSVATGIIVVLIIVCFLLKGRYCGYSK
AKQNCDEDKQHFQVKLPGLKMYIDPFTYEDPCEAVHEFAREIDVSYIKIEGVVGAGEFGE
VCRGRLRLPGKGEASVAIKTLKAGYTEEQRRDFLCEASIMGQFDHPNIIRLEGVITKSIP
VMIVTEYMENGSLDSFLRKNDGQFTSIQLVGMLRGIAAGMKYLSDMNYVHRDLAARNILV
NSNLVCKVSDFGLSRVLEDDPEAAYTAKGGRIPVRWTAPEAIAYRKFTSASDVWSYGIVM
WEVMSYGERPYWDMSNQDITRVYDEGEKAPPPKKNDTTFRCLHVSIDQKHRERQRNYCRV
GSNEVNAMVRSERSYRGGGGEGAMPQNPFLEHSVPLFIAFRSVGDWLDVIKMGRYKDNFA
VAGFNHLESVAQMSLDDVIRIGITLVGHQKKILNSIQIMRAQMVQMQDSGVQV
>Pmar_5131.1 P. marinus
VSPACPSGTFKASTGLGSCLACPANSRTPGEASMSCVCRNGYYRADKDPAETPCTSSPSA
PRDVTAVVNKTSLTLEWALPRDLGSRDDVLYNVVCRRCTDTRRLNCGRCLEPVEFVPRQQ
GLTETRVFISGLLAHTPYTFEIQAVNGVSERSPYPPQSVAVNITTNQAAPSQVPIMHHVS
STMNTIKLSWPQPDQPNGIILDYELRYCEKNQDFHNSSTVKSQTNTAVVDGLKPGTIYMF
QVRARTEAGFGKYGGKMLFQTLTSEDRENMIREQIPLIAGSAAAGIVFIVVIAVIIIVCS
RRRYERADPEYTDKLQHYTGGHVSSRHKIYIDPFTYEDPNEAVREFAKEIDVSCVKIEQV
IGAGEFGEVCSGRLKLPGKREMAVAIKTLKAGYTERQRRDFLSEASIMGQFDHPNVIHLE
GVVTKSRPVMIITEFMENGSLDSFLRQNDGQFTVIQLVGMLRGIAAGMKYLADMSYVHRD
LAARNILVNSNLVCKVSDFGLSRFLEDDTSDPTYTSAM
>Pmar_7260.1 P. marinus
PIFKGPCPRGLVCCFRLITTNSCRTGRLIFTVNLLDTTNIFGQLGWEATPTTGWEEMAIQ
INQTALIQAYQVCNVVKPNQNNWLRTRWIARDPAQRVYVEIKFTLRDCGSMAGVASTCKE
TFNLYYYEADSGSSANMDESLFAKVGTIAADSNFDNVDVKDRVLKLNTEVRELPPLSWPG
FYLAFQDVGACVALVSARVYYKKCPAVVRDLARFPDTVANALSSPLVEVRGSCVPNAVAA
TDPRMHCSTDGEWLVPIGLCVCHAGYEAAGSQCRACGLGYYKNTTGGQPCDLCPPESFTT
RLASTHCGCKAGYYRAEADPVSSPCTRPPSAARNVISSVNETSVILEWSPPQDSGGRGDV
SYRLVCQTCGGGRSPCAPCGDSVRFLPRREGLAVPGVTIAHLLAHTNYTFRIEALNGVSR
LSPAPPKAVSVSLTTNQAAPSKIRSLWYEMQTKDHVKLSWFQPDQPNGIILDYEIKYYEK
GQRDTSYNILHTKVPSASIEGLRAKIVYGFEVRARTAAGFGKPSDPVEINTDQYRKVTLV
VCSGGFTEHSNTTRVAVIVVCAVILLAFIVFTVVCIRRSCFFLLPSIAGDENVYMKLKFP
NRRNYVDPFTYEDPNKAVQEFAREIEPSWITIEKVIGSGESGEVCRGRLRVPGQAEAPVA
IKTLKAGYSEKQRRDFLSEASIMGQFEHPSIIRLEGVVTKSKPVMIITEFMENGSLDAFL
RGNDGQFNVGQLVGMLRGIAAGMKYLSDMNYIHRDLAARNVLVDSKLVCKVSDFGLSRGL
EDDPQAVYTTHGGKIPIRWTAPEAIAFRKFTSASDVWSFGVLMWEVMSYGERPYWDMNNQ
DVIQAIEEGYRLPAPMDCPPALHQLMLDCWQKERTERPNFAQILAYLDKLLQNPASLHAT
SQSSVELSNPLLERKAPEYTMFCSVGEWLDDIKMGRYKENFTNAGLVSWPAVAQMTPDDL
QRLGVTLAGHQKRIMTSIQTLRVQLANAHNVNVSC
>Pmar_7951.1 P. marinus
WEEVSGFDETMNTIRTYQVCNVFEANQNNWLRTKFIERKGAQRVYVEMKFTVRDCSSIPN
VPGSCKETFNLYYYETDGDVATKTKPFWMENPYIKVDTIAPDESFSQVDLGGRMMKINNE
LRSFGPLSKNGFYLAFQDFGGCMSLISVRVFFRKCPPVVQSFAAFPETLTGPESTSLVIA

RGSCILNAEEVNVPIKLYCNGDGEWMVPIGGCTCKPGYEPSGGRSTCNACPLGTFKPQQG
EHECQQCPPGSRMSSVGGTSCVCRNGHFRAPSDPPDMPCTSTPSAPRGVMSSVNETSVVL
EWSAPLDSGRRSDLAYNVLCRLCRKDRQVCTRCNEAVRFEPRQLGLRQTRVLVSELMAHT
PYTFEVQAVNGVSEQSPYPPQSVAVNITTNQAAPSSVPIIHQVTSSVTSITLSWPQPEQP
NGIILDYELRYYEKSQDERNASFMRSQTNTARVDGLQPGTIYVFQVRARTVSGFGRYSGK
MYFQTLEEGSYPERLKEQLPLIAGSAAATIVFIVAVVVIAIVYCRKRSYGRPDPEYTDKL
QQYHTGHSTPGMKMYIDPFTYEDPNEAIREFAREIDISCVTIEQVIGSGEFGEVCRGRLR
MPGKREMYVAIKTLKSAYTEKQRRDFLSEASIMGQFDHPNIIRLEGVVTKSRPVMIVTEF
MENGSLDSFLRQNDGQFTVIQLVGMLRGIAAGMKYLSDMNYVHRDLAARNILVNSNLVCK
VSDFGLSRFLENDPTDPTYTSGMGGKIPIRWTAPEAIQYRKFTSASDVWSYGIVMWEVMS
YGERPYWDMTNEDVINAVEQDYRLPAPMDCPAALHQLMLDCWQKSRNDRPKFGQIVSTLD
KMIRNPASLKAMSCVTSAPSLPLLDRTMPDFTSFASVDEWLDAIKMGRYKDNFANTGFSS
FDLVSRMTAEDILRIGVTLAGHQKKILNSIQMMRAQMNQIQAVQV
>Pmar_9310.1 P. marinus
SARRSPARLALCTFPRCGQVLGAECCVLSPAVNILDSKTAQEELGWIASPPEGQWEEISG
YDEFYTPIRTYQVCHVMKPNQNNWLRTNWITRGGAQRVFLELKFTLRDCNSIPGIPGTCK
ETFNVYYHESDSERVSGIHESQYVKVDTIAADESFTEIDLGGRIMKLNTEVRDVGPLAKK
GFHLAFQDVGACIALVSVRVYYKRCPRTVRGLAVFPDTVTGADSSSLIEVRGACVDNSAE
KEAPRMFCSVDGEWLVPIGRCVCGPGYEQAEDGCQ
>Pmar_9675.1 P. marinus
NFLSLTAWCDVSHTTVINAIEQDYRLPAPMDCPAALHQLMLDCWQKERNERPKFGQIVNT
LDKLIRTPASLKTVACVTPGISQPLLDRSVPDFASFGSVDEWLDAIKMGRYKDNFASAGF
TSFDLVSRMTAEDILRIGVTLAGHQKKILNSIQTMRVQ
>Pmar_9793.1 P. marinus
VDLLNTMGIQGEMGWRAFPPTGWEEISGIGDQNMPDRFYQVCNVMKQNQNNWLRTHWIRR
DGAQRAYVELKFTLRDCNSIPGVSRTCKETFNLLVRETDSDEEPELQEGLYSKIDTIAAD
ESFTHVDVGDRVLKLNTEVREIGLLSRKGFFMAFQDVGACVALVSVRVYYKKCPAVVREL
ASFPDTVTGTDSSSLVEVRGSCVRNAVASGDPRMHCSTDGEWLVPIGQCVCRPGYEAIGS
ICKACPLGYYKEAAGDERCSLCPAHSYSTRETPARCFCEAGYYKAESDAVSMACTRPPSA
ARNVISSVNETSVILEWSPPQDSGGRGDVSYRLVCQTCGGGRSPCAPCGDSVRFLPRREG
LAVPGVTIAHLLAHTNYTFRIEALNGVSRLSPAPPKAVSVSLTTNQAAPSEILVVQKEGE
TRDSLTLSWSEPQQPNGIILEYELSCCHKDSKEQNCTLVKASARGATVSGLRAGAVYTFR
VRARTAAGFGAYSTRVFYSTSQASPLTGEMKEITILGACVSMVILLLIFFIACFISGGRC
RHTKGGHVPDEEKLNFWSGHIKLRRIKTYVDPHTYEDPAQAVHEFAKEITVSCISIQRVV
GAGEFGEVYSGVLRVPGQAEAPVAIKTLKAGYSEKQRRDFLSEASIMGQFEHPSIIRLEG
VVTKTTPVMIITEYMENGSLDSFLRKQDGQFSVGQLVGMLRGIAAGVKYLSDMNYIHRDL
AARNVLVDSKLVCKVSDFGLSRGLEDDPQAVYTTHGSKIPIRWTAPEAIAFHKFTTASDV
WSFGILMWEVMSYGERPYWNMTNQDVIQSIEEGYRLPSPMDCPPALHQLMLDCWQKERTE
RPNSGQILVYLDKLLQNPASLNVPSHSNAQVSDPLLGQAVPGYTTVRTVSEWLDAIHMAR
YRDNFLAAGYTSLEAVRHMTLSDIHRIGVTLVGHQKKILNSIQVMRAQTLPA
>Pmar_10202.1 P. marinus
SSCCLSMLILLTLSTIPCLNKNILQAEKSRELNLMDTTTVMGELGWKSTPSTGWEELTIM
LNQTTPVQTFQVCNMGSNQNNWLRTRWIARDPAQRVYVEIKFTLRDCGSMAGVASTCKET
FNLYYYEADSGAEIDDGKFAKVGTIASDMIFDKTDVIDRVLKLNTEVRELPPLSWPGFYL
AFQDVGACVALVSVRVYYKKCPAVVRELASFPDTVTGTDSSPLVEVRGSCVPNAVASGDP
RMHCSTDGEWLVPIGQCVCRPGYEAAGHECRECGTGYFKVLAGEENCTPCPNHSYTTSET
STECVCESDFYRAEGDGPASPCTRPPSAARNVISSVNETSVILEWSPPQDSGGRGDVSYR
LVCQTCGGGRSPCAPCGDSVRFLPRREGLAVPGVTIAHLLAHTNYTFRIEALNGVSRLSP
APPKAVSVSLTTNQAAPSKIDSLNADRPTKDTVQLSWPAPVKPNGVILDYEVKYYEVGQR
DTSYNILHTKVPSASIEGLRPDLVYGFEVRARTAAGYGKPSDPVEIDTSEDGFVTHPGAL
TQVLVIASCVFVAVVLIVLALVYVRRRRSRNIAARGDEEEQFPIKPNEVHLRTKRNYVDP
FTYEDPNKAVQDFAREIEPSWIIIEKVIGSGESGDVCRGRLRVPGQAEAPVAIKTLKAGY
SEKQRRDFLSEASIMGQFDHPSIIRLEGVVTKSKPVMIITEYMENGSLDGFLRGNDGQFS

VGQLVGMLRGIAAGMKYLSDMNYIHRDLAARNVLVNSKLVCKVSDFGLSRGLEDDPQAVY
TTHGGKIPIRWTAPEAIAFRKFTSASDVWSFGVLMWEVMSYGERPYWDMNNQDVIQSIEE
GYRLPEPMDCPPALHQLMLDCWQKERTERPNFAQILAYLDKLLQNPASLHAVAHPGSERL
SNPLLERKAPEYTMFCSVGEWLDDIKMGRYKENFTNAGLVSWPAVAQMTPEDLQKVNVTL
AGHQKRIMTSIQTLRSQLANGVRT
>Pmar_10282.1 P. marinus
CAACERGFYKEFAGDNSCSKCPPHSSARARGAASCSCEEGYFRPRTDPQSVACARPPSAP
GNVISNVNETSVTLEWSPPGETGGRRDVSYSIVCRRCPSEPAGACAPCGAAVRFLPRQAR
LLNTSVVVRDLLAHTNYTFEVHGNNGVSELSGVPSRFAAVNVTTNQAAPSPITDVQKGLI
SEDAIILNWAEPKQPNGIILDYEVKYYEKDQKESKYTTLRSNVTSTTITGLKPAVVYALQ
IRARTAAGYGDYSPAVEFKTSPDYVASPSEQKQDTLIWAFVFAVIVVLLLITAFFIIKRR
RCGYSKAKQDYDEEKTRFHSGHVKLPGLKMYIDPHTYEDPNQAVHEFAKEIDASCVKIER
VIGAGEFGEVCSGYLKHPGKKEVCVAIKTLKAGYTEQQRRDFLCEASIMGQFDHPNIIRL
EGVITKSKPVMIITEFMENGSLDLFLRKNDGQFTVIQLVGMLRGIAAGMKYLSDMNYVHR
DLAARNILVNSNLVCKVSDFGLSRVLEDDPEAAYTTRNEKTKHRHCNVEPQAFKTRMAIA
DTYWTKTYGIVMWRHVKYGERLYFNTSTHAQEVIKAIEEGYRLPAPMDCPLPLHQLMLDC
WQKERSDRPKFGQIVGILDKMIRNPNVLKTSASGDVTTRSANSLLDQSIPDFGSFRSVEE
WLDAIKMGRYKDNFASAGYTSLEQVVQLDSDDIVRIGITLIGHQKKILSSVQSMKADLVQ
LHNQGVHV
>Skow_11098 S. kowalevskii
TQARPRDMATTGFTFSPTSTLFLTVFLAVLTLTVCLQVPLYNSQDQRSLGWTTYHETSSV
QPEWTETGPSGGRRYQICRFANGGRQNNWLRTTWIDAKQANRVAVEIDFSMRECVDIPGA
VSCKETFDLYYYETDSDNADQNWPPWSSPPYNKVDRIAADGRFTHDTEVINTKTRHFGPI
TRNGFYLAFQDQGSCLAILKLKVYYLACQEITMNFAYFPKTPAGPKST
>Spur_027145 S. purpuratus
MDNLRNCIIFIVLCAWTSYAKEVKLYDSNAFSGLYWTVYPPQDPDSQTYVGWTESGASYE
RIFQTCHVSDPRDNWLRMPYIERQGANRIHVEVKFTMYSCTGIVDAQLCKETFDVYFYQS
DTDEATETSPDWTAPPYQKVARIAAEGRFSDPDTLDEEVVNVRIENFGPVTANGFYIAFR
DQGACMALLQVRIFYQVCPQVVRDFAIFNQTNEGPETHTLVTVPGTCVKNAQPVPPGSTP
QYICQNEGIWSLNQGGCGCSPGYEASADRTRCEECQIGTFKSDIGTTECSPCPAKSHADT
VAVTRCTCIPDYYRAPGEEASSPCTAPPSAPTSLTAVVDEKSSVVTLTWRNPENLGGRTD
IDFRIDCKRCPADVQFSSIQRIGQGYEVNVSRLSSHTTYEFKVHSYNEVSSQGTVDNYAG
VNATTFSSVPSAVRDLKVVEDDVNDESIKLRWRQPLFPNGEITNYQVIYRTSAETQENVK
YHYQAVLDGEVQETTVHGLVPGTEYVFEVAANTASGVGAYSQQVLATSSGSGGQSVPTVI
LAAAVSAIFIIVVLVAVVIFLVFWRRWKAKQQLYYQISTLPPRKKEVPPSENGHLIYANG
TNGEAITFGHSTVILPHIKKVKTYVDPFTYEDPNQAVKEFATEIDATQIRILEVIGGGEF
GDVCSGLMLMPDKTTIKVAVKTLKTGATDKDRSDFLSEASIMGQFDHPNVIKLLGVVTKT
RPAMIVTEFMENGSLDKYLKENDGRFTVTQLLGMMRGVGFGMRFLSEMNFVHRDLAARNI
LVNEHLVCKVADFGLSRRKELDGAYETRGGKIPIRWTAPEAIGYKKFTSASDVWSLGVVM
WEIMSYGERPYWNWPNQDVIKAVEKGYRLPPPMECPEAIHQLMLDCWQRDRNHRPTFTTI
VSTLDRLIRNPNALRPLAKNGSPLGLSSSLNDLSRFNSVSDWLDSLKMGRYKDSFTAAGY
VRLEDIARLSQSDLPRLGVTLAGHQKKIMKGIHSIRAQLEQAAETLV
>Ctel_198909 C. teleta
MRREGGGGRGSGFNATLLDMARPVIKTPGQRSCDVCGLMEGALKRRYALRVYTVCRVEAQ
QVNNWLRTPYIPRGTANRVYVEVTFTMRKCTKYHDPERLQQCKESFKLLYYEAESDFANS
MRPTWDESTYTLVDAIAADQVFTDLSNPVLNTEVRDLPITRRGLYLAFFDQGACTTLIRV
RLYYHMCPSVTKNFAVFPNRTTGQERTALEKVEGQCVEHAAMDKAPTYLCNSAGAWMYAQ
GGCKCMPGYQPYEDTACVACPVSTYKWRTGDDACRSCPAFSEAPEEGAVECECLTGYYRS
ASDDKAMPCSTPPSAVSNLSVTFFDQSLVNLSWTQPDQLGGRADLHYRVECSGCGANVKY
QPSKTGFNSTRISISGLTPDTRYKLYVYAENGVSKQSGSTVNSFVECFPAVVTEVRVVSV
GVNYITLGWSPPLKEPGVVIVLYEVRYSVHELNENSSAVDVGNRLNLTLSGLTPQTQYHF
QLCRMAYRVTFLXTRTQSDLASLGKSSDSPVGIIAGSAVAVVVCMVIVIIMVVIFLNRNR
VKTYIDPHTYEDPNQAVREFTKEIDASCIRIESVIGGGEFGDVCKGSLRVPGKGELTVAI

KTLKAGASDKNRLDFLTEASIMGQFDDPNVIYLEGVVTKNNPIMIIIEYMENGSLDSFLR
TNDGKFTIIQLVGMLRGIASGMRYLSEMGYVHRDLAARNILVNESFVCKVADFGLSREME
CDDTDGAYTTKARSLSGGRPLKRSRTASSRPPRPYWNWSNQDVIKAVDRGFRLPPPMDCP
EAIYQLMLDCWQKERSHRPKFNAIVKTLDKLIRAPELNGASVVIFRPHQLLDPNLPDLTN
YSSLEEWLQSIKMDRYTEVFVHAGFQGMEQVAQLTLADLLELGITLVGHQKKIMNSVQTL
RAQLYGAQVSHGFMV
>Lgig_105485 L. gigantea
WLQGSFNQDRRGYRVYTSCYVKNANVNNWLRTPYIERGDANSLHIEVKFTMRKCSRITDP
SSLQQCKETFNLYYYEADMDIANEMRPTWDAQTYTLVDKVPARHLYEQPTEIKINTETRR
IALRPNLRGVYFAFQDEGACVTLISIKVFYVVCPNITMNFASFPETPTGDSESSLVDRQG
QCVRNAIEMQPPTYLCRSDGSWYQDPKGQCLCMPGYEGTSDSTQCTACNKGMYKWSQGKE
ACKPCPHHSYANSPGAVECNCEHSYFRSPQDEKSMACTQPPSAPKNLILESMKSTTATLL
WEPPQELGGRTDLQYQVECRLCNSQVEARPGWRAFNTTRVTLKGLAPNSQYQIVIYAKNG
ASDISGHISSADITITTDANVKIINVRVISKDSNHITLTWGVQLPNGANTAVRNYEVKYF
PINSEGHHKIKTTTEPNFTLTDFGFDTEYFFQVRGYTDKGWGVYSDPLPVSTGNKGEYEL
KDPPIGIIAGSIVAVILCMAIATIMIIILLRRNRQNCNEKKECDTPYGHVTMPLFPSPGS
IHSSVRTYIDPHTYEDPNQAVREFTREIDISHITIESVLGGGEFGDVCKGKLRVPGRPEM
TVAIKTLKPGATEKNRLDFLTEASIMGQFDDPNVIFLEGVITKNNPIMIVTEFMANGSLD
TFLRNNDGKFTVIQLVGMMRGISSGMKYLSEMGYVHRDLAARNILVNESLVCKVADFGLS
REIETDNTDGAYTTKGGKIPVRWTAPEAIAYRKFTSSSDVWSYGVVMWEIMSYGERPYWN
WSNQDVIKAVEKGYRLPPPMDCPEATHQLMLDCWQRERSHRPKFFHIVKTLDKLIRAPEL
LRKMAKPR
>Tpol_63971 T. polymorphus
ATPMGIIAGSIVAVAVCISIVIIMIIIFWRRNNQGCSEKNPNDCENLQYTNRHTVHCHIE
PNQMNGSLTVPLFTSPGSVRSYVDPHTYEDPHQAVREFTKEIDASHITIESVIGGGEFGD
VCKGKLRMPGHPEMCVAIKTLKPGASEKNRLDFLTEASIMGQFEDANVIALEGVVTKSHP
IMIVTEYMENGSLDSFLRNNDGQFTVMQLVGMLRGIASGMKYLSEMGYVHRVSSR
>Tpol_7305 T. polymorphus
PLRPGSKQFTMERVCTFRIKALGLLWILTLLFSTNIADEAVLLDTTLETQDNLDWQAYPS
STGWQEASFDMGNDDLVRVNAVCQIDQAGSKNWLRTPFIPRGSAKRLYIEMKFTMRECSE
YPDPKSLQQCKETFNLLYYEAESDYANSVLPRWDTLTYRHIDVIAADSKFSHINNAVINT
EVRSVAINQKGVYFAFLDAGACTTLISVRIYYKKEKS
>Hpsi_102387 H. psittacea
MVVIYFKKNDRFCNEKHHGDCDTLHYSNVQCPPPIVMEIPHPFHGGLTVPLFAPAHHVKT
YVDPHTYEDPQQAVREFTKEIDASHITIESVIGGGEFGDVCKGKLIIDAKKIDMIVAIKT
LKPGASEKNRLEFLTEASIMGQFDDPNVIYLEGVVTKSNPIMIVTEYMQNGSLDTFLRTN
DGKLSAIQLVGMLRGISSGMKYLQEMSYVHRDLAARNILVNESLVCKVADFGLSREIEGD
NTDGAYTTKGGKIPVRWTAPEAITYRKFTSSSDVWSYGVVSWEVMSFGERPYWNWSNQDV
IKAVEKGYRLPPPMDCPEAMHQLMLDCWQKERGHRPKFAQIVKTLDKLIRSPELLRKIAK
PRQHNVLDPSVADMTRFKSIEEWLTNIKMERYLNDFLRAGHSTMEHVPRLTVDDLDRLGI
TLAGHQKKIMNSVQTLRAQISGAQVSEGFLV*
>Hpsi_234025 H. psittacea
GHYGRGKETLQLLYYEAGKQLTRDMPLWKIGLGYYSPITNKIAADYVFTTNDELLINTQT
RSLPLTMDGVYISLYDTGACSSIMYLRAYYIACPSVTVNFAHYAETPAHVNDQSIVEVAG
TCVPNAVI
>Dpul_79285 D. pulex
MAAARRSWVEESFTNFEKGINWRSYVVCDVAYNNVNNWLWTPFIERGEANRIYIEIKFSM
RDCSLFPGTALSCKETFSLLYYEFDAATREPPPWEPESYKLIDRIAADEGRFTSSNEVII
NTEVRSIPVTKKGVYFAFRDQGACISLLAIRVYYITCPEVTASFAAFPATPTGRELTSIE
QSNGVCVANAVEDAPPKYLCKGDGNWYLQGGGCLCKPGYEANADKQTCNVCPPGKYKHRS
GDDKCQTCPDHSMAPYSGSSECRCNQNYYRALKDPKSMPCTQSPSKPQNLTVNFVDQSTV
VLSWNQPHNLGGRSDTVYRVVCDACNVGVTYNPPQGTFNDTKVTISGLNPVTTYRFQVFA
DNGVSDQVPPNEHQYIDIAVTTEASVPSSVSNVRIINVRPTELTLRWDAPDDPYSDIEMY

EVRYFQKGFENNASSMLINRPESAFSSLRQQTVYGFQVRAKTTHGWGEFSSPAFKTTGAV
LATYEHTEENLQVRIIAGAVVAGIIIVVVIIVVTVLYLRRSNDECNKKQPSDCDTLEYRN
GEANAPRSYVDPHTYEDPNQAVREFAREIDAAYITIEAIIGGGEFGDVCRGNLKLPQRPE
MLVAIKTLKLGSSDKARVDFLTEASIMGQFEHSNVIYLQGVVTKSNPVMIITEYMENGSL
DTFLRANDGKFQVLQLLSMLRGIANGMQYLSEINYVHRDLAARNVLVNSQLVCKIADFGL
SREIESTTEGAYTTRGGKIPVRWTAPEAIAFRKFTSASDVWSFGIVCWEVMSYGERPYWN
WSNQDVIKSIEKGYRLPAPMDCPEAIHNLMLDCWQKERAHRPAFTAIVATLDKLIRCPEL
LQRLAQSRHTEPLCPEAPDMLQFKSIEEWLASLKLSRYVDNFQQAGVTTLDAVTRLSHAD
LSGLGIILLGHQRKIMNSIQAMRAQLSISMSEGFLV
>Lith_18666 Lithobius sp
LVSMLRGIAAGMQYLAEMNYVHRDLAARNVLVNANLVCKIADFGLSREIESTTEGAYTTR
GGKIPVRWTAPEAIAFRKFTSASDVWSFGIVAWEVMSYGERPYWNWSNQDVIKSIEKGYR
LPAPMDCPEAIHQLMLDCWQKERSHRPTFANIVKTLDRLIRCPESLRKVAQNKNQNPLAP
DAPDMTQFATVEEWLSSIKMTRYLESFERAGVTTMDAVARLTIKDLTSLGVTLVGHQKKI
MNSVQTMRVQISANMSEGFLV*
>Lith_21051 Lithobius sp
AAGLVLASLLVLDITRIQLVNGKEVVLLDTTQEAALEWTRYPYGPQALTQGWVEESFTDF
DKGINWRSYVACDVAYDNVNNWLWTPFIERGEANRIYIEIKFSMRDCSLFPGTALSCK
>Lith_3827 Lithobius sp
GTALSCKETFSLLYYEFDAATKEPPPWEPESYKLIDRIAADEGRFTSSNQIIINTEVRSV
PVTKKGIYFAFRDQGACISLLAIRIYYITCPNVTTSFAYFPETPTGREVTSIEQAEGQCV
PSAAQEETPKFLCKGDGNWYLLSGGCKCMAGYEANLDKQSCEVCSIGKFKYSVGDERCQP
CPEHSTAPYSGSVECRCNEGYHRALKDPKSMPCTQPPSAPQNLSVSFVDQSTVILSWNAP
SYLGGRTDTMYRVMCNACSHAVTYVPSQTSFNDTKVTISGLNPVTTYRFLVYAENGASGF
SGNSQNVDITVTTEASVPSLVSNVRVTNVKNTEITLAWDPPNDPFSEIEMYEVRYFVRGL
ERNATTVLTKHEESQFSNLKQRTEYGFQVRTKTTHGWGEFSLPVFKTTGQMLAYVVSDDD
NLQVRFIAIAVVAVVVLLFIVIIVTVLFLRRSNDDCNKKQPSDCDTLEYRNGEVTTPLFT
QVGPPRTYVDPHTYEDPNQAVREFTREIDASHITIEAIIGGGEFGDVCRGKLKIPSRPEM
TVAIKTLKPGSSDKARMDFLTEASIMGQFEHPNVIFLQGVVTKSNPVMIITEYMENGSLD
TFLRANDGKFQVIQLVSM

Additional bilaterian Eph receptors that included only in the supplementary tree and not elsewhere in the manuscript See Supp_tree_additional_Eph_tyrosine_kinase_domain_complete_mining_tree_file and Supp_tree_additional_Eph_LBD_complete_mining_tree_file. The domains were chopped and used for these trees. Trees can be found in the additional file 3

>ACYPI064034-PA pep supercontig:Acyr_2.0:GL350049:209653:345030:1 gene:ACYPI064034 transcript:ACYPI064034-RA gene_biotype:protein_coding transcript_biotype:protein_coding
MHKYHQQHHHDDSNHHHHRHNHHYLNRNHRCCLTAVFLLLAVAIVSPGRAEHVVLLDTTT
EPSLRWTTYPYGPDANAAGWVEESYINFEKGINWRSYVVCDVTKQNVNNWVWTPFIERGL
ANLIYIEVKFTIRDCSLFPGYALSCKETFSLLYYEFDVATREPPPWEPDSYKSVGRIAAG
EGRFNANNEVVINTETKAVKVTKKGVYFAFRDQGACISIMAVKVYYIVCPEVVINFANFS
ATPTARELTQIEHATGKCVDNAEVVGGGAPTYLCKGDGKWYLPSGGCKCKAGFEADIEAQ
TCIICPPGKYKYGVGDDKCQPCPAHSKAPDQGMSECRCNTGYYRSPKDPKSVPCTQPPSA
PQNLTVNFVDQSTVTLSWNPPNFLGGRTDIVYRVTCDMCGPSVLFMPNNEVFNDTKITIS
GLSPVTTYKFHVWAENGVSNLTSSENRQFVDIAVTTTEASVKSASVNNVRVMLVKASEIT
LSWDPPMASFFDSGDDDAAVEVYEVKFYPRGDESNVSNKLTADRHMVFTALRPKTDYGFQ
VRAKTAHGWGEYSPTIYKTTGQLLGSAYIGDEDNMEVRIIAGATVAVVVVLVVVIIMTVL
FLRSRSNDECNKKQPSDCDTLEYRNGEVTTPLFTQVGSTTSRSYIDPHTYEDPNQAVKEF
AREIDASYITIEAIIGGGEFGDVCRGKLKVLGSPSVEVDVAIKTLKPGSTDKARNDFLTE
ASIMGQFEHPNVIFLQGVVTRSNPVMIITEYMENGSLDTFLRANDGKFQVLQLVGMLRGI
ASGMQYLSEMNYVHRDLAARNVLVNAQLVCKIADFGLSREIESTTEGAYTTRGGKIPVRW
TAPEAIAFRKFTSASDVWSFGIVVWEVMSYGERPYWNWSNQDVIKSIEKGYRLPAPMDCP

ETIYQLMLDCWQKERTHRPMFMSIVKTLDKLIRCPDTLRRIAQNRSVNPLSSDAPDMTQF
GSVNEWLCSIKMARYLDNFEQAGIVSPKAVARLTVADLTALGITLVGHQKKIMNSIQAMR
AQFSANLSEGFLV
>ADAC004174-PA pep supercontig:AdarC3:scaffold_829:37111:44154:1 gene:ADAC004174 transcript:ADAC004174-RA gene_biotype:protein_coding transcript_biotype:protein_coding description:Eph receptor tyrosine kinase [Source:LNCC;Acc:AD04174]
MDKHLSLLLLLCYALYQRLDVVHGDQVVLLDTTKEATLEWTRYPYGPQAQTPGWVEESFT
NFVKGINWRSYVVCDVAYNNVNNWLWSPFIDRGPANRLYIEIHFTIRDCSLFPGNALSCK
ETFSLLFYEFDAATREPPPWQPESYKLIGRIAAGEGRFNQNSDVDINVEVKSIAVTKKGV
YFAFRDQGACISVLAVKVYYITCPAVTINFAHFNETPTGREVTIIEQQMGVCVENAEAFE
QPTYLCKGDGKWTILSGGCRCKVGYEPDNEKQTCNVCPVGKFRSAEVKACTICPLNSKSI
KIGSPYCPCLNGHYRHPRDGKHMPCYKPPGMPTNLTLLFIDQTSAILSWNAPQRAPDEQL
DPVFRSDIVFRVKCAACTSNVVFNPSSETFNDTKLTLTNLEPVTTYTVQIHSQHGVSYPI
VNPEAPGTGGTGGMYENVSTVGYGGHYGYHATEPTPVVNVRAASDLDEIKTEFAEITFTT
ESAILSTVFNVKVIQITNKDADLVWDKPMHSDSPIEYYEVRWFPKSEVDAMNKSVLSTKE
SKVHIGDLLENTEYGFQVRCKTLNGWGTFSNIVYAQTHQSVSPVYDDSFQMRIVAGTTVM
VVVLLVLVIVVTVVFLKSKNHDDIDKKTNNHLPLPLDYASNEVHAMDTTPIVKTMKSNVT
TPLFGTSRSYVDPHTYEDPNQAIREFAREIDASYITIEAIIGGGEFGDVCRGRLKVPPNF
VQEIDVAIKTLKPGSSEKARCDFLTEASIMGQFEHPNVIFLQGVVTRSNPVMIITEYMEN
GSLDTFLRANDGKFQTIQLIGMLRGIAAGMTYLSEMNYVHRDLAARNVLVNSLLVCKIAD
FGLSREIENASDAYTTRGGKIPVRWTAPEAIAFRKFTSASDVWSYGVVLWEVMSFGERPY
WNWSNQDVIKSIEKGYRLPAPMDCPEALYQLMLDCWQKQRTHRPTFASITQTLDNLARQP
QVLLTTRNSPDNPVARLGSTGIVDDMSQDMMVTGGAGGIGNSAQQQHQQQQQQQQQQQQQ
QQQQQQQQQQQLQQQQRVGGMLLGGTSERVLNSSGGANVVSSGMGTLGSSLGGLSNTGTM
TGASAPAIFINTDLWLESIKMSRYSQHFKEAGLVTAQQLSRLTAQQLSDMGITLVGHQKK
ILHQARQIDTII
>AGAP000489-PA pep chromosome:AgamP4:X:8635241:8667713:-1 gene:AGAP000489 transcript:AGAP000489-RA gene_biotype:protein_coding transcript_biotype:protein_coding description:Eph receptor B1 [Source:VB Community Annotation]
MERFLSLLLLCYALYQKFDGVVSDQVVLLDTTKEATLEWTRYPYGPQAQTPGWVEESFTN
FVKGINWRSYVVCDVAYNNVNNWLWSPFIDRGPANRLYIEIHFTIRDCSLFPGNALSCKE
TFSLLFYEFDAATREPPPWQPESYKLIGRIAAGEGRFNQNSDVDINVEVKSIAVTKKGVY
FAFRDQGACISVLAVKVYYITCPAVTVNFAHFNETPTGREVTIIEQQMGVCVENAEAYET
PTYLCKGDGKWTILTGGCRCKVGYEPDAEKQTCNVCPVGKFRSAEVRTCTICPLNSKTNK
VGSPYCPCLSGHYRHPRDGKHMPCYKPPGPPTNLTLLFIDQTSAILSWNAPQRAADEQLD
SKFRSDIVFRVRCAACTSNVVFNPSSETFNDTKLTLTNLEPVTTYTVQVHSQHGVSYPIN
GSAVVAGGSGYHATEPPMVVMVNARAGGPGSSSDLDDIKTEFAEITFTTESAILSTVFNV
KVVQITNKEVDLVWDKPIHSDSPIEYYEVRWFPKSEVDAMNKSVLSTKESKVHIGDLLEN
TEYGFQVRCKTLNGWGTFSNIVYAQTHQSVSPVYDDSFQMRIVAGTTVTVVFILVLVIVI
TVVFLRSKNHDEIDKKTNNHLPLPMDYASNEVHAMDTTPIVKTMKSNVTTPLFGTSRSYV
DPHTYEDPNQAIREFAREIDASYITIEAIIGGGEFGDVCRGRLKVPPNFVQEIDVAIKTL
KPGSSEKARCDFLTEASIMGQFEHPNVIFLQGVVTRSNPVMIITEYMENGSLDTFLRAND
GKFQTIQLIGMLRGIAAGMTYLSDMNYVHRDLAARNVLVNSLLVCKIADFGLSREIENAS
DAYTTRGGKIPVRWTAPEAIAFRKFTSASDVWSYGVVLWEVMSFGERPYWNWSNQDVIKS
IEKGYRLPAPMDCPEALYQLMLDCWQKQRTHRPTFASITQTLDNLARQPQVLLTTRNSPD
NPVARMGTAAGGVGDDLTQEMLAAAAAAAAAAAAAAGSGGMVGVMGGSSQQQQQQQQQQ
QQQQQRAATLMMGGGSGTSERVLNTGSSMTNVPGSMGTLGSTVGGLSNTGTMSGPGNPGI
FISTDLWLEGIKMSRYSQHFKEAGLVTAQQLSRLTAQQLSDMGITLVGHQKKILHQARQI
DTII
>AGLA005888-RA pep supercontig:Agla_1.0:KB933694:392464:415205:-1 gene:AGLA005888 transcript:AGLA005888-RA gene_biotype:protein_coding transcript_biotype:protein_coding
RIAAGEGRFNSNSEVNINTEVKSIPVTKKGVYFAFRDQGACISLLAIKIYYIVCPEVTVN
FARFPSTPTGKEIVIIEQATGTCVSNAEVVGGTPTYLCKGDGKWTLPTGGCKCKAGFQPD

MEKQTCNVCQRETYKAETGDGPCLPCPPHSQGPDYEPPSAPQNLTVTFVDQSNVALSWLQ
PENLGGRSDTVYRIKCDACSLGPVQYNPNTETFNDTRITISGLNSVTTYRFQIFAENGVS
HLQLKSSPEYADIVVTTEALVASSITNIRVLSVRWFQRNDIDYSNSTSLLTADLSATITG
LQQRTEYGLQVRAKTQRGWGAYSPVIFKTTGQVLNTGKQKRPARRTSGVTRGVFLAYIGD
EEGLRLQLVAGGIVAVVVILVAVIVLTVVFLRSRSNDECNKKQPSDCDTLEYRNGEVHHS
LDNPPIVTTHTNVTTPLFTGISGSSRTYIDPHTYEDPNQAVREFAREIDASCITIEAIIG
GGEFGDVCRGKLKMNGVDIDVAIKTLKAGSLDKSRNDFLTEASIMGQFEHPNVIFLQGVV
TKSNPVMIITEYMENGSLDTFLRANDGKFQVIQLTGMLRGIASGMQYLSEMNYVHRDLAA
RNVLVNSQLVCKIADFGLSREIESATEGAYTTRGGKIPVRWTAPEAIAFRKFTSASDVWS
MGIVCWEVMSYGERPYWNWSNQDVIKSIEKGYRLPAPMDCPEAIYQLMLDCWQKERTHRP
SFQSIVKTLDKLIRVPDTLRKIAQNRSTNPLSADAPDLTNFTSVEEWLNSIKMARYLENF
HAGGINTMDAVVNLTVKELTELGITLVGHQKKIMNSVQNIRAQIRVNGSEGFLV
>BGIBMGA002079-TA pep supercontig:ASM15162v1:scaf23:497313:501749:1 gene:BGIBMGA002079
transcript:BGIBMGA002079-RA gene_biotype:protein_coding transcript_biotype:protein_coding
MLTVRRCKSQITNWGIFFEPVANPLHEFQAPSVEVHVGYEHVNGNALSCKETFSLLYYEF
DVATREQPPWEPESYKLVGRIAAGEGRFNTNSEVDINTEVKSIAVTKKGVYFAFRDQGAC
ISILAVKVYYITCPDVNINFARFPATPTGREVTVIEQANGTCVANAEVAPGEKAPVYLCK
GDGKWTLPSGSCKCRAGFEPDHNNQICTECAPGKFKSHTGDEHCIPCPDHSESTIYAAAE
CKCTPKYYRAKKDPKTIPCTQPPSAPDNLTVTFADQFSISLTWQPPHNTGGRSDITYKIS
CANCGPTVEFRPGETGLNSTRVTISGLDPVTEYKFQVYAENGVSELSPDPANHIEITAVT
EASVVSVVSKLRILNTESDRLTIAWNPPQVDLTDPDDTIESYEVKCFPKDSQDKNTNTTL
RITKDPQVTITGLRKDTEYGIRVRAKMKRGWGEFSGIVYARTNSVTETSFIDEEGAQVRL
VAGVMVAVVVLTVIAIIATVLFLRSRADDECDKKQPSDCNALNYRNGEVYTGPDRPAKTS
SNATTPLFAGTGSRTYIDPHTYEDPNQAVREFAREIDASCITIEAIIGGGEFGDVCRGKL
KLPASCGPEIDVAIKTLKPGSTERARRDFLAEASIMGQFEHPNVIFLQGVVTKCNPIMII
TEFMENGSLDTFLRANDGKFRVLQLVGMLRGIATGMQYLSEMNYIHRDLAARNVLVNSQL
VCKIADFGLSREIESTADGAYTTRGGKIPVRWTAPEAIAFRKFTSASDVWSMGIVCWEVM
SFGERPYWNWSNQDVIKSIEKGYRLPAPMDCPEAVYQLMLDCWQKERTHRPTFSSIVKTL
DKLIRCPDTLRKIAQNRSADPLAGDTPDLIQFTSVEEWLECIKMSRYIEKFRAAGISDMD
AVVDLTVHQLASLGVTLVGHQKKIMNSVQSMRAQIRGSGPYGFLV
>BIMP12060-PA pep supercontig:BIMP_2.0:JH158802:621881:715610:-1 gene:BIMP12060 transcript:BIMP12060-RA
gene_biotype:protein_coding transcript_biotype:protein_coding
MLVCIGAGNDEEEKKMIALTTKNRTSRFTFNKPSQSSSNSGKIDEITIALAMVSLLGPTV
ASPFNQRSSSLIAVVLLDTTQEEKLEWTKYPFGAEANTPGWVEESFTNFDKGINWRSYVV
CDVAYNNVNNWLWTPFIERGPANRMYIEIQFTTRDCSLFPGNALSCKETFSLLYYEFDVA
TKEPPPWETDSYKLIGRIAAGEGRFNTNTGVVINTEVKSIPVTKKGVYFAFRDQGACISI
LAIKVYYISCPEISVNFAHFPATPTGREVALIEQTIGTCVDNAVVIEQPTFLCKGDGKWY
LPNGGCHCKPGYQADVEKQACTECAIGKFKHEAGSHSCEACPAHSKSSDYGFTECRCNAG
YFRAEKDPKKMPCTQPPSAPQNLTVNFVDQSTVILSWNAPHMLGGRTDTTYRVVCDACSM
GVKYIPNTEVFNDTKITITGLNAVTTYRFQVFAENGVSALAGKSEYVDITVTTEASVPSL
VSNVRITSVKSSELSISWDAPVTEVGGDSDLVERYEVRCYPRYDDATNATVIQTSELSAT
FKGLKPSTDYAIQVRAKTTRGWGEYTPIVYKKTPHAMGLDYVGEDDNMQVRIIAGAIVAV
VVLLVIIIMTVLILRSRASDECNKKQPSDCDTLEYRNGEVHCKMDSSPIVTTHTNNKSK
SSLTTPLFTPAVGVAAASAGGAGGGGARSYVDPHTYEDPNQAVREFAREIDAGYITIEAI
IGGGEFGDVCRGKLKLPPDGRTEIDVAIKTLKPGSADKARNDFLTEASIMGQFEHPNVIF
LQGVVTKSNPVMIITEFMENGSLDTFLRANDGKFQVLQLVGMLRGIASGMQYLAEMNYVH
RDLAARNVLVNAALVCKIADFGLSREIESATEGAYTTRGGKIPVRWTAPEAIAFRKFTSA
SDVWSMGIVCWEVMSYGERPYWNWSNQDVIKSIEKGYRLPAPMDCPEAIYQLMLDCWQKE
RTHRPTFANLTQTLDKLIRSPDTLRKIAQNSVRPPAHNPYYVTSHAAAAQAAAATAAIPA
ATTGPFVDVVGHQAHQAQSTIAVPVMPPGAGRSLHYHTHAATHALPQQPPLTYPNWVPFT
HFG
>Bm5071 pep supercontig:Bmal-4.0:Bm_v4_Chr2_contig_001:5445833:5472991:1 gene:WBGene00225332
transcript:Bm5071 gene_biotype:protein_coding transcript_biotype:protein_coding gene_symbol:Bma-vab-1

MQPPVNSPVLSPTVTVKLISFSIHQLLLLFTLWSMVTSKKVILLDTSNSTTELRWQTHSN
MDDDEIGWLEETYRGPDGIDNRRAYVVCNVDQQNVDNWLRTPIIHVNGANRLHVEVTFTM
RDCSEFPGNARSCKETFRLYGVQVMNNEKYQNIWNSDYWDLIDRITADTGRYSKSDPTTA
AVNQEIRSYTVTKDAVYFAFRDSGACISILNVKIFYEVCPEITRAFVHYPQTVTGAEAHS
IIAVNGKCVPNSSPIDSIKQPTYVCKATGSWDMANGECYCDKGYASSIKYNTCTACSIQS
YKAKNGLGDCESCPGHSSTRHIGSAECQCDAGYYRADDEGPEFSCTQPPSKPSHVVVARI
DDTSVIIEWDEPLVLGGRKELWYRYQCPECPSTTIAHPAGKTFTTQRLQLTALKAGTTYT
VLIFAENKVSKVESVVSQYALVEFTTRTTVPVVNGLRIESIQENGVTIAWKPIGTMKNEL
SYEIESLHNDSSAVVKTTRSYYTFESLKPQFMYSFRVRIVNEHGYGTWSEPLWYQTGHGL
PFAPISHHVDDDDIDINDEYMHNNSIVDLHWSSPGPPLWIWVLLICTFVVIVLLTLLICL
RQNRNRKRLSDCDGIDSYKNGTLTPDFSAPVSQGHVQNFFRGKLSSPLINYGSSQSTNYG
ETITRFKPYVDPAAYEDPNQALLEFTNDVDPALIRITEIIGSGEFGEVCKGILQPSYRMT
SSDICQVQTVAIKTLKPGSSDKAKGDFLMEASIMGQFTHENVIRLIGVVTKNEPIMIVIE
YMENGSLDQFLRKNDNGVLKLMQIIEMLRGIAAGMKYLTEKGFVHRDLAARNVLVDSNLL
CKIADFGLSRGVEGSVEQEYTTNGGKIPVRWTAPEAITHRKFTAASDVWSFGVVMWEVCS
FGERPYWDWTNQKVISEITLGYRLPSPMDTPISLHNLMLQCWHIDRHKRPTFAQILKILE
EYVRQPALIYSDGIYLDNTTLSNVGFSTFGTLPRTLDAEISAASLSLSSQLSLDEFLKRI
GLGHCALKLNMAGVSRVSDLIRLGHIDLLSYGLIAEEVQMIRNALKRLCPTTTLQHMRNR
PEHQRLPPPHHFSPQLHSPVRTSSRLATMPRVLSSISTSNKDSFFV
>CBG13447 pep chromosome:CB4:II:2740753:2763473:-1 gene:WBGene00034210 transcript:CBG13447
gene_biotype:protein_coding transcript_biotype:protein_coding gene_symbol:Cbr-vab-1
MRLYNSRILNPFYSIFLLQLFVIEFVNSHQEVLFDLSKATPATRWDSLALKHDRDDVWME
ETWRNPAATDEKHANQRAYVTCNYDMDQPSNWLFSHFIEVQSARRIYVELLFNIRDCLGF
TDPKSCKETFTVFLKQLKTSHPGSAKIEREQFAKDMKNWQNIGRMARSNSNTTTETIGFE
IEPDTKMIRIAFEEQGICLSLLNVKIYYRVCDEFTDQLVQFPAQVTGAKETDMVRMNGTC
IPNASRKIAGVDLIGLCMATGSAIKTSGECVCDSGYSEIADSNGARCESCPPNTYKPKGQ
SMCKPCPANSVSGEAASSCRCVNGYFRADDEMVSQSCTQPPSRPIKLVANAITATSTRLS
WNEPSSLGGRPEIWYEVRCSGRGECGSVVMTPSDKRLSTRSIQINGLRPSSDYTFLVFAK
NKVSGELAEFSEKSAVIDIRTRSEEEDVPPVTHLRVDASQSDGITIAWSVSDSDIHDFEV
EVRPAIVKKRAFETRHVNQTYTTFIGLRHDTVYQFRVRVKDDLRWSSPISYQLGKGLLSS
SSSTSEEDTQFLNQTGSALLIIIALILVVIAIALCMIVVQKKTKNRKQMSDLDVLDTYKQ
DSMTPDYHTTSRHHHHQGNLPANLHEQLRSTTKLNAPLIPSFGSPISQPPPYYGGVHTNS
GKYKTYVDPTTYEDPYQALIEFTFDISPNDVFITQVIGGGEFGDVCLGGLSRNSPAAAKW
NVASTMARGFESEQYETVAIKTLKSGSSAKAKAEFLTEATIMGQFSHPNVIRLIGVVTSS
EPVMIISEYMVNGSLDQYLRNADQRGDKVHWEKITEMLYGIASGMKYLTDMGYVHRVSWI
SDLAARNVLLDHELRCKIADFGLSRGVRSEGSSIEPEYTTNGGKIPVRWTAPEAITHRKF
TPSSDVWSFGVVIWEVCSFGERPYWDWTNQKVISEVMIGYRLPPPMDCPMGLYRIAQWCW
KMERHERPTFTQLLAAFHKYILQPTLIETDPGELPRRVMSQAALNSMNPSYGSVSMPTPP
SSAAPMPSLDDFLRQIGLNHVYGKLVSNNIHSVSDLANTSHLDLLACGLISPECSIVRDG
LNGRIVTSSGSPGNSSGTLHATTRGTRTTRPREEGFFV
>CBN08102 pep supercontig:C_brenneri-6.0.1b:Cbre_Contig330:44170:60224:1 gene:WBGene00146827
transcript:CBN08102 gene_biotype:protein_coding transcript_biotype:protein_coding gene_symbol:CBN08102
MRLPNSRILNPNLLITIILSHLIRIVASHQEILFDLSKTGSDLKWDQLSLRHDRDDVWME
ETWRNPAATDEKHANQRAYVTCNYDMINPSNWLFSHFIDVQSARRVYIELLFNTRDCDAY
TNPKSCKETFSVYLRQFKTARPGTSKMEKERFSSDIDNWKNIGRLARSNSNMTTETIGME
IEPDTKMIRIAFEEQGICLSLLNVKIYYRVCDEFTDQLVHFPAQVTGQKDNDIVRMNGTC
IPNASKKIPGVDLIGLCTPSGTTMRTSGECVCDSGYSEIADSNGARCESCPPNTFKPKGQ
TMCKPCPANSISSEAASSCRCVTGYFRAEDEIVSAPCTQPPSRPIKLVANAITASSARLS
WNEPSSLGGRPEIWYEVRCSGRGECGSVVMTPADKKLSTRSVQINGLRPSSDYTFLVYAK
NKVSGQYPEFSEKNAVIDIRTRSEEEDVPPVSHLRVDATQSDGITIAWSVSDTEISDFEV
EVRPAVIKKRAFETRHVNMTYTTFIGLRHDTVYQFRVRIRDDLRWSQPISYQLGKGLLSA
PATEISESQFLNQTGSALLIIIALILVVIAVALCMIVVQKKSKNRKQMSDLDVLDTYKQV
NAPLIPSFGSPISQPPPYYGGVTSGKYKTYVDPTTYEDPYQALIEFTFDISPNDVFITQV

IGGGEFGDVCLGGLSRSSPAAAKYNVANNTMGRGFESEQYETVAIKTLKSGSSAKAKAEF
LTEATIMGQFSHPNVIRLIGVVTSAEPVMIVAEYMSNGSLDQYLRNADQRGEKVHWEKIT
EMLYGIASGMKYLTDMGYVHRDLAARNVLLDQELRCKIADFGLSRGVRSEGSIEPEYTTN
GGKIPVRWTAPEAITHRKFTPSSDVWSFGVVIWEVCSFGERPYWDWTNQKVISEVMIGYR
LPPPMDCPMGLYRIAQWCWKMERHERPTFTQLLATFHKYILQPNLIEQDPGELPRRVMSQ
AALYGAVPNPPSTAAPMPSLDDFLRQIGLNHVYGKLVSNNIHSVSDLANTSHLDLLACGL
ISPECSIVRDGLNGRISSSSSTPPGSSGTIHATTRGTRTTRPTREEGFFV
>CBN17142 pep supercontig:C_brenneri-6.0.1b:Cbre_Contig60:557855:575625:1 gene:WBGene00155867
transcript:CBN17142 gene_biotype:protein_coding transcript_biotype:protein_coding gene_symbol:CBN17142
MRLPNSRILNPNLLITIILSHLIRIVASHQEILFDLSKTGSDLKWDQLSLRHDRDDVWME
ETWRNPAATDEKHANQRAYVTCNYDMINPSNWLFSHFIDVQSARRVYIELLFNTRDCDAY
TNPKSCKETFSVYLRQFKTARPGTSKMEKERFSSDIDNWKNIGRLARSNSNMTTETIGME
IEPDTKMIRIAFEEQGICLSLLNVKIYYRVCDEFTDQLVHFPAQVTGQKDNDIVRVNGTC
IPNASKKIPGVDLIGLCTPSGTTMRTSGECVCDSGYSQIADSNGARCESCPPNTFKPKGQ
SMCKPCPANSISSEAASSCRCVTGYFRAEDEIVSAPCTQPPSRPIKLVANAITASSARLS
WNEPSSLGGRPEIWYEVRCSGRGECGTVVMTPADKKLSTRSVQINGLRPSSDYTFLVYAK
NKVSGQYPEFSEKNAVIDIRTRSEEDDVPPVSHLRVDATQSDGITIAWSVSDTDISDFEV
EVRPAVVKKRAFETRHVNMTYTTFIGLRHDTVYQFRVRIRDDLRWSQPISYQLGKGLLSS
PATDISESQFLNQTGSALLIIIALILVVIAVALCMIVVQKKSKNRKQMSDLDVLDTYKQD
SMTPDYHTTSRHHQGNLPANLHEQLRSTTKLNAPLIPSFGSPISQPPPYYGGVTSGKYKT
YVDPTTYEDPYQALIEFTFDISPNDVFITQVIGGGEFGDVCLGGLSRSSPAAAKYNVANN
TMGRGFESEQYETVAIKTLKSGSSAKAKAEFLTEATIMGQFSHPNVIRLIGVVTSAEPVM
IVAEYMSNGSLDQYLRNADQRGEKVHWEKITEMLYGIASGMKYLTDMGYVHRDLAARNVL
LDQELRCKIADFGLSRGVRSEGSIEPEYTTNGGKIPVRWTAPEAITHRKFTPSSDVWSFG
VVIWEVCSFGERPYWDWTNQKVISEVMIGYRLPPPMDCPMGLYRIAQWCWKMERHERPTF
TQLLATFHKYILQPNLIEQDPGELPRRVMSQAALYGAVPNPPSTAAPMPSLDDFLRQIGL
NHVYGKLVSNNIHSVSDLANTSHLDLLACGLISPECSIVRDGLNGRISSSSSTPPGSSGT
IHATTRGTRTTRPTREEGFFV
>CJA06042 pep supercontig:C_japonica-7.0.1:Cjap.Contig17207:85009:107923:1 gene:WBGene00125246
transcript:CJA06042 gene_biotype:protein_coding transcript_biotype:protein_coding gene_symbol:Cjp-vab-1
MLNPFLQLCILLFFTANTVTTHQEVLFDLSKVGSDLKWDQVSLRHDRDDVWMEETWRNPA
ATDEKHANQRAYVTCNYDMINPSNWLFSHHIPTKNARRVYIELLFNTRDCDAYTNPKSCK
ETFSVYLKQFKSSIPGSTRIEKEKFAEDVDNWKNIGRLARSNSNMTTETVSMEIAADTKL
VRIAFEEQGICLSLLNVKIYYRVCDEFTDQLVYFAPQVTGPKATDIVRMNGTCIQNASKK
IQGVDLVGLCMSTGNAIKTSGECACDSGFSQIADSNGARCESCPSNTYKPKGQTMCKPCP
QNSVSSEAASSCRCVNGYFRAEDELVSMPCTQPPSRPVKLVANAVSATSARLSWNEPSSL
GGRAEIWYEVKCSGKEECGNVVFTPSDKRLSTRSVHVNGLRPSSDYTFHVVARNKVSAQY
TAYPEKNAILDIRTRSEEEDVAPVSHLRVDASQSDGITIAWSVADSDVIDFEVEVRPAVI
KKRAFETRHVNMTYTTFIGLRHDTVYQFRVRVRDDLRWSEPISYQLGRGLVSSQSSMDGG
VDAGESQFMNQTGSALLIIIALILVVIAVALCMIVVQKRSKNRKQMSDLDVLDTYKQDSM
TPDYHTTSRHQANLPAALHEQLRSTTKLNAPLIPSFGSPISQPPPYYGGIHTNSGKYKTY
VDPTTYEDPYQALIEFTYDISPNDVFITQVIGGGEFGDVCLGGLSRNSPAAAKWAANSST
MGRTEQFETVAIKTLKAGSSGKAKAEFLTEATIMGQFSHPNVIRLIGVVTSSEPVMIVAE
YMANGSLDQFLRNADQRGEKVHWEKISEMLYGIASGMKYLTDMGYVHRDLAARNVLIDTE
LRCKIADFGLSRGVRSEGSVEPEYTTNGGKIPVRWTAPEAITHRKFTPSSDVWSFGVVIW
EVCSFGERPYWDWTNQKVISEVMIGYRLPPPMDCPMGVYRIAQWCWKMERHERPTFTQLL
AMFHKLIVQPNLIEHDPGELPRRVVSQGALNTYGAIGVPTPPSTAPPMPSLDDFLRQIGL
NHVYGQLVSNNVHSVADLANTSHLDLLACGLISQECSTIRDGLNGRISATSTPSSGGGTI
HATTRGTRTRPREEGFFV
>CPIJ014812-PA pep supercontig:CpipJ2:supercont3.649:177378:187910:-1 gene:CPIJ014812 transcript:CPIJ014812-RA
gene_biotype:protein_coding transcript_biotype:protein_coding description:Eph receptor tyrosine kinase
[Source:UniProtKB/TrEMBL;Acc:B0X7T1]
MTKTRGCVPKQHEEAQTALPFFPSGSLKAAAAPHNVAQKPQTPTSEEERFAVCWVRELGW

GVDSVQDIHVHGETLCELNPIIMAQGSVPTTTMMKLLFLRGRRRGHHQLLICILQPHPIQ
WVEESFTNFVKGINWRSYVVCDVAYNNVNNWLWSPFIDRGPANRLYIEIHFTIRDCSLFP
GNALSCKETFSLLFYEFDAATREPPPWQPESYKLIGRIAAGEGRFNQNSDVDINVEVKSI
AVTKKGVYFAFRDQGACISVLAVKVYYITCPAVTVNFAHFNETPTGREITIIEQTTGTCV
ENAESYESPTYLCKGDGKWTILSGGCRCKVGFEPDHEKQTCNVCPVGKFRSAEVDACTIC
PLNSKTNKIGSPFCPCLSGHYRHPRDGKHMPCYKPPGPPTNLTLLFIDQTSAILSWNAPQ
RAVDEQLDTKYRSDIVFRIKCSACTSNVVFNPSSETFNDTKLTLTNLEPVTTYTVQIHSQ
HGVSYLLVQESASDKAGAAGYDNTSFNNHYHHATEPPNVRSSSDLDDIKTEYAEITFTTE
SAILSTVFNVKVISITNKEVDLVWDKPMHADSPIEYYEVRWFPKSEVDAMNKSVLSTKES
KAHIGDLQENTEYGFQVRCKTLNGWGTFSNIMYAQTHQSVSPVYDDSFQMRIVAGSTVAV
VFILVLVIVVTVLFLRSKSHDDIDKKTNNHLPLPLDYASNEVDDTCQLVFWQRDRVATVR
CCCGQLPKQN
>CRE17286 pep supercontig:C_remanei-15.0.1:Crem_Contig64:28215:55387:1 gene:WBGene00065095
transcript:CRE17286 gene_biotype:protein_coding transcript_biotype:protein_coding gene_symbol:Cre-vab-1
description:CRE-VAB-1 protein [Source:UniProtKB/TrEMBL;Acc:E3MRW9]
MCSKYVDFYQFFLSIPEVLFDLSKQPSDLKWDQVSLRHDRDDVWMEETWRNPAATDEKHA
NQRAYVTCNYDMINPSNWLFSHFIEVQSARRVYIELLFNTRDCDAYLNPKSCKETFSVYL
KQFKTSRPGATKIEKDRFEKDIDNWKNIGRLARSNSNMTTETIGMEIEADTKMIRIAFEE
QGICLSLLNVKIYYRICDEFTDQLVHFRPQVTGPKETDMVRMNGTCIPNASRKITGVDLI
GLCMSTGSGIKTSGECVCDSGYSQIADSNGARCESCPPNTYKPKGQSLCKPCPVNSISGE
AASSCRCVNGYFRAEDELVSQPCSQPPSRPIKLVATAITASSARLSWNEPSSLGGRPEIW
YEVRCSSSARGECGSVVISPADKRLSTRSVQINGLRPASDYTFLVFARNKVSSEIGGGAE
KSAVIDLRTRSEEEDVPPVSHLRVDASQSDGVTIAWSTADSDVTDFEIEVRPAIVKPRAF
ETRHVNMTYSTFIGLRHDTVYQFRVRVKDDLRWSQSISYQVGRGLVSSAPSEELGESQFL
NQTGSALLIIIALILVVIAVALCMIVVQKKSKNRKQMSDLDVLDTYKQDSMTPDYHTTSR
HHHQHGNVPANLHEQLRSTTKLNAPLIPSFGSPISQPPPYYGGVHSGTSGKYKTYVDPTT
YEDPYQALIEFTFDISPNDVFITQVIGGGEFGDVCLGGLSRNSPAAAKWGATVPRGGGGA
SEQYETVAIKTLKSGSSAKAKAEFLTEATIMGQFSHPNVIRLIGVVTSSEPVMIVAEYMA
NGSLDQFLRSADQRGEKVAWEKITEMLYGIASGMKYLTDMGYVHRDLAARNVLLDQELRC
KIADFGLSRGVRSEGTSIEPEYTTNGGKIPVRWTAPEAITHRKFTPSSDVWSFGVVIWEV
CSFGERPYWDWTNQKVISEVMIGYRLPPPMDCPMGLYRVAQWCWKMERHERPTFTQLLAT
FHKYILQPALIEQDLGELPRRVMSQATLGYGSVVPTPPSSAAPMPSLDDFLRQIGLNHVY
GKLVSNNIHSVSDLAATSHLDLLACGLISPECSIVRDGLNGRISSGTTPPSSSSGSNGTI
HATTRGTRTTRPREEGFFV
>CapteP198909 pep supercontig:Capitella_teleta_v1.0:CAPTEscaffold_366:151742:164045:-1 gene:CapteG198909
transcript:CapteT198909 gene_biotype:protein_coding transcript_biotype:protein_coding
MRREGGGGRGSGFNATLLDMARPVIKTPGQRSCDVCGLMEGALKRRYALRVYTVCRVEAQ
QVNNWLRTPYIPRGTANRVYVEVTFTMRKCTKYHDPERLQQCKESFKLLYYEAESDFANS
MRPTWDESTYTLVDAIAADQVFTDLSNPVLNTEVRDLPITRRGLYLAFFDQGACTTLIRV
RLYYHMCPSVTKNFAVFPNRTTGQERTALEKVEGQCVEHAAMDKAPTYLCNSAGAWMYAQ
GGCKCMPGYQPYEDTACVACPVSTYKWRTGDDACRSCPAFSEAPEEGAVECECLTGYYRS
ASDDKAMPCSTPPSAVSNLSVTFFDQSLVNLSWTQPDQLGGRADLHYRVECSGCGANVKY
QPSKTGFNSTRISISGLTPDTRYKLYVYAENGVSKQSGSTVNSFVECFPAVVTEVRVVSV
GVNYITLGWSPPLKEPGVVIVLYEVRYSVHELNENSSAVDVGNRLNLTLSGLTPQTQYHF
QLCRMAYRVTFLXTRTQSDLASLGKSSDSPVGIIAGSAVAVVVCMVIVIIMVVIFLNRNR
VKTYIDPHTYEDPNQAVREFTKEIDASCIRIESVIGGGEFGDVCKGSLRVPGKGELTVAI
KTLKAGASDKNRLDFLTEASIMGQFDDPNVIYLEGVVTKNNPIMIIIEYMENGSLDSFLR
TNDGKFTIIQLVGMLRGIASGMRYLSEMGYVHRDLAARNILVNESFVCKVADFGLSREME
CDDTDGAYTTKARSLSGGRPLKRSRTASSRPPRPYWNWSNQDVIKAVDRGFRLPPPMDCP
EAIYQLMLDCWQKERSHRPKFNAIVKTLDKLIRAPELNGASVVIFRPHQLLDPNLPDLTN
YSSLEEWLQSIKMDRYTEVFVHAGFQGMEQVAQLTLADLLELGITLVGHQKKIMNSVQTL
RAQLYGAQVSHGFMV

>EDO39123 pep supercontig:ASM20922v1:NEMVEscaffold_106:288739:297428:1 gene:NEMVEDRAFT_v1g209449 transcript:EDO39123 gene_biotype:protein_coding transcript_biotype:protein_coding description:Predicted protein [Source:UniProtKB/TrEMBL;Acc:A7SAX4]
MKVWVYLVVDLLVFLYSSHAEKVPLANFMNGKKWRERWSDSGSSSWDLSRLELGACDDTS
SSQPNGWVRSDVINTGPATRIELTATFTARDCSSFSGGSYCRHDFDVYAHQSETQYWGLA
PDPNNSPPGTYSKIGTLNATKLWTSFVNKTQNVQTFSLILENSKPYVYLALHYNGGCLAV
QSILVEYFRCADMTLPSTLVQLRATVAPANNSINVSGACAPNSKQVSGAGDLYGYCQSDG
EWSSGLFSGECQCDAGHQNTTTGGGGAECQACHVGTYKAIAGDLGCTGCPNNSAAMETGS
TGCTCVSGFYRKSGESNNNTCTGPPTAPTSLDTNFLNDSVVTLSWSLPNNTGGRSDVYYR
VECFTCNGNSIVCVKACGGTVTQHGTSARVSGLSAFTYYMFRVYARNGVSDEAERGGQEA
EFAKLVIQTHDAVPGKPEITAVEKVGLTHVRVIWKLLKPNGIITSYELKYHVTGKSANEK
AVTINATRATVDIDRDKGYQFRVRARTRLGYGPFSDPVGLQAGQTSSSSSSPLVIATSVL
GAVPVLVILLVAANLSVIKRLGSGNFGHVDKAMAYGIPGFPGQVTVAVKTLKASADEKDK
TDFLAELNLMKSLRPHPHVVRLIGCCTRQAGELSSTNIEFQVANHLAIILEYLPYGDLLG
YLRRSRGHEDWYCSGDLRPPSRLVSKDMVKFAWMIADGMAFLAANKDYINLDLYNDELYA
NFDAA
>EDO39124 pep supercontig:ASM20922v1:NEMVEscaffold_106:307378:322357:1 gene:NEMVEDRAFT_v1g209451 transcript:EDO39124 gene_biotype:protein_coding transcript_biotype:protein_coding description:Predicted protein [Source:UniProtKB/TrEMBL;Acc:A7SAX6]
MKVWVYLVVDLLVFLYSSHAERVLLANFMNGKPWRERWTSSASSSWDLSRSELGACDSSL
SQPNGWVRSDVINTGPATRIELTATFDARDCSSFSVGSYCRHDFDVYAHQSETQYSGLAP
DPNNSPPQTYSKMGTLNATKLWTSFGNKTQNVQTLSLILENSKPYVYLALHYKGGCLAVQ
SILVEYFRCMNMTLPSTLVQLGATVAPANNSINVSGACAPNSKQVSGAGDLYGYCQSDGE
WSSGLFSGECQCEAGHQNTTTGGGGAECQACKVGTYKAVHGYFVCASCPDNSVAMEIGST
SCTCVRGFYRKANESISSACTGPPSPPTSLYKPFLNSSAIILSWSAPNNTGGRTDDDGSV
CVEACGGTVAQQGTSARVSGLSAFTYYMFRVYARNGVSDEAEMGGQDAEFAKLVIRTRDA
VPGKPEITAVEKVDLTLVRVSWKLLKHNGIITSYELKYHVTGKSANEKSVTINATRATVD
IDRDKGYQFRVRAQTRLGYGPFSDAVSLQAGQTSSSSSSPLVIGTSVLGAVLVLVILLVS
AQEEYDDIGETNPGLDAIYSCVGEDEAWEVPPQNLSVIKRLGSGNFGHVDKAMAIGIPGF
PGQVTVAVKTLKYYLAIILEYLPYGDLLGYLRRSRGHEDWYCSGDLRPPSRLVSKDLVKF
AWMIADGMAFLAANKCVHRDLAARNVLVGEHNTCKISDLGLARDVSQDIYTRTSSMVLWD
SSLGDFYNCVVVPLSLAGQTGSSSSSPLVITTSVLGAVLVLAILLVAAQEEYEDMGETNP
GLDTIYSCVGEDEAWEIPPQNLSVIKRLGSGNFGHVDKAMAIGIPGFPGQVTVAVKTLKS
TAQKKDKTDFLTELNLMKSLRPHPHVVRLIGCCTRQTGELSSTISEFQVASNS
>EDO40993 pep supercontig:ASM20922v1:NEMVEscaffold_77:357690:366024:1 gene:NEMVEDRAFT_v1g207152 transcript:EDO40993 gene_biotype:protein_coding transcript_biotype:protein_coding description:Predicted protein [Source:UniProtKB/TrEMBL;Acc:A7S5K7]
MMFANAKNPSGELVPRFVAEPDQRILSFSHFSTTNISVIAFISGAVWRARFSGSAQNAVE
HTFCGAHADQPYKQERAYFGKISLVLVYNGTTSYSMADLGDFYCIWDIHSRTFRHSGRFD
IVLLFSITTLPWSINLRIMDFFILVTSRNLKGKSDNWSSAIIENRSTIVSDRQFTICACF
YDVPKCEIIFVFFNIINYAENWLDILLDGPRAGWSITGKSVPGQIANWLHSGSSNQYSVC
DVSSSKEPTNWLRTDYINAKDAKRVDIKVIYTIRKCKDLSKFCKETFNLYVYHASQSEPS
PDPATMPFTWLRKANVTHLPSGPATDADKNTMLVSLVPRKEGIFLAFYDQGACMSITKVV
VSFNFCPPRGGILVDFVRTVSPLSDSVQKKVEGTCVSENSVNTTALTAVCTSDGNWNLSG
DVKCYCKKGYAYDKVNNDCKGCPPGNFKSTLSNTGCRPCPENAQPNTLRTECVCRNEYFR
LLNHNMTVPCKPLPDGPRYANASALNSTAVRLTWTPVTPMSEGISYEIDCFVCTDEKDTK
CVVQCGKSPVSYDPGRSGLVQPNATIAGLSPETKYMFKVYTVTDLNKVADRKMWQFAQAF
VMTGKGSKKPPANQEKSADFQKMALTIGIPVIVFIIVLVVGCAVFIWK
>EFV53427 pep chromosome:Tspiralis1:GL622789:5943971:5948283:1 gene:EFV53427 transcript:EFV53427 gene_biotype:protein_coding transcript_biotype:protein_coding
MTQLCSTQQQQQLSSGVKDRREWSEESFANVKEGNNWRTYTVCDFYKDSANHWLRTPYIA
RGNARRLFIEMTFTMRACKEFPQTALTCKETFTLLYHEADHDVANESTSLPWNERSYRIV
DKITSNGARYAHSAAGEAETAAAMATPLLAEHASKTTVVRSVPVSKNGVYYQVCEEVLAN

FAWFDETPAGPELTSIQETVGQCVDNAKPTGESMPKFVCKADGSWDLLSGSCQCNAGFEA
FDSHCSACKMGHFKDVKGSVGCRPCPLHSHTVSNGSVFCQCEPGYYRAQVDALSEPCTQP
PSKPQNARVEFVDQNSVVIAWNRPVSNGGRSELWYRVDCRDCPAGIRYGSELMSSNRIHV
TNITISGLEPSTLYTVFVYAENDVSALLNGLPMYAVVEVTTTALAPVILSELRVESVAEN
EVSLAWHAPEVPLHLGTAYEPPTLQRNRHGKPKLHVQYYLVDYAARRGVATSRTGDHIRI
TTQQPHAFVQGLLADTEYGFKVRAYTSRGWSAWTDTKWIRTSASSQYNSVAGGLNPHGSS
STKYLYAAIGVLLFIISVLLTLAYRRRLPFHLCGYNKQPSDLDVLEYKAVNSAIVQLPRR
SGSAYKPYVDPLAYEDPKQALREFANEIDSTAVALDEIIGEGEFGEVYKGKMCVPGCGVE
VVAIKSLKTGLSVKAKLDFLSEASIMGQFDHPNVIRLRGVISRSEPAMIVTEYLENGSLD
NFLKRNGDRLDTVQLVRMLNDVASGMAYLSDKGYTHRDLAARNILVDGKICCKIADFGLS
RELEGSTELAYTTKGGKIPVRWTAPEAITFRKFTSSSDVWSFGIVVWEVLSLGDRPYWDW
TNQEVVDAVDQGYRLPAPHNCPATLYRLMLACWDKDYTCRPTFHQLRCMLSDFLHCPDLM
YSPTFLTGTSQQNQQFYTLSARVFAAGSFPYACSVEDFCRKLNLDRRFAQVFHAHGVHRP
VIVSELTSADLASMGLSRVDQEIVTRALVKFRLANANRMQQSNAGTVGRRSVRVYEKSPV
IASNTRVYTTPGLVEQTTMDNNDSLPRRSNGFFV
>EFX79285 pep supercontig:V1.0:DAPPUscaffold_30:149315:159973:1 gene:DAPPUDRAFT_104369 transcript:EFX79285
gene_biotype:protein_coding transcript_biotype:protein_coding
MAAARRSWVEESFTNFEKGINWRSYVVCDVAYNNVNNWLWTPFIERGEANRIYIEIKFSM
RDCSLFPGTALSCKETFSLLYYEFDAATREPPPWEPESYKLIDRIAADEGRFTSSNEVII
NTEVRSIPVTKKGVYFAFRDQGACISLLAIRVYYITCPEVTASFAAFPATPTGRELTSIE
QSNGVCVANAVEDAPPKYLCKGDGNWYLQGGGCLCKPGYEANADKQTCNVCPPGKYKHRS
GDDKCQTCPDHSMAPYSGSSECRCNQNYYRALKDPKSMPCTQSPSKPQNLTVNFVDQSTV
VLSWNQPHNLGGRSDTVYRVVCDACNVGVTYNPPQGTFNDTKVTISGLNPVTTYRFQVFA
DNGVSDQVPPNEHQYIDIAVTTEASVPSSVSNVRIINVRPTELTLRWDAPDDPYSDIEMY
EVRYFQKGFENNASSMLINRPESAFSSLRQQTVYGFQVRAKTTHGWGEFSSPAFKTTGAV
LATYEHTEENLQVRIIAGAVVAGIIIVVVIIVVTVLYLRRSNDECNKKQPSDCDTLEYRN
GEANAPRSYVDPHTYEDPNQAVREFAREIDAAYITIEAIIGGGEFGDVCRGNLKLPQRPE
MLVAIKTLKLGSSDKARVDFLTEASIMGQFEHSNVIYLQGVVTKSNPVMIITEYMENGSL
DTFLRANDGKFQVLQLLSMLRGIANGMQYLSEINYVHRDLAARNVLVNSQLVCKIADFGL
SREIESTTEGAYTTRGGKIPVRWTAPEAIAFRKFTSASDVWSFGIVCWEVMSYGERPYWN
WSNQDVIKSIEKGYRLPAPMDCPEAIHNLMLDCWQKERAHRPAFTAIVATLDKLIRCPEL
LQRLAQSRHTEPLCPEAPDMLQFKSIEEWLASLKLSRYVDNFQQAGVTTLDAVTRLSHAD
LSGLGIILLGHQRKIMNSIQAMRAQLSISMSEGFLV
>EKC33758 pep supercontig:oyster_v9:scaffold695:472045:519122:-1 gene:CGI_10023292 transcript:EKC33758
gene_biotype:protein_coding transcript_biotype:protein_coding description:Ephrin type-A receptor 4
[Source:UniProtKB/TrEMBL;Acc:K1QIZ1]
MARRRTVFSVAPYFIVLICLSSVGLCTREDSSRFSNLKNWTAHINGQSDSNTGPFVPLEE
NLLRCIIGRMWEDPQARACGSKGVTKDEYFVMAGLFANPSPVILGEIRHLIHPFTLPNEW
PLKMFELSNWIEGSYRDNGNIIRIFSSCSVSSPDVDNWLRTPYIPRRGANSIRIEIRFSM
RRCVQHSDPSTIKQCKETFNLYYYEADADIANAHMPTWDSVSYKHIDKIAADNLYDSNTD
IKLNTEVRDVSLSPGMGGLYIAFQDLGACVTLISIKISYIMCPNVTKNYAFFQETPVGRE
NPGFEEHYGVCVPNAAEKTRPKYLCQSDGVWIYGSGGCECLSGYEGQGENACVECRPGFY
KWLLGNTPCYPCPANSYTNAPKSVECNCADGYFRNPRDSKNSSCTQPPSKPRNLVAQEIT
HNSITLAWIPPVSDGGRKDVQYRITCSPCETVSFNPGWQNLNQTRVTITGLTSSTTYSVR
VSAVNGVSNQSNIPIEFDEGQFVTKASTQTIQHFQISSRNQAEVTVTWDIVPGIRNQVLF
YQVKSAPRLGGYGDIFLKNTSSTHQNYTFKNLNLGTEYSLQVRCYLVDKGWGDFTKPLHV
IPGDTAGEKKMDPPPEMSVGIIIAAVLAVILCMAIATGMIVILIKRNQGNCQNDKGGGEY
DPPYNPVTMPLFPPNDCVKSYVDPHTYEDPNQAVREFTREIDASHITIESVIGGGEFGDV
CKGKLRNPVKPEMSVAIKTLKPGATDKNRLDFLTEASIMGQFDDQNVIFLEGVVTKSHPI
MIVTEYMENGSLDTYLRNNDGKFTIIQLVGMMRGIASGMRYLSNMGYVHRDLAARNILIN
DNLVCKVADFGLSREIDIDTTDGAYTTKGGKIPVRWTAPEAIAFRKFTSASDVWSYGVVM
WEVVSYGERPYWNWSNQDVIRAVDRGYRLPPPMDCPEALHQLMLDCWQKERGNRPKFDII
VKSLDKFIRAPELLRKIAKPRPEFLMDTNRTLPMNRQGEVSNFTNVEGWLISIKMEKYMD

NFLNCGYRTIEQVLKITQQDLEQRVGVTLIGHQKKIMNSIQTLRAQRFGQFPQVSEGFLV
>EMLSAP00000000364 pep supercontig:LSalAtl2s:LSalAtl2s1053:94623:99252:1 gene:EMLSAG00000000364 transcript:EMLSAT00000000364 gene_biotype:protein_coding transcript_biotype:protein_coding
MCVSHFGWRLLLGILSFSQWAHGEIHVLLDSTKEAQLDWNRFPSSGTPGWVEESYTDFDK
SINWRCYVACDVGYPNVNNWLRTPLVRXRGVARLYIEIKFSVRNCRLFPGEALQCKETFR
LYASSSSSSSGGDSWDQADYTMLDRIAADEGRITSNDEMIINTEVRSLDVSNMKRGLYFL
FQDEGACLTLLAVKVYYLTCPRVVLNLAQYPETPTGKGVASIERXQGKCVPHAVPKGDLF
NLCQADGSWAYSSGTCLCVEGYEAHNLTGLCAPCPIGKYKAGKGNVQTCTDCPARSKAIK
MGSGLCECDPGYYRGAEEPLSVPCRRPPGSPRDLKFKFLGPRTAVLNWNAPLEERNKDTK
YRAECYQGCADNSVHFDRKEIPFYETNVKVSDMDFGKTYIFRIYAVNAVSNVSGRLDNYS
EISIRTDDIDPLSKDADHTPNEEYISNNMNINVDQVHSHKVVLSWNWPKKDEKEDFLLRY
YEVKYFPRHSEHNNSQTIVTRNKKAEIDRLKEASTYGFRVRVKSGDGNHHHWSRFSSPPV
YQTTLDSFETPIFVGEHHQSGSSGVVVDRSSVIIGSIXAVIVTLLLVALLFFFVRKGGSS
GWIKWLSSIKMTRYSENFASAGIKDMTEVAKITLAQLQQDLGITLVGHQKKIMECWQRYK
QLPFQNYLCFVVKGP
>ENN72307 pep supercontig:DendPond_male_1.0:Seq_1102970:300449:308694:-1 gene:YQE_11050 transcript:ENN72307 gene_biotype:protein_coding transcript_biotype:protein_coding
MLTLFPGNALSCKETFSLLYYEFDAATREPPPWDADRYKLIGRVAAGEGRFNSNSEVNIN
TEVKSIPVTKKGVYFAFRDQGACISLLAIKIYYITCPEVTINFARFPATPTGKEITIIEQ
AQGICVNHAEVVGSSPIYLCKGDGKWTLPSGGCKCKAGFQPDMDKQTCNVCQPGTYKSEV
GDYLCFLCPKYSHGPDYGLTECKCNQGYFRAPTDPKNMMCTQPPSAPVNLTVNFVDQSNV
ILSWLPPENLGGRSDIAYRIKCDACSVGLVQYNPPMEYFNETRMHISGLNAVTTYRFQIF
SENGVSHLAPRPNTDYADVMITTEASVASSITNIRVTSVKSTEITLAWDAPITDGDLESD
QVETYEVR
>ENN72308 pep supercontig:DendPond_male_1.0:Seq_1102970:312967:314016:-1 gene:YQE_11051 transcript:ENN72308 gene_biotype:protein_coding transcript_biotype:protein_coding
MDNIVEKLWVEESFTNFQKGINWRSYVVCDVAYSNANNWLWTPFIDRKEANRIYIEIKFT
IRDCSLFPGRLITLQPRGTVLRFIDVILQIQLPILWFC
>FBpp0126032 pep supercontig:dana_caf1:scaffold_13099:2646460:2721368:1 gene:FBgn0099834 transcript:FBtr0127540 gene_biotype:protein_coding transcript_biotype:protein_coding gene_symbol:Dana\GF22840
MESTLCTSQGGGQKICSTFYPKRDLCIEKDLLRKIQFAPTSEKPLVAQKEHQFASRTSRL
LIIWFIVLTIHFRYARADQVVLLDTTREATLEWTRYPYGPQAQTPGWVEESFTDFVKGIN
WRSYVVCDVAYHNVNNWLWSPFIDRGPANRLYIEIQFTIRDCSLFPGNALSCKETFSLLF
YEFDAATREPPPWQTDSYKLIARIAAGEGRFNQNSDVDINTEVKSIAVNKKGVYFAFRDQ
GACISVLAVKVYYITCPAVTENFAHFNETPTGREITIIEKQNGTCVKNAEPYEHPTYLCK
GDGKWTILTGGCRCRGGYEPNLLNNTCTECAIGTFKSADVMKCTPCPPNSNTSKPGSAFC
KCLPGYFRNPRDQKHMPCYSPPPAPKNLTLLFIDQTSAIISWTAPKKNEFQLTDDQTKKY
HSDIVYKLLCKMCNSNVMYNPASDTFTETKVTLSNLEPSTIYTVQIHSTHGVSHFISNSS
DFVLNDHSFTNASSVNTLLDLNEIKTEYEEIVFTTESVQLTTSFNLRILSITNNNANLAW
DKPVHSDFPLEFYEIRWFPKHDLDANNKSSLSTKETKAQIEDLQENTEYGFQVRCKTLNG
YGSYSSIIYAKTSHPINSGYDDSDQVRFIAGAIVTGVLFLVIFIVATVYFMRSKHQDEID
KKSGNHLPLPLDYASNEVNTMDTTPIVKTLHLNVTTPLFGHNRSYVDPHTYEDPNQAIKE
FAREIDANYITIEAIIGGGEFGDVCRGRLKIPPNFVQDIDVAIKTLKPGSSEKARCDFLT
EASIMGQFDHPNVIYLQGVVTRSNPVMIITEYMENGSLDTFLRVNDGKFQTLQLIIMLRG
ISSGMAYLSDMNYVHRDLAARNVLVNSLLICKIADFGLSREIENASDAYTTRGGKIPVRW
TAPEAIAFRKFTSASDVWSYGVVLWEVMSYGERPYWNWSNQDVIKSIEKGYRLPAPMDCP
EALYQLMLDCWQKQRTHRPTFASIVSTLDNLARQPQSLLTTRNSPESDNTHILDAQRGQN
VFISTDLWLEHIKMSRYCQHFKDANLINAQQISRLTAQQLSDMGITLVGHQKKILHQARQ
LDTII
>FBpp0163312 pep supercontig:dmoj_caf1:scaffold_6498:1152138:1160901:1 gene:FBgn0136849 transcript:FBtr0164820 gene_biotype:protein_coding transcript_biotype:protein_coding gene_symbol:Dmoj\GI14095
MLCCALICGKLHTHIHTLFIHTYMLIATGAYIHIYNIHIYEIYKMSAIHWTFRIFQTQNS
NQNRNSQATEISIRTKNNKLTSLIPFSNFSIVLLIWIISVIYNLQIANADQVVLLDTTRE

ATLEWTRYPYGPQAQTPGWVEESFTDFVKGINWRSYVVCDVAYHNVNNWLWSPFIDRGPA
NRLYIEIQFTIRDCSLFPGNALSCKETFSLLFYEFDAATREPPPWQTDSYKLIARIAAGE
GRFNQNSDVDINTEVKSIAVSKKGVYFAFRDQGACISVLAVKVYYITCPAVTENFAHFNE
TPTGREITIIEKQNGTCVENAEPYEAPTYLCKGDGKWTILTGGCRCKVGYEPNEVNNTCT
ECPAGTFRSAEVRKCISCPANSNSSKTGSPMCNCLAGYYRHPADGRHMPCYKPPNAPTNL
TLLFVDQTSAIISWSAPTRDDNQLVTANGLEKYRTEVVYKVRCSSCSSNVMFNPSTDTFN
ETKITLTNLEPATTYTVQVHAANSISYVADIGLYYNLTYSSSDNALSNTSSFSNINGHQL
DLSEVETKYAEIVFTTESAMLSTVFNLRVVAITNKEASLVWDKPGQTDAPIELYEVRWFP
KSEMETINNTTLSTKETKALIYNLLENTEYGFQVRCKTVNGFGAYSNMVYAQTHQSVGTV
YEDSMQIRFIAGAIVTGVFFLVIFIVATVYFMRSKHQDELDKKSTNHLPLPMEYASNEVN
AMDTTPIVKTLHSNVTTPLFGNSRSYVDPHTYEDPNQAIREFAREIDANYITIEAIIGGG
EFGDVCRGRLKIPPNFVQDIDVAIKTLKPGSSEKARCDFLTEASIMGQFDHPNVIYLQGV
VTRSNPVMIITEYMENGSLDTFLRVNDGKFQTLQLIVMLRGIASGMSYLSDMNYVHRDLA
ARNVLVNAQLICKIADFGLSREIENASDAYTTRGGKIPVRWTAPEAIAFRKFTSASDVWS
YGVVLWEVMSYGERPYWNWSNQDVIKSIEKGYRLPAPMDCPEALYQLMLDCWQKQRTHRP
TFASIVSTLDNLARQPQSLLTTRNSPENDSSTHILDAQRGHNIFISTDLWLENIKMSRYS
QHFREANLVSAQQISRLTAQLLSDMGITLVGHQKKILHQARQLDTII
>FBpp0182284 pep supercontig:dper_caf1:scaffold_42:439755:447960:1 gene:FBgn0155780 transcript:FBtr0183792
gene_biotype:protein_coding transcript_biotype:protein_coding gene_symbol:Dper\GL18177
MPSTYFSVHKRERKNDCTGTRNTISSMSKLASLQIQFHKINTLVPSTLVPIPKPMHNGSR
HIARLSLLCISWLIAIQCNLRFTYADQVVLLDTTREATLEWTRYPYGPQAQTPGWVEESF
TDFVKDINWRSYVVCDVAYHNVNNWLWSPFIDRGPANRLYIEIQFTIRDCSLFPGNALSC
KETFSLLFYEFDAATREPPPWQTDSYRLIARIAAGEGRFNQNSDVDINTEVKSIAVNKKG
VYFAFRDQGACISVLAVKVYYITCPAVTENFAHFNETPTGKEITIIEKQNGTCVENAEPY
EPPTYLCKGDGKWTILTGGCRCKVGYEPNDVNKTCTECPVGTFKSPEVRKCTPCPPNSNS
SKTGSPFCKCLPGYYRHPRDGRHMPCYSPPAAPTNLTLLFVDQTSAIISWSAPARNESQL
VEGQLHSYRSDIVYKIRCSMCNANVMFNPSTDTFNETKITLTNLDPVTTYTVQIHATNGI
SYLIDSSLHSNETTANNEISFANASTASNPLDLNEIKTEHAEIIFTTESALLSTVFNLRI
LAITSKDADLEWDKPLQNDAPLEFYEVRWFPKLELDSINKTALNTKDTKAHIEGLMESTD
YGFQVRCKTINGFGSYSNMIYAQTLQSVSSVYDDSVQIRFIAGAIVTGVLFLVIFIVATV
YFMRSKHQDELDKKSTNHLPLPMDYASNEVNSMDTTPIVKTLHSNVTTPLFGNSRSYVDP
HTYEDPNQAIREFAREIDANYITIEAIIGGGEFGDVCRGRLKIPPNFVQDIDVAIKTLKP
GSSEKARCDFLTEASIMGQFDHPNVIYLQGVVTRSNPVMIITEYMENGSLDTFLRVNDGK
FQTLQLIVMLRGIASGMSYLSDMNYVHRDLAARNVLVNAQLICKIADFGLSREIENASDA
YTTRGGKIPVRWTAPEAIAFRKFTSASDVWSYGVVLWEVMSYGERPYWNWSNQDVIKSIE
KGYRLPAPMDCPEALYQLMLDCWQKQRTHRPTFASIVSTLDNLARQPQSLLTTRNSPETD
GAHILDTQRGQNIFISTDLWLENIKMSRYCQHFKEANLVNAQQISRLTAQQLSDMGITLV
GHQKKILHQARQLDTII
>FBpp0228658 pep supercontig:dvir_caf1:scaffold_13052:1246727:1257231:1 gene:FBgn0201456
transcript:FBtr0230166 gene_biotype:protein_coding transcript_biotype:protein_coding gene_symbol:Dvir\GJ14241
MQSKHYSFRLHDITKNCHDTDLSKNKAACTQFSVQIKQSQMPNPMPLQCNISESRQLSKL
SIFQLIWIICIAYQLNIANADQVVLLDTTREATLEWTRYPYGPQAQTPGWVEESFTDFVK
GINWRSYVVCDVAYHNVNNWLWSPFIDRGPANRLYIEIQFTIRDCSLFPGNALSCKETFS
LLFYEFDAATREPPPWQTDSYKLIARIAAGEGRFNQNSDVDINTEVKSIAVSKKGVYFAF
RDQGACISVLAVKVYYITCPAVTENFAHFNETPTGREITIIEKQNGTCVENAEPYEAPTY
LCKGDGKWTILTGGCRCKAGYEPNDVNNTCTECSAGTFRSAEVRKCVSCPPNSNSSKTGS
PMCKCMPGFYRHPADGRHMPCYRPPGAPMNLTLLFVDQTSAIISWTAPAKEDALLINGVE
KYRTDVVYKIRCSMCNANVMFNPSTDTFNETKITLTNLDPVTTYTVQIHATNGVSHLVEN
GAFANETYLNSETPFGINSSFSNSNTYQLDLSEIKTNYAEIVFTTESVMLSTVFNLRVVA
ITNKDADLIWDKPGQSDAPVELYEVRWFPKSELEAINKTTLSTKETKARIDNLMENTEYG
FQVRCKTINGFGSYSNMVYAQTHQSVGSVYEDSMQIRFIAGAIVTGVFFLVIFIVATVYF
MRSKHQDELDKKSTNHLPLPMDYASNEVNAMDTTPIVKTLHSNVTTPLFGSSRSYVDPHT
YEDPNQAIREFAREIDANYITIEAIIGGGEFGDVCRGRLKIPPNFVQDIDVAIKTLKPGS

SEKARCDFLTEASIMGQFDHPNVIYLQGVVTRSNPVMIITEYMENGSLDTFLRVNDGKFQ
TLQLIVMLRGIASGMSYLSDMNYVHRDLAARNVLVNAQLICKIADFGLSREIENASDAYT
TRGGKIPVRWTAPEAIAFRKFTSASDVWSYGVVLWEVMSYGERPYWNWSNQDVIKSIEKG
YRLPAPMDCPEALYQLMLDCWQKQRTHRPSFASIVSTLDNLARQPQSLLTTRNSPENDSS
HILDTQRGHNIFISTDLWLENIKMSRYSQHFREANLVSAQQISRLTAQLLSDMGITLVGH
QKKILHQARQLDTII
>FBpp0325417 pep chromosome:ASM75419v3:4:440297:450980:1 gene:FBgn0195747 transcript:FBtr0361710
gene_biotype:protein_coding transcript_biotype:protein_coding gene_symbol:Dsim\GD24409
MLKSKLASLCKNTYTLSFCVNMYNMYGHMYSWILNWTRNTNNAIDIGKSASKGVQANYKI
KLLENDPNKMKMVGCHPNDKDEVKYDGIKSKTSSVTILTSVQSDAIRTLKSAFYKEQAPI
SEQNRSQICSRMSLLSTILLIIISIHFKLARADQVVLLDTTREATLEWTRYPYGPQAQTP
GWVEESFTDFVKGINWRSYVVCDVAYHNVNNWLWSPFIDRGSANRLYIEIQFTIRDCSLF
PGNALSCKETFSLLFYEFDAATREPPPWQTDSYRLIARIAAGEGRFNQNSDVDINTEVKS
IAVNKKGVYFAFRDQGACISVLAVKVYYITCPAVTENFAHFNETPTGREITIIEKQNGTC
VENAEPYETPTYLCKGDGKWTILTGGCRCKAGYEPNYINKTCTECPLGTFKSPEVTKCTP
CPPNSNASKTGSPFCKCVSGYYRHPNDGRHMPCYSPPPAPTNLTLLFVDQTSAIISWSAP
AKNESLSSETQSKIYHSDIVYKIKCNMCSPNVVYNPSTDTFNETKITLTNLEPVTTYTVQ
IHAINSVSHINEFNRHSNESSLVAVNDIIFSNTSLLNIPLDLNEVKTGQAEIVFTTESVL
LSTVFNLRILAITNKDADLEWDKPVQSDFPLEFYEVRWFPKVELDAINKSALNTKETKAH
IVGLLENTEYGFQVRCKTINGFGSYSNMIYAQTLQSVGSVYDDSVQIRFIAGAIVTGVLF
LVIFIIATVYFMRSKHQDELDKKSTNHLPLPLDYASNEVNSMDTTPIVKTLHLNVTTPLF
GNSRSYVDPHTYEDPNQAIREFAREIDANYITIEAIIGGGEFGDVCRGRLKIPPNFVQDI
DVAIKTLKPGSSEKARCDFLTEASIMGQFDHPNVIYLQGVVTRSNPVMIITEYMENGSLD
TFLRVNDGKFQTLQLIVMLRGIASGMSYLSDMNYVHRDLAARNVLVNAQLICKIADFGLS
REIENASDAYTTRGGKIPVRWTAPEAIAFRKFTSASDVWSYGVVLWEVMSYGERPYWNWS
NQDVIKSIEKGYRLPAPMDCPEALYQLMLDCWQKQRTHRPTFASIVSTLDNLARQPQSLL
TTRPSPESDGNHILDGQRGQNIFISTDLWLEHIKMSRYCQHFKEANLINAQQISRLTAQQ
LSDMGITLVGHQKKILHQARQLDTII
>FBpp0414687 pep supercontig:dgri_caf1:scaffold_14822:805564:816806:-1 gene:FBgn0131387 transcript:FBtr0464396
gene_biotype:protein_coding transcript_biotype:protein_coding gene_symbol:Dgri\GH23930
MQSTLWRFGFLKRKKSRHDDDATCSTKNKLASTQLPVPIVKYQMPMQCYTLRCVDTTQLS
KLKIIQLILLIAIAYILRTVNADQVVLLDTTREATLEWTRYPYGPQAQTPGWVEESFTDF
VKGINWRSYVVCDVAYHNVNNWLWSPFIDRGPANRLYIEIQFTIRDCSLFPGNALSCKET
FSLLFYEFDAATREPPPWQTDSYKLIARIAAGEGRFNQNSDVDINTEVKSIAVNKKGVYF
AFRDQGACISVLAVKVYYITCPAVTENFAHFNETPTGREITIIEKQNGTCVENAEPYEPP
TYLCKGDGKWTILTGGCRCRVGFEANDANNTCKECPVGTFRSSEVRKCVPCPPNSNSSKT
GSPLCKCVPGFYRHPADGRHMPCYKPPGAPMNLTLLFVDQTSAIISWSAPVKDDLYLANT
VEKYRTDIVYKVRCIMCSSNVMLNPSTDTFNETKITLTNLDPVTTYTVQIHATNGISSLI
EPYANETYTNSESPFVNASSFLSGNSQQVDLSEIKTKHAEIVFTTESVMLSTVFNLRVVA
ITNNDADLVWDKPVQSETPIELYEVRWFPKSELDTINKTALNTKETKIHLTNLLENTEYG
FQVRCKTINGFGTYSNMVYAQTHQSVGSVYDDSIQIRFIAGAIVTGVFFLVIFIVATVYF
MRSKHQDELDKKSTNHLPLPMDYASNEVNAMDTTPIVKTLHSNVTTPLFGNSRSYVDPHT
YEDPNQAIREFAREIDANYITIEAIIGGGEFGDVCRGRLKIPPNFVQDIDVAIKTLKPGS
SEKARCDFLTEASIMGQFDHPNVIYLQGVVTRSNPVMIITEYMENGSLDTFLRVNDGKFQ
TLQLIVMLRGIASGMSYLSDMNYVHRDLAARNVLVNAQLICKIADFGLSREIENASDAYT
TRGGKIPVRWTAPEAIAFRKFTSASDVWSYGVVLWEVMSYGERPYWNWSNQDVIKSIEKG
YRLPAPMDCPEALYQLMLDCWQKQRTHRPTFSSIVSTLDNLARQPQSLLTTRNSPENDST
HILDSQRGHNIFISTDLWLENIKMSRYSQHFREANLVSAQQISRLNSSTAIRYGYNFSRA
SEEDFTPSSAAGYNNIDSVFFKMYIHTFNIYINIYI
>GSADVT00000371001 pep supercontig:AMS_PRJEB1171_v1:HG380759:12711:18022:1 gene:GSADVG00000371001
transcript:GSADVT00000371001 gene_biotype:protein_coding transcript_biotype:protein_coding
MFMKLVFIFNCFIYSIIQATKEVVLLDTREARDYLNWDKTSTNGNLNDGWGELSFEAKLF
PTLLLSSSTSTLWRTQYVCDITSERHVDNWLRSPYFKRQQAQRIEIELGFSIRDCSTFQT

PNEIRSCRETFELYIHESDYEDNDFLWSSYQLVDIIAGNPSSSSSGNNLTVNVKTRGISI
NKNGFYIAFRDQGACVSLLYVKIFYRLCEDTTVGLVHFPETPAGAHLTDIVERHGICTTN
SKMLNKPLGFCKGNGEWAFQETSLRDSCHCQDGYELLIDNNNAMNNGLLSRAICKACSIG
TFKSTISNNQRCEPCPLNSHTKDRGASSCICNDGFFRLNTSLLKSPCIGSPTEPQNVTVD
DIDQASVKISWKQPIEYSNDIVYRIECNHLIEGRSIPCESYISYYPNQTFINNTSVQILG
LDADTNYNVEVYAEQLSTHLLSKSVDLSFTTKRPIPKLIRDINVRRISLNTILITWSSND
FDLFQIRYWPLINEHHKSLVTLLHNNFTLITASDNYKFQLRGRTRFGWSLYTHEKVLSLS
SILIDEQFLTNAKLTNMSTKMSENKNILLIGPLIILALLVTVIILAIIYSKKKRSCRLKT
ASDCESLDYQKRQGHGIIVITGGYRPMMLRYTNNPANKSVTPWSAPLWPPIPTTAKTYID
PHTYEDPTKAVKDFSRELDPNLIVIESVIGGGEFGDVCRGRLRKPNLMKDVPVAIKTLKQ
GATEKTRLDFLSEASIMGQFDDENVIYLEGVVTKHHPIMIVTEYMENGSLDAYLRANEGN
RLLDALQLTRMLRGIASGMTYLSEMKYVHRDLAARNILVNKDLVCKVADFGLSREIDEGD
SYTTKGGKIPIRWTAIEAIDYRKFTSASDVWSFGVLSWEVLSFGERPFWNWSNQDVIKAI
KNYYRLPPPMGCPDVLYRLMLTCWHEDYLQRPKFAEIVQQLTQFIQIPSRLKYKIMQSKI
FSWRKKSEHILLY
>GSADVT00007809001 pep supercontig:AMS_PRJEB1171_v1:HG380783:499258:503439:-1 gene:GSADVG00007809001
transcript:GSADVT00007809001 gene_biotype:protein_coding transcript_biotype:protein_coding
MFPTLLLPSSTSTLWRTQYVCDITSERRVDNWLRSPYFKRQQAQRIEIELGFSIRDCSTF
QSPNEIRSCRETFELYIHENVYESENPLWSSYKLVDIIAGNRFTSNNYGSQSLGNKNNLT
VNVKTRGISVTKNGFYIAFRDQGACISLLYVKIFYRLCEEKSIGLIHFPETPTGAHLTDI
VERDGICSINSKMIRKPLGFCKGNGEWTFQETSLLDSCHCQQGYELLIDNNSKNNICPNG
TFKSTISNDQHCEQCPLNSHTKDKGSTYCFCNQGFFRLNASMFNSSCIGGPNEPENVTID
DIDQVSVHISWKQLNDNVYHIECTHSISCESYIIYQPNRTFINGSKVKIIGLDDDTRYKI
KIYAEQVSTHLYSQSIDLAFTTKPSISKQIRNVQIRRITFDQVVITWSPDDFDQYHIRYW
PLIDENNKIFVRLPMNNFTLTTTSDIYKFQIRAHTKLGWTSYTEEMVISLRSIFIDQSFS
SDVTRKLVENKNILLISPLIILGLIITVIILAIIIRFYNRRRICRLKTASDCESLDYQKR
QDSYFYYSVEYVSGSAPLWPSISTTTKTYVDPHTYEDPTKAVNDFAHELDPNSIVIESVI
GGGEFGDVCRGKLRKVNLMKDIPVAIKTLKAGATEKTRLDFLSEASIMGQFNNENVIFLE
GVVTKHHPIMIITEYMENGSLDAYLRTNEGNKCLDTLQLTRMLRGIASGMTYLSEMKYVH
RDLAARNILVNKDLICKVADFGLSREIDEGESYTTKGGKIPIRWTAIEAIDYRKFTSASD
VWSFGVLCWEVVSFGERPYWNWSNQDVIKTIKNAYRLPPPMGCSDALYQLMLACWNDNYL
QRPKFCEIVQQLTQFIQTSNILVPLAKQKFVFVNHPDEPNLAQLTSIADWLHNLQLDRLL
YLFRNHNYTNLSQICHFTVLDLKEILGNQMTIDEQNRLTDSLTHVRSQLVLISSNSLLTS
EGYLV
>GSADVT00013698001 pep supercontig:AMS_PRJEB1171_v1:HG380811:450832:456013:1 gene:GSADVG00013698001
transcript:GSADVT00013698001 gene_biotype:protein_coding transcript_biotype:protein_coding
MFMKLVFIFNCFIYSIIQATKEVVLLDTREARDYLNWDKTSTNGNLNDGWGELSFEAKLF
PTLLLSSSTSTLWRTQYVCDITSERHVDNWLRSPYFKRQQAQRIEIELGFSVRDCSTFQT
PNEIRSCRETFELYIHESDYEDNDFLWSSYQLVDIIAGNPSSSSSSGNNLTVNVKTRGIS
INKNGFYIAFRDQGACVSLLYVKIFYRLCEDTTVGLVHFPETPAGAHLTDIVERHGICTT
NSKMLNKPLGFCKGNGEWAFQETSLPCQLVHTPSVRNRQKKTCSIGTFKSTISNNQRCEP
CPLNSHTKDRGASSCICNDGFFRLNTSLLKSPCIGSPTEPQNVTVDDIDQASVKISWKQP
IEYSNDIVYRIECNHLIEGRSIPCESYISYYPNQTFINNTSVQILGLDADTNYNVEVYAE
QLSTHLLSKSVDLSFTTKRPIPKLIRDINVRRISLNTILITWSSNDFDLFQIRYWPLINE
HHKSLVTLLHNNFTLITASDNYKFQLRGRTRFGWSLYTHEKVLSLSSILIDEQFLTNAKL
TNMSTKMSENKNILLIGPLIILALLVTVIILAIIYSKKKRSCRLKTASDCESLDYQKRQV
SGHYGPDVFFNSGWSAPLWPPIPTTAKTYIDPHTYEDPTKAVKDFSRELDPNLIVIESVI
GGGEFGDVCRGRLRKPNLMKDVPVAIKTLKQGATEKTRLDFLSEASIMGQFDDENVIYLE
GVVTKHHPIMIVTEYMENGSLDAYLRANEGNRLLDALQLTRMLRGIASGMTYLSEMKYVH
RDLAARNILVNKDLVCKVADFGLSREIDEGDSYTTKGGKIPIRWTAIEAIDYRKFTSASD
VWSFGVLSWEVLSFGERPFWNWSNQDVIKAIKNYYRLPPPMGCPDVLYRLMLTCWNEDYL
QRPKFAEIVQQLTQFIQIPSRLVPLAKQRFFVDHPDHPDFTQVMSINNSVTIV

>HMEL008812-PA pep scaffold:Hmel1:HE670833:157588:160652:1 gene:HMEL008812 transcript:HMEL008812-RA gene_biotype:protein_coding transcript_biotype:protein_coding
MMWICAAVAAAVIAALHVSGEQVMLLDTTTEDTLGWTRFPYGLQASTPGWLEESYTNFEK
QINWRSYVVCDVAHHNVNNWLWTPFIERRNANRIYIEIKFTIRDCSLFPGNALSCKETFS
LLYYEFDVATREQPPWEPESYKLVGRIAAGDGRFNTNSEVDINTEVKSIAVTKRGVYIAF
RDQGACISILAVKVYYITCPEVTINFAKFPATPTGREVTIIEQANGTCVQNAETASGEAP
PVYLCKGDGKWTLPSGSCKCRAGYEPDHTNQICNECSPGKFKAHTGDDPCTPCPDYSEST
GFASIECKCVPGFFRAKKDPKNIPCTQPPSAPENLTVTFADQFSISLAWQPPHKAGGRTD
VNYKIHCSNCGPNVEYRPAASGLNSSRVTVMGLDPVTEYKFQVFALNGVTDLTGEPPKKI
EVTAVTEASVVSVITKLRVVSVESNKLSLAWNPPPIDLTDPDDAIESYEIKCYPKDNLEK
NANATVKITKEPHVIISGLKGDTEYAIRVRAKMKRGWGELSAIVYARTGSVLETTFVGEE
EGAQVKLVAGVMVTLVVISVVAIIATVVILRLRSDDECDKKQPSDCNALNYRNGEVYTGP
DRPAKTSNLIQFSSVEEWLECIKMSRYIEKFRAAGITDMNAVVDLTVHQLASLGVTLVGH
QKKIMNSVQSMRAQIRVSGPYGFLV
>ISCW022934-PA pep scaffold:IscaW1:DS950795:109073:153283:-1 gene:ISCW022934 transcript:ISCW022934-RA gene_biotype:protein_coding transcript_biotype:protein_coding description:Eph receptor tyrosine kinase, putative [Source:UniProtKB/TrEMBL;Acc:B7QJ81]
MDLREGNVPSVCHEDGIEGATWVEESFTNFEQGINWRSYVVCDVAYENVNNWLWTPFIER
GPAHRIYIELKFSMRDCNLFPGMALSCKETFSLLYYEFDAATREPPPWEPESYRPVDRIA
ADEGRFTSNAEVIINTEVRSVPVTKKGVYFAFRDQGACLSLIAVKVYYMVCPNVTTSLAH
FPETPTASEVTAIVATEGQCVANARVSQAPPRMLCKGDGNWTLPSGGCACLPGFRAVGSS
CQKPPRAPQNLSASCVDQSSVTLSWQAPSHQGGREDTTYRVVCDMCSPSVTFSPARQGFN
ETSVTVTGLRPVTAYRFQVYAENGVSGFDKGHYAEITVKTDALVQSVISNVRAKAIKSTE
VLLAWDAPQDPFPEVERYEVRFFPRKWAKNETVLQSHKLQLAVTGLRSRSEYGFQVRVKT
TRGFGEYSDTVYLTTGQYTHSPDEDKVQVRIIAGATVAVVILVIIVIVMIILYIKRSSDD
CNKKQPSDCDTLEYRNGEVTTPLFTHCTGAASRAYVDPHTYEDPNQAVREFTKEIDALHI
TIEAIIGGGEFGDVCRGKLKCPGRPEVTVAIKTLKPGSSDKARMDFLTEASIMGQFDHPN
VIFLQGVVTKSNPIMIITEYMENGSLDTFLRANDGKFQVIQLVGMLRGIAAGMQYLAEMN
YVHRDLAARNVLVNANLVCKIADFGLSREIESATEGAYTTRGGKIPVRWTAPEAIAFRKF
TSASDVWSFGIVCWEVMSYGERPYWNWSNQDVIKSIEKGYRLPAPMDCPEAVHQLMLDCW
QKDRSHRPQFTNIVKTLDKLIRCPESLRKIAQNRHQNPLDPNAPDMTQFKTVEEWLCSIK
MTRYLESFQRSGFSSMEAVSRLTLKDLTALGVVLVGHQKKIMNSVQTLRAQMNATMSDGF
LV
>KFM62390 pep supercontig:Stegodyphus_mimosarum_v1:KK114425:65238:98555:1 gene:X975_26035 transcript:KFM62390 gene_biotype:protein_coding transcript_biotype:protein_coding description:Ephrin type-B receptor 2 [Source:INSDC protein ID;Acc:KFM62390.1]
MYIEVKFSMRDCNLFPGMALSCKETFSLLYYEFDAATKEPPPWEPDSYKLIDRIAADEGR
FTSTSEVIINTEVRSVPITKKGVYFAFRDQGACLSLIAIKVFYITCPSVTLNFAYFPETP
TGHELPAIVSAEGQCVANTAVVDTPRLLCKGDGNWTLPSGGCKCNPGYEPVDQSCQLCSP
GMYKEKVGDELCMPCPLHSSASFSGSVDCQCENNFYRAPKDPKSWPCTEPPSQPLNLSET
FVDQSSVLLSWLPPKFLGGRNDTFYRVVCDKCGSTVAYIPSQQGFTDTKVTVTGLSPVTS
YNFKVYAENGVSSFTKSMFAEITITTEASVPSVVSNVRIKPLKSSEMLLEWDAPDDPFSD
TENYELRYFPKGHERNGSTLTTTKLEAVIRNLKEETLYGFQVRAKTTHGWGDYSKVIYKM
TGAKLATRELVTDEQVQVPMIVGVAITVLVLVIVVIVIIVLYIRKSSDECNKKQPSDCDT
LEYRNGEGKKLVPALVHPPIVPTAWLTTPLFTQCGASRAYIDPHTYEDPNQAVREFTKEI
DASHITIESIIGGGEFGDVCKGRLKLPCQTEVTVAIKTLKPSSSDKARMDFLTEASIMGQ
FDHPNVIYLQGVVTKSNPVMIITEFMENGSLDTFLRANDGKFQVIQLVGMLRGIAAGMQY
LAEMNYVHRDLAARNVLVNGNLICKIADFGLSREIESATEGAYTTRGGKIPVRWTAPEAI
AYRKFTSASDVWSFGIVFWEVMSYGERPYWNWSNQDVIKSIEKGYRLPAPMDCPEALHQL
MLDCWQKERAHRPTFAVIVKTLDKLMRCPESLKKIAQNRHQNPLDPNAPDMTQFKTVDEW
LDGIKMARYQENFQQAGITTMDAVTRITLKDLTALGVTLVGHQKKIINSIQTMRAQLNAN
MSEGFLV

>KNC34063 pep supercontig:ASM118794v1:JRES01000108:193395:277644:-1 gene:FF38_07520 transcript:KNC34063 gene_biotype:protein_coding transcript_biotype:protein_coding
MHAILSNACDLCQKKKMLKFVANILLSLFHRYLRIHHQKVFNMLYPYCGGHHSRSHKKYI
LPVYNVDFNNGLSTEKSNFLINNINKENSYNNNASNIQQKTSLCASTMQILTTISTKTLP
FILPLSYKKAITAKYSSSLSTYTSTSSAFIATNYLLSTPSHSSVTSQDDSCHKHLFTSSY
KPSSLNMFINTVACGGSAPSMYFPKNGQFIMKLIVIFILLIMQIGFASADQVILLDTTRE
ATLEWTRYPYGPQAQTPGWVEESFTDFVKGINWRSYVVCDVAYHNVNNWLWSPFIDRGPA
NRLYIEIQFTIRDCSLFPGNALSCKETFSLLYYEFDAATREPPPWQTDSYKLIARIAAGE
GRFNQNSDVDINTEVKSIAVTKKGVYFAFRDQGACISVLAVKVYYITCPAVTENLAHFNE
TPTGREITLIEKQNGTCVENADPYIPPTYLCKGDGKWTILNGGCRCRVGYEPDMANKSCN
ECPIGTFRSVEVSKCIPCPQNSNASKAASGYCKCLSGFFRHPRDGKHMPCYKPPGPPTNL
TLLFVDHISAILSWNTPVRDHTVDNNQNSKYRSDIVYKVVCASCSSNVVFTPSADTFNDT
KLTLTNLEPVTTYTIQIHSMNGVSYIIENATADEKSQKNSNESFSNLDKDETSKSSDDVL
IGNKASSSNNNQQIDLSQITTEWAEIVFTTTESAIQSTVSNVRIVLTTSNEIDLIWDKPV
QSDVPIEFYEVRWFPKPDFDTINKTALTTKETKAHIENLNENTEYGFQVRYKTLNGYGTY
SNIVYAHTQQGVSSVYEDNIQMRMVAGATVAVVVILVLVIVATVLFLRSKNQDDIDKKSS
NHMPLPLDYASNEVHAMDTTPIVKTIQSNVTTPLFGNSRSYVDPHTYEDPNQAIREFARE
IDANYITIEAIIGGGEFGDVCRGRLKIPPNFVQDIDVAIKTLKPGSSEKARCDFLTEASI
MGQFDHPNVIYLQGVVTRSNPVMIITEYMENGSLDTFLRVNDGKFQTLQLIVMLRGIASG
MAYLSDMNYVHRDLAARNVLVNSQLICKIADFGLSREIENASDAYTTRGGKIPVRWTAPE
AIAFRKFTSASDVWSYGVVLWEVMSYGERPYWNWSNQDVIKSIEKGYRLPAPMDCPEALY
QLMLDCWQKQRTHRPSFASIVSTLDNLARQPQALLTTRSSPENDGTHIIDGQRGHNIFIS
TDMWLENIKMSRYSQHFKEANLVTAQQISRLTAQQLSDMGITLVGHQKKILHQARQLDTI
I
>LotgiP105485 pep supercontig:Lotgi1:LOTGIsca_4:1978819:2012386:-1 gene:LotgiG105485 transcript:LotgiT105485 gene_biotype:protein_coding transcript_biotype:protein_coding
WLQGSFNQDRRGYRVYTSCYVKNANVNNWLRTPYIERGDANSLHIEVKFTMRKCSRITDP
SSLQQCKETFNLYYYEADMDIANEMRPTWDAQTYTLVDKVPARHLYEQPTEIKINTETRR
IALRPNLRGVYFAFQDEGACVTLISIKVFYVVCPNITMNFASFPETPTGDSESSLVDRQG
QCVRNAIEMQPPTYLCRSDGSWYQDPKGQCLCMPGYEGTSDSTQCTACNKGMYKWSQGKE
ACKPCPHHSYANSPGAVECNCEHSYFRSPQDEKSMACTQPPSAPKNLILESMKSTTATLL
WEPPQELGGRTDLQYQVECRLCNSQVEARPGWRAFNTTRVTLKGLAPNSQYQIVIYAKNG
ASDISGHISSADITITTDANVKIINVRVISKDSNHITLTWGVQLPNGANTAVRNYEVKYF
PINSEGHHKIKTTTEPNFTLTDFGFDTEYFFQVRGYTDKGWGVYSDPLPVSTGNKGEYEL
KDPPIGIIAGSIVAVILCMAIATIMIIILLRRNRQNCNEKKECDTPYGHVTMPLFPSPGS
IHSSVRTYIDPHTYEDPNQAVREFTREIDISHITIESVLGGGEFGDVCKGKLRVPGRPEM
TVAIKTLKPGATEKNRLDFLTEASIMGQFDDPNVIFLEGVITKNNPIMIVTEFMANGSLD
TFLRNNDGKFTVIQLVGMMRGISSGMKYLSEMGYVHRDLAARNILVNESLVCKVADFGLS
REIETDNTDGAYTTKGGKIPVRWTAPEAIAYRKFTSSSDVWSYGVVMWEIMSYGERPYWN
WSNQDVIKAVEKGYRLPPPMDCPEATHQLMLDCWQRERSHRPKFFHIVKTLDKLIRAPEL
LRKMAKPR
>M03A1.1b pep chromosome:WBcel235:II:4572000:4590951:1 gene:WBGene00006868 transcript:M03A1.1b gene_biotype:protein_coding transcript_biotype:protein_coding gene_symbol:vab-1 description:Ephrin receptor 1 [Source:UniProtKB/Swiss-Prot;Acc:O61460]
MRLYNSRILNPHQSIFILVLQCLITIVTSHQEVLFDLSKVGSDLKWDQVSLRHDRDDVWM
EETWRNPAATDEKHANQRAYVTCNYDMINPSNWLFSHFIEVKTARRIYIELLFNTRDCDA
YLNPKSCKETFSVYLKQFKTSRPGSTKIEKERFSEDIDNWKNIGRLARSNSNMTTETLGM
EIDSDTKTIRIAFEEQGICLSLLNVKIYYRICDEFTDQLVYFRPQVTGPKETDMVRMNGS
CIPNASKKIPGVDLIGLCMSTGSGIKTSGECVCDSGYSQIADSNGARCESCPTNTYKPKG
QSLCKSCPSNSISSEAASSCRCLNGYFRAEDELISMPCTQPPSRPIKLVANAITATSTRL
SWNEPSSLGGRPEIWYEVKCSGRGECGTVVMTPGDKKLSTRSVQINGLRPSSDYTFLVFA
KNKVSAQFPEFSEKNAVIDIRTRSEEEDVPPVSHLRVDASQSDGITIAWSVSDSDVSDFE
VEVRPAIVKKRTFETRHVNMTYTTFIGLNPETVYQFRVRIRDDLRWSQPISYQLGRGLMS

SPSSNEVEESQFLNQTGSALLIIIALILIVIAVALCMIVVQKKSKNRKQMSDLDVLDTYK
QDSMTPDYHTTSRHHHHQGNLPATLHEQLRSTTKLNAPLIPSFGSPISQPPPYYGGVHPN
SGKYKTYVDPTTYEDPYQALIEFTFDISPNDVFITQVIGGGEFGDVCLGGLSKNSPAAAK
WSVSNTTMGRGGGGGGYESEPYETVAIKTLKSGSSAKAKAEFLTEATIMGQFSHPNVIRL
IGVVTSAEPVMIVAEYMANGSLDQFLRNTDQRGEKVHWEKITEMLYGIASGMKYLTDMGY
VHRVSFLRDLAARNVLLDMELRCKIADFGLSRGVRSEGSSVEPEYTTNGGKIPVRWTAPE
AITHRKFTPSSDVWSFGVVIWEVCSFGERPYWDWTNQKVISEVMIGYRLPPPMDCPMGLY
RIAQWCWKMERHERPTFTQLLATFHKYILQPTLIEHDPGELPRRVQSQSALNTYGSVNVG
VVPTPPSSAAPMPSLDDFLRQIGLNHVYGQLVSNNIHSVSDLANTSHLDLLAYGLMSAEC
STVRDGLNGRISGSPPGSSGTIHATTRGTRTTRPPREEGFFV
>MESCA003372-PA pep scaffold:Msca1:C780742:9168:21855:-1 gene:MESCA003372 transcript:MESCA003372-RA
gene_biotype:protein_coding transcript_biotype:protein_coding
MTESAKNLSSFGNYLNDYELQMTSDKTDSTDKHKKILEIAVKPDYTMERGSWSSKLDFIL
SVVGLAIGLGNVWRFPYLCYKNGGGAFLIPYFVTLILAGIPMFFMELALGQMLTIGGLGV
FKIAPIFKGIGYAAAVMSCWMNVYYIVILAWAIFYFFMSMRKDVPWRTCNNSWNTPACVN
PYERQNLFCWNSDETKICALAGQNLTFSDLTDPVKEFWEGITLPGAIEGIKFYVIPNFSK
LSQSEVWIDAVTQIFFSYGLGLGTLVALGSYNKFTNNVYKDALIVCSVNSSTSMFAGFVI
FSVVGFMAHEQNRPVAEVAASGPGLAFLAYPSAVLQLPGSPLWACLFFFMLLLIGLDSQF
CTMEGFITAIIDEWPQLLRRRKEIFIAIVCGLSYLVGLTCISQGGMYVFQILDSYAKKCL
ANQIIIQKKMKYSIFLTICFLNSTISDQVVLLDTTKEATLEWTRYPYGPQTQTPGWVEES
FTNFGRGINWRSYVVCDVAYNNVNNWLWSPFIDRGPANRLYIEIQFTIRDCSLFPGNALS
CKETFSLLFYEFDAATREPPPWQPESYKLIARIAAGEGRFNQNSDVDINTEVKSIAVTKK
GVYFAFRDQGACISVLAVKVYYITCPAVTENFAFFKETPTGKEITLIEKQNGSCVENAEI
DEMPPTYLCKGDGKWTILNGGCKCKVGYEPDFEKQSCKEANSLYIVQVYSHSKEDIIGNY
SEISFTTEYTISTEILKVYIEKVFENEIYLKWDKPLSSIEFYEVKWFIKGGDIFDMNKTS
SLRTKELETVIENLLENTEYGFQVRWKTTNGYGPYSNIIYATTQSGIHTVYDDSAQMRIY
AGATIAVVIVLVLIIFATVFFLRTKNHDELEKKTTNHLPLSMDYASNEVNSLDTTPIVKS
KANELSAFGHPFPPFSQMSTTDVECFQGKIFYNSDCSAVIIDPHEKHAKTEKKINQSQPP
PPIIHTFRVVTASDVEKNVDSQKSIPQKPILMCFICKLSFGNVQIFQQHSNTEHGCHYTT
KRTRESPDIVFRTNCDGRHKEDDAATAAATNFLSLAAFSNIFQYSQQKNNLNVCQEHGNV
KGNDCKACELSILRTSPSSTTPSFTIGACPDHVNGRPLGVECSRCELILSSSRLTTNSQT
MSSTRNSCKTLKCPQCNWHYKYQETLEIHMREKHPDGESACGYCLAGQPHPRLARGESYT
CGYKPYRCEICNYSTTTKGNLSIHMQSDKHLNNMQELNTTQNINLTINGPPAPNIQPQVS
SVGAQPTAQSPVSSQSSNAKAKPNFRCDVCSYETSVARNLRIHMTSEKHTHNISVLQSNM
KHFQTLNLIQQQQITNILPDNFSNNLLPEAAIADMAYNQA
>Mdes004538-RA:cds pep supercontig:Mdes_1.0:GL501545:1727195:1738494:1 gene:Mdes004538
transcript:Mdes004538-RA gene_biotype:protein_coding transcript_biotype:protein_coding
MDHIDRINCSRLPTAIEIKNSDINTIPTKNSRRPPQHKNSSSKHTKLPFDKYTNEKYSRH
SILPFKQCNEHRTHLKRLERPNNQLHHNYHHLHEFPESTTRTSTRFNFKQNNQYFSRFYL
IFSFVLIYLLDKINCDQVVLLDTTKEATLEWTRYPYGPQAQTPGWVEESFTNFVKGINWR
SYVVCDVAYNNVNNWLWSPFIDRGSANRLYIEIQFTIRDCSLFPGNALSCKETFSLLFYE
FDAATREPPPWQPESYKLIARIAAGEGRFNQNSDVEINTEVKSIAVTKKGVYFAFRDQGA
CISVLAVKVYYITCPAITENFAYFNETPTGREITIIEKQTGVCVKNAESIEPPTYLCKGD
GKWTILTSGCKCKVGFEPDYEKQTCTVCPAGTFRSVEVTKCTPCPLNSKATKSGSAFCAC
IDGYYRHPRDSLHMPCYRPPSKPTNLTLLFMDQTSAILSWNAPQRPTNELYDSKYRSDIV
FKVKCSACGSNVVFIPATETFNDTKLTINNLEPVTTYTIQIHSLNSISYAIYDVDSNVDV
NVSDVQNHANSHANSNSGSVSTNDPPSEYAEITFTTESAILSMVFNIRIISITHNEVDLA
WDNPMHTEVPIESYEVRWFPKMELDAVNKTTLSTHEQRAHIDNLIENTEYGFQVRCKTAN
GFGQYSNIVYGQTHHGVSPVYDDSIQMRILAGGTVAFVCILVLVIVASVWFLRSKNQDEL
DKKANNHLPLPMDYASNEVHAMDTTPIVKTIQNNGYDGYYRQRRNFPVTTPLFGTNRSYV
DPHTYEDPNQAIREFAREIDASYITIEAIIGMNNACTMGLGSSMVGIIDAKERVAFVNAD
LWLESIKMSRYSQHFKEANLVTAQQISRLTAQQLSDMGITLVGHQKKILHQARQLDSIM

>OVOC11316 pep supercontig:ASM49940v2:OVOC_OM4:13638447:13640621:-1 gene:WBGene00248125 transcript:OVOC11316 gene_biotype:protein_coding transcript_biotype:protein_coding gene_symbol:OVOC11316
MDSIWLEETYRGSEGIDNRRAYVVCNVDQPNVDNWLRTPMINVKGANRLHIEVTFTMRDC
SEFPGNARSCKETFRLYAVQLMKNEQYQNVWNSGYWDLIDRITADTGRYSKHDPTTATVN
QEVRSYAVTKDAVYFAFRDSGACISILNVKVLSFNYIY
>Ocbimv22021576m.p pep supercontig:PRJNA270931:KQ416048:80972:81646:1 gene:Ocbimv22021576m.g transcript:Ocbimv22021576m gene_biotype:protein_coding transcript_biotype:protein_coding
WLLGSSSYAGETVQAYSSCYVTSPNINNWLRTPYIERGDAERLHIEMTFTMRKCVRHNDP
SSLQQCKETFNLYYYEAESDFANHQMPTWQPNVYSKIDTIAADTLYVQPRKVQVNNETKH
VTIKRKGVYFAFQDTGACITLLSVRIFYVTCPNVTVRYAFFMETPTGPTVSSLVQKKGVC
VGNAVEESNPTYLCRSDGSWDILMGSCKCLPGYQGINGTKCVSK
>PHUM576020-PA pep supercontig:PhumU2:DS235870:166885:181359:1 gene:PHUM576020 transcript:PHUM576020-RA gene_biotype:protein_coding transcript_biotype:protein_coding description:Ephrin type-B receptor 2 precursor, putative [Source:VB Community Annotation]
MLDLCAWIEESFTNFEKGINWRSHVVCDVTYNSVNNWLTTPFIERGPANRIYIEIKFTIR
DCSMFPGNALSCKETFSLLYYEFDGAKPEPLPWEPDKYKLIGRIAAGEGRFNVNSDILIN
TEVKSVPVTKKGVYFAFRDQGACISLLAIKVYYITCPETTINFAHFPATSTGKEVTVIEQ
AMGKCVENAQEVGKPTYLCKGDGKWYLPTGGCKCNPGYQPDFNKKTCNVCPPGKYKHSTG
DDKCQGCPEHSKAPDYGFSECRCNSGYYRAAKDPKDMPCTQPPSAPQNVTSYFVDPSTVI
ISWNPPAQRGGRNDTVYRVLCEGCGVSVSYIPNTDFFNDTKITLTGLNPVTTYKFQVYAE
NGVSDLVGEKSQFTEIIVSTEASVVYNVANLKVNSVKSPEVVLSWDAPPPTTNDAADEVE
NYEVRYFIRNDNGNASTKLTKDTTITLTDLKQRTDYGFQVRAKTNRGWGDFSPIVFQTTR
QVLHYVGEDDNIQVRIIAGATVAVVVLLVIIIIMTVLFLRSRGSDECNKKQPSDCDTLEY
RNREVTTPLVTQFGSSSRTYIDPHTYEDPNQAVREFAREIDAGCITIEAIIGGGEFGDVC
RGKLKLPPDGRSEIDVAIKTLKPGSSDKARNDFLTEASIMGQFEHPNVIFLQGVVTKSNP
VMIITEYMENGSLDTFLRANDGKFQVLQLVGMLRGIASGMQYLSEMNYVHRDLAARNVLV
NSTLVCKIADFGLSREIESTTEGAYTTRGGKIPVRWTAPEAIAFRKFTSASDVWSFGIVC
WEVMSYGERPYWNWSNQDVIKSIEKGYRLPEPMDCPEAIYQLMLDSWQKERTHRPTFASI
VNTLDKLIRCPDTLRKISQNRTMNPLAPDAPDLTTLSSVDEWLTSIKMARYLENFAQAEI
TTMDDVCRLTVKELTALGITLVGHQKKIMNSVQVLRAQISANLSEGFLV
>RPRC012412-PA pep supercontig:RproC3:KQ034221:334256:391020:1 gene:RPRC012412 transcript:RPRC012412-RA gene_biotype:protein_coding transcript_biotype:protein_coding gene_symbol:EPH description:Eph receptor tyrosine kinase [Source:VB Community Annotation]
WVEESFTNFEKGINWRSYVVCDVAYHSVNNWLWTPFVERGNANRIYIEIKFTIRDCSLFP
GNALSCKETFSLLYYEFDAATREPPPWEPESYKLIGRIAAGEGRFNTNSEVIINTEIKSI
PVTKKGVYFAFRDQGASLKIIGHSVYYITCPEVTINFAKFPTTPTGREVTFIEQATGRCV
DNAEEVETPTYLCKGDGKWYLPSGGCKCKPGFEADVEKQTCNVCPPGKYKHATGSDHCQP
CPPHSKAPDYGFSECRCNSGYYRAEEDPKSMPCTQPPSAPQNITVNFVDQSTVILSWTEP
SFLGGRTDTVYRVQCDVCGQAVSYIPATDTFEDTKVTISGLSPVTNYRFQIYSTNGVSEA
AGTDGEYIYIDVTTEASVTSASVHNVRVTAVKAAEISLAWDPPISPDPEVDVLVETYEVR
CFSKQEDGNATSKLTEEQKVTFTSLRQRTEYGFQVRAKTTHGWGDFSMPIYKTTGQVLGT
AYIGDDDNMQVRIIAGATVAVVVLLVIIIIMTVLFLRSRGNDECNKKQPSDCDTLEYRNG
EVTTPLFTQVGSTGSRTYIDPHTYEDPNQAVKEFAREIDASYITIEAIIGGGEFGDVCRG
KLKLPPDGRSEIDVAIKTLKPGSADKARNDFLTEASIMGQFEHPNVIFLQGVVTKSNPVM
IITEYMENGSLDTFLRANDGKFQVVQLVGMLRGIASGMQYLSEMNYVHRDLAARNVLVNA
QLVCKIADFGLSREIESTTEGAYTTRGGKIPVRWTAPEAIAFRKFTSASDVWSFGIVCWE
VMSYGERPYWNWSNQDVIKSIEKGYRLPAPMDCPEAIYQLMLDNWQKERTHRPTFGSIVK
TLDKLIRCPDTLRKVAQNSIYFTTSPALAPSSLYSICKLYLPSKVSQTGNPLAPDAPDMT
QFSSVSDWLASIKMSRYAENFEAGGVTSLEAVVRLTVQELTSLGVTLGHQKKIMNSVQAM
RAQISANLSEGFLV
>SMAR002974-PA pep scaffold:Smar1:JH431264:114482:125163:-1 gene:SMAR002974 transcript:SMAR002974-RA gene_biotype:protein_coding transcript_biotype:protein_coding
MSQDVISVVLLDTTQEASLEWTRYPYGPQALTQGWVEESFTEFDKGINWRSYVACDVAFD

TVNNWLWTPFIERGEANRIYIEVKFSMRDCSLFPGTALSCKETFSLLYYEFDAATKEPPP
WEPESYKLIDRIAADEGRFTSNTQIIINTEVRSVPVTKKGIYFAFRDQGACISLLAIRVY
YITCPNVTTSFAYFPETPTGREVTSIEQAKGQCVTNAAQAETPKFLCKGDGSWYLLSGGC
KCMAGYEANIERQSCEVCPLGKFKYTVGDEKCQPCPDHSNAPYSGSVECRCEEGYYRAPK
DPRSMPCTQSPTAPQNLTLSFVDQSAVILSWTSPLYLGGRTDTVYKVVCDACGPAVSYVP
SQERFNETKVTIAGLNPVTTYRFLVYAENGVSEFSKTNKYVDITVTTEASVLYFIVPSVV
SNVHVVNVQNTEITLKWDAPDDQFSEIEMYEVRYFIRGMERNASSILTKHEESVFTNLKQ
RTEYGFQVRAKTTHGWGEFSQPEYRRTDQMLGTAYVGDDDSVQVRIIAGAVVAVVVLLVI
VIIMTVLFLRRSNEDCNKKQPSDCDTLEYRNGEVTTPLFTQVGPPRTYVDPHTYEDPNQA
VREFTREIDASHITIEAIIGGAPDAPDMTQFATVEEWLGSIKMTRYLDSFDRAGVSTMDA
VARLTAKDLNNTIGVTLVGHQKKILNSVQAMRAQISANMSEGFLKQCQQGLLAQLKQCQQ
GLVRPVNSDG
>SPU_027145-tr pep supercontig:Spur_3.1:Scaffold1381:67008:111610:-1 gene:SPU_027145 transcript:SPU_027145-tr gene_biotype:protein_coding transcript_biotype:protein_coding gene_symbol:SP-EPH description:Uncharacterized protein [Source:UniProtKB/TrEMBL;Acc:W4ZG41]
MDNLRNCIIFIVLCAWTSYAKEVKLYDSNAFSGLYWTVYPPQDPDSQTYVGWTESGASYE
RIFQTCHVSDPRDNWLRMPYIERQGANRIHVEVKFTMYSCTGIVDAQLCKETFDVYFYQS
DTDEATETSPDWTAPPYQKVARIAAEGRFSDPDTLDEEVVNVRIENFGPVTANGFYIAFR
DQGACMALLQVRIFYQVCPQVVRDFAIFNQTNEGPETHTLVTVPGTCVKNAQPVPPGSTP
QYICQNEGIWSLNQGGCGCSPGYEASADRTRCEECQIGTFKSDIGTTECSPCPAKSHADT
VAVTRCTCIPDYYRAPGEEASSPCTAPPSAPTSLTAVVDEKSSVVTLTWRNPENLGGRTD
IDFRIDCKRCPADVQFSSIQRIGQGYEVNVSRLSSHTTYEFKVHSYNEVSSQGTVDNYAG
VNATTFSSVPSAVRDLKVVEDDVNDESIKLRWRQPLFPNGEITNYQVIYRTSAETQENVK
YHYQAVLDGEVQETTVHGLVPGTEYVFEVAANTASGVGAYSQQVLATSSGSGGQSVPTVI
LAAAVSAIFIIVVLVAVVIFLVFWRRWKAKQQLYYQISTLPPRKKEVPPSENGHLIYANG
TNGEAITFGHSTVILPHIKKVKTYVDPFTYEDPNQAVKEFATEIDATQIRILEVIGGGEF
GDVCSGLMLMPDKTTIKVAVKTLKTGATDKDRSDFLSEASIMGQFDHPNVIKLLGVVTKT
RPAMIVTEFMENGSLDKYLKENDGRFTVTQLLGMMRGVGFGMRFLSEMNFVHRDLAARNI
LVNEHLVCKVADFGLSRRKELDGAYETRGGKIPIRWTAPEAIGYKKFTSASDVWSLGVVM
WEIMSYGERPYWNWPNQDVIKAVEKGYRLPPPMECPEAIHQLMLDCWQRDRNHRPTFTTI
VSTLDRLIRNPNALRPLAKNGSPLGLSSSLNDLSRFNSVSDWLDSLKMGRYKDSFTAAGY
VRLEDIARLSQSDLPRLGVTLAGHQKKIMKGIHSIRAQLEQAAETLV
>SRAE_X000163000 pep scaffold:S_ratti_ED321_v5_0_4:SRAE_chrX_scaffold2:2943595:2947601:-1 gene:WBGene00267204 transcript:SRAE_X000163000 gene_biotype:protein_coding transcript_biotype:protein_coding gene_symbol:SRAE_X000163000 description:Receptor protein-tyrosine kinase [Source:UniProtKB/TrEMBL;Acc:A0A090KXA9]
MKTIYFWKYTTLLYFLIYLVDSAKVLLHDSYVYKEELPYEYFSENSEKSDHPTWEQESYY
DEDKNPIVVSTVCYEGNKPNFVKNWIALPKVEKGSAKKLHLEISYSINNCSQTRPFCKDY
FDVFYMPLNGDDNTKLKIKNIDNWKHLKNLNIHSGKVTKGVQDFIVTTIDYTPESDAVIF
GIREENTCSSILRIRIYFKICPSQTINQFQLPETVVGEDLYGPTFYHVDCVKNAVMLGTK
PTMKNAFCKANGEWEVLTDNTCQCLAGYSPNDSGSCSPCTKGSYKNTIGNGICKACPRNS
HAYDNGTHICECDIEFYRADNEDASHQCSQPPSSPVNVRTLELKSSEAIIIWSNPTYMGH
RSDIYYSITCEFCDESKHCVKCSKSVTSSFNPERIETKQITLKNLEPGRDYIINVFSHNA
VSKKAAGNLHNKAIFTFTTAKPFNFKMNTPKLLSHLNNGTLLISWEPYDDEKYLKLGNMY
YQIEIKNDKDIVLMDSIQTALKIENIDIYKSHIIKIRVHDPKKGWSEWSDELFIKNKYNK
GYKISNQQYESFSNDMIIENEKSELSLYMSITQLYSQNPKIFYIIGCTIVVLLIILNILY
FCYTKNNKKGDYFAELVKSQQSDIPYNINITPSHLYYGKQFDSQFLNGFGSATTFVDASA
NKTYVDPSTYGDLSVAVKEFTKQIPKSNIFLTGEILGGGEFGDVQKGILLLTGSTKSRNI
IDGNELIVAVKTLKKCVTEQEKKYFTMEATTMSQFHHDNVLRCIGVVGTDYVEMIITEYM
VNGSLINFLKTISGDTYSKVKLAHMCLDVAEGMQYLHMKGYIHRDLASRNILLDQFYRCK
IADFGLSRNGFNNENVELEYTNAMAKKIPIRWTSPEALEKGLYTAASDVWSFGILMWEIF
TFGERPYYDWSHRKIYEEVLQGYRLPKPDDAPEILYRVMVECWIYDKNERPTFTAITNEI
KSYIRIYEDNGDFLANSPLYQSPSDVNYDVARDGNCNSRISNSNYFFTSHPNVSQLPLNT

SHRMSNWDIARHSLGDLEKQELENALGKFNIQYLIHHLYRMKIFSVAQLANLSLARLADM
DITLNEQKQLAVVIDHIVTSRRAVTNVSRNLKNNQAPAIPQFTKTLNKDSPDGGFLV
>SSCA007952-PA pep supercontig:SscaA1:JXLN01013880:25878:28751:1 gene:SSCA007952 transcript:SSCA007952-RA gene_biotype:protein_coding transcript_biotype:protein_coding description:Ephrin type-B receptor 2-like protein [Source:UniProtKB/TrEMBL;Acc:A0A132AF98]
MRDCNLFPGMALSCKETFSLLYYEHDGLSKEPPPWEPESYKLIDRIAADEGRFTSNNEVI
INTEIRSVPVTKKGVYFAFRDQGACLSIIAIKIFYITCPNITTNFAFYPETPTGRELTTI
VETDGECVANAQQVEVPRLLCKGDGNWTLPSGGCKCMPGYEPMGQSCQVCSPGKFKPIVG
DDLCQPCPKHSKSSYRGSSECRCDVGYYRAPKDPKWMACTQPPSAPQNLTISFMDQSTVI
LTWSEPKYLGGRNDTVYRISCDYCPTTTTYVPSQENFNDTKITISGLNPMTSYHFQVYAE
NGVSGYDSSQFVEITATTEAAGMFWNIRKSSKKIA
>Smp_124210.1:pep pep chromosome:ASM23792v2:1:6494040:6556873:1 gene:Smp_124210 transcript:Smp_124210.1 gene_biotype:protein_coding transcript_biotype:protein_coding description:Putative ephrin receptor [Source:UniProtKB/TrEMBL;Acc:G4V6K2]
MKSCLQLGYLHQSIECKEHLDILAYHTDVHLSSMDFTELHILTFQEGESFTNELITSSSL
KRVTIQIRPKMKWLQIAFRDNGSCVLIDRLIAYHLSCPATKFRLINLPETEAGHNKEVRQ
IHVQCIPGSIFVNQYSIYTKSNDHLIHNNKLLNNLNDENNGLALCMADGTWHLQTNHGCV
CDAGYELVEHTETCQACPRGMFKSSPGLQPCSICPLNSIAPHAGFRICHCLSGYFRIAPN
LSAEHSCLGPPSAPRNLNAIHINGTSVVLSWDPPIRSGGFHETLYHVQCQGCQIDTVKYQ
PGNHVNETKVTITGLNPQTRYQFDVYAHNAVSTKTGTMWINVASVMVTTGSDFLFNLFKP
EKNKNKMKSVLLIDNIPVNIIPYHQIRIISQLSENRCGSTYMAKLFNYNLPTPSVMMTSF
SRSHDSGTIYSLKNIGPTDNDSKINNIMISNIVQNKWNQDFQKSPNDNHPILTLKNNNDH
LLVMNDFTELHNDKLITSSHPDINNSHYIYVFIQDFSHLERLRNRQYDYKNLFSKVPWIQ
RRQSSSTSIEVHRKIKKFLGLNEKLMKFIKQYTQFDHVNIVKFYGLSIIEISKAYSLCSM
ITEFCINGSVDMYLKNVLQGNQPITLDNNNHTISFNLSQTIKMLLQILSGLEYLTLNSYT
VENFTSKSVFLDGCFNCKLKIQFNSVVCSNKQYPTIDNNKINDGFFQPLLVVIIGISEDY
>TC033957_001 pep chromosome_group:Tcas5.2:LG8:2651389:2682756:-1 gene:TC033957 transcript:TC033957_001 gene_biotype:protein_coding transcript_biotype:protein_coding description:Ephrin type-A receptor 4-B-like Protein [Source:UniProtKB/TrEMBL;Acc:A0A139WE15]
MVMMRLLALVLTLIHYKTADAEQVVLLDTTGEPTLDWTRYPYGPQANTPGWVEESFTNFQ
KGINWRSYVVCDVAYNNVNNWLWTPFIDRGVANRIYIEIKFTIRDCSLFPGNALSCKETF
SLLYYEFDAATREPPPWDADRYKLIGRIAAGEGRFNSNSEVNINTEVKSIPVTKKGVYFA
FRDQGACISLLAIKVYYITCPEVTINFAKFPATPTGKEITLIEQATGSCVPNAEIVGGTP
TYLCKGDGKWTLPTGGCKCKAGFQPDVEKQTCNVCGPGMYKAEVGDENCLPCPGHSTGND
YGLSECRCNNGYYRAPTDPKNMSCTQPPSAPQNLTVNFVDQSTVALSWAPPDNLGGRTDT
VYRIKCDACSLGLVQYNPHTDTFNDTRVTISGLNAVTTYRFQIFAENGVSFMRTEAPEYA
DVTVTTEALVASSITNVRVSSVMSSEITLAWDAPSAAGDGNDIENDLVETYEVRWFPRSD
MDYSNASSLLTTDLFATITGLQQRTEYGLQVRAKTQRGWGAYSPVIFKTTGQVLNTAYVG
DDEGIRLQLVAGSIVVVVVVLVVIIVFTVMFLRSRSNDECNKKQPSDCDTLEYRNGEVHH
SLDNPPIVTTHTNVTTPLFTGISGSSRTYIDPHTYEDPNQAVREFAREIDASCITIEAII
GGGEFGDVCKGKLKTNGIDIDVAIKTLKAGSLDKARNDFLTEASIMGQFEHPNVIFLQGV
VTKSNPVMIITEYMENGSLDTFLRANDGKFQVIQLVGMLRGIASGMQYLSEMNYVHRDLA
ARNVLVNSQLVCKIADFGLSREIESATEGAYTTRGGKIPVRWTAPEAIAFRKFTSASDVW
SMGIVCWEVMSYGERPYWNWSNQDVIKSIEKGYRLPAPMDCPEAIYQLMLDCWQKERTHR
PTFQSIVKTLDKLIRVPDTLRKIAQNRHLPPCLNPYFRQDLRHVSELPARESFFDYGLPD
VILLQNYEMSQWLSQERFNLSSDFGSSDQTLFTYQGDTHTPSHTYTKRGAWVTFSNM
>TDAL011489-PA pep scaffold:Tel_dalmanni_2A_v1.0:NLCU01000505:19913:33302:-1 gene:TDAL011489 transcript:TDAL011489-RA gene_biotype:protein_coding transcript_biotype:protein_coding
MHEQIMKQHFSWKNNVDQFVFKNCLNDQICRRNFKLRKKLCEYNQKYKEENDSELKSIAK
TGTLIISTSPPHRTSNTFSIKYFTSSLPIRLSNSFSYSTQSNTTKNFMLSLIFNSVHDLI
ITGSLLTLMTLLGNQYYKTHLEKIVLFTTQMLPFCTGITCKIQLRLHSLLFAWFWLVFTL
HIGFGSADQVILLDTTREATLEWTRYPYGPQAQTPGWVEESFTDFVKGINWRSYVVCDVA
YHNVNNWLWSPFIDRGPANRLYIEIQFTIRDCSLFPGNALSCKETFSLLYYEFDAATREP

PPWQTDSYKLIARIAAGEGRFNQNSDVDINTEVKSIAVTKKGVYFAFRDQGACISVLAVK
VYYITCPAVTENFAHFNETPTGREITIIEKQNGTCVENAEPYEPPTYLCKGDGKWTILNG
GCRCKVGFEPDNINKTCNECAAGTFRSDEIANCIQCPPNSNALKSGAAFCKCLPGFYRHP
RDGKHMPCYKAPGPPTNLTLLFVDQTSAILSWNSPVRDSAESVFNIKNTKYRSDIIYKAR
CSSCSPNVVFNPSTDTFNDTKLTLTNLEAVTTYVVQIHTINGLSYIESEIYLNDTIVGNT
AVETLGISDTKPITPKRSQQSIDFDNIKTEYAEISFTTESAILSTVFNVRVLSTTSNEVD
LVWDKPVQSDAPIEFYEVRWFPKSELDAINKTALNTRETKAHIEGLNENTEYGFQVRCKT
LNGFGTYSNIVYAHTHQAVSSVYDDSIQMRIIAGATVAVVFILVLVIVATVLFLRSKHQD
DLDKKSSNHLPLPLDYASNEVHAVDTTPIVKTIQSNVTTPLFGNSRSYVDPHTYEDPNQA
IREFAREIDANYITIEAIIGGGEFGDVCRGRLKIPPNFVQDIDVAIKTLKPGSSEKARCD
FLTEASIMGQFDHPNVIYLQGVVTRSNPVMIITEYMENGSLDTFLRVNDGKFQTLQLIVM
LRGIASGMSYLSDMNYVHRDLAARNVLVNAQLICKIADFGLSREIENASDAYTTRGGKIP
VRWTAPEAIAFRKFTSASDVWSYGVVLWEVMSYGERPYWNWSNQDVIKSIEKGYRLPAPM
DCPEALYQLMLDCWQKQRTHRPTFSSIVSTLDNLARQPQTLLTTRNSPETDGTHIMDGQR
GHNIFISTDLWLENIKMSRYSQHFKEANLVTAQQFFR
>TriadP53296 pep scaffold:ASM15027v1:scaffold_2:3550505:3552372:-1 gene:TriadG53296 transcript:TriadT53296 gene_biotype:protein_coding transcript_biotype:protein_coding
MKTFTAFFYLLLVHSSYTLAAKELLLDTNKYQAANLGWPGWKSCTSPPGHLGNWAYTKFL
NETVGKYTICYQGHHQRAWLISPYIPFGVAKKVYVNIEYSTKSNCSIRSKNCTSFLYFIG
LQFQENIGNQFCEKSELYSRIRDTLDLEEIIHAKESSLLADQKGRKVSLLGQHRKFAAFV
TVQENMNGMYLFLDGKGSCSIIQNVQVWYKVCHPKQIGNVYFPAAATEQTGTNVSYSNGS
CTGKSQLVDDKVQPIMSCFPNGTWSKPQNLQSCACKAGHELQNNICQRMSENIIVVIIVF
DLYLSYIFI
>TriadP56933 pep scaffold:ASM15027v1:scaffold_5:7869232:7875658:-1 gene:TriadG56933 transcript:TriadT56933 gene_biotype:protein_coding transcript_biotype:protein_coding
MGLTVFLLQSGYQTANRPPWNQWQNCPVNVNSNQGDSVKFELKQLGVVQYYYICNQVRNQ
RTWLISPYFGLNSAKEIYIKIRYTNTQVCPQNRFCASGLGIYASVLFDPIDGNICQTAVL
KIFKGLVESQHQDRPIRTIPQPYKYRDTSNVSPQQTPIPFLIAREWRLANMHITIEDRGT
CSEIEAVSVWYYACPLMNYPDAFFPRTVAPAAMNGSITVTGICSGSSTNLPYGLKTTAEC
QPNGKWANYVNNCPCNAGRERIFVSCQDCKFNYYKSLPSSGKCERCPNSMITIRKGAREC
VCLKNSILHISGECITMPVCKLFYLAVKNTSAILQFSRIEEQITRNMSFDLECFNCSLKD
INFRRYTVYTANITLDKQLMVTNLKPATSYALRVVPWIHIVDGKQKKTPCETVIFRTLDG
VPGPPTKVKTQSKKGNPLVHVSWKPPTLPNGAIRRYLIMFSLKEAFNATAEDLEKDVIVE
VDHTVLFYNISTLPVRTVFHVTIRAATVHGTFGPPSVTRNVTVYERDRTTSSVIGSVTGG
AVATGLGVFALKFGMMAKDGVSAGKIIAVVDKCNYT
>TriadP56934 pep scaffold:ASM15027v1:scaffold_5:7878428:7883892:1 gene:TriadG56934 transcript:TriadT56934 gene_biotype:protein_coding transcript_biotype:protein_coding
MVLILGYILQYFFLCITLTRSEQDTAVSEYQWSRWQSCSEEVLTNQTNNNDQLLWQLDNP
IYMCDYSSDQISWLISPTFAVLPNKKITVHIQYIKAPNITVGDDSIPELQAIVVPSAKVS
ESQFCQQTSQVSDKYIQLKRESNIATELALEKEKNLKNHAHHLQNVEEMDMDIKSTPKLV
YARLIIQNNRSCSQVNSVRIINTVCEKIIAEGAIFPESNPPSVQNGPKLVIGHCTGSVVA
NVSKPVAWCQPDGNWSHFINACRCPRGKERRQQYCVKCQINYYKPNSSALPCLPCPANTS
TTVRGSVKCICNSKQIKLNSQLPCQPIPKCKVRFSSANTSSLIQVLSFGSRKTRAMTFRL
QCRNCSVSGIQYNRNLKLKTRIKINSLLPNAMYVIKISPIVIANGKKYLKMPCATLKIKT
KDGVPGAPRQVHVLQDKFYPLIYITWKPPRVSNGLIIKYRILCRRLLENHATLSTLAKEE
IFVQEVDGVVQFANISHLRPRSHYSITISATTKLGQYGPQSYPAFIHIGPVISDDTKTDA
IRLSLAPLIIGGFTAGAMLIAVLIVILKFGGIPKFSLNDGLSAICRNYWINNYCCCCRRR
SAFSDTSDKNELDETDLEDTII
>XP_003400363.1 pep chromosome_group:Bter_1.0:B14:3431:97745:1 gene:LOC100645846 transcript:XM_003400315.3 gene_biotype:protein_coding transcript_biotype:protein_coding
MAPFNMAGVAGLLATCAAAAASAAHLLPLLLLLICPRGTHAEQVVLLDTTQEEKLEWTKY
PFGAEANTPGWVEESFTNFDKGINWRSYVVCDVAYNNVNNWLWTPFIERGPANRMYIEIQ
FTTRDCSLFPGNALSCKETFSLLYYEFDVATKEPPPWETDSYKLIGRIAAGEGRFNTNTG

VVINTEVKSIPVTKKGVYFAFRDQGACISILAIKVYYISCPEISVNFAHFPATPTGREVA
LIEQTIGTCVDNAVVIEQPTFLCKGDGKWYLPNGGCHCKPGYQADVEKQACTECAIGKFK
HEAGSHSCEACPAHSKSSDYGFTECRCNAGYFRAEKDPKKMPCTQPPSAPQNLTVNFVDQ
STVILSWNAPHMLGGRTDTTYRVVCDACSMGVKYIPNTEVFNDTKITITGLNAVTTYRFQ
VFAENGVSALAGKSEYVDITVTTEASVPSLVSNVRITSVKSSELSISWDAPVTEVGGDSD
LVERYEVRCYPRYDDATNATVIQTSELSATFKGLKPSTDYAIQVRAKTTRGWGEYTPIVY
KKTPHAMGLDYVGEDDNMQVRIIAGAIVAVVVLLVIIIIMTVLILRSRASDECNKKQPSD
CDTLEYRNGEVTTPLFTPAVGVAAASAGGAGGGGARSYVDPHTYEDPNQAVREFAREIDA
GYITIEAIIGGGEFGDVCRGKLKLPPDGRTEIDVAIKTLKPGSADKARNDFLTEASIMGQ
FEHPNVIFLQGVVTKSNPVMIITEFMENGSLDTFLRANDGKFQVLQLVGMLRGIASGMQY
LAEMNYVHRDLAARNVLVNAALVCKIADFGLSREIESATEGAYTTRGGKIPVRWTAPEAI
AFRKFTSASDVWSMGIVCWEVMSYGERPYWNWSNQDVIKSIEKGYRLPAPMDCPEAIYQL
MLDCWQKERTHRPTFANLTQTLDKLIRSPDTLRKIAQNRGTNPLAPDAVDLTQLTSVSEW
LASIKMSRYAESFERSGVTTLEAAARVTVQELTALGVTLVGHQKKIMNSVTALRAQMSAT
SQGFLV
>XP_011157319.1 pep supercontig:Si_gnG:Si_gnG.scaffold02162:314000:419612:-1 gene:LOC105194212
transcript:XM_011159017.1 gene_biotype:protein_coding transcript_biotype:protein_coding description:ephrin type-B
receptor 1-B, transcript variant X3 [Source:RefSeq mRNA;Acc:XM_011159019.1]
MAIRMIPFNMAGVAGFLATCAAVAASAARLLPLLLLLIYPRGTPAEHVILLDTTTEEKLD
WTRYPFGPQANTPGWVEESFTNFDKGINWRSYVVCDVAYTNVNNWLWTPFIERGPANRMY
IEINFTTRDCSLFPGNALSCKETFSLLYYEFDAATKEPPPWEPDSYKLIGRIAPGEGRFN
TNTEVMINTEVKSIPVTKKGVYFAFRDQGACISILAIKVYYISCPEVSANFAHFPATPTG
REVALIEQSRGVCVANAVKIAQPTYLCKGDGKWYLASGGCHCKPGYQPDEAKQECKECPI
GKFKHEAGLTTCEPCPEHSKAPDYGFTECRCDPGYYRAEKDPKKMPCTQPPSAPQNLTVN
FVDQSTVILSWNAPHMQGGRSDTTYRVDCDSCSMGVKYIPNTEIFNDTKITITGLNAVTT
YRFQVFAENGVSSLVGKSEYVDITVTTEASVPSLVSNVRITSVKSSELSISWDAPDVGAG
GDNDLVERYEVRCYPRYDDATNATVIQTSELSATFKGLKPSTDYAIQVRAKTTRGWGEYT
PVIFKKTLHAMGLDYVGEDDNMQVRIIAGAIVAVVVLLVIIIIMTVLILRSRASDECNKK
QPSDCDTLEYRNGEGLVVTYMHCKMDSSPIVTTHTNNKSKSSLTTPLFTPAVGVAAAGAG
GAGGAGARSYVDPHTYEDPNQAVREFAREIDAGYITIEAIIGGGEFGDVCRGKLKLPPDG
RTEIDVAIKTLKPGSADKARNDFLTEASIMGQFEHPNVIFLQGVVTKSNPVMIITEFMEN
GSLDTFLRANDGKFQVLQLVGMLRGIASGMQYLAEMNYVHRDLAARNVLVNAALVCKIAD
FGLSREIESATEGAYTTRGGKIPVRWTAPEAIAFRKFTSASDVWSMGIVCWEVMSYGERP
YWNWSNQDVIKSIEKGYRLPAPMDCPEAIYQLMLDCWQKERTHRPTFANLTQTLDKLIRS
PDTLRKIAQNSVRPPAHNPYYVTSHTAAAQAAAAAMPPGPAANTASPFVDIVGHQAHQQA
QSAIAVPVMPPGAGRSLHYHTHAATHAMPHQQSSLTYPNWVPFSHFG
>XP_011157324.1 pep supercontig:Si_gnG:Si_gnG.scaffold02162:314000:419612:-1 gene:LOC105194212
transcript:XM_011159022.1 gene_biotype:protein_coding transcript_biotype:protein_coding description:ephrin type-B
receptor 1-B, transcript variant X3 [Source:RefSeq mRNA;Acc:XM_011159019.1]
MAIRMIPFNMAGVAGFLATCAAVAASAARLLPLLLLLIYPRGTPAEHVILLDTTTEEKLD
WTRYPFGPQANTPGWVEESFTNFDKGINWRSYVVCDVAYTNVNNWLWTPFIERGPANRMY
IEINFTTRDCSLFPGNALSCKETFSLLYYEFDAATKEPPPWEPDSYKLIGRIAPGEGRFN
TNTEVMINTEVKSIPVTKKGVYFAFRDQGACISILAIKVYYISCPEVSANFAHFPATPTG
REVALIEQSRGVCVANAVKIAQPTYLCKGDGKWYLASGGCHCKPGYQPDEAKQECKECPI
GKFKHEAGLTTCEPCPEHSKAPDYGFTECRCDPGYYRAEKDPKKMPCTQPPSAPQNLTVN
FVDQSTVILSWNAPHMQGGRSDTTYRVDCDSCSMGVKYIPNTEIFNDTKITITGLNAVTT
YRFQVFAENGVSSLVGKSEYVDITVTTEASVPSLVSNVRITSVKSSELSISWDAPDVGAG
GDNDLVERYEVRCYPRYDDATNATVIQTSELSATFKGLKPSTDYAIQVRAKTTRGWGEYT
PVIFKKTLHAMGLDYVGEDDNMQVRIIAGAIVAVVVLLVIIIIMTVLILRSRASDECNKK
QPSDCDTLEYRNGEGLVVTYMTTPLFTPAVGVAAAGAGGAGGAGARSYVDPHTYEDPNQA
VREFAREIDAGYITIEAIIGGGEFGDVCRGKLKLPPDGRTEIDVAIKTLKPGSADKARND
FLTEASIMGQFEHPNVIFLQGVVTKSNPVMIITEFMENGSLDTFLRANDGKFQVLQLVGM
LRGIASGMQYLAEMNYVHRDLAARNVLVNAALVCKIADFGLSREIESATEGAYTTRGGKI

PVRWTAPEAIAFRKFTSASDVWSMGIVCWEVMSYGERPYWNWSNQDVIKSIEKGYRLPAP
MDCPEAIYQLMLDCWQKERTHRPTFANLTQTLDKLIRSPDTLRKIAQNSVRPPAHNPYYV
TSHTAAAQAAAAAMPPGPAANTASPFVDIVGHQAHQQAQSAIAVPVMPPGAGRSLHYHTH
AATHAMPHQQSSLTYPNWVPFSHFG
>XP_020722019.1 pep chromosome_group:Bter_1.0:B14:3429:97745:1 gene:LOC100645846 transcript:XM_020866360.1 gene_biotype:protein_coding transcript_biotype:protein_coding
MAPFNMAGVAGLLATCAAAAASAAHLLPLLLLLICPRGTHAEQVVLLDTTQEEKLEWTKY
PFGAEANTPGWVEESFTNFDKGINWRSYVVCDVAYNNVNNWLWTPFIERGPANRMYIEIQ
FTTRDCSLFPGNALSCKETFSLLYYEFDVATKEPPPWETDSYKLIGRIAAGEGRFNTNTG
VVINTEVKSIPVTKKGVYFAFRDQGACISILAIKVYYISCPEISVNFAHFPATPTGREVA
LIEQTIGTCVDNAVVIEQPTFLCKGDGKWYLPNGGCHCKPGYQADVEKQACTECAIGKFK
HEAGSHSCEACPAHSKSSDYGFTECRCNAGYFRAEKDPKKMPCTQPPSAPQNLTVNFVDQ
STVILSWNAPHMLGGRTDTTYRVVCDACSMGVKYIPNTEVFNDTKITITGLNAVTTYRFQ
VFAENGVSALAGKSEYVDITVTTEASVPSLVSNVRITSVKSSELSISWDAPVTEVGGDSD
LVERYEVRCYPRYDDATNATVIQTSELSATFKGLKPSTDYAIQVRAKTTRGWGEYTPIVY
KKTPHAMGLDYVGEDDNMQVRIIAGAIVAVVVLLVIIIIMTVLILRSRASDECNKKQPSD
CDTLEYRNGEGLVVTYMHCKMDSSPIVTTHTNNKSKSSLTTPLFTPAVGVAAASAGGAGG
GGARSYVDPHTYEDPNQAVREFAREIDAGYITIEAIIGGGEFGDVCRGKLKLPPDGRTEI
DVAIKTLKPGSADKARNDFLTEASIMGQFEHPNVIFLQGVVTKSNPVMIITEFMENGSLD
TFLRANDGKFQVLQLVGMLRGIASGMQYLAEMNYVHRDLAARNVLVNAALVCKIADFGLS
REIESATEGAYTTRGGKIPVRWTAPEAIAFRKFTSASDVWSMGIVCWEVMSYGERPYWNW
SNQDVIKSIEKGYRLPAPMDCPEAIYQLMLDCWQKERTHRPTFANLTQTLDKLIRSPDTL
RKIAQNRGTNPLAPDAVDLTQLTSVSEWLASIKMSRYAESFERSGVTTLEAAARVTVQEL
TALGVTLVGHQKKIMNSVTALRAQMSATSQGFLV
>g1515.t1 pep supercontig:LinAna1.0:LFEI01000014:742572:802326:-1 gene:g1515 transcript:g1515.t1 gene_biotype:protein_coding transcript_biotype:protein_coding
MKMWSLEVFLAVLVTLSLRQYYSVEAIDFVVLLDTTIVDGALNYKTHRGGVSGYGWREGS
FNDGENVRRVYMACDIEHSEVSNWLRLPYIPRGDANRLYVEIEFTMRDCSTHRKPEEVFQ
CKETIALYYYEAMSDFATDTLPRWQAEQGSYQMVDSIAADYKFTNLSDYQINLKYRSVPV
SKNGVYFAFLDEGACTSLLSINVYYITCPAVRKNFAFFNTTATGRDVSSVVSKEGVCVDN
AVRVGNGPAPAYLCKSDGTWVWPTGQCYCKAGYQPNVDNTECLACPSGTYKATIGGDTCH
SCPANSNADGKTRATICDCNPGYYRLPHANASQPCIEPPEAPSNLEIQFKDQTTLRVAWM
APDGGNAAHITYHVQCLKCNSQRGQCVECDSTVRYVPIQTGIQQTTVTLHSLNPSTNYKI
NVYSMNGISIPVTDFNHATIIVKTMDAIASKVDNVRVVSKNQTAILVAWDRPVEQHEQYE
VKYFPTDNPFNALSVTTTSLRVIIGSLQASTQYSFQVRGKVFEGWGEWTLPVSVFTEAFI
TREVPTTTVSLAKDDKQTSPGFVNTDGIIAGAAVAVIVSLIIVAVMVVIYLKRNNQPCNE
KNKSDCDQLHYSNVQYPPPVIVDTHMNGSLTMPLFAPPGVREFGDVCKGKLRVPGKMELS
VAIKTLKPGASDKSRMDFLTEASIMGQFDDPNVIYLEGVVTKSNPIMIITEFMENGALDQ
FLRTNDGKFTIIQLVGMLRGIASGMRYLSEMGYVHRGGKIPVRWTAPEAISFRKFTSASD
VWSYGVVAWEVMSYGERPYWNWSNQDVIKAVDKGYRLPPPMDCPEATHVLMLDCWQKERN
QRPKFAQLVKTLDKLIRSPELLRKLAKSRPHNIFDPSVPDMTRFTSIEEWLCSIKMERYL
DNFLQAGYENMEQVSRITVTELAHLGITLVGHQKKIMNSIQTLRAQLSGAVQMSEGFLV
>g27553.t1 pep supercontig:LinAna1.0:LFEI01001124:80781:86738:-1 gene:g27553 transcript:g27553.t1 gene_biotype:protein_coding transcript_biotype:protein_coding
MTGALAYVEQIWDTNDNMDEWQILQVDSDSDESKQWQKVQVLSAFQTCTLESGQENWLVS
PPIPLPPELPSGALLPLRIRVSFLMHACDYTQYSLDECKNYIQLYAGYFPPGTNSINASS
LDAVNLIQDIQLRSPGAPDSVDVVYNASAARAQGSVTDQIRIALRDQGSCTQGLSVSVAY
FICMPIQGVLVSFPKSFPSTSPVQGSCISGAHPSNPPLQRVCSFDGSWSPQNGRCVCKPG
YIRTAAGCVETSPCESYPCFNGGTCHEENGGFLCECSVGFAGQTCENANGPCTRNPCLHG
QCSQSGVDGYSCYCQDGWMGTNCDQKDHCFRQECSGNGYCLNKQNTYSCQCQLGFTGLNC
QDRVCDLATCYNGGSCIPDSYAPDGYKCQCTEDFEGLQCLDRIQRCTYTVRVETSRAGRA
GTDERVVVTLGVQKFGELKKAQFEVQGDFEYGNVDEATKTLPLCGSLRQIEIHLRHDKTN
YLNINDWKLRQVAVIVDDNIIIKKYVCYFNTWFSPGDYKYRACSLL

>g4371.t1 pep supercontig:LinAna1.0:LFEI01000053:582971:595624:-1 gene:g4371 transcript:g4371.t1 gene_biotype:protein_coding transcript_biotype:protein_coding
MKTVNMAAVLKACLSSLLIFNCAGDEVLLMRSTDTFDNWTMLQLDGDDNSDPQTQWTKNN
VLKAFDVRNFAGNQNNWVVSPFISVDPLVTSVRVRMSFILYQCDYTIYTVDQCKQSFKLY
SKVSTSGQTIDAAFVNSFTERGQVDLNDPGNPDSKDTTFNITAGSAGFYVALKDEGAHIQ
GVTIEVHYFKCIALQNGLISFPASFPSDTVVTGQCAPNSSPVGSLERVCYSTGQWSSGTG
SCGCDPGFARNSSGNACYRLSPCDSAPCYNGGVCSNSADGSTFQCACSGIYSGPTCEIVQ
GSCTPDPCQNGGTCADLGGSYQCSCPSGYRGSECQNRGQSVRIVSSVPLLLATGGHSVRI
RTFVSTILVKMAAVVLTMEMGRDIFVPVLECTGEGIVKTSESRCAGADSCVIFCFLVAWF
GLFAINTVVWRGNIENGPGYLHDEMEVKKLNEKEGESAPQNSEKPKA
>tetur38g00020.1 pep supercontig:ASM23943v1:HE587338:27027:51937:1 gene:tetur38g00020 transcript:tetur38g00020.1 gene_biotype:protein_coding transcript_biotype:protein_coding
MQPTVKFCYCEPFWIILLFLIIHHFGVYICAQKTSVVLLDTTRESSLDWTRYPYGPQSKT
PGWVEESFTNFERGINWRSYVVCDVAYKNVNNWLWTPFIERHDANRIYIEIKFSMRDCNL
FPGMALSCKETFSLLYHEFDSAKEQDPPPWEPESYKLIDRIAADEGRFTSNSETIINTEV
RSIPVSKKGVYFAFRDQGACLSLMAIKIFYVACPNITTNFAHFSSTPTGKELTAIVPAEG
TCVPNAVEIEKPRLLCKGDGNWTLPSGGCECMPGYEPLGQSCQVCPPGTFKPSPGSELCG
PCPTHSTALYRGSTECRCDRGYYRASKDPKWFPCTQPPSAPQNLTIKDVDQSSVLLTWSP
PKYLGGRNDTLYRVECEQCPSTTTYLPSSESINETKVTIQGLNPSSTYRFIVYSENGVSG
SELSQFIDITVTTEPSGYISLVITEDDNGHKFNTNPIPGITNIRIVNVKSSEIKLSWNPL
GDGLMDDLERYEVKYYPKGAERNISTAFTTKEEIVLKNLQDKTDYAFQIRGKLISGWTEF
SKPVYQRTGQLVGSTAYIGEDDSVQVRIIAGVTIAIVVLVVVIGVMIVLYLRRNTDECNK
KQPSDCDTLEYTRNGDVPSLEHPPIVPTARFNSPFSVTTPLFTHCGTSRAYVDPHTYEDP
HQAIREFTREIDASHITIEAIIGGGEFGDVCRGKLKIPGRPEVTVAIKTLKPGAADKARM
DFLTEASIMGQFDHPNVIYLQGVVTKSNPIMIITEYMDNGSLDAFLRANEAKFQIIQLVA
MLRGIAAGMQYLSEINYVHRDLAARNVLVNAALVCKIADFGLSREIESANEGAYTTRGGK
IPVRWTAPEAIAFRKFTSSSDVWSFGIVCWEVLSYGERPYWNWSNQDVIKGIEKGYRLPP
PMDCPEALHQLMLDCWQKDRSTRPTFSQIVKTLDKLMRCPESLQKIAPTRYPPLDPFGDP
VIPDVVQFKTIDEWLSSIKLTRYRPNFEAAGLTNITSVFHLMPQDLSLLGITVVSHQKKI
LTSLQNLRAQTSIGTPEGFLV

Ephs in Cnidarians

>Pphy_15204 P. physalis
VVYQDEPNAIILEYMVNGSLDKFLQMNEMQFTIDELLEMARGVASGMKCLSEMGFVHRDL
AARNILVNGERECKIADFGMSLEIKIDDSTETKCGKVPVRWTAPEAIQFKKFTTASDVWS
YGVVLWEIMSYGERPYWDWSNLDVLEKVTSGYRLPPPMGCPRVVHDLMLHCWNKDRAKRP
LFSTIRNKIFSWLRDPSLLEDIAQSINLDPNLDYTVMQTVNKWLETIGMGRYAQNFIEQG
FATPRQLLLLTMSDIEALGVGPIEHQKKIYKAIENTRAQVESQIKNNN
>Pphy_15205 P. physalis
VVYQDEPNAIILEYMVNGSLDKFLQMNEMQFTIDELLEMARGVASGMKCLSEMGFVHRDL
AARNILVNGERECKIADFGMSLEIKIDDSTETKCGKVPVRWTAPEAIQFKKFTTASDVWS
YGVVLWEIMSYGERPYWDWSNLDVLEKVTSGYRLPPPMGCPRVVHDLMLHCWNKDRAKRP
LFSTIRNKIFSWLRDPSLLEDIAQSINLDPNLDYTVMQTVNKWLETIGMGRYAQNFIEQG
FATPRQLLQCIHGTGLNVEHIDVNVNGLSSILHDDWFLPCHCCF*
>Aele_129846 A. elegans
MPVKEILGNQIRELVYIFVLLLATSNASKEIIKQDSGSIDQTSNKWEREDQNDCGLRWKE
KREDGTVYYDTCKSVTTGNKPCNLTTENWVIPAEVNSVFIEVGGTWHNCTQCTPHFDLLV
KFPAKSSSYVQVMKLPQKTALKKDKFFSYDDSIEVPVNGYRNMTLKFRTTYFCGDISKVR
LYYLRCPSRSKELVTFSSKPAPTGSNNLTIQGYCTAGSDPDPSNQAREPYMICRPSGDPI
IHGKCLCKAGHFPKDNECKECGNNEFKSTIGNVGCDKCGKSSTSEKFGKTKCECKNSHYR
ETGKEENYNETCYRLPDAPSDLTIKDVTNEKANISWSKPAHGSAPLGKYFIDCLNCPADE
KTFPVNTTDTSIHLSGLKPAASYKLQIYNKNSITGLVKNEGFWNKEIFYLNTTSGGKPGR

IQQLKHVVNENSTVTLTWNPPFYKGGDDLVYEIHYGKQTHKTKDTKWTIETGTEDKIYIV
KIYATAVVKGIRVKGDVVNSQEITVKGGIPLPLTIAVAASVIVLVILVLVASFCVWKKRH
PSYLQVVRMENGAVQLPHKFFSHAKLYVDPTTYNAVDEAVQEFAKEIDRKDLTVGEQLGG
GEFAEVFKGLLYRNGAYKDVAIKTLKSGVVSKRDRDDFFGEAAILGQFSDCTNVVKLEGV
VLKDRPNWIILEYMKNGALDSYLQKNDNKFDVTQLLGMARCVANGMTYLSEIGFIHRDLA
ARNILVDEFNVCKVSDFGMSREIKLDDTYDTQGGKIPVRWTAPEAIQYKKFTSASDIWSY
GILLWEIMSFGERPYWDWGNYTVLERLNAGYRLPPPMNCPKVIHDLMLKCWHKDRTKRPK
FKEIGDQIEQWIRTPLLLRGVAATVVRKRDENLDYTVLETINKWLEAIGMDRYTETFLTN
GYSTPRQVLNLTMDDLEKLEIGPIGHKKKIYKAIQNTKEQVESRHNSSSEKTAKKYNLPN
FKK*
>Aele_22617 A. elegans
LVTVYKIRAVDHYGSPILKCISYIKGDISMKIYWFVMIYVCCESKVIQLYEESQRNEWRP
KGECIGSWGWLTSSKTWETVVKTGYKQNCSLYTNYWPINVNLNMVFVNITGNGNSIPQCV
DHYTVKVMQGGKNSTNYQKIANIPPSKKTRGFFNYTETLSINVFATTLGLKFAFQSTGFC
GTISQFQVFYYACPTNMELAKFKSHLAPSQTDSPIEISGECVAHSVPSVKTLKPLMKCYY
NGRSEIQGGCSCDKGFEKTEKNECEGSSANLITINLDCIHHF*
>Aele_55285 A. elegans
QPCGKDAVSNGGVACKCKDNYFRLIGKENVDVADCYRPPGEPQNIVKRNITEDSVIIEFQ
QPDIIGDAPLGVYVLNCTACGKGGHFPMYSKTSPFNVTGLGALATYQLLIYNRNRITNQT
GHFNPFMFNITTLVGGKPGVIQDFQVEPKGNGSVVLRWKAPFIKGGRELEYVVKYGPTTQ
TTKYTYLILKSKSTTQKYQAEIYAQVTINGKTIKGEVYRSEIIEVKAYGISLYTTIGVVV
GIAVLIIVISLIFFFVWRRRYPRYVQRMEDGTVITPSRFLPGSKLYIDPLTYGDVDDAVE
EFANEIDRKKLTMNRLIGGGEFAEVYTGTLVKPSGKVTNVAIKTLKTGSSKKDRDDFLSE
AAILGQFTDTNVISLEGVVMKDRPHLIVLEYMANGSLDQYLQKNDLQFQVVELLGMARCV
ASGMAYLSDKGFIHRDLAARNILVDEHNICKISDFGMSRELKLDDTYETQGGKIPVRWTA
PEAIQFKKFTSASDCWSYGVLLWEIMSYGERPYWDWGNYTVLERLNAGYRLPPPMNCRKV
IHNLMLECWNKDRTKRPRFKDIRERTEQWIRSPELLGEIASVVTKTDENLDYTVLETIHK
WLEAIGMEQYSELFVNNNYSTPKQILDVVDNDLIRIGIAPIGHRKKILKAIKNTRNQVDA
RHRGGSVVNDSFRKKTFSLSRKK*
>Nbij_1526 N. bijuga
HRDLAARNILVDEFNICKVSDFGMSREIKVDDTYDTQGGKIPVRWTAPESIQYKKWTSAS
DIWSYGILLWEIMSFGERPYWDWANIKVVERVNSEYRLPPPMNCPKVIHDLMLKCWHKDR
TKRPKFKEIGDQIEQWIRTPVLLRGVAQTTVRKRDEDLDYTVLETIAKWLEAIGMGRYTQ
TFLNQGYSTPRQVLSLTMDDLEKLDIGPIGHKKKIYKAIMNTKEQVDSCSAAGGNKYT*
>Nbij_150435 N. bijuga
LKYIIKYGEQSHTTMETTWAISTGTEDRTYKITVYASAFVNGKREDGTAFTYAQDIIVKG
KGIPLVLTIVVAASVIVFVIIVLLSAFCIWKKRNPAYLQVVRMENGAVQLPNKFFSHGKL
YVDPTTYNAVDEAVQEFAKEIDRKDLEVGELLGGGEFAEVFKGTLYRNGAYKEVAIKTLK
SGGMVTKRDRDDFFGEAAILGQFSDCSNVVKLEGVILKDRPNLIILEYMKNGALSSYLQE
NDGKFDVTQLLGMARCVANGMTYLSGIGFIHRDLAA
>Nbij_35443 N. bijuga
GIVLWEIMSFGERPYWDWSNLDVLEKVTSGYRLPPPMGCPRVVHDLMLTCWNRERAKRPL
FSSIRNTLIAWLRDPKSLADIAQSIQLDPNLDYTIMQTINKWLETIGMGRYTQNFLEQGF
ATPRQILLLTMADIEALGVGTLEHQGLQCQPDFAFFLL
>Clat_76838 C. lathetica
MKIYVLLLVVGCAGDQIQLHQKPAAFKGLDAWTKQISCNPSWLKKREPLGLIWETCVGTD
KYIDNNNCNLYSEYWLFGRSDVNAVFINLYGGGVNCSHHKYHCTSKLGLKIYVGSDGGAD
SDNKYKNISLIPNDPKPGTNLHFYDYNETFQVNVYGNSAVKLRLHTTYFCGQLKGANIYY
YTCPSVSKELVKFPPQNAPGVQDSPSKIVGVCVDNGIKITDELYMNCYYDGNSTIHGKCE
CNKGYEIHGDFCKECDGNKFKPNVGNEKCKACGKNSQAQGRISCTCIDNYFRETGKENDD
EADCYKPPSAPTIRAIRNITEHSAIVEFNEMTEDDIGDGSIGKYILNCTKNCPQKGNFPM
ISKTSFNITGLGSYVTCHFTIYYSNNITKLTSHRNFNQFNFTTLAGGIPGKITGFKVIYN
SDGSITLTWKGPEVRGGRKLEYGVEYGDTKVVIKYTNITIKSGSSTNKYVFKIYAQYMDG

KKLVKGIIFTSEEITIKGGIPLLTTIIVVVAIIIVIILIAITSFCIYRRRNPPLRERRED
GTVIQPNRFLPGSKVYVDPTIYKDVDDAVGQFAYELNRSQLTFGGLIGGGEFAEVYTGTL
LRASGKLTNVAIKTLKSGAGKKDRDDFLSEAAILGQFTDANVITIEGVVMKDRPHLIVLE
YMANGSLDTYLQKNDMQFQVIELLGMARCVASGMAYLSDMGFIHRDLAARNILVDEHNIC
KVTDFGMSRELKLDDTYETQGGKIPVRWTAPEAIQYKKFTSASDCWSYGILLWEIMSYGE
RPYWDWNNYTVLERLNDGYRLPPPMNCRKVIHNLMLDCWNKERAKRPKFKEMREKIEQWI
RSPELLAEIASVVTKTDENLDYTILETVQKWLEAIGMEQYTEMFLNNQLTTPKQLLDIGD
NDLLGIGINPIGHRKKILKAIKNTRDQVDARHRGMSMNGTLRKKTFSLSRSKK*
>Clat_102964 C. lathetica
SGSTDQRSLKWERYTVKSCGTSRWSQTVSNGIRSYSTCTARNPSSTPCSLITEDWELPDE
INTVYLNIQGDMNNCSNIVSVAGYDKCTSYYVLKAYIGNSINADSYKNISNIPLRTTGFV
PGTKLKRYNDTILIQVTGYKHIRLKFETTYWCGVINGVKMYYYSCPTTSAELVHFNQRPA
PSGSDAFEVYGRCTSNADLGGFGMPKMTCYSNGSATIHGKCICREGYHRNNHQCDPCKKD
EYKSEPGNSDCKKCGMNSQSGKTVGVKCICNELYFRKRGSEKNFSRDCYRPPDAPSKITI
SNITHDYVQISWTAPADSGSSPLGEYHIICMNCLVEAEKVFPVYTNDTSINIFGLKSVAN
YELKIYNFNYVADIIDSNNWKVTAFNFTTAAGGKPGKILGLHDIKNDDGSITLKWNPPFN
KGGTNLKYVVKYGDQSGSTKETTFIIPTGTEDKVYKVQIYAQATINGATVDGELFTYYKE
IKVKGGIPLTLTVAVAAGVVVAVIVFLLSAFCIWKKRHPPYLQVVRMENGAVQFPSKIFS
HTKVYVDPTTYNAVDDAVQDFTTEINRKELNIGELLGGGEFAEVFKGTLYRNGLYRDVAI
KTLKSGAGKRDRDDFFSEAAILGQFSDSPNVVKLEGVILKERPNLIILEYMSNGALDSYL
QKNDNQFDVTQLLGMGRDVAKGMTYLSEFGFIHRDLAARNILVDEFNVCKVSDFGMSRAI
NLDDTYDTQGGKIPVRWTAPEAIQYKKFTSASDIWSYGILLWEILSFGERPYWDWGNYTV
MERLNTGYRLPPPMNCPKVIHDLMLKSWHKDRTKRPKFKEIVMQIEQWISTPLLLREVAS
VVRKRDENLDYTVLETINKWLEAIGMQRYTETFLNGGYSTPRQILSLNMEDLEKLEI
>Clat_108627 C. lathetica
MINISTSTNINALKNLTFNLKKDSELSSIEPIPGVQNKAETFAYQNAYIFDNMSTATTVQ
LEFVSLGYCGKVIDVTMYYYRCAGESSKSLASYKSTPAPSKRKIVSKIPGNCVKNSRLDS
SYEPYLKCYYNDTMQFFGHCICNPGYTIAETSCKQCNTDEYKPQEGNQACFKCGRNSKSD
DGIKCLCNGDFYRAIGKESNFSSECYEPPSVPRDVRLTDVTSYKATLNWIKPEKAGSSQL
GLYYILCTNCPTKMGINITTSGVNYTLNNLGAFASYYFDITNENNITSLTGKRHTAKIEF
KTKPGAPGKIHDFVQFSRSDGTVKISWKPPFAKGANILWYVIKYGEHHLEQKELYFVIEP
SSQDETYNVEIFTKVVIDGKTLNGATYTEKVTVNGGVPLTQYLLITFSVLVFVIIVLIAS
FCVWKKRNPAYLQVVRMEDGSVKLPTRFYTGGSLYVDPKSYADVDDAINDFAYEIDRSDL
TTGDMLGSGEFADVYKGTLARGGKTHSVAVKMLKADASKADRDDFLSEAVILGQFNDANV
IRLEGVVYQDEPNAIVLEYMINGSLDKFLQEHDMQFSVDELLEMARGVASGMKCLSEMGF
VHRDLAARNILVNAERQCKIADFGMSLEIKIDDSTEEKVGKIPVRWTAPEAIQFKKFTTA
SDVWSYGVVLWEIMSYGERPYWDWSNLDVLEKVSSGYRLPPPMSCPRVVHDLMLDCWKKE
RAKRPLFSVIRSTIFGWLKSPDSLEDIAPSSIQLDPNLDYTIMQTINKWLEAIGMGRYTQ
NFLEQGFATPRQILLLTMDDIEALGIGPSEHQKKIYKAIEMTRSQVEAQIKTKSTDGKGN
FFAFMYGSRSRECEVVMLTEEDTT*
>Atet_45096 A. tetragona
PPYLQVVRMENGAVQFPHKLFNNAKVYVDPTTYNAVEDAVQDFTTEIKREDLNIGELLGG
GEFAEVFKGTLYRNGFHKDVAIKTLKGGAAKRDRDDFFSEAAILGQFCDCPNVVKLEGVI
LKERPNLIILEYMSNGALDSYLQKNDNQFDVTQLLGMGRDVAKGMTYLSEYGFIHRDLAA
RNILVDEFNVCKVSDFGMSRAIDLDDTYDTQGGKIPVRWTAPEAIQYKKFTSASDIWSYG
ILLWEILSFGERPYWDWGNYTVMERLNTGYRLPPPMNCPKVIHDLMLKCWAKERTKRPKF
KEIVEQIEQWISTPLLLTEVASVVRKRDENLDYTVLETINKWLEAIGMQRYTETFLHNGY
STPRQILSLNLEDLEKLEIGPIGHKMKIYKAIQNTKLQVDARHNSMNGKTKKAPKQLKT*
>Hvul_13820 H. vulgaris
MTINCYMLINLVFSFCCVTNYNGEIVYLINDYSDSKLPSKWINIPKYLKYNCVDKWNLDY
FEYCFVGNSLYPENYCVLESSIQNLSNVSNVFINITIKTKTCQYRKIKELNCSNFLNLSI
SNGENEIMQVSLPGITVPVEYQDQYWLSNDLIVSDVINKSTIQLKFISHNYCGVLLNVSV
FYYKCPNKTIQLVNFPNSPAPSLNNSPFKIKGVCSKNAVTYGTINPYMNCYSNGSYILFG

ECWCKEGYEKNDLDCEACKDGYFKNKVGNNICEKCSLNSKSVLGTSCICNEGFYRFQGNE
NNSAAICYKAPSRVQNVTVHNITVQSATLSWMIPTDHFVDYFYINCTNCPSQNFTLRHTL
QTCIFIDGLDSYTKYNVVIESRNNVSALIGKVISTYVQFKTKIKAPGSVQDVVTNVNSDR
SVTIIWKSPHWKGDKKLKYKVKYNGNEFIISETHYTISSSSTDKSFTVEIAGYFIDDDGN
QLNGFVYKKLIFLKGQSKLSAVFGTVGGVIGLIAIVMVVVLIVWRKKHPLYLQAVRMEDG
TVRIEGNHFLNRGRLYIDPNTYDCVDDAVQEFANELDNKNLEVGNFLGGGEFADVYKGTL
LKNEKRIDVAIKMLKHGASKHDRDDFLGEAAILGQFTDYNVILLQGV
>Hvul_13823 H. vulgaris
KLIMKNIFWLYKYIAWNICTAVNTEKYYITDTDDVSILSKQWAFSKFNSSYTCAGAWQWV
DKTREWKTCLKASTTMLQNCILISTTYNVTGTNTVFIEINFSTTLCQSLKSPSCNEYLTF
SAYIGTNVSEVILNNTIQSNNLTDVSNKFNTFYDTKGIENMKLAFTTSDYCGILKNVSLY
AYWCPSTIKELAYFPKVAAPSIKNSPQKVQGTCIENSISNVAPYIECYFNGSYKTNVECK
CKKGFEFKENSCQVCGPNYFKNDDANVPCQACGANSVFNLITSCVCLTGYYRIFGEVNNS
AAACYKPPSPPKNITIQNITEQSAFIEWVLPEDSPFQGYFYLECLNCSIQDNFPKNTTQN
QIILDKLDSFTEYNLTISYINNISQLTGKYEKYAFSFQTKFSAPGLIRDITIKYNSDGSI
ILTWMPPLKNNGGNISYKVKKNDVVFVTSNTYFEINSALFDKTYIIEIMCFVGTSNDIQI
AGPIFKETITVKGTLKLSALIGGICGAFAIILIILLIFFIIWKKFHQIPTEVVRLEDVKS
NYHVINGEKIYVDPTTYNKVEDAVQKFAIEIDRKNIKSGKLLGSGEFADVYKGTLIKDGK
NITVAIKRLKSNASKNDRDDFFGEAAILGQFNDQNVVHLYGVLLKDSPNEIVLEFMVNGA
LDKFLQTNDMQFTTLQLLGMARGVASGMKYLSEMKFIHRDLAARNILVNEDYCCKVADFG
MSRKINIDDTYETKGGKIPVRWTAPECVQYKKFTSASDMWSYGVLLWEIVSYGERPYWDW
GNYLVLERINSGYRLPPPMGCPKIIHNLMLRCWEKDHLMRPKFSEVVSLLENWIKNPELL
KEEDSSTVNKSDGSLDYSTLKTVNQWLEAIGMEKYDQNFLEKDFFTLKQVIMSIIIEEDL
LDLGIRPVGHRKKIFKSIETIRNQIEKNV*
>Hvul_18252 H. vulgaris
IECLEGYYKTDSGNHPCSKCGANVKPGAAPRILCECLPNYHRQIGRDSNEADCFESPAQV
ENIKVLNITSDQATITWDEPRKGSFELGFYVIQCNNCPSYRNEFPKKTTDLSITVTNLAA
YATYKLCIYNENNATNLTGIRYYALFSFLTEKGVPGKITDVTNTSDSDGNVILKWNPPFA
LGSDQLTYVIEYGDKTERTKNEFIFIKQNLFDKTYRVRIGTEVIIAGKVLSGPDYTFEIK
VPGGVSPVTTLGIAAGIIVFVVVVLVASFCIWKKRNPAYLQVVRMEDGTVKLPARFYSGG
KLYVDPKTFLLVSDAVSQFAYEIERSEIVFGELIGSGEFADVHKGVLMKEGKKEPVAIKV
LKNGASESDREDFLSEAAILGQFSNPNVIFLEGVVLKDEPNLIVLEYMEYGALDSYL
>Hvul_31223 H. vulgaris
LPSRLFKNQAKAYIDPTTYKDVDDALEEFANELDRKDLRVGDILGEGEFADVHKGILLRD
GKLIDVAIKKLKTSASKHDRDNFLGEAAILGQFTNQNVICLEGVVLRDKVNMVVLEYMVN
GALDKFLQKNNNQFTILELLGMARGVASGMKYLSEMGFIHRDLAARNILVDELKNCKVAD
FRINQKIAIDETYDTK
>Hvul_37681 H. vulgaris
MECFSNGDYQVYGKCICKEGFEPKDFLCRECSSESFKQNVGNTPCQKCGLNSNSYNPWTS
CICXLGYFRAIRSEKNYSETCYKPPSVPEDINVFNITSQSAYLKWRHPKDGSAVNGYYFI
ECVTCSDQDTFPKNTTKTEIFLHGLGSFGDYSLKISYRNFVFSLTNKFSSEVVNFKTLTG
KPGEVQNFQKKLNQDSSVTISWKPPFSKGGPNLKYSVKYDGKEIITFQNSTNIKSGAEDR
TYKVEIRAITSVDGKELYGPALMVDVQVKGGVKLGAALGAAFGVLFFIVLLFILGLYIWK
KRHPMYFQAVRMEDGTVKLPSGPFKSQGKVYVDPTTYKDVDDAVEEFAYELERKDLKFGT
LLGGGEFAEVYKGTLLRDGKPIDVAIKILKNGASKHDRDDFLGEAAILGQFADQNVICLE
GVILRDKPNLIVLEYMANGALDKFLQENDNQFTTLQLLGMARGVASGMKYLSEMGFIHRD
LAARNILVDELKNCKVADFGMSRKINIDETYDTKGGKIPVRWTAPEAVQFKKFTSASDMW
SYGVLLWEIMSYGERPYWEWGNYEVLERINSGYRLPPPMGCPKVIHNLMLNCWNKDRSKR
PKFSSIVILLEKWIRNSDLLTEIAPSVVTKADENLDYSVLLTITKWLEAIGMEKYATNFL
DKGYATPRQVIGLTIDDLEELGIGPIGHRKKIFKIIQSTKAQIEGGFNKNRHASVSSKLK
LVPSLKK*
>Hvul_37726 H. vulgaris
LEFMANGSLDKFLQKNDMQFSILQLLGMXRGXASGMKCLSEMGFIHRDLAARNILVDESF

CCKVADFGMSXKXXXDDTYXTKGGKXPVRWTAXESIQFKKFTSASDMWSYGVLLWEIMSY
GERPYWDWGNYEVVERVNTGYRLPPPMGCPKVVHNLMLECWNKDRCMRPRFSGVVSMLES
WIRAPNLLLEKAASVVQKNDEKLDYTILQSITKWLEAIGMEKYANNFLEKGFATPRQILN
VSFEDLLKLGIEPIGHRKKIYKAIQNTKVQVNFFFTVFM*
>Hvul_37741 H. vulgaris
LGSFLSQLYLKKFIHILLQCLHGRTWPGKEFILKALSTLTIKCRKEMIDSISVNEILNKM
IKECEKDNLSYKLSAVDCFTSVANEWQTDVFEEFKPVLKTLFEQKFLENEDNQVEKQDLL
LCAGCESLGRLWPYQSTSQRIHFNYTASLFTNLLNNSTWKTQKALLIASTSFVERTIWSE
ACHWYNEDETNIVTAFLNIFVTEVFKCMKSTAYSSVKLQTLKFFNELYSSAKSCNELYNV
IKPFEDELISITILQLLGMARGVASGMKCLSEMGFIHRDLAARNILVDESFCCKVADFGM
SRKINVDDTYDTKGGKIPVRWTAPESIQFKKFTSASDMWSYGVLLWEIMSYGERPYWDWG
NYEVVERVNTGYRLPPPMGCPKVXHNLMLECWNKDRCMRPRFSGVVSMLESWIGTPNLLS
EKAASVVQKNDEKLDYTILQSITKWLEAIGMEKYANNFLEKGFATPRQILNVSFEDLLKL
GIEPIGHRKKIYKAIQNTKVQVNFFFTVFM*
>Hvir_14528 H. viridissima
MYFQVVRMEDGTVKVPSGPFKTQGKLYVDPTTYKDVDDAVEEFAYELDRKDLKFGSLLGG
GEFAEVFKGTLIRDGKPIDVAIKK
>Pper_114993 P. periphylla
MDDIIIAPAVLNPRRNLSLFVLVVVLIAVLGSVVTDAMATKKILSTGISQHVNFEGPGSS
WTVGGTTPNLIVETCSGFVSGTAPNKYVITPYISTDPAKQVKVEIGYQLKRCVSSPLLTP
ASHCKETFGMYVYRSTDTSGRSIASIFVHEATLR*
>Pper_114995 P. periphylla
MARIGRISMGIISSGARALRKLKLLVLVMVSIAVFGSVVTNCTMAVKKILSTGISQHVNF
EGPGSSWTVGGTTPNLIVETCSGFVSGTAPNKYVITPYISTDPAKQVKVEIGYQLKRCVS
SRLQPASHCKERFRMYVHRTIDVSIRTTLSEYVHEATLTNTSVFDPSINYNPFSREVWVN
MKNKDHLRIAFRDQGICGVINKLIISYLVCPTQSENLITFEETPAPASVNEPVIISGSCT
SNSNPDPRKPLAMICFANGTSLTSGTCLCNKGYEKNKTTCKACSRNFFKDSTGNRPCSPC
GKYTKIPNVPRIDCKCWDGYYRPKRRLADWTVNCEKLPGRPRNVNVTKITSASAVLTWLG
SNNQGTGGKVQYKIICNSCKDKKVYSTNETGLNFTLSPLGSYADYNVSVIAVNNVTQSIG
KWNEGCIVFKTKTGVPGNIRNLKITRNNDGSVTLTWDAPFATGGPDIQYVVKYG
>Pper_114987 P. periphylla
MARIGRISMGIISSGARALRKLKLLVLVMVSIAVFGSVVTNCTMAVKKILSTGISQHVNF
EGPGSSWTVGGTTPNLIVETCSGFVSGTAPNKYVITPYISTDPAKQVKVEIGYQLKRCVS
SPLLTPASHCKETFGMYVYRSTDTSGRSIASIFVHEATLR*
>Pper_2496 P. periphylla
AFRPGQYVVKYGSNTITTKGTDVNIKQPDKETTYTFSVSARTSAGSSAPVKSPAVTMHGS
KKGVSSTIAIAGGVSLFVLLLVVTAFAIYFWKKRHPKHLQAVRYATGEVKLPQKFSGGAK
VYIDPTTYQDLTEAVREFAKKKK
>Aaur_11068 A. aurita
MPFAEIPYDDLEFFECCGGGTFGSVYRARWKSQDKVKEVAVKRLLTLDKEADVLSVLSHK
NVIQFYGAVSKQPNYCLVTEYAALGSLYEYLSNDVLDFKSILQWAKEIASGINYLHNEAP
FKIIHRDLKSKNVVITSDLNVKLCDFGSSRFMSQTTKMSMAGTFPWMAPEVIQSMPVSET
CDTFSYGVLMWELLTNEVPFKGMQGVQVAWIVVVKEERLTVPSTCPPMFASLLRKCWLTD
PRERPDFKEIRSLLDCMCEDPELEDETNSFIQHKNEWRKEIESTMEKLKNIERNLTLKQR
DLHQRELRLLLKEKQKSSKAINKADLKDWTEDDVHAWMQQMGNEAVDLYQYADVLKENHI
NGRRLLMLTIDDLKEIGILSYGHRMDLFDLIQRLRDEMEHLAHFPPLQVVGNQELYCSDC
PVITLTLLFGNHCRLGATPMDHKWKMFLEVDADDSALTCIKDVTFHLPTSLEYYTVTHPP
YVVDRWNAAGHDNSPIYVECNVSYEKNVKKPRNTKHLHEVLLKERK
>Aaur_11280 A. aurita
SVSIATPKMGITALMLAADCGNESIAYFLVQQGASVNIRDKMGQTALFHAVEGGHINVVK
LLLDCDADIEARQSQTLLTPLMVAAIEGHAVIFSMLLEYGADLKAKSHLGETAVTLANKF
GNITIVNVIENMCQNVKKVQLRGEAGLFENDLGVRDGPEKIARLAKEAREASMQKAKKTN
VMRGRDMVSPIDARDYEPVEGSWVRPEFGALEKELKDMALSEKDKSLSQSAPNVEALKGD

ISQTIVIPARKTKVSRICYEAPKQPILPPSQDQDGVEQSSIADILDSLKLTKYYPCFEKE
DVDMDTFLTLTDEDLKHIGIDKLGPRKKLISFITKHCKSRTVDNKDDDASAGKDAEGAIQ
QAHAYVEYLQAQLYKEKQGRHELESYFVAERGRLDQIQKSAKQMNKTLNETASLLDTLRT
TCNTTAEQLTSMQRDLSSDQNTVAENVKEAASFHQQSLNIINKITSQFSFVVRTSQFLNG
LCTDEGAGES*
>Aaur_12598 A. aurita
MADDESSSAAFRLVEDWLRSMGLICYAQSFYDNGYENIDICKEIGEEDLNVLGVTNIKDR
NDILAGVCRIKQKAVYFELESDEDVPSEPIIRKLDPFLLITRVKAELEKENVQLTEPPYF
YPDGSLGSLDSLAEQLSFKFETYHEDVYNALDQLRRRQLPTGLYNDVNELMEMQKDQEKK
KRFGSKAIGPPKPKREDREEPKAYLNVETPFRRTTSMSLSKHDKRITKSTTNLADVELDN
QPLEVSHKKKHKKGKSVSKSFSTREKSKALFGLSIRKRSPTSGDKDKINKLNNRESFVAK
DLTMNQKELTELMLKVKHGEISQDKVLDQVNLNYRQSVMMDSNFDHVKSLPKHQLTKKEK
KQLR
>Hdig_11359 H. digitata
MLRSCVVSLVLIEALLVLTAADNITILTVPKAGSIWSWKASGTSPIPWSQKSSPARYAVC
DKDQINHWLRSDVFQVKDAKRVDVTVKYAVSNCSGLTGYINCKVNLDLHVYKTSTKHG
>Hdig_16446 H. digitata
NDTSQELIMSVAIGVAALVVIVIIIILAVYLYKRRHNRAESKQHLAMTPDLIPLDGHRMY
IDPSTYGSPMEALMSNSEEVDFHSIKLDKYIGGGEFADVYKAILTKGLETEIVAVKRLKP
KSSTKNKEDFILEASIMGQFTHPNVIELKGVVTRSPDSPMMIVTEFMESGSLDHYLKLRD
GQLTVLQLVGVARGVADGMSYLSKMNFIHRDIAARNILVADNMASKVSDFGLSRELDDSE
DNPNSEYQTQGGKIPVRWTAPEAIRYRKFSSASDVWSYGILLWEIMSFAERPYWEWDNFE
VMQRVDSGYRLPAPMNCPKAIHNVMLDCWEANKINRPKFPEILQRLEDMIRSPETMSDDQ
VSSRFSKVGSQANFGEVSSVEEWLVQINMRQYTDNFVNAGLTELRQVTTVNEDDLKRIGV
KLVGHRNKINKSIDAMKTHFESMNNGNEIRKA*
>Apal_25755 A. pallida
SHAAYNHEGLTICPVMNWEFLSLVSVLILVSSTFKTWSEAKNETIIKVPYKNEQSVWTWS
WTNQWTTRNDEDGKALFYVCDKTPHGDPNKWIRSNKFLVGDAKRIDVTVKYGVIKCSSLP
LIQQCKEKLKLHVFKTAEHHTLTTNKVPEPWKNPEVYHIFGTLSPANLSSGGFLRSFTNT
ETHSFFTNSSTPYHYFAVQYQGGCFELYSVTISYSVCPSLFLPILLPRTVAPVNGAQVQV
QGSCSKNAEPADKNVPIYGHCQANGEWSGTAMSGKCLCKSGYGKYLNGSDAVCKDCPHGT
FQDTPSESACKACPLNSVQDLNRMRCNCASSFYRTKDQTIFDNCDAQPSAPRDFRATFLN
ATTAIIQWKTPSNLGGRKDMYYELQCQKCDNEGKNCSLKCATAKITHSPRHSSKATVKHL
SAYAYYQFKVFAKNGVTHLAEKNGEKPKYAVVTKRTGESVPGSPVLKIVFMSPTSVLLSW
TLTEKNGEITYYQVIYYPVKDKSSRYIFNTSENSLTINGLDKGTEYHFEVRAATKLGVGS
VGSTQIAITKGKGGDTNIPTNGATNTIVAVLASIVVVGLLTIVVFILRKRRVLHPSEETV
RYVPGNRAHNTDISPADPSEEFTDQVAINDPEETSIPRNESNVPYHIPDLDVSEESTEQA
AVPANHFEETTNPTNE
>Apal_103658 A. pallida
PTRRSSDLKNFAKELDTCCLTLEKVIGGGEFGDVYKGVLKRTGEDPLPVAIKTLKKESTK
KNRDDFLLEASVMGQFCDPNVIFLEGVVTKSNPLMIVTEFMSNGSLDNFLKEMDSKLSVL
QMLGMARGVASGMKFLSEMNFVHRDLAARNILVSETLVCKVADFGLSRELEDSAYETRGG
KIPVRWTAPEAIKFRKFSTSSDVWSYGILLWEIMSFAERPYWDWSNFEVMDRVEGGFRLP
PPMQCPTAVHQVMLDCWKADRTQRPKFAAIKNHLDMLIRSPEKLKENIPIQPSQPSIDYT
DVTTIGDWLKSIKMAQYTDRFVQAGYSNLKNLAEFEEPDLDEMGIKLIGHKNKIRKSIRD
VRKKLHRESSVPV*
>Apal_612045 A. pallida
HLYPTIEMSYHLLGFLFLTIWIGQCSVAGDNIHVLNVPNNNKQWNWTTFGSDMMRITNAN
DETTEYQACQMYDAENQPNSWLITDAIKIDKVRRIDVTVEFSIWNCSGKEMRDVKYCKES
FDIYVHLAS
>Apal_215216 A. pallida
MKCTFLSSFIFVSIFMACSEAEKETIIEVPNKNSQGNWIWSWTSQWTKKDGGGKVFYQVC
DVSSDNEPNKWIRSNVFRVGDAKRIDVTVQYAVSKCSSVPSLQYCKETLNLYVYKTAIQH

TISNLVPEPWKKPNFYHIFGT
>Apal_215219   A. pallida
MKCALPSALSSLILASVFIAWCQAETETIIEVPYKNSQGKWIWSWNSQTSKKDGDGKVFY
QVCDVVSPSEPNKWIRSDVFLVGDATRIDVTVEYAVFKCSSVPSVQYCKETLNLYIKKTA
IKHSPLYKLQGPLRRPKDYRIFGTTFPTNLSTGILSRRFTNTETYSFFTNSPTPYHYFAI
QYQGGCFELYSVTISYSVCPSLALP
>Apal_215220   A. pallida
MKCALPSALSSLILASVFIAWCQAETETIIEVPYKKADQPKWIWSWTSQWTSTKDGDGKV
LYQVCDVSSSGSEPNKWIRTDVFRVGDATRIDVTVQYAVSNCSTSVSLAYCKEALKLYVY
NTATQHKVPEPWQTPDVYHIFGITSPTNRSTGSLSRNFANTEKHSFLTNPSTPYYYLAIQ
YQGGCFELYSVTISY
>Apal_215204   A. pallida
TSSFILVFVFVACSKAGKETIIEVPYKSSQSKWIWAWTSNRWTRKTDGHGKVVYQVCDII
PFSEPNMWIRSDVFPIDDARRIDVTVEYAVYKCSSGHVKYCKENLNLYVVYKTAIQHTSAT
VPEPWKTPDVYHIFGTTSPTNRSSDILSRGFANTEIYSFLTNNAMSYHYFAIQYQGGCFE
LYSVTISYSVCPSLALP
>Apal_215207   A. pallida
MTTLLHSFLVYFLLKSMFMTSKADKETILEVPYKNSQGKWIWSWGSQWTKPEVGNVKIYY
QVCDVSSPSEPNKWIRTDVFRVGDATRIDVTVQYAVSNCSTSVSLAYCKEALKLYVVYNTA
TQHKVPEPWQTPDVYHIFGITSPTNRSTGSLSRNFANTEKHSFLTNPSTPYYYLAIQYQG
GCFELYSVTISY
>Apal_215211   A. pallida
ACSEAGKDTITEVPSIKNSQSKWKWSWTGQWDPKDYGKPFYQQCENSDSQTNKWIRSDVF
LVGDAKRIDVIVEYAVLKCSSVPSIQNCKETLNLYIKKTAIKHSPLYKLQGPLRRPKDYR
IFGTTFPTNLSTGILSRRFTNTETYSFFTNSPTPYHYFAIQYQGGCFELYSVTISYSVCP
SLALP
>Apal_215213   A. pallida
ACSEAGKDTITEVPSIKNSQSKWKWSWTGQWDRKDDGKPFYQLCEPSDSNTNKWIRSNVF
RVGDAKRIDVTVQYAVSKCSSVPSLQYCKETLNLYVVYKTAIQHTISNLVPEPWKKPNFYH
IFGT
>Apal_215214   A. pallida
ACSEAGKDTITEVPSIKNSQSKWKWSWTGQWDRKDDGKPFYQLCEPSDSNTNKWIRSNVF
LVGDAKRIDVTVEYAVTKCSSVPSLQNCKETLNLYVVYKTTTQHTSGGINTIPEPWKQPND
YQVFGITSAAILSNGIPSRSFPNTETHSFLTSTSTPYHYFAVQYRGGCFDLYGVTISYLV
CPSIAL
>Apal_271591   A. pallida
CFELYLVTISYSVCPSLILPTNLIKLPRTIAPANGTIRVPGSCAENSQPSAGNATLYQHC
QANGEWISDALSVGCLCKAGYGRYLNGPGAVCKDCPMSTYQNSPSASPCKPCPLNSVQNV
DRKRCNCKSSFYRTDVESAFHNCTGQPSAPQSIEVISKNATFITLRWSTPSNLGGRSDIY
FNVRCKKCNNAGKNCTAECTGAFIFHAPTSSMKATVYHLSVYTYYLFEVFAKNGVSPQAD
KKGDNPKYSVLLSRTGESTPGPAVVTTVKILNATTVFLSWTVQEPNGAITYYEVIYYPVG
QETEKETKNTTSNNITINGLIYGKEYIFKVRASTNIGFGISSNITKTIVLPNKGSHTGEK
GSSTELILGIVIGVSLLIALVCVIVVIFIYRRRKRKEENSPSNDLIPMASERLFIDPSNY
NSPLEAMRSHTNEIELKNLELKINIGEGEFADVYKGIYITGSNREVVAVKILKPTSSTKN
KKDFIREASIMGQFKHPNVIELKGVVTRSPEYPMMI
>Apal_306157   A. pallida
KKLTTKKKDGKKKPGAVPIKKELLIAIVVGVLVLLVFIIVLLIVCIYKRSGSSKSGDMGN
TSSWMFSTEMIPLDDGQKMYIDPSTYGSPMEALLSNTEEIDCHDIKLEKNIGGGEFAEVY
KGILKKGTDTEVVAVKILKPNASYKSREDFILEASIMGQFKHPNVIELKGVVTRSPDHPM
MIVTEFMENGSLDHFLKKLDGKLSVLQQVGICRGVASGMEYLSEMNFIHRDLAARNILVA
DNMASKISDFGLSRELEDNPDSEYQTQGGKIPIRWTAPEAIRYRKFSSASDVWSYGILVW
EIMSFGERPYWEWDNFEVMGRVDQGYRLPAPMNCPKTIHNIMLDCWEKDKNNRPKFSDIV
KQLDELIRSPEKISDQPASRFSTNGEANFGEVKSVEDWLTQIKMDKYADLFKQAGYVNLD

QVKSLEEDDLKGLGIQLIGHRNKMRKSIKAMKIYDENKA*
>Apal_306159 A. pallida
KKLTTKKKDGKKKPGAVPIKKELLIAIVVGVLVLLVFIIVLLIVCIYKRSGSSKSGDMGN
TSSWMFSTEMIPLDDGQKMYIDPSTYGSPMEALLSNTEEIDCHDIKLEKNIGGGEFAEVY
KGILKKGTDTEVVAVKILKPNASYKSREDFILEASIMGQFKHPNVIELKGVVTRSPDTPM
MIVTEFMENGSLNHFLKIHDGKLTVLQLLGMARGVAAGMTYLSGINFIHRDLAARNILVA
DNMASKVSDFGLSRELNESEDNEDSEYTTQGGKIPIRWTAPEAIRYRKFSSSSDVWSFGI
LLWEIMSFSDRPYWDWDNFQVMDRVEEGYRLPAPMDCPKSIHNIMLDCWEADKNNRPKFA
EIVKRLEELIFSPEKITDDSANARYANTNVSIDLEDETPVQEWLESISMTRYANSFKDAG
YVDIAHVKLIKDDDLTRIGVNLIGHRNKISKSIKKMKRKSKVPERDHFADKGEEEAFV*
>Apal_306140 A. pallida
AEKNGEKPKYAKLTKRTSESVPGVPLEMTAKVLNSTSVHLSWIVFEQNGLITYYQISYYP
VGENKEIRVMNTTKNSITITGLEKGTKYVFKVRASTSVGLGPPGNITKVVALPSTNENTG
GFSQDQLFGVVGGVLAAIVLIAVVVIIVACIYRKEKKRRSDSKLHLANSEFIPLDGHKMY
IDPSTYGSPMEALMCNAVEIDSRDLKLDKNIGGGEFADVYKGTLSKTSISEIVAVKILKP
GSSTKNKEDFILEASIMGQFKHPNVIELKGVVTRSPDHPMMIVTEFMENGSLDHFLKKLD
GKLSVLQQVGICRGVASGMEYLSEMNFIHRDLAARNILVADNMASKISDFGLSRELEDNP
DSEYQTQGGKIPIRWTAPEAIRYRKFSSASDVWSYGILVWEIMSFGERPYWEWDNFEVMG
RVDQGYRLPAPMNCPKTIHNIMLDCWEKDKNNRPKFSDIVKQLDELIRSPEKISDQPASR
FSTNGEANFGEVKSVEDWLTQIKMDKYADLFKQAGYVNLEQVKSLEEDDLKGLGIQLIGH
RNKMRKSIKAMKIYDDNKA*
>Apal_307048 A. pallida
MLTMLLGRALVLIFLYLNGSLTEAKNKTLKLVPRKIHDGSKYIWPWKSTGEWTKNEGPGL
RKPVYSVCDIKPSVEPNFWLRSHRNHVKNATRVDVTLKYAVTECRADFKHCKDNLQLFVH
KTLKRYNNIQNKVPEPNKNIESYQNFGTTKPRSISKDLDKPELNNETYSFFTNRSTPYYY
LAIRYQGGCFRLFEVILSYSFCSSMALSSNLVHLTETIAPANGTIQVGGSCAQNAQPLAD
NMTLYGNCMANGEWSSDAIGGECLCMAGFGKYLNGTSASCKKCPDGTYQDTASNASCTKC
PLNSRVNSHRTMCSCKPGYYRFDNETYMDTCTGPPSEPRFPESTFINATSLQLSWSSPSD
LGSRLDVYYEIHCKKCNDEGKDCITNCTGANISHTPRTSRNATVQFLSPYTYYEFKIFAK
NGMSSLAEMRDNREPQFIPLPRRTKQSVPGVPKLLSPEISSTEIKLRWKLEKKNGLITYY
QVTYHELNKEENTLTKNTTKTSIIIKGLKPQKTYIFKIRASTKEGLGKPASKKLTTKK
>Apal_405536 A. pallida
MKRCSSTKMGSNYLKPSLIVGILLFCDLLGCVLSDNETLPLTIQDGWYKEKLPGDMLGFF
FNSGKYLVCDTTEKQEPKSWLRTEFIEFNGAKRVFIYISYTIRKCKSNSKYCKEQFKLYT
AQTSKSASSPKPPNGFEEIAVAKPTNLKDGVPDEKDVIVLNATIKAKMGGFYLAFLDQGV
CLTLYSIKVMYYFCSERGKYLDLVKFPKTISPAEDIVGSQGQVHGSCSDSNAKNTSELLG
VCTTKGVWKMNSHVRCLCKPGYGYLTTPTKKCQACGSGQYKSSLDNSVCKTCPIGSFPFA
QKTECRCKSGLFRMNKKNSEPCYAVPDGPKYANATTWNSTSIRVSWPPPSDKSVTFTIEC
FVCKNDKDEECVEPCNKKIIYHPGKIGLKSRVSVSGLKSSSRFKFQIFGVALLNQKVEKS
EWKYVVAFGKTKKGPPEAPNTVTKADKFKKLMLTVVVPVLAFLVLVIIICAVCLCRRSNR
KYATRVPGYDPSDTHELPTIGVKVYIDPTNYDNPEEALKNFAKE
>Apal_455194 A. pallida
MACNCQRMVFFAFLLLLKTFEKVSGDRVSVLNVPNNNGWTWADSGAEKRWYPQIAISNND
KFYIGCDIAPSQQRDNAWLRSDAIKVNNTRRIDVTVEYSATNCSARTKSLYCRTSFDIYA
HLASEMYTPDKIPDPYLNPGAYELISAVSPKTLAYPKIEKMQNVETHPFEVKPGHSYVYI
ALHYTGGCFAVYGIKAHYYQCPSKALSKSLIQLPRTMSPLKGSKQVKGLCAEHAQPKSNA
DNLYGFCEAEGKWSEDSYKGFCLCEAGYHEVHTSKGTECQECPSGTFKNVVSNSSCLTCP
KRSKQESDRTSCECETGYYRSDTETIFDPCTEPPTAPEGLVSTFHNATFLTMEWSPPKHF
GGRSDINYEVECKQCGIDGKHCKEDCDDAHVQKYLRVLTNTSTLATVKNLSPYTYYQFKV
YAKNGVSKVAENNQYKAQFAKLLIRTEEGVPGKPDITVSKPINPTTVYLRWNLKEPNGVI
IYYQISYFPVGMESSKMIFNTTNTNATINGLKGGQEYFFQVQAGTKFGLGKPRGTTQILP
PGKNEAIPFSWEILAGIVGAAGVFLILILIVVIICLHRKRRRYIQARSSSMEKLDGFLPL
DGHKLYIDPSNYGNPMEALMSVTEEIDRRRIKLEKLIGGGEFAEVYKGSLISCDTKPETV

AIKILKPKSSPKNREDFILEASIMGQFEHPNVLGLKGVVTRSTETPMMIITEFMENGSLD
HFLKSRDGMLTPLQLVGIARGVANGMAYLTDMCYVHRDLAARNILLTENFASKISDFGLS
RELYTSEDNPESVYMTQGGKIPIRWTAPEAIRYRKYSSASDVWSFGILLWEIMAFAERPY
WDWENEEVFTRVEEGYRLPPPMYCPKSIHDLMLECWEKDMNSRPKFPDIVKRLDEMIRSP
DKMNHQEIPSQSVGAQTNLGELTSVEEWLIDINMRQYGDKFVDAGLTELYQITLLDDKAL
KRIGITLIGHRNKIYKSICKLRQHFTAGPDIEITI*
>Apal_549316 A. pallida
MFFQLFATMECMRTSSFILVLIFLACSEAGKETIIEVPYKNSQSKWIWSWVSQWDPEDDG
KHFYQLCESSDSNTNKWIRSDVFSVDDETRIDVTVQYAVLKCASVSLAYCKERLKLYVYN
TAIHHSLIVDGSNAIPEPWKKPEVYHIFGTTSPTNLSTDSLSRNFTNTE
>Apal_549317 A. pallida
GDAKRIDVTVEYAVSKCSSVPSITNCKETLKLYVHKTAIQHSFVNNRVPEPWKTLEVYQV
FGTTSATNLSTGSLSRSFANTETYSFLTNSSTPYHYFAMQYRGGCFELYSVTISYSVCPS
LALPTGLIQ
>Btue_4512 B. tuediae
MILRTILIRNTRHRVARFLFVGRHPKPSDIRNFHQRVTFGASGFSCGKLCRFAERPYWDW
DNFQVMDRVDTGYRLPAPMNCPKVIHNLMLDCWEGDKNNRPKFADIVKRADELIRSPDTL
NDNICTSRFSDAAAETNFGEVTSVEEWLDNINMGKYSNQFEQAGYVNLETIKVLSEEDLT
RIGVKLIGHRNKINKSIKAMKRHFDKPDSSC*
>Btue_32074 B. tuediae
MAYKSFSSTLVIGVILLLHFCGVALSNTDVLLKGPVNGWFSDGGPVSPQIGSWFLYDGKL
YSVCDTSATDREPTNWLRTDFIDLKDAIKVDIVVNYTVRKCQPGKMHCKETFKLYGYQTS
KTLPKPNPI
>Btue_32401 B. tuediae
MACNCQRMVFLNLLLLVLLLGKVIGDRVQIMSVPDKSRTTWKWSWTPSGTQTMWNYDTLL
VGSQTKIYYHACQVVSSGQPNTWLRSDIIDVQHARRIDITIDYAVQNCSAIINAQHCKHN
FDLYA
>Nvec_33736 N. vectensis
MYIDPSNYGDPMEALMSVAKEINKDDLKLEIIGGGEFADVYRGVLTNEDKTQEKVAVKI
LKPGSSHKNREDFCVEASIMGQFDHPNVIALKGVITRTRPMMIITEFLENGSLDNFLKSN
DGRLTTLQLVGIGRGVAAGMAYLSEMNFIHRDLAARNILVSDNLAAKVSDFGLSRELDDS
PENEQSEYQTQGGKIPVRWTAPEAIRYRKFSSASDVWSYGILLWEIMSFGERPYWTWDNF
QVMDRVEGGYRLPAPMKCPKVIHNLMLDCWDKEKTSRPKFADIVRRFDEVIRSPDKMNDE
FTGSRIRYVEFSATNMSMVKSVEEWLENINMGRYADAFVSSGYVDIDRIRHLEDDDLLKM
GINLIGHRNKIRKSIKHINQKLETGELAILNSKV
>Nvec_39123 N. vectensis
MKVWVYLVVDLLVFLYSSHAEKVPLANFMNGKKWRERWSDSGSSSWDLSRLELGACDDTS
SSQPNGWVRSDVINTGPATRIELTATFTARDCSSFSGGSYCRHDFDVYAHQSETQYWGLA
PDPNNSPPGTYSKIGTLNATKLWTSFVNKTQNVQTFSLILENSKPYVYLALHYNGGCLAV
QSILVEYFRCADMTLPSTLVQLRATVAPANNSINVSGACAPNSKQVSGAGDLYGYCQSDG
EWSSGLFSGECQCDAGHQNTTTGGGGAECQACHVGTYKAIAGDLGCTGCPNNSAAMETGS
TGCTCVSGFYRKSGESNNNTCTGPPTAPTSLDTNFLNDSVVTLSWSLPNNTGGRSDVYYR
VECFTCNGNSIVCVKACGGTVTQHGTSARVSGLSAFTYYMFRVYARNGVSDEAERGGQEA
EFAKLVIQTHDAVPGKPEITAVEKVGLTHVRVIWKLLKPNGIITSYELKYHVTGKSANEK
AVTINATRATVDIDRDKGYQFRVRARTRLGYGPFSDPVGLQAGQTSSSSSSPLVIATSVL
GAVPVLVILLVAANLSVIKRLGSGNFGHVDKAMAYGIPGFPGQVTVAVKTLKASADEKDK
TDFLAELNLMKSLRPHPHVVRLIGCCTRQAGELSSTNIEFQVANHLAIILEYLPYGDLLG
YLRRSRGHEDWYCSGDLRPPSRLVSKDMVKFAWMIADGMAFLAANKDYINLDLYNDELYA
NFDAA
>Nvec_39124 N. vectensis
MKVWVYLVVDLLVFLYSSHAERVLLANFMNGKPWRERWTSSASSSWDLSRSELGACDSSL
SQPNGWVRSDVINTGPATRIELTATFDARDCSSFSVGSYCRHDFDVYAHQSETQYSGLAP
DPNNSPPQTYSKMGTLNATKLWTSFGNKTQNVQTLSLILENSKPYVYLALHYKGGCLAVQ

SILVEYFRCMNMTLPSTLVQLGATVAPANNSINVSGACAPNSKQVSGAGDLYGYCQSDGE
WSSGLFSGECQCEAGHQNTTTGGGGAECQACKVGTYKAVHGYFVCASCPDNSVAMEIGST
SCTCVRGFYRKANESISSACTGPPSPPTSLYKPFLNSSAIILSWSAPNNTGGRTDDDGSV
CVEACGGTVAQQGTSARVSGLSAFTYYMFRVYARNGVSDEAEMGGQDAEFAKLVIRTRDA
VPGKPEITAVEKVDLTLVRVSWKLLKHNGIITSYELKYHVTGKSANEKSVTINATRATVD
IDRDKGYQFRVRAQTRLGYGPFSDAVSLQAGQTSSSSSSPLVIGTSVLGAVLVLVILLVS
AQEEYDDIGETNPGLDAIYSCVGEDEAWEVPPQNLSVIKRLGSGNFGHVDKAMAIGIPGF
PGQVTVAVKTLKYYLAIILEYLPYGDLLGYLRRSRGHEDWYCSGDLRPPSRLVSKDLVKF
AWMIADGMAFLAANKCVHRDLAARNVLVGEHNTCKISDLGLARDVSQDIYTRTSSMVLWD
SSLGDFYNCVVVPLSLAGQTGSSSSSPLVITTSVLGAVLVLAILLVAAQEEYEDMGETNP
GLDTIYSCVGEDEAWEIPPQNLSVIKRLGSGNFGHVDKAMAIGIPGFPGQVTVAVKTLKS
TAQKKDKTDFLTELNLMKSLRPHPHVVRLIGCCTRQTGELSSTISEFQVASNS
>Nvec_40993 N. vectensis
MMFANAKNPSGELVPRFVAEPDQRILSFSHFSTTNISVIAFISGAVWRARFSGSAQNAVE
HTFCGAHADQPYKQERAYFGKISLVLVYNGTTSYSMADLGDFYCIWDIHSRTFRHSGRFD
IVLLFSITTLPWSINLRIMDFFILVTSRNLKGKSDNWSSAIIENRSTIVSDRQFTICACF
YDVPKCEIIFVFFNIINYAENWLDILLDGPRAGWSITGKSVPGQIANWLHSGSSNQYSVC
DVSSSKEPTNWLRTDYINAKDAKRVDIKVIYTIRKCKDLSKFCKETFNLYVYHASQSEPS
PDPATMPFTWLRKANVTHLPSGPATDADKNTMLVSLVPRKEGIFLAFYDQGACMSITKVV
VSFNFCPPRGGILVDFVRTVSPLSDSVQKKVEGTCVSENSVNTTALTAVCTSDGNWNLSG
DVKCYCKKGYAYDKVNNDCKGCPPGNFKSTLSNTGCRPCPENAQPNTLRTECVCRNEYFR
LLNHNMTVPCKPLPDGPRYANASALNSTAVRLTWTPVTPMSEGISYEIDCFVCTDEKDTK
CVVQCGKSPVSYDPGRSGLVQPNATIAGLSPETKYMFKVYTVTDLNKVADRKMWQFAQAF
VMTGKGSKKPPANQEKSADFQKMALTIGIPVIVFIIVLVVGCAVFIWK
>Nvec_40994 N. vectensis
MCHKPYNTISSFMLFTHVLVSEVDLPTCGQRVYIDPSNYNDPEEALRNFAKELDKRWLKL
EKVIGGGEFGDVYKGTLKRPGENTIPVAIKTLKQGSTSKNRGDFLSEASVMGQFCDPNVI
FLEGVVSKTHPLMIVTEFMSNGSLDNFLKKMDSKLSLIQLMGMARGIASGMKYLSEMNFV
HRDLAARNILVTETLVCKVADFGLSRELEDSAYETSGGKIPVRWTAPEAIKYRKFSTSSD
VWSYGILLWETFSFAERPYWDWSNFEVMDRVETGYRLPPPMSCPKVIHQIMLECWDADRT
KRPSFALIVKQLDSLIRSPEKLKDNQSVIQSKPNIDYSEITTVEEWLGSIKMGQYTEHFK
QAGYRDLQTIAESEEPDLDGLGVKLIGHKNKIRKSIREVKGKLKRETSLPV
>Pcar_152315 P. carnosa
YVDPSNYGDPMEAVKTFAAEIDRCHIKLECAVGGGEFAEVFKGLFEDSIVAVKILKQGAT
SKTRDDFISEASIMGQFKHPNVIQLIGVVTVSRPMMIVTEFVEGGSLDTFLKNNEGKLTI
LQLIGMIRGVASGMVYLSAINFVHRDLAARNVLVGENMVCKVSDFGLSRELEDNPDSEYQ
TQGGKIPIRWTAPEAIRYRKFSSASDVWSFGIVMWETMAFGERPYWDWSNFEVMDRVEGG
YRLPAPVKCPKILHTIMMDCWDKDRSSRPKFEEIVKRLDELIRTPEMLNDSLVCYTSAVS
ADFTKLSTINEWLGSIHMGQYAANFKTAGYKDLTQVTCLKDNDLKEIGVTLIGHRNKIYK
SIKSMRKHFDNMPEAV*
>Pcar_152322 P. carnosa
YVDPSNYGDPMEAVKTFAAEIDRCHIKLECAVGGGEFAEVFKGLFEDSIVAVKILKQGAT
SKTRDDFISEASIMGQFKHPNVIQLIGVVTVSRPMMIVTEFVEGGSLDTFLKNNEGKLTI
LQLIGMIRGVASGMVYLSAINFVHRDLAARNVLVGENMVCKVSDFGLSRELEDNPDSEYQ
TQDYPLTPWRVQSLILYKPCSPQLKHFEQQGPYAQN*
>Pcar_315635 P. carnosa
MLHFGLYFLTAMTFVAVASSIELVLDNTAEKKQNWFWIIKDPPLPQGASGWERLSNTRLR
YEACDYDLQQSDPRPNYWLLSDQIAVRDANRIDVTVKYLIRSCVKVPKKGGPYCVNVFDL
YVHQSDQFIADPDLYPDPLSNSKAYEKVAEINQTTDIITSETKSILVKGNYVILAFHNYG
ACINLFSVKVTYNVCPDETLSSSLVSLQRTVAPANDSATIRVEGNCITDAVQASGSLFIH
CESNGEWNTTGLEGKCICKEDMENNGGTCQACPDGKYNDQMG
>Pcar_328883 P. carnosa
GVCVTLTKVVISFKYCPVAGSTLVTFPRTVAPANDSDLTEQEGKCTDVNSVNKVKLVSVC

LSNGEWNNTDDLMCLCNPGYELVNGSVAALECKECPSGAFKSAISNAKCVQCPLKSVSNA
ERTACTCEEGFYRSSDVENCKALPQAPVKANFTKVLSTRVVITWHRSPDDNGKITYALDC
FRCKPGEDKNCKELCDRQVRYLPGKENITGVNVTVHGLSPSSFYLFRVYSVNELNQLERN
KDKWRYAEVSVETKESKHHPTEGGAGGLNKTMKMALYIGVPIVAVLVLLAILFALCFCRS
RSKEGYKPPVELKDGQVILPMNGQRHYIDPSNYEDPEEALRTFAKELEKKWISLEKIIGG
GEFGDVYQGTLSRPEEEPLLVAVKTLKTAAGRKNRQDFIAEASIMGQFCDPNVIFLEGVV
TKTSPMMIVIEFMSNGSLDNYLKKLDGKLTILQLLGMARGVSSGMKYLSEMNFVHRDLAA
RNILVSESMVCKVADFGLSRELEDSAYETSGGKIPVRWTAPEAIDYRKFTPASDVWSYGV
LLWEIMSFAERPYWDWGNYEVMDRVKGGYRLPPPLGCPKAIHQIMYGRLLGVFHLGRLGD
CCSNFIELVRGTNQLV*
>Adig_00802 A. digitifera
MSAYPWEVAKFSLPIKERTSRVILAIHDSGACIVLNSFMVTYSVCPDMVLPDSLLVLPQT
VAPTNESEIVRVSGKCVDNSKETLQGPEAICGKNGKWILTDSAKEACLCNPGWQRDVAKC
RECPVGSFKENLGNTRCTKCPMNSFSNADRKYCKCRQGFFRAPRETIAENCTALPSKPRH
LKTLTKNQTTVVLSWSHSHHRGGRSDLFYEIECKIACQKEQTSCSQDCGSQVLFLPRQRN
LSDTQATITKLFPQTSYRFTVYAKNGVSGVAEGKGFASNFAHLKVDTLESVPGKPELTVK
RIDSTSVRVSWNLKNGNEGIHYFLVTYYPLEDKFDRHTKNTTESAITIDGLQPDVVYQLV
VVAKNRMGYGSVSEEVNIQGKGTVTVAEIQQSEDNTKLWLLTGAVVGGTLFIFFVIAIVV
VIRAKRRKSDRKRANTFEAAELFSTDGLAQYVDPSNYGDPMEAVRTFAAEIEENQIKLES
TIGGGEFAEVFKGFYDGSAVAVKRLKLLLVTETAYGRVQFGVRCGIKEAVRLSKTRPMMI
VTEFMEGGSLDSFLKEQKGKLTTLQLIGMIRGVASGMAYLSAINFIHRGGKIPIRWTAPE
AIRYRKFSSASDVWSFGIVVWETMSFGERPYWDWSNFQVIDRVEGGYRLPVPVYNANFKT
AGYKDLTQVTCLKESDLKEIGVALIGHRNKIYKSIKSMRKHFDNMPEAV*
>Adig_00805 A. digitifera
MEQFWATGLIVLAFSSWASSKEEYIMKTPDCRTCTWDWDTSAFLRFSGLISPWYHLYGLS
TNNRYAVCNPDNKEEPNNWLRSYVIEVGDIGRLDITFRYFTRACQPSASFCKEYFYAYVW
ESNTSVTVRQIPDPIKNFQLYRRFANVTRTRQSDETNLTVYLPVTSKYIVMGIRDQGGCR
ILYSVKISYKVCIEKTLEDSLLSLPLTISQKESTPVQGICSANSRQIVPGNLSVLCDSDG
EWNTSRLESRCVCKKDMESKGGVCTVPSKAQVFFEQLSDTVVRISWRLDSANGMLLAYYL
TYTRVDDTKDSKTIKTTKTELILTGLKLGKTYSFVVAAENHIGRSYSNSVIKTIIDNDSS
THSTSDALTFIVICVILSAILLIGATFVGVYVYRQRKARNRSGRKRERVSRVGEDNSDNL
PATPQTSRGDVMTNHGQYMEMTDTPDLELERNEIKFIRLLGSGNFGEVYRATVKDCTVAV
KSLKENASQKDKQDMFTELHMMKYLKSHNHVVQMIGYSTRSGECRISLKVNE*
>Adig_03916 A. digitifera
MEHFLIAALIVLALSSLASSKENYLMETPDCNSCIWQWNNEVYSPVNKIITPWYHPSGRL
IFNRYHICNLRNSQEPNSWLRSYVIAVGDIERLDVTFRYLSHRCDPPVGFCKEYFYAYVW
ESNTSVAEQPIPHPINNFKLYRLFANITRQSDQETNLTVPLQVTSKYIVLGIRDQGGCRI
LYSVKVFYKVCGAETLEDSLVTLPSISTQVESTSVQGNCTANSVQIVPGNLTVVCDKDGE
WNASRLESSCVCKEGFENMTGECKECSVGTYNDGKGFDCTVPGTPEDITVTYLSETNSVT
LSWFLKCKNGIIQEYGIEYFNMDDSSGSKSTKDSEIQISGLPAGKTFKFQVYAVNNFGTG
SPGVKEFLIPKGNVEKHLPLTFIAAASGGGAFVLIIVAVICFAFVVRSRRRSAQSVQKDY
MRSLERGLDNNLPMGQRRYINPAEYLDLMELLQTFTTEVDRSKTKLNGMIGQGCPMMILT
EFLESGSLDHYLKTCKGQLTTIQLLGIARDVACGMVYLSGMNFIHRDLAARNILVGENLS
CKVSDFGLSRELADDNPDSEYETQGGKIPVRWTAPEALQSRKFSSASDVWSYGVLFWEIM
SFGDRPYWEWNNYDVRNLVNLINFQRKNHVSKKV*
>Adig_04039 A. digitifera
MEHLWITGLIALVFSSWASSKQKYIMETPDCRTCKWDWDTSASLPINSTTEGLTSKTSPW
HHPQGLETNNRYTICYLDNKEEANNWLRSYVIKVGDIGRLDVTFRYFIRACQPSVSFCKE
YFYAYVWESNSSVKPQQIPDPIKNFQLYRRFANVTRQSGETNLTVPLQVTSKYIVMGIRD
QGGCRTLYSVKISYKVCIEKTLENSLVSLPLTISEEESTPVQGICSANSGQIMPGNLTVL
CDSDGEWNTSRLESRCVCQEDMENRGGVCLETPSEPRNVSVYFVNESSAVLTWLLPEITG
TPIDISYDVTCQKSCEYFGSDCDDQTCNRGIDDQFTAEELNTTIFTATNLAPFVNYTCRI
TAKNRVSKRAESKTEATDGERNSFTYINLTTKGSVPGAPEDITVAYLAETNSIILSWIVK

CKNSIIHEYRIEYFSVDDSSGSKNLSTRENKIQIGSLPAGKTLRFQVYAVNNFGIGSPGV
MTFQIPKGNFGKLLLTFIAAASGGGAFVLIIVAAICFACVVRSRRFRRSAQSILNDYMRP
LEEGFDSNLPMDQRRYINPENYLDLKELLQTFATEVDRRNTKLNGLIGQGEFADVYKGSI
QTPKGNLVVAVKVLRPGSSEKNQKDFLSEASIMGQFNHPNVIRLVGVVTQTCPMMILTEF
LESGSLDHFLQTRKGQLTTIQLLGMARGVACGMIYLSGMNFIHRDLAARNVLVGENLSCK
VSDFGLSRELADDNPDSEYVTQGGKIAVRWTAPEALQSRKFSSASDVWSYGVLVWEIMSF
CDRPYWEWNNYNVMNRVENGYRLPSPINCPKLVHNLMLNCWESDKIKRPTFADIVDSIDN
FLLSPEDLNDGLSPVAEKFKAKPQQEFQSVDEWLHYINMDKYSDVFHAANINSLDKVTEL
GDKELREMGIKLIGHRNKMTKSIQAMKENSVQ*
>Adig_12429 A. digitifera
MEHLWITGLIALVFSSWASSKQQYIMEAPDCRPCTWNWDTSAFFPFNGSTGSWTHPIGQN
TNNRYAICNPDNKEEPNNWLRSYIIEVGDIGRLDVTFRYFTRACLPSASFCKEYFYAYVW
ESNTSVTPEQIPHPINDFELYRRFANVTRQSYETNLTVPLEVTSKYIVMGIRDQGGCREL
YSVKISHKVCIKKTLEDSLVLLPLTISREKSTPVQGRCGKNSRQIVPGNLTVFCDSDGEW
NSSRLERRCVCKEDMENRGGVCLACPSGTFNEGKGFNCTETPSEPRSASVHFVNESSAVL
TWLLPEITGTPINMSYDVTCQTSCEYFGNDCDDQTCNRGIDGQFTEEGLNATIFTATNLS
PFVNYTCKITAKNRVSKRAESKTKATNGERNSFTYVNLTTKGSVPGAPEDITVAYLVETN
SIILSWTVKCKNGIIQEYKIEYFSVDDSSGSKNLSTRDSKIQIGNLLVGKTLRFQVYAVN
NFGIGSPGVMTFHIPEVGNRGKLPLTFIAAASGGAAFVLIIVAVICLARVVRRRRIRHSP
QSILEDHMRPLEQGFDSNLPMDQRRYINPENYLDLKELLQKFTTEVDRCNTKLNGLIGQG
EFADVYKGSIQTPKGNLVVAVKVLRPGSNEKNQKDFLSEASIMGQFNHPNVIRLVGVVTQ
SCPMMILTEFLESGLLDHFLKDLAARNILVGDNLSCKVSDFGLSRELADDNPDSEYVTQN
ISGGATHNVSTVTDNTYI*
>Adig_12482 A. digitifera
MEHLWISGLIALVFSSWASSKQQYIMEAPDCRTGIWDWETSASLPINGKISSWYHPSGLN
TNIRYAICNPDNKQEPNNWLRSYVIEVGDIGRLEVTFTYSIKACQPSAPFCKEYFYAYVW
ESNTSVTTEQIPHPIKAFQLYRRFANVTRQSYETNLTVPLEVTSKYIVMGIRDQGGCREL
YSVKISYKVCIKKTLEDSLLLLPLTNTQEKSTPVQGSCGKNSRQIVPGNLTVFCDSDGEW
NTSRLESKCVCKEDMENRGGVCLACPSRTFNEGKGFYCTETPSEPRSASVYFVNESSAVL
TWLLPEITGTPIDMSYDVICQTSCEYFGSDCDDQTCNRGIDGQFTAVGLNTTIFTATNLA
PFVNYTCKITAKNRVSKRAESQTQATDGERNSFTYVNLTTKGSVPGVPEDITVTYLAETN
SIILSWIVKCKNGIIQEYRIEYFSVDNSSGSKNLSTRDSKIQIGSLLAGKTLRFQVYAVN
NFGIGSPGVKTFHIPKVVNLGKLPLTFIAAASGGGAFVLIIVAAICFACVVRSRRFRRSA
QSILSDYMRPLEQGFDSNLPMDQRRYINPENYLDLKELLQTFTIEVDRSNTKLNGLIGQV
GLIAQRVEHCTTIEEPGSSEKSQKDFLSEASIMGQFNHPNVIRLVGVVTQSCPMMILTEF
LESGSLDHFLKTRKGKLTTIHLLGMARGVACGMVYLSEMNFIHRDLAARNILVGENLSCK
VSDFGLSRELADDNPDSEYVTQVMNRVENGYRLPSPINCPKLVHNLMLNCWESDKTKRPT
FADIVGSIDNFIRCPKDLNDDLSPVAE
>Adig_13888 A. digitifera
MSSAEILVIEPPSNFGVSTWQASKEKGSGGGGWIMPTPFVRAFQVCDIKANPENWLRSSF
IQLKGAKRLEITMAYKMKGCPSQAKDTCKTYIGLYVQHSDGELVDSDPLKVKYDFVENIV
PEGSPLSPDVLTPFTYHGEIVTKAQGLYIAIKDEGACIQITNITVGYNYCPEKGRNLIMF
PRTIAPVNDSNLMRKIGKCSDKNAVSQEALVGVCLSSGEWNISKNAKCLCQKGYELTDSF
ECKGIAIYAHRQLTTSSCVSARMSRY*
>Adig_13889 A. digitifera
MNPYKWSFIVFLTTAVSKVVGDSEILVIEPPSNLRVSTWQASKEKGSGGGGWIMPTPFSR
TFEVCDIKANPENWLRSSFIQLKGAKRLEITMAYKMKGCPSQAKDSCKTYIGLYVQHSDG
ELVDSDPLKVKYDFVENIVPEGSPLSPDALTPFTYHGEIVTKAQGLYIAIKDEGACIQIT
NITVGYNYCPEKGRNLVMFPRTIAPLNDSNLVMKIGRCSDNAVSQETLVGVCLSSGEWNI
SKNAKCLCPKGYELTDGCKECPKGLFKSTISYSKCERCPQNSVPNPTRTSCTCEDGFYAL
TLLVPCKPLPVAPLNASAIIVKQTSLTISWVPRDDANSTLGYSIDCFRCKSLQDKECRES
CSQNVKFQPGGDVIYTNNVTALGLQSGSFYLFRVYSVNELNQQEKTQTNGIILQCMCIPR
VRNPELISYLNCMQWRRRRKKGYSSPVELKDGRVSLPTRGQQLYVDPSNYGDPEDALKDF

AKEQDKRWIQLDIIGGGEFGDVYKGLLTRPDEDAIPVAVKTLKADADQRSRKDFMLEAS
IMGQFCDPNVIFLEGVVTKTLPLMIIIEFMSNGSLDNFLKKMDGNLTVQQLLGMARGVAS
GMKYLSGMNYVHRDLAARNVLVSEGMVCKVADFGLSRELEDSAYETKVIDRIRGGYRLPP
PLDCPKAIHQLMLNCWQFDRNKRPKFADIVKKLDVFIRSPDKLNVVAPAEPKSPKLDVSG
VTSVHDWLDSIKMGQYSDLFSRAGFTQLSQFAEEEELDLNDMGIKLIGHKNKIRKSIKDV
KRTLNKDNAL
>Adig_13891 A. digitifera
MKANAYRRHFIASTIFFAILESWRMQVGAEKGILLHKSQNMVSWNWLTSIKFGPGSGWSR
DDNSGGYTVCDLSDTLVNSTNSWLISEYVDLQGAKEIHIDAEFTVRECPSQLRYCKQSFN
VYTMQVNGPVVGGISEGDVQSGNFAFVDKVNATNLWIPRNAVQRNQAKVTFDVNGSKGFY
LAFQDKGACVALLTVTLSYNYCPELANNGATFKKTPAPLTSNANISVIGECFNGSSVYPS
QRIMSMMCLSSGQWXXXXXLADNISTCLCPSGYELIMDTCSECFPDYYKDSISNGKCVPC
PDNSYSSSSRNSCLCKEGFYRTTQDANNESCSAPLSGPRDINITIISNNVAIVVWTRPAF
DGGRADLRYDIHCGVCANMRQCSNSCLEVTFWPSEVDLITPLVTLSNLHAEVLYNITVIA
KNGVSHQLGISSAQSLYKTFFLPRETSTRRPRTSNINSPVPTAYGNLTITDAMANLGQQR
LNNTVPVAVGISVAVTFVICFFVAVVVIFLLTKSWKKKQLCLRSGFVISCIGVPV*
>Adig_15481 A. digitifera
MEHFWITGLVALVFSSLVSTEQHYIMETPDCSSCIWSWDTAAYFQFPELGLSWHQPIGKS
NLYSICNPNNPQEPNNWLRSPKVIEVGDIQRLDVTFRYSSRQCLQSASFCKEYFYAYVWE
SNTSVPTREIPHPINDFQLYRRFANITRQSDQETSLTVPLHVTSKYIVLGIRDQGGCRTL
YSVKVSYKVCSEKTLEDSLVSLPSTVSLVKATPVQGICVANSRQIVPGNLTVFCDSDGEW
NTSRLESRCVCKEDMENTRGVCTACPSGTYNEGRGLNCTETPSKPRRAAVYFVNESSAIL
TWFVPEITGTQTNVSYDVNCRPSCEYFSGCENKTCNSDINGQLTGEGLKTTMFTAANLAS
FVNYTCKITAKNRVSKIAAAKTRASESEGTITYVNLRTKGSVPGAPEYDTVAYHTETNFI
TLSWIVKCKNGIIQEYRIEYFSVDDSSGSKTLSTRHNKIQIGSLPAGKTLRFQVYAVNNF
GTGSPGVTTFQIPKGNPGKLPLAFIAAASGGGAFLVIIVAVICFACVVRGRRRSAQRMQK
DYVRCLERGLDNNLPMDQRRYINPGDYRDLMELLQTFTTEVDRSKTKLNGMIGQGEFADV
YKGSIQTPKGKLVVAVKVLRPGSNEKNQKDFLSEASIMGQFNHPNVIRLVGVVTQSWPMM
ILTEFLESGSLDNFLKTRKGQLTTIQLLGMARGVACGMVYLSEMNFIHRDLAARNILVGE
NLFCKVSDFGLSRELADDNPDSEYETQGGKIPVRWTAPEALQSRKFSSASDVWSYGILVW
EILSFGDRPYWEWNNYDVINRVQSGYRLPLPGNCPKLVHNLMLNCWESEKTKRPTFADIV
DNIDKLLRSPEDLNDGLSSVTEKFEVKPQPEFQSVDEWLQHINMDKYSEVFLAANVNSLD
KVTGLGDKELREMGIKLIGHRNKMNKSIKAMRENSVQ*
>Adig_18229 A. digitifera
MGVNPHNSYAICNPDNKEEPNNWLRSYFIEVGDIRRLDVTFTYSIRVCQPSASFCKEYFY
AYVWESNTSVTQQQIPDPIEDFHFYRRFANVTRQSGETNLTVPLQVTSKYVVIGIRDQGG
CRELYSVKISYKVCVEKTLEDSLVSLPLTMSEEESTPVQGICSCNSVQVVPGDLTVFCDS
DGEWNTSRLESRCVCREDMENRGGVCLQTPSEPRSASVYFVNESSAVLTWLLPEITGTPL
DMSYDVTCLTTCEYFGSDCDYQTCNRGIDGQFTAEGLNATIFTATNLAPFVNYTCKITAK
NRVSKRAESKTKATIGERNSFTHVNLSTKGSVPGAPEYIAVAYLAETNSLVLSWVVKCKN
GIIHEYRIEYFSVNDSSGSKTLSTRDNKIPIGSLPPRKTLRFQVYAVNNFGIGSPGVTTI
HVPKGLLSNTSHLNAAIIIKLP*
>Adig_18761 A. digitifera
MVNLESIQGQRFWRCRDSKLPNPDPLQERYDFVENIVPEGTPLSPSVAKPFLYHGAMVTK
AQGLYIAIKDEGACMSITNITVVYNYCPEKGRNLIMFPKTSAPLNDTHLVREIGNCSDKN
SVSQEALVGICLSSGEWNISKNAKCLCQKGYELTDLFGCRECPKGLFKSTVSYAKCHRCP
QHSVPNPTRTSCTCEYGFYALTPLVPCKREFS*
>Adig_18762 A. digitifera
MNPYKWSFIIFLTTTVSQVVGDSEILVIEPPSDKGVSTWQVSKGKGSKGAGWTMPSTFQR
IFGVCDVKRNPENWLRSFFIQLKGAKRLEITMAYNMRGCPSDAKKSCKTYIGLYVYHSDK
ILVDSDPAKVNYDFVQKIVPEGPPLRPNVQASFIYHGEIVTEAQGLYIAIKDEGTCIAIT
NITVGYNYCPEKGRNLVMFPRTIAPSNDTNLVMKTGRCSDKNTVSQEALVGVCLISGEWN
ISKNAKCLCEKGYELTDEDECKECPKGLFKSTISYAKCRRCPQHSEPNPTRTSCTCEDGF

YALTLLVPCKPLPLAPLNASAIIVKQTFLTISWVPRDDVNSTLGYSIDCFRCKSLQDKEC
RGSCSQNVKFQPGGGVIYTTNVTVLGLQSGSFYLFRVYSVNELNQQEKDRDKWNYTAVRR
RRKKGYSSPVELKDGKVSLPTRAHQLYVDPSNYGDLEDALKDFAKEQDKRWIQLDIIGG
GEFGDVYKGLLTRPDEDAIPVAVKTLKADADQRSRKDFMLEASIMGQFCDPNVIFLEGVV
TKTLPLMIIIEFMSNGSLDNFLKKMDGNLTVQQLLGMARGVASGMKYLSGMNYVHRDLAA
RNVLVSEGMVCKVADFGLSRELEDSAYETK
>Adig_20930 A. digitifera
MEHFFITGLIALALSNLVSPKEKYLIKTPDCDSCIWEWNNKAFFQFNRIVSPWYHPPGRL
NRFDICNLHNSHEPNNWLRSYVIEVGDIERLDVTFRYLSHRCDPPVGFCKEYFYAYVWES
NTSVAEQQIPHPINAFQLYRLFANITRQSDQETNLTVPLQVTSKYIVLGIRDQGGCRILY
SVKVHYKFCNEKTLEDNLASFPAILAQIESTPVQGVCIANSRQIAPGNLTIFCDSDGEWN
TSRLESRCVCEEDLENREEMCSADFLNAQFRVDCCDISSKMTACPTGTFNEGRGFNCTEI
PSIPRNTKVTFKNQSTVKLTWQLPEVTGYHTDVYYDVECRNTCNDEVNCGETACRGQVSY
LPSKQGLHETHVLITHLSSFVTYEFRIYARNRVSGVAQKMHGIVGNYALYRVQISFEQLS
DTKVRISWRLDSANGILLAYYLTYTRVDDTKDSKTIKTTKTEVILTELKVAKTYSFVVAA
ENHIGRSYSDSVIKTIIGNDASPYNTTLIVICVTLSTILSIGATLACVYVYQQRCHLLPA
HKTRFSQLYLSHNSSSSRPFMQTNQNVQASYARISSRRAYAETVRGALADNMGKNELDSD
VTTLVTGLFVPSETLVPSGKKSLDHTPGPSGEQETEEMPETAEDEPVLPHLGVRFQDDFL
REMIGYSTRTEETQTDCTWFPHFPAQVSGPLLLILEYMPYGDLLGYLRISRGHNDTYNSG
EKKPTSRLTDMELLSFGWMIADGMSYLADMKVVHRDLAARNILVGENKICKISDFGLARD
VNSEVYVRTSQARLPVKWMPPESLFLGESSTKSDVWSYGIVLWEVFTIGDSPYPGVKPRE
VASLLERGYRMPRPNHISEELYAVMSECWLEKPEDRPTFRRICAAMKRLINDRRVGYSRG
TFDVV*
>Adig_24585 A. digitifera
RGSSEKNQKDFLSEASIMGQFNHPNVIRLTRKRQLTTIQLLGMARCVACGMVYLSEMNFI
HRDLAARNILVGENLSCKVSDFGLSRELADDNPDSEYVTQGGKIPVRWTAPEALQSRKFS
SASDVWSYGILVWEIMSFGDRPYWEWNNYNVMNRVENGYRLPSPINCPKLVHNLMLNCWE
SDKTKRPTFADIVDSIDNFIRSPEDLNDDLSPVAEKFEAKPQQEFQSVDEWLHYINMDKY
SDVFHAANINSFDKVTGLGDKELREMGIKLIGHRNKMTKSRQAMKENSVQ*
>Ever_12042 E. verrucosa
MKCNEGKSMAVAVKTLKTDHLAKNENDFLLEASVMGQFNDLNVIHLEGVITKSFPRMIVT
EYMSNGSLDRFLRLNDEKLTHLQLIGMARGVASGMAYLSAMNFIHRDLAARNILVNDDLL
CKVADFGLSRFVETEGSDGEHSISGGKIPVRWTAPEVIRYHKYSFSSDVWSYGILLWEIM
SFGEKPYWEWENHEIIEEVEKGFRLPAPKDCPRAVHNLMLSCWKANRSERPKFEHIRQVL
NGWITSPKTIVEDSKDSGIGDWLHSIKMGEYRSTFLNADYESPHQLMGIGDKDLLKIGVR
LIGHRKTILKAIKAFDDTKDADEINMSLTRTESNLLQPSQSDI*
>Ever_14808 E. verrucosa
MLVAAKTLKPNASEKSCRDFFLEASAMGQFDDPNVIRLEGVITKVVPKMIVTEFMLNGSL
DHFLKKNDGGFTAVQLFSMARGVASGMKYLATINFIHRDLAARNILVDDDMVCKVADFGL
SRELETAESCQGEYLTQGGRIPVRWTAPEAIKHRIFSTASDVWSFGILLWEIMTSAQRPY
WDWDNFKVMERVEDGFRLPCPVNCPQAVHELMLKCWDKDRSLRPRFQDIIVTIDKWIESP
ESLHQSIMRDRSNQIPYTESTTSVEDWLDNIQMSSYMPHFAAAGYENLSDLAGLLDNDLK
ALGISLVGHRNKMLRTIRSLPITKE*
>Ever_2330 E. verrucosa
FDDPNVIHLEGVITKSIPHMIVIEYMSNGSLDHFLKQNDGNLTDLQLVGMARGVASGMKY
LAAMNFVHRDLAARNILVNEALLCKVADFGLSRELENEESSRGEYCATGGKIPVRWTAPE
AIKYRKFSNASDVWSYGILLWEIMSFAERPYWDWDNFKVLERVDEGYRLPAPKNCPKALH
ELMLLCWEGHRDKRPEFSAIVGMLDDWIRSPETIRYEKLSKRPNSSASLNDPAMLIKEWL
HDIKMSSYIPHFTQAGFTQLSELGGLMDTDLQSIGISLIGHRNKMLRTIRSLPITDATNK
TPWNRAPSMKV*
>Ever_2431 E. verrucosa
GGGEFGDVYKGSMTDTEGKKHPVAVKTLKKDATPKSEKDFLMEASVMGQFDCPNVTKLEG
VVTKYHPWMIVIEFMANGSLDNFLKANDGRLSHLELITMAKGVAVGMEYLAGMKFVHRDL

AARNVLVDGNLGCKVSDFGLSRELETTDSSRGEYATTGGKIPIRWTAPEATKYRKFSSAS
DVWSFGILLWEIMSFAERPYWEWDNFKVLEEVDEGFRLPPPQNCPKGVHDLMLQCWNAER
SERPSFARITVVIDKWIRSPETMNDEIGLSSPIGEWLDSIKMNSYETNFVEAGYKSKSEI
TELTDEDLITIGITLIGHRNKIMKGINSLKSKKKGQSI*
>Ever_3065 E. verrucosa
KNLTFTSVDHDRIALTWLPPSNIDSTRIIYEIGCRVHCNLDKQCTTDRCKKLKFIPSRQN
LLHTNVTIAGFRNEGLLYVFEVRAKHRFYNNSEESLGIEWNFVAINYTDFKEVIPTVSKS
FNVVDVVIGVSVLFLAVLLTIAGTRCYNRWKRRKRMLSFDSENPEILTHCEVPLTDVTTH
YVDPSNYDSVENVLRKFTTKLNEKNLKTEAIIGEGEFGVVWKGHLQTDEKECTVAVKCLR
SGLSAKLRRDFLAEASVMGQFKNPNVISLHGVVVKKHTVKIVMEFMSNGSLDQYLVKNNG
RLTTLQLLGMARGIASGMNYLSGRNFIHRDLAARNILVDENLTCKVSDFGLTREICQTSN
YETSGGKIPIRWTAPEVMKHRKSFIASDVWSYGIVLWEIMSFGQRPYWEWDNFRVLQEIE
SGYRLPAPLKCPKTVHTLMLHCWEKERSNRPTFIRILDKLDEWIRSPETIHHSPATKARR
NTMELIEPQSPIHEWLDSIKMSKYASNFTEAGYNRLSQTVNLTEQDLSDMGITLVGHRHK
IHQSIPLVT*
>Ever_3805 E. verrucosa
ILKNLKLLATLKNEKDQTGKTHRFHFEPKSRPFSIVFLSTGSCTLIQEISLYSFFCEKNT
SSGVNLPKTEAPANGSERVNVSCPENTLNPGNDEAYGLCSSEGKWDIISPCMCKKGYTLN
TIEEGCKECQKNTYKDAVGNEKCTDCPSNSKASPSGQVHCSCQDGFYRFQGENYTHPCYR
VPSPVTEIKFIDRTETSITLRWQASEPPDQEVLYDIKCNKCTSGSNSGSCEEPCGSSVTF
KPSQNNLTRTNVTIRGLKGDTEYEFVIYSKNSNSLRINMTNWSSERKKIKTEGTSKQEGL
SKLVVIAVPVLVVVLMVIIIILGVCLFKRRKIRRFRASQRLRGGGDFPLIPGEKNYIDPT
TYDNPERAVSEFTKELDRDVIKFEALIGGGEFGDVYKGEIKLPGQPATKVAIKTLKAGAS
YKNRTDFLIEASVMGQFKNLNVITLEGVVTRSTPLLIVIEFMENGSLDKYLKENDGLLKP
LQLLGMTRGVASGMEYLAGMHFVHRDLAARNVLVNANLACKVADFGLSRDLGNTQDSEYE
TQGGKIPVRWTAPEAITYRKFSTGSDIWSFGILLWEIMSFAERPYWNWGNDEVMTRVDQG
YRLPPPMNCPKTVHQLMKECWLTDRTKRPPFKEIVRIIDEWIKYPEKLNEDYIKVRRTEP
LDYSTFTSVKDWLEAIKMGMYASNFSKAGYEELTEVAQLTEDELLKKVGVRLVGHRHKIY
QRIKEMSEDMRLKREQSVRI*
>Ever_19512 E. verrucosa
AYDNESIFENTTFTVPRDLKDTNTFILKLEYKIVAGVGTLLIIILFIALIFTLRRKRHRH
SKCDFHDNEIMLPNIGQKMYVDPMNYVDAETAMKEFANEIDPALLILERLIGGGEFGDVY
KGTLTLEDGKTMLVAAKTLKPNASKKSCRDFF
>Ever_5433 E. verrucosa
MSYVEISFEDLEFYECIGWGSFGSVYKALWKSKEREVAVKKVLRLDNEAEVLSLLSHRNI
IQFYGVLNSSPNYGLVTEYACLGSVYSYCSDSSKELQFEKIVMWATDISKGMNYLHYEAP
FKVIHRDLKSKNVVISSDMTGKICDFGTSKLATNTTQMSLSGTFPWMAPEVIQSLPVSEL
CDVFSYGVVLWEMLTREVPFKGMEGVQVAWFVVTKEERLTIPSTCPSPFANLMKQCWQSD
PKDRPSFKEIIPILKKMDGNGSLKDETNSFLRQKDEWRKEIDLTLEKLSRLEKNLNLKVK
ELEEREERLLQRERELEQQYVAKVVSKHDVNAWTEEDVFLWVEHIGIQVCNRTISNYAQH
FLDNNINGKRLLVLTIDDLKDLGIQSLGYRLEILDQISKLQSENDLMLHFPPLEKGEAVC
DDRCQKMVTLTLMFGNHCRIGRTEQDCKWKMYADVDVDGDSPYLSYIKDVTFNFSSSEPI
VLKNPPYVMEHWQKCEKQPSTVVECILNYTSDVKKPRCTKYIHHVQLQEGGATYQRTAEL
MLKQSAVGIARPVSPSLSNRSDVSLDSWSSHRSVSLPGNVTCTTSTPFLSAWERGKASEI
LFQSTGTRGLATSTLNTAAAVTAVRRTSSGSMSASSPKSSPSPSYARKPNVSSPQTPPSK
GKQTESPGSSGSSSPFEWTYVKSKRKESGHTSDKHPETGSMGHGRSGGSSNRRTGHKPGN
SGGITRGRGQNLRYGRSVSDNTGNRGSKQKDFDSYKKTASEGGGRRR*
>Ever_20101 E. verrucosa
TAIGATLFIFILIVIVIYRRKRMKKKTLDQEEGMLLNQCPQAYIDPNKYGNPEKAVRKFA
KEIDRREIKLEGLIGEGEFAKVHKGMLCRPNEPPVAVALKVLKPGSTAKNREDFLTEAAV
MGQFKDLNIVALEGVVTISHPVMIITEFMHNGALSNYLVQA>Tadh_12344
TYVDPADFGDMEEAIKRLAKKLPDAAIRIEDKIGEGQFGSVSRAVWTKDSGEKIDVAVKS
IKEDCTLKAKKDFLMEAAMIVQFEHRNIVRVEGVVLSKGRLKMIVMEYLANKSLDNYLRE

NNHKFTKLQLAGMASGVAAGMEYLSDKGFIHRDLAARNVLVGDMMLCKIADFGLSREVQQ
DDEEDQAQYTTEGGQIALRWTEPEAIISRGFTTASDIWSYGITLWEIFSYAARPYGEWGN
KKIMHYVNQGYRLAPPEKCPRCIYSLMLRCQLNDRSKRPTFSNIVQFFDNLFRLPDDLDM
MVYLLQIKFDSVADISISSWLEDLKLECYVRLFVNSNIVNVKNLLVLNEKRLTDMGITSL
NHIKKILKAIQS

Ephs in Placozoans

>Tadh_53296 T. adhearens
MKTFTAFFYLLLVHSSYTLAAKELLLDTNKYQAANLGWPGWKSCTSPPGHLGNWAYTKFL
NETVGKYTICYQGHHQRAWLISPYIPFGVAKKVYVNIEYSTKSNCSIRSKNCTSFLYFIG
LQFQENIGNQFCEKSELYSRIRDTLDLEEIIHAKESSLLADQKGRKVSLLGQHRKFAAFV
TVQENMNGMYLFLDGKGSCSIIQNVQVWYKVCHPKQIGNVYFPAAATEQTGTNVSYSNGS
CTGKSQLVDDKVQPIMSCFPNGTWSKPQNLQSCACKAGHELQNNICQRMSENIIVVIIVF
DLYLSYIFI
>Tadh_56933 T. adhearens
MGLTVFLLQSGYQTANRPPWNQWQNCPVNVNSNQGDSVKFELKQLGVVQYYYICNQVRNQ
RTWLISPYFGLNSAKEIYIKIRYTNTQVCPQNRFCASGLGIYASVLFDPIDGNICQTAVL
KIFKGLVESQHQDRPIRTIPQPYKYRDTSNVSPQQTPIPFLIAREWRLANMHITIEDRGT
CSEIEAVSVWYYACPLMNYPDAFFPRTVAPAAMNGSITVTGICSGSSTNLPYGLKTTAEC
QPNGKWANYVNNCPCNAGRERIFVSCQDCKFNYYKSLPSSGKCERCPNSMITIRKGAREC
VCLKNSILHISGECITMPVCKLFYLAVKNTSAILQFSRIEEQITRNMSFDLECFNCSLKD
INFRRYTVYTANITLDKQLMVTNLKPATSYALRVVPWIHIVDGKQKKTPCETVIFRTLDG
VPGPPTKVKTQSKKGNPLVHVSWKPPTLPNGAIRRYLIMFSLKEAFNATAEDLEKDVIVE
VDHTVLFYNISTLPVRTVFHVTIRAATVHGTFGPPSVTRNVTVYERDRTTSSVIGSVTGG
AVATGLGVFALKFGMMAKDGVSAGKIIAVVDKCNYT

Ephs in Ctenophores

>Mlei_02521a-PA M. leidyi
MILSVIKIFFLIYLIAADEVTLFDLGDSHQDEWYSTQKDEDDDLGSKKWGAYGNPFEFYV
CQISGIKQDNFLRTPFIFTHNATYIYVNIQYRLSPCTESKVGVQVNNCAQSFHLHMKEVQ
QDSAERCSGRPSSRGLRVQLLAPSKSCILTVLPSTPSPRQTIYTLTLTHNSCVDQSANQR
REREFFTNQEPVFGIVCMSGSVGNGLKQTTLPGGGVLKRLTRRSLDQSATSIPGDSQSDW
LPWRTCPLMRQGGFSIIHICKVFNVRRDYPYCIGEVTKRLFKYDNTAGRTGGHARKVAIG
QTTELCRVMKSAVGRSGEPPITLCKTSDSERGVKGYWYDTFQKSGCYCDRGFELSDDLCR
ECESGYYKKNSGNTEVCRKCPGNSVSIPGTAYKCECVFGFQRANATNLDEACI
>Mlei_35913a-PA M. leidyi
MLDHGKTGTLSTRSGSLQYNRRTYVPVPKYADPQDALCKMARTLDPSHITLERVCGQGQF
GDVWKGTFTVPGCKPKEIAAKILKDNSLLDQKREFLAEAAIMVQFEDPNVIELIGVVTRN
DKFMIITEYMKNGSLKEYLKEIKGLKSPVELLSMARGIVSGMKYLSEMNFIHRDLAARNI
LVDEYGVCKVSDFGLSRMLEEDSGYYKPHLGGKIPVRWTAPEAITHWTYTHSSDVYSMGV
VLWEIFTWGAEPWGNVSNDMVLKYLKDNIKLPKPSGCGDVVYNIMLSCWLDCTKRPTFAQ
LHSEFDKLIFLSSIDRNGGFPSMMRQYANSEERLMSVADWLQGMGMSQYESHFMNTGWDT
IDKVVLMEDESLHKIGITLAGHQKKIKTAIDFMKSSTQSSHHNLMMSSDGSLPRCNTLPH
THSSSPCLPAPRFGTMT
>Bero_3603 Beroe sp
MAEVESQGKPAAVVTVPSGVQEGEGSATSLDELQLDAPKPVRKETVPSSGNCVPDAFYWT
VTEVANWIEDIGLWQYKQCFTENFVDGRKLIMIDSNSLPRIGIQDFEHIQRICKEIRALL
GIEAPDSKRTIYLPPRVLRVD
>Baby_41769 B. abyssicola
SGHYGRVSVIVWTATTYEVTLLSMLETSSDWVSTQNDQDDNRAYNKWSTTGNPSEFYVCK
LEGPDQDNFIRSPFIELGGLKFVYVEIEYQLSSCTQSREDVERNNCEQSFHLHLKESDDA

EDPGLVDEYNFKIANEKYKAIVPGTFGTLVTATVPVALTEGKSGVFFAIRDTGACINIKN
FYVRYRLCPETTEDAVRYTNTVLNGVDIEVTGTCVDNSTHPENEPPIKYCRTENSGAWEK
GFQKSDCFCDRGFELRDEVCKECESGFYKRSQGNKSPGCIKCPDNSMSLPGFTYKCECLD
GFQRPNEQDIDQACIEPPDPPKKGENFNVSSKTKYTVTFKIPFLDPTLQYMIEVVKKTDY
DLVKVESNFVIYNTSVEITDLQPDQTYLITFRIANGSVESEPLNFVVETRGEVNKLMYYI
GAICALAGLVIVITLVVFIVVCKKRRRKKKFASFDVRESAMDWGKKSTLGTLGTNQSNSL
SFRKYVPLPKYADPQDALRTDRNLDRCHITLERVCGQGQFGQVWKGLYSLPGFKPKEIAV
KVLKEGSSMDQKREFMLEAAIMDQFEDPNVIELIGVVRSSDQFMIVTEYMRNGCLQNYLK
EIKGKKSVLELLSMARGIVSGMKYLADMNFIHRDLAARNILVDERGVCKVSDFGLSRMLE
EDSLFYKTSGQKIPVRWTAPEAIVHQKYTQKSDVYSMGVVLWEIFTFGAVPWGNATNDMV
LNYLKQRDKLQKPDSCPAVVYDIMMQCWQDDYTKRPTFGALHADFDKIILSSIDRKTPFP
MSTLESYTSSCSEEPMLTVSQWLHGLGMSQYESHFMNTGYERIDSVVLMDETDLQKIGIT
LAGHQKKIKAAIDSINAYTSGLHTYPNYTPDGSLTRVEIPRFNSLPGAHQSSPLLPLSQH
TGTMT*
>Patl_251921 P. atlantica
MQVDEYTYKVANDEYTSIIPRTTTNFETTKVPVKLTGRSKGVFFAIRDVGACVTLKDFKV
FAQYCPETVSNAVRLVPDATEREIEI*
>Patl_251869 P. atlantica
MLFSMMDSNRNLWNSTQEDNDPIRARYKWTTRGGYPFDFYVCIIEGNKQDNFLRTPFIPT
NEYSFVYVELKYRINSCADSREGVVRDNCAQAIHLHMKESDESGDAMQVDEYTYKVANDE
YTSIIPRTTTNFETTKVPVKLTGRSKGVFFAIRDVGACVTLKDFKVFAQYCPETVSNAVR
YPETQLNSTDKLVTGKCVSNATHPVGDPPKKICRVDEGSWQEELQQSDCFCDRGFELSAD
ICQECEPGFFKMRSGNKERCQPCPLQSTSLPGYTYKCECLEGYQRTDENRFELPCIAPPG
PPDQDKIKILREGKFEVTLTLPETSLDYQITINPSPRDNMMQLAPSGNYRVQDGTAILTD
LRPDTEYMVFVRVKNGSIESEPVPVIIRTAKEKPSIQEMLLGTWGIVIASGIVIFILVIA
VVLLCKRNKRQKIRHRRDHHINTGTASTLRSGTLSNTKYVPITSFAEHHDLLANARDIRR
SELDLAEECGKGHFGTVYRAIYTSPTGERREVAVKLAKESSNPDIIREFKSEASIMVQFN
DPHVIELVGVVYSDRFMIVTEFMKNGCLKNYLGEIRGMRTPAALLRMARGIVLGMKYLAS
MNFIHRDLAARNILVDEQDFCKVSDFGLSYILEENNYYKPTGNFPVRWTPVEAIRHRRYS
EKSDVYSMGVVLWEMFTFGEEPWGNLTNEQVLEKLHDQRKMERPHSCPDRVYKTMLKCWD
DDFMKRPTFTELQIEIESLMSTIDRNQVTIEIPVEEDDCLSVGNWLASMGLQQYQSHFMN
TGWDRLDGVRLMGEDDLQKIGIELAGHRKKIKSAIDTMNEYITPSWPDTLSSSRSLPRYN
TLPSPGAYFQPPQYRCGTLK*
>Vmul_37755 V. multiformis
WLRTPSIQTGGLGFVYVELTYWLDSCSNSKTGVTRDNCQQSFYMQFLEANKTDDELPPVI
NEYKYTKISEDSSIVPAALGTNIDETIPIRLSGYSKGVYLAFRDRGACIKILQVSVKYRF
CPEIVSQAARYKKTMLLGESVEVIGLCVPNSTSRSEDPPTKYCRDEQGWEKERITPTCTC
DPGFRLNTTHHCQSCSPGFYKADYGNAKECTRCPSNSMSMPSTATWCDCNKGFSRVDPSD
VHSGCIAPPAAPDKKQFKLVNQTSSSLAWEAPPLEGNLQYRLQVSKKDETGVFAIEVNKE
IEQNRFMLTDLEPGQEYLVSVRVANGSAEGDSIMIIEKTDSQLFGKYGTILLIVTCALVI
VILLGVICAVLHCKRRKSSRHAMLSECRDPMLDYAKTGTISSGTFSSCQNRKYIPISMYT
SEHDAIKNAMTIDKIFIKLTEKCGQGHFGDVWKGMHQEPGGAARLIAAKILREGATIKQK
RDFLIEAAIMSQFEDPNVIHLIGVVKSDPIMIVTEYMEKRKK
>Cast_22203 C. astericola
AITAGGFRFYVCRIEGMLQDNWLRSPFIRTDMLKLQYVYVKLKYSLRSCASGRDGVKRDN
CHQTFYLHYKESNKTDEGDAINEYDYNVANKESASIVPQVLGAPSETVVPIRLSGNSQGI
YLAIRDRGACITLTDVFVWYRYCPEIVSQAARYNATILAGTDVEVIGQCVENASTRSNDI
PTKYCWESKANGGWEKDRYSAPCLCNRGYEMQDNKLCVECRPSFYKRDRGNKEKCISCPP
NSISVPGTAYQCDCLSGYKRANVTNLDDGCIAPPAPPDKNRFELVTHTGNELGWDGPALK
DGLEYRLKVSKKSDGIVTVIEVNKEISNRSFKLTDLEPGQEYIVTLTIGFGSVEGEPIIF
IEKTRVQIFVKYGILLMVVIGVLVVALIGILGFYCKKEKST
>Edun_188839 E. dunlapae
SFYYLNDQHSFLRTPFIPTRTTDHLYVELEYGLKPCTDANEDVTRDNCQQFFYLHALEVS

RRTAT*

Ephs in Sponges

>Ccan_99557 C. candelabrum
MGVITSWRYNGRLCDFSFHICGSEVGAQDNWLVSPYIPLAIAKRVTVSVEFTMYSCNPKL
VHCRDSFRIYGLQTDNPNITASMINETSFGLLVSLAGNVRDGMTHNYGEVTLDVKKPYLF
VAFRDEGACMTLFVVKVAYNV
>Cpro_59066.22 C. prolifera
TSYSYHYWLRSIIMDALKISLLLVLLTCWAGLAWGQLTPGEAPGDFDPTCREGAMRAPMH
IFPESWELMLDSFDYSNLYDPNGLLPVWVAVSFDGDSGTDADSNWNQILGGPCTQTIRTS
TDTSPTLPQDNWLFTQYIRYNAAVEIFFNVTFEFTCNDCVRYVTLYRFETDGVQEESERT
NRDNYQPFFGTEEDSRLETQGPSGESVATTLRFPRPSSGRAGFYLAFRDEGTRVEIDRVI
LYYRVARGFSEPFISCPDVPLPEVGERSSLSCECIGETMGVESLERSCDSSGVCTDEGLA
CECSPGFEFNSTFGLCTPCPMEMYKDGIANDSCLPCPELSEARNIGTAVCECIPPNSRAG
NEGAEVACRDRRLSTAAAVAITFFVTFIIVGVAAFIGGGLLAYCCRERIGKVV
>Cpro_75438.40 C. prolifera
TTLFRSDSLFHPIRISTRTSLICAISMTTILQVTLLLALSVSTAWTQAPYDYDPDCRSGD
NMAHRDRFSDNWRILIDTYDYPNLYHPFSFPAPVWKSVDFDFPSGYNPTDGWFQIIGGTC
TWALNTDTYRSPQEQHNWLFSQFISYEGANEIYLDVTFRLSSCRDNPRCIHDYVDVFNFP
TDGIVSDDQRMDRDNYAHMYRLQQPPTGTQVSISERFPRSDNEGFYMGFEDRGTAGQVQR
VIVYYTVCPSRQNELVVYPEVGTPPVGGPDMTFLAECVPFSHGVTSLEVIAFSGNSTCRD
VAEGGARCECDDGHEISGDRKSCSLCPVGTYRSAGTTSTCVTCPQNSNAEFEGASICICD
PDFFRSPEDGPDAPCTRAPGPVDNLVVTQTDDSSVELEWNKPIKTGGRDDLYYQLTVQET
NNGFTTVHETEIVSIADTIRYSISGLQSFTTYQITVVSHNGVSDQEDPALEQLRTERVLA
TTTEGVPGVPLVSPLCTVVVWSAPLEMAGDVVGYQARLRQSDDAVIDLDADANFFITEDA
DRDANVVVEMRAHNSQFYGPWSSPVPLDCAPAVSCPTTTPCVCDCAATTPLATTTAAPTT
TPPPTTTAAPSTPAPTTSSPSPACKPPIFTLKGQEVKNGSLVPLLDIGTDDDALMMETEL
EECCRDQRMGECYYPDGTLVGIANDQQTFYRNRAREQFIRLNRRSGTAFGDHPTGLYRCE
LPDSCGTLTSLYVRIE
>Cpro_78554.29 C. prolifera
TVDVNLNGNADGLYLAIRDEATCIVITRMLVFYSVCPGGAVDFVMRPETLAPIVMRNPIP
LKVDAECVAGASPENGEGAILNCNQGGTWTVVPGAGCQCNPGFMAAANGRSCTGCPAGEY
LSVANAECRMCPDNSEGTVSGLTVCPCVDGYYRAEGEEDRKCTRPPSEPTNVRVDSRTSD
SI
>Cpro_78852.34 C. prolifera
STATIWRSTVQPLRGMWLLNWCAILCLLPWQVWTQAPGDFNEDCRTGQNEDLHIFPDTLE
LLLDSYDYSNLINMQGLLPAWQTRDYDASDPSIGWQQVLGGPCTQTPSVNTQTLNFENEQ
DNWIFTQYVSYREAVEVIFNVTYNLAQCIAAPACTTQYFTLYRFDTEMLASNSRDIVDTE
NYVPLFGTLENSRLEPTSTSSSSSFNQILYLNRPSATGFYLGIQDTGSAGSLGRILVHYL
VARGRTEDLLTCPDVPLPPQGGTATNTEQCSCEENASPVTSLSRTCTASGVCNEDQVCAC
SEGYELSGGNCVVCVENTYKAGVSNNSCQACPANSESRGEGNTICDCIPPNTRNPANPDD
PCTTPPSSPRLILGGGGAPRVSDNRTSISVCWLPSLDSGGIPETLHYNIYLYDTAVNVSL
FEKVNSEPIIDATMDNMTRICYEVPINDPETSYGVVVVASNGATVDPDNFTDVAGVQDRF
VVFFVALGDLSDPTTVESPTCVTSIVVTLFGTLIPTAVITAVVVFLVVWCCLKNKDVSSA
SSSYPSKPAPSQVKYHASGSYDNKNEYIDSGVSSPVTATSPVPVKPRPPVPQARPAPAGP
GRKPPPRPPNKPAQA
>Cpro_79320.39 C. prolifera
YYRAEGEDDRECTRPPSEPTNVRVDSRTSDSITLVWDEPEDLGGRTDTFYILFYQEVGTD
EVVMAKRVNGTTGTITGLRPGVEYMVFVSAHNGVSQMVVEEEVPMIPLGVSTLAIDIPIV
VGAVVAAFAVILLVFIAIIIVYIMYRVYYSKQMQSRYDMNGHEAIPIPVSKHRTYVDPTT
YASPDEAVEEFAKEIEPKTLRLDSEIGAGEFGTVFRGIWKEQPKKQVAVAIKTLKPGSSG
RMRTDFLNEASIMGQFHHDHVIKLYGVVTKVDPAMIVMEFMDNGSLYSHLRANDEKLGNQ

RLLKMTRCVCSGMEYLATVGFVHRDLAARNVLVARDEVCKVADFGLSREVIDDEYNVQKG
GKIPVRWTAPEAIYRRKFTSSSDVWSFGVLMWEVMTYAQHPYDEWDNQTVLDQLEGGYRL
EIPHGCPDAVYEIMFDCWNKELSERPSFTAILKRLDLLLNTESPPKRPPKKLRSTVNSAT
NPLQCKSVEDWLESVKMERYVPLFQQNGINSVNQLVHLTEPDLREMGITLAGHLHRLTQS
IESGYVELKRNSTFAT
>Cpro_80288.25 C. prolifera
LPVKMNCGRTVGVVFCVAILVSVTSAGAPGDEDSFCTNELPLSGTTPPTNLKILLDSYNY
VNLLPPQDGAVWQAVDNDNNNGAGRGTEWTQNSNIPCRQAVTVCGFEVGPQDNWLITQYI
STVVNDTRLAQVSVIVEYELVGCTSLQCQRTFVVNAYETSTEDNSIAADINNTDYRLVDR
IATDDDTGQTRQNRTVDLNLNANADGIYLAIRDETTCIVITRMLVFYSVCPGGAVDFVMR
PETLAPIVMRNPIPLKVDAECVAGASPENGEGAILNCNQGGTWTVVPGAWCQCNPGLVAA
ENGRSCIGLIKVLHTFSVVKCILNCYRLSSWRIFVCG
>Cpro_80288.49 C. prolifera
LLLSSFASPPDQVYVAMFSATHGVGITLCVSVLVSVVSAGAPGDEDSFCTNEFPSSAIPP
NSKILLDSYNYINLLPPQDNAVWQEVDNDNNNEAGRGTEWSQNSNIPCRQAVTVCGFEVG
PQDNWLITQYISTVVNDTRLAQVSVIVEYELVGCTSLQCQRTFVVNAYETSTEDNSIAAD
INNTDYRLVDRIATDDDTGQTRQNRTVDLNLNANADGIYLAIRDETTCIVITRMLVFYSV
CPGGAVDFVMRPETLAPIVMRNPIPLKVDAECVAGASPENGEGAILNCNQGGTWTVVPGA
GCQCNTGFMAAANGRSCTGCPAGEYLSVANAECRMCPDNSEGTVSGLTVCPCVDGYYRAE
GEEDRECTRTSPDVADISYWYQDSEDSSFWAVRVNVTTLTITDLLPGREYTLTVKAEDGV
SFVAGFSNDTVGTTVVLGSVPTQPTLCGGGPLTLTSESGSIETLNWPSSAYPLNTNCMWD
IVCPSGNEININFDANFRIAGRMPACDRDRLYIFDCNEAITYGPFCHLTAPQPITSSCNA
IKVRFESGNDRGSSRTGFRMHYMCVPPTNTSAPPSTLPPQCGGGLQVRDEGSGSIETLGY
DQDPYPINTNCLWQIQCQASQKVHITFSESFKIAGAMPDCAKDQLEIFDCDGNVLYGPFC
HLTAPQPIMSQCAAVDVQFHAGDERGVKRTGFRLNYECV
>Cnuc_32701 C. nucula
MERTCLRNVFVLLLLVSFQRGRSQAPGDENEFCVNETERNPPSYREPNRDILFDSYNYPA
LVESVIDVTNPSVHNVPPVFTRYSLEGKLNWQQQRGLGNCRVEVFNCKVAGDGSTGSNDE
EDNWMVTQHVKVSSTVREPRVFLRFTLTRCPSNAGTTCRDSFALYIVPSNVSSGISQDDV
TMFSSTLSPVRITGSDNEATSKYHTITRGSLTGYEGFRIALQNENACVTIHRIQVLYWIC
PAGRQGLAYVETAGELNHVGTFTCVGNSSLIDPAKNEGLCKVSTTGDEESADWVTLGGGA
VSLSQACVCHPGFGSIQINGEDQCQECPPGMFKEEVANV
>Cnuc_32703 C. nucula
VFNCKVASGGTRNSNEEEDNWVVTQHVNVSSSVQEPLVFLRFTLTSCPSDAGTTCRDSFA
LYIVPSNVSSSISQDDVMTFSSTLSPVRITGVDGTPTSKYHTITRGSLTGYEGFRIALRN
ENACVTIHRIQVLYWTCPAGRQGLAYVETPGEPNNVGTFTCVGNSSLIDPAKNEGLCKVS
TTGDEESADWVTLGGGAVSLSQACGCHPGFGSIRRNGEDQCQECPPGMFKEEVANVPCSP
CPERSFTNSTGSVSCQCDEGYYRGMNETASLPCALPPSAPKNLTVVGTGSRRVTLSWNRP
DFSGTRTPLFYTISFRAEGDTQWTTGPPVSNSSPHIVNYTYSGLSPHVKYQVRVSADSSA
TDSIPGSLIGERSAMESFMTEEDVPGQGSPPMLNVPLFTTTIVVTLVEPDMPNGEVVQYN
IRWYNVEEGVNNSQVVRVDGQSMQYSLEGLSPGRYVVQVQIVNALGVGDYSEPAEIEVVG
VPAWFII
>Cnuc_9016 C. nucula
VPAWFIIAIIGGVFGAGVLVAVSVVVICLIRICVRKRKERESRRLSVDTEGAALVSFGKN
GVHLPEKKKKIYVDPRNYASTDAALKDFAKEIPPHEVRLEKVIGGGEFGDVCQGWWHKIN
EMKPVKVAVKTLKSGSPNKARQDFLAEATIMGQFDDPNVIFLYGVVSKSEPVMIVMEYMS
NGSLEHYLKKNDGLLNVHRLVKMAVGVASGMKYLAGIGFVHRDLAARNVLVDDKENCKIS
DFGLSRETEENVYNVKTGGKIPVRWTPPEAIHFRKFSEKSDVWSFGVLLWEVMSYGCQPY
ADWGNQQVLREVDKGYRLPCPEDCPDCVYYVMMKCWDKEHRIRPVFAEVKDELEDLYKND
FVGRTKPARRREPVLGPLSYSSIPEWLEAIKMEQYIDNFVDGGLSLKECCELSQDDLPTL
GITIIGHQNKIMTSINCESRRGSMANGTD*
>Avas_56846 A. vastus
KYSMIADTKLNLMKYYGVYLSILMMSQHFIQPSNGQSCNTMLPVHSQTILDSTTYHNINT

AGQILPFLIPGWIDTDVDGIIYNTPERLGWKPVLFTGSCGPAYLSCPKASIIVDSSQEMN
WIATDYYETKGNVIHIRVMCFALTGTGFATDCRDAFQIFYYESDQDITITNSLLSQFLPA
DSNKTVSRVVSAEFTKSKKGFYIGFFNNIPCPELNEFLFYYFECPATNISDLYSLS
>Avas_56851 A. vastus
EDGILYNTPERFRWQALLNTLSCGPEYRSCLQGSVNIVDSSQERNWVVTSYYETEGNVIH
IRVMCFALTGTGFATDCRDAFQIFYYESDQDITITNSLLSQFLPADSNKTVSRVVSAEFT
KSKKGFYIGFFNNIPCPELNEFLFYYFECPATNISDLYSLS
>Avas_56854 A. vastus
KTKYSMNTGMKLMEYLAVYLSILMMLSQHLIQPSNGQSCNTMLPANSQTILDSNTYHSIN
MAGQTHPFDIPGWISFDLDGTIYNAIERLGWISNLNTGSCGPEYRSCLQGTFNNSDSSLE
MNWIVTDFYETEGNVIQIRVLCTLLNGTGFATDCRDAFQIFYYESDQDITITNSLLSQFL
PADSNKTVSRVVSAEFTKSKKGFYIGFFNNIPCPELNEFLFYYFECPATNISDLYSLS
>Avas_63694 A. vastus
NKTKYRMLASVFLSHFLFTLFLLLRETSAAPTDCSTAVPSNSVVIFNSRNSNFINMNDMT
QPLAIPDWTLFNADQIMYETGLGNVTLEWRIAVLIGQVSDPCTPEYSVCTSSSQLLNTSL
EQNWILTNYFLTEAINIQIRIQCSSNIPFTIDCREALRAYYLDSDQNLTNVIPLLDQFQP
ADYSTNNIPSNGSGVINFSFNKTQRGFFVGFLNDRPCVTITSILIFYYIQCPTVPPASTS
LFSLPATLAPNTTQEPLLISPSCPGDLDLPSSVRAECYANGTWRLPPARERCECPVNTFQ
TQTESCSPCPDRSSSLGLRGARNCSCEAGWYRGEGEGVSLSCGQSPSVVQDLRIDLSSGV
LAVWNLPSQLGNRDADSLSYRVCYRQSLSSGVDTCVEVAETRYQLTGITPSFEYVLAVTS
LNPVSTASGVYNTFNATFLSSFPNLTMPVYLNGYLNWSYSLYAREQYQFQIRYQSSSGIV
MTNVTPSSCRLISGSNRVCSIMISDLNVQEPIVISVLNPSGPLSDPISRTYDLGTKPRQL
TILDYIFYVIIPVSLFVFLAILFLTLTVCIVVVCRKRRAFKKHFNNEMESDIPLQAQLYQ
DPSNYADLNNAVRALAKEIDPNDIERESMVGDGEFGDVWKGSLMRHDRKVPVALKFLKPS
PTAKNKDDFFKEASIMGQFCHPNVIFLYGVTLKKPIMIITPYMDNGSLDKYLVNNVYSLA
LGDQIALCCGVSRGMVYLTRKGYIHRDIAARNVLVDKDMTPKITDFGLSRFSEDNLYKVK
TGGKIPVRWTAPEAIVYRKFNSASDVWSFGILMWEVMSFGQLPYAAANNIKLFDQIQDGM
RLESPDRCPSVVYNLMIKCWDEAPDNRPSFEQLEQALVQILEINKVRPTSFNLKLSHQNP
LNYVTLESWLTSLKLDRYILNFTNNGYTQLAHVWHLCEQDLFTIGIIPVGHRNKIMNSIY
KANNQLCFTYSIPV*
>Avas_82832 A. vastus
QNKTKYRQMLAFNSFLSFFFINILWIHLTTCANRTIYSSIGQSPRFPIEGWIDIHDDIVY
VNNDKIQWIFNTNSGHLGPHYSVCQGSTSTPVDTDLERNWLISNYYKSEADTIYFEIQCI
TFSMFPVPDCRDAFTIFFLQSDNPIDLNNSISQFSSIESTFLSGSTSIILANISLNMNGF
YIGFLNQGLCVELESFIFYYNICPPLTISEMSSVPETPVPRLSQQPTQVNISCPPGSLSQ
NRAECYSNSSWVLYPCECVAGWRQLSNLSCAACPVNSYKEFVGNERECVTCPSQSSTDGN
TGSVMCECGAGFYRGEGESVSMECGRSPSAVRNIRVERYSVERAMLRVLWDEPVEVFNRS
VSYNLSLYTRESDRDVLRWRGISVDLWHNLSESGLEESREYVLVVTPLNNLVELSGMENS
ENISFVSTFPLLSNFSLVGNNSIFEFVYELKGGVSELSFELNYTSNGSVMMQAAVNGCSL
VSGDTYLCRVTVNNFNKSENFVLTLITFSSVETDAFSDVFNLMMIETTTLQTSSSNVTSI
RTNTTNSPNQNSPVSDLNIIVYIIPIVLILLIVVIGFAVGICICLCLKKRRRNPTVYKKE
HRSQNFPLEKLYQDPGLYNDLNTAVRRFAKDIDHQDLEFGDSIGEGEFADVCRGLLKKQE
QQITVAIKILKPSASKKNKEDFFREASIMGQFKHPNVIFLYGVTLIKPIVIVTPFMENGS
LDDFVRRNDASLTAIQQATICMGVSCGMAYFTKIGFVHRDLASRNVLIDRDWTPKITDFG
LSRETEEEFYNVKSGGKIPIRWTAPEAIAYRKFNESSDVWSFGVLMWEVMSFGRLPYSGI
ENFNVLAEVQAGYRLERPDFCPEPMYQHILSCWKTDYNDRPTFNELKNRLTVLIDNNFGN
KNKRRSTRLSMVIQNLSIEEWLESLKLPRYIPIFQDAGYCYITQVYSISNETLMSIGVIP
LSHRHKILKSLSDMYKAVQHQVGQL*
>Avas_82838 A. vastus
MFIIIKVFSSPKLICLILFWSIIPQVYLQSCLTFPSNSRVILNSAILPDVAAGISGFQVF
DVDGMTYPYTVNELSGFESFMCRRSNANVINCGPAYQCCFTIVQVEVDPSIENNWVISNY
YQTEAEVIQIQISCKQLQASPVPDCRSAFYIYYYESDTSVSNISTFLPQFSPATLSINQT
FNDRLVYATFNKEKGGFYIGFFNNGTCSEINTFQIYYNICPSLTTNEMSSVPETPVPRSS

QQPTQVNISCPPDSLSQNRAECYSNSSWVLYPCECVAGWRQLSNLSCAACPVNSYKEFVG
NERECVTCPSQSSTDGNTGSVMCECGAGFYRGEGESVSMECGRSPSAVRNIRVERYSVER
AMLRVLWDEPVEVFNRSVSYNLSLYTRESDRDVLRWRGISVDLWHNLSESGLEESREYVL
VVTPLNNLVELSGMENSENISFVSTFPLLSNFSLVGNNSIFEFVYELKGGVSELSFELNY
TSNGSVMMQAAVNGCSLVSGDTYLCRVTVNNFNKSENFVLTLITFSSVETDAFSDVFNLM
MIETTTLQTSSSNVTSIRTNTTNSPNQNSPVSDLNIIVYIIPIVLILLIVVIGFAVGICI
CLCLKKRRRNPTVYKKEHRSQNFPLEKLYQDPGLYNDLNTAVRRFAKDIDHQDLEFGDSI
GEGEFADVCRGLLKKQEQQITVAIKILKPSASKKNKEDFFREASIMGQFKHPNVIFLYGV
TLIKPIVIVTPFMENGSLDDFVRRNDASLTAIQQATICMGVSCGMAYFTKIGFVHRDLAS
RNVLIDRDWTPKITDFGLSRETEEEFYNVKSGGKIPIRWTAPEAIAYRKFNESSDVWSFG
VLMWEVMSFGRLPYSGIENFNVLAEVQAGYRLERPDFCPEPMYQHILSCWKTDYNDRPTF
NELKNRLTVLIDNNFGNKNKRRSTRLSMVIQNLSIEEWLESLKLPRYIPIFQDAGYCYIT
QVYSISNETLMSIGVIPLSHRHKILKSLSDMYKAVQHQVGQL*
>Avas_85009 A. vastus
MLTITNCLSPKLICLVLFWSIIPQVYLQSCLTFPSNSRVILNSAILPDVAAGISGFQVFD
VDGMTYPYTVNELSGFESFMCRRSNANVINCGPAYQCCFTIVQVEVDPSIENNWVISNYY
QTEAEVIQIQISCKQLQASPVPDCRSAFYIYYYESDTSVSNISTFLPQFSPATLSINQTF
NDRLVYATFNKEKGGFYIGFFNNGTCSEINTFQIYYNICPSLTTNEMSSVPETPVPRSSQ
QPTQVNISCPPDSLSQNRAECYSNSSWVLYPCECVAGWRQLSNLSCAACPVNSYKEFVGN
ERECVTCPSQSSTDGNTGSVMCECGAGFYRGEGESVSMECGRSPSAVRNIRVERYSVERA
MLRVLWDEPVEVFNRSVSYNLSLYTRESDRDVLRWRGISVDLWHNLSESGLEESREYVLV
VTPLNNLVELSGMENSENISFVSTFPLLSNFSLVGNNSIFEFVYELKGGVSELSFELNYT
SIGGVMMQVAVNGCSLVSGDKYKCNVTVINFDAKIDFVLILNTFSSTEVGARISITRNFT
MPDTTRLSTSTFNSSTFFTTPIPSDSAPALSMSTVFYLILASSAVALAIVVTVTIICFIL
CCYLIKKPVKKLYSAIKDETRPLDIPFSPTYQEYTDPSLFGDFREAVRHFAKEVNIEDIT
ISKVIGNGEFGDVCAGTLNQEGQLISVALKTLKPNSSEKSKSDFFLEASTMGQFEHENII
PLLGVTLNAPIMIITPFMANLSMDKFLRLKDRKLSVIQLGNLMLGVARGMVYLSSRSYIH
RDLAARNILIDHDLTPKIADFGLSRVIEEDFYTMHRRGKVAIRWTALESILYYRFNTASD
VWSFGILAWEIMSFGAMPYEGMDILNLVEKLEAGYRMPKPESCPQLLYQLMEECWQESAE
KRPSFEQIHGNLQDMIESHFGARRYARLSKTFQDDPLSFSCVGDWLDSLTMSRYEDIFNE
NGFTSLNPVWGLNEQDLLRIGIIPVNHRNKIMTSIRKANRVLGRTFKEYSTTFI*
>Avas_85019 A. vastus
MLTITNCLSPKLICLVLFWSIIPQVYLQSCLTFPSNSRVILNSAILPDVAAGISGFQVFD
VDGMTYPYTVNELSGFESFMCRRSNANVINCGPAYQCCFTIVQVEVDPSIENNWVISNYY
QTEAEVIQIQISCKQLQASPVPDCRSAFYIYYYESDTSVSNISTFLPQFSPATLSINQTF
NDRLVYATFNKEKGGFYIGFFNNGTCSEINTFQIYYNICPSLTTNEMSSVPETPVPRSSQ
QPTQVNISCPPDSLSQNRAECYSNSSWVLYPCECVAGWRQLSNLSCAACPVNSYKEFVGN
ERECVTCPSQSSTDGNTGSVMCECGAGFYRGEGESVSMECGRSPSAVRNIRVERYSVERA
MLRVLWDEPVEVFNRSVSYNLSLYTRESDRDVLRWRGISVDLWHNLSESGLEESREYVLV
VTPLNNLVELSGMENSENISFVSTFPLLSNFSLVGNNSIFEFVYELKGGVSELSFELNYT
SNGSVMMQAAVNGCSLVSGDTYLCRVTVNNFNKSENFVLTLITSSVETNAFSEEFNLLMI
ENATPSNTMTSRTNSTINTTQPTTNSSICSNTLSQLLPILLKVSTFTSFGMFLIIISLAL
IMIALLICTRKNLNKSKKILPYPRN*
>Avas_85324 A. vastus
QWSYELYGKRDYVFEVNYTTTVSSISNRIVSQKLNSDECHCSTNALCTCILSTPNLNSTH
NVTFLLLVNVSRNLTKTNLNFAHSFTPSSTLSPSVTPSVAQTSAPSIMFIIIGVVGIIIV
ILIIIFIILIIGFMIRRRKSVRSKFSHQKFDNTGLKGTNPIDSIPEKIYQDPNTMYQDLN
EAVRALTKELNPSNIKIERVIGEGEFGEVCKGTLQVNYRTVPVAIKTLKPNCSEKSKQDF
FKEASAMAQFSHENVIYLYGVTLTKPIMIVTPFMENGSLDKFLTCNKGNMLVIDLGRICH
GVARGMCYLSRIGFVHRDLAARNVLIGSDLTAKIADFGLSRETEEDIYNVKTGGKIPVRW
TAPEAILFRKFNMASDVWSFGVLMWEVMTSGDVPYVDKDNFVLLQDIQQGYRLEQPNDCP
DQLYQLMLKCWDAVPELRPTFSDLEAEISAMVLYNFLPKPRGRPSRSTVQSPLNFTSVDE
WLKHLNLEKYSDNFKSHGYTMISSVWHMSEHDIMAIDILPAGHRNKIMKSIHEANNKLSR

TYSVRV*
>Avas_93988 A. vastus
MLYEYKLMRRELLIQLLTCISLSLIQFSNGQQCNTMLPANSRTILDSNTQHTINMAGQRI
PFAIPGWTRIDVDGIIYTPTDTLEWRPPALTTGQCGPEYIACIQGANTAVDSTREMNWLV
SRYYETEAKVIYIRIKCAALSGSGFATDCRDAFQIFYYQSDQATTINSSLLSSQFLPAES
NKTVSGVVSTEFTKSKEGFYIGFFNNIPCPALKELLFYYFECPATNTSDLYSLPATLAPN
STEVFVRVNISCPTNSLHRDPLRLVAECYWDGTWRLPAESVCECEAGYYLSVSNANSSMP
CVMCRVDTYKGETGNSEECSVCPFRSSTRGMEGSSECLCDEDWYRGEGESVSESCGQSPS
AVVNLRVERRDRIVSRVVWDEPLFLGNRSENELRYNVSYYPSLTPSSEESVGELREIELV
LSSGDIADSTEYVFVVTSWNSVSFVSGVFNRANVTVLSSFPTLLSISYNESSEYLQWSYE
LYGEREYVFEISYTNISNGSVSLTLNSGECGCSNNFLCSCIVATPDLDSIQDVSFRLMVN
VSGILTNTSLSTLISMLIRTATSTSNPITTSNTTSTISTFPSTFSLPILFGIIGVSILVI
LIITLSICICLIIFLTRKVSVRSKSLTQGLSGTALVENPKTNSAHTNAVIDVTYQDPRLY
LDLNKAIRSLTKELEHCNIKPHCVIGSGEFGEVCKGTLRKNDREVTVAIKTLKPNSTEKN
KEDFFREASAMGQFTHENVIYLYGVTLTKPIMIITPYMENGSLDKFLVNKKGSLVLNELA
NICLGVARGMNYLSKRGFVHRDLAARNILIDSDLTAKIADFGLSRETEENFYNVKSGGKI
PVRWTAPEAILYRKFNMASDVWSYGVLMWEVMSFGNIPYGDMDNYKLLEEIQQGFRLPQP
EICPYSLYTLMARCWDTNQDNRPTFIDLEREIHLMMKNNFHPKPRGRVSRQVVEPASPLN
FTSVDDWLTSLKMERYSDNFKANSFTQISSIWHMTEHDLMAIDVIAIGHRNKMMKSIHTA
NNRLSRTYSVRV*
>Avas_93993 A. vastus
AVNSSLEINWLVSRYYETEAKVIYIRIKCAVFSGSGFATDCRDAFQIFYYQSDQARTITN
SRLSQFLPAESNKTVSEAVGAEFTKSKQGLYIGFFN
>Cele_137847 C. elegans
MYCLVFLVVVLQAPVGDAGGGLCNTPGARVLLDSYAFPALLPSPWTTTMPGGWNINSSTR
CFQSVAVCDASAGQNNLLVTDFVVLQPPELQFINVLVEYVCPSFSDTCNTDFSLHIYETS
IENRTESLNLDNYIEIVPVVLNNSEITRLLTMRTGFYLAFQDTSSCVEITRVRVLDATCP
SVVNAFVGYPVASSGTSVNGNCVEGAVLNTGSLTTQCLSNGSFDFGSGSCHCMGGWEQSN
DSVCSECQQGFFKRDDGNSMCEQCPVNSGNEGTGNTDCDCDMGYRDAAVRCSECDNNFFM
NSGMMCFSCPDGSTSPGGEATACTCNSALAIENGDTLTMNQPCNSCRDNYFRSSTGCVSC
PAKSSRSFGDEDSVCFCDSNGATASNAPNTTGLDCVGCAAGFFMGDSGMCTPCPSNSMSI
NHDDISCTCNEGTTLPGDAGDMTTSEPCESCDAGFYRADGVCIDCPANSERLNSADPEQL
CTCISIDYMRRVTAGFEGNCL
>Cele_48040 C. elegans
MHRCTECQRSVTLYVYEAFNDDFPDQAREASNYHQVNIVAPDESFGTNSQIQTREANLYS
EAEGFYLAIRDLSTCISVERVVVSYSVCPGGTED
>Cele_75907 C. elegans
TQNQTREVDFDTDAEGFYIALRDETTCIVVQRLIVFYNICPGGAEDFVMRPETIAPRIER
ITRPLEVTAQCVEGASAVGGNEVRLNCNQGGVWSAISGSGCTCDPGFSASTDRRSC
>Cele_85965 C. elegans
KNLRRAMMSETVKATLLTISLLGILQGIDALLGAEAPGDHNGECRIGRNSVLHNFPDDWE
ILVDTYDYRNLFGVSLDGSAPVWSARSFDGDNLQWEQSFGGICTQSIFACTFGGEQQKNW
VFSQFINYQDANELFFEVNFRFSRCRDTPTCSNDFATMYRYDVNDPAPERENPEKYRPLF
GDEESSRLQQLPAPIRSVEKNLSLLRPEPRTRGFYIGLQDTGTCGQVERIIVYYTVCRAR
ENELVRYPKVGTPPRDGPDMVFS
>Cele_124391 C. elegans
VKPLTTCSTMSSTTPQNNWVFTQYIRYRDAVEVIFNITHNFRRCTNFPTCDITFITLYKY
DTDGIVDEGMRTNIDNYQPLFGTEEASRLDGAGSAVDVTEVLRFSPTAGREGFYLGFRDT
GTCVSIGRIFLYYMVAVGRTEAFLQCPDVPLPSASGQTLSTLTCTCVNGTTGVGSLERSC
DRNGMCNEDQICRCNAGFGYNSTLETCTSE*
>Cele_133760 C. elegans
MFKVSLHDILLQWTQKTIIPCRQTVSVCGFELGPQNNWLITQYISRRVGDVLLPQVSIQV
VFEQRGCNVEL

>Hamb_7684 H. amboinensis
MYFHVLVLLGYSFISLNNGQSTAPNDTGSFCLDMSALPHYPPTVKRLMDSWSYQRLLSAN
NSGPWITLDEDKDNITWAAVIGETCREKVNIREPQNLPQSNWVITQYINKTILPQHNTAP
TNIYINISYILDSSCSEVMCRTNFQLLYHLANMSNANMSTHTPGYTLLANVSSLDDTSLH
IKTFSFVMDPQNVGFYTAVRDIGSNITITRLFVYRLIGPEMQIGLTLFPETPAPTTECVN
ITGTCVANANTTSGRPPVLTLDPSGSWTYGEECFCIVGHQPRMIEGSKQCQACSPSTYKS
TVSNTDCISCPSNSRSTAHGVSICNCNSNYYRAPTDSADDPCTAPPSAPRSIIVSSRSNV
SATIRWNTPSNSGGRDDVFYNVFYLSSRNFSNEVRANSAPVNQTYFNFMSLEPKTFYSVR
LVAENGVSDQSPNTNLRQVRTSFVTRDGVPRQVSGVQVVSGTFVEWNRLMDNDRGTITMY
QVQLYSNPSNNGRLIDIANTASVYRVNITTDFPTVGQPVFVIVRAFTELGAGDWSEPIQF
AAINPLAPTSVVPSSTVFTASATTTQSTSSAGTNSAGGGTVTPPTATPSTTPTNGSASRP
YISIVAVLCGVVLILILVSAGALGYFAFRKLRIKYFEPSPRDQTDSGTSPLVRYEVLPEA
TGESEEDERVQINPIAINN*
>Hpop_15474 H. populiferum
RPLSPFFSTNGNVIHVQLSCSNPVGTIPSNLDCTSAATVMYYETDTALASIQISTLNDFI
PATVEQLSAVATILASFTTTKQGFYLAFNNSGLCITIEDILIYYFQCSAMNGYNSIGSLP
NTSAPAMTQQPLQVQFTCPNNSYMNASLTAECNANGSWVIPEASPCHCHAGYRVQYQSSV
HSCQVCPVDTYKHTIGNTDCTTCPANSHTDGVTGEITCVCRALDVSHSVKIVIVPIIILT
CILIFMIIVLCYFILYQKRKK
>Ifas_26594 I. fasciculata
MYRVASIVVLFVSFFAVNSAQQAPGDEDDLCLLVSYRPSVVNQEILMWSFNYRNLLEHQG
ARWIVLDEDSNNDGSSTIAHTTWTESGSSCTRVASVCGFGHGPQDNWMITQFINNTLRQE
DVYIRVTFSINSDDCITKCQTA
>Ifas_36548 I. fasciculata
IGLSTTWTQTRSSCSRVTGVCGFEEGPQDNWMITQFINNTRRRSELYIEVVFSLNSQCTS
TCQTTMDMHVLQTNISNQSFVRDVSVFDPP
>Ifas_39595 I. fasciculata
HLRYAKTCPPVPECYIQSTVSVFENSSRFVLTNNIRDGRTLITRTNRVSADLTTSGLYVA
FRDRGACFGISEVLVYYPVCDSISLDFGASFLTTQFPGGNSVGSCFTNMAIDLNSINDSF
EATCTLSNNESLTNWTINEG
>Ifas_43965 I. fasciculata
GQNLIQRVQTLSANLDTTGFYVALRDTGTCTGVSEVTVFYPVCDGISLDFGANFAETRFP
GETSSGTCFENMSINVDTPNEPFDATCILK
>Ifas_9729 I. fasciculata
EIGFVHRVSLINMIVMEYMENKSLDFYLRKNDDLLGPKRLVTMAQGVAAGMQYFGEIGFV
HRDLAARNVLVSKEEVCKIADFGLSRETVDNAYDVKTGGKIPIRWTAPEAIQYRKFTTSS
DVWSYGVLLWEVMSYGQQPYWDWSNHKVLDEVEAGYRLEIPKDCPQTVYQVMLDCWNLDH
RLRPKFDEIGKELETLLQNNFRSSRSIANNQIIQSSVCPLDYDSIDKWLNAIKMDRYIEN
FRQSGLLQPRDCITLTHTTLMEMGITLSGHQHKILSSIQTANSRLQREPSYKI*
>Ifas_20154 I. fasciculata
SGGPSGCTCLPGYEFTSRATTSQCRACQPFTFKNTLSNDVRCRPCPMFSNSSDEATAVCP
CDSGYFRPLDGSEDAMPCTRPPSPPRSITYSDVTNTSVFIMWEASIDNGSRTDLVYTISQ
NVTDDDNVTTPNTSIMLMGLIPFVHYEITVTADNGVSSQDGNEEGRTLNIFIMTLGVPDP
TVLVNAPNTQVVGQSLTL
>Kvar_4564 K. variolosa
SVSAGCSGQVISSCSSSSSAPQNNWLFTQYISYNTSIEVFLNITHDFRRCITDCSPFITL
YKYDTDGIVSEEERTNRNNYQPLFGTEAASRVEQEGSTGDVSETLNLVRTVVRDGFYLAF
RDTGTCVSIGRVILYYRFTPGRSETFLSCPEIPLPIAGSQMISTMSCTCANTTSGVGSLD
RSCDQNGVCTEDQTCECDLGFGYNSTLEMCIECPVSTFKGVVTNSTCQPCPALSEARDPG
SAICECIPPNTRSGRDGPEADCRDRRLSTSAAVAITFFVTFLIVGVVAFLGGVLATYLCM
KCRSK*
>Kvar_25216 K. variolosa
AVSAGGGGDTCSPPEVSVTTQVLLDTYRTSSLQPAWNFGDSWGLSATTSCFQSFAVCNES

GSDNLLVTPFIGVSAADEISIFVEYTCPSLEPPNLCNTDFSLHIYQTSAPNQVGSTEISN
YALVQAVAPNNSRIPYNLADSGFYLAFRDTMSCVEITRVQVLYTQCPETVREFAQYPITS
SGAAAPGECVMNATLR*
>Kvar_137296 K. variolosa
IIGSMSFVPSIQLLSDQPVAKTVLNQKVEMLSFAIVVSVWMAHASAQAPGDYNLTCRTGE
NAPNHVFPAVWRVLVDTYDYLSLYNSLFGSPPWTAVDIDGDSLNWDPNFGGPCRFVLRPC
TGQSSQNNWLFSQHIRYEGAREVFFNVSYRFTQCIEPRCNMRYVTLYRYNRDTIDTGASD
RNNTNNYQPLLGDEVSSRLEQPPDTDGDVEETLAIARPANNLGFYLGVRDEGTCGEVTRI
IVYYVVCPLRVEGLVTYPETGVPAQSGADVVFDAECAPNAHNTTTLQVTAFAITSICSPV
APGGARCECNAGYLISSDGLSCEACPAGTYRTISDSSCQACPANTMTDVVAAPQCNCING
FFRSPQEGPDTGCTEPPGGCTGINLASMTSRSITVRWSPPAITGRSDFFYRVFHTDPDNI
GEFILARDNLRDVVMYEITGLVPFTPYIIRVTTHNGVSDLDPDGADDRMCAISTMTRQGV
PGLPSDVQYLCTVVVWQEPTEPNGDIIGYDIEFSTGSRRIGAGMTFIITNNDERMPGTTA
RVRAVNGAGVGAWTDSGDLGCGPGGTDCTNCPSTLPVESSCPSQPTPPATTECPVMTCPT
TQPPPAATECPVVTCPTTQ
>Kvar_222743 K. variolosa
MMRLSWFVVVSLATWQASGQVPDSSCLTGAMTADQHIFPPDWEILMDIYDYLLGPSWPER
DFDADGNQIVSSTTWEQIGGVCTSTYQACSRSTSPQNNWLFSHRISYGDAIEVFFNISFQ
FSRCRDNSGCNNDYVTLYRYDTNELVSDSARTNPNNYIPLLGSMLESRLQQLPTPAREVN
LNYNLVPSQASSGFYLGIQDDGTCGTVNRIIVYYRFSSGRMQDLLTCPDVALPPQGTTSQ
ETCTCNANASPTSQTLNRVCDADGVCNEDQACACSPGFELANRMCRACPEGEYKIGISNS
SCLSCPTNSESRGEGNAVCSCIPPNIRNPERPQDPCTTSPSSPRLLLTSDGVPQVSQDSI
TVCWLPALDTGGLDDLHYNIYLYDTTNPAPEFNKVNDELLTQEEGGDNQASICYVLQDIN
TQTSYGVIVVAANGATGDPAMLRDLDQVQGHSALI
>Kvar_222854 K. variolosa
SVSAGQTRETDFGTDEEGFYIAIRDDSTCIVVNRLIVFYNVCPGGPRDLVMLPETIAPRI
QRTSQPLEVTTQCVNGASPISGDVVKLNCIQGGVWSSTSGFGCSCNTGFNTSADRRSCLG
KNYMYCTHR*
>Kvar_222799 K. variolosa
CVGRGAASASLVMLQSPKLRMASTLQAFLIQWSMLSVGLLSVVSAGAPGDGDTLCTETLL
TDGGVERAPLPANAKVLMDSYDYLTLLSGSPGAVWQQVDLDDNNAIGRGTEWSQNSNLPC
RQTVTACGFEEGMQDNWLITQYIRRRVNGVLLPQVSVQAVFELRGCLTGTACQRTYVLNV
YETSTENSTLARNTESYRQVGRVATDDDTGLTAQNQTRETDFGTDEEGFYIAIRDDSTCI
VVNRLIVFYNVCPGGPRDLVMLPETIAPRIQRTSQPLEVTTQCVNGASPISGDVVKLNCI
QGGVWSSTSGFGCSCNTGFNTSADRRSCLALCGAGEYYSVTAAECRTCPGNSVGGDSGLS
VCPCEDSYYRAEGEEDLPCAKPPSMPRNVQLIGQTITSITLSWDPPVDFGGRESVDYYLC
YEADGMEEDCTIVRVVTMGTITGLKPGVKYSISVTAMNSVSPFATLASSSSAPPILVETT
SVDRTPVIAGAVVAVIVIVVIVILIIIIMLVFILYRSKIAKDKYPVTEAIQIPVNKHRTY
VDPTTYASPDEAVEEFAKEIEPKNLRLDAEIGTGEFGRVFRGIWKDIPKKQVTVALKTLK
PGSTGKMRADFLNEASIMGQFKHDHVIKLYGVVTRVDPAMIVMEFMDNGSLYSYLRKNDE
KLSSQSLLKMVQGVCAGMEYLATVGYVHRDLAARNVLVSRDELCKVADFGLSREVIEDEY
NVQKGGKIPVRWTAPEAIFRRKFTSGSDVWSFGVLMWEVMTYAQHPYDEWDNQTVLDQLE
AGYRLKLPHNCPEPVYEIMLDCWNKDLATRPSFAEILKRINSLLNIESPPKRAPKRLHNL
ATLR*
>Kvar_2233 K. variolosa
RTALAQSPIGGCEREGTPDACPGAPADYSPTCFTGNFADSHVFPFYWDIVLDTHDYLTVY
AGLHGFTFGRPVWKNKEFDGEEFDGDGVTWEGGRGGICQSSLYTCSFREKNQDNWLFSQF
VCLGDAKELTFDVSFRFTRCAGIPACEHPYVTLYRYCVDEPVSDSDRIDPQNYVLLNGHS
ALI
>Lapi_88092 L. apicalis
TFGRGLFAKLGVGWANFSGDCVPRTVLMLVLLICSCPSMICPRRYTRPSMMCGAVCVALL
LTLSPLVTCQAPGDESGSCTPSVADRTMVLLDSYNYETLIPPWSEISSDEDNRTWVEVSG
SGSCRQSVQTCSGNSASSHFLITQYIDVSMAQEVNVIIDIASCTTPLSGCTTEFSLLAYS

TSAMDSPGSMDISNYREATAVVMNNTQIPITVSTAAVLYLAFDGSSACVVINRVQVLYKI
CEGTLNELAMYSEATSGEIGDGMCVSNSMPMAGANLSAPCGFESAMFDFSSAGGCECDAG
YNGTLCDECFPNYFMSGGLCTECGSGSTSGGGRAVQCACGDGTATADGQSDTTTASCDSC
HAGYFRSQGTCMECPAMSNRQFNQAQELCFCEDSRATSENARNTTLLPCEGCASGFYKAA
AAPTVCMQCPDNSITERFDDTSCVCKVGTRLNSNLDTTTTEPCASCAPGFYRSSSVCVRC
PSNSFRSMATDRDSICECSTKYARINTNASAESCLPIVGFRSASVTFTEGPSVHGVNLSV
SVEVMVGFNVPLSRVDVPRVNFSTNVEILPNTLSQVVPIQFLDNEVALEESSVFVIEIPT
DDTPNFVVGALGELYDSIEVTIRDDDNVEVGFTEATINLNESTRMDISIMRRNSIASDLR
LRISGVEPPLMLMPESLDVVFASDGPDTTTISVSTADDTVGGQTGRSSTLRLEYIGSPEF
SQSVIIGGERTSEQIKIIIIDNDPSPT
>Lapi_96494 L. apicalis
MVSALILPALLLALQQRVLVSGQSPGDFVPSCRTGLNTNNHIFAVDWRILLDSYDYANLY
NPQLTPSPVWISRSFDSDSGGTGNWNQVVGGVCTWSLRTCSFSSPSQDNWLFTQFVSYST
AVEVLFNVTYSFSECQQSSCNPYITVYKYDTDGIVDDAARSDRGNYLPLFGTEQESRLED
AERMGRPIDRTLRFNRPSGRGGFYLAFRDQGTCGMVTRVIVYYRVIDGFTDASVSCPEVP
LPVQGSGEPSTLSCSCVLSDSSGVESLSRTCGENRVCTEGQRCACDPGFEFNATANTCTI
CPLQTYKAGISNGSCQSCPSLSFAVTPGSTVCECISPAIRAEGAGPEEECRLSVPGGVSS
AAAVATTFFLTLLVAFGVGFPLGLLVMYLCIKRGS*
>Lapi_108357 L. apicalis
MKAVVATILLLSARVVQITTLTIIPDYDFECEPEGIPEFCPGAPADFDPKCRSGLNRANH
IFPENWRVVLDTYDYPNLYDSGRVQPPAVWEVGNFDGDNFPWRQIVGGICTWSLYICNFH
TPNQNNWLFSQHVCLGDAKEVYFNVSYRFTQCRNNPSCHDDYLTLYHYDTDGFASTEERT
NIDNYQPLFGDEVSSRLKQTSASVTRSFLRPQPRRKGFHLGVKDSGTCGQVNRIIMYYTV
CLARQNGLVLYPEYATPPKDGPDEIFHAKCVCHAHPVTNMAVIASSETSTCKEEADGGAM
CECDGGYYISDDLESCLPCAKGFYRAAGESNTCQPCPPNTTVDAIAAAQCECLSGLFRDV
PNEGPGSGCTKPPLAPTELQVVQATNTTVTISWRFSLARHDDYFDVCYKNFVDFAIAICE
EKFIPASTCLESHCEYTIRKLTPFSSYLFNVSAQNGVSDQDSQNNDRRVATFVDGTTEGV
PNSPFLKRLCTVVIWKPPTRPSGIIKAYDLCFGQCSGQSLLDPTCYNFAADDNFFVTSDE
MRAQDIGVQIRTNNSIASSPWTECLPIDCAKLNKPSASCPLEVPNTDTPTTRPPTTSTPT
TIPPSTQPATRPPTTQPTTRTPTTQPTPTPST
>Lapi_126388 L. apicalis
DLRPGEEAMNLLAMSRASFSCILLLACICLHVFGQAPGDHDPSCREGLNRNNHIFPAEWR
ILVDTYDYPNIYDANQNPPPVWVAHDGDGDSINWQQFVGGICTWNLRTCSGQATQDNWLF
SQYISYEGAKEVFFNVSFNFNECTPRPFCTRPYVTLYRYDRNTMATEVQRTNPSNYESNP
LLGTLQASRLDQSGSSRQVEDTRSLTRPASSNGFYLGVRDEGTCGQVVRLIVYYVVCPGR
VDGLVTYPETGLPVQGSSDFIFDAVCASNSFNTTTLQVRASSSTSSCSIIAPGGARCVCN
AGYIRSGDGASCQACGAGMYRAVSDSSCSPCPANTVTDAVAASECSCVDGFYRTPQEGPA
TACTEPPGACTNIRVDSTTGSTITASWSRPAITGRSDYFYRVWRSDPENIGMFVQVIDNR
RDTRSTVTYPVTGLEPFTSYIVRVSTHNGVSEQDPDGHLRQCEVTTMTAEAVPGLLEEVS
GFCNVIVWQAPAKPNGVITGYEVLFSNNAQPQTRQANENLHLTAAEQRENETTFQVRAIN
SAGSGALTTPQLVCPGPCLMEPIRTNATSDSITISWSRPNLIGTSQSITYNITVGDRTIN
DIRDAGTVITREVSELTPFTTYTLTISATNGENSEECRTVAKTEGKESTSPIDPIDFCQV
LVWNPPASPNGILSGYYIQFSELTETKRINNSEQTFYMTTAEEQAVGGSVRIGARNHIGD
GRWTDISHDCSGVAATAQSADLFGGGIGVEASIVIIVLCTVVITLVIATMLFLPIALCIR
KRSNPSTSDTPTASSKLQYQLT*
>Lapi_154083 L. apicalis
FSAPIFPPNSRILMDSFNYLNLLPPHSGAVWQEVDNDDNNVEGRGTSWSQNSNNPCRQTA
NVCGLQLGTQDNWLITQYINRRVNGTSLPQVSVLVEFELRGCTELVCQRSFVINKYETST
ADNATARDTGSYDIVDWVSTTDDSGETRQNRTREINFNTDADGFYLSIRDETTCIAVTRV
IVFYHICPGETEGLVMRPETLAPPIDRLSPPLEVTVECVDGTSPENGVASKLNCNQGGVW
TPISGSRCRCDEGTFTSVDGQSCTGCGAGMYLSGQGCTVCPNNSMSMEAGLAECPCVEDY
YRAPGEEDLPCTQPPSSPRNVRVESQTSNSITLIWDPPDFLGNRNDTQYQLFYQKVGSTE
KEFSHSVNITEGTITGLEEGVEYSVFVRAVNGVTAFAEGGGSEEEVPLTVLTLRSPDTTT

PIVVGVVIAAIVVILLVLISLVAAIIFYREFFRSKYDVKAHEAIPIPVNRHRTYVDPTTY
ATVDEAIEEFAQEIEPKLLRLDSEIGAGEFGTVFRGIWKEQPKKQVAVAVKTLKPGSSVR
MRSDFLNEATIMGQFNHDHVIKLYGVVTRVDPAMIVMEFMDNGSLYSYLRKNDEKLDTQK
LLQMSRFVCSGMEYLATVGFVHRDLAARNVLVSRDEVCKVADFGLSREVIDDEYNVQTGG
KIPVRWTAPEAIYRRKFTSSSDVWSFGVLMWEVMTYAQHPYDEWDNQTVLDQLEAGYRLE
KPHKCPQEVYEIMLDCWDKELSNRPGFEAILKRLSQLLHTDSSPSKRPPKKPRSEVNSAT
NPLHCKTVDNWLESVKMDRYIPMFQDNGITSVNQLVHLTEPDLRDMGITLAGHLHRLTQS
IENGYMELKRTSTVGAP*
>Lapi_159044 L. apicalis
KTFGRGFFKAAGSSFIIPTSAKMGLFLQVASLFSLLLTAHKTLAQAPHDHNEACRLGRNT
ANHIFPQEWELLVDTYDYTQLFNPSSTSSVWRSETLDGDNDQWNQFVGGACTWSLYTCSH
RSGPQNNWLFSQYISYGSANEVFFNISYSFTQCRDNPRCENDYVTLYRYDTDSIV
>Niph_32084 Niphatidae sp
KWIQRLLLAFYFHVVSSQAPNDVSQFCKDNSLVPDYPETVKTLMDSWSYQYLLGDKGVWQ
SVSLDDDSEHWMQSGDGTTCANTVSVREPMNLPQDNWLLTQHISKSLPQQADLYPTLVYV
NISFSIDSTCTNCGDYFQLLFLYTDSALSDINNRTSEFTSLGNLTAKPGLPVTVVTKTFA
INPDQDGFYLALRDYGSNITVSRLMVYRLIGPAMQVGLTLFPETPAPTLNCTTVAGVCVA
NANTTNGTLPRLNLDPDGSWTHSEDQCYCIEGHQLVSLEGSDQCQACSPGTYKFLKDNSD
CLNCPMNSDSSVYGASMCMCWDEYYRAPNETPDVKCTQAPSAPQFLQTSSITNTSVVLNW
NTPQSQGGRTDTYFDVFYVANSVPRTQANDGPVLGNTYQVTGLNPITFYEVTVIAENGVS
SQAGGEDLRSAIAFVPTSIGVPPPVEGVYVRMLDDSVVEWLPANAINGDITGYQVQLYSS
EENGPMIDVEWSMVAYVVNVTEDVPLSGQPVYVRVRASSNAGFGNWSDQVVFANVNPIAP
TSTSVPVTTPPSTGSPSSSSPGSSPTSVPPSTTPPSSSSSALTPSSSTTTTSTTTSNHHP
ATSSSATPTESSSDSSPVLAIVAVVVGAFGILLLVGVGALAYFIFRKVRLRKQKRQDEID
YAPLVHYNPSAADGGGGDGEEEITVELKDHPIN*
>Niph_54283 Niphatidae sp
TQSTWGTSLSILAATTASMAWLLLILHLSVLTVSAVSVAPGDANPLCLSNPNRQPVPSNQ
KVMLDTQDYLEQTLVTVNGENGIWRSENFDNISNSNLFEKWVDSAQSASCFHRFLACDTE
NLPQNNWLITQYINITANGVDELFINMTFTTVLSGDDCPGCQQSFSAWSYETNVLDETGR
SNQSFYTDTSARFFHKENNVNRESAEDSFALTSKGLYLAVVDSGSCTRIDHLYVFYYVCP
AQVVNMVQYPETVSPPTTTPQDRVAIGTCIDNASPGMALELECNIFGNWESNDLSCSCDP
GYENITDLCEPCVEGEFKSAQGNHDCTACPASSNSTLPGATICECVEGYYRTSTETAIDN
CTAPPTVPLTPMAISIGTMMVSISWTPPTSDGGRDDLSYTITYSNGSFTSPVYSVVDGTE
YSITQLTPSTQYTISIESVNGVSDQDSINAASRTVSFNVTTDGRGPTTPNDVRIGTGDNG
TQILLWAEPEFLFGTLLHYNILLSATENSTDASVVANTTERVFELDSLNLQQGTYYIWVQ
AITDEETGDLSLSVEYTIDTTIQITPLVSPSMRSTSHMTHSTQSTSLVSQSISYIPQSTP
QITPQSTDTASAYAVSMNLIVVAVVLMVPFLVIG*
>Niph_54284 Niphatidae sp
TQSTWGTSLSILAATTASMAWLLLILHLSVLTVSAVSVAPGDANPLCLSNPNRQPVPSNQ
KVMLDTQDYLEQTLVTVNGENGIWRSENFDNISNSNLFEKWVDSAQSASCFHRFLACDTE
NLPQNNWLITQYINITANGVDELFINMTFTTVLSGDDCPGCQQSFSAWSYETNVLDETGR
SNQSFYTDTLARFFHRENNVDRESAEDNFDLSSKGLYLAVVDSGSCTRINRLYVFYYVCP
AQVVNMVQYPETVSPPTTTPQDRVAIGTCIDNASPGMALELECNIFGNWESNDLSCSCDP
GYENITDLCEPCVEGEFKSAQGNHDCTACPASSNSTLPGATICECVEGYYRTSTETAANM
CTAPPSAPLAPMATSVDTMMISISWTPPTSDGERDDLSYTISYVNGSFTSPVYSVVDGTE
YSITQLTPSTQYTISIESVNGVSDQDSINAASRTVSFNVTTDGRGPTTPNDVRIGTGDNG
TQILLWAEPEFLFGTLLHYNILLSATENSTDASVVANTTERVFELDSLNLQEGTYYVWVR
AVSNYGLGDLSDPVKHVQFGTDSTTSGSSVVPIAVGAGVGGAVFILLLLILVVVVIIVIV
SRRNSEKDKYVVAAYSATQSGGIVEMGELPTNDPAVSNRMYVDPRNYSSLNEAFDETGIK
EIPPKEISNLDEIGVGEFGLVYKGTWSLKNVKVAVKTLRGGSTEEQRRNFLFEASVMGQF
KHPNVVKLYGIISRVDPIMIIMEFLENGSLDRYLQKHLDKLGLQRQMKIAYGVAVGMEYL
SRIGFVHRDLAARNILVDKDETPKVADFGLAREMVENEYGVQRGGRIPVRWTAPEAISHR
SFTTASDVWSFGVLVWEIMTYGETPYEGWDNFTVFNRLEEGERLKQPRYCPDDVYQLMLR

CWEQERSERPTFQAITEGLEIMIEGKPKPKPKPKHLKSSLSPPPSSAPPTGPQIVSIDEL
LNSISMSKYVDLFHQHHLFTIQDCLDLTEDDLERIGVTLGGHQYKILKNIKIRQEELGN*
>Ocar_5890 O. carmela
IKDVVGGNNGGRMDAIVDVSNGEKYVFQVRAETSAGAGPYSSPFSPEGGDPGEGLNLLVV
IVPAVAGAVVLIGAIIVIVICCKRGMCGNGSSMNSNYNVSFASNEAALLPRKGIYVDPTI
YASADEAVKTHASKIDPSHVDLQKVIGEGEFGEVYLGLLKGKEGKNQKKQKGKKTKIAVK
SLKPGASAKDRDNFLMEASVMGQFFHENVILLIGVVTEGPRMLIVTEFMDNGSLDNYLKD
HDNNLELKVQLQMALDVSRGMDYLSSMRFIHRDLATRNVLVDANLECKVADFGLSREAVD
ENAYDVKTGGKIPLRWTPPEAIDYKKFTTSSDVWSFGVLLWEIMAYGEHPYWDWTNQKVL
ERVRQGYRLPPPMDCPVEVHRIMLMCWETERTDRPTFGKLSEELQTGRDTLQRSRVYESV
DSWLADLQMDRYVNQFEEAGYIDMSQLASLSHEVLKDRIGVSIAGHRNKILKSLQGYKQL
LPS
>Ocar_909 O. carmela
MLSGASSRLPSLATFLLLIQLSNQQGFDLLCQQAFADNTGKPWRSPPPNTRFLYVSQDQQ
KVIPPWISFQRDNDQSLGSAASEPVEWVTGGGTCSRAFRVCGYKRVQRQDNWLLSPYINV
SEAEAVSIDLSFTMTKCIGPFGGNCQLLFDVYTNLTSDVNVTEASFNDTDFGKFTRISGT
YDDGLTLNSGEVKLEVKGFPGLYIALRDTGSCTSLSNVIVKSYVCPDDVSGLAQFPLTNS
QLKSRVVTGTCVANATSSGIPQMRCEPNGAWDYTSLLMECQCMAGFEAINDSCSACGPGF
YKSSNMNGLCSACPLKSYSSAPTSTFCPCQQGYDRADDEPDIGNVPVCSSTDQEPGDAPQ
NVLAVVNGDTVRLYWDPPTKPYGAIERYTIRYFVNGTGDFKTKDVVGGNMVDGRMDATVG
VSNGEKYVFQLRAETSAGAGPYSSPISPEGELFSGWEYPLIGSVDMRSSL
>Pfic_17511 P. ficiformis
VNDTFYSDTGVRLFHTTEQGSQTTASQRFTISSRGMYLSVVDGGSCTAISRIVVY
YNVCPYQVVNMVVYPETVAPPFTNPQDRVVTGTCVNNSSPISGDLNQLECEVSGVWGDTQ
VMCSCNAGYENISGICEACMEGEYKSLQGVDDKCIVCPENSNSTSVGALSCDCLDGYYRV
ENNMVDTRCT
>Pfic_36041 P. ficiformis
QWVDLDQGIVCRHRFIACDYNNQPQKNWLVTQYINISDNGVDELTIYMTFSSVTQCACQQ
SFSVWAYETNVIDETGRVSNTSY
>Pfic_10202 P. ficiformis
DTGVRLFHTTDQGSQTTASQRFTISSRGLYLSVVDGGSCTVVSRILVYYNVCPYQVFNMV
VYPETVAPPFSNPQDRVVTGTCVNNSSPISGNLNQLECEVSGVWGVTQVMCSCNAGYENI
SGICEACMEGEYKALQGVDDRCIVCPENSNSTSVGALSCDCLDGYYRAEDEGVDNDCTAP
PSAPINLLTTNIQGTFVTISWTAPSMTGERSDISYNITYSNGSFNSIVYSVSKTHHTLTG
LIPQTTYTISVISINGVSEQDPDVESRTVSIIVTTDVSVPTSPLDIELSSSGEGVASILS
WTEPASLFGIFQHYNIRVAEVDVSNQSTVRDTTNETMYDLSDLDISAGTHYVWVQAVTDI
GEGELSTSVRYVRVMEDESTSSNILSLPAIVGTVVGVVLFLMCVIIIVPVVIVVLMVCIN
RNEKGRDYSVNSIPSHFTPNHSGEMELRIIDNDDNVRHRMYVDPRNYSSLNQAIEQTGVK
EIPPKEIINLDEIGVGEFGLVYKAQWSLKNVSVAVKTLRGGATENQKQNFLFEASVMGQF
RHPNVVKLYGVISRVDPVMIVMEYLENGSLDRYLQKNLDKLGLVCQLNIAYGVACGMSYL
SGIGFVHRDLAARNILLTKEKVPKVADFGLARELVENEYGVQKGGRIPVRWTAPEAISHR
SFTTASDVWSFGVTLWEIMTYGETPYEGWDNYAVFQRLESGERLQQPKDCPDDVFQLMCN
CWEANRSERPSFDQICENLEILINGKPRPKPKPKHLQKSPPPPLLHPSQSNPLIAQGTTI
EELLRSIGMLKYLDLF
>Cvar_1757 C. varians
MDLWNYVNLLPPRIGAVWDQVDIDTNNPVLVTTNWIQQLNSPCQATTQVCGFSAGAQNNW
LFTQLINRTINGTHLSEVMVLIELGQQGCDISLMCQRTFNTHIYETSLEDDVARRNINNY
QQAQRISPSDTSGSRVNETVVITFQTDHSSFYFAVQDVTSCIIITRLLAFYHVCPSQTVN
LISLPEIIAPRMGEAPITVTAT
>Cvar_18894 C. varians
QQTAQVCGFAAGQQNNWLITQLINKTVNVPQVTMTVEFEMHGCDITLNCQRTFNTHIYET
STENAIAARNINNYQQVQRVSPEVTDGSRVNEAIMVNFSTNHSSFYFAVQDETTCIVITR
LIIFYYICPAQTANLIQYPETIAPSTGLQIGSSSSQISAFCVENAEPENGLAPTVTCAVE

GIWDSIVPGAGCRCVPGYFRENETCTPCPAGTYLSPNNGSCLTCPANSVSEEEGLTQCTC
VEGYYRALQGEEDLPCDLPPSPPSNLRIVERTETSITVAWDPPMQQAGTRKLSYGVYTSV
NGDPKVNVGVVNETLIVIPNLLPGQSYTLYISSENSITDQLSENQSAIIIASIETSTLEN
ARPHEGPLVAAIVPPLFILTLVGLALLTVVILIIAKQKKLVKTAANQEVYYSTVGPPSLP
VYDEITTLDRLQKMKDTCSMTTTSFSDDTDEVNGNQSDPNFALNQNPAYGTNDSTAPAPE
FEIDKNEAYDNPELCDLPKSSKSQANPDHSTMDTDSDTDSHYYY*
>Cvar_19692 C. varians
MMAFHCLWVTFLVELLVLVNPGSTVAPGDEEPVCEQLRAIGAAPIPAYSANMRVLMDSWN
YQNLLPERTGAVWDQVDNDGNNPSGTATNWIQNSNTPCQQTVQVCGFSAGPQNNWLITQL
INRTVNGTRLPQVSVTIEFELQGCDVTLNCQRTFNTHIYETSSVANAERRNLTNYRQVER
VSPDTTDGSRLNVTITFSFNTNHSSFYFAIQDETTCIVITRLIVFYNSQTDNAQGQAQTA
IRVIKGGNVGQCPSEEERERARNEVHQIANSAIASSICNDTPGWRRVAFINMTDIRYNCP
SGLSLTSYSRRTCGRSHTYRGCSSTTFSVGGMPYSWVCGRIKGYQFGSTSAYGQSSQDID
SHYVDGVSLTYGVAGSRNHIWTFAAAQKGQRSCPCIRKGKATLPFVNGNHFCEVGIHNPK
KFTASNPLWDGQGCAGSSTCCQFNNPPWFCKQLDQPTTEDMEIRMMANVISGYFLEDEDT
PVQLIEIFVQ*
>Cvar_19694 C. varians
KSKFSDKDMMAVLCLWVIFLVELLVLVNPGSSVAPGDEEPLCEQLRAVGVAPIPAYSANM
RVLMDSWNYQNLLPVRTGAVWDQVDNDMNNPDGITSNWIQASNTPCQQIAQVCGFSAGPQ
NNWLITQLINRTVNGTRLPQVSVTIEFGLQDCDTTLACQRTFNTHIYEISTENTTAARNI
NNYRQVQRVSPDITAGEGVNETVTVNLNTNHSSFYFAIQDETTCIVITRLIVFYNSQTDN
AQGQAQTAIRVIKGGNVGQCPSEEERERARNEVHQIANSAIASSICNDTPGWRRVAFINM
TDIRYNCPSGLSLTSYSRRTCGRSHTYRGCSSTTFSVGGMPYSWVCGRIKGYQFGSTSAY
GQSSQDIDSHYVDGVSLTYGVAGSRNHIWTFAAAQKGQRSCPCIRKGKATLPFVNGNHFC
EVGIHNPKKFTASNPLWDGQGCAGSSTCCQFNNPPWFCKQLDQPTIEDIEIRIMSNANSG
FLLEEEDTPVQLIE
>Cvar_19695 C. varians
MMAFHCLWVTFLVELLVLVNPGSTVAPGDEEPVCEQLRAIGAAPIPAYSTNMRVLMDSWN
YQNLLPPRTGAVWDQVDNDGNNPSGIATNWIQNSNTPCQQTVKVCGFSAGPQNNWLITQL
INRTVNGTRLPQVSVTIEFGLQDCDTTLACQRTFNTHIYEISTENATAARNISNYRQVQR
VSPDTTTGERVNETVVINFNTSHSSFYIATQDETSCIVISRLIAFYHVCPSQTIGLVHIP
ETIAPPTGRPPIPVNERCVENAQKVDDDVFVCSPGGIWTPVTPLGTSCRCELGYIHRRLN
GTDICLRQLSCESLDNLIDIHINRNELRCQRKLDCSGIRCFSQNSFLRNFIEVFDIRYLL
CTNLQPSRSVLWLQLLGEEDPTNNNRRSVLLNRNVTQNVNFDLPVMSGGLTFGVYQFTVE
FLGSGGGSGIALAIQLIRSGVTETVIGLMDIIPFSTEDCSPATPPRNDTPTTQTPLTTTT
DTPTTQIPLTTTTDVPPELILTPPNEVQSHCSHVIWRRSPNVSCEDIIGYDIWLFNPDTK
EEVVRRVDARGTFHDFLLLDKDLTEQKSTTVQIRIVSSTEVGPYSLPVEVGCFIPQPSSV
YFELK
>Cvar_19697 C. varians
MMAFHCLWVTFLVELLVLVNPGSTVAPGDEEPVCEQLRAIGAAPIPAYSTNMRVLMDSWN
YQNLLPPRTGAVWDQVDNDGNNPSGIATNWIQNSNTPCQQTVKVCGFSAGPQNNWLITQL
INRTVNGTRLPQVSVTIEFGLQDCDTTLACQRTFNTHIYEISTENTTAARNINNYRQVQR
VSPDITAGEGVNETVTVNLNTNHSSFYFAIQDETTCIVITRLIVFYNSQTDNAQGQAQTA
IRVIKGGNVGQCPSEEERERARNELHQFAASEIANYTASTTTMPSP*
>Cvar_27357 C. varians
MRLSTVKFFGCLVILISTFAVIDAVAPSDRNPTCRTDFRYIVLHNWIPYAEDTSSGTAWK
VLIDQYDYESLVGSGMSSADWSTLDVDSDGKTFAHGSGGTCSFQLKIPDTFRTPNSNNWI
FTQAIDYIDAVELIVNATVRFTACTQRLNVQPPERCTHDYVILHRYDTNTQSETQRTTTT
NYQPYKDNSESSSRLQQDRNDIRSNTNIINRFRRPINFNYTYLGLRDIGSSGSVVRIFMY
YEVCPRKVEGLVIYPEVPHPGRLPGASTRTTRLASCAEHSHNTTSLDTYAYQDGRCEQSV
TCVCDAGYEQSQQNPPQCVACAAGKYRSAQNISCTNCPANSNSTMAASEYCSCNEGHYRV
TGSEGVGVGCTAPPPRIPECTVSQRFDTGVTIQWTTPADCGGRTDCYYQININDGSPKRH
SPGFRPNTQETYAINNLQPDTTYSITVSIHNGVSNQDSDNARIRECTIVAKTIQGSVFML

DGVTYLNNTAVLLEDIDANPPLICTTTHTSCCTGNTQVRFFDPDGNPILTQTSQGSISLH
LQADELPPLGAYRCEIPDSRGTLQNLYIKIGTITRNATASTSTSTEVPCLNLYIFIGVAV
GLVMLATIIIVLTCVVCLCKNHERKSVKLHRNGGARNAGFNDIKL*
>Psub_7666 P. suberitoides
MVSLWLALLAVLVWEVTGQSPVCQLVPTSGECVCGDSEVHSYCSSSSPCAPDDFQIFCRT
SERLRDEHILPDEWELMLDSHDYQNLIDTSTAVWVNRDFDGSGGAWLQRGGACQWDLRAC
AFASGFQNNWFFSQHIKYPLGSVAGFPTVVYLNITIRFSQCLGNSACPNPYINLYRYDTN
EVVSGTQRVNTANYMPLFGPGTEMTDSRLVQVGASAIGFVREFVAPGNTGFYLGVQDEGT
CGQVQRIYFYYTPCKAMQDGLVNYPELVRPPNGSPPNTAQACCAPNARVASGSSLNFRAY
SASDGRCERNVVCECIPGYRIDESGTGCTACPAGTYRSSMDPQIACLNCPMNTLGDAVNA
SVCECIEGYYRDANEPPGKECTTIPSAPNNLSCSTTGNVIAVTWQAPSDLGGRTDTDYIV
EYQSVTSTGDFIQDSQTNKTMVQIGGLDFVTQYRVRVVAENGVTDQEMRTGFRQIRTEDT
ICETLEGPPGPPSNIKLINNVVFLWQPPTKPNGIIQGYHIFISYNSSEDSGSVNVSFDAS
IFVYVLRMGVVPENVTAFFSVRANTSAGHGEFSDPIIFETFSPPVVVITRNVCTLDDMAL
ALSISAVVAFIVGFFVMLFLCLLHLCCRKCCCATDKYSTSKEGIPNTVALSDTKVEWT*
>Psub_10078 P. suberitoides
MXXIFRRKFTSSSDVWSFGVLLWEVMSYAQHPYEAFDWDNQTVLDRLESGYRLDNPKDCP
QAVYNLMLECWHADLTRRPPFSHIVQTLEQLLETNMNGPNVPNNAAPKKAGKTYLSSANI
VNPLDYTAVDDWLRAIKMERYQSNFDTEGYKSVNQILHLREEDLREMNITLAGHLYRITS
SIEKAQTQLNRQPSVRV*
>Psub_14382 P. suberitoides
MRLIQLLILTLTILEITWACIDDETAQFESFCRTSERLADEHILPDEWGLLLDSHDFQNL
IENANPSWRRLSNDADTGQFVQRGGVCQWDLRACSFTSGTQDNWVFTSYIRFPSVLDVDE
VTEVFFNITFRFSQCSGNPACTNDFVTIYKFETDTPTSSNQVIPNNYLTNPLMGTVEQSR
LQQSGESQQTVVWSIPKPTTN
>Rfib_47059 R. fibulata
SSKKTYRDMLELKYFILVLFFIKVMVCENNNIYRSFSSSPVKEIPGWEVYNDDNTTYADE
GSSDVDTVTWILNSNDGLPGPEYIACQGGRNTVVNHTLEDNWLITTFYSSSASTIYIEII
CNYPRDREGNCSELFSTHILQSDTLVDVSFNHDQFEQISIQTLNSNNVTYSFSLSLNGFF
IAFRNTGYCVRLSSFRFYYEECPSSDQLPGVPVPTPSNEPTFSNLICPEGSISLRQAECY
SNGSWLVPETAVCKCERGREKRNQSCIVCPENTYKFDIGNDNTCVRCPGMSNTNSSRGSV
SCECDEGWYRSAEESVDMLCGRSPSVVGDLLLEREGGSTRVSWTAPVDVWNRSVSYNVSL
YLEKEGNVELVWSDNIEDTEYVLSESVLGSMSREYLLVVTSLNNLVVLSGVENNVSVRFV
SRFPEVVDMSVRLNDGILEWQYRLEGGVSSLSFELKYTNNGGELSEKTVNGCVAVSTAVY
KCSVSVVELNESLNIVITLLPLSPDVTNGMISQTYNLDNPMVRKTITSAITTVRNPSTQQ
TTSTPTTQTELLTLETKIYLVIAGGLCTLIIFVSCFIIAFIICCTLIKRRILRIYRSFSG
KDHVRYISVDTTATITKPVLFRDPDMFQNLIEAVRYFAKEVNNIDIKIDQVIGNGEFGDV
CLGTLNQNGHSVDVALKTLKPDVTERHKSDFYREASIMGQFHHENVITLLGVTLQQPIMI
VTPFMHNFSLLQYLRRNPHNITLTQQGKMALGVASGMTYLSSISFIHRDLAARNVLLDEN
MTPKIADFGLSRETEEEIYTMRKGGKVPVRWTALESILRKRFNSASDVWSYGVVLWEIMS
FAQEPYDKMEVCTLVHQLQEGYRMPAPLNCPDNVYQLMASCWLEDPDQRPTFTEIHSTLS
RMTWLNIEVRKSRRLTGSADLLCFSSVLEWLESLGMERYEDNFAKNGYTSLPSVWNLREH
DLMSIGIIPIGHRNKIMTSIRKANQVICLTGSTRV*
>Rfib_47064 R. fibulata
SSKKTYRDMLELKYFILVLFFIKVMVCENNNIYRSFSSSPVKEIPGWEVYNDDNTTYADE
GSSDVDTVTWILNSNDGLPGPEYIACQGGRNTVVNHTLEDNWLITTFYSSSASTIYIEII
CNYPRDREGNCSELFSTHILQSDTLVDVSFNHDQFEQISIQTLNSNNVTYSFSLSLNGFF
IAFRNTGYCVRLSSFRFYYEECPSSDQLPGVPVPTPSNEPTFSNLICPEGSISLRQAECY
SNGSWLVPETAVCKCERGREKRNQSCIVCPENTYKFDIGNDNTCVRCPGMSNTNSSRGSV
SCECDEGWYRSAEESVDMLCGRSPSVVGDLLLEREGGSTRVSWTAPVDVWNRSVSYNVSL
YLEKEGNVELVWSDNIEDTEYVLSESVLGSMSREYLLVVTSLNNLVVLSGVENNVSVRFV
SRFPEVVDMSVRLNDGILEWQYRLEGGVSSLSFELKYTNNGGELSEKTVNGCVAVSTAVY
KCSVSVVELNESLNIVITLLPLSPDVTNGMISQTYNLDNPMVQNTVSSKSIPENTTSAQI

AISVVTFAILLVSVITILGLLAAVYFRHRRRKCDDTPTLYNNVYSLEAIYQDPGLYDDLN
TAVRRFAKEIDHQDLEYKEYIGEGEFADVCKGVLKKDEQRIMIAIKTLKSDTSEKNKHDF
FGEASIMGQFNHPNVILLHGVTLAKPIVIVTPFMENGSLDDFMRQKEKTITTIQQVRICV
GVACGMEYLTKIGFVHRDLAARNVLIDSEWTPKICDFGLSRETEEDLYNVKSGGKIPVRW
TAPEAIAYRRFNEASDVWSFGVLVWEVTSFGRVPYAGIDIVDLLEKVQFGYRLEKPDLCP
DPLYNLMLSCWQSKARSRPTFSEVLNTLNSLIVANFVNKQRNGVSVYNPESFSTEDWLDS
IGLSRYSRHFQEDGYCSLIQTCSLTNEDLIKLGIVPLTHRSTIMRSISEMHEIMLQDIRP
VSVSD*
>Rfib_49817	R. fibulata
ITKTYRMTHLLLLLLISLLYIPSLIQTQCNVELPNNSVVILESNNGVPTYEGFDGWNRFQ
VDSLEYTVSSGGSTLDDTLEWRSPLSIGAQCGPIYDSCFETDLVLPSDVNTTEMNWLVSR
YYETEANVVFIRIHCSILLQYETDCRKTFQIFYYESDTSVTVTQSLLSQFLLAGNTTNQL
INDQVVLASFYTSKNGFYIGFFNQIPCARLTEFLFYSYPECPAISGVYFVSAVLPPSPVD
EYIRVNVSCVLPFVIRYPLLPTADCYWNGTWRLPPDNVCECPSGAYLSSDSNSSSLSCVT
CPVDTYNSVVGDSMKDCIVCPLRSNTSGLTGSVECVCEDGWYRGEGESVSESCVQSPSVV
VNVHVKRTPDNVTELTVVWDTPTYLGIRDDSKLSYRVSYYKSVPPESKKEVELSEKQFVL
SDVSESTEYVIEVTSLNGVSTLSGVYNTVNVTVLSSFPDLESIRYNMTTNILEWSYSLYG
NREYVFELSYVSTESVVTTSVYLNMSECMCTGDYTCVCNVFVADLNPTLPISFQLFFEND
VLTYLNYNWIPNSTPSPDPPASLLYFIIIGVVLIVVILLIMFFVVLFFSMFIVRRKRSVR
SKHTTQGEIPLIQSFPTSTVEAGLDMLYQDPSLYEDLNKVIRSLTKELDHKDIEVSSVIG
NGEFGDVCKGSLNINFRVVPVAIKTLKVSHSEKSKQDFFKEASSMGQFSHENVIYLYGVT
LIKPIMIVTPFMENGSLDKFLVSNAETLNVLDSGKICLGVANGMNYLSKRGYVHRDLAAR
NVLIDSDFTPKIADFGLSRETQENVYDVKSGGKIPVRWTAPEAILFRKFNMASDVWSYGV
LMWEVMSYGKPPYGDIDNYTLLEKVQQGYRLEQPDGCPYLIYSLMLKCWDTVPELRPTFS
ELYFEVSSMVENNFNPRPNNRSSKSFAHSPLDFNSIEDWLTSLKMERYINNFKKNGYPNL
SSVWHLSEHDLLALDIIPIGHRNKIMTSIHKANHKLSRTYSVRV*
>Rfib_55118	R. fibulata
SVCPIALITSDPDIEKNWIVTSYYETEANYIQIRIECTSNTIITDCRTSIIAYYFESDEN
LTDVSSLLNQFKPTQLINITNDDDFFYFSFSKTERGFYIGFLNNRPCVNIASILFYYFEC
PAVTANFYFVNATLAPNSTHQPTVIPLSCVIGSQVVTPGTAQCYSNGTWVLPPDHIVLCE
CVSGTEGINSSCTNCSINTYKTEPGNTICTICPDNSNTDGETGATVCVCDADWYRGVGES
VIQSCGRSPSAVRNLRIQRGVVLAAEWDIPSDLGNRNETSYQVDLLETLSGMTTSEFIIS
MTEYNLDTVSSHTEYTITVTSRNAISSVSNVFRETSLTFLSSFPDITTSTYMDSYLSWSY
TLNAVSDYLFQLSYTSTQTGSDVTVTVNSSSCDQTDNTYVCRVLIPNLNASTGVSLSLLA
SSGPNVGIFSSKLSVETIPTPETLSIMMIIYYFILPIAGCLIVTILMLILFSCCVFCCMR
RRRRGFSPKTKESELMPMCAQQYQDPCLYEDLNKAVRALAKEIDANDIEKDSLIGVGEFG
DVWKGYFSRNNQKLPVALKILKPASSEKNKDDFFKEASAMGQFSHPNVIFLYGVTLRKPI
MIVTPFMENGSLDKHLTNNINNIPFKELIAICYGVSRGMVYLSLLGYVHRDLAARNIILD
KDLTPKITDFGLSRETEEDFYRVQTGGKIPVRWTAPEAILYRKFNTASDVWSFGIMTWEV
MSFGQVPYGDTDNFTIMEELQKGYRLPAPDSCPSIIYNLMLQCWSEDPEHRPSFTKVQES
LLQIIETNNTRPASVRMNTQNPLNHTTMESWLMSLKLEKYVDNFRDNGYSQISSIWHLTD
AHLFNIGVIPAGHRNKIMTSIHRANSQLSYTYSVPL*
>Rfib_2729	R. fibulata
MISLFKFIFSHLLLTFLSCQIQVYSQGNNNPSNLRYIFNSLDFPNANVFPRYQLLDLDNL
TYNISYESPLGGTRTVEETFECQQTSVLTSRGEPTGAYACCLSSGISTPVNTSVENNWIV
SDYHVTDATAIEVIVMCESVNRYPVPDCRNASKIYFYESDSTLVVNTTTLNEFTLVTSSV
NATDNDGIIRASFDKTKKGFYVGIQNNGICTFMRTIQIYYLFCPAVSINQSTSI
>Slac_37090	S. lacustris
MVDVESNPTGTIDVSVQLGASGGMYLAAQDTGTCVSISRLTVFYYVCPQQVLGFISYPQT
VASNGLITQLVCAPGASVVQQPQASCATGNLGVWGPPSGSCSCSPGYTNMSQTTQTCPPG
TYKSVQGSSVCLPCPSNSNSSVNRSTQCACLQGYFRAAGSGPNIGCIAAHLPSAPQLLTI
GATNTTAVVLSWSPPQDSGGMGVVFYTVYYQAVSGSLKMTLGNITTTSVTVTNLSPATEY
MMTVVAENGVPGDEANKSVSITVTTESVFT

>Slac_65214 S. lacustris
LALIGIIAVVVLMVFFVFSRSNKGVYLPPSTELVDIPQNRGTRMYVDPRTYQSTSQAVNT
VATEIPPKYIKLVEEIGGGEFGTVYKGTWNERKPPVPIAVKTLKPGSND
>Slac_1485 S. lacustris
EMLDLICRALLVLLSAAQVMCQAPADTNCKVDSKIPNILTLMDSNGYNNLLGEVWTATNG
DSSSTTWAPGTGGNCQRTLQVCEARTMFFKANNWLFTQYIPRPQLEYDITIYINVTLRMT
TSCSSQPMANCPRFLLVYNYISNQQQDVSVYTNSSKYTLLGNITSSSSNSPPITTTLNFT
LKAGDSGLYLGFRDQGSCTMLNRVYVYNYQCPQKQIGLVDLPLTAAPIVKNSPMAILASC
VAGGINTTSLSLQCSSGGVWSGSSTCICRAADGYRNVANRCEGCGAGLWLDSTSITCSAC
PANTMSSMPVTSVCPCNQGFFRAFDNLPSDNCAAPPHAASNVQVTQITVDSITVQWDYIP
DLSERSDYYFTLQYKDGDMTQYIKAANITALTYTIHNLSPYTIYEMQLITSNGVSDQDTA
NVFFRTVTFSCKTAEGVPGIIDDITISSRNVIWQPPAQSGGDITGYELLVLRGSQMTNKS
VEGNFYYVRDADIPNGNGTIFVQVRAINLAGSGEWSAKHILGSSCIEAPPVTLPSHLALQ
SSESCVMWPIPEGEVTQYNILYNNTIITLNYTTNWHCFVLNQIQDTITVQVRAVNSAGTG
PYSSVDLKNVLFHCQKPHVSSKRTSEPTLASPHAG*
>Slac_57087 S. lacustris
MDSNNYATLLGPGGVWTAADFDGSGTTWSPGSGGNCQRTLFVCDKSLAPNNWVFTQYVSK
ADNIVANITIFINITVRFSSCNPSCPKILSVYNFIVNK
>Slac_51050 S. lacustris
VLMDSNNYATLLGPGGVWTAADLDGSGITWTPGSGGNCQRTMYMCEKLPTANNWIFTQYI
YKSVSAVANITVYINVTLRFSTCTPACARSLGVYTFITNTPQTTI
>Slac_51281 S. lacustris
LMDSYDYVTLLMVPPPGMGTWKGVDADGDGHYWSQVGGKACRQTPASCAFSYGKQNNWMM
TQHINTTVNGFALQQGAIIARVNFSLSGCLSGASCQQSFFLWLYMASN*
>Scoa_1400 S. coactum
MTAMPSMKLPAAVVLCFVSAIRLATGQQLVDPSLFVANGTRQFVSVSCSATQPATIPANT
RLIYSTAARSFLFTGQFPWTRLNLDGSVGALTQWQGGFGSPCTVGMQVCEESALSARNVV
VSPYLGAVSAFAPAGGLLAVVIDIEFQMTCSVLPPCTYNTVKVLAWPVQAQRQTIDSADV
ASFTSIGLLDGVGRAALNLGDLPRLTERLYYRVPAGTGGLYIAFEAAGACVGIVPVSVSY
CICLAATVGEIVLPQSTSLDVSRRVLGRCAAQRNLVPDVTSHEGNVSVECSRDGVWITAS
VLGQCICDRGYDRPTRSVPCSACRSGTYKPNVGDAVCLICPANSDSSTAALRCGCKVGFY
RDTSSSLPDIDTMACLGPPRQLVASQFTLDNITASSIELSWSATQSDGGRADITYEVSYR
QSNDSSWIPVTGRTGSSLTATLLGLLPFTNYSIRVFTRSGISDEAGLSASDGTELNLIQT
GESTPSLPRSVRLSPLANGVRVHFSEPEQPNGVILGYRVVLMHIGGTARFTRNVTGGALS
VDFFNLTDTQRTYQAEVGAYTSAGFGRFRTSVNRISPGPSDFTPPPPTTTPATTSTAAAE
TDIPSSSIDLSVVVGAVCGGVIFVVVCLIIVIVIVYHRRSRASPKYNVRVSRSASSSTEM
SSPSSASIFRRRSLSARSATQENTNYSPPVLGQLSGTENRQFVDPTIYHDVSQALHDFTQ
EINPAFVVLKKQIGEGEFAYVHRGEVKISNKSSQIVAVKVLKEGADHVIKKDFLMEACIL
GQFQCPNIVALIGICSKDRNRPAMIMTEYMENGSLDHYLQTHDQQFKPNELVAMSLDAAR
GMEYLSGMNFIHRDLAARNILVSITKSCKVSDFGLSREADDENAYEVKHGGKLPIRWTAP
EAIQFRKFTTSSDVWSFGILLWEVMSYAERPYWDWGNSEVMDAITQRKYRLPPPAGCPNV
VHNLMLECWNKERTQRPTFPQLREKIEHMLQSTLPMENIADVQVPDHNAPISPYSSIEDW
LKNLQLSQYTENFTACGYAQVS*
>Snux_6723 S. nux
RDRPGMSVVITLLPLSPNVTNGTFSQTYNLLNTFISRTVTPLSTSVTPPTTQNESLTQTV
KIYLVLAAALCILILIVSCSIIWFIACCLLIKKRILKLYNSYSTKKSTVKYVTVDTDANP
AKPDFFQNPDMFENLKEAVRHFAKEVNNVDIKIDEVIGNGEFGDVCMGSLNQDGHSVVAL
KTLKSDISERHRSDFYGEASVMGQFHHENVITLLGVTLQQPIMIVTPFMSNFSMQQYLID
NSINISLSQQGKMALGVACGMTYLSSISFVHRDLAARNVLLDTDLTPKIADFGLSRETEE
DFYTMSKGGKVAVRWTALEAILRKRFNSASDVWSYGVVLWEIMSFAQQPYGQMEICALVQ
QLQEGYRMSAPLNCPEKVYQLMQSCWLEDPDERPTFPQIHSQLKGLSWVNLEARKSRRLT
GSFQAELLTFSSIGEWLESLNMQKYEENFTKNGYTSLHSVWNLGEHDLVCIGIIPCAHRN
KIMTSIRKANQVINLTESTSV*

>Snux_119736 S. nux
RDCRDASRIYIYESESPLTVESNTLNEFTPVTNSINVTANDGNIRASFSKTKTGFYVGIK
NSGTCTFIRTIQIYYSVCPSMNISQVISTPEIPVPTLGNESL
>Snux_99202 S. nux
MLCSLYTLSILLILSTQDVCATDCTVEAPNNSQVILDSRTYHTLDPNFRSVVIPGWTLLD
VDGQTYSVTGGVITLEWRVLTSAGDCSPHYDVCPAAVITPNIDMEMNWILTSYYITEADN
IQVRIHCASVQAPFIIDCRTAITAQYLESDQNMTDVTPLLSQFKLTQLSINNTFNDGYVY
FSFSKTEAGFYIGFLNSIPCVNIDSILFYYSECPAVTSEYFSVSATLSPNSTQEPLIIPL
TCPTASQAITPVTADCYADGSWILPPASQELCECVSGAEASSSSCTLCPLNSYKMEPGNT
TVCVACPENSNTDGMTGANECVCDVDWYRGVGESVTQSCGMSPSAVRNLRIIRGDVLSAE
WDLPSNLGNRNATSYTVDLLETVSGTTSTDFVISMLEYSLANEVNSDTEYTITVTSRNAI
SSVSGVSPAVSLTFLSSFPDITTSTYTDDYLSWSYTLYANSVYKFQLSYMSIQTGNNVDL
IVNSSSCEQNTDTYVTVCTVLLPNLNTSSAVMLSLLVSSGPNTGLYSGTISIVTDPTFPI
TTNDVIYYIVLPVSGCLIVSILLLMLLSCCVFCCVRHRRRRELVLKTKDSEMLPLHSQLY
QDPSLYEDLNKAVRALAKEIDAEHIEKDSIIGVGEFGDVWKGYLTRNNQQLPVALKILKP
ASTEKNKDDFFKEASVMGQFSHPNVIFLYGVTLKRPIMIVTPFMENGSLDKYLTNHINNI
PFRKQIAICYGASRGMVYLSLLGYVHRDLAARNILLDKDITPKITDFGLSRETEEDFYRV
QTGGKIPVRWTAPEAILYRKFNTASDVWSFGVLMWEVMSFGQVPYGDTDNFTIMEELQKG
YRLPAPDGCPSVIYNLMLNCWNESPENRPTFTNIQESLLQIIETNKARPVSIRMNSQNPL
NHTTLDSWLASIRLEKYVYNFREHGYGQISSIWHLSDADLFNIGIIPAGHRNKIMTS
>Snux_109225 S. nux
KKLTDIVFRMTHMLFLLLISLLYTPTYLSQCNTELPDNSILILDSNTHHTVDGNGVMQPV
AFVGWNIFNVDSIEYLVGNPPNDFNDRLEWTGPTLNTGQCGPTYRACFQSDLVPFATVDT
EAEMNWLVTSYYQTDANVVFIRITCDRISNYEQDCRKTFQILYYESDTSVTVTESLLSQF
LLADNSTNQLMNDEVVVASFYTSKRGFYIGFLNQIPCVELIEFVFYYYNECPATNSSVYS
VPAVQPPTPADESVRVNVSCVLPFVNRQDELLPTAECYWNGTWRLPAGTSGCECTSGFYL
VDVSCVSCPVDTYKRAAGNSIQDCIVCPLRSNTRDLTGGVECECEDEWYRGEGESVNESC
VQSPSAVVNVSVERVSTGDGIDLRVVWDTPTYSGTRDDVSELLYRVSYYKSVSPESEEVV
DELSERQFVLIDAVESTEYVIEVTSLNGVNELSGVYNTVTVSVLSSFPSFDYIRYNSTNR
ILEWSYSLYGNREYVFEYIYTSVESNAVTSAYLNVSECMCAGGYTCVCSVIAQDLYDTLP
ITFRLFSGGLSVLADTDLSYTVTISVIPETPAPPLSFSQLIIIIGAVLSVVLILIMSCVI
LFCVFCLCSRKRSVRSKKSRGDIPLIESMPSTVVQNGHAQMYQDPILYEDLNTAIRSLTK
ELDHKDIVVASVIGDGEFGDVCKGTLKINYRIVPVAIKTLKAHSCDKNKEDFFKEASIMG
QFSHENVIYLYGVTLTKPIMIVTPYMENGSLDKFLVSQDDALNLSDLGKICLGVARGMHY
LSNTGYVHRDLAARNVLIDSDLTPKIADFGLSRITHENVYDVKSGGKIPVRWTAPEAILY
RKFNMASDVWSYGVLMWEVMSYGKPPYGDLDNYTLLEQIQHGYRLEQPDGCPYLLYSLML
RCWDTVPEIRPSFSELFYEVSSMVENNFNPRPKNRFSRSIAFSPLDFTTVDDWLTSLKME
RYIDNFKKNGYTNLSSVWHLSEHDLLSFDIVPVGHRNKIMTSIHKANNKLSRTYSVRV*
>Twil_7820 T. wilhelma
SVVATIDFLLDNNLGQNVHNGSNHPPTRPPKPKKSGKKVTAAKNPLDYQTVDDWLQSIKM
DRYAPLFENNGITSLNQVVQITENDLKEMGITLAGHLHRITESIEAAQIQLSRQPSTRI*
>Twil_13911 T. wilhelma
APLPVMCGAFGCVMYEIWSLGHKPFDSYKNSELVSLIDRGIRLPPPPGTPKPLYTLMIEC
WHPTPFHRPNFSSVVQKLSVPDEKILKWSDEDMAVHPEASKIGADLDTAVELYKNLQDSY
LYSL*
>Xtes_94992 X. testudinaria
MMVSRSLLCLLSALPFILSLAELGITQSVSPGDADSYCLDQSGRIPTSNTEHVLLDTQNY
RSGALVTATGGNGIWLSVDFDEPNSPFHRKWYDNNQGIECKHYLLACGTDQSGQNNWLIT
QYINITDVGVKELTINVTFATDSTCTNCQQSYSISAFQTNEIDETFRANSSAYYDTGVQL
IHTAGQNRYSFRQERFKVTSTGLYLAVVDEGSCTAITRLVVYYDICPYQVKNLVIYPRTV
APSISFSQDIDIVGSCVENASPVSANLQLTCELNGVWDNVDVSCSCNPGYELSNNGFCDG
KYDIFQCSICYIFYTIACLKGYFKLSQGNDSCKKCPENSNSTTSGATNCTCITGYY
>Xtes_94993 X. testudinaria

PGQDNWLITQYINITDVGVKELTINVTFATDSTCTNCLQSYSISTFQTNEINETFRANSS
AYYDTGVRLVHPARQSNISSAQGRFNVTSTGLYLAVVDEGSCTAITRLVVYYDICPYQVK
NLVIYPRTVAPSISFSQDIDIVGSCVENASPVSANLQLTCELNGVWDNVDVSCSCNPGYE
LNNNTSCDACLEGHFKSFQGNDSCKKCPENSNSTTSGATNCTCITGYY
>Xtes_94995 X. testudinaria
RLVHPARQSNISSAQGIFNVTSTGLYLAVVDEGSCTAITRLVVYYDICPYQVKNLVIYPR
TVAPSINFPQDVDIVGSCIENASPLSANVRLHCELNGVWDDVDVSCSCNPGYELSNNGFC
DGKYDIFQCSICYIFYTIACLKGYFKLSQGNDSCKKCPENSNSTTSGATNCTCITGYY
>Xtes_54061 X. testudinaria
VMGQFNHPNVVKLYGIISRVDPVMIVMEYLEKGSLDRHLQRNLDKLGIPTLLKLAHGVAS
GMYYLSKIGFVHRDLAARNVLVAKDDTCKVADFGLARELVDNEYDVQKGGRIPVRWTAPE
AISHRSFTTASDVWSYGVLLWEIMSYGETPYEGWDNYSIFQRLENGERLRQPQNCPDDVF
QIMLNCWNAERSERPNFEELVNRMEILAYGKPLLKPKPHNLQSPNRVSPRPPPPGSHSVE
TIENLLHEIGMSRYIENFHKHELYNVSDCFGFTEEDLEKIGVLLGGHQYKIFKNLRIKEE
ELGRLSP*
>Aque_03862 A. queenslandica
MSTLFCFFLLLLGSLNIFQCVSSQSVAPDDADPDCSDIPIRNVTFPNERILLDSQNYEST
LLVGAEGENRTWLTIDYDGGGVGRPGAAGFVDSWYEGNQGTQCLHRINVCATLIPGGTSS
FQSNWLVTQYINISEPGISQLRINVTFTSNLVCSSQQCAQQQTFEVQTYETNEPDETNKG
NISYYSYAGVRLIHTAEEGATTDSESFAVSSSGLYLAIWDQGSCTVINRIVIFYNICPYQ
VLNKTIYPETVAPQGSFDPDKNVIATCIDNASPTSSNSLSLRCRLLGVWIGSASCQCNAG
YEANGTVCQACQEGTYKSVAGDSECNGCPNNSYSASPGAVSCQCLSGYYRTPQEGVAVNC
TAPPSSPQNLTNTSVTKTNISISWLEPTYNGGRTDLYYVISIKSSTNIIEYSTGHTNYKL
MGLTPFTTYEIQVMARNGVSDQDNANDNTRT
>Aque_05648 A. queenslandica
MVSFSSNLVCSSQQCAQQQTFEVQTFETNELNDINRGNISYYSYAGVRLIHTAKEGATTD
SESFAVSSSGLYLAIWDQGSCTVINRIVVFYNVCPYQVLNKAIYPETVAPQGGFDPDKNV
NATCIDNASPTSSNSLSISCRILGLWIGSASCQCNTGYEANGTVCQACQEGTYKPFTGDS
ECNGCPDNSYSASPGSVSCQCLSDYYRAPHEGVAVSCTAPPSSPQNLTNTSVTKTIINIS
WLEPAYTGGRIDLYYVISINSSINVIEYSTGHTNYTLMGLTPFTTYEIQVIARNGVSDQD
NANDSSRTVTITVTTLNPCNETCSSGFYLNPDGCTCGSCGVSNCLRCQLLNSSCCTQCQL
GYEINDCECEAASSSSSVDVYESVSSSLDSSVTNPPSTETPQLSSSTLTIVAPVVFVLIL
CVCIIIVFVIVIIVRRNRKKKFVNLDLCHVDDFRISLDLNSAYQKVHHQNEETSTDDTVM
KQKLIKYLIPSSQIDIQETIGQGEFGIVYKATMKATNKSQQEIALKTLK
>Aque_11657 A. queenslandica
MESISLFILRLSFPLLLVLSNGVLSSAPGDTGGYCLSNSALPDYPQTVKRLMDSWSYGLL
LGPGGVWSTIDADNDNKTWSPVPLETCRERVSISEPQNLPQDNWLITQYINKSIPSQYNN
EPVTIFINISYILDASCASVNCIPNFQLLYHLSNASDSSLASHRPGYSLISNVSSVDDVT
LHMKTFSFNLSPADTGFYIAIRDIGANITITRLFVYRLIGPAMQSGLTLFPETPAPTTDC
INITGLCVANANTSTGSLPVLSLNINGSWSYDDVCMCIPGHELMLIEGTNQCQACAPSTY
KSTLSNTVCTSCPPNSDSTVHGSSVCQCDSGFYRAPNDGPSMPCTGPPSAPVGILVNVTT
STYSLISWGTPASTGGRTDVYYDIYYYSTANATRVKANPQPLYATSYNFTGLSPLTTYLV
TVSAENGVSNQSSSTQRSIFIAITTPEGVPMPVVGVKVLSGGQYVEWNVSSSAHGVITMY
EVVFYADLSDPSRRGRYIPVPSDVTVYQVNITRDFPSSGQPVYVSVRAYTQVGPGDWSEP
IVFSQTNSLAPTQKSPSSTTSIKIIATVTLSTTSGLATTSVSSSSQIPGTTDQSTTTPTA
TPTESGSNPVVSIVAVLGGAIGILLIVTLGAVGYFIFRKLRLKYMAKEETDHHVPLVKYE
VVSGEEEENRVQINPLAEDS*
>Aque_12969 A. queenslandica
MHVHQISCLDQLVNLKWACFRGNALTDIQGIESCHKLEELTLDDNYLQNCLSLHRFPHLR
WLSLENNHLTSLPLSSSPLPQLTYINISRNYIRDITGIETLTSLQEFYASHNYLENLRQL
FSLKPLVHLVAIDLTCNPLASHSAHYRLFCIYHFPTIKAIDARAVTNTEIGLAKEKLGGR
LSQDLVYEKCHSNDFNAIKKLDFVQCGLKHVDLSPVSNLDNLVSLNLEHNSLTSFSGLIH
LSKLKVLCLNHNRVEYLIRPTGSGPSSTSSGGGGGGGGDPVMQNLQVLHLAYNGINSLVP

LQLYRLPGLRALFLQGNEISRIEGLDGLHHLTELVLDRNKIKCMQENSLQHLVTLKEFHL
EENRLSDLSHFESVKNLERLYLGMNRIQDYSELEKLGCLSYLIELSIISNPISRRMQHRS
LLIYKLPSLQSLDGVMVTSDERQTAEATFSDRCQHQDEDQADTALLPSLTNAGQVKVMNV
PLYMSTHTSRQSQAMEARALHLQTLQAFQDRSLSFFQCVSSQSVAPDDADPDCLDIPIRN
VTFPNEKILMDSQNYRSTLLVGAEGENRTWLTIDYDGGGVGRPGAGEFVDSWYEGNQGVQ
CLHRLHVCATLIPGGTSSFQSNWLITQYINISEPGISQLRINVTFSSSLVCSSQQCAQQQ
TFEVQTYETNESDDMNRGNISFYSYAGVRLIHTAEEGATTDSESFAVSSSGLYLAIWDQG
SCTVINRIVVFYNICPYQILNKAVYPETVAPQGVFDPDKNVNAACIDNASPASSNSLSLS
CRLLGVWGGSASCQCNPGYEANGTVCQACEEGTYKPFTGDSECNGCPDNSYSASPGSVSC
QCLPGYYRAPHEGIAVSCTVAPYDTNEKCDKSNSSSNVTILLDTNHYDATDLLGENAQNG
AWVSINETWKEVSMNKEECRHNLTVCKDIHPQQYSNWLVTQFINISDSRVEKLSINVTYS
TNCQTEDCQQSNSSFSIWIHETDTVDNVDQNDISLYQYTRLDFPEDDMDNSTTVNALFDV
SKSGLYLALVDMTSCTTLYRLVIYYDDNLLECKCSITSSESTTFTTTTQEKSLVITFTSV
SEASTMIQMSIHSTTQPKYHIGSSSSVDVYESVSSSLDSSMTNLPSTETPQLLSLTLMIV
APVVFVLVLCVCIIIVFVIVIIVRRNRKKKLVKLDLRCVGK*
>Aque_16689 A. queenslandica
MISFCYSLILSFLSLSVTVATAQQNISVAPGDADPYCLDIPIRNVTFPNEKILLDSQNYR
SLLLVGAEGENRTWLTIDYDGGGSGRGEVAFADAWFESNQGVQCLYRINVCANLIPEGSS
SFQSNWLITQYINISEPGLNQLRINITFSSNLQCENQQCGQQTFEVQTYETNEPDEMNRG
NISYYSYAGVRLIQTAVEGATTDSETFAVSSSAIYPETVAPQGNFDPDKNVNATCIDNAS
PATPNSVSIKCRLLGVWIGSASCECNPGYEANDTACQACPAGPYKYDTDTDCSDCPANSD
SVHIGSSYCQCSNGHYRAAYETVNMDCTAPPGPPHSFSFSFTSTSINITWGPPINNGNRS
DIFYHVKVMDQSNGNFVNFYNTSNTFYQLTGLTPLTTYTITITSNNGVSDQDMSNELGRQ
VSINVKTTSSPPSSPQSLILQSSPTGQPTVLTWSEPQYPYGVIICYNILVSKFLDSDTAI
VRGSVNGTTLRYDLNQIDLVSGHYFISVGTSI*
>Aque_32612 A. queenslandica
MATLFCSSLLLLGSLSFFQCVSSQSVAPDDAEPFCSDIPIRNVTFPNERILLDSQNYRSA
FLVGAEGENRTWLTIDYDGGGVGRPGAAEFVDTWYEGNQGVQCLHRINVCAALIPGGTSS
FQSNWLITQYINISEPGISQLRVNLTFSSNLVCSSQQCGQQQTFEVQTFDTDKPDDINRG
NISYYSYAGVRLIHTAEEGATTDSESFAVSSSGLYLAIWDQGSCTVINRIVVFYNVCPYQ
VLNKAVYPETVAPQGGFDPDKNVIATCIDNASPTSSNSLSISCRILGLWIGSASCQCNAG
YEANGTVCQACEEGTYKPFAGDSDCNGCPNNSYSASPGSVSCQCLSGYYRAPHEGIAVNC
TAPPSSPQNLTNTSVAETIISISWLEPAYNGGRTDLYYVISINSSISLMEYSTVHTNYTL
MGLTPFTTYEIQVMARNGVSDQDNANDDNRAVTVTVTTLNPCNETCSSGFYLNPDGCTCG
SCGVSNCLRCQLLNSSCCTECQLGYEINGCECEAAVGPVSSSLDSSVTNLPSTETPQLSS
SMLTIVAPVVLVLVLCVCIIIVFVIVIIVRRNRKKKLVNLDLHCVDDSRISLDLNSAYQK
VNHQNEETSTDDTVMKQRLIKYLIPSSQIEIQETIGQGEFGIVYKATMKESQQEIALKTL
KGPFSKSDVDCLLEECLLMSNFDNPNVLSLIGVCADLGSAPGIIMPYMSKGSLLSYLQKE
KSYLIVEASSEESTVLNDEDQADTALLPSLTNAGQVMNVPLYMSTHTSRQSQAMEARALH
LQTLQAFQDRSLSFFQCVSSQSVAPDDADPHCSDIPIRNVTFPNERILLDSQNYRSTLLV
GAEGENRTWLTIDYDGGGVGRPGASEFVDSWYEGNQGVQCLHRINVCATLIPGGTSSFQS
NWLITQFINISEPGISKLKINVTFSSNLVCSSQQCGQQQTLEVQTFETDEPDDMNRGNIS
YYSYAGAHLIHTAVERTTTDSESFAVSSSGLYLAIWDQGSCTVINRIVVFYNVCPYQILN
KAIYPETVAPQGGFDPDKIVNATCIDNASPTSSNSLSLKCRLLGVWDGSASCQCNAGYEA
NGTVCEACQEGTYKPFAGDSECKDCPENSYSASPGSVSCQCLPGYYRTLYEGITVSCTGK
*
>Aque_32613 A. queenslandica
MDSITQEFADWKQDTSGYHVYYTLFFTFTAWQFKFLSISRRMQHRSLLIYKLPSLQSLDG
VMVTSDERQTAEATFSDRCQHQDEDQTDTALLPSLTNAGQVKVMNVPLYMSTHTSRQSRA
MEARALLLQTLQAFQDRSVAPDDADPHCSDIPIRNVTFPNERILLDSQNYRSTLLVGSEG
ENRTWLTIDYDGGGVGRPGAAEFVDSWYDANQGVQCLHKINVCASLIPGGTSSFQSNWMV
TQYINISEPGISQLRINVTFSSNLVCSSQQCAQQQTFEVQTFETNELDDMNRGNISYYSY
AGVRLIHTAEEGATTDSESFAVSSSGLYLAIWDQGSCTVINRIVVFYNVCPYQVLNKAVY

PETVAPQGGFDPDKN
>Aque_37706   A. queenslandica
MPALFCISLLLLGSLSFFQCVSSQSVAPDDADPDCSDIPIRNVTFPNERILLDSQNYRST
FLVGAEGENRTWLTIDYDGGGSTRPGAGGFVDAWYEGNQGVQCLHRINICATLIPGGSSL
PQSNWLVTQYINISEPGISQLRINVTFSSNLICSNQQCAQQQALEVQTFETNKPDEMDRG
NITYYSYAGVRLIHTAEEGATTDSESFAVSSSGLYLAIWDQGSCTVINRIVIFYNVCPYQ
VLNKTIYPETVAPQGGFDPDKNVNATCIDNASPTSSNSLSISCRLLGVWIGSASCQCNPG
YEANGTVCQACQEGTYKPFAGDSDCKICPNNSYSASPGSVSCQCLSGYYRAAHEGVAVSC
T
>Aque_37707   A. queenslandica
MELSDQDNANDNNRTVTITVTTHPTVPSHPRNVRLSEGSLLTWSDPMNLFGSELVEYLVL
SNPLNDTNSAVVRVRLPPTANSYDLTDLNVPNGFHYIWVRAVSKIGEGEFSTSVLYNRSN
SFMNPTLPTVNNEPLILSVSLGALALIVFIVLVNALLWIVLVIKRKRKQKRKKEQSAYID
STLNPSYQMTTARSPPTTMLSSPITQDNVAYNEIAAIPHLNCLNPNEKFYEEIDDIAVDT
AGTATAAAPSTTPTYWESVAPYDANEKCYNSTSSSNLTILLDTNHYDVMDLLGKNSTWVS
INETWKEMSMNQECRHNLTVCHSRSQHSNWLVTQFINISDSRVEKLSINVTYSTNCQTKD
CQQSNSSFSIWIYETDTVDNVDQNDTSLYQYTGLDFPEADMDNSTTVNASFDISKSGLYL
ALVDMTSCTTLYRLVIYYDDNLLECKCSITSSESTTFTTTTQEKSLVITFTSVSGTSTMT
QMSIDSTTQPTSSVTNGTTEPEDDNVTTVIAITAGFVIVTVILIIIVIINVFLSLAVLKR
RKTHAQQNVTSKDVFLASYDPYQEQANDGEEEYDYDKKEGVDDVKTFTVEETAIVEPGEN
TEECEHN*
>Aque_41463   A. queenslandica
MAAVLKPAMSRFLLLSFLLSTVAAQSIAPDDADPTCSDIPIRNVTFPNEKILLDSQNYRS
VFLVGAEGENRTWLTIDYDGGGVGRTGAAGFVDSWYEGNQGVQCLHRINVCATLIPGGTS
SFQSNWLITQYINISEPGISQLRINVTFSSNLVCLNQQCPQQQTFEVQTFETNEPDEMDR
GNISYYSYAGVRLIHTAVEGATTESESFAVSSSGLYLAIWDQGSCTVINRIVVFYNVCPY
QILNKAIYPETVAPQGDFDPDKNVDATCIDNASPTSSNILSLRCRLLGVWIGSASCQCNP
GYEANDTVCEACPTGTYKKSPGDTSCDDCPDNSDSLNTGSTSCQCLPGYYRAAHETDDVA
CTAPPGPPVSLRNTSFNSTSISIAWDPPVSNGSRSDINYLVSITDQSNGNSIVPHTTNTT
IYKRGGLTPLTTYTITVTSRNGVSDQDLSNETNRQVSINVTTMSSPPTSPQSLSLQRPTP
SGQSTILGWSEPQNSYGTVIRYNIFVSEFNDTDTATVRGTVNGTTFRYDLSQLDLTSGSY
FVWVQAVTIHGESDLSPPIQYLHVEAPTPTDSSTSSDLFPVVAGASIGVVLVVIIVVAIV
ILLFVICFYTNTRRKSTYSEYGSRVANSGYNHDESDTNIELGTLPPPSNNPNRMYIDPRT
YASMNHAIEQNRVKEIPPKEIANLEEIGVGEFGLVYKGVWTLKNIPVAVKTLRGGATDEQ
RSNFLFEASVMGQFHHDNVVVLYGVISRIDPVMIVMEYLQNGSLDRYLQKNLDKISHERL
SKMSYGVAKGMKYLSSIGFVHRDLAARNILVANDETCKVADFGLAREMVENEYDVQKGGR
IPVRWTAPEAISHRSFTTASDVWSYGVLLWETFSFGETPYDGWDNATIFQRLENGERLHP
PRGCPDDLYKIMQACWNIERSERPTFENIVECFEVILFGKPKPKPKPAHLQSPNRPIPPT
TSNSSAPNGNNKAADSIDELLGSIGMTRYIDIFHAKGLYNVADCFGLSEDDLQRMGVTLS
GHQYKIVKNIHLKEEELGRVSPDFI*

Proto Eph-like in unicellular holozoans:
First four sequences contain Ephrin binding (Eph_LBD) domain, these are possibly true Eph receptors

>m.8217_Salpingoeca_urceolata g.8217 ORF g.8217 m.8217 type:3prime_partial len:328 (-) comp3171_c0_seq1:3-986(-)
MAESCLFSLRNIVSAAAIVAIVLAAASPAVAGTDILEEEHFSSNFDGSDWICTDDPSVLT
PCSSSVCEEEWCWKFINSGGIGKNGMEACKRSTNNDAVLSNDATKWLATPVIDVTGLVQV
EISFRFDKTPCASNTCDNTFAMYALPVDGGVTDFDGKIDIDTGISKNAGQLAFQTENHVL
AVSSVPSSHIRIAWFDVGGCVQLDDVVIQYSYCAAQTINGVSYPRTLSDDSKTVTSTGTC
ASGFPQDSSFVCEDDGDWLQVGTSPFCSATPPNPNSGGSGGGGGSNTAVIGGVVAGVLVI
VLVVGGLWWRRTHGGGGGGGGYGGHGKP

>m.240923_Hartaetosiga_gracilis g.240923 ORF g.240923 m.240923 type:5prime_partial len:1023 (+)
comp16549_c0_seq1:1-3069(+)
CLSFIPLFIIVFLKMGRLCYQQQLPFLLLLVAVALLSKRILAGQLNRIVKDTIIPIVPPN
TWFCVDLGDEKGTDPKYIENTCGKDMGDEWGFGDEPDEAETSFQSCSSSLSSKFETILVF
PELNVTGLSKVEVELNVTLDPPPLNGCGSSDCLFFYAVDTRAINYNWGDGSFDVGTEIHD
QLNDMALFSTPSSFMSFFNLKTEVNADMRLFTLNFNQNVDSLFLFLVDTGECTTFDGATV
YAYVCEMTSFEQQGAWFPQRFPSSDVVEGTCLPGWNESTNGKPTARCSDGNGEFGYFFES
NPCVNPNVTTTSATTTIPPATSLSTSTPTSTPTVAVTPGSSGALSSGAAAGIAVGVILLI
IIVAVLLFLQLRSGKKTAPVYLHQPHIPMTPNDLYHSKVNTQYNQLLYSPPSQSDSTMYE
DPSVYEDPSDLKTHLISCGRIVEQEDVQFIAQIGQGEFGEVYHAMVTFNKGEAPLDCAIK
TIRPRSSKEDQLSFFKEAAIMIQFHHENVIGLLGIVFHQDSAMIISELMTNGSLLSYLSK
HINKLTLTEQIKCAMDIARGMSYLSGKGFIHRDLAARNVLVSAEYVCKIADFGLSQEIDD
DGDYFVSEGGKIPIKWTSPEAIHDHKYTSASDVWSYGIVLWEIMTYGMKPYPGMSNFVVM
KKLEDGYRMPQPEGCPAAIYDVMAACWEEAWRDRPEFSAIVNALHALYSSADRGEELKVM
SLQDQYVAPSSHRSSGLDYSVPLTEDIVEDYDLPNQSDMEEGEGMANAQQAQHEIISHSL
PMQNSVQSVHNQEQSNGDYIPLEQQKGVDLQQQRRLGEYADPTAQESETVVVDSYADPQD
SVPADHGVHKPKPPQRTASLPPADTYADPQDAIQDNAKTPQQSEQGEHNQNQQGTKQEPS
NEYADPQDSLSMQPQPQSKPKPKPRPKPRSKRLKPSPPSTDSSDYAQPQDSISTTPSGIE
TPNSRPQQLQQQQEPTDNYADPQDALPITTHPSLPHKKLPTPPPNQHTSLPTSDYTDPQD
AL*
>m.108007_Microstomoeca_roanoka g.108007 ORF g.108007 m.108007 type:3prime_partial len:775 (+)
comp15202_c2_seq1:119-2446(+)
MKQLSASSTAVVVLFALLRFVCLQAEAQSTFVERVTPILLSSNWTCTEADGSFVAFNPCL
TSSTGIDQAWFLNTMENSSLFEVCPQLKVNRDRTQFNVLISPSIPVMRDTSVVIEVHASV
DNNLCPMGACDSNLGLIAFPTIRFNELFRNGTDAQIIELYPDAVMKLKSITTSPSLGHVA
RFVLSSETRYIINGLQSVDEITLVFVDQGTCSKVNSVALVREVCEPATLNHARFPEAKKD
LTVDGSCVDGFFSPSGNPRAVCLTSGFLLVQDVCRPITTTMRSTTVNMTTLPDSLQSKGK
SSISPALTAGIVLISLLVLISASAGIYFLIARRGTNKTNPAYELQPHPSRLSAMYRVNLN
TEYKSIKDPQYSPADSGMYEDPSLYADFSQLSSMISKKTRQIPMASVALITCIGSGEFGE
VYHAVVTFSDQELDCAVKTLREGSTRDDMLEFLKEAAIMCQFNHPNVIGLLGVVLDQTPN
MIVTELMTNGSLLSYVAKHSDTLDVQRQLAMALDIALGMSYLSQKGFVHRDLAARNILVT
ADLCCKVADFGLSKEIDDSSDYFISDGGKIPIKWTAPEAIQQRKYTTSSDVWSFGIVVWE
IMAFGEKPYGNMNNFEVVRKVEEGYRLEAPAKCPDAVHELMCLCWEIDRRQRPTFSSLVT
MLENLRSDANNGSKLVLKNDSRIYQLPDNNQATMNNYDMPLAEHKDIANVSLTFAQNPLH
ASPRTTINFQPNYGQPTTDTTANRFVTQQNYAQPQDTVQQNYGQPQDTVQQNYGQ

>EGD75115 pep:novel supercontig:Proterospongia_sp_ATCC50818:supercont1.17:315855:321267:-1 gene:PTSG_06770
transcript:EGD75115 description: TK protein kinase Salpingoeca_rosetta
MMMCRGVVVLVTVVSLFLGLCIQSQPTAAQELIQKDFDSVTLNRTEWLCYNADSTASLRL
LQSCPLTVSEHAWSAEIPSGFATCSSAAQPANAQQFNVLVSPPISVPDDLYVSFRIDFTQ
NNTLCPDGACDSQLLVAVVDTAVLENNLPMAADYNLYVVDNDLATFFSDMASSGTNSLAR
MSLLESSIMSPRLRRSVTEVSLVFFDRGTCSTVANVTVVRTRCLPTAELFNATLPGTDLG
RAASGTCNAGFASADTVLASCEEDGYKLLSGDCAPAMTTAAPAGTTTDPNGGSSSSSTGG
LEPGPIAGIALGVLLLILGGVAAGFFLRSGGGSNRKTAPALYELEAKAPRMSGMYRVNLN
LQYNQFDHGAGADDGMYEDPSKYEDFSQLTSLVSRKTRHIPSDSVTLIACVGSGEFGEVY
HAAIKFDPAKPEEVDCAVKTLREGSTRDDQIEFLKEAAIMCQFNHPNVIGLLGVVLDKTP
NMIVTELMPHGSLLSYLQKHTDTPLTRQLQMSLDVALGMSYLSKKGFVHRDLAARNILVA
NDLTCKVADFGLSKEIDDSSDYFISEGGKVPIKWTAPEAIQQRKYTSSSDVWSFGVVMWE
IMAFGEKPYGTMNNFEVVRKVEEGYRMDAPANCPAPVHTLMHMCWELDRRQRPTFEAIVD
MLEKMTREATNNVKLEEPGRSRTYERPESGMDYDVPRREKTATAATAAAAGVAMQPPQHG
QQQQQQQQVYQPTYQLPQDDVKQQQQQAQHQHQQTYQPTYQLPQDNVHQQQAQQQQQQQQ
QVYQPTYQLPQDNVGRQQGGGTTYIPQPQLSQQQSQQTYQPTYQLPQDTMQRQQQQQHGG
PTYVPQPASSHTPANQMTYQLQPNTTAQPASTSSAMLPPSDPAPSRPRPKPRPKPRSRQS

STRPPHAETDI

<u>These remaining proto Eph-like sequences lacks Eph_LBD but are the closest hits to the metazoan Eph-receptors (These are not true Eph-receptors, nonetheless the sequences are provided here)</u>

>EGD72856 pep:novel supercontig:Proterospongia_sp_ATCC50818:supercont1.10:1297594:1308890:-1 gene:PTSG_04584 transcript:EGD72856 description: TK/RTKC protein kinase Salpingoeca_rosetta
MATASTTVMLALVLPVVLLACAGPSKAQDWRVPLPSQDTPYDDLFHVGPVMSFTESIVYC
SDPEYQDRTIARLDDPAQVEQLNVLRYTRGTFPTSGTGGNSPSTGGGSYFWDCANLRECT
DTQYETLAPTATSNRECAELTVCASGEEYETVAATATSDRQCQPLTECSDIQYESRAPTA
TTDRGCSDLTFCATETEVQTTAPTATSDRECEPMEDCVRGNTYRGEERPDDWGEDDPTVD
ANEPEPLLFTTTGCRRITKCSGNEYQSRPHTVYHTAICMRVSRCESDEYEPRPATDTTDT
LCLKYSQCVDGVEYEARPPTDTTDRDCQPIADECSAEDGFYEAAAPTATSNRVCKPFMRR
PEFALGTGTSLSPSLSLLFNNESLSSAIAPVAESADTTSFSAVVAASSPDGLGVNVRVRN
QGYLTAYDTFMLPDEYGYQEDSNVPLAVAWSAANGQATISLTQSIADVTPPRRAYVLVLE
AADTRSTCILSSTSTLPGPCTAAFLLVVPVIATVDCAGAQHYVLTGNATTADMEWTPLRQ
RLLRASPLVHYSHTLETAPGNTFDNNTYSQGVFVHSFYSSATVQPPSWCLVQVTVLPGVT
LTISSVGHVFLPQSSSGGSRAISLDYIIDQQQISGAGLQVNALYGAILPLHGFGLVLQPP
DGESFSFQVADANTEASVSTRLEFCQQPGLTEWEDDAIQVEAQTVFHYTGNGQRQVDLSA
SSALIAADGQCFRFTLTTAALTQPFQLERLVFVLNGAAASSDTPISRDVYSTFYSKSQNF
VVYKTDHSSSVSTTDPGAAVTIQDITPPTFENCPTVVSVRADRNSATAVATWTEPTAVDN
VEVRSVERSHAPGAEFSVTDSPHEVTYTAQDTSGNTGTCTFLVLVDYDHVTLPSAFNVRF
TDITSVEVGSVPLGGNVVQSVYADQILAPGNRAPLFYAVTGVVPETNTLSVGLQPSTGDA
FALVFGEQEVRARFVIDITLVRLTDEQAADVPPSTTTTPTTTTTTTTITTTTGTFTGTT
RPGSSPTTPSDAPPTTASTGTTTTTEAPLVLQDALPFEQNENEFVSAALRLGNLSLPNSL
QFGDGGPNNIMQSDGAGNSGPFFRNENGDVILEFQSSADSTIKFNTTTNDVETIRVRGTS
RIFTQELSFSGVNVLLQYPGLFSLADARRRRRSHGGSHEPATFGLTPDSSVRVEYLFLSS
DEQGSTPTRTDGFLTFEDDVPPTYGNTCPDNITAIAPADASAANVTWVEPHATDNRRVVS
DVGSLTPPVLLPIKTPSDPDYQVVYEAMDQFGATATCVFYVRVVDADAPVVTCPPSQLLN
ADETTGVGVLDASLVEPPRVVDRGRVYTPPDNLQATFDVETAQSTYAIGVTQAQISVTDD
WGNAGTCTFEVEVVDVTPPSALNCPSVGPSAVSDDGSDVAASWTDVTWTDNDGPPTVEAS
HTTGDLFPIGVTLVSIVATDASGNSAACEFNVTVVAAGSGTGTGGSAAGSLSTAGIVGVA
VAVVAIIIVLCVVIAVLIVRARKRPHDFEAILEEMRQLKALTGFDTAKGEGPVKPRELKR
SFVKILDVLGKGNFGNVCKGLLNEPGRPQYIVAIKTLHEEAGDIGRRELLQESAVMAQFQ
HPNVVGLVGVVTAGEPMLMVIEFCELGALNAYLAANDPPLDMKYRFAVDCAAGLAYLASR
GFVHRDIAARNVLLSSEERCKISDFGLSRETEHSDYYHSRKGGALPVRWTAPEALENQKF
SSASDCWSFGILIYEIWTRAARPYEGWSNQKVWAKVTCGYRLPRPRKCPKEVYHLMLLCW
HHDPFQRPQFDELRRAFESLRSGVPNGSPLLEEPLSPVPNLVPNERAETRFNSYQTEGRS
VSPAGTAATASTNMTGTTSASSNGTKYVALEKQQRSDILYPTQPDGNPPSTRTPTTLDNS
TDGTTVCVLDSSSPVPFKQYTPAGQPRRSVVYDLTRSEDGDSVVDVDVHGHPPVIDPGPA
RPSTEGLYDAGENTANPYAPDDYEKGGRRPTDYTRATPQLGDDDDDGDDGHEYAGEMLSG
NAQSGRASPRLPVSEYEDAMAAHTLQDYEDTAMPADMMAETVFAENGTSGTHYGSQQQQQ
QQQQQQQGGRMRGIGGGGQEVSFIRRRHDTYDGTEVYNVAEHDYAEPDEDAKEGYLSVEG
DDSSTRAVTRRNTDMSEPLTPVLPPQLPPVHEYEDAELVQGNGEYENTNVLGMHDGDATQ
QRQQSNYEDAKMKRRSADFHEYLQPNTPG

>cds.Acanthoeca_spectabilis_TRI_1_14_NORM_comp65185_c0_seq11|m.158325 Acanthoeca_spectabilis_TRI_1_14_NORM_comp65185_c0_seq11|g.158325 ORF Acanthoeca_spectabilis_TRI_1_14_NORM_comp65185_c0_seq11|g.158325 Acanthoeca_spectabilis_TRI_1_14_NORM_comp65185_c0_seq11|m.158325 type:complete len:1447 (-) Acanthoeca_spectabilis_TRI_1_14_NORM_comp65185_c0_seq11:745-5085(-)
MAQTTFVTRVLAWKPQRVNAQCAYNVSAIDSADSCDQALGLNTTSPFSLLTGSTLPPNHV
CSNESSCLAFYGAATTTCCISDEPILNLEPVASEPDRANTSLLTPLNTNLTVGVAYGIQP

PDTWTPYLSQGCGAESNLRLSVTVSGPPVVPPFRPSDEVLMNAETGFMFVRLSRAYSATV
TIVVSSDAGAVVTNVNVASWTFSGYDPDVTEVSVASHPTTAHPEYGPGQRDCENGQRVDG
VRYDRHFTCDCAGTAFVGPRCELSSRPRLSLETNYGQIIPVNDSASDYEFYNRTKWAFGK
TYSIAPFNLSRAYTTRPGVGRVYVPATSLSYGLTFFGVVQEIRGFFVDGNNGEMLVKIPR
TRLNLSAFLLVDAPNALPATAANLTFQILPADVDNAQAQGPGGMGCQNGGTKTDVEDGES
EFDLQYACSCRSGYSGANCEIDVAAAASGGNSGSAWAFAAIGVIVGVLFVVLVVSRYLVY
RAQNKPEDMLAMQAEILSSLGMGGVSMNVAPDEMGFRLSFNESVGQAVALQSHNLDDTFE
KEILECLRRLSGLPSRLVRLLRDPHTKVTMDVDDSCALLRVKKPRQEVPPGLQETYAAAL
NAHAEKRNISLREHVVREVSVAVPVHVPREIDRHAVIRLGLLGEGNFGEVFKASISIGGV
PLAAAIKTLKATDGEGRGELLREAALMALFKHPNVVLLIGVVTVPRNMPALLVVEYCEEG
MLLDRVRNASVKSVTSMLLTYCRDVSCGMHYLSSRRIVHRDLAARNVLLDSGFNCKISDF
GMSTALGHNDSSDYASNYVRLRGELPVRWSAIEVLQAGKYSRASDVWAFGILVYEVMSGG
EQPYGDAPNLAEVAERIKARKRMKCPEGCPPAVYKRVMLACWRTDPVNRPGFSQLSDILE
DLGASKSDAEAGKSATLMKQQSEDDAGTWELGLKDRRLIGPSIYHVDQVLAPAVIQCVQP
PWKDSNGRLVEPPSSATIAHTVHAVVKPKSKNVPCPRDGQRGCAYVDTLSAQDDVGRATA
LLSYTWGYKVASVGSALQRWAEQESRNPKRTYIWICSLCLNQHRLLKAVTPEELANEFGP
RVTTIGRILPMLEPWFEPQYLSRAWCLFELYTAIRKRGKVQIEVILTEAEYHSFMNAMAT
QGYSCIDQAFQSIHSEAATASQPADLQAIQTLIRNKPGGFEKLNATVKQHLGRWFESHGA
IKSSSRVAHSRDSQSGSLASVSEMSNGSEFQDVSMQNVNKDSKRGSRTRSLWKGRASRSR
KGRTYSVGESAKAAKSPHPASKATARATSDRTELSIVSNGLSQVGNELLGSTPVTSAPSV
RNEATTVPLHSNVTVPDFREVENDPDFKEVEDGYLDVSPEFSVGDRCVVDSRGPGTIMFN
GLIKGQHRFGIALDKPNGLNDGSAFGKKYFDCQPHHGLFCKAQQVSEETVLLPGPVQSGE
EFGGFV*

>cds.Acanthoeca_spectabilis_TRI_1_14_NORM_comp64141_c0_seq1|m.146412
Acanthoeca_spectabilis_TRI_1_14_NORM_comp64141_c0_seq1|g.146412 ORF
Acanthoeca_spectabilis_TRI_1_14_NORM_comp64141_c0_seq1|g.146412
Acanthoeca_spectabilis_TRI_1_14_NORM_comp64141_c0_seq1|m.146412 type:complete len:227 (-)
Acanthoeca_spectabilis_TRI_1_14_NORM_comp64141_c0_seq1:609-1289(-)
MPCPLQVYAELEETGGLDVNASRSVKSRATDAVKDGSHLGPGTVRKLVSEGEGDDVTFPK
EFKRKDVTIENVLGKGQFGEVCQGYFSMTVGHVKTQHPVAIKTITDTSADAQKAFNEEAA
VTWIFQHPNVVGMFGVVTTGLPRLLVLELCENGELLKYVKSKNTDQSLKRLLHIVKDIAS
GMRYLASKHFVHRDLAARNVLLDRNYQAKVCDFGLGRNFKDDEYYR*

Monosiga brevicollis

>jgi|Monbr1|26435|fgenesh2_pg.scaffold_14000101
MQMTECCPCNTITCRVCVCTLVRLSLSLSLDQWATRNSIVLLSLSLFGSGSETGSASLLIAACMQSLSLS
LCASLFFLSSSLGRHHHPRPAPLLRNKQVLELCRSLRRGSVRAASQIGPPRSQTGSVCRFGTALPSSHNQ
SSSTSTINLDLRLEQSADTVVDLSSLPLNVTFLLPSCSYFRFDNTSFIPHLAFQPTTLPNTWTWLSTGAI
RLQLVDPGIVNPEPNASLVLVLYTLNPASVASSCPECDADPAACDAALRATSVTIRLQNVLTPDPTPDAN
ALVNASCLNASDSVTCPLCPPGRYTPDLQTCIAGSPCPAGTEPYLPASQQADTICQPCVPGFSASPDLDS
PCVLLAPCLASTESFAFDPSAPVNDPVAAQDAVCHSRPDRLPDLDVQILGRGAHPVLASGNTQAPHALVA
RIAMLASLPSALPVAFNITVDSSAGDRLYTVLLPQEPSPFTPFSVPLWPNATIHIRAVLDDDAPPIPAPI
TTLVESDLNAAPAGNLTFDHGSVSRVLLTVDNCSTLANASYTLELESLHTSTVLEVPLNANPCNVARKFV
APTLADHSYWVRLVATRLAAPDNDDPSSTTNPDPNATSTTTPVPSTSVVEVVGAQYFRPSTSLAARLATF
NWSLDLTPVLPLSTSGQAVVGWDLSLLTGNLYNDQCRAMRTTLQATRLDADGGSEEDTMTLAALTQQTCH
DLLRPRAITFGVESSATYAITATITLNESSVVNDSATLETLLSAAPAVSNVVLSTTTTTATITWSLASDD
NRARAIDLSYTSSQSNNAGTLTLPTSANNLTLTQLWPNETVTITLQTRGNCLACASTPVVVRAATQPMPN
LVVTDIVATNVTFDAIALAWQPPSLNEEDGVEHAYLRLTVRTMFFDFVEEHDIPLTQTARITADLLSLRT
YRFQFQVLNTTYGAGEVVEQNFTTLQDPRIPASVVLDPLRYQFVRATQGRPTASQTQLQISWTTENIVVG
NATFVLNITINNASTSSIRVVGQEAYTLTVGSGVYVRATIAVAGITATQAVQVPPAPTLLHINTNATCTE
LECGPRGTHFLIASREDDPLNFLYRTLMCDASNTFYLCDLEEFSSYTLWASYADSRSNSSFSLPVGVDTD

AAIPSAPPLNVAVSEITATTAQLSWESPPPLTLNGKLQSYRVVITSPGLPASSQDPTTTSLMLTALRPYR
AYTVTVAVQNIEGIGPASMGVVFTTAQGQASAVRSLEAVNITSTSITLTWAPPELPNGIVTSYNVTRAFA
NSVGETLTISATGLTATIPATPYTEYAIVVVAINSAGASLPNNIEVLSLSAPPTAVLAAPAIVRRGSRNV
TLSFSTTPLTGAINGPVASLVYLIQVARVAEVDGETEEIVTYNATASPVTVEGLRPNQVYQTSLLVMNDA
GSGPTSAFSTFQTAPALPSAAPVIVTINAIENNLLVRWDGLDAQDQNGQVSAYFLIVTPQSLPDLPLNLT
IQADGDAGVTVVQGLEYNEFYLVSIAAINQVGVGPFSEPVIAPETQRSSGNAPRFVRVANITSRAALIEW
QPPTGTTSLGGYALYAQGPSFQLANGTDLKYPLGAVDTPQGFGLVVDADTTAFWVQGLVPDTQYQFYVIA
FSRSDVFYDSSAIVGGFTLEDRPTLPLDLRFPATYLQATSLQFSWNRPDPDNGEITGYRVCYARRTNASA
GCTIQELAQLEYVCADTIFTAYAASGLMAYSNYIFGVEAYTEIGSSSFAVSCVRTAVSAPIEAPSVQVTS
LGSNGFTVGLYAPVTRTGPIVRYRFNYEWTSTIRTLCPDGQAARQCVVDIVVDADDSEVGQLSVANLEPN
AEYRLYARAYTERNGALLAGPASTPILVTTLPSRPGEAVQDLGNRTTINATSLVTSWRPPYFPNGEIVEY
NVSWVQSSECGDAENNTLLQSFFPHPADAAQPVAVEDRSITGTELVAWQDGGLVNFTIPDLLPYTLTSIW
VAAATVVGYGPTSDPLCIRTAEAPAQPVTATRIVSVGTDNLALTWQSPQRINGELLNYELYACPLSDRGA
ATAAAQSNACADAVSTTTSTTSTTSSSSTTTTSTTPTTPTTSTSTTSSTANGGSTSNIQPGTEDVDASQG
STLTAQPASSIATSSTSTSSTSSTSSTTRSTTSSTSPTSVSSGVSTVPTLSTVPSGSTSVGTTTTSTPTA
PPDPTLCRAPLPSCARTPVLVTAPAGTAQAQLTGLHPNTLYAVGVRAINNAGAGALQFALRVVLTATDIP
AVAPVQPNVSDLSTSGSATDSTRSAQLSWAPLSPTDANGEVTYEVVVERVGRTYNTGTIAARNAAAGPDE
PDAEVDSAAIADQAVATQPLMNRRRRQEQGTFTTTTPQLLLDELLPAALYNVTIAARTSAGRGPRSAPIT
FNTTEGIPFDAPTVSAFETADGVRISWDPPSQPNGQIVSYILWTSVDGEEASSQLLTARMYDLSLDKERS
VSYEFRVAANTAAGRGPASDTEAVTIAASSSGQGDSGSDVIVGIVIPVVVVLIILAVLLVYQERRRARRQ
VDKILAEQAVLGEKPYNFKNLHQDLMEENSNEDDEELYGHIHRNKQPYELPSRSLVLDKILGSGAFGEVW
LATLDPTQLPSKSTAGVDPPLKGKTPIKVAVKMLKKTADRREAINLLAEASLQAQFDHPNVLTLLGAVTI
KSPVLVMPFCPNGSLELYLRREDANESWFPFFILDIARGMSYLADFNFVHRDIAARNILLEHNFNCVISD
FGMSRGVANADDYYTASSAKVPIRWTAPEAVVSRKFSEATDVWSFGITMWEIFTHGERPYGPKSNFQVQM
EVLNGYRLSQPTRCDDAFYLIMRSGWDPDPHKRPAFKDIYNMLKAIIIYRELMDTRNVSASSDSNGNPMT
RRLTQVDETTSRPQPALPLNLSTKLDTSSQV*

>jgi|Monbr1|25247|fgenesh2_pg.scaffold_9000181
MTAPLHPCRLALLAILLLALVTIPSTAFGGGGGGGSREECDEGEYLGRFLIYERCYSSVSDATHFRVPAAC
PANSYQDQDDHRSTRCKFCPAGKYNSPIYYTSQANSINDCKTCPFGQTSNGDRSNCYNCPVGKVGAEGGT
CVDCFGNSYADETGLASCKDCSTGGFVVVENSNGLNVGCEMCDIGTYQSGNDHTQCLTCPEGKLATSEGA
TVCYDCDGVIVEQEGQLRCEVCAGRVVDGTCEPCAQNEYLSGTSCVSCPVGHIQSATDASLCVPCAAGTY
LAVITEASETTYECKACPGGTYSASSGATQCTAVSAGYVASADQTSQIACGLGMYSSGGTDTCHSCPAGS
ICGAQACSTCTACTLGTSYTNDEHIQCLTASSCAKGSYITAAATTSSDRECDFCPANTYSDTRNAPSCTS
CPTDTISGEGAQECYTSATACQPGEFYSQEASGCEDCPAGSYTSGAGRTSCDSYDNCLAGQYVTATPTPT
SNRACAVCPAGKYSDSLNAASCTICDASIEYCPFAATAEPTAQPAPCAAGSYQAVGPTSSTARTCVACSV
ERSEYQPNGGADSCLTLSTCAPGQYVTTFATSSTDRACSSCAAGFTATTNTATQCNTWTTCRAGEVQTMP
PTITSDRVCTTCASGTFAANATYCQPWSDCPAGSYVNYAPSTYQDRICRSCTVGTFSTTANAATCTPVTP
TCNVTVSWEVLGASLTNDRICQPVDVCNPTGNNTELCTCPGEGTCSQCSYQGDYNGYLLSSIDQMPNAAT
SSDQPSECVSLTGTGSTSDQCAALCEASSDCVAFFLYDTATDDGQCCLKASFDTGVMQSRIGGSFFQLLA
CDSCISGYISEGLGCTAASLAPNFTVAVETLSLNMKVAEGTVLTHFVATPRSSTNESLSITYAISAVSDS
AFASAFALNSTTGELLLASSVDGSPSVFTLTIQAKDNRTECRLDEVQQTAGGCVTTKTVQVAVVGFAGCP
AITRMTSYLGERQSVTVDVGTIALPPTISNLFSVEVTQPPSYTLAAGDYQVTYSVVEAMDVGAQPTCAFT
ISIVQGFELTASLIAHTFSTTTRKAADYIAEKSPRVDGELILSSFTVTTNLLDNDFAFGIAGPNQPFSLD
LPDELSAALRLSLVWCSEGTMPSSGYNLLPAEVSLNGATSELTFTDGGSGYTDDLACFRVQATSQTFTGR
MSWTTIDVNMPFSSNNPNAVPEPTRRRRALTTYFPADGSSVSMQYNSTDAGIFTEEPGPYLTLSDIIPPY
FLGCPANQYATALAPNYTVAVTWTEPIAFDNVALDEATLQRSHAPGDYFSVLNSPHTVTYDIRDEAALAA
ETCSFRIYVSYEEDPVTGLAADMVAQPRGVIVDMPQYDPSVVEKVALLDATAQRLPFSSNLGTKTGLHFD
INTKGSDPIMIRQTETLQAYFFRFDLTWNSASGAPTASGATATFELVNEDGSEISCPELQGVQPLRGSTI
RIDPNSGAIVFQASSLTYDCAVDFDTITVNLDWPAVDLGTSQTHVYQLTTTSSVDIILLHDGTELEVEDG
LRAAVMFDDIPPSIRSCPTSTSFSAVQNTNYGIRSWTLPVFSDARGVRFLRRFGRFGSSGPWVELVDGVN
ADVSGRSVPFSVGSTTDQVQLTLELATAPAFNILYEVEDWFGLTSNCSSSLRVIDTQAPDLTLQVMNTYN

LSANSATVTVDRDDFILDMQDNANLGVSVVEPQPTFDLGVGVHSVTVRVLDAWARQATGTVVINVEDHVG
PTIRCFAPNLVETKTGTTRVVNYTRAEASDNDAVANLTCSHEPGDSYPIGRTAVVCNATDMSGNLNSCEF
VVVVDEIAVQESRNGTLASASNASSTMTIGVAGAVAFCLVLVIVFLVLRARSHARRPADWNSVFNMIEQL
KNGDDVRIPRELKRANLTMLEELGRGAFGLVYKALFEESPNPGFLVAVKSLHHTAGVSDRQELLEEAAVM
VQLDHPNVVKLVGVVTVGEPLLVVLEYCEKGSLKSYLENNPDVSESQKIRFCKQISLGMEHIHEKNFVHR
DLAARNVLLDALMRCRIADFGLAREENDGADAYYRSRSGNLPALDERKFSESTDVWAAGILFYEIYTKAG
LPYDGWSNQRVWVEVSAGYRLPCPESCSKAVYDLMLACWDETPGFRPSFGKLAEQLSDLITDDVEGGYLR
ISDADSQGTSAQGSELYDNGGQAETRLQQVTSRVVTKDDVGQRVYVEGYNTMGTLMFYGPHQSKAGLRAG
VVLDKPIGNNDGTVGGHKYFDCQSGHGVLVNVSKVSLAEPLQPEGEDFAAHYDMGNGEDEVDTTENFSDA
HALYDMGQSENVIEEDESEEPPSGFSTLDSTQLDTAQATIGTDAPALQINQMFEESALEGLTAFGFDEPD
DVQMHQRESVPTHPSSTADVGRRVLVEGYGEGVLKYFGPHMTKRGLRCGVALDEAIGNNNGFIGDHAYFT
CPDKCGVLVIPTKVTLL*

Amoebidium_parasiticum

>cds.gnl|Prjna189477|G13375_064169|m.165057 gnl|Prjna189477|G13375_064169|g.165057 ORF
gnl|Prjna189477|G13375_064169|g.165057 gnl|Prjna189477|G13375_064169|m.165057 type:complete len:670 (+)
gnl|Prjna189477|G13375_064169:364-2373(+)
MHPFFCTLALVASSHAITTTTAISSTPPATTTTTTTAATITPSSDICEDELCYMATTAAG
AFISAPCPAGYNLTFTWTSDPAAVTWGQDLILNAWWMLAANTSVPGYVPEAQYGRGLQVA
EANLVVCNTSTAEGQQCPNDYTPNTTLWSPAAQPNTALSVSPIQRVIAEPSDPVKAADVS
LFNYTATQTFTVLNYTDNLVVVPRMVFYDSNGTAWMITRRCDPAIAVTPGVTPPAPTLTA
EVQWTVIVVISIGLLIAICIIAILFVKYRRLVGESKELRLQKYPRDKPHASSSPSALDAV
CGHSGPLPRNTFIALEQVGQGEFGSVYKGAVFYRGQAALVAIKTLKPKHAEMQSEFEREA
EIMKRLSHEHIVRLVGVVEATEGEPLCLLMEFMPGGNLLNHLRASPGPPSLREALWYAQS
IAQGMQYLASQCVVHRDLAARNCMLGAPTLHSGGLPVVKISDFGLARAHTDKAYYQMGGD
ELLPLRWLAVEAMTERRFTQASDGWAFAVTVWEILTGGEVPYTGLQTYNLAASIALGLRL
PCPHTCPPALYSLMASCWHTDPSQRPTFTDMVTQLETLSNVFSSASQQKLYEIENPYPSM
KDVLVPMEPSPLTYTEVRSVVSESLLEPPKNPYEVEISVSPMGAIPSISFDDEQYVKPES
CSYAMSGQQ*

Sphaeroforma arctica

>SARC_10364T0 | SARC_10364 | Sphaeroforma arctica JP610 TK protein kinase (918 aa)
KGTFSANSALVYVWAAGNDLDCCSLAVLIALREAGVTVYVDPACLQENSVSDVSNYYLAW
EFEFNQPKSWECLGPCASLLDSDVCPTGLTCVSTSYKDYTCNCPKGYSYSPIQPDDLSIV
ETDEDTDDPTTVVDTGRGPERLEDCVNDDECALGTHDCHPEAQCTDTVGSFTCKCNAGFS
GSGQDCFPENFAFFRPPIYAVLSEHFDGGDVDAELDTHVLGVYMFDILVQRGQCSLLCYT
DASCKEFVIDTSGQYSGRCITKSEASNDTIALFEQTVLKSAYVYTKIQPVAVLNYLGPSV
GYLSGEGAFNLSREVRLKETECASWCNELSSCRSFDVALGGSNAGKCQLNTVTSVATSLD
THTNTGIDSTGQETNITTRVALDNTTAQSTAPAIPQYNFDEGNVFRYTRQLLDLSTDCGD
LFGCLTCTSDNRCVICESPFRLSSAALCECEKGQYLQNFHVPYKCNDCSANKCRQTYLPC
SAYNDLICASCPTGWYGRQCDQISDIDETEKPISADSIDPAITSTLPGEQFPSNGKESSS
AAAGNPILIKGSPNITIILIVAVLVAVLMASLVAVIIVLLIRRKRRKETKRRESKTESVE
LDDSPSSPTPGIRQLPPVDTKLKTTFIDPMAVTVLRTIGNGNFGVVSEGKLSVGEETHHV
AIKELKHKMAVAKDEELLAEAAVMTQLYHDNVLRCYGVSILTESNSPCFLMEYMSGGSLN
EYLVRNHGKISIAVRIFFAHQIARGMAYTIARGIVHRDLATRNVMLGPMCPGESKVAVCA
VKLADFGMSRYFTEGNYYQMANQTGPLPIRWMAPEALTLSKFDERSDVWSFGVSLWEIFS
GGKIPYENIESHNLFTSIDNGLRLKKPESCPDSIHNIMLQCWETNPKDRPCFYTIERRLR
AILCFYEPELVNTVGDY*

Ministeria_vibrans

>cds.comp9810_c0_seq6|m.25039 comp9810_c0_seq6|g.25039 ORF comp9810_c0_seq6|g.25039 comp9810_c0_seq6|m.25039 type:5prime_partial len:422 (+) comp9810_c0_seq6:3-1268(+)
WCAAGPPPLESTFAAALATAAQTALPGATNIIEPGDDYGDFDRVRKDKSRHEDLYKTVRR
KRPTRTVKLEDGEEDGADGIGSNATLERAHNAQFQTLFDELMPKAFPREWLTYVDFLGKG
EFGKVMLAVSSEPGGGKTAGPKRTLVAVKTLHEGAAAQTQNDFLQEALLLKQFDHPNVLR
LIGVCSDVTPWLIVLEHCPHSDLRNFLRALVVARSLVTFADQLHMCYQVASAMEYLSQRG
FVHRDLAARNILVGNRMVLKVADFGLSRMTDVGNEYQMVSKTKMPVRWMAPESILFMRFS
TRSDVWSFGILIWEIMTFGTKRPYERMSAPELIQSLTDGRRLPRPFNCPEDLHIVMLMCW
SHNPANRPTFGELSELLKNSRDESTATTGKGLQDLPKLLEDASYEKEGGEGEGGVSGATV
A*

Capsaspora owczarzaki

>CAOG_01676T0 | CAOG_01676 | Capsaspora owczarzaki ATCC 30864 TKL protein kinase (1352 aa)
MAPHRPLTVLRRPVVAVLVAALALVLFPQHSAYSNAVGIICPVQPARRVAGCGETLELIL
NVNSVTTHSIEIAWTMFAPTGTSEYQVYRDGTFIQSVSIGTFSYNDTGLALGTTYSYVVQ
AFNDGGQPVRRVIAVGGSMSSNSVTQQTAYNPALNSSSLALSPSSSWSISMSWSSIVVDS
GDTFSLTPDMTYTVAYAPSTNPAQLLAFSLKNESCVLTNLQPATAYQVVLTVANGLGGTT
TLSSTAMTMDEVALSAPLRVGSNTPTFSMLEQTRVLVSWGNVFAPQQPNSFPVTYVLTRT
SGATTAQMTLNATTDSAFYATGLSLYTSYTFSVTATQNGLTSVSASAVVMTPAGTPVLAG
ATSSTRYVKGVLFTWPNPGVTPNGPTVSPATMQVSTNFGTSWTNVTTNALNLTTTTYTTL
SCMRRYVNAANFVSLAQTVSAYPYSWEPTFDSLFWSSLASRGSTWLNFTWSAATPNGPGP
VKYRVVQLPSTTVVDYTSPLSSTWYNVTGLTPYTSYTFTVTARNQNSNESAVQTTAALTT
LPDVPVWIGAPTTAPTATAQSLSFAWSGLLNWRGPPGSYNWVISPAPFYGSSTGSTLQPS
LTVPYLEPYTQYTLTLNATQSAVSGRRATAAPFTSIPYIFQATTLAGEPGWDANGILVSA
GSTVISTRYVAVTLTWTNAVEAGTPLFTCNLTATPAYNMSTIPILSDSTVTTATMLFHMN
TTVTFTLSVTNQVGKSATTVVLFFTPDLSGSLSLSKKHAATSASLREQTNTFASSASSKS
SAVATSTHATGRPGVGTSPSTGSGGIPLAAIAGAIAGIVVLAIVIAILVVRRRRRRQPNA
RRGQAKGSVAELLPMHDSALVAIRAPAAGARKEPTSTTAQQQAHDHDHYHVYAQNNDSHH
NEEVYAAPGPSTNSNRPRESVVYEGTGSVYYSQADKAGNKTSRNNPRDSSVYTTTSPEYA
QMDGSSTMYETANPIYSSADLLPGARPAISTAAYKQTSGARHRDSNIYATTSTPALSGPD
GGADEEYREIEGTRGEYYYGNANARIYGTASTTAKTIRGALKLGRTLGSGAFGMVHLGTL
AASGLPRDSEHLIPAGQTSLQVAVKLLQEDADEKSRRDFDSEAAMMSQFSHPNIVLAIAA
LVEETPHMLLLEFLPYGDLRELMIKSKSALIAWKLPEFSHVLAQIASGMVYLESVRFVHR
DLAARNCLVGPDLTVKISDFGLSRVLEEDSDYYRVQTKGRLPVKWMSIESLVFRTFTTQS
DVWAFGIVAWEVFSYGNTPYPQIAPIDMAVHLENGGRLGCPDSCPPDLFQTVAQCWDFDP
ANRPRFQDLADTFAALVGSDVRDIGARLAQK*

>CAOG_09852T0 | CAOG_09852 | Capsaspora owczarzaki ATCC 30864 TKL protein kinase (1877 aa)
MKPRLLLLAVALLAAVGSVRADLEITFTAATGNSITLNWAAYSSFASPMEYRVTRTPGSA
TPVYTGTALTFADSSLTKNTAYTYTVSVYSNSGVGGTLVGSAGAQNSQTTANDVTLNADS
ILMYATSNVNEHALSWPSLVANTGASTAGPPATITYTVEYKKVASGSYIGWTGYSSGTSA
PALVLAPGTNYNVRITVSSGILGTASVNQVFTGRTYSVAPVIGTALAAGTITATQVPLSW
TVTSSGQESNLALAQVLSRNGVDLTTLPAGTGSYTGSLPQPFSFTDSGLLPYTSYTYTVR
ATTVAGNSTSSPVSALSVTTASAPPTVAFLTTAPYITQNSVTVGWSLTTLNGPTPITFVL
KRGSTTLTLPGGTSSTSATGQFFFTDDLDLDPCTAYSYTLTATTSDSKSFTTPAKSFTTL
ADKAVLSPTVTSLSYTYNAFSFSWTNSALSPCAGSVGTSGYQLSLSVNSGAATLVSPTTT
TSYSLSSGVLPSATYAFSLVFTNAQGNASDPVYLTTFTTFADNPTVTALGVTANSSNSLT
FQWTGTANGGGPLFYKVDRTSPSVLSIKNFADSLTSATDNTGLLPFTDYTYSVQARNSQT
PTANLSTVATATFKTAASQATFSSSTVSSTPAANSILFSWTAATWNSLTGDYYWTISGTP
APSPSSGVVTTNSVTVSNLRAYNQYTFTVYARSGSGNTYDSAVLTASNIQTLAAAPTIVA

GQFTSTGQTYNSVSLEWNGVLTLINGPTTNSATQWLVERRLNNPQGSFVSVGTFAAATLS
TTDNDASLAPYTVYDYRLTVRNDQYSTTAILSSIRTAAAPPTIDPSQIVPAFVSNGPTSY
AVTFNLTGAYYRGTPASASVTFLLRYKLTSSGSFTSAPTFTSDTTTVTMQASQVYDLEWT
ITNAGPLSSTPSTGSVSVPALAPYFNAPSITTSDVTTTTADISWPAPTTKNGGDPIVYWV
QYTAGQPNPGSQTIQTFSSTLLATSTTVSGLYPGVIYTWTVLAKNANSGFSSPFSSVSSL
RQDPADPIWQTAPTLSASGQTTSAFVFNWAAPVYAQDNPSNLKYRVVATQITASPAFPDP
TLFSQTVSINSAAVTVAAASNTGYEPYVQYNVTVHVKSSTNNFVPSNTFLTVTTAGSKPS
WGALTQSSLTGDRASRQITLEWPDVPYPQDASAVTYSVSRGGTPIVSGLTTSSYVDTGLT
PFTDYAYIITAVNSKGSTALGAAVTFKTTEATPLFAGSETLTMTPNANGASITYAAPNFP
YGTLAGYTVSVVDTSFTDSHATPTQSSNSAANVFTRTITSLFPFRTYAVTVQATNGAGTS
PPSDSRTGTFTTSPSAPILDESLLDAQVTSNTSITVSWKGPQPWERNSVLSTYRVECYMQ
EEDGAWTFVNAPPRTPLPADNAAYNTATQQSYQEVFLGLLPYRNYTFNVFVIGSSLTSSA
IQVLARTLPGLPPTPLQATVIPISDTQARVSWPAVDDINGPIVSYVVEGQRLLSGYNETW
PVPVVYAELTNGRNVTTVSGLAAESKYRFRVRTFTAAGFSLGPFGTSATTLEAAGGSQGW
VVAVVLIPLILIALVVLLLVRRRSQRTVKRPKDSTGPVYQEAVEMKISPLNHSTGSHKEE
DIEAYAAVGQKQPDPLYQALSTDAAEVVYDYASAYVAGSNSRRVREGLTIVKYLASGNFG
DVALGRVPFNMLPPRAQALLGSTTPEIVQVAVKSVKSDADEKSRQDFESEAKLMAPFVHT
NVVRLLAALVESEPHLVLLEFVQYGDLHTLLKKSKKQSFWWTQNEQIHAIRQIALGMEYL
GTLHFVHRDLAARNCLVGQGMVVKIADFGLSRQLADENDYYRMQTRGKLPVKWMAPETMT
FRKFSTMSDVWSFGVE*

Monodomain cupredoxins identified in this study

Monodomain cupredoxins in Fungi

>Afum_Cu_5740 A. fumigatus
MAAVLNLILAISWLPLIYAQSDSQTETPTSTAVHTVDVGEHSFDPDTLSVSPGSKVEFHF
YPSIHSVTQAAFSNPCHPLNQSSFFSGTDGESFTLTVNDTSPIWYYCGEVGHCQAGMVGV
INPPRNSRDTLDAFRSAAKNTENSTVPAAVGGGTLGKPSTASTTSSASSQTTTSSGSSTS
SATTSSSSTSTSTSTSTSTSSPSPSPTSEGSRFCSSLSIVMLLSVSMAM
>Afum_Cu_1558 A. fumigatus
MLKLSIMSMTVLLFFQSRTTHGAVAMTPSSTATHTVKVGPKQYSPHNITAAVGDVIVFEF
YPRNHSVVKADFMAPCVPAAGEIFYSGQFNNHGQLEWSLVVNDTEPTFFYCTAVDSCLFN
GMVGVINPNETMTWEAQYAQAQQYPYMLVPGQSPPAEGTGYLSSGSNAHKKSSFGGGAIA
GTVVGGVAFVAAITLCRNRERQGSSSQVGRMERTAHWARFTLSLSSGASPPAVSPPLQNG
SSNWESAMKQQPPQEIRQPSELEATNVVGGTPGG
>Ncra_Cu_27829 N. crassa
MPSLSNILLAATLAFSSAKTIPVKVGESTFEPANIKADVGDILEFWFYAHNHSVVTSTAA
KPCEPKTDNGFYSGQTGVAFRVTVNSTTPLWFYCSQGKHCQAGMVGAVNAKSTDDFDKFK
TAAKAAASNVTPAGDVFGGSVAKASSSSGSGSSGGSSSTTLVPVHSGSASASGAWSSATS
STMPTSGAGAVGVSFSVLVAALGFAL
>Ncra_Cu_33967 N. crassa
MTVKTHVVSVGYNVFSPNKISAEPGEAIQFQFVAGNHTVTQSTFDNPCQPIAMHSNVTGI
NSGAKDGKSMNGTTSGYTVMVKNKNPMWLYCAQGKHCQNGMVMVVNENPSSNATKSLQNY
KALAAKATTIVPSGSGSGSGSGSGSGSGSGSGSGSGSGSGSDSGSGSGSGTGSGTGSGST
NGSTTTGPSSGTTPSNSTSPSGSSTAVPVTAGAGILSAAGTTSMFALVAGAVAFLFL
>Ncra_Cu_27669 N. crassa
MKVTTAVLAALSMAPFALGKVAHNGGGSTVTVTATQASTAPASDATHTVTVGGPAFSPAQ
TKAAVGDTVIFTFLSQNHTVTQSAFDKPCVALPGGMDSGFQANLNNTVAMQVMVDTPLWF
YCRQANHCGKGMVFSINPTAQKTHAQFQAQAIAQNGTGTDSGITGGNASAAPPAAPSATV
GGDTTATAVAGGAAQATGNIVTGVGQVAADGSCVCAVSCAAGSFPNVAQGVNAFGGVGGS
IPRAMALGAPPAA
>Mory_Cu_03946 M. oryzae

MILIAWLAAATAPVSALQIPDLQTTPSTPPKIVPVTVGSTSLRFYPEDVRAAIGDTIQFQ
FWPNNHTVTQAIGPQNPCRPMEAVNGAEPPVNSGTDGAPFSMQVKSMDPMYLYCARAPHC
ALGMVMIINPKNDSDVETYRKAAIAKNETIYGNIAVAGGQVGFIPNNQAVPPPS
>Mory_Cu_08291 M. oryzae
MQFKAVLLAVASVACVETHKVNVGESSFSPEEVKAAKGDTIEFHFFSSSHDAVMGLADRP
CEAMGMMDGSFASGPEGDQFRVEVNSTEPALVFCSVGMHCANGMVMAINPNGEKTATKFK
DAARGKASKAPEGVYGGTWGNAA
>Mory_Cu_02330 M. oryzae
MCLTSLIAQKGDTIRYSFFAANHSVVESNFADPGGAGFVPTASTTFTIELNDTRWIYHDA
YKQKASQATPSSPQSKRPGELRFQPNNITEPAGTVVKFNFNPANHTVTSSSFDKPCEPNQ
DGFSSGPSGVSFHIEVKDEKPVWFYCAAGPATGGHCSKGMVGAINAASGNTLEAFVEKAK
TVLAASIPPKAPIGGVVTANGTQIATFNGNVLSHVPKPGDNTTEAISGMAGGSQPLNYGW
GETLSPAALEHLEVLLLIDNAIVSVIGGAAAQALVHRTTLTDALQHFNQGVKPACRVSFP
TGSIDEWLAAVQNLLTLQIGVLTDAITQLASSDPWLAKSRSSAVVNMIQRHKASPAAREP
LLPAKLALSFVRREFVQSCPEEIKGGLAAAKQLPELTFSDRKKLGSTDSVTAVNVVIPAE
AAWGSLQFTVVRRSDGVPANMYGTVKLQEVADVAVA
>Mory_Cu_06653 M. oryzae
MQFATVALAALAMFTGVQVHAVRVGQNIFSPNKITARTGDMVQFMFMGGNHTVTQSPFDD
PCQPISKTMKNVTGVHTGAASAAYTIRVNNTNPIWLYCATGPHCKNGMVMVINEPTNPQR
SLAEYKKAASQITGAAGVPGGPSGGQSGTTPSTGGTGGGSTGGNGSTGGTGSGTGTGSGG
STPGGSAPGASGSPAPVPAAGSMVAPSLGLIALAFSYLL
>Mory_Cu_12247 M. oryzae
MLSLKTLAVTSALALQVNIRIDVGKNVFSPNSTNAAIGDVLEFHFYARNHSVVQGRFDQP
CAPFPNNPFYSGSEGNEFQVTVASTAPAVYYCSQTQHCARGMYAVINPSGQNTLQQYGSR
VTANITAIDPPAGIRVPNGGVYSIQSSQQTSTASSATNTPTGATTTAGAGPTMTNNPTST
KSGFAVPTQAPVAGVVGVALAALMLV
>Mory_Cu_13261 M. oryzae
MRTTNGLVLILAGLAAALPGGNGRLRPVPIQLIPVVVGGPTFVPNMVLAAVGDVVQFQFS
NGNHTVTQSAEGQACTPLQMTDPNAVHSGKDGQQFNMVVTKTDPMFLYCATGPHCQLGQI
MVINPSNTQQLVNYNKISALATKNVDGLKVNGGSVGQIPLGAAFVPAPPEDGGKGKGDAG
KGKGGKATGGKGKGDGAAAPPAAAPAATPAAPPAATPPAAAPAAPPAAAAPAAPPAEGAA
NPAPAPAPAPAPEAAPAPAPAPA
>Mory_Cu_8593 M. oryzae
MKFSSALALGAAPLALAKAAHNGGGATVTVTATPPPAEVTHNVTVGGPIFEPNEIKAKVG
DMVVFKFLSQNHTVTQSAFDTPCNPLAGGMDSGFQANPNNTVAMQVMVETPLWFYCRAAQ
GAHCGKGMVFSINPTAAKTHSEFAAKAIASKGLGGGGAITGNPGGGGAAAPPAAAPPAAN
PAAPAASGSLAPLPPTGGAAASPGNPTTGQGKLQPDGSCQCVVTCAAGSFPAMVQGLNNF
GGMPGSIPANMVGM
>Mver_Cu_01248 M. verticillata
MKAFVAMVAPAKTWDVNVVNGTFSPQEINIAPGDTVRWPNNDADHAIVETNPGARSCVAK
AGGFNSGTKTKGQEYQHIFPNATVVNYKDGVGANCSKGATGTIYVGPRPSGSPASGSTST
TTSANRAATRTAAPISSPTHGGASTAEKSVLLGVACMLAALVF
>Mver_Cu_02882 M. verticillata
MHAIAAIVAPVKTWDVNFVNGTFSPQELDIAPGDTVRWPNNDGANHAIVQTIAGARNCTS
MPNGFNSGVKTKGQAYERVFPNATTINYKDGVDANCAKGATGTIYVHTGARPSNSTTPSG
PSNSATTSGPSNSATTSGTRTAAPISSPTHHSAANGLSVEKSVILGVTCFIGALAL
>Mver_Cu_09232 M. verticillata
MRNLYAISIIAVLLAPVLHMVTIKNFSFQPQEVDIKPGDQVTWTNNDIIDHTVVSNDGTS
FASSNLATGQSFSFTFKTAATLPYHCSIHPFMLGTVKA
>Spun_Cu_98513
MKTMKAPAFIAAAALAVAAPLASAQITGKQQTVKFTWAGGACSPLVGGVDSGKLNTPVVI
KVGANTFEPKEVNVAPGTWVKWEFAAATHNVKQVADPNSCDPMAGGFASANLNAGASFMQ
QFTQPGPNYYVCTFNQHCSKGMKGTVNVQGAGGTQGGNNPTGSTSGAATTHTITVGQGGQ

KFTPETLSIAKGDTVMWMWAGGPHNVKQTADKDSCTPMDGGFQSTNLQTGGMFSRTFGAN
ETTDRIWYVCTVGNHCQNKGLITLDGTPGGGAPATQASAAQPGSRARLAFGGVAAGVAAA
FVL
>Spun_Cu_97705 S. punctatus
MVTFHVEDGPHSVTQTSGDNCDMLINGIDSGKLKRGGRMAVRFITPGIRWFACNMDDHCD
RDMRFAINVIDNGTDSQSGTPVSQSDTPVKPLVETTASSRVNTPIPRVNIQAAFIAGGAV
VGGAIIGGFKLVLILRRRREKKIKLEEQRGRREPPV

Monodomain cupredoxins in unicellular holozoans

>Apar_Cu_154092 A. parasiticum
MLYSATAITVALLGLCAVTSATKTVRILWEEPLSGANELASSLRPLDQVTFEFTGDESSV
MNFFNKKSYDECNLNQARLLSPTGPTYRLLASGWQYFGSGVDDQCLKGRRVAVHVQQYGV
NTDGDSLHQAVQAAQAAALPPKPQTSNSTLAKPAINSTAVEWTRETPQTLYVTAGSAVLF
KYTGHSIVMLRSVDDFSCKMNRILRV
>Apar_Cu_235353 A. parasiticum
MKVTLIAALAGVAASVTTPPSTSGTKIVPWVVPGNYATPEKLTVGETITFKCAGMDHNIW
QVSKAGYDGCTATDATTSTFWGGCDDGETKVIASALKEGTTYFICTEGGNDEAKINGEDE
PRTGGHCLAGQKMEVIVSAAGASPSAGGAHPTAHPTATSHEGHTMHAATTTMGAEPTLPM
EVMYDLLGDFDF
>Apar_Cu_52294 A. parasiticum
IEGGEFVPQLSSTNDLDALLTEPETINARLEAGTAPAPAPAQAVSVQWIRPTNPIAEGMT
VRVGDSVGFRFTGGHNVYLFRDQTAYQGCRFGQATLLEEPYMWKPTQPGTYYFGCGISDH
CSRGMKIRVNVTA
>Mvib_Cu_4624 A. parasiticum
SPCASDATCTLQAVSGFMCECGKTGASSPCENGGTCALIVIPWCLDGSGTPTASHNVEVG
DRVRWDILSGTHTVSTGVTGIMTFDSGTLTAGQTYDQVMTAEGTISYQCNFHPASMQGVL
NVAPRTTFFSECDVGYTGATCGTN
>Sros_Cu_10601 S. rosetta
MSVFVLLVLVVLVAAVLAGTTSEVETVLWQFQRELQDALGPLTVEQGTIVRFRWEPYGFA
HGLFQVTNQNDFDNCNLVGTELVARSTRGDYNATLDPGTYFFVGGVGSNCETGMKVRVTV
LEQGETPPTVMTTTPIPAIESDLAKVVVVDWNVPKPDVEANQDDIIQFRWSGSISSSVRR
VTQENYEACNFNNPLEIVSESTTSNVVNLRVWTSPATIVNEATTGSGDSVALDISAEEAH
TFSAVPGSGFAPSRVRVRARAEDLDDGDARESKTFTCSRPEGGVSAINCIVPADGSTSTP
PPTSTNTTQQERDCECFRQLLAHHPSQRVLACSKDGCFATVDDGTAGWSTLPAIRSLIGT
GTDETSTIATPATVFSDPLFEGSGDTPLDRHVQTSSSAAADWGITRKGVLVNTAGTWAFR
A
>Spyx_Cu_574755 S. pyxidium
MPAQSMGHNVVIADTANIQAVGTDGMTAGADNDERVYAHTDVIGGGESTSITFSTERMTA
GGDYSFFCSFPGHWAVMQGKFEFK
>Spyx_Cu_588060 S. pyxidium
GREVVIPPELVEIGIAKGEIAFEPNSVNIPANSRVKLLFINKSEAEGMFHNFVLVSLGAG
QEIATKGLKAGKAKERVIVNTPLLAMGETITIEFDAPPKGSYHYICTFPGHTNMVGRLNV
>Spyx_Cu_645215 S. pyxidium
DTFKVKAGDKIILEIDNLDGMEHNLLIAKPGTLEKVGAAADAMLRDPKASEKIPEVLFST
KMIGPQELYTLTFTATKPGNYPFACTFPGHWRMMNGIMVVES
>Spyx_Cu_662530 S. pyxidium
MSIRSTLLVLFFASFIFPADNCIVNIEATHFSKSEINVSASCTSVTINLKNSGKLPATLM
GH
>Spyx_Cu_676302 S. pyxidium
QRVVIKPIVNEKFDLKEFEVTAGDYVEVRFSNIDFMQHNLLILSPGSLEKVGQAADKMAT
HPDGAALMAEVLYATKLVDPDAEAVLRFEVDKPGDYPFVCTFPGHWHIMNGIMKVKPKK
>Spyx_Cu_689772 S. pyxidium

VVFNKLPHNIDLHAVTGPGGGAESSFVAPGHEKVFSFKTLNPGLYVYHCATAPVGMHIAN
GMYGLILVEPDGGLS
>Spyx_Cu_92599 S. pyxidium
GEEREVTLNGFDPVLKIAHDDDYEARLVLNTVSQKAFDKKLLYAKAGQTIELVLNNKDEM
PHNVVVIQPGTLDAFGEMVDSFLKKPGAAEASRYVLGVVDMLDPAESASVIIQLDEPGRY
PFVCTFPGHWRMMQGEIIVTAPGNYSDDPEAPQLTITGGGGSHDFLKQYGVNDGRLLSMN
GEVTVRYTENTSNLGAMLSDTDVLLISNNKPFDVASQQAIFKRVNEGMRLMINHPGAWDW
PKYNVGGGSTSQN
>Spyx_Cu_120943 S. pyxidium
AVVSTQSSRVVFLTPQAADIAIMDQVNKQFLPHILVVQKGTRVAFPNSDSIKHHVYSFSE
AKRFELKLYRDKQPEPMLFDTTGVVEMGCNIHDWML
>Spyx_Cu_120946 S. pyxidium
MRKTWLTGLMSMMTLNVIAANVTLVPGAVVFLTPQAADIAIMDQVNKQFLPHILVVQKGT
RVAFPNSDSIKHHVYSFSEAKRFELKLYRDKQPEPMLFDTTGVVEMGCNIHDWML
>Spyx_Cu_15160 S. pyxidium
SVIEIDNPAAEDIATMDQVDKQFSPQVLVIQQGQAVEFPNSDNIRHHVYSFSSVKPFEIK
LYSGKQEAPIVFEKT
>Spyx_Cu_16573 S. pyxidium
QSVLFLFLVSLADIANPEITITIKDSLFHPSVVLVPANKKVRLVIINEDEDPEEFDSFSL
NREKE
>Spyx_Cu_215319 S. pyxidium
MTITKFKKKHVSCVLGLWLFMLLGALNSSPVPVFKLEIKNHLFYPAEIIIPANTKVRLVI
INRDDTPEEFESYELNREKVILGNSQTIIFIGPLPPGEYPFFGEFNMSTAQGKVIAR
>Spyx_Cu_614696 S. pyxidium
MTRITLLLLLLFSQCLLAQQIAVVWLKGSDSHYTMSQKKRAFVPHVLAVPQGAHVEFPNL
DSIMHHVYSFSKTKQFELKLYHDKPEKPINFAQTGVVELGCNIHDWMLGYI
>Spyx_Cu_652424 S. pyxidium
MTQKDTLFRPFVLPVSVGTTVTFPNLDEFRHQVYSFSKPKRFELRLYGQDTTNTITFDKP
GVVALGCNIHDNMLAFIYVSEAPLVGKTDTEGMVTFENA
>Spyx_Cu_652843 S. pyxidium
DKIFISRDQAEVFKANLTGSPMVLNLKGGKTVKLMFKSTNARTAQSESGDLYIDEVFWIP
KYKTLRGLAQAMATHKHLRITYSTP
>Spyx_Cu_654475 S. pyxidium
SFLTVPAKQKIRLIIINHDNTPEEFDSFDLNREKVIFANSRSRIFIGPLDPGDYYFFGEF
NPSTAQGRIIVTDDQSKGAKNV
>Spyx_Cu_152634 S. pyxidium
GRLPAPALDNAAGRTCEDACSVSVPTPVSWAARTSTAMTVIAGDTLVFNATGTQNLVEVT
AAGFAACDVPVLVGVTIKEHDFSASVTEATEGSHYFVCTVGNGARCSQDMLRLHVTAVAR
PQTEHLECIDKPPPYGGDRDGRICQC
>Spyx_Cu_457588 S. pyxidium
VEIDACQAEQRNGVDVCADIDACVDFPCSSKDAQCIEGRVCDAGFVLGPGLPAPALDNAA
GRTCADACSINIASPIPWNAGMTPFEKTIYIGDSIEFVSSDGVTHTVIQVKSTSYDVCND
GNLFQYSPDILATVGFRVKVTPSVVTDYYYVNSVLEGQRCSSAGMRALIHVVERPQTENL
VCYDLPPPAGAGPDGRSCECARACECIAGTRRDLDSENGDCVNIDACVAFEQVTCRDLPP
PATGDSADGRVCTCRDGLTPNANNICTEADAVCPHSPKALFCVSSRSPCPSSTVLGC
>Spyx_Cu_70505 S. pyxidium
LRGSEQLAKRAEMKEPAEAASLTSCPPVRIEDLPWGEHDGRRMVPAASWNQRAFGKNVFA
LHRPVGRPGPTVLSLEPGDSLNFTIQGTHNIVQDTRATFDACESSSSVQFESSDGWKS
>Mbre_Cu_33299 M. brevicollis
MKTVLGGLAVLFMVSPTTSELTLQWVFNPVFPNLRSFLGDPTVEFGTQLRFSWTNSPHGL
LELSSQAAFDACDLTGVDTVLLQSPTTSSTNLAVSMDRAGRRWFVDNYASNCGAGMKVQV
DTLGIGTTPPTTTTTAPPDTSFVQPSPSDATIVTWHWTSQPQPDVHIPENGFVRFQWDQA
GDVVHEFTNGPQDTGDILLNGTEAQTDGFFEVQQMNVGLVSAVNLILPPGGVYKAHAVRV

QPANQATDAQTFYCATTPLFQTCVAHREVEPELPPTNLTRPAYDDGCFTTADDGLAGWAE
MTAPTRLLGFDQLYSLVTVNLIKNATSDVEDLHTIPRSENTFHVVGTGIEWGVTPQGMHV
NHDGTWILKPEKV
>Aspe_Cu_127618 A. spectabilis
SQMAMSKDDPADLKAQYTWSINGVKYEDAPIRLKYGERVRFKFVNETMMSHPMHLHGMWS
ILDTGNGKWDPVKHTININPGMTVYSETEVDEPGQWAFHCHLSYHMASGMFRKVIVEGGP
AVAALENGV
>Aspe_Cu_128312 A. spectabilis
ISSHWERVFLGLMFGACFGFYQALIKPSSIHNQGSCDVTVTTQEFVPNSISITVGETVCF
TPGSSHNVLQTESAGSCTQAVPLVFGSTVLGDPVQHTFDQVGTFFYMCSPHCSAGMTGTI
IVAALTAASTYGDSYSPTSAAQTAVPSTYGNSYSPTSGTTVPTSYGSPPAGSQSTAAPVV
>Aspe_Cu_136914 A. spectabilis
RTAPAALRSVDLITEGANFKGEQFWSFNGTGRSIEPLITASPGETIRIRMVNDTQFPHAM
HLHGMHFSEVLPDGTLGPLRDTLLMLRGETREIAFQAHNPGDWLFHCHMLSHHAAGMGTW
VRVTA
>Aspe_Cu_147913 A. spectabilis
MYMPNFKNLVGLQSPAFAGAGHSGGHNDGASRTIRITMEDNFYDKEKIIVRGGETVRFVI
ENKGEFVHEFNIGTAAMHAGHQEEMMMMMEHGALEADKINMDMMEMDMGNGTMKHDDPNS
VLLEPGKSAEVIWTFPKDAELEYACNVPGHYESGMVGKVRL
>Aspe_Cu_16005 A. spectabilis
GVLSANTIAVRVEDTGGEGTVIQIKTVKNEKYDISEFVVQAETPVEIVLENVDFMQHNLV
IVAPGQKEKVGAAADRMAADPTGAEKMGEVLYATALVNPEEKVVLRFTADKPGMYPFICT
FPGHW
>Aspe_Cu_164102 A. spectabilis
MLRLHIAVIPLLLFLLSISCRKGETVEQVRDSEYTLEASMYFAKDGTRNPTLRANKGDRV
RITIINGETMTHDIAMEKIGIKSGTLLEKGSTTNITFWAESDDTYYCTVPGHRAAGMVGK
FQIVEGDLSSDWKAEGEAFKNPLFNRPSPVHEENTPVAFDGDFFLSSGGTTNYKLKGTLT
SVPFEVTHPFASFRVSGGALADTRLNDYLKKNIFIRIIDNESGTSPIPYIRDDIWAHISK
EAAKAMTLPDGFKVTLAASEPDVVRTIDARGRLWVVEGHTYPVPAAEGKGRDRILIFEDT
NGDGTLDKRKVFMEGLNMASGIEVGAAPYLLFIPIDKTNDGPPKILLDGWGLDDTHEVLN
NLRWGPDGWLYGVHGVFTHSNVGKPGAKDDQRGAVWRYHPTRHEFEHMYHVIQGAR
>Aspe_Cu_182790 A. spectabilis
SYHTFTAAGTFFFKCNPHCGAGMTGNITGATNTVTPTVNATGTPTLAPACNVTITTSTFV
PNTVTIVTGDTVCFTPTSTHNVVQTDGQGSCTELTPSIFGSATLR
>Aspe_Cu_220103 A. spectabilis
MKKIIIIATLIFGAVFSINAQDKQVISLEQTPGEFTQKEIKVSPGTYVFAVHNNEVGHDV
GFVLVEKGKDVSKPENHIQTAYVTKAVANGETQHSKPTVLKTGTYVYFCPLNPTAIDNE
>Aspe_Cu_229749 A. spectabilis
MQISKKIAGPKFMENGDMLVTLNLSWIGHGSSLVKWGRMTESGLTLLRAPAIWFPHTILG
IRSVKLTAHGMPSMSTSESWENGAEKQLVIGTSPGLKYDVTEVRVKRGTKLELTFNNNDD
MLHNLVITEKGKESVDEVGRLALLLGTELVLNHTGILQPESSETIYVQIDTPGEYWIVCT
FPGHSSIMRAKLIV
>Aspe_Cu_276485 A. spectabilis
MKHLLLTIALASSFAAGEAARTVEIDMVETGEMLFKGGDLDFKEGETVRFVVRNEGELDH
EFILDSQKKNAAHKNEMADMSGMNMGHNEPNRIRLAPGEDAEIIWTFANNGTFEAACLIP
GHYESGMFREVSVTH
>Aspe_Cu_298288 A. spectabilis
PASLGGDYFWSFNGKGINGDPLARLDRGQNVRLKIVNDTAFPHAMHLHGIHFHEVAENGT
LGPLRDTTLLERGQTREIAFVADNPGQWLLHCHMLSHAASGMMTKIVV
>Aspe_Cu_368214 A. spectabilis
MNRLTIASVILTGILIYACGGSTELTDQASEQMEQDSIALTIEGNQFSTDELKVTEGQVV
TLTLKHTGKMAKESMGHNWVLLKSGTDVAVFGTSAVTAPDNEDQVIANTIVVGGGEEATV
TFTAPKAGYYKYICSFPGHWGVMQGTLAVSP

>Aspe_Cu_454707 A. spectabilis
GAIQNPATSKLHVVSQKDKKFLPENITIRVGDTINFRNDDKVFHNVYSFSTARLFDLGAS
GQEKKVVFDKPGEVLVECAIHPKMRMKIIVEK
>Aspe_Cu_459874 A. spectabilis
MKHMVFAAAMMASETHEVQMFNRGAMLYQPEYLQIAPGDSVRFIPTQPSHNAATIDGSKI
NEDFTVTLTESGRYGIKCSPHFAMGMVMVIDVGNVDVAQELPEDLPKRAMQRFHD
>Aspe_Cu_504422 A. spectabilis
MSIRNILLGLFFIGFISAADNCTVDIEATTFSKDEITVPATCSTITVNLKNTGKLPAALM
KT
>Aspe_Cu_517596 A. spectabilis
SFSALMGHNLVISKKEDLIGLTNDSFEASFENDSRAVVVSKVIGGGESTSVTFKTDLFNK
EDEYVFFCASPGHWSVMQGKFKLAQ
>Aspe_Cu_55573 A. spectabilis
GATFNNVPAVSVKTGKVIVLKTIPNKEYDLKTLEVEAGQTVAIIFENVDFMQHNLLIVKP
GTLETVGAAADKMAADANGAEKVPELLYSTRLVNPGEKVVLTFTASEPGDYPYLCTFPGH
WRIMNGILKVRNGKSA
>Aspe_Cu_56717 A. spectabilis
MIRQLATGLALAALMGSAETIEVQMLNKDGERMVFEPAFVKAEPGDTIKFIATDRGHNAE
TVDGGKVDEEIEVTLDTEGYYAVICKPHFAMGMVMTIAVGDTEVPADFLEGRIPRKAKDR
FEA
>Aspe_Cu_569991 A. spectabilis
SFSGTEYSVLLTADEPGEWAFHCHLLYHMSAGMMSTVVVAEYDEEDLPATQPAQHDMGGH
HDHH
>Aspe_Cu_73704 A. spectabilis
VVFGREVLANRTDQLSPLVPVVVAATAEGTFAREMPMTSQKSRGLTSPLCDVTVTTNSFI
PAHIVVTVGAVVCFVPNVNHNVVQTIGNASCTALASPDFGASSETTLGEPIVHRFDTTGS
FFFKCNPHCTFDMVGSVLVFSASPTLAPTDSNAPTSNSPTSSPTTPSPTTSSPTTQPSTN
PTNLPTSTSPTSSSPSDQPSSTSPTSVSPTSSSPTNQPSSASPTSSSPTNQPTSTSPTSS
SPTNTTPTDAPTTSPTSLPSVAPSRNPTAFPTAPTHSPTSFPTLGNNFQRTGGCSFYLWM
KMMQALSHPSDVGYWGLNLYGQYPDGSLRGCTGQYHSESGNLDRFALSNQAFSSGIDLTN
TNQVGVNFLIFRNQFNPAAFPSELFYMSNSLLSITRD
>Aspe_Cu_8948 A. spectabilis
SRSRTFFFMCAPHCGAGMTGQVSPATPPPCDVTVTTPTFSPSSISITVGQRVCFTPGASH
NVVQTGSSGSCSALANPDFGSTTLGQRVEFTFSQAGTF
>Aspe_Cu_91753 A. spectabilis
MLSFALISSGAALAAGSHDGGHKRTIEITMYDNYYEPQSLDIKEGETVRFVIRNAGEFVH
EFNIATAAMHEAHRPEMMMMVEHGVLEPDRINYDAAKAMQASMGHGHKEGNSVLLEPGKS
GEVIWTFPDTGELEFACNVPGHYETGMHGPVKLGN
>Aspe_Cu_94040 A. spectabilis
ACDVIIPTSAFVPNNISVTIGQTVCFEPEGFHNVVQTDAQGSCTAHTSPTFGSITLGQRV
VHTFTQLGTFYFMCGVQFHCPPMTGEVTVSTASSQPQTSSPTSPAPTTLLTSTPTVSSTS
SSPTSEPTEQPITAPTST
>Aspe_Cu_276483 A. spectabilis
MKSFKTCTLAAALILSALSAHASDSQQPHLRARTVDITVRETGYMLFDPDALHIENGAVV
RFRVTNSGKLDHTFFLGSFDEVTENRMTHDAANFISIPSGETGELIWKFSDVTNLEFACL
VPGHRDAGCGGSSSCMITSPHPPDIERAPMSLMDRYHVLVHDVSALDYNDTTPDWVHPFI
HLILVLAPGLLIGVALYLVLLWSRPAKPVARMNHVRGLDAGLFSSVLRYTRRQQAQMIML
SLLALPTLELPKQIVNNALESATFPVEVLGQSLDQVIFLLLLCGLYLLLNGVVTLTVL
>Aspe_Cu_28470 A. spectabilis
PVEAEIVQKNRNFHPHTLIIPKNSRVDFPNQDNTQHHVYSFSPAKAFNIELYAGRPTEPV
VFDKTGVVEIGCNIHDHMQAFILVTDCDS
>Aspe_Cu_335984 A. spectabilis
MSVNKWILALSMLLLCMPSWLKEYKLILKDHLFYPAEIKVPANKKLRLLIENQDDTPEEF

DSFDLNREKVLYPGRTSVIYIGPLSPGRYDFFGEFSPNTARGTVVATEEKDAD
>Aspe_Cu_37764 A. spectabilis
EQLKSEYVWSINGKTLSEVMIKIKRGEAVRVTLNNYTMMHHPMHLHGHFFRVINKNGEYS
PLKHTVDVAPMSSTTIEFDANEDGDWFFHCHVLYHMKGGMARVFSYQTPRDERLKNYKLS
NIMDMDNHWFTWGRVQAASQMSSFELVSSNTRNQVNFDAEYGYNKNLEANFSYERYLSDY
FRGFVGLNSENEQNDSTHKIETAGTIGARWMLPYFIDTELRIDTDLKLQFSLGTEYLIFP
RTMLFTGLDYVLSKSFVLTASRFGLGA
>Aspe_Cu_437501 A. spectabilis
MILLFGALLLTGLFLLMRPVTPSLDRIVDFAVKDGAEGPAVVAVIEGTPVTLRFVTNQKD
EAHLHGYDLAAELKPGQRSEITFVASISGRFEIELHKSHAKLAVLEVQP
>Aspe_Cu_445213 A. spectabilis
MPDVVSVSAEARSWRFAYNGHVTEDVLHIPAGRPVDVAITSADVVHSFWVPRLAGKLDAI
PGHVNTLRIEADVPGTYGGLTAEYNGPGYRAHVFTVRALNAPDWDAFKT
>Aspe_Cu_4486 A. spectabilis
LSVGPLKPGPTFAEPHDSIIVHVVNPLSVWNRQWADTVIRDGNRVMSSSAPTFSLESFKV
KEGDEVTVIVTNIDDIDDLTHGFTMGDHGVAFEVGPQATASATFVASKPGVYWYYCQWFC
HALHMEMRGRMFVEPRS
>Aspe_Cu_470184 A. spectabilis
MKHTNILRPLAVMLGLALSIDTYDITIRIDTGIGYNGSQTPTILKFQEGEDVVIRVKNNL
RESTSIHWHGLILPFQQDGVPGISFNGIKPGETFTYRFPIEQAGTYWFHSHSGFQEPDGA
YGAIVIAPKGGEAVPTQRDYVVQLTDKHPHSGSRIFRNLKGSADYYNRAQRTAQDL
>Aspe_Cu_472242 A. spectabilis
LFHFINEVSLVLLIGITFALIYFAIKYRRRSEDEISVVPLLVCFVIFGWGNAYEIQVSGF
GWNVKYDNGAQTLNEIHVPEGRPVKLVLQSSDVLHSFFVPDYRVKHDVVPGRYTYVWFEA
EEAGESIVFCTEYCGTGHSGMLAKVIVHTEEDFETWLEKNGGGVSGTPVEQGEQLVQLQG
CTTCHSDDGSRLIGPSLRDFGKVKKLF
>Aspe_Cu_487908 A. spectabilis
VVFSSSTAPQILLVNAGETVTFTNPVDNQESHCATQFFEGLFDGPLQPGESAAFTFDTPG
EYFFNDCVNPQNTGKIIV
>Aspe_Cu_489355 A. spectabilis
VMAREWIRYPALGAQPGDLYAVNEIHVPTGRPVKFNLRTDDVQHAFFAPQLRVKQDAVAG
LIIPVWFEIPKAGEYDLVCAELCGWGHYKMRATIVAEPEEKVQAYLKQLQEEQNFDGVVE
DEED
>Aspe_Cu_513838 A. spectabilis
YQEEIIRDGNRVMSQAPNFSLESFSVKQGDEVTVYVTNLDDIDDLTHGFCMANFGVAMEV
GPQATASVTFIAERPGVHWFYCQWF
>Aspe_Cu_535986 A. spectabilis
VFPMMVVHEGDYVELTLYNSPNNMMQHNIDFHSATGALGGGSLTLINPGEKTTLRWKATR
PGTFVYHCAPGGPMIPWHVVSGMAG
>Aspe_Cu_63219 A. spectabilis
AVVWLKGSDSHYTMSQKKRAFVPHVLAVPQGANVEFPNLDSIMHHVYSFSKTKQFELKLY
HDKPEKPVNFAQTGVVELGCNIHDWMLGYIVVVDGGIYGQTDKQGMVELSLPEGKFTLAV
WHDTIEMTNNAKPLSYQLKQALLPKLELSSDEFDDY
>Aspe_Cu_70975 A. spectabilis
YIWSINGIKFADAPIRLKYGERVRFKFVNETMMTHPMHLHGMWSILDTGKGKWNPIKHVV
SVAPGTTVFMETEVDAPGQWAFHCHLSYHADSGMFRKVVVEGGPKDKSAATTTNMKTG
>Aspe_Cu_772 A. spectabilis
NLVQVSAQAREWRFTYAALETTGVLHIPAGRPVDIAITTADVIHSFWVPRLAGKLDAIPG
HTNTLRLEADAPGTYQGVSAEFSGAGYDAFAFQVVVYDESDWAAFLQGQP
>Aspe_Cu_82504 A. spectabilis
MIVVANDGIIGVSETYDVVVTIPTENTAYEFLATNGPIQEMSLQVMYPEITGKIETKVNS
THSMNSTQDAMLKAPYVWSMDNKVLSETKILIKKGENVRIVLHNNSMMRHPMHLHGHDFR
VLNGQGEFAPLKNVLDIMPMETDVIEFNANVEGDWFFHCHILYHMMAGMNRVFSYESQSP

NPYLPDKEWAYKKLQRESNKLHLMAENDFASNGNDGMAMYQNSRWSFGTEWRLGYNDQDG
YETESHIGRYIGRNQWFMPYVGFDYRKTNGISEKNLFEQASTKDNRAVISLGAEYTLPML
VTLQGEVFTDGKVRFQLMREDIPISSRLRFKYIVTRNLGISTHDMGFGI

Salpingoeca_urceolata.

>m.272312 g.272312 ORF g.272312 m.272312 type:internal len:123 (-) comp19331_c0_seq1:2-370(-)
ASPTDNVLVDWSFGRQGPTELAVPVNTTLVFEWASAFHSVVSVSPTEFAQCSGTFVSSTF
SGQAIQGPAVGPMSVKYKLEQPGSLFVACTLAGGLHCTLGNMRINVTVFEPTTTLQTTTT
TTP
>m.521872 g.521872 ORF g.521872 m.521872 type:5prime_partial len:107 (+) comp151470_c0_seq1:1-321(+)
VDPVVVESGAAADDTVGWSTATTVAEASKTVFVGETVMWTWNDGLQHNVVSGLRGSPSAG
VLFTSGGPSLTGAFSVTFTQPGAFDYFCQVHIWMRGVITVVEGEFG*
>m.170178 g.170178 ORF g.170178 m.170178 type:3prime_partial len:147 (-) comp17823_c0_seq3:3-443(-)
MPCGANTMRLAALTLALVLCCPHAAVAHNHNVIELDVGSGPTWEVNGTTNAPLTVTAGEE
YTIVVNTAVSSHPLAFRSDTNANGGGQGITNGVSSPVGTNAYNVTIMFNQTGTIYYYCSA
HPATMEGMLTVEAAPTTPEPTTTTPEP
>m.526004 g.526004 ORF g.526004 m.526004 type:internal len:53 (-) comp174656_c0_seq1:1-159(-)
SAFSFYPFSFEHTFDKVGKYEYVCVPHAVPMKATITVVDGEDDDDAKKDKKDK
>m.496964 g.496964 ORF g.496964 m.496964 type:3prime_partial len:139 (-) comp49698_c0_seq1:1-417(-)
MMALRLALVALTLLAMVSFSQVGANVFQLDVGSGPTWVVDTVTNAPLTLTANETYTFVVN
TGVSAHPLAFRTDTFANGGGQQVTDGVSSPVGTNPYNVTVTLGSAGVFYYYCEIHPSTME
GMLTIDVDECAATDPCDDN

Helgoeca_nana

>m.174133 g.174133 ORF g.174133 m.174133 type:internal len:108 (-) comp24351_c0_seq1:2-325(-)
IHNVVQTGSAGSCTALAGPDFGSTTLGQRVEHTFTQAGTFFFKCAPHCGSGMTGTIQVTA
ATTAAPSPPPCDVTVTTSGLTFSPNSVTITVGQRVCFTPGGIHNVVQT
>m.174136 g.174136 ORF g.174136 m.174136 type:5prime_partial len:118 (-) comp24351_c0_seq2:216-569(-)
IHNVVQTGSAGSCTALAGPDFGSTTLGQRVEHTFTQAGTFFFKCAPHCGSGMTGTIQVTA
TTTVPATVGDSSESSGSSVDSAEVAGIVVAACVVVIIVGILVHVKRRKNQDRITMMA*
>m.174140 g.174140 ORF g.174140 m.174140 type:internal len:176 (-) comp24351_c0_seq3:2-529(-)
IHNVVQTGSAGSCTALAGPDFGSTTLGQRVEHTFTQAGTFFFKCAPHCGSGMTGTIQVTA
PTLAPVSSGPTIAPTAAPSVSPTSTPSASPTANPTTVPTSEPNALPTRNPTAAPSSSPTT
SPTFAPSALPTPAPSPPPCDVTVTTSGLTFSPNSVTITVGQRVCFTPGGIHNVVQT
>m.220783 g.220783 ORF g.220783 m.220783 type:internal len:205 (-) comp25791_c0_seq1:3-617(-)
TTWAPTWAPTDAPTDAPTTWAPTWAPTDAPTDTPTTEAPTAAPTWAPTYQNHAISWGYNV
SGASITITAGESVTWFWDQLPAIDHNVVSGVWHSPPAASEGAEFESLLQSSGNFTHTFTQ
AGTFPYFCRPHYVFMIGTITVISAPTSAPTDWPTASPTAVPSTSTPTWSPSMPPTTLMPT
WSPSADPTYAPTDAPTTWAPTWTPT
>m.225799 g.225799 ORF g.225799 m.225799 type:internal len:210 (+) comp25914_c0_seq4:1-633(+)
RSQSPTAAPSASPTRSESPTATPTAEPSSFGPSSCDVTVTTDGFAFTPNAVTIFVGQRVC
FIPGDTHDVVQTTAIEGCTAMPTPDFGSTTLGQRVEHTFTTAGTFPYMCTPHCGGRMTGT
VLVVSSAPTPTPTEEPTRLPTQAPSETQTTAPSDTPTDSPSTIPTIEPTMIPTSASQTGA
PVAVPTDSPITNLPTTAPSTNPTRSQSPTA
>m.309157 g.309157 ORF g.309157 m.309157 type:internal len:192 (-) comp27422_c0_seq6:1-576(-)
QYGFGWCQDVVPTPSGSTSSRPTDSTTQIDPTQTPPTTTITPCDAYTDQIQCPVTQCQWN
SGPGSTTTAGVSTTVSIIDTTPTMNCDVTITTSGITFSPSSVTVAPGETVCFVPGPSHNV
MQTDSPGSCTATDSPLFGSPTLGDAVQFTATVTGTFYYMCVPHCSLGMVGSFIVAHPTTA
PTTAAPTTAAPT
>m.31421 g.31421 ORF g.31421 m.31421 type:internal len:215 (-) comp12314_c0_seq1:2-646(-)
PLKRTPTHAGRKAARCHPQAAPSKMSPSNRSPPLPPPRCAASTATTTKSSGPTTTTAHQH

GAMLKSSLHMLLVMLSLVGITNATVATHRISWGVTSTAGRDITVEVGDSVTWEWDQTPVA
NLSVVSGVPADASTHGTHFASAEQSSGNFTVQFTQPGAYPYFSPGDYSWYMQGTITVISA
PTAAPTAAPTWAPTTPAPSAYPTTYAPSWSPSASP
>m.460389 g.460389 ORF g.460389 m.460389 type:internal len:120 (+) comp227825_c0_seq1:1-363(+)
GQNVCVSPGLTHNVVQTGSLGGCTSLANPSFGSTTLGQVVHHVFSQPGTFFFKCSPHCGA
GMTGSIEVVAVAVTAAPIAMVSPTQPLTTLPPINAPLTPSTAPPSAGAPSNMSPTLPGSP
>m.61553 g.61553 ORF g.61553 m.61553 type:3prime_partial len:175 (+) comp17576_c0_seq1:2011-2538(+)
MMAVPGGRRLRRHEGPGGGASRSFGVLTVVALFALTPSAVPSNAPTAAPTACDFTVSTNG
LTFTPDILEVTVGDRVCFTPGAGHTVTQTDEVGSCAEESSPAFGSTTLGEAVTHVFSQVG
TFPYKCIPHCGAGMRGVVEVTAAATTTPATTPSAAPSTSPTAAPTTSPSATPTTT
>m.7810 g.7810 ORF g.7810 m.7810 type:internal len:148 (+) comp5413_c0_seq1:1-447(+)
APITTAAPTSAAPTTAAQSCDHHVVTIGNSFSPAWLEVRAGDRVCFLPFSAHNVVQTDAV
GSCTADPSPTFGSTTLGQYVSHVFSQVGTFSYMSAPHCGGSMIGVVHVGIAAPPRRPTSP
APNTTAAPTSAAPTTAAPTSAAPRATGC

Salpingoeca_kvevrii

>m.82305 g.82305 ORF g.82305 m.82305 type:internal len:206 (+) comp8259_c3_seq1:2-622(+)
AAPDVEHDGRRMVPAASKRLPKLMIQYPTAVDGSMRLLWNQRAFVGVLTCCAHVCRETMM
IRGVLLALGLIFGVTGGTIVEVDWTFGRPGPTVLSLEPGDSLNFTIQGTHNIVQDTRATF
DACESSSSVQFESSDVLAPTSQYSGEAVQVEFPSAGVFYLICTVGGGVHCHDGNMRLTIE
VGQQSTTTSTAVPTSTTTTTSTSTST

Didymoeca_costata

>m.351431 g.351431 ORF g.351431 m.351431 type:internal len:162 (-) comp53075_c0_seq1:1-486(-)
VWSVRGLRLEEESAYWYPPCTRGAVTRWVRLTESGGCSSIGDTGTTHYISWGFNSPHKYI
TIQTGDTVVWKWDLDNDFHNVVSGVRPNQDGLFQSSYMSSPASFSYTFNTPGNYSYYCTP
HPGMDGIITVQSAAVDTSLNILSGLTAEAIATAIQDNGRASS
>m.349778 g.349778 ORF g.349778 m.349778 type:internal len:296 (+) comp43919_c0_seq1:3-893(+)
REGGSSAPVSISWGLDTSQEAATTTIAKGDKVTWILDVDEDPHSVVGGKNGIPAGTFDSG
EIAADGTYERTFNNVGIFPYFCDPHAFMQGTITVVKEVLILPSVYTTTASASLTYSASKT
PSVTGVNPTSGKEGDLLTITGSNFVLSDTEVIIGGQFCQITDLQPTELKCTLGPTPGGNH
VVFVTVANMGTATTTNVMFTSVLSVSGISVSSGSFGGGTTLEVSGSGFGGGKDKRRERRD
GAWGNWVIFDKPVEEGENEQLGSLVSICGVPCTVSESTYSSLTCQSGPLHTVDSVL
>m.352174 g.352174 ORF g.352174 m.352174 type:internal len:81 (+) comp57448_c0_seq1:2-247(+)
RNNNANVLFESPLQSSGTFTYTFNTVGSFPYHCSPHPGMEGVITVQQNSETVVAHIITGD
TATAISSAITANANNNGNIAI
>m.353103 g.353103 ORF g.353103 m.353103 type:5prime_partial len:272 (+) comp63651_c0_seq1:2-817(+)
QFEQVSKLIKLRNDLDTERAVFVVERGGFDTHNTWDMYPMFNDISTGLNSFVQEMKTQGV
WEDVTVMTVSDFARTLTSNGQGSDHAWGGNHFIAGGSVNGGKMFGSFPDTLTAGGDLNIG
RGRLIPTTPWEAPWYGIAQWFGVNETQMSGVLPNAANFPPEQIFNQTELYKSTNNQTTTN
YNIGWKVPAFPRTVTNIKVGDTLTFTWLGGFHDLYQTTSSTCNYNGATMLAARTSSNGNY
VWTATSTGIYHFACTVGSHCGGGMQVSVTVV*

Choanoeca_perplexa

>m.299906 g.299906 ORF g.299906 m.299906 type:internal len:96 (-) comp114241_c0_seq1:2-289(-)
VSTPSTSFSGTVSAGQTVLWVWSDSFPHNVVSGPVGSPDGKFNSGNFVTQSSFAWTFTTA
GQYPYYCGVHSSMAGVITVVEASTTTSTTTTTTTKP

Microstomoeca_roanoka

>m.293472 g.293472 ORF g.293472 m.293472 type:5prime_partial len:79 (+) comp225509_c0_seq1:1-237(+)
VEFIPEGASGWTSAMGENFTTEPLTVEGVYLYKCDPHWGAGMGGAIVVGEPTNLEAIKAT
DPKGALGRLVRKTDKALK*

Savillea_parva

>m.132052 g.132052 ORF g.132052 m.132052 type:3prime_partial len:144 (+) comp11323_c0_seq2:224-658(+)
MSSSGPPFSSAWLVCICAAALVRSGEACDVTVTTSGLTFSPASVVIAVGQRVCWTPGSTH
NVQQTDAASSCTLATSPIFGSSTLGERVEHTFTQTGSFFYRCGPHCGAGMLGRVVVTPTV
APTTAVTTTVATTAAPPPCDVTVT

Hartaetosiga_balthica

>m.141534 g.141534 ORF g.141534 m.141534 type:complete len:260 (-) comp40844_c0_seq1:25-804(-)
MFIQPFVVLLMVFVIATQGREIDVNWGIDTWPQASISISMGDTVKWTWSDGSPHNIVSGS
GRIDDGLFTSGALVAQLGYTFNFTFTTPGDFSYFCAQHSFMLAKITVNPTTSGSTSSPPS
PTPQATMPTTATTTLPPFNVDCSTKTSSFECEAVRTCIFNFDKNKCRNKTCLESTQKPTC
EALSGCKFNSTISICYEEGTDIPCSRYTQEKFCPPSRCLFESPMCFEEGSPPCTSFSTKE
ICLMYNETCAFGNASFFNL*

Salpingoeca_dolichothecata

>m.45680 g.45680 ORF g.45680 m.45680 type:5prime_partial len:333 (+) comp17440_c0_seq1:2-1000(+)
FFWLAIACALMFLLLAVLLLAATTLAQSDACSSEGLCTVSVVAFQFSPRVLEINAGDTVQ
WQLGFASVTQGTAPFSCEAMNDTDLKSSGGDSYSYTFTNPGVYNYLSADECEEYSMYGQV
LVADVGAISASSTIEDKTGTLWTLVVVLVIFPLIVLIFLTYFAYTTHEMGSFARVRAQEQ
LKRLQKSQAGEEKVWMDGSRHVPHIEVNLDLAIGDDEVSNEDQQSYDEFYGRLCILRHDR
NTFAAMLTEMKKRLEAAPGDQEKLEKYTPIVTDLTRLLKLMKNKTATDKSMPADGMELLV
WAHGTLERYLEQQAAKRRKREARQNPHLETDT*
>m.74764 g.74764 ORF g.74764 m.74764 type:internal len:223 (-) comp20455_c0_seq2:2-670(-)
SMYGSWLSVLFLFLLCMRCKGATHTISWGFDTSGASIEISVGDVVTWLWDQDPAFHSVTS
GSDQVPDGRFSSGAPQGSGTNFSVTFSNENNLGTGSYPYFCTFHPSMTGTITVTVPKNFA
LCNLPPRSLSVEQCVQSDLLCLWGGRCGANCSGIDTLEVCGEFPHCTFIGGSCGLAGNPP
DPTATSTTAADTGTTTPAIDINSTPAPTTTTTLTTGTTTATTT

Codosiga_hollandica

>m.146013 g.146013 ORF g.146013 m.146013 type:internal len:99 (+) comp52698_c0_seq1:2-301(+)
VNDSIYITAKNQVYLVSQPTTFQIVQTSTGWNPAAITVNVGDTLNFTWPSSSLDSVSWVM
ADLTTSLESSGSPTLGGSAQFTVWQAGVVFYLSASKGFV
>m.174300 g.174300 ORF g.174300 m.174300 type:internal len:129 (-) comp53300_c0_seq2:1-387(-)
NKMATVRIALHVADQQPAYVNVGSGSGWSDVQAFARAAAPALFAPHTQFHWSYLDEDNDQ
VTIQSDDGWFEAVRLTVAQGTATLHLSVKPLIPAPTTSKACAPSKSCCPPKQVATTESTA
NACCPPKTA
>m.174303 g.174303 ORF g.174303 m.174303 type:internal len:85 (-) comp53300_c0_seq3:2-256(-)
HTHTHTHVVNVGSGSGWSDVQAFARAAAPALFEPHTQFHWSYLDEDNDQVTIQSDDGWFE
AVRLTIAQGTTTLHLSVKPLSPAPT
>m.307646 g.307646 ORF g.307646 m.307646 type:internal len:190 (+) comp55321_c0_seq9:3-575(+)
TTTTTTTMTTPTTGKTFTQMWNTSIGLSNFLQISAGDSVVFVLDSNQHTVNITSTSSVVA
NSGILAGGSAWKYTFQQGVFSVSCDLHPSMMRIIVNAGPVPSSNLTTQTTQVGPPTTNTA
TTSIPSTTTPDFSPSNILLYWNTSIANMHPNTVQLFAGDTLVFQTDSMIHSVNLTMNGGS
DVSTGVILII
>m.325046 g.325046 ORF g.325046 m.325046 type:3prime_partial len:225 (-) comp55548_c1_seq1:1-675(-)

MTETLAWSFGLINAAGKYLSQETFGFNINAQGQSLKRKSIWWIESEPGSDKVYFRSYLNK
YLTANAAGVFAGNADSKGPSEQWEIQVQPDGRWALLSAFGYYAGGTEAALTAYTKTISED
RLWIVQLAMHPQVNLKNVNRKRYVHLEAGALHAEEDIPWGDDATITLAFFKEGKYGLQAS
SGEYLSSSGALRANPADDTKFILKFHGDQVSLAGNNGLYLTALGA
>m.367869 g.367869 ORF g.367869 m.367869 type:internal len:232 (+) comp56088_c0_seq8:1-699(+)
IDLECPPCTSSTTTTSTTMTTRRTSTTTVPTTASTTRQPGTCSNHFECADGLEYCDRTG
HCYICESCSFYNDALDNHCPVCTTTSTTTPRPTSSTTRAPSSTSAAPTTTSTPGPRNYVV
NWGYSVPTSTYSFTINVGDSVTWMGSDTFPHNIASGANGRADGVFRSGDIVVGVIYTRDF
PVPGSFPYFCQAHPGMAGVITVVAPANSASSAASSSSSTSSTTTSTTTTTTT
>m.80985 g.80985 ORF g.80985 m.80985 type:internal len:128 (-) comp50714_c1_seq2:3-386(-)
TTSTTTTSTITPVPTLCEVQVLPQQVTVEWSISLGQAPLAIRVGDSIVFHTDSRPHTVDI
GMINPATGLLVRIVSSGLLPGGSSYSFQFLYAGNYSITCGVHSSMVLTLGVSCPPTATTP
NTTTTTTT
>m.929775 g.929775 ORF g.929775 m.929775 type:internal len:175 (-) comp168977_c0_seq1:2-526(-)
GNTFTNGVFNNGLQTGTLGFTVPQTAPDTLYYICQYHSGMAGTITIINPPVATFTVVSDG
FSAYNVDGSSNAALTLQRGLTYAFTVDAPGHPFWIKTTQTTSQNDAVSSSSVINNGAQSG
VVYFSVPNNSPDVLYYICEYHMLMTGTITIATDPAVSTIPPTTTTTTSTTTTTTT

Mylnosiga_fluctuans

>m.258446 g.258446 ORF g.258446 m.258446 type:5prime_partial len:178 (-) comp21551_c0_seq1:112-645(-)
SGTLQPGATYSLTFNSAGWYPFFCSIHPVMTAAINVVPPPTTTTTTTTTTTTPLTIGGQ
VVLFNLTRAGSTFVQGLYVQRGYRNGRPLYARINPDTGTPWMESIGGIPYPVQLGYTPTA
SSYPGCTAVQGWWCWFDFDCLYVACTLVDIVEPLSSSNITWNRYGAGVLPLPVLTWL*
>m.259294 g.259294 ORF g.259294 m.259294 type:internal len:60 (-) comp22351_c0_seq1:1-180(-)
ASPIASAGTLASSGTLQPGSTYSLTLNSVGWYPFFCSIHPVMTGAINVVPATTTTTTTTT
>m.261638 g.261638 ORF g.261638 m.261638 type:internal len:262 (+) comp24781_c0_seq1:2-790(+)
TTTTTTTTTTTTKTSTSTKTTTTQTTTKATSTSTSTKSVSVASTTTTKTTVTTTTATTKA
TSTTKVSAASCAAIGCNVFRATDPCHCDKSCVTYGDCCVDYSTLCAASTSTSSMTSTSTT
TSTTRTTTTMTTTKATTTTTTTTKATTRPPSTVTIPWGIATLDTFPTTVTVNVGDTVLWV
WSDAALHTVTGGANGVQDSRGIASGFLIGLGSTYTWVASAPGTWPYFCIAHPYLAGTIIV
NSPATQATTSTATTTTTTTTTT
>m.272457 g.272457 ORF g.272457 m.272457 type:internal len:113 (-) comp44050_c0_seq1:1-339(-)
NSFDSTFAGSGTLRAGDQYSVRFDLPGEFPFFCKIHPKMMGVIRVSRSKEASSSTTTAVS
TTTTASSTIITSTTSAASTTTSAIITFEPSFKGSTTTSVANGIFTKSQPTTTT
>m.283079 g.283079 ORF g.283079 m.283079 type:internal len:87 (-) comp81472_c0_seq1:1-261(-)
PITYTWTPTAAGSYPYYCIVHQAAMAGTITVVAPSQTCPNSGANGIMQCRNGQTCNVNTA
GWGCCATLGGRGACPADKPVLCAGQSC
>m.283252 g.283252 ORF g.283252 m.283252 type:complete len:82 (-) comp81911_c0_seq1:18-263(-)
MGGPVTLRIKEGDTVVWRIHGRHAVNSGHNGIYDGLFTSGRPGMREVFAHTFKQRGTFPF
FCPAHAGILVGKVIVGSESAL*
>m.283321 g.283321 ORF g.283321 m.283321 type:internal len:101 (+) comp82025_c0_seq1:2-307(+)
VVWALSVDARPHTVTASPIAGAGILASSGTLQPGATYSLTFNSVGWYPFFCSIHPVMVGA
INVAPVTTTTTTTISTTTTTTVDPLRCGASFGNAACPASAP
>m.74191 g.74191 ORF g.74191 m.74191 type:internal len:101 (-) comp7758_c0_seq2:3-305(-)
VVPWGLPSRPGNLTVTVGTTVVWALNVDTRPHTITASPIAGAGTLTSSATLQAGTTYSLT
FSTVGWYPFFCSIHPVMTGAINVVPTTTTTTTTTTTTTTT

Monodomain cupredoxins in Sponges

>Hpop_Cu_30906 H. populiferum

RPSAGQTHTVDAPAGVPGCEETNSCFTPADITINAGDTVEWNNIDTAAHTVTSGNPANGP
SGIFDSSLLMADATFAFTFEDAGEYDYFCMVHPWMVGSVSV
>Hpop_Cu_44825 H. populiferum
RPSSKIGKNFAITFEKPGVYGYKCTPHYTSAMVGLIIVKGEGRKEDWQDNLDAAKAVKQR
>Hpop_Cu_54677 H. populiferum
TFFFFVLPLLVVVGSPTLATEYDINIPSGDPGAPSESTTGVTTGEITVYPRDTVSWHNGD
GPAHTVTSVTQSGEENGIFDSGLFHAGESFTWQFEDLGDFYYYCYLHSWMNGVVHVTDNL
GSVQTIDKIASGKKKKV
>Hpop_Cu_57484 H. populiferum
DLRPGVQAMTFNGTNPGPMMVVHEGDYVELTLKNPKTNLLAHNIDFHASTGALGGGALTL
IQPGQQVTLRFKADKPGTYIYHCAPGGFMIPYHVVSEKRKK
>Hpop_Cu_32908 H. populiferum
TFGRAKAICSFFVLFAIAAVSSTPAAFTEVTIIPNTGTDDCVNIEYGCYTPGIATVDLGG
KVIFSNTDSKAHTFTAGNLRDGPSDVFESGFLASGNSAEYIADTLGEFDYFCMVHPWMAG
MLI
>Avas_Cu_98067 A. vastus
MKGLHTFAAGCIIYAILFAYTLGSLFFFIGFFIRFPYGSWEIKSLRRNQPWHTLDTEGKY
MFICCPHGMFFLSNLANINLNPLALVADIVFRLPSMREFTLLIGGINASWKSVKNASEKG
RSLLIIPGSTREMKYSRKSKYSLPYVFEWEWYKRLFGHYIFITMGEYELFYPKRTTVHHI
VGEPINVVQPSEEEISELSIKFYQGLEKALEIYNEKYKKSIKLEYAP
>Ifas_Cu_19075I. fasciculata
MRNGSYSPFTKEHCACTQVINITKGECIELVITNYLGDNDTRIESSHPVHLHGHYFDVVK
IGYPSYTSADYTPLHRKYNNRNKDVECKINNSTGKNVSCEHFITVKKNETIQEVVWAKNQ
SSNFESNKTYAEKDTVIVPFGGYTVIRFEADNPGWWFFHCHIEIHQLEGMAALIQEIDPN
AVQVANSSQVGSNSGCHNNANCFFYMTIYVIAS
>Cnuc_Cu_19759 C. nucula
IPGEGAYEDVTVSKDTTVFFKWTTGYHNLVEIRNVPTRDVCEFANTGASEVGQVEKEWKG
APSSTGEVHVSDLSVGEHHFVCTVATHCVRGMKVRVIVTEDDNSTTEY
>Cnuc_Cu_29902 C. nucula
TTYPSWPFEFSSSAISHYSTMDVDWNIAQNERSTHGVYQVDQLAYKTCNLSNGIVWHHTA
EGTVSIAFKENRTWYLVDAVEHNCRSGYKLSVKVGNIHEIPGEGEA
>Cpro_Cu_568567_9 C. prolifera
LFRWSGSYHNLLEMSSPIDSDCKFVDESFTIGQVQVGWLKNAASSGNVLVQALSAGEHYF
TCSVGDHCQRGMRLTVRVEPELQPNLLEDTRDMSRTVHTVRWLIQDYQDM
>Cele_Cu_55819 C. elegans
KDPAISGEVLVNGLSVGEHYFTCSVGDHCLRGMRLTVRVVSGQQQTLTQETQINVMPDHT
VRWLIQDYEDLTIVEGEAVIFAWDGFHSLHQVTESMYTTCNSSAAPLHVWEDASVDNRAT
VRNLAPVLSDPVPAECADNIDCYFSYSTNPAHTPQLTRVDVSEAIT
>Cele_Cu_86692 C. elegans
ILGLIANPAGQAFADVGHSGKTRTVQFVMNDNYYEPESIAVKKGETVRFEIENKGVLVHE
FNIGTAETHAAHKEEMLMMMQHGVLSPDKIHHDKMDGGDHSMKHDDPNSVLLESGESANI
IWKFTDATELEFACNIPGHYESGMVGTLTV
>Kvar_Cu_250112 K. variolosa
SLGEHYFACSVGDHCLRGMRLTVRVESGAQQTESQVATTSEHTVRWLIRDYEDLTITEGD
SVTFVWDGFHSLHQVTEAVYSTCNSSTAPLYVWGDPSVDNRVSVEYLTPVLSDPVTAECA
ENIDCYFSYSENSEHTPQLTGVEVRK
>Cvar_Cu_11918 C. varians
SILVHKLNPDIVWQRDDAADVIRDGDRVMYSIAPSFSLEKFTVRQGDEVTIYVTNLDDID
DLTHGFCLSNFGISFEIAPQATASVTFIAERPGVHWFYCNWFCHALHMEMRGRMLVEPRA
A
>Cvar_Cu_977 C. varians
WGEPSVDNSATIDGLVPGSYYFLCSIAGHCDAGMKIKIVVLVLENGIDASCAENVDCSFS
YSAAATPQLVQIQPAAAVAGTVFTLRGCGLVPQGASVGASINSITKAPCENVVFHSFMQD

IEEIQCIISDYESGHYFVDVFVDGKGYASVNPAELVPGTIRNIYPVLFIT
>Pfic_Cu_52139 P. ficiformis
MEQTVTVDMPDGFPGCEVDDTCYIPADVSINAGDTVSWINSDTAAHTVTSGSVTDGPTGI
FDSSLVMVGAVFEHTFEEAGTFEYFCMVHPWMDGTVTV
>Pfic_Cu_53194 P. ficiformis
PPVFPNTVLEPLAGTAGCEQTDEGCYNMPILTVAPGTTITFSNTDNAAHTLTSGSPADGM
TGVFDSGLVLVGATYEFTIDEEGEYSHYCLVHPWMIGQIIVEATHGDDDAMMGDDE
>Pfic_Cu_43166 P. ficiformis
EHYIVCSVGSHCKLGMRFTVTVVPEGEENNFIEDTDQTTVYTIPWYIRTYAPLTVVEGEK
FLFAWDGFHSLYQVTEEVYDSCNTVAIP
>Niph_Cu_59056 Niphatidae sp
MPGNYSVENTGVHPTLGAWEPSVIEIEQGSTVTWKWSGHNLANPVEFEVHQTSSLTSSTP
IGFASEQSVEGSFSYTFTESGTFYYSTDLTISLSPIFGTIIVKP
>Xtes_Cu_102830
LALAILGVAVAAAPATVMASDGAPSVVVDIPQGYPACADDDSCFGPHTVTVGAGDTVVWT
NSDGVLHTVTESGGAFDGWMLPGEEFAVTFDTPGTYLYGCTVHPWAGGVVVVDQDAMPDP
AGIAQDDVPEPVREDLIIMYDME
>Xtes_Cu_102832 X. testudinaria
MKPVMAVLVFGMASMAAPLTAMAGGEGSHPTAGVSVSEGYPQCADTDSCFVPHTITVEPG
TAVTWTNHDDVVHTITENSPVPAFDGWTFPGEEFTFTFDESGTYLYGCTLHPWASGVVVV
DQELADQVVVDQDAAAPEPADVPEPVMQDAIAMYDSEGVAAFETLTVVAEGAFPQVVGLP
AIFLHDADRPLADVLDELEDSDGVWVDYIFHNPRTSSDEVKTSYLVLVDAMLLLYGVVGD
GASVQTYASNVPFVLDAGTLEIVAHANPNLSGGDIRDAISSGQSLEFVSDMLDRYGSLWL
SYPSADPAPGVEYTRVYMVLRD
>Xtes_Cu_102833 X. testudinaria
PGMAVLVMGVAAMAAPLTAMAGHPTVNVAVSEGYPQCADADSCFEPHTITVEPGTAVTWT
NYDDVVHTITESDPVPLFDGWTFPGEEFTFTFDESGTYMYGCTLHPWASGVVMVGSGATQ
EDTADPEMADPEVVEPEVVEEDNPQLAYDDELISLYMDEGAGAFDTYNIVAHSQSPPFVG
FNVAPLLEKASIPIEVMLEIISQEDEGVWLSYPLPDPTGNIIGYERGWMKLVEEMIRIYN
IDPENTSFMSDDPSYPFVTHIESKTIVAHGSNLDRVGTMSVILVDSSVPYEEFLEMEEGD
GVWVEYVFTNPTTGEDQSKRSWVVVHD
>Xtes_Cu_102835 X. testudinaria
AITLAVLGVAAAAAPATAMASTGGMPSVTVDIPEGYPKCADDDSCYDPHTVTVGVDDTLV
WTNSDRVLHTVTEVGGAFDGWLLPGEEFAVTFDTPGTYLYGCTVHPWAGGVVVVDQDAMP
DPAGIAQDDVPEPVREDLIIMYDMEGATAFDALTVVAEGAFPQVVGLPAIFLHDADRPLA
DILAELEYGGAWVKYVFNNPRTASYEDKTSYLVL
>Xtes_Cu_107017 X. testudinaria
MLKKFAALTLMACAIFTVIGQPARASPPIPTPFTVRMIDNGPFAYDPIDLTFSVGEVVNF
TFIGEDAFHTFTVPDLDIDVEVEAGETTGLDFVFEVAGTYELTCIPHEALGMVGTITV
>Xtes_Cu_123071 X. testudinaria
TQVGIRGSTFITGGLDEPGLYVFVCKVIADDPATQIHLGNVNKASGGATIPLLDLSDSLT
ILTRTASFPTTVQVSPDDPGTLTFFVITDPANPPAPLTTNDNDTWIFLVGAETVQGIAGL
TNATNPDTGA
>Xtes_Cu_123795 X. testudinaria
GKKKRETPAPGGQISSAGITVLDETYIIDSAFVYVKNAPRGKYAVPADEDQKGCVYIPHA
LALQTNQTLKILNSDDTTHNVHALTNINRKFNDSMTEEGSQMSKTFSKQEIFKIKCDVHS
>Xtes_Cu_1283 X. testudinaria
QTYTVIMPEGVPGCEVTNECYIPADVTISAGDTVEWVNADTAAHTVTGGNLTDGPSGVFD
SSLIMVDAIYAYTFDDAGSYDYFCLVHPWMVGSVTV
>Xtes_Cu_154742 X. testudinaria
VVIYARVRGADKNPVMDQKACAYYPHVLAVVAGSTVDITSSDPVAHNVHSHAKKNDAINY
QIPQPGKVIPHKVAKAEEIKFTCDIHAWMTGYIVAVPNNTGYKNADSGWISS
>Xtes_Cu_160846 X. testudinaria

PTRELTVLVGGANEYFPRHLEIRAGDTVTWRMNGKADDPHTVTFARDLDEVVDIVPGEFI
FAPVLVDPTRRPGAPVEEYRAGGYFNPMIDTFSLKFPKPGTYQYICGIHEFHRGTVVVKE
ATPNQEDITRAA
>Xtes_Cu_163915 X. testudinaria
LRVYRPIIVGDWRDFRGAPEALMEAGECFDGPRTLIDVGAAVKWQSESDEMQYIDFENPA
AVGGLRGREEIAPGASVSRTIDAAGTYSYVCGSPGNSE
>Xtes_Cu_17449 X. testudinaria
MTANVTNAKGVPGCEAIDKCFVPHIVTINVGDKVMWSNIDQSTHTVTSGVLTDGGHDGVF
DSGLLKFGDRFSHTFEEAGQYPYFCTVHAWMSGLVIVQDSEEGDGTDPDIDDGGETAGGG
AEETPDAFDDRQIAELEDVLGAGAAYFVTTWETGFPGDSITIPVGGAAGTYTVDWG
>Xtes_Cu_180680 X. testudinaria
GIIMFVGPDTYMVAQPFYEIRILEGEGGVPDFEPDAAVVPQGHAIRWTNEDPVVHTVAST
VGFGELFSSEIISKDGTYTLDTAGMEAGEYEYLCTLHTWMTGTFVI
>Xtes_Cu_27655 X. testudinaria
LGAIFAMAAAGILSVGFGLVGPDTYLIPEPIIEATMLLGNPDYEPDLLEVPQGYGVQWTN
EDIIAHTVSSMADAGATFDSGLVAEGDTYLLDTATLDVGEYEYFCIVHPWMQSLLIVGEP
EAVEDTPEAAQE
>Xtes_Cu_32871 X. testudinaria
EPAPVVAMMAENLMVLEEGANPEIRVDSGDEVTITVTNAGRSFHSFGIVTNPDNPASVVW
GSEIAAPTNPLKAGESGSATFLAGSPGMYHYICTVPGHQIQGMKG
>Xtes_Cu_32872 X. testudinaria
AGGPNLMVLEEGANPEIRVDSGDEVTITVTNAGRSFHSFGIVTNPDNPASVVWGSEIAAP
TNPLKAGESGSATFLAGSPGMYHYICTVPGHQIQGMKG
>Xtes_Cu_36302 X. testudinaria
MTKALLLVASLLVLTAGFTAPRIPPTPVEELSIMNFQHADATIKAGTVVIWTNNDKPLHT
VTHINVGGERLFDSATIAPDVGFRFYFTEPGTYDYQCLIHPVNMKGTITVTE
>Xtes_Cu_40331 X. testudinaria
ISVRKFSVKSVAALLAAIVAIAAVGQAARVSPPIPTKVTVQMIDNGPMAYDPVDFTFSVG
EAVNFTFIAEAAFHTFTVSDLDINVEVNAGETGGLDFIFEEPGTYDLICVPHEALGMVGT
ITV
>Xtes_Cu_49327 X. testudinaria
PGTAVTWTNHDNVLHTVTDSASDPAFDAWVNIDEEFTYTFDEPGTYAYGCTIHPTARGVV
IVDSVESQEETDEPAIPAESEENLAYDDEFIALYMAEGTDAFESYTLVAHSTNALYVGLP
VTPVLDQAFIPIDVMLDILAEEADGVWLSYPTADTQGNLVGYSRGWMKMVEEMIRLYDRD
PETASFMSPDANYPFVMDPDTTMIVADGSNLGLVGVVSVLLTDSTVPLEDLRALNEGEGV
WTEYTFTNPQTGMEQAKRSWVVAHD
>Xtes_Cu_52242 X. testudinaria
MEIEDMYLKRSLLIVLFLLGSLQQQIHVINAEARIFNPDVIYVKPGDNVRFVNMTSHDAV
SINQVPLPDGQVFDSKMGNDFTLQMDIEGVYPYVCIPHIGFGMVGVIVVGEPVNVDAAME
AARANLKGPYRRLIGKILKVQR
>Xtes_Cu_52934 X. testudinaria
MFALASIGEAARVSPPIPTPFTVNMSGVDPGVFDPVNLTFSVGEAVNFTFFSEDAFHSFT
VSDLDINVEVNAGESGGLDFVFEEAGVYELICVPHEALGMVGTITV
>Xtes_Cu_53246 X. testudinaria
MAKRSYVSWATPLAAQDAGSKMFLPSVLAVSQGDTVSVTVINNIPGDPPNHGFAIPAFDV
ETVVNHGEKKTVEFTADKAGIFDIRCQLHPAHVHGQLIVED
>Xtes_Cu_54960 X. testudinaria
VGYSIGIAAVAAAIAAFGASEVSTPTLPVIPVEEPEMVWDVISPEGVAGCEVTDTCFIPS
MLEISVGDTVAWYNNDVAAHTVTSGSPSTGPTGVFDSSLVMAGTVFEHTFEESGTYDYFC
LVHPWMVGTVQV
>Xtes_Cu_6593 X. testudinaria
MRQALRKLIVSALFAAGVLSAVGLLVWYDVVSTPTAQAEPHIVMPTKRPGCEEDSLCYIP
DVIAAEAGQTIIWRNIDVAFHSVTSGSYGEPDGLFDSGHMEPGDTFSYTFGESGTYRYHC

TLHPWMAGVIHI
>Xtes_Cu_67971 X. testudinaria
MTIMLAVAASIVFYQVFYLLLKPSVDEHILSEWVVNMIPGDAQQQDNFMPKKIDVQLGID
NHVKWDNLDDTAHTVTPDHRYEDPYSGEFGSDGVIMPGESYEFLFTDPAEIKYYCIPHPW
MTGEMIITKQR
>Xtes_Cu_87561 X. testudinaria
LDINTNEQHCITTTKGAKSEELVGKVVVYVRVSGHTENPKLDQKDCRYLPHVIAVPTGTT
VDLTSDDPVAHNVNAHATKNEAKNLLISKKGQKFPYTLTKAEAIKLTCDVHAWMSGYIVA
VDSNTGQKDEKGNFIHPDDYEKSDDTGKYTIKDVATGRARVVVWHETLGTANKTVQIPET
GELTVNFKSSEFKKPKTK
>Xtes_Cu_116295 X. testudinaria
LPLPEVQWLIAGAGALVLVIMCYSIVAFRGQEDEITVIPLIVVTIYGVIGNEMTVEVEGL
SWFAYPDHGEFQTTQMVVPVNQPLLLSMTSVDVLHSFWVVEWRVKQDIVPGISTELRVTP
TEVGEFQLRCAELCGLRHAYMLADVRVVSQEQFDAWIDEQ
>Xtes_Cu_140051 X. testudinaria
NWMTYPGPDGTEDDLDLENQLHVPVNKIVHIRLTSIDVIHSFFVPNLRLKQDALPNRFVN
VWFEATKPGTYEIPCAELCGFQHSGMLGYLNVHTQEDYEAWVAERWAQ
>Xtes_Cu_142493 X. testudinaria
TVTPVAWADHHAEEEAAAGDKMFVPSVLVAEQGDTVRIKVVNKIPGEPPNHGFAIPAFDV
EEVVNSGETKTVEFVADKAGLFDVKCQLHPAHLSAQLIVHEGG
>Xtes_Cu_152644 X. testudinaria
TDKDEVLSRSFSNSAVLDLDYAAVITYRDGQYAPSRVSIPVGGQVLWVNEDQVFWPAANL
HPTHKQYPGSNIMKCRTSERIMIFDACEAMGPGAAYAFTFNQVGEWRFHDHINP
>Xtes_Cu_155756 X. testudinaria
DAETITVVGQFWTFEHEGTKEVGELHLETGKAYRLEITSTDVTHSFNIHDYVVLLDAVPG
RVNTVWFAPTEAGVHDIQCREYCGLIHYNMRGTLYVEDPT
>Xtes_Cu_47395 X. testudinaria
IHIRFSLWGLLLLGVAGCTPAGPLRIQITGEIWFRYPGPDTDDDIIKPGELHLPLGLAIE
LELTSRDYIYTFALPHLDLHQVAVPDLTFALTFTPQTTGAFAFVGDQMCGFEHESLNGRV
VVESPAAFVDWLAGP
>Xtes_Cu_55624 X. testudinaria
ALALAVLVAGVAAAAAPAALSEVTITPVPGVPGCEETPEGCNVPMVAAVDVGGAVIMSNT
DNVAHTFTSGTVDGGADGAFDSSFLLPDDSFEWRPDTPGEYPYFCMVHPWMSGMIVVQDD
GDADADRHAGSDMEADHHAGEGMGADGGDASDAAATATWISEPMPDEVMKIVIEFAGIHH
VNYLITATQSGSEEVHDHDSTSIHKTAPLGSSDLIDVKITFEVLVFEDIKPATIEMMEST
APAATGMLTASLWATEPMADQRMTII
>Hamb_Cu_4607 H. amboinensis
MKRRRRSMSFFLLVVIGFNCFLPTPRRTIYEWWTPGHLATINNVVNVYLYDTLILKCNNT
VSTVHYSSSQAAYNSCTLSQFSEVGACQHGHTDISIAIIPGLPQGIQYQEDTTYYYASSH
SNDCSSGMKIAMNVVASSTNALTPTATNTQSATPSATNTASVSPTAGGTGEGNSGSFLEH
KRQHKEEILMSRMDSNESSEKFIGNNNGNYKDTSSSFKNGNSPKHPPPAPPTSNGISPVI
NGSKRKAPGPPVSKSLGPSTSTRSSGPAPSVTKSMGPTTYRGGPKSSAVKPTSIGGPPKN
RLAPKSSVEPASLPASLPNRRQAPLPPPPTAPVSRGGKNKL
>Scil_Cu_89286 S. ciliatum
MFVCEPAVQTELSSATAKEYVVQITRKGFSTSALVINEGETVTWKWASYEGTNGHNVVQV
DAMAGGWNIFDVSLSGNNLHVVKGGLTSGKVVGTGEYSYTFRCQGAYHFISQGLSKRRSV
LTITVQKRPSQIIRVADDGFHPRTLRIDTGTAVMFVWEACSLPRAVRPLRNCEGECDSDC
MDNNTMMMRDTSGTYQEVFSEPASLDGTARHLCIVAVQRTVNVSDNGFSPALLEITSEEK
KQCKKGQYVVRMLDGMPEESAAALKS
>Ocar_Cu_134339 O. carmela
MGCSRPSVSSLLSLCTGRDVEILVDSRSFTPSHVVVRSGQTVRWQWLEGVHAIHQVSTDG
KKIFNVEGGVKSGQMEEEGSFRHLFKAPGLYQFISHGHSIPSVVSVEVIKKPERVVKLSD
DGFSLDSIHVCVGEKVRWRWDDCRRPQSMSQARFNSRSGELEVLYDRNQAPPSLTGSFSF

TPTGASYATGTCRLFIVKATEEAQVRDDGIVPASIVITEGDKKHYSVHLFFYSPGRSQKL
SVVELRH
>Ccan_Cu_109514 C. candelabrum
ISSFFVLFALVAGAMTPAAFSEVTIIPADGVPGCEETAEGCYIPTEATVDVGGVVIFSNT
DSAAHTFSAGSAAEGLTGEFDTGLLMVGTTYEYSPDTVGEIPYFCLVHPWMVGTIIVQEA
GGADEGDDHGDATVTGMWASTPTSGEMMEISIEFEDSEHVNHDIMVTQDGEEAAHHHDGK
GMYTTAPLSSSEPVDITITF
>Ccan_Cu_125022 C. candelabrum
VTWANEDTVQHNIQSQDEFGKISGVFNSPPLETGDRFKFTFEEEGIYNYYCSLHPWRAGA
VAVKEKN
>Ccan_Cu_114223 C. candelabrum
MGCSRPSLLPVTTCGSRDVVINIQERGFSVSSVAIYSRQSVSWRWCEGFHNIMQVTCDGG
RLMAVKGGIKSGLQRNAGNFSYEFQIPGRYQFVSQGHNPSVLLDVSVVARRPKTIKLSDD
GFSPRYLEAKLGETIIWNWNECVRPQAVRRVYFNSKLGKFEHMDHEINYVAPSICGSYSH
TFKSPASYVSNTTHLCLVKVTNSLEILDEGFDHVLLSVIE
>Ccan_Cu_11800 C. candelabrum
MKFFFYFPNMLELLLAAWKIISWENARQLKFVNKDYVDSNQLLVQFGGTDTWEYQYLPEV
NTETPAKKVRFNMTGQGDEEVSAAAAESTSVCQDESDRRELTELQESEIADAMMDVQQQK
QVQKQALVRRRSVESKPGGLLKRSSSSDMSASRSWLKLKPSDVIEFVGSHKENSQTVLHL
TNATDSTLSFKVKTTSPEHFSVKSSVGIVVPGETIKVNITLHQGHYSLPSVDQLAVLWKT
ISSKHI
>Ccan_Cu_153316 C. candelabrum
IKSPTPDVLVQVTGKNWMTYPGPDGTDDDLKLENELHVPVNAVVHIRLTAVDVIHSFFVP
QLRLKQDTLPNRFINVWFEATQPGRYEIPCAELCGFGHSGMLGYLNVHTQEDYDAWVRER
WPSTQ
>Ccan_Cu_160755 C. candelabrum
GKPVRVILASKDVIHSFFMPNLRFKQDMVPGREIIGWFQANKPGKYEIPCAELCGFGHSG
MKGWLYALAPEEYQSWREKKEIKQKKKKX

Monodomain cupredoxins in Ctenophores

>Patl_Cu_15384 P. atlantica
WPLRPGSLKNEATGSGDKFEAGGMNVGATYKWNNMTFFADMDKKDIEYTNESDPVAKSEQ
TTMTFGAAKIHNVESNARVIFDVRYTNVSAEDVDNAGAKTEKTVTVAFETEANADTNTTT
VKAGATLNFGKLKVDGAIGTATAGSLRLDTAMADVAVTYF
>Patl_Cu_411399 P. atlantica
MKKFLIAFLGLFSFSNPCQVTVEATQFNTKAISVPASCESFTVDLKHVGSLASAVMGHT
>Patl_Cu_417317 P. atlantica
HNIDFHAATGALGGGSLTDVAPGEEVMIRFKLLKPGVFVYHCAPGGMMIPWHVVHGMNGA
ITVLPREGLDGEGNKLS
>Binf_Cu_18230 B. infundibulum
ELEPKIKWKLSHTVDIIGYLSIDNKNLIPKKARSTENDKNPNTNMNILDLQHNKSVTIVI
RNRSFMNHPMHLHGHHYEVLDIALRKVKNCDIEFCPLLDREKRGVLKDTVILPPGGAVAL
RIQSDNPGVWFFHCHIHDHLLSGLALALNERNYMFSQTKFPKDYPSCVYSGNLQFLRTAT
CDCDKDDEQFMKSFENTVKPTLKKNGKRALQMKCSTASGCKVQGH

Monodomain cupredoxins in Cnidarians

>Pcar_Cu_435192 P. carnosa
GIHPEYGAWNPQTIVVNAGDSVQWRWMSPELSGLHFGVQETRDSSPSGYDGKGFKSARSG
SGNYSQQFNLPGTYFYSSGEVDPYGAIVM
>Pcar_Cu_62093 P. carnosa
LKPGMYNFLCYSCMVGVTADGFKPSAVTVAQSQSVLWSWANQGDEEHNILHVRPPQDQQQ

LTRVRGLSAFDSGPKTTRSTFFHTFDVPGRYFITSQGTDSQLCVVDAIENAAYVSPP
>Apal_Cu_7673 A. pallida
MASGISSAVSIPSFGLKASVKPAVVLAIEILLGDNGLAFEPANFSVPAGEKIVFKNNRGF
PHNVVFDEDEVPAGVDAAKINSGGEIYSVTLTEKG
>Hvul_Cu_13222_1 H. vulgaris
NHSPVKITVKSKPVIDIKVTDDGFSVAVIKLYRGDTLRWSWKNCEEPHNVMERKYCMDHG
GYTSLENQENARYTGTYFRTFDTATQSYDESRAFCIVQVLEKS
>Atet_Cu_65930 A. tetragona
AGEAVTFQLKLKNGTPQHSQVYQVYRSEDSVVRVHGGFTSGNELLASEKEWTQEFNLARD
YYFKFGNHKPVKICVKEKPVMDIKVTDLGFQVAVVKLYKGDTIRWSWRNCEEAHNIVERR
YCLEHSGYRTMENQENSRYTGTYFRTFDTPTQSKVETRSFCIVQVLEKQRLKDGGILRKI
VEVTCGWRPSKTSSTMNHSIAIRQ
>Clat_Cu_35282 C. lathetica
CSCYNELTIPFNKTIQMVWTNHGPGANRHHPIHLHGHSFHVLKMGYGKYNSTTGLIIEGN
DDIECIGSTCYSTRWKDNNRKGDRMPGLKITNAPQKDTLIIPTGGYAVIRFRSDNPGKWL
MHCHLEFHVMQGMSMVINEAPEKHVKSPN
>Nbij_Cu_650 N. bijuga
PIHMHGHSFHVLKLAYAEQDPETGKLYTDQKRNHPDIDCGGGLNLCNEPRWKNESWRDGN
ATGLNLGNAPMKDTLVIPPGAYAVIRIKSDNPGKWFMHCHIDVHMIGGMAIVLSEAPDRT
PKPPHYFPRCNDGQDSVVGVTKIDEENKNKDNKDEKEPDTICVESNSYLVPLFAMGTFVV
LLMVVMVTAF
>Pphy_Cu_8558 P. physalis
MLSLLLSAFVTAKRIELDWIIRPTNRPMQEITAAVGDTIVFNWRGRHNVFVLPDCDGKGK
HKWDSFQCPNDGKWGVELGESSPVEYTIPAKYNPGDYICFVCEVGGHCSAGQHTTLLVAG
KPQPEPSTEIGIDWRIPSNKMDKSMQLVHAAP

Monodomain cupredoxins in Bilateria

>Pria_Cu_4381 Priapulus sp
ITAGGFKFIPQEITIKKGQTLRWDNQEKRQYHSVWFEALGEEEPEDYLFPEDTYDRQFDQ
VGKFTYRCGPHPKMLG
>Hpsi_Cu_55220 H. psittacea
RYFFLFSKWLIFRWCGPHHNVIYFSAPKMYENCSFGKLKKKAKKLPLSNNDLKEQFKQYS
AARLGIGKHYFACTVTGHCKYGGMKIIVDVKETCRKL

Other monodomain cupredoxins used for comparisons

Bacterial

>Athi_Rusticyanin WP_065972088.1 rusticyanin [Acidiferrobact
er thiooxydans] >OCX44072.1 rusticyanin [Acidiferrobacter th
iooxydans]
MESVKRARVAKGAAMVAGAVFSVGVACAALVPTSAFKKATLPQVKAMLEKDNGKVSGNTV
TYGKNANVVAAAVLPGFPFPSFEIHHVKNPTLDFPAGATVKFTFINTNKGFGHSFDVTKK
GPPYAVIPQIAPIVVGTGFSPVPAAGKFGYATFTWHPAAGTYYYVCQIPGHAATGMFGKI
VVK
>Pkna_Azurin WP_043255707.1 azurin [Pseudomonas knackmussii]
>CDF86538.1 Azurin [Pseudomonas knackmussii B13]
MFRQLAAVSLLALFSAPLLAAECSVDIQGTDQMQFSTNAISVDKSCKTFTVNLSHPGALA
KNVMGHNWVLTTAADMQGVVGDGMAAGLDKNYLKDGDTRVIAHTKIIGAGEKDSVTFDVS
KLKADEQYTFFCSFPGHSAMMKGTLTLK
>Pchl_Azurin WP_023970237.1 azurin [Pseudomonas chlororaphis

] >ETD35465.1 azurin [Pseudomonas chlororaphis subsp. aurant
iaca PB-St2]
MFAKLVAVSLLTLASSQLMAAECKVDVDSTDQMSFNTKEITIDKSCKTFTVNLSHSGSLP
KNVMGHNWVLSKSADMAGIATDGMAAGIDKDYLKPDDSRVIAHTKIIGAGEKDSVTFDVS
KLTAGESYEFFCSFPGHNSMMKGAVVLK
>Aspa_Azurin WP_050448215.1 azurin [Achromobacter spanius] >
KNE26356.1 azurin [Achromobacter spanius]
MLFKKLCVAAVLAAASAPVLAAECSVDIAGNDQMQFDKKEITVSKSCKQFTVNLTHPGKL
AKNVMGHNWVLTTTADMQGAVNDGMAAGLDKDYVKPGDARVIAHTKVIGGGEKDSVTFDV
SKLAAGTAYTYFCSFPGHFALMKGSLKLVD
>Dtsu_Azurin WP_043790509.1 azurin [Delftia tsuruhatensis] >
KEH09360.1 azurin [Delftia tsuruhatensis]
MAMQWKPVLLALAMTAVAAPVLAADCAVEIEGNDAMQFNKPTISVPTSCKQFTVKLKHVG
KLPKTAMGHNWVLSKTADLQPVANDGIAAGAAKDFVKDKDARVIAHTKVIGGGETDSVTF
STAALKAGEAYSYFCSFPGHSALMKGTLTLAK
>Kpne_Plastocyanin WP_077273885.1 plastocyanin [Klebsiella p
neumoniae] >XP_020180739.1 plastocyanin, chloroplastic [Aegi
lops tauschii subsp. tauschii] >XP_020162516.1 plastocyanin,
chloroplastic [Aegilops tauschii subsp. tauschii]
MAALSSAAVSVPSFAAKAATPMRSSRMVVRASLGKKAASAAVAVAASAMLLGGSAMAQDV
LLGANGGVLVFEPNEFSVKSGETITFKNNAGFPHNVVFDEDAVPSGVDVSKISQEEYLNA
PGETFSVTLTVPGTYGFYCEPHAGAGMVGKVTVN
>Vvul_Plastocyanin WP_017428787.1 plastocyanin, partial [Vib
rio vulnificus]
GGSDGSLAFVPNEFSVSSGDKIVFKNNAGFPHNVVFDEDEIPQGVDASAISMSEEDLLNA
PGETYSVTLTEKGSYTFYCSPHQGAGMVGKVTVK
>Maer_Plastocyanin WP_002788485.1 plastocyanin [Microcystis
aeruginosa] >CCI18491.1 Plastocyanin [Microcystis aeruginosa
PCC 9807]
MKKLGLLVATVVLVVSSFFFNTASAAAETFTVKMGGDAGTLQFDPPTLTIKAGDTVKWVN
NKLSPHNIVFDSTKVPEAQASKLSHKGLAFSAGESFESTFSEPGTYSYYCEPHRGAGMVG
TITVQ
>Gher_Plastocyanin WP_017294320.1 plastocyanin [Geminocystis
herdmanii]
MIKKLGLLLSSLLLAVTMIAVNPVHADTVEVKMGADSGMLAFQPAKVTIKAGDTVKWVNN
KLAPHNVVFDSSAKNGEKLSHKGLVFSAGESFEATFAEPGEYTYYCEPHRGAGMVGTITV
E
>Shof_Plastocyanin WP_017746015.1 plastocyanin [Scytonema ho
fmannii] >KYC38329.1 plastocyanin [Scytonema hofmannii PCC 7
110]
MTFIASSLRRFGLALLTLFLVVSSFAVFTPSAAADTYTVKLGSDKGMLAFEPSKLTVKPG
DTIKWVNNKVPPHNVVFDTAKNPAKSADVAKNLSHKQLLMSPGQEYETTIPADATPGDYT
FYCEPHRGAGMVGKLTVQG
>Ssub_Plastocyanin WP_026079923.1 plastocyanin [Spirulina su
bsalsa]
MKKLGLVLSTVLLLVASFALAVAPAAAETYTVKMGADSGQLKFDPATLTIKAGDTVKFVN
NKLAPHNVVFDKAPELSHKQLLFSPGESFETKFDTAGSYSYYCEPHRGAGMVGTITVE
>Tbou_Plastocyanin WP_038085369.1 plastocyanin [Tolypothrix
bouteillei] >KIE13022.1 plasmid stabilization protein [Tolyp
othrix bouteillei VB521301]
MTFIASSLRRFGLALLTLFLVVSSFAVFTPSASADTYTIKLGSDKGMLAFEPSKLTVKPG
DTIKWVNNKVPPHNVVFDAAKNPAKSADVAKSFSHKQLLMSPGQEYETTIPADATPGDYT
FYCEPHRGAGMVGKLTVAG

>Scya_Plastocyanin WP_015192812.1 plastocyanin [Stanieria cy
anosphaera] >AFZ35141.1 Plastocyanin [Stanieria cyanosphaera
PCC 7437]
MTKKLGLLLSTVLLVVTSFFLTANPAAAETYEVKMGSDNGMLQFVPSSLTIKSGDTVKWV
NNKMAPHNVVFDSSKVSDDIATKASHKSLAFSPGESFTTTFDQPGEYSYYCEPHRGAGMV
GKIVVE
>Rint_Plastocyanin WP_008234430.1 plastocyanin [Richelia int
racellularis] >CCH67623.1 Plastocyanin [Richelia intracellul
aris HH01]
MKMIVKALRHMSLAAVLTVVFVMSSFPVFVSAAAAETYTIKMGTDKGMLAFEPSKLTIKA
GDTVEFLNNKVPPHNIVFDSGKSADKTIADRSSHKQLLMSPGQSFQVSFEGATSGEYPFY
CEPHRGAGMAGKIIVE
>Fmus_Plastocyanin WP_016859453.1 plastocyanin [Fischerella
muscicola]
MKLIAAGLRRFGLAVLTILLVVGSFAVFTPSAAADTYTVKLGSDKGMLAFEPSKLTVKPG
DTIKWVNNKVPPHNVVFDPGQSKSADLAKAWTHKQLLMSPGQEYETTIPADAPKGDYTFY
CEPHRGAGMAGKLTIQ
>Cthe_Plastocyanin WP_015157412.1 plastocyanin [Chroococcidi
opsis thermalis] >AFY90875.1 plastocyanin [Chroococcidiopsis
thermalis PCC 7203]
MKLFAATLRRLGLAVLTIVLVASSFAFFSAPAAAETFQVKLGTDKGMLAFEPSKLTVKPG
DTIEFVNNKVPPHNVVFDTAGTPNKSADLAKSLSHKQLLMTPGQSVSTTIPADAPAGDYT
FYCEPHRGAGMIGKVTVAS
>Paer_Azurin 1JZG Pseudomonas aeruginosa
MLRKLAAVSLLSLLSAPAECSVDIQGNQFNTNAITVDKSCKQFTVNLSHPGNLPKNVMGH
NWVLSTAADMQGVVTDGMASGLDKDSRVIAHTKLIGSGEKDSVTFDVSKLKEGEQYMFFC
TFPGHSALMKGTLTLK
>Tfer_Rusticyanin 1RCY Thiobacillus ferrooxidans
TTWKEATLPQVKAMLEKDDGKVSGDTVTYSGKTVHVVAAAVLPGFPFPSFEVHDKKNPTL
EIPAGATVDVTFINTNKGFGHSFDITKKGPPYAVMPVIDPIVAGTGFSPVPKDGKFGYTD
FTWHPTAGTYYYVCQIPGHAATGMFGKIVVK

Plant monodomain cupredoxins

>Uper_Plastocyanin 1IUZ_chanin A Ulva pertusa
AQIVKLGGDSLAFVPSKISVAAGEAIEFVNNAGFPHNIVFDEDAVPAGVDADAINSKGET
VVRKLSTPGVYGVYCEPHAGAGMKMTITV
>Atha_Blue_copper sp|Q07488|BCB1_ARATH Blue copper protein
OS=Arabidopsis thaliana GN=BCB PE=1 SV=2
MAVFKTVTFLVLVFAAVVEDYDVGTEWTRPMDPEFYTTGKTFRVGDELEFDFAAGRHDVA
VVSEAAFENCEKEKPISHMTVPVKIMLNTTGPQYFICTVGDHCRFGQKLSITVVAAGATT
PGAGATPAPGSTPSTGGTTPPTAGGTTTPSGSSGTTTPAGNAASSLGGATFLVAFVSAVV
ALF

SRS superfamily sequences used for comparisions

>XP_008887044.1 SAG-related sequence SRS11 [Hammondia hammon
di]
MKTASFKFAALLGVAAFAPQLILCSSSDTTAEVKECTNDSLPLTGGAQVSLKFKCADTYT
MGPNSDDDVYAYTNGACTATSQKLNDLVPGSKRSKSAEGSKAMRSTKSDPQSQNVYTLVL
GAAPKEQQVFCYTCTATSSAATVTTVAQDNSKKPCNIIVTVPQASSSGPLESGVTPAVLA

GVALLGTFAMH
>XP_003881326.1 srs domain-containing protein [Neospora cani
num Liverpool]
MAKGQTQKQFFSPISRCDQSYGTFFSRTPLQTALPNGAIVHPALLSSTSAAKVGKSREGS
RCSRTRTVPFEYKMGVLPRVLLVIGISITLSSSRAPIAAAGTELSKTSAQTCDNTGGVTV
QFPEAGRSVQFKCGSPFDTLVPSDPLYVFVAAAAPQGRNHLGLKGEQEVAEDITVAGHTK
KLLRDLYKDATLDHGSGNDSGYTLQIPDTSRTEEGKTLKYFCKRGGKSVLDVAKDACPVT
IRIPAKKQEPGTDDSSEDDPQQPENVLTCTAESGKQKATVSKANQTVQFSCDPPATSTLT
PEAALKVFDNKDGACTKEVDLSTLVPQARRSEREGNGVYTVTFPQLPSEKQALCYSCSTT
GGSGSLRTAACQIHIDVSAATAVSTTTHTTSNATSRVHAAISQAVVGILFVSVAGIACLS
>XP_003883828.1 SRS domain-containing protein [Neospora cani
num Liverpool]
MRLREHGGTPPALLAATLFKIVFLSSLTLFSEVASRSDYTHVCSVGTEEVHAAANSTINF
QCGPMLTLAPEASSNMVYRGTSCKEKAELSWLIPGAELFSADAQYMRKRSQNQTHSSVYT
LRIGDSPAEEVQICYRCKASQINAIYGEAWGDDPVDRGHITTDTLCKMVIRVAAAPKTPA
AAFVANLGRSLSVGLAFLTGLCF
>XP_003881327.1 srs domain-containing protein [Neospora cani
num Liverpool]
MRTIKMAMMRFATAVAGITVLTGCGRVLADPEIPGTDENICTDGKYLNLALPLGQEILTF
KCGPKITTLDPSDPLLVYTENSSPGQGVPLNTVMPGATFQHGQGGSSHTLRVPNSSRPPK
ETYILYLCKEAGAAGENGPVEANDLSSAAPRRMCLVDIKVAAKSPEGETENGTSPPQGQE
DTLPPDVNTCNAGQDKELTVSEPGELKLKCSAGLSFQPEETSAVFDDGDGKCETVQSLTT
LVPAAVRSDQKGIVTVGIPRLPSTGNKAICYQCTSGEADNSDTRESSRTETSCKFRITVL
SSTSADPESNGDGPIFRGSKSMAAAFFSVVAALATGVMALAF
>AFO54960.1 surface antigen 2 [Toxoplasma gondii]
MSFSKTTSLASLALTGLFVVFKFALASTTETPAPIECTAGATKTVEAPSSGSVVFQCGDK
LTISPSGEGDVFYGKECTDSRKLTTVLPGAPAGRNNDGGSSAPTPKDCKLIVRVPGADGR
VTSGFDPVSLTGKVLAPGLAGLLITFV
>XP_003883827.1 SRS domain-containing protein [Neospora cani
num Liverpool]
MNFCKISCRAPLALKAVAGVFVLVNYALMSNASEMAAPIQCASGETKEIQAPTSGSIVFQ
CGANLQLTPTDGAVFQGEECTEQTKLDSLVPGANLVTKAQKSKSASPTYTLTFDDTPPEA
QVLCYKCVAPTADAGGSGRSEAQGGAEQAQKECKLIINVPGVEGLVTSSGSVSASLTRKC
LAAVLAGLLIALP
>XP_003884599.1 SRS domain-containing protein [Neospora cani
num Liverpool]
MARMAAYLGRWGLVALLATSSAILHASADKAEGSESSTPITCDNGQKSGTAGPGSPLYFK
CGTGMTLKPTEVGKVCEQAACTSEVPLTNLLPGAHLADPSKKTLDREQEQQQKDVYVLTV
PNAPQSKQTVYYKCEGTSAAGVQPPPTSLQARDKKECKITVTVEAAPKVSTTAPQATSDA
TISTTTTEAPVASSGMGAPNYMAVTAIFGAVAAHDLLL
>EPR60025.1 SAG-related sequence SRS47C [Toxoplasma gondii G
T1]
MSGKAGSSFQASRGLGYAIGVVVFGVLVCLPVRGTAADATVVTCKKQDGIVSLALKETTK
LIKFNCEESWTLYPDLSSSEASKFCEDSSCKNPVPLRGIYKLESGDVPVPSGRVPGRVAS
KTYTLSMTSQPEKSETLYFNCRAPEAQQDEAERTRSSQATSGSTKTTCIIQVAVHGSESF
SDGNSETECTNGRTLVEVIPDSGPKKVVKFRCADQWTLSPVNFEKTLDGEQCTTEQDLAD
LHLDANRVEGKSYKEGSQVPAYALEISEFPTGKDHVKLCYKCTKNSEGALRTSDQNDNEC
TIIVDVAPKKSPTGENDEEEQPNENPSTSDSRDHHKSSFVTVGAFLLCSVLGAGQFL
>XP_018635103.1 SAG-related sequence SRS59K [Toxoplasma gond
ii ME49]
MKRQRPLKTISVFFVFCLGLASAPSSIVASVNDSGGSELGDLAGALRGRQSGREAVDGPE
INVCSSWAGISINLTQDQTEARFQCGPKTVLAPLQNPDEKVYLEEDCRSATLLKDAVPGA
EWAAIDETKGAYKLTLPPHRKKAETLYYRCKSEALIVAEISLIWFIKQYHEASEEARKKI

LETLSRYYDLVRVITLVIAGIIERKVKIIKAIKALLQLANQTVHDLLEREEVVQAIEDLY
NYIKQHIPEIDKHLKAIVEAIKGLVDYVKNLISQKLAERWELITELLKNVADATGVTCKV
KIVAKEEPLAGDPGNEKTCSDAKHPVYIKLGKGEREAVFKCGDGLTTLEPSQNTDKPKFC
ESIDCNDTAELETTFPGAYWDERNKKANIYRLVIPTVSRKDTRMYYKCKGTSDSADPCTV
LINVKSTETDDDEEEDVQECTVGTEKKVTLSPTDTVKFKCNLGTVVHPPFSSGSPKVFDD
SDGSCSAQASLTSLVDASLTEDSSHGKYTMYTMNLNARPAETKNLCLQCSSGKQNCKMRI
HVPGTDSTSSGPGSLSGFLSSVVGLVVCAGFFLVY
>EPR62843.1 putative SAG-related sequence protein SRS26B [To
xoplasma gondii GT1]
MRRHLCLLPLFLSAAASFATGSASASSTLSAEINTQKVVQDTLTDPLEGQQCTGDDTTIE
IVVGPKDEETTFKCVEPLTTLVPADCTSDSDTCPTMAVSAKGQIIQRVCEDENCESPKPL
TDVFPGASRTDDPAKHAYTLTIPKDNRPQKDVWYQCRSPLRTNPCKVKISVAAALADPPE
NQQCSQAGQTITVDVGPQLTEAAFKCVDPHTTLVPADCTGTVCPPVAVSRSEHTAPRICE
DANCERPRLLTDVFPGASRKDDTANHVYLLTIPKDNRPQKDVWYQCRSPLRTNPCKVKIS
VAAAPIAPPATSQENKCTNAGEVLNVSASPASPVKFICTKDLSLQPDDKYVYENSDDQCQ
KKVELSTLVDAQLTGVTQPTLTSGDTTYTLTINKMPPKKALLCYKCVGADAHLNSLRVSA
GRDAKPECLVKVTVEADPTATDTSTTPTETPSIPTTSSAMLHGSALTVSHNVAVSKRPSR
NSPRNVEISVATTLADLSEDHNCYELKGSIAVVVGLKPENQTIKHGGAMTNIVSANWADG
PCLELVTSRDVQTV
>KFG57154.1 SAG-related sequence SRS47B [Toxoplasma gondii R
UB]
MSFMNSRFFQVLGRRPCVVGFAIAAGLVCLSACGTKPVSETVVSCEEAPKFVSLSLPEQN
SSLMFKCPDGSSLFPDEKEGTSTQYCKDASCSQTAALIDGLKLQEVQDADVDDAHSEGRA
TAAQAKAYKLTVETQPEQAHTLYFLCKSTDGTLDESLPAKDARDPHPTSKTTCTFQFPVY
SKHTLSDPPAEECKEGQTLEVASDFKPKKVTFRCADNWTLSPANFEKAYKGEACADDSEA
TLADLGFGAKRVEGKAATLSAKSPAYALEITDFPAGPNNLKLCFKCKRLSDQKQITSEPS
EDECRIIVDVAPKKTQPDEEHGEDGPAGDEKPGDESTGQPSTDGDGNEKPSTSGASDHFK
HSILIFGALLLASVLALRPILA
>XP_002368205.2 SAG-related sequence SRS29B [Toxoplasma gond
ii ME49]
MSVSLHHFIISSGFLASMFPKAVRRAVTAGVFAAPTLMSFLRCGAMASDPPLVANQVVTC
PDKKSTAAVILTPTENHFTLKCPKTALTEPPTLAYSPNRQICPAGTTSSCTSKAVTLSSL
IPEAEDSWWTGDSASLDTAGIKLTVPIEKFPVTTQTFVVGCIKGDDAQSCMVTVTVQARA
SSVVNNVARCSYGANSTLGPVKLSAEGPTTMTLVCGKDGVKVPQDNNQYCSGTTLTGCNE
KSFKDILPKLSENPWQGNASSDNGATLTINKEAFPAESKSVIIGCTGGSPEKHHCTVQLE
FAGAAGSAKSSAGTASHVSIFAMVTGLIGSIAACVA
>XP_003883569.1 SRS domain-containing protein [Neospora cani
num Liverpool]
MATHACVVRRKADAACFAKLSASQSCLTAKSVNRSVSVFALLFGVVLAVGVSGAPFKSEN
EKFTCLPKQGNADQWVALVYDSQHSITFACDGGTPLPSKLLSEDDGLIVCNESDGEDECE
KNAAPLSTFLPGAKKEWVTGTLQQGIKITIPDEHYPATSKAFRVGCKAGKNVCLLNVYVQ
SRESEVIGQVAHCAYSSNVRLRPITVNPENNGVTLICGPDGKAFPDDYMNHHCTELDECK
ERPYSAVFPGFSSSFWTGEASGVAGATLTIPKDQFPSTAQTIYLGCTGHPDDKQVTCVVP
VNIEEVAKPAGAGSNPGGGSQPDQSSEKRDGEQVNKGKPPTGGSGGATTGKQNASQNAKD
KGETGGENGDSPVLRGDACDELPSYVALSAASLTATAIFAY
>KFG51369.1 SAG-related sequence SRS29A [Toxoplasma gondii p
89]
MVRTSLIEHRTGRRVVAAPDRGSLQVCIVFNIALSATIAAMMTSPLLVASMTSPLLTWDG
NKVTCHPEKGDVDDWIGLVPQQNEVTFACEGKGVTPVPKNLVNSKSREVCAQGMSANACE
KDPRPLSEILKGAQNDWLNGEDLSRGITFRVPKNNFPGLPRIFRIGCRTRSNICMISLHI
YAKSAYTSGQVTECAYESNVSLPPVRITPATNHATIVCGPYGGISPLDYKKEFCTGEPHT
SCGRQKYVDVLPRYTDAWWNGDPSTAEGATLTVPNGHFPKETQTLKFMCLERLDTQPRRC
VVTVEIAAGVSGSKPSGGESIPPQAPGTEGSRKEAPNTNNSGGTPTGGGSDDDTTAPGTG

SSGTDSRGDLGDGRDQDAKGGMSRGNSESGSNGGATQPSRLLGLPSAVIGTLAAAMFGAV
>XP_003882257.1 SRS domain-containing protein [Neospora cani
num Liverpool]
MLDCNEMAGDSQARCLGCYGPGSTLQMFQYYGGQRKLRQSFRMGLTLMVLLASTSTQYDN
PSFFFAARGVPVQAGTPASECETSTSGTTTCTCLTKDSKTKALYAALSEDTNVLQVSCQR
ELHCAPDDLSGQKVCPGTTEQLHECTSAPGSPTCINVNSLLTSSESSVTWDTVPETEGYT
TKKLTIPTERFPFTDKKFVVGCVSNGRSTDHCKVTVTVAARATAKNDQTVTCAYGANSNK
EPQTITLSPSQNTFTLVCGTDGDILPSTYQKKYCGSSENNTTSACEEKDYTSIITTYQET
WWQNPEGTDAYILEIPPNDFPAQSANIVVGCTKAVPTASKADGDPSETVSSSVCKVVVTI
EALANSSSATMSGVMASLALGGVAFIGVLAHAM
>KFH05385.1 SAG-related sequence SRS12D [Toxoplasma gondii V
AND]
MSASQPVKANLIHFLSSGCIDVSCCFKFFAAVFSGSPFFSSTFAGCYPAFTMAKVSRMHR
RCGVRSKARKMMAVCLGVVVLLASGAVVAYEMGEGLLRRSLSSETALNSVATCNLGTGDT
IATPGTLTLSESSLSATVSCSGQGNTFVPADLKNVCDGQTTIAEGKHSDTTNCTIGSVAA
GKTVSLQQLLGANHEIQWSTPQNTETTEQGEVRTLKLTVSDLPRTDKSFVVGCQKNADTN
PSCKVTVNVNARPSSVDDKNVVSCAYGQASNEKAVEVEMSEEKNTLTVDCGKDGSMQPPD
YTTQYCAPDGGKLEECTEKYSDILDGFDSKWWTKTDDSTSATLTIPKTNFPTEKKLFLVG
CTPKPTASHKDEKAPIPPKSDVKASSCRVLVTVKAASSATSAFNPQVVAATTGVAALSAL
LAGSV
>XP_003880697.1 srs domain-containing protein [Neospora cani
num Liverpool]
MTTSVAKLWTPTSAGAAQFLLVVSDYLCFVLVVAVLHAEALRQYETLSISVGEERESPCT
VSNGVALCDCSTGTSASATKLTARLSTTVNTIQLTCANADVGMPDPMESDMVCPVDSPDL
SQCKPAGRALQAHKAANQAFPITQLLNGATDASLTWQQQKLKKIYALEIPPNLFPLTDQQ
FGVGCGKKGTNTSCKVKVTVAAKPSASVNQIVSCAYGADSNTGTALQSVTISPTHPSVTL
DCGSAGVVQPEDHTTEHCPAGAPVQHKCDQPYTSIFKDFQPSWWSGDTETSHKATLTVPN
DKFPVDEQKFVVGCKSQETINGGESTAGSTVCSVEVTVLGTSEMSSGGANTPSVLVTAGV
SVVAAYFAFASLEAAFLQDYFSVTS
>XP_003883788.1 SRS domain-containing protein [Neospora cani
num Liverpool]
MAIVRVVRGNRGCGLVVAAVFSVFTLLARFTEGASSGNVVACDVSSTSHTYPVTVTPSLP
SFQITCSPGGATAIPSTFAADQKVCTGTVAAACSTSQALKELIPSAQPGWWVSKNFPTSA
IQFTIPSGSFPRYTPAPFFLGCTKSSKNCLVTVAVQPTPAQYNQATQTVTCAYDQNNTFQ
VTLSPSSNKFTLNCGPNAVVQPTAIQTSYCPQGITATGCSGGAAAQPYKTIFHSYPSSGG
WWTGEVDSDQGGVFTVPSSAFSSTSSSFFVGCAGPERSVAAHNVCSVQVTVAAITPTATE
SSAKGVSVGWREWVLIATAVVGGRSLSV
>XP_003879773.1 srs domain-containing protein [Neospora cani
num Liverpool]
MELLSDRYLISPNSRRTCGSWSSSRVSFVCILLFLVAGIDSWNLQLHGGTIVSGATAASG
RSASRTVESICKNGAENETTCTCAASVPKGHDTSVLTGSRPEESTESSPKTAELVFSGPT
NTLTLICEGGGTPVPNPATSPSDKVCPSSAEIDKCSKNQDSGNNPVSITTLLTGENADLI
KWVDNKATHNKKSYSLTIPGVNLPLTDKAFSVGCLTNGDASTVACKVTVNLVKLSQTTGD
TVECAYGATSNAYRQQVTLSPTQNTLTLVCGKDSAILPTAYQTIFCSAGNAQESCSDTYQ
SIMPQYEEDWWTLSPSESGTYHLEIPSDMFPEEEKRIVVGCKYTQSASGPGQRDEGEVAA
KNRICNVDVTIAASKSAASDAIVPSVMFSGLSSVVMLGVGFPFLN
>XP_008888917.1 SAG-related sequence SRS38D [Hammondia hammo
ndi]
MAYTTMVGEVSVVKATSSTMRFSLAHGVKLSGYPSSFLIRALADEPQVSSVCVPGEGVTK
CTCAAQESDSPKQLEATFSEKLSALELVCGQQSFAPEVSDNKRVCPADTSDFKDCKDNNA
RTKSIDLTTLLTGNTESIKWEAFTRAAQDTTKKLSIPPTNLPYTDQHFAVGCLNNDKTTA
KCKLTVAIEARASVTQDQTVTCAYGKTSNQKHQSIKLSPSQNKFTLVCGKDGEVLPTNYQ
STFCVSKNGVNASAECSGTYTDVIPAYETKWWKHDATQHTFTLEIPEGGFPEKETLIMVG

CQKSKNAADTDNKTVTCAYGKTSNQKHQSIKLSPSQNKFTLVCGKDGEVLPTNYQSTFCV
SKNGVNASAECSGTYTDVIPAYETKWWKHDATQHTFTLEIPEGGFPEKETLIMVGCQKSK
NAADTDNKVREEASSDSPTVCSVDVTLEAVPSSASLSGGIDGVFSWFCSVGAFLTVSHLM
M
>CCA30039.1 SRS domain-containing protein [Neospora caninum
Liverpool]
MRVHFSFLFKIYPKPSIKPRFRDHLRPVPQVVAVIYIVEGVIVSSYNMAHSSRVKCRGSE
TSGTVPLQLSRGLGVRMGLRVVLVFVSTLLLSDFPSFPGCVRALADEPTDPTCVTNEAVT
KCTSTKPDARDKALTATLSEKKNILEVACKNPDLKCAPDGLESNEVCPSTTASLKDCKNE
NRDSTPTPFDINTLLSGSPKDVSWRNCVDATCTTKKLTIPPENFPFVDQQFIVGCVDKSA
SKDTMKVTVTLQARASATDEQTVKCAYGKNSNPTHQAVTLSPEKNSFTLVCGTAGEVLPT
TYETNYCISDNGDDATASCQGSYAEIFPNYEQTWWTTTANTNEYKFTIPEGQFPAEEQKI
TVGCQKTTTEPGKQRTEREEKGSSVCSVDVTIVASASSPATLTSGMAVILLLVGIFYTTL
L
>XP_008889467.1 SAG-related sequence SRS57 [Hammondia hammon
di]
MQLCRHRAAGPANLARQSLPLGRFFAAFGLCVLCAILGSGDRGLFVAAGKPKSNITYFGT
LTKQGPSWYRCSPTRAKEEVVGQVTLNKEHPDMTIECVDEGLGAEFLPLEGATSSYPRVC
HIDAKDKGDCEQNRPFLTDYIPGAKSYWYNIEKVENNGQKSVLYKFTVPWILLPPAKQRY
KVGCRYPNHEYCFVEVTVEPTPPMVEGKRVTCGYSESGPVNLEVDLSKDANFIEIRCGEQ
HYPQPSTYTLQYCSGDSVDPQKCTPAPLSNIFPEYSSSWWKGALNAPNGATLTIPPGGFP
EEDKSFLVGCSLTVDGPPFCNVKVRVAGNPRKWGRYGDGRHPGRGRTRRGTDEEGQAETE
SSAGASSRIASVALAFLLGLLVHAAA
>XP_008888179.1 SAG-related sequence SRS19A [Hammondia hammo
ndi]
MVFFPHCFETDISNVRTMMRTPTVPRLCRGLRSRAHKLMVIYLGAVALLPGEPAIAEKFV
EGFQHRTLEKGPTKTQNGPTFEGGVAKCSLTPSDTGEITKHGATALTLSIQNLTAKLECH
GPMNQAVPLGLGVVCDPSQKGINGKCQFGASTGGIEAMDITLQRLLGTEKFVSWTEDAPY
EHTSRGEKWTLNLTADDLPLEDTPFFVGCQEDPTSDDSAKNPSCVVPVHVEARPSVQADN
VVTCAYGHNSNKKPVEVEITSEKNAVTIECGDLGFVGSRNVATECCATDDPYGEECTGKN
YTDVIPKLEASWVEKDGVTTSVTLTIPVSGFPSKDLKIRLACLPRKASIATDPETTTGQF
GAATSCNVIATVKTTRSASSAVSPLWAVTASGAAAVAGLLFISF
>ESS32458.1 SAG-related sequence SRS40E [Toxoplasma gondii V
EG]
MVRTARIEQRRRGFKSKVRKLLAVCMGGVLLLSSRQAVAIPHEGILRRSLSGSSPATNKI
VPTFNKQVATCTVPSDSAGRNGATATITALTLSKENLSATLKCSGANYVAVPESMKKVCS
ATVNDATLAGCKAAADNAGEKQITLQELLGSSGPVEWTKASKEGDVAKDGEEWTLQLQES
DLPLTDKAFFVGCDNNAVAGKDVQTPSKECKVDFNVKARPSFVAENNVVTCAYGKESNPK
PLKVEMTTEMNTLTIQCGSQGVLNPKSYTATFCDPQDTDMQNCTEKKFEAIFPSFVETWW
AARSKDQSATLIIPEAEFPQSEQQFRLSCIYKEAQSTGTNTAEKVGENSDSAKAAPTSNC
HVIVTVTARSSTSSSGHLVTTVAGAAALTGLLAGSY
>CEL72442.1 TPA: SRS16E [Toxoplasma gondii VEG]
MHSTRVVESSSQYLMFTSPTILILQLGKDKTSTYMQRKGGKYVHFVLKNCITGSSRDVIL
MKVCLSPQAFASISLFVFLCDYWRDGARASDKMVMMMGSMQQRRGGLRSNARQLLAVCMS
GVLFLSSGQAVADNLLEGLLHRTLQQQLEVTARLTVAGSDAKCDFFTPTTQDSPPVSGSL
TLSKGSMTATFECSATQALSISTIPTTIGQNVCDPKKTTNGTKCQFGANDSAGTEVTLKD
LLETDRTVNWKENEQREESKTSQKWSLELHNEDLPLSDKAFVVGCQATSAARGKELGKTA
ACKLTVNVEARASSLAENNVVTCAYGKGSNPNPVEVEMSTEKNTLTINCGSDGSLQPTTY
AEEYCVADSKDVNRCSTTRFVEIFPKFLKSWWVTETQKRNSATLTIPQTDLPEADQQFLV
GCVPKKTAPEDPKKYEESGTETGAPTSCTVLVTVKAASSASHASPTVQILAAASSAAAVT
GFIVSSLGVGW
>KFH03525.1 SAG-related sequence protein SRS19D, partial [To
xoplasma gondii MAS]

MPADPTTQYCEPGDAESGTCKGKNYIDVLPKFEASWVKHDSQKHSVTPTIPEDGFPSKDE
KIRLGCAPRENAKDTVSATSPDQGFIFQTSFLSDSTMARTQMTRRLGGGFRPRASKLMAI
CLGGIALFSTGEAVTDTFLEGLQSRSLQQSSVQTGPTFGDGVATCELKAAAEQAAAASTN
ALILSKGKLTAKLVCSGDGNAAVPESLTTVCKPGRADGTEKCKFGTATVSEGTEAELKSL
LGVNSDVKWEMISTSETDNQGQTWTLTLNEGDLPFKDTPFFVGCKKTAKGKAGASGASP
>XP_003885673.1 SRS domain-containing protein [Neospora cani
num Liverpool]
MSLSFHKTSPRQRKQCTRQIYGVAFPSFVAVLFFVVAESVQIFVEAKGERLGTLKPLGKN
RYSCVQEDGTGPNPGVGSIELNAQNPKLFLLCVGQENEFMPLDGATSQLAVCPMSATTKA
ECDRNSTPLKNFLPRATERWMERTLVLDSSDSKFVLTIPPNGFPPARQQFKLGCRAKGHY
CMLTVTVDPFSAAVDGQRANCAYSLPDPVSLQLSMSEEKNSITILCGKQHFPQPSTYNLN
YCAGSSVDPDQCAASSMTEIFPTFSASWWKGKSNSEQGAVFTIPKGHFPSRATHFLVGCS
EKVDAGSFCNVKVSVAAATVTTTRAPSNGAESWRTDFAFVLSAFALHLSTA
>XP_003884886.1 SRS domain-containing protein [Neospora cani
num Liverpool]
MMPVCAFGGSQVWRGLRCAFGVSIVVGFVFLSCRGETSAKEPVTCPPTTNFLPVWTVEGI
PFFFKCPEGSSLFPTASDGKFKTFCRNSSCTNQAELDTVYTTLQEAAAGDERVVPAPAGA
QTKVYELEVHTEPEFPTTLYFLCKSTQDRATESLATTEARSTSDTMESTCTFQVAVFSRK
PLSDAASEKVCEFGNTMKITLNPKSNKVTFRCGDTGVVWPLNFENAFKGEACTEEVSLAS
LGLGAFRVEGESSDETAKAPAYTLEVSEFPTGTGPVQLCYKYTKPPEQEIEQKSESDVCK
VLINVAPKSETEDGKPGDDRPPTSGDQPSTGDTIGLATHSLIIACGTFLSCAVAIKHAL
>KFH12913.1 SAG-related sequence SRS55A, partial [Toxoplasma
gondii MAS]
MGGFQVFKVFRNAVSATILIGLACWSACAAELENVISCKNGTDFVAASLKQANSKIVLTC
ADDATIFPAIDSKQFCKDAGCTQQDQLTQVGVSAGAASEVEVAGVSQKTDKLTLTLTENR
TNSQTVYFQCIKPKKLREESGPTEQDSATKVACTIQLAIHGSAPASTPTEEKECKYGQSL
HIILRPDSRKVTFRCQEDGVLSPTNFEQTFKGNTCSDEADKVNLTELVPSASLVEGRSSV
SKDDLSTFSIRGYLDAYTLEVSGNLNTDTELCYKCIRTATDRLATEPDISNECKILITVS
GKTTTPDQQGDEPQSGDSDVPTDQPNSDAPDET
>XP_003884898.1 SRS domain-containing protein [Neospora cani
num Liverpool]
MVSKVSGAFCAMKGPAIVVGLLCLSGSLLLSNANSTDATCSESKTLASVALTKESSPIKI
KCPDGSDVFPAVTDKSNNSYCTDSSCAQQAPLDPAFSIQADAAASGAVGGVGVSLQSAKA
NAKSYTLTMHQSSQNSSTLYFQCRTPGESKARSREEFLKKRVEESQATETKCTIQVAAYG
SEAAAAAETEKECNLGGKLSVSLNPKSQSFTFHCAEGSSLQPVNFENAFAGNQCADQKAL
TEFGLVASLLEGKSAASGTPAPPASTGALPAYTFTVSKFPAENTPVLLCYKCEKTAAVSE
HVDETPSECTALIEVSGESATGDGQEDNQDPSELGTSSGAAKHAADTLLIALVTLLFSAL
AVDASV
>CEL77162.1 TPA: SRS domain containing protein, putative [To
xoplasma gondii VEG]
MQMAARSLHVKHSICSWPHCFRWQQQALLSVISGAVVVSSLVAVVCAAAQIPLTCNDNSR
FLTKSLKTKGESIAFQCPEGLTFVPNVAEQESCADSTCNKKRKLLTDFITLEPTQHTLSA
AVQQQSSTAPKVTITLKDPEEESSTLYFLCKKLAEDKRLFKFRAAQGPEEQTTCTLQVSA
WGKKQTAASSEHQCTTSKNVNVTLDSQKQDVTFSCGDMTLSPANFENALEGEACDTENDL
TSLGLPDASLVEGSSGTENKPAYTFNVSSLPAGNPTSICYKCKKRTQGPQQRTEDCTVRI
KVKAEKPPHAQPEGDDSEDGATDGTQTSTEQTSTSGVEIRNVSAIVSVPSLVCLAAGAAS
FS
>KYF47445.1 SAG-related sequence SRS55F [Toxoplasma gondii A
RI]
MEGILLGGFPLSNGLRNAVCVSLLIGILSMSACSAEAPVALTCNENTDFVAGALDEIGSQ
AELKCQEGHSLHPDKASKTFCKDAACTQEIQLSDDRVSWTTATDNEQLLSRKSVDGNVLT
LVEYPEKSQTLYFQCRNKKNEEVQEENRLAQTDKAPKEPCVIQVAAYGTRAAATVEEEHV
CTIGNDKSVTLNSTSNKVTFRCASDADTLSTINFEEALRGDGCDQTVKLADLKLSASLVE

GMSALSATNAVPYLLESPTETDLPTYTFEVAKLPKEQTVLCYKCIKKTEHESIHKSEPAK
DCKFRITVPAAKPNPQGPGEDGQQESHQDPNGQTQQGDDLNPSEPDQKPDGAEKPTGSSA
RKSTTGVAILAVLMMFSALPYMR
>XP_018635616.1 SAG-related sequence SRS55N [Toxoplasma gond
ii ME49]
MEGTALRGFQVSKSLQTAVVVSIFVGLAFLSNGAASSDAAVANQEKTDFAVATLTTEQSE
VTLECPNQSTLYPKTDSKKFCRDAACTQERTFQEAFVSVRWVNGTGQERTEDREKPNSNT
LTLDKYPILSTTLYFQCRNEKNDQEQEQKVVSEEDETPQISRVIQVAVYGARAATTIKKE
KECKDDQTVKLDVTTRAVTFRCASDATMLPINFERVFQGDNCEEQVDLKTLVPSASLVEG
MSATSPGNAVPWSMAELPTFTPAYTFAFLLLPASEKKLCYKCSPPKTLEVTAEAGRQPKE
CMVRITVSGKQTGPDTPTSPENGEEQPILPEQQGGNGHSGQENQEDSHSGSNEPGGQGDN
SNSDKPHQKPGESQTPTGSSSRKTTTSGWMLAIITVFSAFVTISSNSD
>XP_003884896.1 SRS domain-containing protein [Neospora cani
num Liverpool]
MKLSQARYLRRRQQALQSALSVAVFVFSLVSLVSVSAGNPVTCTSGDGFVAAILKSQSNS
FVFQCPEDSTFFPEIGTKKFCTTSSCSTTADLDKSFMTLQPTKPTLSASLDPQINNPSQT
TITLTAPQANSSTLYFLCKKSTGDGLTSTIGAEQESQRETTCTLQVSAWGSNTVAVTPGR
DCGDSMTLSPANFEKAFQGTNCSTEDDLNSLGLPNAALVEGDSATSDRPAYTFSVSSLPQ
PNPVSICYKCTKGSVNLREVSNECTVRIRVPAAQSDQPGEEEHADGETDGTQTSTEQTST
SGAEILDLRAAVSALYFSCLIGVAAAFA
>EPR60006.1 SAG-related sequence protein SRS48K [Toxoplasma
gondii GT1]
MSSHFGPLPRHRRVTFGCSCPRTGFPQAAATYPLLFSGVPFGSMIISLLLSLPRIWAFLCM
SQFSPHSNSVLSLQRDNCLMGHGPPQWRLSRTWPLDTLHGGCRLGGAMSYSAGGSSEFPP
LRLRRVWNCRELLVNWIPLSSARCLVVVFFNRRPAFDFIRSRDNSSPIIFLVVCFGDQRG
FRGSLSDLEASPVNSFSRVEDNKGKAILLFLKMVGRVSGAVCGVKALRSAVRAAVVIGLF
CLSGGVMAEEDTSDVAMCSADKKNQTVVSVSLKNVNDSIQFACPENFVVFPAFQEPSEAA
QFCKDSWCTAQARMGEAFTISHKKPAAQQKDTKKEQQLENLHVYTVTMKQQNLTSSTLYF
QCRPEEERVEARVDTPGGKFDPNTTKCVIQVAAYGSKPAADEATEKECTLNTGLSATLHT
SSTSFTFRCPKGSRLLPVNFDKAYEGLECKKKRSIARMGLGASLLEGKSATSVTTASPSS
TEAATDSSSRAETVAAQAAVEVLPAYTFSVSEFPEKDVQVCYYCVDGSLKKEEINTRAEV
CKALIEVKGVPKPDHAASSGTATFSTNMLVMAGAILISSALVMSISA
>XP_003881691.1 SRS domain-containing protein [Neospora cani
num Liverpool]
MAPTRGLVSSGRQYVRLNACLFVMFLCSFRLPAVAGGTPKCTDSTPVLDLIATDTTPVIF
ECGEKVTNLYPRPAEAGAVMACTSSACTSVISLESLEATLQQQKSATKPYTFTISSTPTQ
ASTVYLQCSSTESPADHSRVDEEDPEQTGKRCTVQIAVWGPPIQGSTQYPTPKLWRRRVG
LDAAVIVPLANVLFPGKCAANQSTLNLEVNSSNKSVTFACGDGNVLSPALFDHVFSADGC
TEESPLADHLSTASLVQHGTTEAGEDAKKPAYTFQVTSLPAEEKTVCYQCKKSSPSGRSN
DPPCTVYIKVAKEETDSGSTTTAPPDASGAETGRWSCLTLITSLISVSVLLAKMI
>XP_008884156.1 SAG-related sequence SRS20C [Hammondia hammo
ndi]
MAPKGGVAAPGRQYASLEACFSLIFLCTLFPPAVAQAAAQQCTTSTPVLSLTATATDSPV
QFTCGENVTYLYPQQPTGGPYTACANSTCTSIVSEDLPKATLSQGNEKQNTFTLTETPAH
ASTVYMKCSSIQPTGNPSEAEQRDQVQTEQQCTVQIAVWGAPVRGSNTYPEPRKCGDGEA
TLNLEVASPNESVTFACGEGGTLSPALFDQVFTTEECSGESPLATHLSGASLVQHGAALG
ETTNSAYTFRVTNLPNNETTFCYKCKAAVVSKADGNDGDKCTVFIKVAKEKADTGSTTVA
PPEESGAERGPWNMALMISLFTVTSALMKTL
>XP_003881503.1 SRS domain-containing protein [Neospora cani
num Liverpool]
METTSCSFGCLWGSASRWAGFVGLRRREGSVCLSGISCFSVALLVLIVAALLSPQVVLGQ
QKEVATPTCNSETGPLTLRIPLAQQNYVSSVTSEPVTFKCGAGLTSLVPGKKSSTGSFTE
FCVDSECTRTASLSSINMTLAETSKAGKLGRLPQETTGTPGTSGRKLSTQDTTYTLTLQE

VPEMGQSVYFMCSSNSSTEREDTSSRNEDTEKRCIVQISVWGQSGPALENDGKLDLHPAC
FQAVTSECGCVRVCVYRRSWQITVCSEDKESVSLQVNESGRSVAFGCGQTRVLSPVLFDK
VFQVSDKGQLTEVSLSSVVSNSTLIEASDDADSFSETPAYELTVKELPNDGNKLLRYQCS
PSESSAKPVNKDSSGTDTSTTKVCNVLIEVTQSGSVAGAAVGGATLAATVLGFVLAFVPS
VLN
>XP_003884631.1 hypothetical protein NCLIV_050290 [Neospora
caninum Liverpool]
MTQNPGVFETATTVASCRRPVFFCRPLRSGLSACFLVVFLCSFRVQFLVAEQNQCTQAKS
VLQLDAKDNTTVTFSCGSGVGHLYPPPPGNSGGKKVCETSACTDAVDVTLTGVKWNPNSN
GGSITTASVPQASTIYVKCTSTPTQQETVTHDARGPGPQGTEEKNCTVQISIWGPPKQGS
ADYPEPSKFASNSLRPQLFTIRLNCKRGCDFAIELPYTVLVFAVFLLVVFCPGTCKDGDT
LNLQITSANQSVTFACGANQNLTPQLFDKVCETDTCDSQALLAHTLSGASMVQHASQKGK
TDTSAYTLTVPNLPDQAKTLYYKCAGTAKQGSKTTECKIAIAVAKAEAGDGSSGPSSETT
TIPPEASGSSAGKWSTTRIASLFSLPLLLTNMM
>XP_008888816.1 SAG-related sequence SRS49B [Hammondia hammo
ndi]
MVVAHSAAACRYSTFRPALLQERRSGHSSVSFVYSSQLLSLSLLALLAASFVQLSVGNEV
SQSISTCDSGGSLVALQITSKTTEVKFKCGTGLQLRENPTGSNKLWGNAACTKEVDASSL
VFTQPSSTPSSSSTLKNHVQGQNAEAAYSLKLKNSTLPQAPFTVYFSCDPKAAPDDGMPG
RGEASVPKKSCLVQVSVFSQQPTPVPDKNKCTDEQVTLAITSNTSSVTFGCSKGATLKPA
LFEHXXXXXXXMSPSCPQSDTLVYRRTAEGVSFDSFSKELSFVSFFKELSPSKKGQNLPA
LLSGTRTGHTSFSVLCSSRCLPLYVFVLFAYNFMQHSQGNSAPQQTVQTCNPGTSPLSLR
LASATNKEVKFKCGEGLELLKNPNKDGKLCGNAACTKEIDASAFTFEPSSQATKQNSTPD
TEYSLGLKDSLPTTPLTVYFSCDPKSVAGGGGPVGVEASDSNKSCLVQVSVFSQQPTPVP
DKNKCTDEQVTLAITSNTKSVTFGCNKGATLKPALFERVFIAEAIQKGDGTSAVKEKEVV
LQDLVPNSSLVENAATESATVNASTESDTVGYTLSCPDLPSSAQNFFYKCVSPSPASNNA
RDGAQKECKVLISVEKKPEPQATATPAPSRGEKRVALTSSFMIVSILLVKMATETLF
>XP_003881502.1 putative protein phosphatase 2C [Neospora ca
ninum Liverpool]
MKLEAATGSDGRKQDTQNSYTLTVEKVPQMGQSVYFICSSKSPTAAGEEVSRSEDAEKRC
IVQISVWGQMIPALENDAVCSEDKESVSLQVNESGRSVAFGCGQTRVLSPVLFDKVFQAS
DKGQLTEVSLSSVVSNSTLIEASDDADSFSETPAYELTVKELPNDGNKLLRYQCSPSESS
TQTVNKGSSGTDTSTTKVCNVLIEAHGQPSCATGAAPHSSADGERMKRRRGETPSLRVRM
EARFMQGRRPKQEDRHVLVSDISTLIEGQDKAAIRALDCRPAALVALFDGHCGATCSEFC
ATQLPSRVVSYLVKSLPKSKRQKLSQHSAATVAEGDALSAAASPSSLSSSPASSPASALL
VKLPSSLPSLVACPFPLPSEPVEELFLTLGPRIISAFKHVDREFLSKFRTLKVGGCTAVV
CLLLGNFVVVASVGDSRAVAGVRRRKRPEHDAQAGSPCSLKGEVERQPGEAQEERDGEES
NGALSQSPSSSTNADSQGPQVKLGGSDEKTQVDHGEAGEEFVWEAVRVSRDHKPNLPEER
ERIEANGGRVIEVGGVARVAPKGFEVVSRGFGDKELKEENLISATPDVFGFHATEEVRLL
IIACDGVWDVMTDQEAVEIAACHLDDPKEAASQVVKRAFERGSQDNLTAIAVVFDH
>XP_003884879.1 SRS domain-containing protein [Neospora cani
num Liverpool]
MSPVCLSAETVRIIAAIAVALICVAAYPSDGAKAALALVEQGQTSVTTVKTCKAGTHSIE
LSLEPNMHSVSFKCGDTNDGKLEPSTTEYFTGDDVSQQKQNLTTAFADATLDAEGENGNK
AYKLSIGRETRTAEKDLYYTCTFTNTNAVLGERRDGENPQETKCKVKITVKPFPQSQQGT
NDEQGDQPNDEQGDQPNDEQQAGPIECTDADTTKETSASAESPLSFKCGAGMSLRPTNLT
DVFDDQDGKCAAEVALQTLVDATLTKTETEATQKGQPVYQLAVKTAPPEDTALCYKCVPS
SSSDTETEIQSEGESSAKECLLKISVKGSASSAFSPTWGPAHGAAAFFVAAQLLRGMVDA
>KFG39207.1 SAG-related sequence SRS23 [Toxoplasma gondii p8
9]
MQATGASVSLAVVLAFLCVLPVFSETSQAPEGTDTTPSCTEKSTLTLILQGNDREASFRC
PATWTLQPADLTQAYENADSSNTVALDTLVSGATLIHDSENSKYTLTLPSERTDKTFQYK
CQKPSQDASGKNTVNSASQSCKIVVKVFASEIKSAIECKEGEINVATVKVTGNALSLKCK

NLTLDPPDVQNVYDDEDGKCESKVALTSLVDGSLAAVAAEQQDNGEYALSISELPTDAKH
LCYKCVAKQSKTQAGDASKECMLKLTVASGAAAFATASISVGILASLASFLSAN
>XP_002372001.1 SAG-related sequence SRS49D [Toxoplasma gond
ii ME49]
MAAAHSAAACRYSTFWPCLLRERQSGTSSVSFVYPSQFLSLSLLVILTGSFAQQSAGNQA
NSQSVTCESNASPLVLRITSKTNEVKFKCGTDLQLRENPAGSNKFWGNAACTKEVDASSV
TFTSSPSPAKVAGNKGTEYSLALKNSSLPLSPFTVYFSCDPPSTTGVGETGKAKVPASAT
TCIVQVSVFSQTAVTVPETNKCKNGQVTVAVTSKSKSVTFGCSEGATLKPALLDHVFIEK
ATEKSGGASTGREEEVVLQDLVPNSSLVENAANTGNDTVGYTLSCPDLPSSPQNIFYKCV
SPASAREQVGTQTECKVLINIEEKPEAETPATPEPSRGEQGVVLGSAFMIAFISCFALVA
GNMF
>KYF49211.1 SAG-related sequence SRS56 [Toxoplasma gondii AR
I]
MERDIDAPHRVSVVENITSTSFHSQDRAPIRLRRRWEDTAMATGASRQDPFVSDKRRIAG
PFSWAFSANLVPFFLFALLAVYLRSELPAAAMGKSNVCTYKSIPVILRIKKPGEAVTFKC
GEPQPHVLPAKVDDEYKLYCQDSLCKATAPLSDVTITTTAGQERWDTEYKVTAGRTLPER
PYTMYFVCTSMESDFEERGNLEVSRSDSYTIYQPKMCKVQVSVWGATRPQSFDKKYECGD
NVSQITHTIKERDSSVTFRCGPGRFLSPGILDVYVEPPTYSNLVSLGAMLPFADLHEHAS
STRDDIPAYTLAVRDLQKTRERKLGYHCVPDSVDTQICSVVINVINTDYDPLTGVVGSEA
PSGAASSLSLVLVLMAFLPSGCS
>XP_003884629.1 SAG2 related antigen SAG2D, related [Neospor
a caninum Liverpool]
MSLSDISLTPQSNGVSITTTDVQQANTIYFQCTDTATETHLSRLDSKPPAGQSSSKKVCT
VQISIWGPPKQGSGYPVPSKFANNSVRPHLFTSHLNCTLWNTCCQFFVYDFVLHPLLRTL
AQTEGAKRKECGEKLDFSRLKRSLLQPPLPGVQSFCTSQSTVNPPAIYCVFLSFLGTCNS
DNGPLRLEITSASQSVTFACGNDQNLTPKLFDQICQSDGCESTAPLADTLAGASLVQHAS
QEGNPNTPAYTLSVPQLPDESQTLFYKCATGTDKDSDPKECNVVITVAKAADGDGSSGPS
SDTATIPPETSGTGAERWSKSLAFFVFSVALLLTKMI
>XP_008883416.1 SAG-related sequence SRS52C [Hammondia hammo
ndi]
MTIYFGCSKPGGTAPPSSNDVQGTRGPSTPASKPTDSTAQCVVTVTVPADPAANTCTLTK
KTMNLEVTSKSKSVSFKCDTDIATLTPAVASGMIYDKLCKEEFKLSEKLPTAKLAETTSG
YTFSLEELPENAAAFCYKCLASAAGNQKGGSGQQPSACTVKINVSAAVPDSDISASATTG
SAAALVFGLTGSLIFTIGGF
>XP_003883768.1 SRS domain-containing protein [Neospora cani
num Liverpool]
MKRLRGVLRLFIRFLAAHVLAACVVRPVLRCACSYDTATCHRGGTTIFVPISAATRTAEF
RCGFGVPRLEPSVESQHVFSSRECNTTVSLESQLPGASLVAAGRDGGPSYILKLKEWPTA
EAPEAAYYQCAPLGTDGPTCKVMVGINRPTSQSSKKSNQPTAQLHQAGKAIPTCSESGTS
VSVHVSSRTRRGYFRCGTELPLLDPPVSEELAYTTRSCRAAAPLAAQVPTMACAYNQPQN
LYILTLKQLPAEEHKTVYYKCVDPGSSAACKVIVHVPPSAPDPQSPRAHLPSSTGVCTES
GTTIRVDVSSRTKRAQFGCGGGMFLLDPPFLSGNLYTTRSCSKPMPLRTQVSGYLLMSPS
GGSFYTLALKSLPVDRPRTLYYRCMSPGRGESCKIAINVPPDPTANQNVVQYRGVPVCSD
NGSTIIAPVSPQTKTAQFKCGPAFFELYPPVASGKAYPSRACVTPAPLGDIVNGSLSESA
GGHNLYTFRVDDLPARESKDVYFKCMAPDRSGSCKVHIKIPRRYSSAKPLEPPNIHVCYP
GPDASLHIRTPAYSSFYVRCAAGLTHFPSEETEVYDNRDGACSTSVSLDRLVKGAELIRV
PQVRSTGSTYLFSVERLPEEQNKLCYLCAPNSNLRTEACRILITVPSRNHSHGRALEPTT
TPSSQASLKRIDSVSIMALLSMSVFTF
>XP_003880400.1 putative SRS14 [Neospora caninum Liverpool]
MHIALCDTECPVGRAVSRPSSNGLKSRGRSAVSLASPRYVSLPLLSLLVAVLVQPQLSVG
KEDSTEYPMCTSAEKPLTLRITEAVTPTDSKNGFVTFRCAEQASLLPAKPAGTVYTQFCK
DSDCTKAASLTKNQLKLEEVTVPEQEPEPEPEPEPEPEPEPEPESETKVEEEKEPQPAVVST
DAVQRVGKMYKLSATELPEKPFTAYFICVTPSEPPLHTSKRSGLAAKSIAGDKCKVQVSI

YGKTKLVLSSKGKVDLQNGSSSSYRVHTRKEPGRPQMSCSIPVCTRRRQTVQG
>AAQ63830.1 SAG5C [Toxoplasma gondii]
MERTTAISNKFRAAAGLLVAVLFMSSPSGVRGRAAAGNKITPNCMASDNITACVCKESET
ASLRKDGQSDSATLSESINSVTIQCPTTGDFKFVPSTLTEVCTAEETNITLKTCEGDNNN
KNKSPITAFLVDVDGNNAPKWTVAPPDSKTHSLTLPAENFPRVDKSFFAGCLKTSSSRQR
NADVECLVKVDVKARTSAVRDGVLICAYGDKSNTSVPEVTLNAENNSLTIQCGEEGEMQP
DPKSLTAYHCTDTNIENCKTVVNLTEVMPSFAKSWWTQDDKNGKAPKLVIPEGGFPAQEE
TIVLGCNVRRKVSSKGQKEEPNITATLPTCRVKVTLTAQPAASDAPTFSCLGLLAVVFTP
FVSFGAY
>XP_003883724.1 SRS domain-containing protein [Neospora cani
num Liverpool]
MERKSFFSSLARPAVGVLLIFFAACLCRLSMAQINRPTSIPIAPTCDVTENGTACVCEES
VKGRAQESASSATLSESHNTISVQCPEQYGFVPSDGTKVCSVDTDTNATDLGTCNTHNEK
IEAFLNPVPASPPQWSASDTTTTPHSLSLPQSHFPLTDKKFFVGCLEQEDRLNKLLQGTP
PKKSCVVTVQVEAKKSSLHENVLTCGYGANSNTEKTPVATLTSDNNALTIVCGSEGQLQP
SGKPTTAFLCDISTEECSTAVKLTDVFPNFTEAWITVEGETGSNKLVIPEDGFPEEDKMI
MLGCSLKTVSGDDKKKNENTTVQEEPTCKVKVVIPAGGRSSAAASGPSSPMYAVAVGLLS
LVVSSTFLTSH
>XP_003883689.1 SRS domain-containing protein [Neospora cani
num Liverpool]
MKVTVTLEARATATEDHIVKCAYGKNSNPAHQTVTLSPEKNSFTLVCGDQGDVLPTNHKE
EYCVSDTGSDEVESCTGAYEGIFANYEATWWAATTDIKEYKFTIPDGQFPAEAQMITVGC
QNTAVEPGTQPKEVGEKDSSVCSVDVRIVASAASSAALTSGMAVTLLSLAAFFSTPLLKI
GVRMGLRVILVFVSAFLLLDSLPFSGCVRAVADEPTDPACVIAGAVTKCTCTSKEVTNKD
LTATLSQEKNVLEIGCNKRELKCVPAELNGKLVCPSTTAELKSCKSGETRTTVQPFDINT
LLSGTSPKVSWQSCEGNGDGTCTTKQLTIPPANFPFVDHKFMVGCVEAETTDTMKVTVTL
EARPSETEDHIVRCAYGKNSNNAHQTVTLTPEKNSFTLVCGNEGEVLPTTYETNYCLTQE
GNDAGAQCTGAYGGIFPNYEETWWTTTPHTNEYKFTIPEGQFPAEEQKITVGCQKKATAE
PGKQRTESEEKDSSVCSVDVTIVASAASSAALTSGLAVTLVSIAGMVYATVF
>XP_002364752.1 SAG-related sequence SRS54 [Toxoplasma gondi
i ME49]
MASVARCVVPVPSDLVPRREMQPTQGWRVKRAFPLISGVGLTLVFIQIASGLFNAAPVIV
IAGAEGQTSTTLACAEDAQHSKISCTCTPGTVEKRTPATAVTHEVTVSGNKSELQLECKT
NFNFVPKDDTGKKVCPPGKEICDCKNESHCVPIDPFFTGTTTTLAWKRVGTSKRENSDTK
SLTIPAENFPYVDGKFLVGCTGSSESPAPCALTVNIEARASVTNDHTVTCAYGNKSNKEH
QAITIKPSQNSFTLVCGDKGEILPKNYVTTYCATQAGQHASDDCKGDYKSILPAYTEKWW
KENPTGSFVFEIPPGDFPEVPVNLTLSCQQKTTRQSSIRDTDSNASDKPSVCTVDVTIEN
GPNSSASATGFLSLTMGSFAAVTALTYTA
>AKQ22481.1 P48/45, partial [Plasmodium chabaudi chabaudi]
SKRRVIFFLYSWCVCGLFLFLIMLSFFGKSRFFLFSFYCLFYFVLVIKSSLGQNEYVSPD
ELNIKTSGFLGYKCDFSTEGIHNLEPDIVERRSVICSINSYFIYDKIKLIIPKQDDPKSK
FKLLPENCFAKVYSDIEGHQEIPIEKTGLVEYTLEENDTNKDYDERIIQISPFNNKDVEF
YCICDNTEQVISHIDGRSALVHVHVLKYPHNIISVNLTDNMYPYLPGTYNKNSFVDYKLE
VGLKEGELLVLACKQIDNKCFQKNDESKNGDLYKTNKIIYHKDFTLFKAPIYVKSNDSTA
ECKCKINETDIYTITVKPDYDEKVIHGCNFSNDLSIRTFTNNMNLLKYNENTDINCNVEI
VQPFYDHLIGISCPGTIIPDCFFQIYKPPTNELKSSEITYLDSQLNIGNIEYYEDIHGNN
EVRIFSIVGAIPQSASFTCMCKMDKITGFMNVKIGSACYAFLSKLLIIFIPLLFMWL
>ALQ43976.1 6-cysteine protein, partial [Plasmodium falcipar
um]
MCMGRMISIINIILFYFFLWVKKSISELLSSTQYVCDFYFNPLTNLKPTVVGSSEIYEEV
GCTINNPTLGDHIVLICPKKNNGDFSNIEIVPTNCFESHLYSAYKNDSSAYHLEKLDIDK
KYAINSSFSDFYLKILVIPNEYKSHKTIYCRCDNSKTEKNIPGQDKILKGKLGLVKIILR
NQYNNIIELEKTKHIIHNKKDTYKYDIKLKESDILMFYMKEETIVESGNCEEILNIKINL

LSNNNVVLKMPSIFINNINCMFSSQDQNNEKNYINLKADKTKHIDGCDFTKPKGKGIYKN
GFIINDIPNEEERICTVHLWNKKNQTIAGIKCPYKLIPPYCFKHVLYEKEIDSQKTYKTF
LLSDVLDTPNIEYYGNNKEGMYMLALPTKPEKTNKIRCICEQGGKKAVMELHIASTSTKY
ISMFLLFFLIVIFYMYVSI
