# Supplementary material for: The first identification of complete Eph-ephrin signalling in ctenophores and sponges reveals a role for neofunctionalization in the emergence of signalling domains
Source: BMC Evol Biol. 2019 Apr 25;19:96. doi: 10.1186/s12862-019-1418-z (PMC6485061; doi:10.1186/s12862-019-1418-z)
Supplement: Supplementary file 3 — Raw tree files of all phylogenetic trees reported in the manuscript. (PDF 271 kb) [file 12862_2019_1418_MOESM3_ESM.pdf]

Fig_4A(Fig_S4)_IQtree_tree_file

(EPHA1_HUMAN:0.6103603180,(EPHA2_HUMAN:0.1307875224,
(Drer_11069.6:0.1006208556,Drer_44917.5:0.0519159488)100:0.071122417
5)100:0.0936959829,((((((EPHA3_HUMAN:
0.0149668294,Drer_21706.11:0.0292313565)100:0.0545963925,
(EPHA5_HUMAN:
0.0520073439,Drer_75583.4:0.0667172154)100:0.0320516115)98:0.0325948
110,(((EPHA7_HUMAN:0.0262309778,
(Drer_93563.5:0.0000023175,Drer_131364.1:0.0000023175)100:0.01899726
71)100:0.0657878356,(EPHA10_HUMAN:
0.4619727891,Drer_141023.1:0.2873100714)100:0.2517227868)76:0.033493
0013,((Drer_41799.6:0.1020090587,EPHA8_HUMAN:
0.1555391066)100:0.0808397553,(EPHA6_HUMAN:
0.0282975971,Drer_121462.2:0.0766050611)100:0.0912081273)92:0.017650
0478)78:0.0122179693)72:0.0085453618,(((EPHA4_HUMAN:0.0122974197,
(Drer_123962.1:0.0270305405,Drer_3161.9:0.0667126506)100:0.023917453
9)100:0.0449958468,Pmar_10282.1:0.2528185197)58:0.0117759585,
(Drer_30606.8:0.0160668325,Drer_96552.3:0.0530005785)100:0.068354899
1)89:0.0367261939)53:0.0090995604,(((((EPHB1_HUMAN:0.0178865834,
(Drer_111570.2:0.0000023175,Drer_121980.1:0.0000023175)100:0.0223460
754)100:0.0386810812,(Drer_40208.7:0.1082284151,(EPHB4_HUMAN:
0.0547492802,
(Drer_88414.3:0.0370230697,Drer_135357.1:0.0349413408)100:0.04310014
31)100:0.0544193953)58:0.0405785027)46:0.0162490158,(EPHB3_HUMAN:
0.0302354279,Drer_140419.1:0.0139535476)49:0.0328497648)88:0.0196565
728,((((EPHB2_HUMAN:
0.0128769652,Drer_112928.2:0.0250625085)74:0.0059108586,Drer_43755.7
:
0.0111701819)82:0.0165025857,Pmar_5131.1:0.0205449401)98:0.035670843
5,
(Pmar_7951.1:0.0520616990,Pmar_9675.1:0.1807871669)82:0.0311084436)5
3:0.0000028000,(Drer_113830.2:0.3309552629,EPHB6_HUMAN:
0.5483517071)99:0.2238215334)61:0.0081578172)87:0.0657387636,
(((CiEphb:0.0841426517,CiEphd:0.0876042788)100:0.0629685616,CiEpha:
0.4156265493)100:0.0873128801,
((BfEph1:0.0360496527,BfEph2:0.2031223902)100:0.2136812451,
((((Ctel_198909:0.1685905834,Tpol_63971:0.0960569476)84:0.0011713291
,
(Hpsi_102387:0.0982639938,Lgig_105485:0.0677001068)92:0.0065605351)9
8:0.0447909318,
((Dpul_79285:0.0997684289,Lith_3827:0.0000025847)58:0.0000026863,Lit
h_18666:0.0194456887)100:0.1536136493)97:0.0662037572,
(Spur_027145:0.2529907730,
((((((((Adig_18762:0.1972917340,Pcar_328883:0.1602845145)100:0.06855
14924,
(Apal_103658:0.1293796539,Nvec_40994:0.1093064326)100:0.0657664444)9
8:0.0934976875,((Ever_12042:0.4038044895,
(Ever_2330:0.1688794143,Ever_2431:0.3690637961)98:0.0356247615)93:0.
0846542481,Ever_14808:0.3678076316)96:0.1561889506)32:0.0072909476,
(Ever_3805:0.3411519389,Ever_20101:0.5194391524)58:0.1102537543)42:0
.0297511934,Ever_3065:0.6024065334)54:0.0327162407,
(((((Adig_04039:0.0396856996,Adig_24585:0.0143322165)79:0.0000025201

,Adig_12482:0.0757027115)97:0.0121958416,
(Adig_12429:0.0226734126,Adig_15481:0.0517844130)96:0.0111819527)74:
0.0010435935,Adig_03916:0.4774643997)100:0.3109292900,
(((((Apal_306157:0.0136686944,
(Apal_306140:0.0341661927,Apal_271591:0.2284043843)98:0.0207846347)9
8:0.0683594845,
((Apal_306159:0.0999476889,Btue_4512:0.2363245821)91:0.0153174355,Hd
ig_16446:0.1717101069)79:0.0162069817)82:0.0483282008,Apal_455194:0.
3025765415)99:0.0590498981,Nvec_33736:0.1972451167)99:0.0601427642,
(Pcar_152315:0.0000026066,Pcar_152322:0.0842567668)100:0.3021672801)
96:0.0690505635)91:0.1071870465)87:0.0690594068,
((Scoa_1400:0.6932708029,
(Ocar_5890:0.5225606902,Sros_Peph_EGD75115:1.0208728766)84:0.0406355
591)86:0.1082476961,(((Cnul_9016:0.4390546884,
(Ifas_9729:0.3471738999,
((((Cpro_79320.39:0.0608758109,Lapi_154083:0.0793706482)100:0.032196
1639,Kvar_222799:0.1298133502)100:0.1656998015,Psub_10078:0.27840630
29)100:0.1565954897,
((Niph_54284:0.1367839614,Pfic_10202:0.1673128205)100:0.0845364717,
(Xtes_54061:0.1717469005,Aque_41463:0.2192308704)68:0.0080196573)100
:0.4407515180)100:0.1605650072)92:0.1191230447)96:0.0818599070,
((((Avas_63694:0.3844500446,
(Rfib_55118:0.0958623591,Snux_99202:0.0385743108)100:0.1509390231)10
0:0.2395500115,((Avas_85324:0.2264587312,
(Rfib_49817:0.1238409280,Snux_109225:0.1117026676)100:0.1389517978)9
6:0.0850424297,Avas_93988:0.1904872114)100:0.1228473763)97:0.0768392
327,
(Avas_82832:0.2354475629,Rfib_47064:0.2743484443)100:0.2791329797)99
:0.0535398559,(Avas_85009:0.4616927591,
(Rfib_47059:0.1226695755,Snux_6723:0.0985214759)100:0.2282772327)100
:0.3408414004)100:0.3798392475)80:0.1308710174,
((((Baby_41769:0.1891571315,Patl_251869:0.6803136693)95:0.0619982582
,Mlei_35913a-PA:
0.1553318202)99:0.3171396037,Vmul_37755:0.3290350176)100:0.434467341
6,
((((Sros_Peph_EGD72856:0.5482117623,Mbre_Peph_25247:0.7672101381)100
:0.2822421188,
(Aspe_Peph_146412:0.9255890382,Aspe_Peph_158325:1.0809019872)91:0.18
69525394)91:0.1099491893,Mbre_Peph_26435:1.1774052080)98:0.200003378
0,((Mvib_Peph_25039:1.0548986158,
(Cowc_Peph_01676T0:0.7493616136,Cowc_Peph_09852T0:0.5371981116)100:0
.5788958596)100:0.1516017111,
(Apar_Peph_165057:1.1576167508,Sarc_Peph_10364T0:1.0295884240)100:0.
5881019563)95:0.3322321466)92:0.1457676308)80:0.1664451566)56:0.0702
938561)65:0.1070248933)41:0.0128124295,
((((((Atet_45096:0.0380648519,Clat_102964:0.0240813476)100:0.0706431
361,
((Aele_129846:0.0000021491,Nbij_1526:0.1234061882)100:0.0250215533,N
bij_150435:0.0789302958)100:0.0327600854)100:0.1446684741,
(Aele_55285:0.0902205362,Clat_76838:0.0667016895)100:0.1128850262)92
:0.0431451048,Hvul_18252:0.3341171052)84:0.0608685924,
(((Hvul_13823:0.1358953134,Hvul_37741:0.6966343443)98:0.0740157957,
((Hvul_31223:0.1888336885,Hvul_13820:0.1937924334)91:0.0087625343,Hv
ul_37681:0.0558404855)97:0.0591850428)95:0.0647534114,

```
((Clat_108627:0.0785311957,Nbij_35443:0.0970092552)90:0.0160612019,P
phy_15204:0.0446392639)100:0.3363252194)91:0.0628088537)100:0.188206
4707,Tadh_12344:0.9920930180)60:0.1279848946)100:0.2020661595)99:0.0
700259493)99:0.0747315172)100:0.0781669538)73:0.0318532290)79:0.0213
165249,Pmar_2234.1:0.2826392146)75:0.0141018060)67:0.0262359957,
((Pmar_7260.1:0.0158612756,Pmar_10202.1:0.0260454893)100:0.026814513
6,Pmar_9793.1:0.0986054196)100:0.1654108725)80:0.0410812260,(CiEphe:
0.5910615501,CiEphf:0.8023471928)67:0.1211499866)97:0.0406755896);
```

Fig4A(Fig_S4)_MrBayes_tree_file

```
#NEXUS
[ID: 2837186988]
begin taxa;
	dimensions ntax=142;
	taxlabels
		EPHA1_HUMAN
		EPHA2_HUMAN
		Drer_11069_6
		Drer_44917_5
		EPHA3_HUMAN
		Drer_21706_11
		EPHA5_HUMAN
		Drer_75583_4
		EPHA4_HUMAN
		Drer_123962_1
		Drer_3161_9
		Drer_30606_8
		Drer_96552_3
		EPHA7_HUMAN
		Drer_93563_5
		Drer_131364_1
		Drer_41799_6
		EPHA8_HUMAN
		EPHB1_HUMAN
		Drer_111570_2
		Drer_121980_1
		EPHB2_HUMAN
		Drer_43755_7
		Drer_112928_2
		Pmar_5131_1
		EPHB3_HUMAN
		Drer_140419_1
		Pmar_7951_1
		Drer_40208_7
		EPHB4_HUMAN
		Drer_88414_3
		Drer_135357_1
		EPHA6_HUMAN
		Drer_121462_2
		Pmar_7260_1
		Pmar_10202_1
		Pmar_9793_1
		CiEphb
```

CiEphd
Pmar_2234_1
BfEph1
BfEph2
Ctel_198909
Dpul_79285
Lith_18666
Hpsi_102387
Lgig_105485
Lith_3827
Tpol_63971
Spur_027145
Pmar_10282_1
Adig_18762
Apal_103658
Nvec_40994
Pcar_328883
Ever_3805
Adig_04039
Adig_12429
Adig_15481
Adig_24585
Adig_12482
Apal_306157
Apal_306140
Apal_306159
Hdig_16446
Nvec_33736
Apal_455194
Pcar_152315
Pcar_152322
Btue_4512
Ever_12042
Ever_14808
Ever_2330
Ever_2431
Adig_03916
CiEpha
Atet_45096
Clat_102964
Aele_129846
Nbij_1526
Nbij_150435
Aele_55285
Clat_76838
Hvul_13823
Hvul_31223
Hvul_37681
Clat_108627
Nbij_35443
Pphy_15204
Hvul_13820
Hvul_18252
Ever_3065

```
			Drer_113830_2
			Scoa_1400
			CiEphe
			Ocar_5890
			Cnul_9016
			Ifas_9729
			Cpro_79320_39
			Lapi_154083
			Kvar_222799
			Psub_10078
			Niph_54284
			Pfic_10202
			Xtes_54061
			Aque_41463
			Hvul_37741
			EPHA10_HUMAN
			Drer_141023_1
			Pmar_9675_1
			EPHB6_HUMAN
			Avas_63694
			Rfib_55118
			Snux_99202
			Avas_85324
			Rfib_49817
			Snux_109225
			Avas_93988
			Avas_82832
			Rfib_47064
			Avas_85009
			Rfib_47059
			Snux_6723
			Apal_271591
			CiEphf
			Sros_Peph_EGD75115
			Tadh_12344
			Ever_20101
			Baby_41769
			Mlei_35913a_PA
			Vmul_37755
			Patl_251869
			Sros_Peph_EGD72856
			Mbre_Peph_25247
			Mvib_Peph_25039
			Mbre_Peph_26435
			Aspe_Peph_146412
			Cowc_Peph_01676T0
			Cowc_Peph_09852T0
			Aspe_Peph_158325
			Apar_Peph_165057
			Sarc_Peph_10364T0
			;
end;
begin trees;
	translate
```

1	EPHA1_HUMAN,
2	EPHA2_HUMAN,
3	Drer_11069_6,
4	Drer_44917_5,
5	EPHA3_HUMAN,
6	Drer_21706_11,
7	EPHA5_HUMAN,
8	Drer_75583_4,
9	EPHA4_HUMAN,
10	Drer_123962_1,
11	Drer_3161_9,
12	Drer_30606_8,
13	Drer_96552_3,
14	EPHA7_HUMAN,
15	Drer_93563_5,
16	Drer_131364_1,
17	Drer_41799_6,
18	EPHA8_HUMAN,
19	EPHB1_HUMAN,
20	Drer_111570_2,
21	Drer_121980_1,
22	EPHB2_HUMAN,
23	Drer_43755_7,
24	Drer_112928_2,
25	Pmar_5131_1,
26	EPHB3_HUMAN,
27	Drer_140419_1,
28	Pmar_7951_1,
29	Drer_40208_7,
30	EPHB4_HUMAN,
31	Drer_88414_3,
32	Drer_135357_1,
33	EPHA6_HUMAN,
34	Drer_121462_2,
35	Pmar_7260_1,
36	Pmar_10202_1,
37	Pmar_9793_1,
38	CiEphb,
39	CiEphd,
40	Pmar_2234_1,
41	BfEph1,
42	BfEph2,
43	Ctel_198909,
44	Dpul_79285,
45	Lith_18666,
46	Hpsi_102387,
47	Lgig_105485,
48	Lith_3827,
49	Tpol_63971,
50	Spur_027145,
51	Pmar_10282_1,
52	Adig_18762,
53	Apal_103658,
54	Nvec_40994,

55 Pcar_328883,
56 Ever_3805,
57 Adig_04039,
58 Adig_12429,
59 Adig_15481,
60 Adig_24585,
61 Adig_12482,
62 Apal_306157,
63 Apal_306140,
64 Apal_306159,
65 Hdig_16446,
66 Nvec_33736,
67 Apal_455194,
68 Pcar_152315,
69 Pcar_152322,
70 Btue_4512,
71 Ever_12042,
72 Ever_14808,
73 Ever_2330,
74 Ever_2431,
75 Adig_03916,
76 CiEpha,
77 Atet_45096,
78 Clat_102964,
79 Aele_129846,
80 Nbij_1526,
81 Nbij_150435,
82 Aele_55285,
83 Clat_76838,
84 Hvul_13823,
85 Hvul_31223,
86 Hvul_37681,
87 Clat_108627,
88 Nbij_35443,
89 Pphy_15204,
90 Hvul_13820,
91 Hvul_18252,
92 Ever_3065,
93 Drer_113830_2,
94 Scoa_1400,
95 CiEphe,
96 Ocar_5890,
97 Cnul_9016,
98 Ifas_9729,
99 Cpro_79320_39,
100 Lapi_154083,
101 Kvar_222799,
102 Psub_10078,
103 Niph_54284,
104 Pfic_10202,
105 Xtes_54061,
106 Aque_41463,
107 Hvul_37741,
108 EPHA10_HUMAN,

```
                109     Drer_141023_1,
                110     Pmar_9675_1,
                111     EPHB6_HUMAN,
                112     Avas_63694,
                113     Rfib_55118,
                114     Snux_99202,
                115     Avas_85324,
                116     Rfib_49817,
                117     Snux_109225,
                118     Avas_93988,
                119     Avas_82832,
                120     Rfib_47064,
                121     Avas_85009,
                122     Rfib_47059,
                123     Snux_6723,
                124     Apal_271591,
                125     CiEphf,
                126     Sros_Peph_EGD75115,
                127     Tadh_12344,
                128     Ever_20101,
                129     Baby_41769,
                130     Mlei_35913a_PA,
                131     Vmul_37755,
                132     Patl_251869,
                133     Sros_Peph_EGD72856,
                134     Mbre_Peph_25247,
                135     Mvib_Peph_25039,
                136     Mbre_Peph_26435,
                137     Aspe_Peph_146412,
                138     Cowc_Peph_01676T0,
                139     Cowc_Peph_09852T0,
                140     Aspe_Peph_158325,
                141     Apar_Peph_165057,
                142     Sarc_Peph_10364T0
                ;
   tree con_50_majrule = [&U]
```


```
(1[&prob=1.00000000e+00,prob_stddev=0.00000000e+00,prob_range={1.000
00000e+00,1.00000000e+00},prob(percent)="100",prob+-sd="100+-0"]:
3.534655e-01[&length_mean=3.52877327e-01,length_median=3.53465500e-0
1,length_95%HPD={2.51977200e-01,4.46042800e-01}],
(95[&prob=1.00000000e+00,prob_stddev=0.00000000e+00,prob_range={1.00
000000e+00,1.00000000e+00},prob(percent)="100",prob+-sd="100+-0"]:
3.517241e-01[&length_mean=3.56082678e-01,length_median=3.51724100e-0
1,length_95%HPD={2.59655200e-01,4.56509300e-01}],
125[&prob=1.00000000e+00,prob_stddev=0.00000000e+00,prob_range={1.00
000000e+00,1.00000000e+00},prob(percent)="100",prob+-sd="100+-0"]:
4.671574e-01[&length_mean=4.67563129e-01,length_median=4.67157400e-0
1,length_95%HPD={3.51405900e-01,5.95157900e-01}])
[&prob=7.64966002e-01,prob_stddev=1.98892441e-01,prob_range={6.24327
808e-01,9.05604195e-01},prob(percent)="76",prob+-sd="76+-20"]:
9.522481e-02[&length_mean=9.53273416e-02,length_median=9.52248100e-0
2,length_95%HPD={1.33583100e-02,1.75363800e-01}],
((2[&prob=1.00000000e+00,prob_stddev=0.00000000e+00,prob_range={1.00
000000e+00,1.00000000e+00},prob(percent)="100",prob+-sd="100+-0"]:
```

9.967341e-02[&length_mean=1.00492511e-01,length_median=9.96734100e-0
2,length_95%HPD={5.66170600e-02,1.43601200e-01}],
(3[&prob=1.00000000e+00,prob_stddev=0.00000000e+00,prob_range={1.000
00000e+00,1.00000000e+00},prob(percent)="100",prob+-sd="100+-0"]:
8.256438e-02[&length_mean=8.43663511e-02,length_median=8.25643800e-0
2,length_95%HPD={4.80734400e-02,1.24506700e-01}],
4[&prob=1.00000000e+00,prob_stddev=0.00000000e+00,prob_range={1.0000
0000e+00,1.00000000e+00},prob(percent)="100",prob+-sd="100+-0"]:
4.490825e-02[&length_mean=4.66085521e-02,length_median=4.49082500e-0
2,length_95%HPD={1.85248300e-02,7.68723800e-02}])
[&prob=1.00000000e+00,prob_stddev=0.00000000e+00,prob_range={1.00000
000e+00,1.00000000e+00},prob(percent)="100",prob+-sd="100+-0"]:
6.951818e-02[&length_mean=7.13966262e-02,length_median=6.95181800e-0
2,length_95%HPD={3.50674100e-02,1.11869800e-01}])
[&prob=9.99911115e-01,prob_stddev=1.25702285e-04,prob_range={9.99822
230e-01,1.00000000e+00},prob(percent)="100",prob+-sd="100+-0"]:
8.479642e-02[&length_mean=8.56642007e-02,length_median=8.47964200e-0
2,length_95%HPD={4.04295300e-02,1.32662500e-01}],
(((5[&prob=1.00000000e+00,prob_stddev=0.00000000e+00,prob_range={1.0
0000000e+00,1.00000000e+00},prob(percent)="100",prob+-sd="100+-0"]:
1.671644e-02[&length_mean=1.80153669e-02,length_median=1.67164400e-0
2,length_95%HPD={3.22274200e-03,3.57460700e-02}],
6[&prob=1.00000000e+00,prob_stddev=0.00000000e+00,prob_range={1.0000
0000e+00,1.00000000e+00},prob(percent)="100",prob+-sd="100+-0"]:
2.702201e-02[&length_mean=2.84562703e-02,length_median=2.70220100e-0
2,length_95%HPD={9.93405000e-03,4.89404300e-02}])
[&prob=1.00000000e+00,prob_stddev=0.00000000e+00,prob_range={1.00000
000e+00,1.00000000e+00},prob(percent)="100",prob+-sd="100+-0"]:
4.847490e-02[&length_mean=4.97118235e-02,length_median=4.84749000e-0
2,length_95%HPD={2.11954700e-02,7.95707100e-02}],
(7[&prob=1.00000000e+00,prob_stddev=0.00000000e+00,prob_range={1.000
00000e+00,1.00000000e+00},prob(percent)="100",prob+-sd="100+-0"]:
4.687216e-02[&length_mean=4.84194493e-02,length_median=4.68721600e-0
2,length_95%HPD={2.16508000e-02,7.84283300e-02}],
8[&prob=1.00000000e+00,prob_stddev=0.00000000e+00,prob_range={1.0000
0000e+00,1.00000000e+00},prob(percent)="100",prob+-sd="100+-0"]:
5.886186e-02[&length_mean=5.94923079e-02,length_median=5.88618600e-0
2,length_95%HPD={3.07838500e-02,9.33872400e-02}])
[&prob=1.00000000e+00,prob_stddev=0.00000000e+00,prob_range={1.00000
000e+00,1.00000000e+00},prob(percent)="100",prob+-sd="100+-0"]:
3.086136e-02[&length_mean=3.22212657e-02,length_median=3.08613600e-0
2,length_95%HPD={9.19857600e-03,5.71072000e-02}])
[&prob=9.29314253e-01,prob_stddev=3.80249413e-03,prob_range={9.26625
483e-01,9.32003022e-01},prob(percent)="93",prob+-sd="93+-0"]:
2.623634e-02[&length_mean=2.76896778e-02,length_median=2.62363400e-0
2,length_95%HPD={5.58777100e-03,5.14547000e-02}],
(((9[&prob=1.00000000e+00,prob_stddev=0.00000000e+00,prob_range={1.0
0000000e+00,1.00000000e+00},prob(percent)="100",prob+-sd="100+-0"]:
1.621398e-02[&length_mean=1.75560173e-02,length_median=1.62139800e-0
2,length_95%HPD={2.05055600e-03,3.53561300e-02}],
(10[&prob=1.00000000e+00,prob_stddev=0.00000000e+00,prob_range={1.00
000000e+00,1.00000000e+00},prob(percent)="100",prob+-sd="100+-0"]:
2.428208e-02[&length_mean=2.59778417e-02,length_median=2.42820800e-0
2,length_95%HPD={6.68541900e-03,4.86910500e-02}],

11[&prob=1.00000000e+00,prob_stddev=0.00000000e+00,prob_range={1.000
00000e+00,1.00000000e+00},prob(percent)="100",prob+-sd="100+-0"]:
5.859169e-02[&length_mean=5.97144452e-02,length_median=5.85916900e-0
2,length_95%HPD={2.98900800e-02,9.06182400e-02}])
[&prob=9.99888894e-01,prob_stddev=1.57127857e-04,prob_range={9.99777
788e-01,1.00000000e+00},prob(percent)="100",prob+-sd="100+-0"]:
2.236512e-02[&length_mean=2.37367936e-02,length_median=2.23651200e-0
2,length_95%HPD={4.96546300e-03,4.34118200e-02}])
[&prob=1.00000000e+00,prob_stddev=0.00000000e+00,prob_range={1.00000
000e+00,1.00000000e+00},prob(percent)="100",prob+-sd="100+-0"]:
4.167205e-02[&length_mean=4.34360101e-02,length_median=4.16720500e-0
2,length_95%HPD={1.48787600e-02,7.32291500e-02}],
(12[&prob=1.00000000e+00,prob_stddev=0.00000000e+00,prob_range={1.00
000000e+00,1.00000000e+00},prob(percent)="100",prob+-sd="100+-0"]:
1.335247e-02[&length_mean=1.46924663e-02,length_median=1.33524700e-0
2,length_95%HPD={2.14580400e-05,3.14459900e-02}],
13[&prob=1.00000000e+00,prob_stddev=0.00000000e+00,prob_range={1.000
00000e+00,1.00000000e+00},prob(percent)="100",prob+-sd="100+-0"]:
5.033730e-02[&length_mean=5.15277556e-02,length_median=5.03373000e-0
2,length_95%HPD={2.50201300e-02,8.11666200e-02}])
[&prob=1.00000000e+00,prob_stddev=0.00000000e+00,prob_range={1.00000
000e+00,1.00000000e+00},prob(percent)="100",prob+-sd="100+-0"]:
6.108082e-02[&length_mean=6.22290886e-02,length_median=6.10808200e-0
2,length_95%HPD={3.34237200e-02,9.32434000e-02}])
[&prob=6.88013866e-01,prob_stddev=5.44290896e-02,prob_range={6.49526
688e-01,7.26501044e-01},prob(percent)="69",prob+-sd="69+-5"]:
1.351972e-02[&length_mean=1.55760506e-02,length_median=1.35197200e-0
2,length_95%HPD={5.62775200e-05,3.48836000e-02}],
51[&prob=1.00000000e+00,prob_stddev=0.00000000e+00,prob_range={1.000
00000e+00,1.00000000e+00},prob(percent)="100",prob+-sd="100+-0"]:
2.026492e-01[&length_mean=2.04386176e-01,length_median=2.02649200e-0
1,length_95%HPD={1.48048900e-01,2.67610500e-01}])
[&prob=9.91711479e-01,prob_stddev=9.33339469e-03,prob_range={9.85111
773e-01,9.98311186e-01},prob(percent)="99",prob+-sd="99+-1"]:
2.582498e-02[&length_mean=2.71877650e-02,length_median=2.58249800e-0
2,length_95%HPD={3.61432600e-03,5.30917200e-02}],
(((14[&prob=1.00000000e+00,prob_stddev=0.00000000e+00,prob_range={1.
00000000e+00,1.00000000e+00},prob(percent)="100",prob+-sd="100+-0"]:
2.102370e-02[&length_mean=2.23625711e-02,length_median=2.10237000e-0
2,length_95%HPD={4.35955000e-03,4.30982900e-02}],
(15[&prob=1.00000000e+00,prob_stddev=0.00000000e+00,prob_range={1.00
000000e+00,1.00000000e+00},prob(percent)="100",prob+-sd="100+-0"]:
2.620791e-03[&length_mean=3.70384609e-03,length_median=2.62079100e-0
3,length_95%HPD={3.84318500e-06,1.07951600e-02}],
16[&prob=1.00000000e+00,prob_stddev=0.00000000e+00,prob_range={1.000
00000e+00,1.00000000e+00},prob(percent)="100",prob+-sd="100+-0"]:
2.562985e-03[&length_mean=3.69588607e-03,length_median=2.56298500e-0
3,length_95%HPD={1.78618800e-06,1.11277400e-02}])
[&prob=1.00000000e+00,prob_stddev=0.00000000e+00,prob_range={1.00000
000e+00,1.00000000e+00},prob(percent)="100",prob+-sd="100+-0"]:
2.310677e-02[&length_mean=2.43800868e-02,length_median=2.31067700e-0
2,length_95%HPD={5.23698800e-03,4.54076300e-02}])
[&prob=1.00000000e+00,prob_stddev=0.00000000e+00,prob_range={1.00000
000e+00,1.00000000e+00},prob(percent)="100",prob+-sd="100+-0"]:

6.518657e-02[&length_mean=6.69808607e-02,length_median=6.51865700e-0
2,length_95%HPD={3.14247900e-02,1.04774300e-01}],
(108[&prob=1.00000000e+00,prob_stddev=0.00000000e+00,prob_range={1.0
0000000e+00,1.00000000e+00},prob(percent)="100",prob+-sd="100+-0"]:
3.343498e-01[&length_mean=3.37167132e-01,length_median=3.34349800e-0
1,length_95%HPD={2.47111500e-01,4.26909700e-01}],
109[&prob=1.00000000e+00,prob_stddev=0.00000000e+00,prob_range={1.00
000000e+00,1.00000000e+00},prob(percent)="100",prob+-sd="100+-0"]:
2.049954e-01[&length_mean=2.07133435e-01,length_median=2.04995400e-0
1,length_95%HPD={1.30656100e-01,2.77219700e-01}])
[&prob=1.00000000e+00,prob_stddev=0.00000000e+00,prob_range={1.00000
000e+00,1.00000000e+00},prob(percent)="100",prob+-sd="100+-0"]:
1.874720e-01[&length_mean=1.90152620e-01,length_median=1.87472000e-0
1,length_95%HPD={1.22679300e-01,2.63707800e-01}])
[&prob=5.92329230e-01,prob_stddev=3.41595961e-01,prob_range={3.50784
410e-01,8.33874050e-01},prob(percent)="59",prob+-sd="59+-34"]:
2.341764e-02[&length_mean=2.48506502e-02,length_median=2.34176400e-0
2,length_95%HPD={3.11302000e-03,4.79303400e-02}],
((17[&prob=1.00000000e+00,prob_stddev=0.00000000e+00,prob_range={1.0
0000000e+00,1.00000000e+00},prob(percent)="100",prob+-sd="100+-0"]:
8.254652e-02[&length_mean=8.41186419e-02,length_median=8.25465200e-0
2,length_95%HPD={4.79471200e-02,1.26378700e-01}],
18[&prob=1.00000000e+00,prob_stddev=0.00000000e+00,prob_range={1.000
00000e+00,1.00000000e+00},prob(percent)="100",prob+-sd="100+-0"]:
1.268497e-01[&length_mean=1.28127999e-01,length_median=1.26849700e-0
1,length_95%HPD={7.89494000e-02,1.76012900e-01}])
[&prob=9.99933336e-01,prob_stddev=9.42767141e-05,prob_range={9.99866
673e-01,1.00000000e+00},prob(percent)="100",prob+-sd="100+-0"]:
7.269401e-02[&length_mean=7.42704605e-02,length_median=7.26940100e-0
2,length_95%HPD={4.19749000e-02,1.15317000e-01}],
(33[&prob=1.00000000e+00,prob_stddev=0.00000000e+00,prob_range={1.00
000000e+00,1.00000000e+00},prob(percent)="100",prob+-sd="100+-0"]:
2.804326e-02[&length_mean=2.95626675e-02,length_median=2.80432600e-0
2,length_95%HPD={8.04759700e-03,5.15057200e-02}],
34[&prob=1.00000000e+00,prob_stddev=0.00000000e+00,prob_range={1.000
00000e+00,1.00000000e+00},prob(percent)="100",prob+-sd="100+-0"]:
6.429792e-02[&length_mean=6.53487141e-02,length_median=6.42979200e-0
2,length_95%HPD={3.36007000e-02,9.61888200e-02}])
[&prob=1.00000000e+00,prob_stddev=0.00000000e+00,prob_range={1.00000
000e+00,1.00000000e+00},prob(percent)="100",prob+-sd="100+-0"]:
7.688772e-02[&length_mean=7.88081127e-02,length_median=7.68877200e-0
2,length_95%HPD={4.33339700e-02,1.19242000e-01}])
[&prob=6.10439536e-01,prob_stddev=2.32863484e-02,prob_range={5.93973
601e-01,6.26905471e-01},prob(percent)="61",prob+-sd="61+-2"]:
1.731986e-02[&length_mean=1.91125002e-02,length_median=1.73198600e-0
2,length_95%HPD={1.05803200e-03,3.85550600e-02}])
[&prob=5.45153549e-01,prob_stddev=3.10264666e-01,prob_range={3.25763
299e-01,7.64543798e-01},prob(percent)="55",prob+-sd="55+-31"]:
1.373214e-02[&length_mean=1.51986848e-02,length_median=1.37321400e-0
2,length_95%HPD={2.84516900e-04,3.26043600e-02}],
((((19[&prob=1.00000000e+00,prob_stddev=0.00000000e+00,prob_range={1
.00000000e+00,1.00000000e+00},prob(percent)="100",prob+-
sd="100+-0"]:
1.432098e-02[&length_mean=1.54715015e-02,length_median=1.43209800e-0

2,length_95%HPD={4.05916500e-04,3.19336600e-02}],
(20[&prob=1.00000000e+00,prob_stddev=0.00000000e+00,prob_range={1.00
000000e+00,1.00000000e+00},prob(percent)="100",prob+-sd="100+-0"]:
2.626409e-03[&length_mean=3.75261869e-03,length_median=2.62640900e-0
3,length_95%HPD={9.06559000e-07,1.14334600e-02}],
21[&prob=1.00000000e+00,prob_stddev=0.00000000e+00,prob_range={1.000
00000e+00,1.00000000e+00},prob(percent)="100",prob+-sd="100+-0"]:
2.626323e-03[&length_mean=3.77484888e-03,length_median=2.62632300e-0
3,length_95%HPD={2.55028400e-06,1.12516100e-02}])
[&prob=9.99777788e-01,prob_stddev=3.14255714e-04,prob_range={9.99555
575e-01,1.00000000e+00},prob(percent)="100",prob+-sd="100+-0"]:
2.475329e-02[&length_mean=2.61797313e-02,length_median=2.47532900e-0
2,length_95%HPD={7.72165100e-03,4.81128200e-02}])
[&prob=9.99977779e-01,prob_stddev=3.14255714e-05,prob_range={9.99955
558e-01,1.00000000e+00},prob(percent)="100",prob+-sd="100+-0"]:
3.409805e-02[&length_mean=3.58399728e-02,length_median=3.40980500e-0
2,length_95%HPD={1.08187300e-02,6.23222600e-02}],
(26[&prob=1.00000000e+00,prob_stddev=0.00000000e+00,prob_range={1.00
000000e+00,1.00000000e+00},prob(percent)="100",prob+-sd="100+-0"]:
2.798986e-02[&length_mean=2.93080368e-02,length_median=2.79898600e-0
2,length_95%HPD={9.63787500e-03,5.16206300e-02}],
27[&prob=1.00000000e+00,prob_stddev=0.00000000e+00,prob_range={1.000
00000e+00,1.00000000e+00},prob(percent)="100",prob+-sd="100+-0"]:
1.278090e-02[&length_mean=1.44650118e-02,length_median=1.27809000e-0
2,length_95%HPD={3.71696200e-04,3.16174700e-02}])
[&prob=5.90173770e-01,prob_stddev=7.92867165e-02,prob_range={5.34109
595e-01,6.46237945e-01},prob(percent)="59",prob+-sd="59+-8"]:
2.464545e-02[&length_mean=2.62327017e-02,length_median=2.46454500e-0
2,length_95%HPD={2.24299000e-03,5.19268400e-02}],
(29[&prob=1.00000000e+00,prob_stddev=0.00000000e+00,prob_range={1.00
000000e+00,1.00000000e+00},prob(percent)="100",prob+-sd="100+-0"]:
9.205824e-02[&length_mean=9.36617494e-02,length_median=9.20582400e-0
2,length_95%HPD={4.77563700e-02,1.39183900e-01}],
(30[&prob=1.00000000e+00,prob_stddev=0.00000000e+00,prob_range={1.00
000000e+00,1.00000000e+00},prob(percent)="100",prob+-sd="100+-0"]:
4.803876e-02[&length_mean=4.85401122e-02,length_median=4.80387600e-0
2,length_95%HPD={2.05243100e-02,7.52796100e-02}],
(31[&prob=1.00000000e+00,prob_stddev=0.00000000e+00,prob_range={1.00
000000e+00,1.00000000e+00},prob(percent)="100",prob+-sd="100+-0"]:
3.401226e-02[&length_mean=3.52494491e-02,length_median=3.40122600e-0
2,length_95%HPD={1.35711900e-02,6.07688300e-02}],
32[&prob=1.00000000e+00,prob_stddev=0.00000000e+00,prob_range={1.000
00000e+00,1.00000000e+00},prob(percent)="100",prob+-sd="100+-0"]:
3.298659e-02[&length_mean=3.40932702e-02,length_median=3.29865900e-0
2,length_95%HPD={1.25934600e-02,6.03454900e-02}])
[&prob=1.00000000e+00,prob_stddev=0.00000000e+00,prob_range={1.00000
000e+00,1.00000000e+00},prob(percent)="100",prob+-sd="100+-0"]:
3.943221e-02[&length_mean=4.07616364e-02,length_median=3.94322100e-0
2,length_95%HPD={1.43148300e-02,7.04432900e-02}])
[&prob=1.00000000e+00,prob_stddev=0.00000000e+00,prob_range={1.00000
000e+00,1.00000000e+00},prob(percent)="100",prob+-sd="100+-0"]:
5.649430e-02[&length_mean=5.82650571e-02,length_median=5.64943000e-0
2,length_95%HPD={2.48536600e-02,9.71207500e-02}])
[&prob=7.00324430e-01,prob_stddev=7.97581001e-02,prob_range={6.43926

937e-01,7.56721923e-01},prob(percent)="70",prob+-sd="70+-8"]:
3.864153e-02[&length_mean=4.04097902e-02,length_median=3.86415300e-0
2,length_95%HPD={1.45089200e-02,6.98156600e-02}],
(93[&prob=1.00000000e+00,prob_stddev=0.00000000e+00,prob_range={1.00
000000e+00,1.00000000e+00},prob(percent)="100",prob+-sd="100+-0"]:
2.443478e-01[&length_mean=2.45795771e-01,length_median=2.44347800e-0
1,length_95%HPD={1.68416300e-01,3.24947500e-01}],
111[&prob=1.00000000e+00,prob_stddev=0.00000000e+00,prob_range={1.00
000000e+00,1.00000000e+00},prob(percent)="100",prob+-sd="100+-0"]:
3.589529e-01[&length_mean=3.61720574e-01,length_median=3.58952900e-0
1,length_95%HPD={2.70860400e-01,4.55663600e-01}])
[&prob=9.99911115e-01,prob_stddev=1.25702285e-04,prob_range={9.99822
230e-01,1.00000000e+00},prob(percent)="100",prob+-sd="100+-0"]:
1.435284e-01[&length_mean=1.42086477e-01,length_median=1.43528400e-0
1,length_95%HPD={5.33964300e-02,2.21106900e-01}])
[&prob=6.07106351e-01,prob_stddev=3.28491497e-01,prob_range={3.74827
785e-01,8.39384916e-01},prob(percent)="61",prob+-sd="61+-33"]:
1.545903e-02[&length_mean=1.73110324e-02,length_median=1.54590300e-0
2,length_95%HPD={1.06276100e-03,3.64148200e-02}],
((((22[&prob=1.00000000e+00,prob_stddev=0.00000000e+00,prob_range={1
.00000000e+00,1.00000000e+00},prob(percent)="100",prob+-
sd="100+-0"]:
1.327345e-02[&length_mean=1.46205227e-02,length_median=1.32734500e-0
2,length_95%HPD={2.78043100e-04,2.92228000e-02}],
24[&prob=1.00000000e+00,prob_stddev=0.00000000e+00,prob_range={1.000
00000e+00,1.00000000e+00},prob(percent)="100",prob+-sd="100+-0"]:
2.448438e-02[&length_mean=2.56844466e-02,length_median=2.44843800e-0
2,length_95%HPD={7.83021500e-03,4.48309400e-02}])
[&prob=8.04608684e-01,prob_stddev=2.37891575e-02,prob_range={7.87787
209e-01,8.21430159e-01},prob(percent)="80",prob+-sd="80+-2"]:
8.161666e-03[&length_mean=9.56781814e-03,length_median=8.16166600e-0
3,length_95%HPD={2.42322800e-04,2.18728900e-02}],
23[&prob=1.00000000e+00,prob_stddev=0.00000000e+00,prob_range={1.000
00000e+00,1.00000000e+00},prob(percent)="100",prob+-sd="100+-0"]:
1.275382e-02[&length_mean=1.39186708e-02,length_median=1.27538200e-0
2,length_95%HPD={1.54424000e-03,2.88368400e-02}])
[&prob=9.91622595e-01,prob_stddev=2.60832242e-03,prob_range={9.89778
232e-01,9.93466957e-01},prob(percent)="99",prob+-sd="99+-0"]:
1.719414e-02[&length_mean=1.85107876e-02,length_median=1.71941400e-0
2,length_95%HPD={2.32798600e-03,3.75146800e-02}],
25[&prob=1.00000000e+00,prob_stddev=0.00000000e+00,prob_range={1.000
00000e+00,1.00000000e+00},prob(percent)="100",prob+-sd="100+-0"]:
2.346012e-02[&length_mean=2.57499647e-02,length_median=2.34601200e-0
2,length_95%HPD={3.72465900e-03,5.24733100e-02}])
[&prob=9.89133816e-01,prob_stddev=3.11113156e-03,prob_range={9.86933
914e-01,9.91333719e-01},prob(percent)="99",prob+-sd="99+-0"]:
2.904999e-02[&length_mean=3.02976169e-02,length_median=2.90499900e-0
2,length_95%HPD={8.42303000e-03,5.58393600e-02}],
(28[&prob=1.00000000e+00,prob_stddev=0.00000000e+00,prob_range={1.00
000000e+00,1.00000000e+00},prob(percent)="100",prob+-sd="100+-0"]:
4.782546e-02[&length_mean=4.98014968e-02,length_median=4.78254600e-0
2,length_95%HPD={1.08837200e-02,9.09927200e-02}],
110[&prob=1.00000000e+00,prob_stddev=0.00000000e+00,prob_range={1.00
000000e+00,1.00000000e+00},prob(percent)="100",prob+-sd="100+-0"]:

1.987158e-01[&length_mean=2.06358863e-01,length_median=1.98715800e-0
1,length_95%HPD={1.03832600e-01,3.35637200e-01}])
[&prob=7.90342651e-01,prob_stddev=5.94257554e-02,prob_range={7.48322
297e-01,8.32363006e-01},prob(percent)="79",prob+-sd="79+-6"]:
3.301016e-02[&length_mean=3.50859722e-02,length_median=3.30101600e-0
2,length_95%HPD={2.45698400e-03,6.79772600e-02}])
[&prob=7.86898360e-01,prob_stddev=4.48757159e-02,prob_range={7.55166
437e-01,8.18630283e-01},prob(percent)="79",prob+-sd="79+-4"]:
1.412490e-02[&length_mean=1.58907573e-02,length_median=1.41249000e-0
2,length_95%HPD={5.25390500e-05,3.40843000e-02}])
[&prob=7.20634638e-01,prob_stddev=3.93699558e-01,prob_range={4.42247
011e-01,9.99022266e-01},prob(percent)="72",prob+-sd="72+-39"]:
5.376146e-02[&length_mean=5.48745199e-02,length_median=5.37614600e-0
2,length_95%HPD={2.29506300e-02,9.04798300e-02}],
(((38[&prob=1.00000000e+00,prob_stddev=0.00000000e+00,prob_range={1.
00000000e+00,1.00000000e+00},prob(percent)="100",prob+-sd="100+-0"]:
6.848783e-02[&length_mean=6.96710282e-02,length_median=6.84878300e-0
2,length_95%HPD={3.45385200e-02,1.06966200e-01}],
39[&prob=1.00000000e+00,prob_stddev=0.00000000e+00,prob_range={1.000
00000e+00,1.00000000e+00},prob(percent)="100",prob+-sd="100+-0"]:
7.734581e-02[&length_mean=7.91330709e-02,length_median=7.73458100e-0
2,length_95%HPD={4.46636600e-02,1.22105700e-01}])
[&prob=1.00000000e+00,prob_stddev=0.00000000e+00,prob_range={1.00000
000e+00,1.00000000e+00},prob(percent)="100",prob+-sd="100+-0"]:
5.211396e-02[&length_mean=5.35860706e-02,length_median=5.21139600e-0
2,length_95%HPD={1.92307100e-02,8.87147500e-02}],
76[&prob=1.00000000e+00,prob_stddev=0.00000000e+00,prob_range={1.000
00000e+00,1.00000000e+00},prob(percent)="100",prob+-sd="100+-0"]:
2.775738e-01[&length_mean=2.79662382e-01,length_median=2.77573800e-0
1,length_95%HPD={2.06736300e-01,3.44645000e-01}])
[&prob=9.99777788e-01,prob_stddev=3.14255714e-04,prob_range={9.99555
575e-01,1.00000000e+00},prob(percent)="100",prob+-sd="100+-0"]:
7.370901e-02[&length_mean=7.62209820e-02,length_median=7.37090100e-0
2,length_95%HPD={3.56253900e-02,1.20850800e-01}],
((41[&prob=1.00000000e+00,prob_stddev=0.00000000e+00,prob_range={1.0
0000000e+00,1.00000000e+00},prob(percent)="100",prob+-sd="100+-0"]:
3.849514e-02[&length_mean=3.96725112e-02,length_median=3.84951400e-0
2,length_95%HPD={8.81176700e-03,7.12263800e-02}],
42[&prob=1.00000000e+00,prob_stddev=0.00000000e+00,prob_range={1.000
00000e+00,1.00000000e+00},prob(percent)="100",prob+-sd="100+-0"]:
1.587127e-01[&length_mean=1.60009387e-01,length_median=1.58712700e-0
1,length_95%HPD={1.08166000e-01,2.16056700e-01}])
[&prob=1.00000000e+00,prob_stddev=0.00000000e+00,prob_range={1.00000
000e+00,1.00000000e+00},prob(percent)="100",prob+-sd="100+-0"]:
1.610728e-01[&length_mean=1.61579056e-01,length_median=1.61072800e-0
1,length_95%HPD={1.02639500e-01,2.20171500e-01}],
(((43[&prob=1.00000000e+00,prob_stddev=0.00000000e+00,prob_range={1.
00000000e+00,1.00000000e+00},prob(percent)="100",prob+-sd="100+-0"]:
1.299927e-01[&length_mean=1.32461592e-01,length_median=1.29992700e-0
1,length_95%HPD={7.56636800e-02,1.89916300e-01}],
46[&prob=1.00000000e+00,prob_stddev=0.00000000e+00,prob_range={1.000
00000e+00,1.00000000e+00},prob(percent)="100",prob+-sd="100+-0"]:
8.332004e-02[&length_mean=8.39477365e-02,length_median=8.33200400e-0
2,length_95%HPD={4.54240700e-02,1.19815200e-01}],

47[&prob=1.00000000e+00,prob_stddev=0.00000000e+00,prob_range={1.000
00000e+00,1.00000000e+00},prob(percent)="100",prob+-sd="100+-0"]:
5.594113e-02[&length_mean=5.75916277e-02,length_median=5.59411300e-0
2,length_95%HPD={2.48579600e-02,9.23755900e-02}],
49[&prob=1.00000000e+00,prob_stddev=0.00000000e+00,prob_range={1.000
00000e+00,1.00000000e+00},prob(percent)="100",prob+-sd="100+-0"]:
8.394696e-02[&length_mean=8.68400547e-02,length_median=8.39469600e-0
2,length_95%HPD={3.90202800e-02,1.43432000e-01}])
[&prob=9.56357495e-01,prob_stddev=6.17198222e-02,prob_range={9.12714
990e-01,1.00000000e+00},prob(percent)="96",prob+-sd="96+-6"]:
3.997353e-02[&length_mean=4.13331524e-02,length_median=3.99735300e-0
2,length_95%HPD={1.15183500e-02,7.34975600e-02}],
(44[&prob=1.00000000e+00,prob_stddev=0.00000000e+00,prob_range={1.00
000000e+00,1.00000000e+00},prob(percent)="100",prob+-sd="100+-0"]:
7.331502e-02[&length_mean=7.41792095e-02,length_median=7.33150200e-0
2,length_95%HPD={3.84787000e-02,1.12859700e-01}],
45[&prob=1.00000000e+00,prob_stddev=0.00000000e+00,prob_range={1.000
00000e+00,1.00000000e+00},prob(percent)="100",prob+-sd="100+-0"]:
1.706571e-02[&length_mean=1.97622207e-02,length_median=1.70657100e-0
2,length_95%HPD={8.81391100e-06,4.68381300e-02}],
48[&prob=1.00000000e+00,prob_stddev=0.00000000e+00,prob_range={1.000
00000e+00,1.00000000e+00},prob(percent)="100",prob+-sd="100+-0"]:
1.415135e-02[&length_mean=1.75631914e-02,length_median=1.41513500e-0
2,length_95%HPD={8.16693800e-07,4.54463500e-02}])
[&prob=1.00000000e+00,prob_stddev=0.00000000e+00,prob_range={1.00000
000e+00,1.00000000e+00},prob(percent)="100",prob+-sd="100+-0"]:
1.175133e-01[&length_mean=1.19074057e-01,length_median=1.17513300e-0
1,length_95%HPD={7.40415500e-02,1.68987800e-01}])
[&prob=9.50646638e-01,prob_stddev=6.97961940e-02,prob_range={9.01293
276e-01,1.00000000e+00},prob(percent)="95",prob+-sd="95+-7"]:
5.972407e-02[&length_mean=6.14782478e-02,length_median=5.97240700e-0
2,length_95%HPD={2.25037900e-02,1.02834400e-01}],
(50[&prob=1.00000000e+00,prob_stddev=0.00000000e+00,prob_range={1.00
000000e+00,1.00000000e+00},prob(percent)="100",prob+-sd="100+-0"]:
1.792123e-01[&length_mean=1.82736075e-01,length_median=1.79212300e-0
1,length_95%HPD={1.24811800e-01,2.49394700e-01}],
((((52[&prob=1.00000000e+00,prob_stddev=0.00000000e+00,prob_range={
1.00000000e+00,1.00000000e+00},prob(percent)="100",prob+-
sd="100+-0"]:
1.380551e-01[&length_mean=1.41152864e-01,length_median=1.38055100e-0
1,length_95%HPD={8.00322800e-02,2.12150500e-01}],
55[&prob=1.00000000e+00,prob_stddev=0.00000000e+00,prob_range={1.000
00000e+00,1.00000000e+00},prob(percent)="100",prob+-sd="100+-0"]:
1.245720e-01[&length_mean=1.26753086e-01,length_median=1.24572000e-0
1,length_95%HPD={7.43695000e-02,1.86430500e-01}])
[&prob=9.54557575e-01,prob_stddev=9.14484127e-03,prob_range={9.48091
196e-01,9.61023954e-01},prob(percent)="95",prob+-sd="95+-1"]:
4.772423e-02[&length_mean=4.97598717e-02,length_median=4.77242300e-0
2,length_95%HPD={4.12772500e-03,9.51672200e-02}],
(53[&prob=1.00000000e+00,prob_stddev=0.00000000e+00,prob_range={1.00
000000e+00,1.00000000e+00},prob(percent)="100",prob+-sd="100+-0"]:
1.016211e-01[&length_mean=1.02879025e-01,length_median=1.01621100e-0
1,length_95%HPD={5.78408300e-02,1.46479000e-01}],
54[&prob=1.00000000e+00,prob_stddev=0.00000000e+00,prob_range={1.000

00000e+00,1.00000000e+00},prob(percent)="100",prob+-sd="100+-0"]:
8.328268e-02[&length_mean=8.35359935e-02,length_median=8.32826800e-0
2,length_95%HPD={4.34637900e-02,1.21396100e-01}])
[&prob=1.00000000e+00,prob_stddev=0.00000000e+00,prob_range={1.00000
000e+00,1.00000000e+00},prob(percent)="100",prob+-sd="100+-0"]:
5.494970e-02[&length_mean=5.65226494e-02,length_median=5.49497000e-0
2,length_95%HPD={2.35437200e-02,9.40520400e-02}])
[&prob=1.00000000e+00,prob_stddev=0.00000000e+00,prob_range={1.00000
000e+00,1.00000000e+00},prob(percent)="100",prob+-sd="100+-0"]:
7.042089e-02[&length_mean=7.16784676e-02,length_median=7.04208900e-0
2,length_95%HPD={2.98301900e-02,1.12857100e-01}],
(56[&prob=1.00000000e+00,prob_stddev=0.00000000e+00,prob_range={1.00
000000e+00,1.00000000e+00},prob(percent)="100",prob+-sd="100+-0"]:
2.429335e-01[&length_mean=2.40206328e-01,length_median=2.42933500e-0
1,length_95%HPD={1.46296700e-01,3.35763500e-01}],
(92[&prob=1.00000000e+00,prob_stddev=0.00000000e+00,prob_range={1.00
000000e+00,1.00000000e+00},prob(percent)="100",prob+-sd="100+-0"]:
3.163613e-01[&length_mean=3.17954686e-01,length_median=3.16361300e-0
1,length_95%HPD={2.00773400e-01,4.30270000e-01}],
128[&prob=1.00000000e+00,prob_stddev=0.00000000e+00,prob_range={1.00
000000e+00,1.00000000e+00},prob(percent)="100",prob+-sd="100+-0"]:
3.463398e-01[&length_mean=3.48417713e-01,length_median=3.46339800e-0
1,length_95%HPD={2.10768600e-01,4.90912100e-01}])
[&prob=5.38376072e-01,prob_stddev=2.67745868e-02,prob_range={5.19443
580e-01,5.57308564e-01},prob(percent)="54",prob+-sd="54+-3"]:
8.591740e-02[&length_mean=8.85236834e-02,length_median=8.59174000e-0
2,length_95%HPD={1.98843200e-02,1.65336900e-01}])
[&prob=5.55286432e-01,prob_stddev=2.46690735e-02,prob_range={5.37842
763e-01,5.72730101e-01},prob(percent)="56",prob+-sd="56+-2"]:
4.417167e-02[&length_mean=4.69699521e-02,length_median=4.41716700e-0
2,length_95%HPD={4.95510500e-03,9.05469500e-02}],
(((71[&prob=1.00000000e+00,prob_stddev=0.00000000e+00,prob_range={1.
00000000e+00,1.00000000e+00},prob(percent)="100",prob+-sd="100+-0"]:
2.519836e-01[&length_mean=2.52708919e-01,length_median=2.51983600e-0
1,length_95%HPD={1.83402400e-01,3.24946100e-01}],
74[&prob=1.00000000e+00,prob_stddev=0.00000000e+00,prob_range={1.000
00000e+00,1.00000000e+00},prob(percent)="100",prob+-sd="100+-0"]:
2.268751e-01[&length_mean=2.28508260e-01,length_median=2.26875100e-0
1,length_95%HPD={1.58708700e-01,2.96902100e-01}])
[&prob=5.77996533e-01,prob_stddev=1.08418221e-02,prob_range={5.70330
208e-01,5.85662859e-01},prob(percent)="58",prob+-sd="58+-1"]:
4.764246e-02[&length_mean=5.00654321e-02,length_median=4.76424600e-0
2,length_95%HPD={5.88832200e-03,9.33429600e-02}],
73[&prob=1.00000000e+00,prob_stddev=0.00000000e+00,prob_range={1.000
00000e+00,1.00000000e+00},prob(percent)="100",prob+-sd="100+-0"]:
1.224448e-01[&length_mean=1.25228109e-01,length_median=1.22444800e-0
1,length_95%HPD={6.94781300e-02,1.84700600e-01}])
[&prob=9.32714102e-01,prob_stddev=1.96095565e-02,prob_range={9.18848
051e-01,9.46580152e-01},prob(percent)="93",prob+-sd="93+-2"]:
6.714377e-02[&length_mean=7.04146692e-02,length_median=6.71437700e-0
2,length_95%HPD={2.09939100e-02,1.24318200e-01}],
72[&prob=1.00000000e+00,prob_stddev=0.00000000e+00,prob_range={1.000
00000e+00,1.00000000e+00},prob(percent)="100",prob+-sd="100+-0"]:
2.252559e-01[&length_mean=2.27724149e-01,length_median=2.25255900e-0

1,length_95%HPD={1.49996900e-01,2.96577200e-01}])
[&prob=9.99800009e-01,prob_stddev=9.42767141e-05,prob_range={9.99733
345e-01,9.99866673e-01},prob(percent)="100",prob+-sd="100+-0"]:
9.900275e-02[&length_mean=1.01397678e-01,length_median=9.90027500e-0
2,length_95%HPD={4.38929500e-02,1.62364700e-01}])
[&prob=8.42095907e-01,prob_stddev=1.43300605e-02,prob_range={8.31963
024e-01,8.52228790e-01},prob(percent)="84",prob+-sd="84+-1"]:
4.877577e-02[&length_mean=5.02403675e-02,length_median=4.87757700e-0
2,length_95%HPD={1.66614800e-02,8.71060500e-02}],
(((57[&prob=1.00000000e+00,prob_stddev=0.00000000e+00,prob_range={1.
00000000e+00,1.00000000e+00},prob(percent)="100",prob+-sd="100+-0"]:
3.396131e-02[&length_mean=3.47751505e-02,length_median=3.39613100e-0
2,length_95%HPD={1.22631000e-02,5.88640500e-02}],
60[&prob=1.00000000e+00,prob_stddev=0.00000000e+00,prob_range={1.000
00000e+00,1.00000000e+00},prob(percent)="100",prob+-sd="100+-0"]:
1.502367e-02[&length_mean=1.71858128e-02,length_median=1.50236700e-0
2,length_95%HPD={1.73720700e-03,3.83303300e-02}],
61[&prob=1.00000000e+00,prob_stddev=0.00000000e+00,prob_range={1.000
00000e+00,1.00000000e+00},prob(percent)="100",prob+-sd="100+-0"]:
6.135945e-02[&length_mean=6.37965871e-02,length_median=6.13594500e-0
2,length_95%HPD={2.34820000e-02,1.10573600e-01}])
[&prob=5.99084485e-01,prob_stddev=8.04494627e-03,prob_range={5.93395
849e-01,6.04773121e-01},prob(percent)="60",prob+-sd="60+-1"]:
1.144884e-02[&length_mean=1.31994615e-02,length_median=1.14488400e-0
2,length_95%HPD={4.90416200e-05,2.99993000e-02}],
58[&prob=1.00000000e+00,prob_stddev=0.00000000e+00,prob_range={1.000
00000e+00,1.00000000e+00},prob(percent)="100",prob+-sd="100+-0"]:
2.965549e-02[&length_mean=3.33314540e-02,length_median=2.96554900e-0
2,length_95%HPD={3.16628500e-03,7.12607300e-02}],
59[&prob=1.00000000e+00,prob_stddev=0.00000000e+00,prob_range={1.000
00000e+00,1.00000000e+00},prob(percent)="100",prob+-sd="100+-0"]:
4.252003e-02[&length_mean=4.41733881e-02,length_median=4.25200300e-0
2,length_95%HPD={1.60130400e-02,7.42874600e-02}],
75[&prob=1.00000000e+00,prob_stddev=0.00000000e+00,prob_range={1.000
00000e+00,1.00000000e+00},prob(percent)="100",prob+-sd="100+-0"]:
2.999978e-01[&length_mean=3.01904688e-01,length_median=2.99997800e-0
1,length_95%HPD={2.11158200e-01,3.88155200e-01}])
[&prob=1.00000000e+00,prob_stddev=0.00000000e+00,prob_range={1.00000
000e+00,1.00000000e+00},prob(percent)="100",prob+-sd="100+-0"]:
2.054054e-01[&length_mean=2.06455303e-01,length_median=2.05405400e-0
1,length_95%HPD={1.26721600e-01,2.79400000e-01}],
(((((62[&prob=1.00000000e+00,prob_stddev=0.00000000e+00,prob_range={
1.00000000e+00,1.00000000e+00},prob(percent)="100",prob+-
sd="100+-0"]:
1.268372e-02[&length_mean=1.38024431e-02,length_median=1.26837200e-0
2,length_95%HPD={1.15180400e-05,3.04578100e-02}],
(63[&prob=1.00000000e+00,prob_stddev=0.00000000e+00,prob_range={1.00
000000e+00,1.00000000e+00},prob(percent)="100",prob+-sd="100+-0"]:
3.078607e-02[&length_mean=3.27097269e-02,length_median=3.07860700e-0
2,length_95%HPD={8.52719300e-03,5.87910800e-02}],
124[&prob=1.00000000e+00,prob_stddev=0.00000000e+00,prob_range={1.00
000000e+00,1.00000000e+00},prob(percent)="100",prob+-sd="100+-0"]:
1.820197e-01[&length_mean=1.80925403e-01,length_median=1.82019700e-0
1,length_95%HPD={3.11921400e-02,3.02320100e-01}])

[&prob=7.95297987e-01,prob_stddev=8.98771341e-03,prob_range={7.88942
714e-01,8.01653260e-01},prob(percent)="80",prob+-sd="80+-1"]:
2.278589e-02[&length_mean=2.42719774e-02,length_median=2.27858900e-0
2,length_95%HPD={3.86289900e-03,4.88879100e-02}])
[&prob=8.44495800e-01,prob_stddev=4.52528228e-03,prob_range={8.41295
942e-01,8.47695658e-01},prob(percent)="84",prob+-sd="84+-0"]:
5.964297e-02[&length_mean=6.08989587e-02,length_median=5.96429700e-0
2,length_95%HPD={2.76909800e-02,9.81831300e-02}],
64[&prob=1.00000000e+00,prob_stddev=0.00000000e+00,prob_range={1.000
00000e+00,1.00000000e+00},prob(percent)="100",prob+-sd="100+-0"]:
8.663994e-02[&length_mean=8.76140297e-02,length_median=8.66399400e-0
2,length_95%HPD={4.34548300e-02,1.29677100e-01}],
65[&prob=1.00000000e+00,prob_stddev=0.00000000e+00,prob_range={1.000
00000e+00,1.00000000e+00},prob(percent)="100",prob+-sd="100+-0"]:
1.318239e-01[&length_mean=1.32244611e-01,length_median=1.31823900e-0
1,length_95%HPD={8.25951600e-02,1.84253500e-01}],
70[&prob=1.00000000e+00,prob_stddev=0.00000000e+00,prob_range={1.000
00000e+00,1.00000000e+00},prob(percent)="100",prob+-sd="100+-0"]:
2.085896e-01[&length_mean=2.10974702e-01,length_median=2.08589600e-0
1,length_95%HPD={5.92409200e-02,3.78380100e-01}])
[&prob=7.70921292e-01,prob_stddev=1.37958258e-02,prob_range={7.61166
170e-01,7.80676414e-01},prob(percent)="77",prob+-sd="77+-1"]:
3.575350e-02[&length_mean=3.75462788e-02,length_median=3.57535000e-0
2,length_95%HPD={5.68140400e-03,7.56063100e-02}],
67[&prob=1.00000000e+00,prob_stddev=0.00000000e+00,prob_range={1.000
00000e+00,1.00000000e+00},prob(percent)="100",prob+-sd="100+-0"]:
2.018539e-01[&length_mean=2.02370511e-01,length_median=2.01853900e-0
1,length_95%HPD={1.43620800e-01,2.64025500e-01}])
[&prob=9.78400960e-01,prob_stddev=1.75983200e-03,prob_range={9.77156
571e-01,9.79645349e-01},prob(percent)="98",prob+-sd="98+-0"]:
4.840353e-02[&length_mean=4.98173967e-02,length_median=4.84035300e-0
2,length_95%HPD={1.16438000e-02,8.87835000e-02}],
66[&prob=1.00000000e+00,prob_stddev=0.00000000e+00,prob_range={1.000
00000e+00,1.00000000e+00},prob(percent)="100",prob+-sd="100+-0"]:
1.510927e-01[&length_mean=1.51825865e-01,length_median=1.51092700e-0
1,length_95%HPD={1.00334500e-01,2.05277200e-01}])
[&prob=9.67379228e-01,prob_stddev=6.91362570e-04,prob_range={9.66890
360e-01,9.67868095e-01},prob(percent)="97",prob+-sd="97+-0"]:
5.069788e-02[&length_mean=5.25638279e-02,length_median=5.06978800e-0
2,length_95%HPD={1.32570600e-02,9.20854000e-02}],
(68[&prob=1.00000000e+00,prob_stddev=0.00000000e+00,prob_range={1.00
000000e+00,1.00000000e+00},prob(percent)="100",prob+-sd="100+-0"]:
3.792878e-03[&length_mean=5.70920558e-03,length_median=3.79287800e-0
3,length_95%HPD={4.08543000e-06,1.70366500e-02}],
69[&prob=1.00000000e+00,prob_stddev=0.00000000e+00,prob_range={1.000
00000e+00,1.00000000e+00},prob(percent)="100",prob+-sd="100+-0"]:
7.344739e-02[&length_mean=7.55081721e-02,length_median=7.34473900e-0
2,length_95%HPD={3.67537300e-02,1.17213900e-01}])
[&prob=1.00000000e+00,prob_stddev=0.00000000e+00,prob_range={1.00000
000e+00,1.00000000e+00},prob(percent)="100",prob+-sd="100+-0"]:
2.050118e-01[&length_mean=2.06729881e-01,length_median=2.05011800e-0
1,length_95%HPD={1.45914300e-01,2.70488000e-01}])
[&prob=9.98088974e-01,prob_stddev=2.38834342e-03,prob_range={9.96400
160e-01,9.99777788e-01},prob(percent)="100",prob+-sd="100+-0"]:

6.509545e-02[&length_mean=6.68077425e-02,length_median=6.50954500e-0
2,length_95%HPD={2.22500200e-02,1.11080700e-01}])
[&prob=8.52317675e-01,prob_stddev=1.20674194e-02,prob_range={8.43784
721e-01,8.60850629e-01},prob(percent)="85",prob+-sd="85+-1"]:
6.329698e-02[&length_mean=6.42149533e-02,length_median=6.32969800e-0
2,length_95%HPD={2.21267800e-02,1.08204800e-01}])
[&prob=9.07404115e-01,prob_stddev=1.03390130e-02,prob_range={9.00093
329e-01,9.14714902e-01},prob(percent)="91",prob+-sd="91+-1"]:
4.617639e-02[&length_mean=4.84057017e-02,length_median=4.61763900e-0
2,length_95%HPD={9.12161500e-03,8.74325500e-02}],
((((((77[&prob=1.00000000e+00,prob_stddev=0.00000000e+00,prob_range=
{1.00000000e+00,1.00000000e+00},prob(percent)="100",prob+-
sd="100+-0"]:
3.425516e-02[&length_mean=3.54104359e-02,length_median=3.42551600e-0
2,length_95%HPD={1.37600900e-02,5.96415600e-02}],
78[&prob=1.00000000e+00,prob_stddev=0.00000000e+00,prob_range={1.000
00000e+00,1.00000000e+00},prob(percent)="100",prob+-sd="100+-0"]:
2.357929e-02[&length_mean=2.50968513e-02,length_median=2.35792900e-0
2,length_95%HPD={7.15599800e-03,4.69094100e-02}])
[&prob=1.00000000e+00,prob_stddev=0.00000000e+00,prob_range={1.00000
000e+00,1.00000000e+00},prob(percent)="100",prob+-sd="100+-0"]:
6.096247e-02[&length_mean=6.22448897e-02,length_median=6.09624700e-0
2,length_95%HPD={3.05780300e-02,9.60448900e-02}],
(79[&prob=1.00000000e+00,prob_stddev=0.00000000e+00,prob_range={1.00
000000e+00,1.00000000e+00},prob(percent)="100",prob+-sd="100+-0"]:
1.910946e-02[&length_mean=2.02942279e-02,length_median=1.91094600e-0
2,length_95%HPD={1.36033700e-05,4.02596100e-02}],
(80[&prob=1.00000000e+00,prob_stddev=0.00000000e+00,prob_range={1.00
000000e+00,1.00000000e+00},prob(percent)="100",prob+-sd="100+-0"]:
5.857369e-02[&length_mean=5.92580272e-02,length_median=5.85736900e-0
2,length_95%HPD={3.47691200e-05,1.18302800e-01}],
81[&prob=1.00000000e+00,prob_stddev=0.00000000e+00,prob_range={1.000
00000e+00,1.00000000e+00},prob(percent)="100",prob+-sd="100+-0"]:
5.066826e-02[&length_mean=5.22844708e-02,length_median=5.06682600e-0
2,length_95%HPD={1.10451600e-05,1.08850600e-01}])
[&prob=6.96769032e-01,prob_stddev=2.06151748e-02,prob_range={6.82191
903e-01,7.11346162e-01},prob(percent)="70",prob+-sd="70+-2"]:
4.190913e-02[&length_mean=4.35969164e-02,length_median=4.19091300e-0
2,length_95%HPD={2.75971700e-04,8.72098200e-02}])
[&prob=9.94111373e-01,prob_stddev=4.11674985e-03,prob_range={9.91200
391e-01,9.97022355e-01},prob(percent)="99",prob+-sd="99+-0"]:
3.111983e-02[&length_mean=3.26092866e-02,length_median=3.11198300e-0
2,length_95%HPD={8.11386600e-03,6.03116900e-02}])
[&prob=1.00000000e+00,prob_stddev=0.00000000e+00,prob_range={1.00000
000e+00,1.00000000e+00},prob(percent)="100",prob+-sd="100+-0"]:
1.087744e-01[&length_mean=1.10548547e-01,length_median=1.08774400e-0
1,length_95%HPD={6.29463800e-02,1.63087500e-01}],
(82[&prob=1.00000000e+00,prob_stddev=0.00000000e+00,prob_range={1.00
000000e+00,1.00000000e+00},prob(percent)="100",prob+-sd="100+-0"]:
8.091884e-02[&length_mean=8.23397781e-02,length_median=8.09188400e-0
2,length_95%HPD={4.57388200e-02,1.24073600e-01}],
83[&prob=1.00000000e+00,prob_stddev=0.00000000e+00,prob_range={1.000
00000e+00,1.00000000e+00},prob(percent)="100",prob+-sd="100+-0"]:
5.409734e-02[&length_mean=5.56119359e-02,length_median=5.40973400e-0

2,length_95%HPD={2.39609500e-02,9.11461500e-02}])
[&prob=1.00000000e+00,prob_stddev=0.00000000e+00,prob_range={1.00000
000e+00,1.00000000e+00},prob(percent)="100",prob+-sd="100+-0"]:
8.912470e-02[&length_mean=9.06045036e-02,length_median=8.91247000e-0
2,length_95%HPD={4.82786900e-02,1.38391800e-01}])
[&prob=7.99853340e-01,prob_stddev=8.89343670e-03,prob_range={7.93564
730e-01,8.06141949e-01},prob(percent)="80",prob+-sd="80+-1"]:
4.873007e-02[&length_mean=4.96705094e-02,length_median=4.87300700e-0
2,length_95%HPD={2.73535800e-04,9.70410300e-02}],
91[&prob=1.00000000e+00,prob_stddev=0.00000000e+00,prob_range={1.000
00000e+00,1.00000000e+00},prob(percent)="100",prob+-sd="100+-0"]:
2.383426e-01[&length_mean=2.42210837e-01,length_median=2.38342600e-0
1,length_95%HPD={1.30982900e-01,3.53102300e-01}])
[&prob=7.24567797e-01,prob_stddev=2.89429512e-02,prob_range={7.04102
040e-01,7.45033554e-01},prob(percent)="72",prob+-sd="72+-3"]:
5.284775e-02[&length_mean=5.41973770e-02,length_median=5.28477500e-0
2,length_95%HPD={8.76692400e-04,1.02579300e-01}],
(((84[&prob=1.00000000e+00,prob_stddev=0.00000000e+00,prob_range={1.
00000000e+00,1.00000000e+00},prob(percent)="100",prob+-sd="100+-0"]:
1.064658e-01[&length_mean=1.07462120e-01,length_median=1.06465800e-0
1,length_95%HPD={5.29611700e-02,1.60499200e-01}],
107[&prob=1.00000000e+00,prob_stddev=0.00000000e+00,prob_range={1.00
000000e+00,1.00000000e+00},prob(percent)="100",prob+-sd="100+-0"]:
4.250065e-01[&length_mean=4.28778016e-01,length_median=4.25006500e-0
1,length_95%HPD={3.27369300e-01,5.38561200e-01}])
[&prob=9.94978001e-01,prob_stddev=6.09656084e-03,prob_range={9.90667
081e-01,9.99288920e-01},prob(percent)="99",prob+-sd="99+-1"]:
5.815682e-02[&length_mean=6.03502205e-02,length_median=5.81568200e-0
2,length_95%HPD={1.68871200e-02,1.06685000e-01}],
((85[&prob=1.00000000e+00,prob_stddev=0.00000000e+00,prob_range={1.0
0000000e+00,1.00000000e+00},prob(percent)="100",prob+-sd="100+-0"]:
1.362693e-01[&length_mean=1.39987667e-01,length_median=1.36269300e-0
1,length_95%HPD={7.68544700e-02,2.16119600e-01}],
90[&prob=1.00000000e+00,prob_stddev=0.00000000e+00,prob_range={1.000
00000e+00,1.00000000e+00},prob(percent)="100",prob+-sd="100+-0"]:
1.732650e-01[&length_mean=1.78677199e-01,length_median=1.73265000e-0
1,length_95%HPD={8.25050200e-02,2.92938100e-01}])
[&prob=6.60348429e-01,prob_stddev=1.55556578e-02,prob_range={6.49348
918e-01,6.71347940e-01},prob(percent)="66",prob+-sd="66+-2"]:
2.736960e-02[&length_mean=3.22706790e-02,length_median=2.73696000e-0
2,length_95%HPD={9.34769700e-06,7.72196600e-02}],
86[&prob=1.00000000e+00,prob_stddev=0.00000000e+00,prob_range={1.000
00000e+00,1.00000000e+00},prob(percent)="100",prob+-sd="100+-0"]:
4.412274e-02[&length_mean=4.57887307e-02,length_median=4.41227400e-0
2,length_95%HPD={1.24091400e-02,8.14954500e-02}])
[&prob=9.82822986e-01,prob_stddev=2.16836442e-03,prob_range={9.81289
720e-01,9.84356251e-01},prob(percent)="98",prob+-sd="98+-0"]:
5.686984e-02[&length_mean=5.78862872e-02,length_median=5.68698400e-0
2,length_95%HPD={1.53414200e-02,9.87771900e-02}])
[&prob=9.62534998e-01,prob_stddev=1.88553428e-04,prob_range={9.62401
671e-01,9.62668326e-01},prob(percent)="96",prob+-sd="96+-0"]:
5.208023e-02[&length_mean=5.32783625e-02,length_median=5.20802300e-0
2,length_95%HPD={1.60116900e-02,9.36309600e-02}],
(87[&prob=1.00000000e+00,prob_stddev=0.00000000e+00,prob_range={1.00

```
000000e+00,1.00000000e+00},prob(percent)="100",prob+-sd="100+-0"]:
6.204558e-02[&length_mean=6.32744616e-02,length_median=6.20455800e-0
2,length_95%HPD={2.41788200e-02,1.07162900e-01}],
88[&prob=1.00000000e+00,prob_stddev=0.00000000e+00,prob_range={1.000
00000e+00,1.00000000e+00},prob(percent)="100",prob+-sd="100+-0"]:
9.604900e-02[&length_mean=1.00649219e-01,length_median=9.60490000e-0
2,length_95%HPD={2.69458500e-02,1.81601900e-01}],
89[&prob=1.00000000e+00,prob_stddev=0.00000000e+00,prob_range={1.000
00000e+00,1.00000000e+00},prob(percent)="100",prob+-sd="100+-0"]:
3.623574e-02[&length_mean=3.80240863e-02,length_median=3.62357400e-0
2,length_95%HPD={1.21666900e-03,7.39539500e-02}])
[&prob=1.00000000e+00,prob_stddev=0.00000000e+00,prob_range={1.00000
000e+00,1.00000000e+00},prob(percent)="100",prob+-sd="100+-0"]:
2.179349e-01[&length_mean=2.18744747e-01,length_median=2.17934900e-0
1,length_95%HPD={1.45615700e-01,2.87278800e-01}])
[&prob=9.86333941e-01,prob_stddev=3.67679185e-03,prob_range={9.83734
056e-01,9.88933825e-01},prob(percent)="99",prob+-sd="99+-0"]:
6.179330e-02[&length_mean=6.39898695e-02,length_median=6.17933000e-0
2,length_95%HPD={2.09398500e-02,1.13484800e-01}])
[&prob=1.00000000e+00,prob_stddev=0.00000000e+00,prob_range={1.00000
000e+00,1.00000000e+00},prob(percent)="100",prob+-sd="100+-0"]:
1.465147e-01[&length_mean=1.49794880e-01,length_median=1.46514700e-0
1,length_95%HPD={7.08669000e-02,2.47595500e-01}],
127[&prob=1.00000000e+00,prob_stddev=0.00000000e+00,prob_range={1.00
000000e+00,1.00000000e+00},prob(percent)="100",prob+-sd="100+-0"]:
5.288997e-01[&length_mean=5.32571232e-01,length_median=5.28899700e-0
1,length_95%HPD={4.10030500e-01,6.59206100e-01}])
[&prob=5.70152438e-01,prob_stddev=7.03932798e-03,prob_range={5.65174
881e-01,5.75129994e-01},prob(percent)="57",prob+-sd="57+-1"]:
9.032668e-02[&length_mean=9.26577204e-02,length_median=9.03266800e-0
2,length_95%HPD={3.59155400e-02,1.48830900e-01}],
(94[&prob=1.00000000e+00,prob_stddev=0.00000000e+00,prob_range={1.00
000000e+00,1.00000000e+00},prob(percent)="100",prob+-sd="100+-0"]:
3.896353e-01[&length_mean=3.92520035e-01,length_median=3.89635300e-0
1,length_95%HPD={2.96269600e-01,4.83153700e-01}],
96[&prob=1.00000000e+00,prob_stddev=0.00000000e+00,prob_range={1.000
00000e+00,1.00000000e+00},prob(percent)="100",prob+-sd="100+-0"]:
3.120596e-01[&length_mean=3.11850081e-01,length_median=3.12059600e-0
1,length_95%HPD={2.25822100e-01,3.92939800e-01}])
[&prob=9.14381583e-01,prob_stddev=2.92257814e-03,prob_range={9.12315
008e-01,9.16448158e-01},prob(percent)="91",prob+-sd="91+-0"]:
8.786544e-02[&length_mean=9.05185599e-02,length_median=8.78654400e-0
2,length_95%HPD={2.62367100e-02,1.65996000e-01}],
(((97[&prob=1.00000000e+00,prob_stddev=0.00000000e+00,prob_range={1.
00000000e+00,1.00000000e+00},prob(percent)="100",prob+-sd="100+-0"]:
2.825904e-01[&length_mean=2.82733581e-01,length_median=2.82590400e-0
1,length_95%HPD={2.02181000e-01,3.74208300e-01}],
98[&prob=1.00000000e+00,prob_stddev=0.00000000e+00,prob_range={1.000
00000e+00,1.00000000e+00},prob(percent)="100",prob+-sd="100+-0"]:
2.024660e-01[&length_mean=2.04992314e-01,length_median=2.02466000e-0
1,length_95%HPD={1.21604600e-01,2.84452200e-01}])
[&prob=8.05519755e-01,prob_stddev=9.27682867e-02,prob_range={7.39922
670e-01,8.71116839e-01},prob(percent)="81",prob+-sd="81+-9"]:
1.061518e-01[&length_mean=1.07646374e-01,length_median=1.06151800e-0
```

1,length_95%HPD={4.05797100e-02,1.72791900e-01}],
((((99[&prob=1.00000000e+00,prob_stddev=0.00000000e+00,prob_range={1
.00000000e+00,1.00000000e+00},prob(percent)="100",prob+-
sd="100+-0"]:
5.510631e-02[&length_mean=5.65526970e-02,length_median=5.51063100e-0
2,length_95%HPD={2.75680300e-02,8.83886600e-02}],
100[&prob=1.00000000e+00,prob_stddev=0.00000000e+00,prob_range={1.00
000000e+00,1.00000000e+00},prob(percent)="100",prob+-sd="100+-0"]:
6.418492e-02[&length_mean=6.58122295e-02,length_median=6.41849200e-0
2,length_95%HPD={3.12842100e-02,1.00486000e-01}])
[&prob=9.89711568e-01,prob_stddev=1.06532687e-02,prob_range={9.82178
570e-01,9.97244567e-01},prob(percent)="99",prob+-sd="99+-1"]:
3.797844e-02[&length_mean=3.97988018e-02,length_median=3.79784400e-0
2,length_95%HPD={6.93596000e-03,7.28713300e-02}],
101[&prob=1.00000000e+00,prob_stddev=0.00000000e+00,prob_range={1.00
000000e+00,1.00000000e+00},prob(percent)="100",prob+-sd="100+-0"]:
9.564321e-02[&length_mean=9.69572543e-02,length_median=9.56432100e-0
2,length_95%HPD={5.55476200e-02,1.41641200e-01}])
[&prob=9.99755566e-01,prob_stddev=3.14255714e-05,prob_range={9.99733
345e-01,9.99777788e-01},prob(percent)="100",prob+-sd="100+-0"]:
1.191894e-01[&length_mean=1.20163717e-01,length_median=1.19189400e-0
1,length_95%HPD={4.53523200e-02,1.92833300e-01}],
102[&prob=1.00000000e+00,prob_stddev=0.00000000e+00,prob_range={1.00
000000e+00,1.00000000e+00},prob(percent)="100",prob+-sd="100+-0"]:
2.020883e-01[&length_mean=2.05802225e-01,length_median=2.02088300e-0
1,length_95%HPD={8.64142600e-02,3.27194600e-01}])
[&prob=9.99511133e-01,prob_stddev=3.14255714e-04,prob_range={9.99288
920e-01,9.99733345e-01},prob(percent)="100",prob+-sd="100+-0"]:
1.055335e-01[&length_mean=1.08005521e-01,length_median=1.05533500e-0
1,length_95%HPD={3.83347800e-02,1.86060900e-01}],
((103[&prob=1.00000000e+00,prob_stddev=0.00000000e+00,prob_range={1.
00000000e+00,1.00000000e+00},prob(percent)="100",prob+-sd="100+-0"]:
9.605767e-02[&length_mean=9.83292800e-02,length_median=9.60576700e-0
2,length_95%HPD={5.62916000e-02,1.44504500e-01}],
104[&prob=1.00000000e+00,prob_stddev=0.00000000e+00,prob_range={1.00
000000e+00,1.00000000e+00},prob(percent)="100",prob+-sd="100+-0"]:
1.332236e-01[&length_mean=1.35836099e-01,length_median=1.33223600e-0
1,length_95%HPD={8.74972700e-02,1.87750600e-01}])
[&prob=1.00000000e+00,prob_stddev=0.00000000e+00,prob_range={1.00000
000e+00,1.00000000e+00},prob(percent)="100",prob+-sd="100+-0"]:
5.802240e-02[&length_mean=6.04036752e-02,length_median=5.80224000e-0
2,length_95%HPD={2.30942800e-02,1.01219100e-01}],
105[&prob=1.00000000e+00,prob_stddev=0.00000000e+00,prob_range={1.00
000000e+00,1.00000000e+00},prob(percent)="100",prob+-sd="100+-0"]:
1.127114e-01[&length_mean=1.14530792e-01,length_median=1.12711400e-0
1,length_95%HPD={6.14798900e-02,1.61896800e-01}],
106[&prob=1.00000000e+00,prob_stddev=0.00000000e+00,prob_range={1.00
000000e+00,1.00000000e+00},prob(percent)="100",prob+-sd="100+-0"]:
1.686782e-01[&length_mean=1.69736393e-01,length_median=1.68678200e-0
1,length_95%HPD={1.13292900e-01,2.24955700e-01}])
[&prob=1.00000000e+00,prob_stddev=0.00000000e+00,prob_range={1.00000
000e+00,1.00000000e+00},prob(percent)="100",prob+-sd="100+-0"]:
2.341521e-01[&length_mean=2.36499329e-01,length_median=2.34152100e-0
1,length_95%HPD={1.65127900e-01,3.17751000e-01}])

[&prob=9.98622283e-01,prob_stddev=1.94838542e-03,prob_range={9.97244
567e-01,1.00000000e+00},prob(percent)="100",prob+-sd="100+-0"]:
9.172582e-02[&length_mean=9.38448165e-02,length_median=9.17258200e-0
2,length_95%HPD={3.75289600e-02,1.54707500e-01}])
[&prob=9.77934314e-01,prob_stddev=9.45909698e-03,prob_range={9.71245
722e-01,9.84622906e-01},prob(percent)="98",prob+-sd="98+-1"]:
8.240001e-02[&length_mean=8.53266468e-02,length_median=8.24000100e-0
2,length_95%HPD={2.49582500e-02,1.48696800e-01}],
(((((112[&prob=1.00000000e+00,prob_stddev=0.00000000e+00,prob_range=
{1.00000000e+00,1.00000000e+00},prob(percent)="100",prob+-
sd="100+-0"]:
2.552837e-01[&length_mean=2.56494523e-01,length_median=2.55283700e-0
1,length_95%HPD={1.81904600e-01,3.32476100e-01}],
(113[&prob=1.00000000e+00,prob_stddev=0.00000000e+00,prob_range={1.0
0000000e+00,1.00000000e+00},prob(percent)="100",prob+-sd="100+-0"]:
7.805827e-02[&length_mean=7.90774214e-02,length_median=7.80582700e-0
2,length_95%HPD={4.23079500e-02,1.15920000e-01}],
114[&prob=1.00000000e+00,prob_stddev=0.00000000e+00,prob_range={1.00
000000e+00,1.00000000e+00},prob(percent)="100",prob+-sd="100+-0"]:
4.089617e-02[&length_mean=4.26507686e-02,length_median=4.08961700e-0
2,length_95%HPD={1.43185500e-02,7.44245100e-02}])
[&prob=1.00000000e+00,prob_stddev=0.00000000e+00,prob_range={1.00000
000e+00,1.00000000e+00},prob(percent)="100",prob+-sd="100+-0"]:
1.265247e-01[&length_mean=1.27900886e-01,length_median=1.26524700e-0
1,length_95%HPD={7.24877700e-02,1.87774300e-01}])
[&prob=1.00000000e+00,prob_stddev=0.00000000e+00,prob_range={1.00000
000e+00,1.00000000e+00},prob(percent)="100",prob+-sd="100+-0"]:
1.488179e-01[&length_mean=1.49586173e-01,length_median=1.48817900e-0
1,length_95%HPD={8.51727200e-02,2.12604900e-01}],
((115[&prob=1.00000000e+00,prob_stddev=0.00000000e+00,prob_range={1.
00000000e+00,1.00000000e+00},prob(percent)="100",prob+-sd="100+-0"]:
1.688939e-01[&length_mean=1.70671113e-01,length_median=1.68893900e-0
1,length_95%HPD={1.12810200e-01,2.34325900e-01}],
(116[&prob=1.00000000e+00,prob_stddev=0.00000000e+00,prob_range={1.0
0000000e+00,1.00000000e+00},prob(percent)="100",prob+-sd="100+-0"]:
9.851028e-02[&length_mean=1.00689698e-01,length_median=9.85102800e-0
2,length_95%HPD={5.67396900e-02,1.49151500e-01}],
117[&prob=1.00000000e+00,prob_stddev=0.00000000e+00,prob_range={1.00
000000e+00,1.00000000e+00},prob(percent)="100",prob+-sd="100+-0"]:
9.367439e-02[&length_mean=9.53879268e-02,length_median=9.36743900e-0
2,length_95%HPD={4.78532000e-02,1.42100000e-01}])
[&prob=1.00000000e+00,prob_stddev=0.00000000e+00,prob_range={1.00000
000e+00,1.00000000e+00},prob(percent)="100",prob+-sd="100+-0"]:
1.024712e-01[&length_mean=1.03772089e-01,length_median=1.02471200e-0
1,length_95%HPD={5.16025300e-02,1.52913300e-01}])
[&prob=9.88844940e-01,prob_stddev=3.14255714e-04,prob_range={9.88622
728e-01,9.89067153e-01},prob(percent)="99",prob+-sd="99+-0"]:
6.087831e-02[&length_mean=6.30179375e-02,length_median=6.08783100e-0
2,length_95%HPD={2.32328000e-02,1.05054600e-01}],
118[&prob=1.00000000e+00,prob_stddev=0.00000000e+00,prob_range={1.00
000000e+00,1.00000000e+00},prob(percent)="100",prob+-sd="100+-0"]:
1.487531e-01[&length_mean=1.49285020e-01,length_median=1.48753100e-0
1,length_95%HPD={9.30665900e-02,2.03463100e-01}])
[&prob=1.00000000e+00,prob_stddev=0.00000000e+00,prob_range={1.00000

000e+00,1.00000000e+00},prob(percent)="100",prob+-sd="100+-0"]:
9.534131e-02[&length_mean=9.80337913e-02,length_median=9.53413100e-0
2,length_95%HPD={4.57812000e-02,1.57465400e-01}])
[&prob=9.40091551e-01,prob_stddev=6.72507227e-03,prob_range={9.35336
207e-01,9.44846896e-01},prob(percent)="94",prob+-sd="94+-1"]:
6.717968e-02[&length_mean=6.94309117e-02,length_median=6.71796800e-0
2,length_95%HPD={1.99081000e-02,1.21067600e-01}],
(119[&prob=1.00000000e+00,prob_stddev=0.00000000e+00,prob_range={1.0
0000000e+00,1.00000000e+00},prob(percent)="100",prob+-sd="100+-0"]:
1.769038e-01[&length_mean=1.76850919e-01,length_median=1.76903800e-0
1,length_95%HPD={1.12349300e-01,2.44281400e-01}],
120[&prob=1.00000000e+00,prob_stddev=0.00000000e+00,prob_range={1.00
000000e+00,1.00000000e+00},prob(percent)="100",prob+-sd="100+-0"]:
1.938869e-01[&length_mean=1.94979370e-01,length_median=1.93886900e-0
1,length_95%HPD={1.33818900e-01,2.63354500e-01}])
[&prob=1.00000000e+00,prob_stddev=0.00000000e+00,prob_range={1.00000
000e+00,1.00000000e+00},prob(percent)="100",prob+-sd="100+-0"]:
1.635874e-01[&length_mean=1.64745983e-01,length_median=1.63587400e-0
1,length_95%HPD={9.62146600e-02,2.36375800e-01}])
[&prob=9.36336163e-01,prob_stddev=2.05837492e-02,prob_range={9.21781
254e-01,9.50891072e-01},prob(percent)="94",prob+-sd="94+-2"]:
6.274337e-02[&length_mean=6.56772547e-02,length_median=6.27433700e-0
2,length_95%HPD={1.38107300e-02,1.22741300e-01}],
(121[&prob=1.00000000e+00,prob_stddev=0.00000000e+00,prob_range={1.0
0000000e+00,1.00000000e+00},prob(percent)="100",prob+-sd="100+-0"]:
2.858511e-01[&length_mean=2.87150192e-01,length_median=2.85851100e-0
1,length_95%HPD={2.07826300e-01,3.67509900e-01}],
(122[&prob=1.00000000e+00,prob_stddev=0.00000000e+00,prob_range={1.0
0000000e+00,1.00000000e+00},prob(percent)="100",prob+-sd="100+-0"]:
9.753312e-02[&length_mean=1.00575664e-01,length_median=9.75331200e-0
2,length_95%HPD={5.39870500e-02,1.56323800e-01}],
123[&prob=1.00000000e+00,prob_stddev=0.00000000e+00,prob_range={1.00
000000e+00,1.00000000e+00},prob(percent)="100",prob+-sd="100+-0"]:
8.772002e-02[&length_mean=8.98554155e-02,length_median=8.77200200e-0
2,length_95%HPD={4.06899200e-02,1.40815800e-01}])
[&prob=1.00000000e+00,prob_stddev=0.00000000e+00,prob_range={1.00000
000e+00,1.00000000e+00},prob(percent)="100",prob+-sd="100+-0"]:
1.768076e-01[&length_mean=1.78104369e-01,length_median=1.76807600e-0
1,length_95%HPD={1.08639900e-01,2.50126000e-01}])
[&prob=1.00000000e+00,prob_stddev=0.00000000e+00,prob_range={1.00000
000e+00,1.00000000e+00},prob(percent)="100",prob+-sd="100+-0"]:
1.835596e-01[&length_mean=1.86425655e-01,length_median=1.83559600e-0
1,length_95%HPD={1.04835400e-01,2.60980800e-01}])
[&prob=1.00000000e+00,prob_stddev=0.00000000e+00,prob_range={1.00000
000e+00,1.00000000e+00},prob(percent)="100",prob+-sd="100+-0"]:
1.720677e-01[&length_mean=1.74946447e-01,length_median=1.72067700e-0
1,length_95%HPD={9.42709000e-02,2.62346700e-01}],
126[&prob=1.00000000e+00,prob_stddev=0.00000000e+00,prob_range={1.00
000000e+00,1.00000000e+00},prob(percent)="100",prob+-sd="100+-0"]:
4.953113e-01[&length_mean=4.97931174e-01,length_median=4.95311300e-0
1,length_95%HPD={3.89347900e-01,6.17911500e-01}])
[&prob=8.38962713e-01,prob_stddev=9.55023114e-02,prob_range={7.71432
381e-01,9.06493045e-01},prob(percent)="84",prob+-sd="84+-10"]:
8.873377e-02[&length_mean=9.11143994e-02,length_median=8.87337700e-0

2,length_95%HPD={3.45501600e-02,1.52310000e-01}])
[&prob=7.61610595e-01,prob_stddev=1.41855029e-01,prob_range={6.61303
942e-01,8.61917248e-01},prob(percent)="76",prob+-sd="76+-14"]:
8.978488e-02[&length_mean=9.18324949e-02,length_median=8.97848800e-0
2,length_95%HPD={3.38821200e-02,1.51085700e-01}],
((((129[&prob=1.00000000e+00,prob_stddev=0.00000000e+00,prob_range={
1.00000000e+00,1.00000000e+00},prob(percent)="100",prob+-
sd="100+-0"]:
1.550448e-01[&length_mean=1.56888965e-01,length_median=1.55044800e-0
1,length_95%HPD={9.71704900e-02,2.20797700e-01}],
130[&prob=1.00000000e+00,prob_stddev=0.00000000e+00,prob_range={1.00
000000e+00,1.00000000e+00},prob(percent)="100",prob+-sd="100+-0"]:
1.446003e-01[&length_mean=1.45968558e-01,length_median=1.44600300e-0
1,length_95%HPD={9.29324000e-02,2.07234200e-01}])
[&prob=8.64294920e-01,prob_stddev=2.53604361e-02,prob_range={8.46362
384e-01,8.82227457e-01},prob(percent)="86",prob+-sd="86+-3"]:
8.956765e-02[&length_mean=9.12661412e-02,length_median=8.95676500e-0
2,length_95%HPD={2.59029800e-02,1.62546500e-01}],
132[&prob=1.00000000e+00,prob_stddev=0.00000000e+00,prob_range={1.00
000000e+00,1.00000000e+00},prob(percent)="100",prob+-sd="100+-0"]:
3.553537e-01[&length_mean=3.57730023e-01,length_median=3.55353700e-0
1,length_95%HPD={2.59454400e-01,4.71757500e-01}])
[&prob=9.93778054e-01,prob_stddev=6.91362570e-04,prob_range={9.93289
187e-01,9.94266921e-01},prob(percent)="99",prob+-sd="99+-0"]:
1.570836e-01[&length_mean=1.65744800e-01,length_median=1.57083600e-0
1,length_95%HPD={5.26609200e-02,3.06939700e-01}],
131[&prob=1.00000000e+00,prob_stddev=0.00000000e+00,prob_range={1.00
000000e+00,1.00000000e+00},prob(percent)="100",prob+-sd="100+-0"]:
2.685288e-01[&length_mean=2.89813833e-01,length_median=2.68528800e-0
1,length_95%HPD={1.21914600e-01,5.63496600e-01}])
[&prob=9.25736634e-01,prob_stddev=1.05024259e-01,prob_range={8.51473
268e-01,1.00000000e+00},prob(percent)="93",prob+-sd="93+-11"]:
2.197491e-01[&length_mean=2.21227628e-01,length_median=2.19749100e-0
1,length_95%HPD={1.17709100e-01,3.31027600e-01}],
((((133[&prob=1.00000000e+00,prob_stddev=0.00000000e+00,prob_range={
1.00000000e+00,1.00000000e+00},prob(percent)="100",prob+-
sd="100+-0"]:
3.387439e-01[&length_mean=3.41096976e-01,length_median=3.38743900e-0
1,length_95%HPD={2.34376700e-01,4.45386200e-01}],
134[&prob=1.00000000e+00,prob_stddev=0.00000000e+00,prob_range={1.00
000000e+00,1.00000000e+00},prob(percent)="100",prob+-sd="100+-0"]:
4.199044e-01[&length_mean=4.21890157e-01,length_median=4.19904400e-0
1,length_95%HPD={3.11363300e-01,5.31579300e-01}])
[&prob=9.33069641e-01,prob_stddev=1.09989500e-02,prob_range={9.25292
209e-01,9.40847073e-01},prob(percent)="93",prob+-sd="93+-1"]:
1.415173e-01[&length_mean=1.43999871e-01,length_median=1.41517300e-0
1,length_95%HPD={4.00516700e-02,2.48043500e-01}],
137[&prob=1.00000000e+00,prob_stddev=0.00000000e+00,prob_range={1.00
000000e+00,1.00000000e+00},prob(percent)="100",prob+-sd="100+-0"]:
4.727509e-01[&length_mean=4.78455704e-01,length_median=4.72750900e-0
1,length_95%HPD={3.37042700e-01,6.36602500e-01}])
[&prob=5.39131594e-01,prob_stddev=1.18788660e-02,prob_range={5.30731
967e-01,5.47531221e-01},prob(percent)="54",prob+-sd="54+-1"]:
1.146667e-01[&length_mean=1.20174667e-01,length_median=1.14666700e-0

1,length_95%HPD={2.94448900e-02,2.27816200e-01}],
136[&prob=1.00000000e+00,prob_stddev=0.00000000e+00,prob_range={1.00
000000e+00,1.00000000e+00},prob(percent)="100",prob+-sd="100+-0"]:
5.831655e-01[&length_mean=5.84441915e-01,length_median=5.83165500e-0
1,length_95%HPD={4.43581600e-01,7.36760600e-01}],
140[&prob=1.00000000e+00,prob_stddev=0.00000000e+00,prob_range={1.00
000000e+00,1.00000000e+00},prob(percent)="100",prob+-sd="100+-0"]:
5.784719e-01[&length_mean=5.79970893e-01,length_median=5.78471900e-0
1,length_95%HPD={4.30967900e-01,7.32651800e-01}])
[&prob=9.62046131e-01,prob_stddev=7.98209513e-03,prob_range={9.56401
938e-01,9.67690325e-01},prob(percent)="96",prob+-sd="96+-1"]:
1.160758e-01[&length_mean=1.20204541e-01,length_median=1.16075800e-0
1,length_95%HPD={3.55470900e-02,2.10965000e-01}],
((135[&prob=1.00000000e+00,prob_stddev=0.00000000e+00,prob_range={1.
00000000e+00,1.00000000e+00},prob(percent)="100",prob+-sd="100+-0"]:
5.155708e-01[&length_mean=5.15632051e-01,length_median=5.15570800e-0
1,length_95%HPD={3.91124500e-01,6.40601100e-01}],
(138[&prob=1.00000000e+00,prob_stddev=0.00000000e+00,prob_range={1.0
0000000e+00,1.00000000e+00},prob(percent)="100",prob+-sd="100+-0"]:
3.908839e-01[&length_mean=3.93396171e-01,length_median=3.90883900e-0
1,length_95%HPD={2.76988000e-01,5.16234000e-01}],
139[&prob=1.00000000e+00,prob_stddev=0.00000000e+00,prob_range={1.00
000000e+00,1.00000000e+00},prob(percent)="100",prob+-sd="100+-0"]:
3.271692e-01[&length_mean=3.31621253e-01,length_median=3.27169200e-0
1,length_95%HPD={2.14569800e-01,4.40003800e-01}])
[&prob=1.00000000e+00,prob_stddev=0.00000000e+00,prob_range={1.00000
000e+00,1.00000000e+00},prob(percent)="100",prob+-sd="100+-0"]:
2.606259e-01[&length_mean=2.62895269e-01,length_median=2.60625900e-0
1,length_95%HPD={1.51519900e-01,3.74205700e-01}])
[&prob=8.93826941e-01,prob_stddev=5.27949599e-03,prob_range={8.90093
774e-01,8.97560108e-01},prob(percent)="89",prob+-sd="89+-1"]:
9.591581e-02[&length_mean=9.94048614e-02,length_median=9.59158100e-0
2,length_95%HPD={1.75592200e-02,1.83172900e-01}],
(141[&prob=1.00000000e+00,prob_stddev=0.00000000e+00,prob_range={1.0
0000000e+00,1.00000000e+00},prob(percent)="100",prob+-sd="100+-0"]:
5.486526e-01[&length_mean=5.51254934e-01,length_median=5.48652600e-0
1,length_95%HPD={4.09356100e-01,6.95292200e-01}],
142[&prob=1.00000000e+00,prob_stddev=0.00000000e+00,prob_range={1.00
000000e+00,1.00000000e+00},prob(percent)="100",prob+-sd="100+-0"]:
5.224234e-01[&length_mean=5.24563311e-01,length_median=5.22423400e-0
1,length_95%HPD={3.93887300e-01,6.58469300e-01}])
[&prob=1.00000000e+00,prob_stddev=0.00000000e+00,prob_range={1.00000
000e+00,1.00000000e+00},prob(percent)="100",prob+-sd="100+-0"]:
2.526310e-01[&length_mean=2.55042100e-01,length_median=2.52631000e-0
1,length_95%HPD={1.36597900e-01,3.65721200e-01}])
[&prob=9.99955558e-01,prob_stddev=6.28511427e-05,prob_range={9.99911
115e-01,1.00000000e+00},prob(percent)="100",prob+-sd="100+-0"]:
1.543718e-01[&length_mean=1.57021072e-01,length_median=1.54371800e-0
1,length_95%HPD={7.16889100e-02,2.44749000e-01}])
[&prob=7.12768321e-01,prob_stddev=3.16141248e-02,prob_range={6.90413
759e-01,7.35122883e-01},prob(percent)="71",prob+-sd="71+-3"]:
1.167513e-01[&length_mean=1.20826096e-01,length_median=1.16751300e-0
1,length_95%HPD={2.93808900e-02,2.18462600e-01}])
[&prob=7.44478023e-01,prob_stddev=9.50623534e-02,prob_range={6.77258

788e-01,8.11697258e-01},prob(percent)="74",prob+-sd="74+-10"]:
1.209838e-01[&length_mean=1.21360612e-01,length_median=1.20983800e-0
1,length_95%HPD={3.64619000e-02,1.97331100e-01}])
[&prob=8.09719568e-01,prob_stddev=5.28892366e-02,prob_range={7.72321
230e-01,8.47117906e-01},prob(percent)="81",prob+-sd="81+-5"]:
1.424375e-01[&length_mean=1.42619523e-01,length_median=1.42437500e-0
1,length_95%HPD={8.68158300e-02,1.98513700e-01}])
[&prob=8.05675303e-01,prob_stddev=5.08780000e-02,prob_range={7.69699
124e-01,8.41651482e-01},prob(percent)="81",prob+-sd="81+-5"]:
5.348693e-02[&length_mean=5.52348478e-02,length_median=5.34869300e-0
2,length_95%HPD={1.81200800e-02,9.88633200e-02}])
[&prob=8.64028265e-01,prob_stddev=1.29693333e-01,prob_range={7.72321
230e-01,9.55735301e-01},prob(percent)="86",prob+-sd="86+-13"]:
5.409223e-02[&length_mean=5.66591702e-02,length_median=5.40922300e-0
2,length_95%HPD={2.02017700e-02,9.71829000e-02}])
[&prob=8.83338518e-01,prob_stddev=1.57002155e-01,prob_range={7.72321
230e-01,9.94355806e-01},prob(percent)="88",prob+-sd="88+-16"]:
6.684751e-02[&length_mean=6.90618995e-02,length_median=6.68475100e-0
2,length_95%HPD={2.70277600e-02,1.17888700e-01}])
[&prob=7.00079996e-01,prob_stddev=1.26990734e-01,prob_range={6.10283
987e-01,7.89876006e-01},prob(percent)="70",prob+-sd="70+-13"]:
3.758015e-02[&length_mean=3.93130189e-02,length_median=3.75801500e-0
2,length_95%HPD={8.57564700e-03,7.09305200e-02}],
40[&prob=1.00000000e+00,prob_stddev=0.00000000e+00,prob_range={1.000
00000e+00,1.00000000e+00},prob(percent)="100",prob+-sd="100+-0"]:
2.061611e-01[&length_mean=2.08043742e-01,length_median=2.06161100e-0
1,length_95%HPD={1.53392200e-01,2.72187900e-01}])
[&prob=5.32420781e-01,prob_stddev=2.22870152e-01,prob_range={3.74827
785e-01,6.90013777e-01},prob(percent)="53",prob+-sd="53+-22"]:
1.858538e-02[&length_mean=2.06620861e-02,length_median=1.85853800e-0
2,length_95%HPD={8.66883300e-04,4.43908900e-02}],
((35[&prob=1.00000000e+00,prob_stddev=0.00000000e+00,prob_range={1.0
0000000e+00,1.00000000e+00},prob(percent)="100",prob+-sd="100+-0"]:
1.634093e-02[&length_mean=1.77777826e-02,length_median=1.63409300e-0
2,length_95%HPD={2.50431000e-03,3.70164400e-02}],
36[&prob=1.00000000e+00,prob_stddev=0.00000000e+00,prob_range={1.000
00000e+00,1.00000000e+00},prob(percent)="100",prob+-sd="100+-0"]:
2.366430e-02[&length_mean=2.46399945e-02,length_median=2.36643000e-0
2,length_95%HPD={5.12111800e-03,4.60270200e-02}])
[&prob=9.99600018e-01,prob_stddev=2.51404571e-04,prob_range={9.99422
248e-01,9.99777788e-01},prob(percent)="100",prob+-sd="100+-0"]:
2.804035e-02[&length_mean=2.96244897e-02,length_median=2.80403500e-0
2,length_95%HPD={7.54329700e-03,5.45952200e-02}],
37[&prob=1.00000000e+00,prob_stddev=0.00000000e+00,prob_range={1.000
00000e+00,1.00000000e+00},prob(percent)="100",prob+-sd="100+-0"]:
8.236681e-02[&length_mean=8.36670237e-02,length_median=8.23668100e-0
2,length_95%HPD={4.78601400e-02,1.19442400e-01}])
[&prob=1.00000000e+00,prob_stddev=0.00000000e+00,prob_range={1.00000
000e+00,1.00000000e+00},prob(percent)="100",prob+-sd="100+-0"]:
1.108421e-01[&length_mean=1.12213663e-01,length_median=1.10842100e-0
1,length_95%HPD={6.23392600e-02,1.61832200e-01}])
[&prob=6.00773299e-01,prob_stddev=2.42605411e-01,prob_range={4.29225
368e-01,7.72321230e-01},prob(percent)="60",prob+-sd="60+-24"]:
5.622790e-02[&length_mean=5.76602324e-02,length_median=5.62279000e-0

2,length_95%HPD={2.01001500e-02,9.80396400e-02}])
[&prob=5.06399716e-01,prob_stddev=2.00777975e-01,prob_range={3.64428
248e-01,6.48371184e-01},prob(percent)="51",prob+-sd="51+-20"]:
6.579495e-02[&length_mean=6.73303522e-02,length_median=6.57949500e-0
2,length_95%HPD={1.85327600e-02,1.18452600e-01}]);
end;

Fig4A(Fig_S4)_RAxML_tree_file

((EPHA2_HUMAN:0.13511917991754582302,
(Drer_11069.6:0.10306284677983901243,Drer_44917.5:0.0544951125697187
9825):0.07251783546872618558):0.09812255862042730536,((CiEphe:
0.60406476961263788628,CiEphf:0.81310771435092388248):
0.13629583435476433362,((((((EPHA8_HUMAN:
0.16084186077738144016,Drer_41799.6:0.10447274922225698035):
0.08415735586889706854,
(Drer_121462.2:0.07858006550941677715,EPHA6_HUMAN:
0.02996076556105451913):0.09502902367082791124):
0.01857871163668832828,
((Drer_93563.5:0.01920050989630712676,EPHA7_HUMAN:
0.02750514998430245162):0.06533176225766322565,
(Drer_141023.1:0.29581659405038918109,EPHA10_HUMAN:
0.47071967005156101216):0.26273116619724162790):
0.03614576660871105657):0.01300044895875987656,
((Drer_21706.11:0.03035319884226391277,EPHA3_HUMAN:
0.01535388452881574113):0.05736734236014268823,(EPHA5_HUMAN:
0.05391135900783265017,Drer_75583.4:0.06918696100777278757):
0.03215760005450392367):0.03425175437098802733):
0.01269542741783683502,
(((Drer_30606.8:0.01221198722327879876,Drer_96552.3:0.05864330442672
624488):0.07286592612673196967,
((Drer_123962.1:0.02778469728192502208,Drer_3161.9:0.069128512448476
68941):0.02444129946865856301,EPHA4_HUMAN:0.01323892655100314050):
0.05083730079183428757):
0.01169292848264508536,Pmar_10282.1:0.26150842819276159945):
0.02917960467269092167):0.00960670209910431151,
(Pmar_2234.1:0.29285961564510426536,((((EPHB3_HUMAN:
0.03000584235323363116,
(Drer_40208.7:0.13747471293017377025,Drer_140419.1:0.005315987144686
67528):0.01136822688733303555):0.03617884031241816456,
((Drer_111570.2:0.02464294047603816235,EPHB1_HUMAN:
0.01671259362189384326):0.03409570092009633208,
((Drer_135357.1:0.03467079981119845083,Drer_88414.3:0.03879383867847
684131):0.04823254290791982590,EPHB4_HUMAN:0.05289495043004538433):
0.09081666302474264429):0.02116545802230653667):
0.02037579155009350620,((EPHB6_HUMAN:
0.56416531935323743774,Drer_113830.2:0.33796768860015247160):
0.23266177278514721394,
((Pmar_9675.1:0.18389670330075486016,Pmar_7951.1:0.05556154778881395
445):0.03467163798076338538,((Drer_43755.7:0.01157451016820631833,
(EPHB2_HUMAN:
0.01331883729798026723,Drer_112928.2:0.02579952216837557225):
0.00607834352250615981):
0.01682447447546659910,Pmar_5131.1:0.02152180399938409885):

0.03265822003949400698):0.00000093905322139818):
0.00806642140975447758):0.06722717440585496140,
(((BfEph2:0.21321042242171459047,BfEph1:0.03633618286220930554):
0.22379499127816532278,((Spur_027145:0.26384987306255142103,
((Tadh_12344:1.02564790296673824521,
((Hvul_18252:0.35784613352115074436,
((((Aele_129846:0.00000093905322139818,Nbij_1526:0.12389001723487846
895):0.02567724175987548194,Nbij_150435:0.08244370706867550080):
0.03371267296826002680,
(Clat_102964:0.02479779641752154901,Atet_45096:0.0392241971817254772
6):0.07228756648152835640):0.15006835079342625838,
(Clat_76838:0.06842299687141467635,Aele_55285:0.09302291417137835416
):0.11504902637268632493):0.04642755862415118284):
0.06166571057740600353,(((Hvul_37681:0.05744006010053999411,
(Hvul_13820:0.20479559992222304277,Hvul_31223:0.19679662609465903289
):0.01105814766360465988):0.06111979829263774339,
(Hvul_13823:0.13745035516601231129,Hvul_37741:0.71607428840557330041
):0.08055473373493989009):0.06614359019082965974,
((Nbij_35443:0.09692377652036762714,Clat_108627:0.08211035045659703657):0.01724553825322330119,Pphy_15204:0.04371180687676497895):
0.34794404294269587474):0.06256168678983722864):
0.18870158012027005157):0.13799049545680422413,
(((Ever_3065:0.62621026230944076829,
(((Pcar_328883:0.16309153659090824817,Adig_18762:0.20726943073567530
296):0.07210686630797308316,
(Nvec_40994:0.11060578770917618707,Apal_103658:0.1346669506230139812
3):0.07108239723219547668):0.09663678302272778187,
(((Ever_12042:0.41995249060795303286,
(Ever_2330:0.16786698189186863295,Ever_2431:0.38768179722573853363):
0.03837908614851621297):
0.08708457045970553756,Ever_14808:0.37731026792914629242):
0.14753260244690313829,
(Ever_3805:0.34605365611546490268,Ever_20101:0.55659045386892924689)
:0.12021579836531934293):0.01687577387539454868):
0.02740973625823706419):0.04028992294821680253,
((((((Apal_306157:0.01484187194200681063,
(Apal_306140:0.03542753911810254258,Apal_271591:0.249083356965007218
79):0.02071418705841697114):0.07079899049480727069,
(Hdig_16446:0.17721826824200426209,
(Btue_4512:0.24103096828892306425,Apal_306159:0.10196461045953021662
):0.01775110228774265733):0.01626763447109156116):
0.05143914749152556781,Apal_455194:0.31427731550705084729):
0.05800015764359350151,Nvec_33736:0.20467807851191649471):
0.06156615046769434163,
(Pcar_152315:0.00000093905322139818,Pcar_152322:0.08848180293711307987):0.31542540947221475722):0.07255252738432577120,
(Adig_03916:0.49480472408055548028,
((Adig_12429:0.02372495269415831470,Adig_15481:0.05262803673150824590):0.01175783292936936723,(Adig_12482:0.07888592331004574965,
(Adig_24585:0.01455233381634944527,Adig_04039:0.04078162835636015698
):0.00000093905322139818):0.01271641857860811298):
0.00000093905322139818):0.32507097074581087215):
0.10467853917918087947):0.06967666903786970400,
((((((Snux_6723:0.10151345796911721842,Rfib_47059:0.1269894092525419

0160):0.23688135267363916170,Avas_85009:0.47585086902803169773):
0.35991751992777470948,
((((Rfib_55118:0.09877584873871157745,Snux_99202:0.03959406802966016
631):0.15965111113196647774,Avas_63694:0.39429336562354067031):
0.24457492699602675912,(Avas_93988:0.19757029419726201969,
((Snux_109225:0.11492043009636355999,Rfib_49817:0.126562326688598680
85):0.14240190806880489993,Avas_85324:0.23463498366274096463):
0.08737585579549629611):0.12741192761513850384):
0.08185472063825820210,
(Avas_82832:0.24548081841419713833,Rfib_47064:0.28541934701206644887
):0.28903130625662332154):0.04680783192307374357):
0.39854406693311972276,(Cnul_9016:0.44696488547615431663,
(((Psub_10078:0.26878545185068947188,
(Kvar_222799:0.13261078779847590425,
(Cpro_79320.39:0.06344396005557295659,Lapi_154083:0.0815223252339354
3318):0.03496627938998618512):0.17907645798680826843):
0.15480716202416941463,
((Pfic_10202:0.17223629217408858993,Niph_54284:0.1440750989962541472
9):0.08482363933808451106,
(Xtes_54061:0.17516801764194087077,Aque_41463:0.22660685118125706294
):0.01256126859993178871):0.45281536143683703832):
0.17173948842174008300,Ifas_9729:0.35326634662685030275):
0.12147324130031585121):0.08400331387239108605):
0.14325392455082511245,
(((((Aspe_Peph_146412:0.96730480530259543848,Aspe_Peph_158325:1.1215
6767020779324362):0.20051080304204954574,
(Mbre_Peph_25247:0.79938189094480227581,Sros_Peph_EGD72856:0.5625718
4925037573286):0.30596179066198098129):
0.12861772385146108544,Mbre_Peph_26435:1.21880420430564107015):
0.19389297361703039524,
((Apar_Peph_165057:1.21877425997876454034,Sarc_Peph_10364T0:1.044646
29077426818249):0.59133582444608923101,
((Cowc_Peph_01676T0:0.77925136086353596898,Cowc_Peph_09852T0:0.55051
126022147234274):
0.58160451866716167402,Mvib_Peph_25039:1.10743554831031687513):
0.14786519563008787559):0.35552140214966493348):
0.14200961589948901675,(Vmul_37755:0.35781163828523715464,
(Mlei_35913a-PA:0.15250468702472885774,
(Patl_251869:0.70444878777456509944,Baby_41769:0.1945919761983281315
3):0.07357714589751165879):0.33803404672204095949):
0.45711808230640788020):0.17423284813523187697):
0.07342442556063336068,(Scoa_1400:0.69736751789752438047,
(Sros_Peph_EGD75115:1.04955304880594035843,Ocar_5890:0.5436014050458
5374721):0.04341234934602776485):0.11657159296518515568):
0.11144645254745993990):0.01265561859721402865):
0.21072913668470369397):0.07064936068244898060,
(((Tpol_63971:0.10167975486299027366,Ctel_198909:0.17864825662364766
656):0.00000093905322139818,
(Lgig_105485:0.07001785666668429442,Hpsi_102387:0.102715960207624759
65):0.00643625446391202314):0.04759694434777998057,
(Lith_18666:0.02100350271263345600,
(Lith_3827:0.00000093905322139818,Dpul_79285:0.10289806364009256023)
:0.00000093905322139818):0.15765698359832849840):
0.06786073544369240762):0.07579249508952484726):

0.08628656539561083139,(CiEpha:0.42673220729242117288,(CiEphd:
0.09063339250375151979,CiEphb:0.08707426749747305961):
0.06553932518516034089):0.08690235234237188300):
0.03141988780340625870):0.02343720825022724275):
0.01398222843713792587):0.02491144151613992538,
(Pmar_9793.1:0.10244777705582684690,
(Pmar_10202.1:0.02686931816819898566,Pmar_7260.1:0.01628325647173214
930):0.02665345194294551842):0.17030594199495704966):
0.04386146342843179335):0.03922343305867728724,EPHA1_HUMAN:
0.62555278478103770734):0.0;

Fig4B(Fig_S5)_IQtree_tree_file

(EPHA1_HUMAN:0.0954251550,EPHA1_MOUSE:0.0078625877,(((EPHA2_HUMAN:
0.0000022250,EPHA2_MOUSE:0.0612333395)100:0.2019744899,
(Drer_11069.6:0.3816053953,Drer_44917.5:0.3202153601)100:0.303749764
1)99:0.1280512761,((((EPHA3_HUMAN:
0.0382827123,Drer_21706.11:0.0987736469)100:0.1118835912,
(((((EPHA4_HUMAN:0.0125538619,
(Drer_123962.1:0.0361804808,Drer_3161.9:0.1254392978)100:0.050987958
0)100:0.0596199434,
(Drer_30606.8:0.0907017022,Drer_96552.3:0.1175330653)100:0.168816215
9)98:0.1050142374,(EPHA5_HUMAN:
0.0573154897,Drer_134983.1:0.1352294377)100:0.1438569125)73:0.042792
5248,
(((Pmar_2234.1:0.1059168401,Pmar_9310.1:0.1494193107)100:0.061127274
5,(((EPHB1_HUMAN:
0.0778364341,Drer_136066.1:0.0160181162)100:0.1063947871,
((EPHB2_HUMAN:0.0673564472,
(Drer_43755.7:0.0204574172,Drer_112928.2:0.0652744145)100:0.03854767
78)99:0.0860047206,Pmar_7951.1:0.1163388779)93:0.0299772153)93:0.029
1056440,((EPHB3_HUMAN:0.0753043927,
(Drer_140419.1:0.0587136880,Drer_40208.7:0.1377036847)93:0.045036960
9)100:0.1207960271,((((((CiEphb:0.1938236032,CiEphd:
0.1937449246)100:0.2086798845,(CiEpha:0.3895958614,CiEphf:
1.0889655300)49:0.1238849068)61:0.1312245889,
((((Baby_41769:0.4984359399,(Patl_251869:0.3403758720,
(Edun_188839:0.7582704485,Mlei_02521a-PA:
0.6030750633)88:0.3623656329)87:0.0717433480)94:0.3308285508,Cast_22
203:0.3335499444)94:0.1104344470,Vmul_37755:0.4672684447)100:0.87548
15700,
(((((Ccan_99557:0.8563190331,Ocar_909:0.6791053778)99:0.5605783853,
((((((Avas_56846:0.1006498191,Avas_56851:0.0673104286)100:0.0492394
886,Avas_56854:0.0673749025)100:0.1336879759,
(Avas_93988:0.0929209702,Avas_93993:0.1306972930)100:0.1382860210)10
0:0.2279341508,Snux_109225:0.4499735813)100:0.2555527932,
((Avas_85009:0.4525943474,
(Rfib_2729:0.2696383442,Snux_119736:0.1861767000)100:0.4312692254)94
:0.2362042141,
(Avas_63694:0.6047922582,Hpop_15474:0.9905111662)86:0.1187595644)72:
0.1510469855)88:0.3674104712,Avas_82832:0.4403851346)90:0.2688068615
,Rfib_47059:0.8703586028)100:0.7080437017)41:0.0961005925,
((((((((Cvar_1757:0.3756645916,
((Cvar_19694:0.0567215543,Cvar_19697:0.0000029840)100:0.0423892721,C

var_19695:0.0602975848)99:0.0366067031)81:0.0087901492,
(Cvar_18894:0.2477636908,Cvar_19692:0.1155525657)80:0.0193148537)99:
0.2284645958,(Cpro_80288.25:0.2331508887,(Lapi_154083:0.2277925692,
(Kvar_222799:0.1926646097,Cele_48040:0.8608257651)98:0.1495618248)98
:0.0651322043)99:0.1442089934)100:0.3774947406,
(Ifas_26594:0.2351761417,Ifas_36548:0.2796972368)100:0.4898315136)69
:0.2305990905,
((Cele_137847:0.7039475481,Kvar_25216:0.3865723651)99:0.3000020193,L
api_88092:0.5656596616)100:0.3489307680)68:0.1317288290,
(Cnul_32701:0.1446118152,Cnul_32703:0.0581452528)100:1.4190847343)35
:0.1288698518,
(((((Niph_54283:0.3484632556,Pfic_36041:0.2807452278)100:0.295519751
0,
(Xtes_94992:0.0681507660,Xtes_94993:0.0962684988)100:0.2468520121)10
0:0.1740855586,
(((((Aque_03862:0.0422792073,Aque_37706:0.0851657595)68:0.0118840662
,
(Aque_41463:0.0259765559,Aque_16689:0.1572078533)98:0.0155252541)69:
0.0079082681,Aque_37708:0.0378043377)67:0.0073503176,
(Aque_32613:0.0448942742,Aque_32612:0.0576966083)71:0.0000023708)83:
0.0409497852,Aque_12969:0.0066531934)100:0.4556560692)100:0.18536465
96,Aque_37707:0.6235981142)100:0.5220753368,
((((Hamb_7684:0.3259331575,Aque_11657:0.2013008540)100:0.4347895132,
Niph_32084:0.6158923464)100:0.4707139487,Slac_1485:0.7077133828)74:0
.2569745733,(Cvar_27357:0.8095514528,((((Cpro_59066.22:0.3586446312,
(Cele_124391:0.3378243232,Kvar_4564:0.2878111176)100:0.1848444283)10
0:0.1424973848,Lapi_96494:0.4023771997)94:0.1866447545,Cpro_78852.34
:0.8324591963)70:0.0712886973,
(((((Cele_85965:0.4253115344,Kvar_2233:0.5279575340)69:0.1313076735,
Lapi_108357:0.4452131630)75:0.0566233455,
(Lapi_159044:0.2056317683,Cpro_75438.40:0.7225565139)86:0.1290266036
)83:0.0707859262,(Kvar_222743:0.4195563245,
(Psub_14382:0.5690096002,Psub_7666:0.5512919632)96:0.2368468266)86:0
.1369373832)87:0.1260878896,
(Kvar_137296:0.4094416214,Lapi_126388:0.3100469790)98:0.1748420516)5
1:0.0969966649)87:0.2275925468)100:0.4394100158)82:0.1349228814)59:0
.
0883941594)51:0.1186749767,Pper_114995:1.6328954463)91:0.1692603916)
44:0.1299221590,
((Tadh_53296:1.0671264309,Tadh_56933:1.2246148628)100:0.5667625002,
(Aele_22617:0.6762176064,Clat_102964:0.9367980667)100:0.8736173330)7
9:0.1317501984)68:0.2178754486,((((Adig_13891:0.8696598153,
(Apal_405536:0.7659188231,Nvec_40993:0.7294028492)98:0.2548841370)90
:0.0789135830,((((((Adig_00805:0.1029655417,
(Adig_12429:0.0917264796,(Adig_15481:0.1830638158,
(Adig_03916:0.1249404646,Adig_20930:0.0584431831)100:0.1944516137)94
:
0.1266574280)91:0.0557269484)85:0.0399725566,Adig_04039:0.1095537176
)76:0.0244534155,Adig_12482:0.1035128086)47:0.0181998018,Adig_18229:
0.1136540425)100:1.0700084617,(((((((Apal_25755:0.3551409395,
(Apal_215216:0.0437839247,
(Apal_215213:0.0000029840,Apal_215214:0.1487311156)100:0.1351288489)
95:0.0763035213)85:0.0328802595,
((Apal_215220:0.0445526916,Apal_215207:0.1005354712)100:0.1222256768

```
,Apal_549316:0.2097373368)75:0.0456656461)66:0.0228565541,Apal_54931
7:0.1281647238)74:0.0196567641,
(Apal_215219:0.0501185453,Apal_215211:0.1230500345)100:0.1602315689)
71:0.0173569112,
(Apal_215204:0.1849183176,Apal_307048:0.8541038399)70:0.0801228284)9
7:0.3690650915,Hdig_11359:0.4725887070)100:0.1928942713,
((Btue_32401:0.5780371171,
(Nvec_39123:0.0219287889,Nvec_39124:0.1008400276)100:0.8146523761)94
:0.0841774823,
(Apal_612045:0.4826326293,Apal_455194:0.5534063215)99:0.3818753543)9
8:0.2016124959)99:0.2641549788)86:0.1401043771,Pcar_315635:1.0157884
975)99:0.3132836980)89:0.1936064640,
(Adig_13888:0.1683422435,Adig_18762:0.1522741794)100:1.1370643748)87
:0.0342164056,(Aspe_Peph_158325:1.9575941879,
(Cowc_Peph_08089T0:1.3025204495,
(Mbre_Peph_9190:0.0372807612,Mbre_Peph_26435:0.0590941862)100:1.5404
573189)96:0.1995792224)98:0.5198404412)82:0.1181614521)75:0.21151111
82)92:0.4632132947)43:0.0577811078,
(((BfEph1:0.1460261821,BfEph2:0.1081705331)100:0.2477681863,
(Skow_11098:0.4507902576,Spur_027145:0.6134731844)100:0.2217423291)9
3:0.0455829218,
(((((Dpul_79285:0.0000027776,Lith_3827:0.0767480423)76:0.0460911494,
Lith_21051:0.0891869791)100:0.3105379235,Hpsi_234025:0.9608338403)70
:0.2587972947,
(Ctel_198909:0.3923318435,Tpol_7305:0.4004365427)98:0.1403543839)42:
0.0532729037,Lgig_105485:0.5273067641)95:0.3721338514)45:0.085297675
2)93:0.0999765327,(CiEphc:0.3844738178,CiEphe:
0.5840823082)99:0.3516537316)49:0.0522381899,((EPHB4_HUMAN:
0.0562251291,EPHB4_MOUSE:0.0275626669)100:0.3391448320,
(Drer_88414.3:0.1664567974,Drer_135357.1:0.1689802783)100:0.09752617
48)100:0.3078412703)90:0.0671797888,((EPHB6_HUMAN:
0.0323240881,EPHB6_MOUSE:
0.0395841435)100:0.5820632056,Drer_113830.2:0.4019277228)100:0.44697
35158)95:0.0834194045)93:0.0780376159)100:0.2846649988)95:0.03946392
96,((EPHA7_HUMAN:
0.0169243441,Drer_93563.5:0.0878875694)100:0.0861598105,
(EPHA10_HUMAN:
0.1542960289,Drer_99872.3:0.1419420160)100:0.1396151380)95:0.0748435
567)83:0.0271393401)68:0.0384209419,
((Pmar_9793.1:0.0230960954,Pmar_131.1:0.4758192574)100:0.2348893504,
(Pmar_7260.1:0.1266342412,Pmar_10202.1:0.1195331072)100:0.2931744804
)97:0.1457521199)72:0.0588487420)69:0.0543560104,(EPHA6_HUMAN:
0.0456841327,Drer_121462.2:0.1644686847)100:0.1369029867)88:0.081634
4571,(EPHA8_HUMAN:
0.1579270390,Drer_41799.6:0.1054645687)100:0.1257166818)100:0.311708
4447)100:0.4500306492);
```

Fig4B(Fig_S5)_Mrbayes_tree_file

```
#NEXUS
[ID: 3328098275]
begin taxa;
        dimensions ntax=174;
        taxlabels
```

EPHA1_HUMAN
EPHA1_MOUSE
EPHA2_HUMAN
EPHA2_MOUSE
Drer_11069_6
Drer_44917_5
EPHA3_HUMAN
Drer_21706_11
EPHA6_HUMAN
Drer_121462_2
EPHA4_HUMAN
Drer_123962_1
Drer_3161_9
Pmar_2234_1
Pmar_9310_1
EPHA7_HUMAN
Drer_93563_5
Drer_30606_8
Drer_96552_3
EPHA5_HUMAN
Drer_134983_1
EPHA10_HUMAN
Drer_99872_3
EPHA8_HUMAN
Drer_41799_6
Pmar_9793_1
EPHB1_HUMAN
Drer_136066_1
EPHB2_HUMAN
Drer_43755_7
Drer_112928_2
Pmar_7951_1
EPHB3_HUMAN
Drer_140419_1
Drer_40208_7
Pmar_7260_1
Pmar_10202_1
Pmar_131_1
CiEphb
CiEphd
BfEph1
BfEph2
EPHB4_HUMAN
EPHB4_MOUSE
Drer_88414_3
Drer_135357_1
CiEpha
Skow_11098
Dpul_79285
Lith_3827
Lith_21051
Ctel_198909
Tpol_7305
Lgig_105485

Spur_027145
CiEphc
CiEphe
EPHB6_HUMAN
EPHB6_MOUSE
Drer_113830_2
Hpsi_234025
CiEphf
Baby_41769
Patl_251869
Cast_22203
Vmul_37755
Edun_188839
Mlei_02521a_PA
Ccan_99557
Ocar_909
Adig_13891
Adig_00805
Adig_04039
Adig_12429
Adig_12482
Adig_18229
Adig_15481
Adig_03916
Adig_20930
Apal_405536
Nvec_40993
Adig_13888
Adig_18762
Apal_25755
Apal_549317
Apal_215216
Apal_215213
Apal_215214
Apal_215219
Apal_215211
Apal_215204
Apal_215220
Apal_215207
Apal_549316
Apal_307048
Hdig_11359
Btue_32401
Apal_612045
Apal_455194
Nvec_39123
Nvec_39124
Pcar_315635
Tadh_53296
Cvar_1757
Cvar_18894
Cvar_19692
Cvar_19694
Cvar_19697

Cvar_19695
Cpro_80288_25
Lapi_154083
Kvar_222799
Cele_48040
Ifas_26594
Ifas_36548
Niph_54283
Pfic_36041
Xtes_94992
Xtes_94993
Aque_03862
Aque_12969
Aque_32613
Aque_32612
Aque_37708
Aque_41463
Aque_37706
Aque_16689
Aque_37707
Hamb_7684
Aque_11657
Niph_32084
Slac_1485
Cele_137847
Kvar_25216
Lapi_88092
Cvar_27357
Cpro_59066_22
Cele_124391
Kvar_4564
Lapi_96494
Cele_85965
Kvar_2233
Lapi_108357
Lapi_159044
Kvar_222743
Kvar_137296
Lapi_126388
Cpro_75438_40
Cpro_78852_34
Psub_14382
Psub_7666
Avas_56846
Avas_56854
Avas_56851
Avas_93988
Avas_93993
Snux_109225
Avas_85009
Rfib_2729
Snux_119736
Avas_82832
Rfib_47059

```
                Avas_63694
                Hpop_15474
                Cnul_32701
                Cnul_32703
                Pper_114995
                Tadh_56933
                Aele_22617
                Clat_102964
                Aspe_Peph_158325
                Cowc_Peph_08089T0
                Mbre_Peph_9190
                Mbre_Peph_26435
                ;
end;
begin trees;
        translate
                1       EPHA1_HUMAN,
                2       EPHA1_MOUSE,
                3       EPHA2_HUMAN,
                4       EPHA2_MOUSE,
                5       Drer_11069_6,
                6       Drer_44917_5,
                7       EPHA3_HUMAN,
                8       Drer_21706_11,
                9       EPHA6_HUMAN,
                10      Drer_121462_2,
                11      EPHA4_HUMAN,
                12      Drer_123962_1,
                13      Drer_3161_9,
                14      Pmar_2234_1,
                15      Pmar_9310_1,
                16      EPHA7_HUMAN,
                17      Drer_93563_5,
                18      Drer_30606_8,
                19      Drer_96552_3,
                20      EPHA5_HUMAN,
                21      Drer_134983_1,
                22      EPHA10_HUMAN,
                23      Drer_99872_3,
                24      EPHA8_HUMAN,
                25      Drer_41799_6,
                26      Pmar_9793_1,
                27      EPHB1_HUMAN,
                28      Drer_136066_1,
                29      EPHB2_HUMAN,
                30      Drer_43755_7,
                31      Drer_112928_2,
                32      Pmar_7951_1,
                33      EPHB3_HUMAN,
                34      Drer_140419_1,
                35      Drer_40208_7,
                36      Pmar_7260_1,
                37      Pmar_10202_1,
                38      Pmar_131_1,
```

39 CiEphb,
40 CiEphd,
41 BfEph1,
42 BfEph2,
43 EPHB4_HUMAN,
44 EPHB4_MOUSE,
45 Drer_88414_3,
46 Drer_135357_1,
47 CiEpha,
48 Skow_11098,
49 Dpul_79285,
50 Lith_3827,
51 Lith_21051,
52 Ctel_198909,
53 Tpol_7305,
54 Lgig_105485,
55 Spur_027145,
56 CiEphc,
57 CiEphe,
58 EPHB6_HUMAN,
59 EPHB6_MOUSE,
60 Drer_113830_2,
61 Hpsi_234025,
62 CiEphf,
63 Baby_41769,
64 Patl_251869,
65 Cast_22203,
66 Vmul_37755,
67 Edun_188839,
68 Mlei_02521a_PA,
69 Ccan_99557,
70 Ocar_909,
71 Adig_13891,
72 Adig_00805,
73 Adig_04039,
74 Adig_12429,
75 Adig_12482,
76 Adig_18229,
77 Adig_15481,
78 Adig_03916,
79 Adig_20930,
80 Apal_405536,
81 Nvec_40993,
82 Adig_13888,
83 Adig_18762,
84 Apal_25755,
85 Apal_549317,
86 Apal_215216,
87 Apal_215213,
88 Apal_215214,
89 Apal_215219,
90 Apal_215211,
91 Apal_215204,
92 Apal_215220,

93	Apal_215207,
94	Apal_549316,
95	Apal_307048,
96	Hdig_11359,
97	Btue_32401,
98	Apal_612045,
99	Apal_455194,
100	Nvec_39123,
101	Nvec_39124,
102	Pcar_315635,
103	Tadh_53296,
104	Cvar_1757,
105	Cvar_18894,
106	Cvar_19692,
107	Cvar_19694,
108	Cvar_19697,
109	Cvar_19695,
110	Cpro_80288_25,
111	Lapi_154083,
112	Kvar_222799,
113	Cele_48040,
114	Ifas_26594,
115	Ifas_36548,
116	Niph_54283,
117	Pfic_36041,
118	Xtes_94992,
119	Xtes_94993,
120	Aque_03862,
121	Aque_12969,
122	Aque_32613,
123	Aque_32612,
124	Aque_37708,
125	Aque_41463,
126	Aque_37706,
127	Aque_16689,
128	Aque_37707,
129	Hamb_7684,
130	Aque_11657,
131	Niph_32084,
132	Slac_1485,
133	Cele_137847,
134	Kvar_25216,
135	Lapi_88092,
136	Cvar_27357,
137	Cpro_59066_22,
138	Cele_124391,
139	Kvar_4564,
140	Lapi_96494,
141	Cele_85965,
142	Kvar_2233,
143	Lapi_108357,
144	Lapi_159044,
145	Kvar_222743,
146	Kvar_137296,

```
                147     Lapi_126388,
                148     Cpro_75438_40,
                149     Cpro_78852_34,
                150     Psub_14382,
                151     Psub_7666,
                152     Avas_56846,
                153     Avas_56854,
                154     Avas_56851,
                155     Avas_93988,
                156     Avas_93993,
                157     Snux_109225,
                158     Avas_85009,
                159     Rfib_2729,
                160     Snux_119736,
                161     Avas_82832,
                162     Rfib_47059,
                163     Avas_63694,
                164     Hpop_15474,
                165     Cnul_32701,
                166     Cnul_32703,
                167     Pper_114995,
                168     Tadh_56933,
                169     Aele_22617,
                170     Clat_102964,
                171     Aspe_Peph_158325,
                172     Cowc_Peph_08089T0,
                173     Mbre_Peph_9190,
                174     Mbre_Peph_26435
                ;
   tree con_50_majrule = [&U]
(1[&prob=1.00000000e+00,prob_stddev=0.00000000e+00,prob_range={1.000
00000e+00,1.00000000e+00},prob(percent)="100",prob+-sd="100+-0"]:
8.485125e-02[&length_mean=8.65232820e-02,length_median=8.48512500e-0
2,length_95%HPD={4.38160600e-02,1.36130300e-01}],
2[&prob=1.00000000e+00,prob_stddev=0.00000000e+00,prob_range={1.0000
0000e+00,1.00000000e+00},prob(percent)="100",prob+-sd="100+-0"]:
1.476041e-02[&length_mean=1.79260163e-02,length_median=1.47604100e-0
2,length_95%HPD={6.33573300e-06,4.53031600e-02}],
(((3[&prob=1.00000000e+00,prob_stddev=0.00000000e+00,prob_range={1.0
0000000e+00,1.00000000e+00},prob(percent)="100",prob+-sd="100+-0"]:
1.257873e-02[&length_mean=1.47775599e-02,length_median=1.25787300e-0
2,length_95%HPD={8.39399700e-06,3.62721000e-02}],
4[&prob=1.00000000e+00,prob_stddev=0.00000000e+00,prob_range={1.0000
0000e+00,1.00000000e+00},prob(percent)="100",prob+-sd="100+-0"]:
4.870766e-02[&length_mean=5.08194984e-02,length_median=4.87076600e-0
2,length_95%HPD={1.51211400e-02,8.75323100e-02}])
[&prob=1.00000000e+00,prob_stddev=0.00000000e+00,prob_range={1.00000
000e+00,1.00000000e+00},prob(percent)="100",prob+-sd="100+-0"]:
1.886357e-01[&length_mean=1.91816003e-01,length_median=1.88635700e-0
1,length_95%HPD={1.05300200e-01,2.86482100e-01}],
(5[&prob=1.00000000e+00,prob_stddev=0.00000000e+00,prob_range={1.000
00000e+00,1.00000000e+00},prob(percent)="100",prob+-sd="100+-0"]:
3.097311e-01[&length_mean=3.13606809e-01,length_median=3.09731100e-0
1,length_95%HPD={2.05543300e-01,4.29405700e-01}],
```

6[&prob=1.00000000e+00,prob_stddev=0.00000000e+00,prob_range={1.0000
0000e+00,1.00000000e+00},prob(percent)="100",prob+-sd="100+-0"]:
2.873725e-01[&length_mean=2.89467898e-01,length_median=2.87372500e-0
1,length_95%HPD={1.85182200e-01,3.96600800e-01}])
[&prob=1.00000000e+00,prob_stddev=0.00000000e+00,prob_range={1.00000
000e+00,1.00000000e+00},prob(percent)="100",prob+-sd="100+-0"]:
2.318189e-01[&length_mean=2.34457824e-01,length_median=2.31818900e-0
1,length_95%HPD={1.24959000e-01,3.55652700e-01}])
[&prob=9.64690458e-01,prob_stddev=1.78182990e-02,prob_range={9.52091
018e-01,9.77289898e-01},prob(percent)="96",prob+-sd="96+-2"]:
9.795014e-02[&length_mean=1.01914588e-01,length_median=9.79501400e-0
2,length_95%HPD={3.37045500e-02,1.79917200e-01}],
((((7[&prob=1.00000000e+00,prob_stddev=0.00000000e+00,prob_range={1.
00000000e+00,1.00000000e+00},prob(percent)="100",prob+-sd="100+-0"]:
3.154516e-02[&length_mean=3.42758266e-02,length_median=3.15451600e-0
2,length_95%HPD={4.23913800e-03,6.85819000e-02}],
8[&prob=1.00000000e+00,prob_stddev=0.00000000e+00,prob_range={1.0000
0000e+00,1.00000000e+00},prob(percent)="100",prob+-sd="100+-0"]:
9.305915e-02[&length_mean=9.46694197e-02,length_median=9.30591500e-0
2,length_95%HPD={4.41748600e-02,1.45658700e-01}])
[&prob=1.00000000e+00,prob_stddev=0.00000000e+00,prob_range={1.00000
000e+00,1.00000000e+00},prob(percent)="100",prob+-sd="100+-0"]:
9.980976e-02[&length_mean=1.01447628e-01,length_median=9.98097600e-0
2,length_95%HPD={4.60658000e-02,1.60160400e-01}],
(9[&prob=1.00000000e+00,prob_stddev=0.00000000e+00,prob_range={1.000
00000e+00,1.00000000e+00},prob(percent)="100",prob+-sd="100+-0"]:
4.029769e-02[&length_mean=4.25586788e-02,length_median=4.02976900e-0
2,length_95%HPD={4.90088400e-03,8.20462600e-02}],
10[&prob=1.00000000e+00,prob_stddev=0.00000000e+00,prob_range={1.000
00000e+00,1.00000000e+00},prob(percent)="100",prob+-sd="100+-0"]:
1.480093e-01[&length_mean=1.50295952e-01,length_median=1.48009300e-0
1,length_95%HPD={9.16943100e-02,2.19831600e-01}])
[&prob=1.00000000e+00,prob_stddev=0.00000000e+00,prob_range={1.00000
000e+00,1.00000000e+00},prob(percent)="100",prob+-sd="100+-0"]:
1.273923e-01[&length_mean=1.29810688e-01,length_median=1.27392300e-0
1,length_95%HPD={6.98285100e-02,1.94641400e-01}])
[&prob=9.02271010e-01,prob_stddev=9.05056455e-03,prob_range={8.95871
295e-01,9.08670726e-01},prob(percent)="90",prob+-sd="90+-1"]:
7.094540e-02[&length_mean=7.35985897e-02,length_median=7.09454000e-0
2,length_95%HPD={2.54274800e-02,1.24397200e-01}],
((((11[&prob=1.00000000e+00,prob_stddev=0.00000000e+00,prob_range={1
.00000000e+00,1.00000000e+00},prob(percent)="100",prob+-
sd="100+-0"]:
1.812508e-02[&length_mean=2.09963969e-02,length_median=1.81250800e-0
2,length_95%HPD={2.44332400e-05,4.72376400e-02}],
(12[&prob=1.00000000e+00,prob_stddev=0.00000000e+00,prob_range={1.00
000000e+00,1.00000000e+00},prob(percent)="100",prob+-sd="100+-0"]:
3.585758e-02[&length_mean=3.77067147e-02,length_median=3.58575800e-0
2,length_95%HPD={9.22542000e-03,7.09138400e-02}],
13[&prob=1.00000000e+00,prob_stddev=0.00000000e+00,prob_range={1.000
00000e+00,1.00000000e+00},prob(percent)="100",prob+-sd="100+-0"]:
1.129396e-01[&length_mean=1.15065363e-01,length_median=1.12939600e-0
1,length_95%HPD={5.74415200e-02,1.72347100e-01}])
[&prob=9.99577797e-01,prob_stddev=5.97085856e-04,prob_range={9.99155

593e-01,1.00000000e+00},prob(percent)="100",prob+-sd="100+-0"]:
4.781157e-02[&length_mean=4.99131183e-02,length_median=4.78115700e-0
2,length_95%HPD={1.62329100e-02,8.70955100e-02}])
[&prob=9.99555575e-01,prob_stddev=6.28511427e-05,prob_range={9.99511
133e-01,9.99600018e-01},prob(percent)="100",prob+-sd="100+-0"]:
5.891364e-02[&length_mean=6.16062604e-02,length_median=5.89136400e-0
2,length_95%HPD={1.63677800e-02,1.08886600e-01}],
(18[&prob=1.00000000e+00,prob_stddev=0.00000000e+00,prob_range={1.00
000000e+00,1.00000000e+00},prob(percent)="100",prob+-sd="100+-0"]:
8.778532e-02[&length_mean=8.87691871e-02,length_median=8.77853200e-0
2,length_95%HPD={3.72062400e-02,1.36776900e-01}],
19[&prob=1.00000000e+00,prob_stddev=0.00000000e+00,prob_range={1.000
00000e+00,1.00000000e+00},prob(percent)="100",prob+-sd="100+-0"]:
9.911115e-02[&length_mean=1.01090348e-01,length_median=9.91111500e-0
2,length_95%HPD={4.73816200e-02,1.57032900e-01}])
[&prob=1.00000000e+00,prob_stddev=0.00000000e+00,prob_range={1.00000
000e+00,1.00000000e+00},prob(percent)="100",prob+-sd="100+-0"]:
1.412205e-01[&length_mean=1.42235032e-01,length_median=1.41220500e-0
1,length_95%HPD={7.68673700e-02,2.10149600e-01}])
[&prob=9.62868317e-01,prob_stddev=2.86286955e-02,prob_range={9.42624
772e-01,9.83111862e-01},prob(percent)="96",prob+-sd="96+-3"]:
8.501679e-02[&length_mean=8.72962373e-02,length_median=8.50167900e-0
2,length_95%HPD={3.05048300e-02,1.45961700e-01}],
(20[&prob=1.00000000e+00,prob_stddev=0.00000000e+00,prob_range={1.00
000000e+00,1.00000000e+00},prob(percent)="100",prob+-sd="100+-0"]:
5.272743e-02[&length_mean=5.56196215e-02,length_median=5.27274300e-0
2,length_95%HPD={1.83732900e-02,9.70588000e-02}],
21[&prob=1.00000000e+00,prob_stddev=0.00000000e+00,prob_range={1.000
00000e+00,1.00000000e+00},prob(percent)="100",prob+-sd="100+-0"]:
1.187183e-01[&length_mean=1.20515574e-01,length_median=1.18718300e-0
1,length_95%HPD={6.81514000e-02,1.75447100e-01}])
[&prob=1.00000000e+00,prob_stddev=0.00000000e+00,prob_range={1.00000
000e+00,1.00000000e+00},prob(percent)="100",prob+-sd="100+-0"]:
1.206569e-01[&length_mean=1.24320700e-01,length_median=1.20656900e-0
1,length_95%HPD={6.86635500e-02,1.89483700e-01}])
[&prob=6.89680459e-01,prob_stddev=1.11277948e-01,prob_range={6.10995
067e-01,7.68365850e-01},prob(percent)="69",prob+-sd="69+-11"]:
3.595325e-02[&length_mean=3.82899367e-02,length_median=3.59532500e-0
2,length_95%HPD={1.56558900e-03,8.04555400e-02}],
(((14[&prob=1.00000000e+00,prob_stddev=0.00000000e+00,prob_range={1.
00000000e+00,1.00000000e+00},prob(percent)="100",prob+-sd="100+-0"]:
9.297780e-02[&length_mean=9.46663661e-02,length_median=9.29778000e-0
2,length_95%HPD={4.48397200e-02,1.49128300e-01}],
15[&prob=1.00000000e+00,prob_stddev=0.00000000e+00,prob_range={1.000
00000e+00,1.00000000e+00},prob(percent)="100",prob+-sd="100+-0"]:
1.321352e-01[&length_mean=1.34695185e-01,length_median=1.32135200e-0
1,length_95%HPD={7.58305100e-02,1.95241500e-01}])
[&prob=9.99511133e-01,prob_stddev=0.00000000e+00,prob_range={9.99511
133e-01,9.99511133e-01},prob(percent)="100",prob+-sd="100+-0"]:
5.493567e-02[&length_mean=5.77752692e-02,length_median=5.49356700e-0
2,length_95%HPD={1.44482600e-02,1.01734100e-01}],
(((27[&prob=1.00000000e+00,prob_stddev=0.00000000e+00,prob_range={1.
00000000e+00,1.00000000e+00},prob(percent)="100",prob+-sd="100+-0"]:
6.869773e-02[&length_mean=7.11067625e-02,length_median=6.86977300e-0

2,length_95%HPD={3.06945700e-02,1.13940400e-01}],
28[&prob=1.00000000e+00,prob_stddev=0.00000000e+00,prob_range={1.000
00000e+00,1.00000000e+00},prob(percent)="100",prob+-sd="100+-0"]:
2.030384e-02[&length_mean=2.23720636e-02,length_median=2.03038400e-0
2,length_95%HPD={1.73322700e-03,4.66804900e-02}])
[&prob=1.00000000e+00,prob_stddev=0.00000000e+00,prob_range={1.00000
000e+00,1.00000000e+00},prob(percent)="100",prob+-sd="100+-0"]:
9.429192e-02[&length_mean=9.63101759e-02,length_median=9.42919200e-0
2,length_95%HPD={5.04369000e-02,1.50415900e-01}],
((29[&prob=1.00000000e+00,prob_stddev=0.00000000e+00,prob_range={1.0
0000000e+00,1.00000000e+00},prob(percent)="100",prob+-sd="100+-0"]:
6.263814e-02[&length_mean=6.50393109e-02,length_median=6.26381400e-0
2,length_95%HPD={2.88716100e-02,1.09328400e-01}],
(30[&prob=1.00000000e+00,prob_stddev=0.00000000e+00,prob_range={1.00
000000e+00,1.00000000e+00},prob(percent)="100",prob+-sd="100+-0"]:
2.337057e-02[&length_mean=2.56841145e-02,length_median=2.33705700e-0
2,length_95%HPD={2.43227300e-03,5.38144100e-02}],
31[&prob=1.00000000e+00,prob_stddev=0.00000000e+00,prob_range={1.000
00000e+00,1.00000000e+00},prob(percent)="100",prob+-sd="100+-0"]:
5.880675e-02[&length_mean=6.06919636e-02,length_median=5.88067500e-0
2,length_95%HPD={2.62119900e-02,9.85488900e-02}])
[&prob=9.98044531e-01,prob_stddev=1.06846943e-03,prob_range={9.97289
009e-01,9.98800053e-01},prob(percent)="100",prob+-sd="100+-0"]:
3.738445e-02[&length_mean=3.96844293e-02,length_median=3.73844500e-0
2,length_95%HPD={9.45067900e-03,7.17822500e-02}])
[&prob=1.00000000e+00,prob_stddev=0.00000000e+00,prob_range={1.00000
000e+00,1.00000000e+00},prob(percent)="100",prob+-sd="100+-0"]:
8.093073e-02[&length_mean=8.32071633e-02,length_median=8.09307300e-0
2,length_95%HPD={3.67410600e-02,1.31315400e-01}],
32[&prob=1.00000000e+00,prob_stddev=0.00000000e+00,prob_range={1.000
00000e+00,1.00000000e+00},prob(percent)="100",prob+-sd="100+-0"]:
1.055990e-01[&length_mean=1.07421186e-01,length_median=1.05599000e-0
1,length_95%HPD={5.34579600e-02,1.62224000e-01}])
[&prob=8.66739256e-01,prob_stddev=1.39843793e-02,prob_range={8.56850
807e-01,8.76627705e-01},prob(percent)="87",prob+-sd="87+-1"]:
2.722007e-02[&length_mean=2.95374595e-02,length_median=2.72200700e-0
2,length_95%HPD={2.32381600e-03,6.27050200e-02}])
[&prob=8.21496822e-01,prob_stddev=3.42538728e-03,prob_range={8.19074
708e-01,8.23918937e-01},prob(percent)="82",prob+-sd="82+-0"]:
3.053956e-02[&length_mean=3.32683991e-02,length_median=3.05395600e-0
2,length_95%HPD={3.78197300e-06,7.16233800e-02}],
((33[&prob=1.00000000e+00,prob_stddev=0.00000000e+00,prob_range={1.0
0000000e+00,1.00000000e+00},prob(percent)="100",prob+-sd="100+-0"]:
6.939418e-02[&length_mean=7.17966067e-02,length_median=6.93941800e-0
2,length_95%HPD={2.85951200e-02,1.18784100e-01}],
(34[&prob=1.00000000e+00,prob_stddev=0.00000000e+00,prob_range={1.00
000000e+00,1.00000000e+00},prob(percent)="100",prob+-sd="100+-0"]:
5.306457e-02[&length_mean=5.54264180e-02,length_median=5.30645700e-0
2,length_95%HPD={1.63415300e-02,9.90974200e-02}],
35[&prob=1.00000000e+00,prob_stddev=0.00000000e+00,prob_range={1.000
00000e+00,1.00000000e+00},prob(percent)="100",prob+-sd="100+-0"]:
1.219909e-01[&length_mean=1.24131945e-01,length_median=1.21990900e-0
1,length_95%HPD={7.11451400e-02,1.84509200e-01}])
[&prob=9.26492156e-01,prob_stddev=6.22226313e-03,prob_range={9.22092

351e-01,9.30891960e-01},prob(percent)="93",prob+-sd="93+-1"]:
4.335631e-02[&length_mean=4.60451469e-02,length_median=4.33563100e-0
2,length_95%HPD={1.32269200e-02,8.62142500e-02}])
[&prob=1.00000000e+00,prob_stddev=0.00000000e+00,prob_range={1.00000
000e+00,1.00000000e+00},prob(percent)="100",prob+-sd="100+-0"]:
1.081850e-01[&length_mean=1.11152749e-01,length_median=1.08185000e-0
1,length_95%HPD={5.41169900e-02,1.69205600e-01}],
((((39[&prob=1.00000000e+00,prob_stddev=0.00000000e+00,prob_range={
1.00000000e+00,1.00000000e+00},prob(percent)="100",prob+-
sd="100+-0"]:
1.649655e-01[&length_mean=1.68823835e-01,length_median=1.64965500e-0
1,length_95%HPD={9.58562400e-02,2.51233600e-01}],
40[&prob=1.00000000e+00,prob_stddev=0.00000000e+00,prob_range={1.000
00000e+00,1.00000000e+00},prob(percent)="100",prob+-sd="100+-0"]:
1.679363e-01[&length_mean=1.71229816e-01,length_median=1.67936300e-0
1,length_95%HPD={9.26080400e-02,2.44309200e-01}])
[&prob=1.00000000e+00,prob_stddev=0.00000000e+00,prob_range={1.00000
000e+00,1.00000000e+00},prob(percent)="100",prob+-sd="100+-0"]:
1.990852e-01[&length_mean=2.00909583e-01,length_median=1.99085200e-0
1,length_95%HPD={1.02343000e-01,2.93289800e-01}],
47[&prob=1.00000000e+00,prob_stddev=0.00000000e+00,prob_range={1.000
00000e+00,1.00000000e+00},prob(percent)="100",prob+-sd="100+-0"]:
3.608184e-01[&length_mean=3.61823100e-01,length_median=3.60818400e-0
1,length_95%HPD={2.01313400e-01,4.99759100e-01}],
62[&prob=1.00000000e+00,prob_stddev=0.00000000e+00,prob_range={1.000
00000e+00,1.00000000e+00},prob(percent)="100",prob+-sd="100+-0"]:
7.886367e-01[&length_mean=7.92670784e-01,length_median=7.88636700e-0
1,length_95%HPD={5.87182500e-01,9.86266800e-01}])
[&prob=9.81667481e-01,prob_stddev=1.36072724e-02,prob_range={9.72045
687e-01,9.91289276e-01},prob(percent)="98",prob+-sd="98+-1"]:
1.405470e-01[&length_mean=1.42679431e-01,length_median=1.40547000e-0
1,length_95%HPD={4.61916400e-02,2.32697800e-01}],
((41[&prob=1.00000000e+00,prob_stddev=0.00000000e+00,prob_range={1.0
0000000e+00,1.00000000e+00},prob(percent)="100",prob+-sd="100+-0"]:
1.254432e-01[&length_mean=1.28175705e-01,length_median=1.25443200e-0
1,length_95%HPD={6.19487400e-02,2.00286000e-01}],
42[&prob=1.00000000e+00,prob_stddev=0.00000000e+00,prob_range={1.000
00000e+00,1.00000000e+00},prob(percent)="100",prob+-sd="100+-0"]:
1.016564e-01[&length_mean=1.04578321e-01,length_median=1.01656400e-0
1,length_95%HPD={4.47875600e-02,1.67032900e-01}])
[&prob=1.00000000e+00,prob_stddev=0.00000000e+00,prob_range={1.00000
000e+00,1.00000000e+00},prob(percent)="100",prob+-sd="100+-0"]:
2.295057e-01[&length_mean=2.30779833e-01,length_median=2.29505700e-0
1,length_95%HPD={1.30213700e-01,3.37840600e-01}],
(48[&prob=1.00000000e+00,prob_stddev=0.00000000e+00,prob_range={1.00
000000e+00,1.00000000e+00},prob(percent)="100",prob+-sd="100+-0"]:
3.659925e-01[&length_mean=3.69120952e-01,length_median=3.65992500e-0
1,length_95%HPD={2.48955600e-01,4.99086200e-01}],
55[&prob=1.00000000e+00,prob_stddev=0.00000000e+00,prob_range={1.000
00000e+00,1.00000000e+00},prob(percent)="100",prob+-sd="100+-0"]:
4.740262e-01[&length_mean=4.78100427e-01,length_median=4.74026200e-0
1,length_95%HPD={3.38369300e-01,6.25425100e-01}])
[&prob=9.99800009e-01,prob_stddev=2.19979000e-04,prob_range={9.99644
460e-01,9.99955558e-01},prob(percent)="100",prob+-sd="100+-0"]:

1.807893e-01[&length_mean=1.85312325e-01,length_median=1.80789300e-0
1,length_95%HPD={7.64482700e-02,2.96762400e-01}],
(((((49[&prob=1.00000000e+00,prob_stddev=0.00000000e+00,prob_range={
1.00000000e+00,1.00000000e+00},prob(percent)="100",prob+-
sd="100+-0"]:
2.222208e-02[&length_mean=2.63931557e-02,length_median=2.22220800e-0
2,length_95%HPD={5.78941200e-05,6.51539200e-02}],
50[&prob=1.00000000e+00,prob_stddev=0.00000000e+00,prob_range={1.000
00000e+00,1.00000000e+00},prob(percent)="100",prob+-sd="100+-0"]:
4.775002e-02[&length_mean=5.13597738e-02,length_median=4.77500200e-0
2,length_95%HPD={1.50381100e-06,1.08698800e-01}])
[&prob=5.08132972e-01,prob_stddev=1.09989500e-03,prob_range={5.07355
229e-01,5.08910715e-01},prob(percent)="51",prob+-sd="51+-0"]:
4.157547e-02[&length_mean=4.81717506e-02,length_median=4.15754700e-0
2,length_95%HPD={2.48986500e-05,1.11914300e-01}],
51[&prob=1.00000000e+00,prob_stddev=0.00000000e+00,prob_range={1.000
00000e+00,1.00000000e+00},prob(percent)="100",prob+-sd="100+-0"]:
7.287700e-02[&length_mean=7.66339287e-02,length_median=7.28770000e-0
2,length_95%HPD={3.87431200e-05,1.52477200e-01}])
[&prob=9.87200569e-01,prob_stddev=2.51404571e-04,prob_range={9.87022
799e-01,9.87378339e-01},prob(percent)="99",prob+-sd="99+-0"]:
2.609258e-01[&length_mean=2.65416897e-01,length_median=2.60925800e-0
1,length_95%HPD={5.71609000e-02,4.57266000e-01}],
61[&prob=1.00000000e+00,prob_stddev=0.00000000e+00,prob_range={1.000
00000e+00,1.00000000e+00},prob(percent)="100",prob+-sd="100+-0"]:
7.100954e-01[&length_mean=7.17471583e-01,length_median=7.10095400e-0
1,length_95%HPD={4.97683800e-01,9.59178000e-01}])
[&prob=7.60855073e-01,prob_stddev=2.16836442e-02,prob_range={7.45522
421e-01,7.76187725e-01},prob(percent)="76",prob+-sd="76+-2"]:
1.911981e-01[&length_mean=1.97365561e-01,length_median=1.91198100e-0
1,length_95%HPD={2.16473000e-02,3.73833700e-01}],
(52[&prob=1.00000000e+00,prob_stddev=0.00000000e+00,prob_range={1.00
000000e+00,1.00000000e+00},prob(percent)="100",prob+-sd="100+-0"]:
3.092930e-01[&length_mean=3.13548404e-01,length_median=3.09293000e-0
1,length_95%HPD={2.00903200e-01,4.33466800e-01}],
53[&prob=1.00000000e+00,prob_stddev=0.00000000e+00,prob_range={1.000
00000e+00,1.00000000e+00},prob(percent)="100",prob+-sd="100+-0"]:
3.167764e-01[&length_mean=3.19049894e-01,length_median=3.16776400e-0
1,length_95%HPD={1.95397400e-01,4.30144200e-01}])
[&prob=9.76601040e-01,prob_stddev=4.08532428e-04,prob_range={9.76312
164e-01,9.76889916e-01},prob(percent)="98",prob+-sd="98+-0"]:
1.426232e-01[&length_mean=1.47827646e-01,length_median=1.42623200e-0
1,length_95%HPD={4.29771200e-02,2.59651900e-01}],
54[&prob=1.00000000e+00,prob_stddev=0.00000000e+00,prob_range={1.000
00000e+00,1.00000000e+00},prob(percent)="100",prob+-sd="100+-0"]:
3.814988e-01[&length_mean=3.85480560e-01,length_median=3.81498800e-0
1,length_95%HPD={2.56416000e-01,5.26805400e-01}])
[&prob=1.00000000e+00,prob_stddev=0.00000000e+00,prob_range={1.00000
000e+00,1.00000000e+00},prob(percent)="100",prob+-sd="100+-0"]:
2.039509e-01[&length_mean=2.08296966e-01,length_median=2.03950900e-0
1,length_95%HPD={7.75993200e-02,3.41502900e-01}],
(((63[&prob=1.00000000e+00,prob_stddev=0.00000000e+00,prob_range={1.
00000000e+00,1.00000000e+00},prob(percent)="100",prob+-sd="100+-0"]:
3.901859e-01[&length_mean=3.91315792e-01,length_median=3.90185900e-0

1,length_95%HPD={2.57643200e-01,5.34948400e-01}],
64[&prob=1.00000000e+00,prob_stddev=0.00000000e+00,prob_range={1.000
00000e+00,1.00000000e+00},prob(percent)="100",prob+-sd="100+-0"]:
3.101531e-01[&length_mean=3.13134900e-01,length_median=3.10153100e-0
1,length_95%HPD={1.86408300e-01,4.43697200e-01}],
(67[&prob=1.00000000e+00,prob_stddev=0.00000000e+00,prob_range={1.00
000000e+00,1.00000000e+00},prob(percent)="100",prob+-sd="100+-0"]:
4.924864e-01[&length_mean=5.05764895e-01,length_median=4.92486400e-0
1,length_95%HPD={2.54423600e-01,7.66415000e-01}],
68[&prob=1.00000000e+00,prob_stddev=0.00000000e+00,prob_range={1.000
00000e+00,1.00000000e+00},prob(percent)="100",prob+-sd="100+-0"]:
5.886700e-01[&length_mean=5.85545822e-01,length_median=5.88670000e-0
1,length_95%HPD={2.87488300e-01,9.01751300e-01}])
[&prob=5.02910982e-01,prob_stddev=7.40386461e-02,prob_range={4.50557
753e-01,5.55264210e-01},prob(percent)="50",prob+-sd="50+-7"]:
2.808448e-01[&length_mean=2.91665954e-01,length_median=2.80844800e-0
1,length_95%HPD={9.05679200e-02,4.97290000e-01}])
[&prob=5.17177014e-01,prob_stddev=7.43529018e-02,prob_range={4.64601
573e-01,5.69752455e-01},prob(percent)="52",prob+-sd="52+-7"]:
2.239089e-01[&length_mean=2.23928913e-01,length_median=2.23908900e-0
1,length_95%HPD={8.35392300e-02,3.56960200e-01}],
65[&prob=1.00000000e+00,prob_stddev=0.00000000e+00,prob_range={1.000
00000e+00,1.00000000e+00},prob(percent)="100",prob+-sd="100+-0"]:
3.020558e-01[&length_mean=3.03433617e-01,length_median=3.02055800e-0
1,length_95%HPD={1.60518000e-01,4.27008500e-01}],
66[&prob=1.00000000e+00,prob_stddev=0.00000000e+00,prob_range={1.000
00000e+00,1.00000000e+00},prob(percent)="100",prob+-sd="100+-0"]:
3.230904e-01[&length_mean=3.30012476e-01,length_median=3.23090400e-0
1,length_95%HPD={1.73547400e-01,4.91610000e-01}])
[&prob=9.99888894e-01,prob_stddev=1.57127857e-04,prob_range={9.99777
788e-01,1.00000000e+00},prob(percent)="100",prob+-sd="100+-0"]:
5.792994e-01[&length_mean=5.80578239e-01,length_median=5.79299400e-0
1,length_95%HPD={3.04276500e-01,8.56286800e-01}],
(69[&prob=1.00000000e+00,prob_stddev=0.00000000e+00,prob_range={1.00
000000e+00,1.00000000e+00},prob(percent)="100",prob+-sd="100+-0"]:
5.883750e-01[&length_mean=5.95170595e-01,length_median=5.88375000e-0
1,length_95%HPD={3.69864000e-01,8.13298100e-01}],
70[&prob=1.00000000e+00,prob_stddev=0.00000000e+00,prob_range={1.000
00000e+00,1.00000000e+00},prob(percent)="100",prob+-sd="100+-0"]:
5.566234e-01[&length_mean=5.64300150e-01,length_median=5.56623400e-0
1,length_95%HPD={3.44063500e-01,7.91206900e-01}])
[&prob=9.98822275e-01,prob_stddev=2.19979000e-04,prob_range={9.98666
726e-01,9.98977823e-01},prob(percent)="100",prob+-sd="100+-0"]:
4.095887e-01[&length_mean=4.09943151e-01,length_median=4.09588700e-0
1,length_95%HPD={1.72485900e-01,6.26929100e-01}],
(((71[&prob=1.00000000e+00,prob_stddev=0.00000000e+00,prob_range={1.
00000000e+00,1.00000000e+00},prob(percent)="100",prob+-sd="100+-0"]:
5.849816e-01[&length_mean=5.85148814e-01,length_median=5.84981600e-0
1,length_95%HPD={3.82227200e-01,7.81858800e-01}],
(82[&prob=1.00000000e+00,prob_stddev=0.00000000e+00,prob_range={1.00
000000e+00,1.00000000e+00},prob(percent)="100",prob+-sd="100+-0"]:
1.578508e-01[&length_mean=1.61510880e-01,length_median=1.57850800e-0
1,length_95%HPD={6.98436100e-02,2.77372700e-01}],
83[&prob=1.00000000e+00,prob_stddev=0.00000000e+00,prob_range={1.000

00000e+00,1.00000000e+00},prob(percent)="100",prob+-sd="100+-0"]:
1.204493e-01[&length_mean=1.23380303e-01,length_median=1.20449300e-0
1,length_95%HPD={2.43139000e-02,2.12322300e-01}])
[&prob=1.00000000e+00,prob_stddev=0.00000000e+00,prob_range={1.00000
000e+00,1.00000000e+00},prob(percent)="100",prob+-sd="100+-0"]:
8.442470e-01[&length_mean=8.49485838e-01,length_median=8.44247000e-0
1,length_95%HPD={6.06140600e-01,1.09731800e+00}])
[&prob=7.10923959e-01,prob_stddev=5.37377270e-03,prob_range={7.07124
128e-01,7.14723790e-01},prob(percent)="71",prob+-sd="71+-1"]:
1.472519e-01[&length_mean=1.52387771e-01,length_median=1.47251900e-0
1,length_95%HPD={9.35127800e-04,2.91732500e-01}],
(80[&prob=1.00000000e+00,prob_stddev=0.00000000e+00,prob_range={1.00
000000e+00,1.00000000e+00},prob(percent)="100",prob+-sd="100+-0"]:
5.807375e-01[&length_mean=5.86218072e-01,length_median=5.80737500e-0
1,length_95%HPD={3.82077400e-01,8.02011300e-01}],
81[&prob=1.00000000e+00,prob_stddev=0.00000000e+00,prob_range={1.000
00000e+00,1.00000000e+00},prob(percent)="100",prob+-sd="100+-0"]:
5.331938e-01[&length_mean=5.41003563e-01,length_median=5.33193800e-0
1,length_95%HPD={3.44231400e-01,7.45840800e-01}])
[&prob=9.00426648e-01,prob_stddev=8.01352070e-03,prob_range={8.94760
233e-01,9.06093063e-01},prob(percent)="90",prob+-sd="90+-1"]:
2.095398e-01[&length_mean=2.15097254e-01,length_median=2.09539800e-0
1,length_95%HPD={5.18980700e-02,3.97838800e-01}])
[&prob=6.46282387e-01,prob_stddev=1.44557628e-03,prob_range={6.45260
211e-01,6.47304564e-01},prob(percent)="65",prob+-sd="65+-0"]:
1.309361e-01[&length_mean=1.38641849e-01,length_median=1.30936100e-0
1,length_95%HPD={7.20823300e-03,2.65392600e-01}],
((((72[&prob=1.00000000e+00,prob_stddev=0.00000000e+00,prob_range={1
.00000000e+00,1.00000000e+00},prob(percent)="100",prob+-
sd="100+-0"]:
9.422241e-02[&length_mean=9.62040581e-02,length_median=9.42224100e-0
2,length_95%HPD={4.91179100e-02,1.47542800e-01}],
(74[&prob=1.00000000e+00,prob_stddev=0.00000000e+00,prob_range={1.00
000000e+00,1.00000000e+00},prob(percent)="100",prob+-sd="100+-0"]:
8.742128e-02[&length_mean=8.94478573e-02,length_median=8.74212800e-0
2,length_95%HPD={3.90943800e-02,1.41422900e-01}],
(77[&prob=1.00000000e+00,prob_stddev=0.00000000e+00,prob_range={1.00
000000e+00,1.00000000e+00},prob(percent)="100",prob+-sd="100+-0"]:
1.559194e-01[&length_mean=1.56108092e-01,length_median=1.55919400e-0
1,length_95%HPD={6.38254800e-02,2.44016900e-01}],
(78[&prob=1.00000000e+00,prob_stddev=0.00000000e+00,prob_range={1.00
000000e+00,1.00000000e+00},prob(percent)="100",prob+-sd="100+-0"]:
1.100824e-01[&length_mean=1.12010807e-01,length_median=1.10082400e-0
1,length_95%HPD={5.35933200e-02,1.75069800e-01}],
79[&prob=1.00000000e+00,prob_stddev=0.00000000e+00,prob_range={1.000
00000e+00,1.00000000e+00},prob(percent)="100",prob+-sd="100+-0"]:
5.926402e-02[&length_mean=6.17342644e-02,length_median=5.92640200e-0
2,length_95%HPD={1.82759900e-02,1.13785500e-01}])
[&prob=1.00000000e+00,prob_stddev=0.00000000e+00,prob_range={1.00000
000e+00,1.00000000e+00},prob(percent)="100",prob+-sd="100+-0"]:
1.652345e-01[&length_mean=1.69449585e-01,length_median=1.65234500e-0
1,length_95%HPD={9.85188400e-02,2.51665200e-01}])
[&prob=8.31918581e-01,prob_stddev=1.63852929e-01,prob_range={7.16057
064e-01,9.47780099e-01},prob(percent)="83",prob+-sd="83+-16"]:

1.090797e-01[&length_mean=1.10617402e-01,length_median=1.09079700e-0
1,length_95%HPD={3.87869900e-02,1.81420100e-01}])
[&prob=8.15519310e-01,prob_stddev=1.69886639e-01,prob_range={6.95391
316e-01,9.35647305e-01},prob(percent)="82",prob+-sd="82+-17"]:
4.945916e-02[&length_mean=5.25976216e-02,length_median=4.94591600e-0
2,length_95%HPD={1.57854900e-02,9.41560000e-02}])
[&prob=8.19830230e-01,prob_stddev=1.76800264e-01,prob_range={6.94813
564e-01,9.44846896e-01},prob(percent)="82",prob+-sd="82+-18"]:
3.905224e-02[&length_mean=4.15981484e-02,length_median=3.90522400e-0
2,length_95%HPD={1.08761000e-02,7.63553400e-02}],
73[&prob=1.00000000e+00,prob_stddev=0.00000000e+00,prob_range={1.000
00000e+00,1.00000000e+00},prob(percent)="100",prob+-sd="100+-0"]:
8.853735e-02[&length_mean=8.89095900e-02,length_median=8.85373500e-0
2,length_95%HPD={2.15578700e-02,1.49357400e-01}],
(75[&prob=1.00000000e+00,prob_stddev=0.00000000e+00,prob_range={1.00
000000e+00,1.00000000e+00},prob(percent)="100",prob+-sd="100+-0"]:
8.638222e-02[&length_mean=8.90109811e-02,length_median=8.63822200e-0
2,length_95%HPD={3.87697700e-02,1.50132400e-01}],
76[&prob=1.00000000e+00,prob_stddev=0.00000000e+00,prob_range={1.000
00000e+00,1.00000000e+00},prob(percent)="100",prob+-sd="100+-0"]:
1.044482e-01[&length_mean=1.06286368e-01,length_median=1.04448200e-0
1,length_95%HPD={3.94088600e-02,1.68739300e-01}])
[&prob=6.56326385e-01,prob_stddev=9.42138629e-02,prob_range={5.89707
124e-01,7.22945647e-01},prob(percent)="66",prob+-sd="66+-9"]:
2.877511e-02[&length_mean=3.12170270e-02,length_median=2.87751100e-0
2,length_95%HPD={7.26739300e-04,6.35956100e-02}])
[&prob=1.00000000e+00,prob_stddev=0.00000000e+00,prob_range={1.00000
000e+00,1.00000000e+00},prob(percent)="100",prob+-sd="100+-0"]:
7.353724e-01[&length_mean=7.39498435e-01,length_median=7.35372400e-0
1,length_95%HPD={5.38638500e-01,9.57976400e-01}],
((((84[&prob=1.00000000e+00,prob_stddev=0.00000000e+00,prob_range={1
.00000000e+00,1.00000000e+00},prob(percent)="100",prob+-
sd="100+-0"]:
3.001862e-01[&length_mean=3.03388175e-01,length_median=3.00186200e-0
1,length_95%HPD={2.13969200e-01,4.00983000e-01}],
85[&prob=1.00000000e+00,prob_stddev=0.00000000e+00,prob_range={1.000
00000e+00,1.00000000e+00},prob(percent)="100",prob+-sd="100+-0"]:
1.085830e-01[&length_mean=1.11376577e-01,length_median=1.08583000e-0
1,length_95%HPD={4.78026300e-02,1.78863400e-01}],
(86[&prob=1.00000000e+00,prob_stddev=0.00000000e+00,prob_range={1.00
000000e+00,1.00000000e+00},prob(percent)="100",prob+-sd="100+-0"]:
4.769395e-02[&length_mean=5.05474188e-02,length_median=4.76939500e-0
2,length_95%HPD={1.27036200e-02,9.50674200e-02}],
(87[&prob=1.00000000e+00,prob_stddev=0.00000000e+00,prob_range={1.00
000000e+00,1.00000000e+00},prob(percent)="100",prob+-sd="100+-0"]:
5.820749e-03[&length_mean=8.28045039e-03,length_median=5.82074900e-0
3,length_95%HPD={2.74580200e-06,2.45048800e-02}],
88[&prob=1.00000000e+00,prob_stddev=0.00000000e+00,prob_range={1.000
00000e+00,1.00000000e+00},prob(percent)="100",prob+-sd="100+-0"]:
1.279279e-01[&length_mean=1.30058226e-01,length_median=1.27927900e-0
1,length_95%HPD={7.12023400e-02,1.91684800e-01}])
[&prob=1.00000000e+00,prob_stddev=0.00000000e+00,prob_range={1.00000
000e+00,1.00000000e+00},prob(percent)="100",prob+-sd="100+-0"]:
1.167159e-01[&length_mean=1.18629221e-01,length_median=1.16715900e-0

1,length_95%HPD={6.14905400e-02,1.82949400e-01}])
[&prob=9.89200480e-01,prob_stddev=5.90800742e-03,prob_range={9.85022
888e-01,9.93378072e-01},prob(percent)="99",prob+-sd="99+-1"]:
7.098128e-02[&length_mean=7.41060313e-02,length_median=7.09812800e-0
2,length_95%HPD={2.24878500e-02,1.29005300e-01}],
(89[&prob=1.00000000e+00,prob_stddev=0.00000000e+00,prob_range={1.00
000000e+00,1.00000000e+00},prob(percent)="100",prob+-sd="100+-0"]:
4.837375e-02[&length_mean=5.07740895e-02,length_median=4.83737500e-0
2,length_95%HPD={1.52158100e-02,8.64228600e-02}],
90[&prob=1.00000000e+00,prob_stddev=0.00000000e+00,prob_range={1.000
00000e+00,1.00000000e+00},prob(percent)="100",prob+-sd="100+-0"]:
1.073746e-01[&length_mean=1.09094881e-01,length_median=1.07374600e-0
1,length_95%HPD={5.85251600e-02,1.58405800e-01}])
[&prob=1.00000000e+00,prob_stddev=0.00000000e+00,prob_range={1.00000
000e+00,1.00000000e+00},prob(percent)="100",prob+-sd="100+-0"]:
1.375448e-01[&length_mean=1.39504552e-01,length_median=1.37544800e-0
1,length_95%HPD={8.37536700e-02,2.00695600e-01}],
91[&prob=1.00000000e+00,prob_stddev=0.00000000e+00,prob_range={1.000
00000e+00,1.00000000e+00},prob(percent)="100",prob+-sd="100+-0"]:
2.098657e-01[&length_mean=2.11588059e-01,length_median=2.09865700e-0
1,length_95%HPD={1.23805800e-01,2.98752700e-01}],
((92[&prob=1.00000000e+00,prob_stddev=0.00000000e+00,prob_range={1.0
0000000e+00,1.00000000e+00},prob(percent)="100",prob+-sd="100+-0"]:
3.929255e-02[&length_mean=4.12845954e-02,length_median=3.92925500e-0
2,length_95%HPD={8.26915600e-03,7.71265900e-02}],
93[&prob=1.00000000e+00,prob_stddev=0.00000000e+00,prob_range={1.000
00000e+00,1.00000000e+00},prob(percent)="100",prob+-sd="100+-0"]:
9.479978e-02[&length_mean=9.67809002e-02,length_median=9.47997800e-0
2,length_95%HPD={5.05595000e-02,1.49599900e-01}])
[&prob=1.00000000e+00,prob_stddev=0.00000000e+00,prob_range={1.00000
000e+00,1.00000000e+00},prob(percent)="100",prob+-sd="100+-0"]:
1.068991e-01[&length_mean=1.09266544e-01,length_median=1.06899100e-0
1,length_95%HPD={5.51374800e-02,1.70294700e-01}],
94[&prob=1.00000000e+00,prob_stddev=0.00000000e+00,prob_range={1.000
00000e+00,1.00000000e+00},prob(percent)="100",prob+-sd="100+-0"]:
1.903042e-01[&length_mean=1.94784134e-01,length_median=1.90304200e-0
1,length_95%HPD={1.13990200e-01,2.87267000e-01}])
[&prob=6.60637305e-01,prob_stddev=7.00790241e-02,prob_range={6.11083
952e-01,7.10190658e-01},prob(percent)="66",prob+-sd="66+-7"]:
4.734499e-02[&length_mean=5.02903378e-02,length_median=4.73449900e-0
2,length_95%HPD={1.00793200e-02,9.40175300e-02}])
[&prob=8.73005644e-01,prob_stddev=2.96343138e-02,prob_range={8.52051
020e-01,8.93960268e-01},prob(percent)="87",prob+-sd="87+-3"]:
1.561929e-01[&length_mean=1.61915909e-01,length_median=1.56192900e-0
1,length_95%HPD={5.07946000e-02,2.89980800e-01}],
95[&prob=1.00000000e+00,prob_stddev=0.00000000e+00,prob_range={1.000
00000e+00,1.00000000e+00},prob(percent)="100",prob+-sd="100+-0"]:
5.839299e-01[&length_mean=5.84733162e-01,length_median=5.83929900e-0
1,length_95%HPD={3.84039700e-01,7.63173000e-01}])
[&prob=7.49633350e-01,prob_stddev=8.01352070e-03,prob_range={7.43966
935e-01,7.55299764e-01},prob(percent)="75",prob+-sd="75+-1"]:
1.780165e-01[&length_mean=1.88289215e-01,length_median=1.78016500e-0
1,length_95%HPD={3.06784000e-02,3.68832400e-01}],
96[&prob=1.00000000e+00,prob_stddev=0.00000000e+00,prob_range={1.000

00000e+00,1.00000000e+00},prob(percent)="100",prob+-sd="100+-0"]:
3.699654e-01[&length_mean=3.74914774e-01,length_median=3.69965400e-0
1,length_95%HPD={2.03214200e-01,5.55135300e-01}])
[&prob=9.97955646e-01,prob_stddev=8.79915998e-04,prob_range={9.97333
452e-01,9.98577841e-01},prob(percent)="100",prob+-sd="100+-0"]:
1.929040e-01[&length_mean=1.99693488e-01,length_median=1.92904000e-0
1,length_95%HPD={5.11598300e-02,3.47708400e-01}],
(97[&prob=1.00000000e+00,prob_stddev=0.00000000e+00,prob_range={1.00
000000e+00,1.00000000e+00},prob(percent)="100",prob+-sd="100+-0"]:
4.806882e-01[&length_mean=4.84670176e-01,length_median=4.80688200e-0
1,length_95%HPD={2.82509800e-01,7.03362300e-01}],
(98[&prob=1.00000000e+00,prob_stddev=0.00000000e+00,prob_range={1.00
000000e+00,1.00000000e+00},prob(percent)="100",prob+-sd="100+-0"]:
3.842316e-01[&length_mean=3.90838473e-01,length_median=3.84231600e-0
1,length_95%HPD={2.21303900e-01,5.96724300e-01}],
99[&prob=1.00000000e+00,prob_stddev=0.00000000e+00,prob_range={1.000
00000e+00,1.00000000e+00},prob(percent)="100",prob+-sd="100+-0"]:
4.406282e-01[&length_mean=4.45732643e-01,length_median=4.40628200e-0
1,length_95%HPD={2.62806400e-01,6.38699600e-01}])
[&prob=9.99155593e-01,prob_stddev=6.28511427e-05,prob_range={9.99111
151e-01,9.99200036e-01},prob(percent)="100",prob+-sd="100+-0"]:
2.871653e-01[&length_mean=2.91305567e-01,length_median=2.87165300e-0
1,length_95%HPD={1.17114100e-01,4.71616400e-01}],
(100[&prob=1.00000000e+00,prob_stddev=0.00000000e+00,prob_range={1.0
0000000e+00,1.00000000e+00},prob(percent)="100",prob+-sd="100+-0"]:
3.340542e-02[&length_mean=3.68362522e-02,length_median=3.34054200e-0
2,length_95%HPD={2.13823000e-05,7.53391900e-02}],
101[&prob=1.00000000e+00,prob_stddev=0.00000000e+00,prob_range={1.00
000000e+00,1.00000000e+00},prob(percent)="100",prob+-sd="100+-0"]:
8.080095e-02[&length_mean=8.28638672e-02,length_median=8.08009500e-0
2,length_95%HPD={3.44196100e-02,1.40414800e-01}])
[&prob=1.00000000e+00,prob_stddev=0.00000000e+00,prob_range={1.00000
000e+00,1.00000000e+00},prob(percent)="100",prob+-sd="100+-0"]:
6.140546e-01[&length_mean=6.15960147e-01,length_median=6.14054600e-0
1,length_95%HPD={4.26816600e-01,7.90844100e-01}])
[&prob=8.53806497e-01,prob_stddev=8.51632984e-03,prob_range={8.47784
543e-01,8.59828452e-01},prob(percent)="85",prob+-sd="85+-1"]:
1.436091e-01[&length_mean=1.50316775e-01,length_median=1.43609100e-0
1,length_95%HPD={2.30205500e-02,2.84583300e-01}])
[&prob=9.99155593e-01,prob_stddev=3.14255714e-04,prob_range={9.98933
381e-01,9.99377805e-01},prob(percent)="100",prob+-sd="100+-0"]:
2.408616e-01[&length_mean=2.47203684e-01,length_median=2.40861600e-0
1,length_95%HPD={9.53223300e-02,3.97992900e-01}])
[&prob=5.37465002e-01,prob_stddev=3.10798901e-02,prob_range={5.15488
201e-01,5.59441803e-01},prob(percent)="54",prob+-sd="54+-3"]:
1.085092e-01[&length_mean=1.14647073e-01,length_median=1.08509200e-0
1,length_95%HPD={9.98636700e-04,2.31926100e-01}],
102[&prob=1.00000000e+00,prob_stddev=0.00000000e+00,prob_range={1.00
000000e+00,1.00000000e+00},prob(percent)="100",prob+-sd="100+-0"]:
7.547355e-01[&length_mean=7.64679211e-01,length_median=7.54735500e-0
1,length_95%HPD={5.48861300e-01,9.83287500e-01}])
[&prob=9.93955824e-01,prob_stddev=6.91362570e-04,prob_range={9.93466
957e-01,9.94444691e-01},prob(percent)="99",prob+-sd="99+-0"]:
1.594975e-01[&length_mean=1.66334906e-01,length_median=1.59497500e-0

1,length_95%HPD={3.23917300e-02,3.15039900e-01}])
[&prob=9.89822675e-01,prob_stddev=2.82830142e-03,prob_range={9.87822
763e-01,9.91822586e-01},prob(percent)="99",prob+-sd="99+-0"]:
2.098372e-01[&length_mean=2.13762844e-01,length_median=2.09837200e-0
1,length_95%HPD={5.98205100e-02,3.79017300e-01}],
((103[&prob=1.00000000e+00,prob_stddev=0.00000000e+00,prob_range={1.
00000000e+00,1.00000000e+00},prob(percent)="100",prob+-sd="100+-0"]:
7.116218e-01[&length_mean=7.18447940e-01,length_median=7.11621800e-0
1,length_95%HPD={4.62769100e-01,9.81919200e-01}],
168[&prob=1.00000000e+00,prob_stddev=0.00000000e+00,prob_range={1.00
000000e+00,1.00000000e+00},prob(percent)="100",prob+-sd="100+-0"]:
8.321377e-01[&length_mean=8.38085752e-01,length_median=8.32137700e-0
1,length_95%HPD={5.76697000e-01,1.11975600e+00}])
[&prob=9.97911204e-01,prob_stddev=1.19417171e-03,prob_range={9.97066
797e-01,9.98755611e-01},prob(percent)="100",prob+-sd="100+-0"]:
4.081010e-01[&length_mean=4.13568770e-01,length_median=4.08101000e-0
1,length_95%HPD={1.49172200e-01,6.89023900e-01}],
(169[&prob=1.00000000e+00,prob_stddev=0.00000000e+00,prob_range={1.0
0000000e+00,1.00000000e+00},prob(percent)="100",prob+-sd="100+-0"]:
5.217090e-01[&length_mean=5.27601737e-01,length_median=5.21709000e-0
1,length_95%HPD={3.07642400e-01,7.45309400e-01}],
170[&prob=1.00000000e+00,prob_stddev=0.00000000e+00,prob_range={1.00
000000e+00,1.00000000e+00},prob(percent)="100",prob+-sd="100+-0"]:
5.965317e-01[&length_mean=5.97340510e-01,length_median=5.96531700e-0
1,length_95%HPD={3.70295500e-01,8.47317100e-01}])
[&prob=1.00000000e+00,prob_stddev=0.00000000e+00,prob_range={1.00000
000e+00,1.00000000e+00},prob(percent)="100",prob+-sd="100+-0"]:
5.389580e-01[&length_mean=5.46679236e-01,length_median=5.38958000e-0
1,length_95%HPD={2.99027200e-01,8.17009800e-01}])
[&prob=5.82040798e-01,prob_stddev=4.83639543e-02,prob_range={5.47842
318e-01,6.16239278e-01},prob(percent)="58",prob+-sd="58+-5"]:
1.496822e-01[&length_mean=1.58240881e-01,length_median=1.49682200e-0
1,length_95%HPD={2.74064800e-04,3.28796000e-01}],
((((((104[&prob=1.00000000e+00,prob_stddev=0.00000000e+00,prob_range
={1.00000000e+00,1.00000000e+00},prob(percent)="100",prob+-
sd="100+-0"]:
3.018269e-01[&length_mean=3.06376845e-01,length_median=3.01826900e-0
1,length_95%HPD={2.08994600e-01,4.09857500e-01}],
105[&prob=1.00000000e+00,prob_stddev=0.00000000e+00,prob_range={1.00
000000e+00,1.00000000e+00},prob(percent)="100",prob+-sd="100+-0"]:
1.650042e-01[&length_mean=1.66949621e-01,length_median=1.65004200e-0
1,length_95%HPD={8.77489300e-02,2.53202000e-01}])
[&prob=7.70565753e-01,prob_stddev=5.54661335e-02,prob_range={7.31345
274e-01,8.09786232e-01},prob(percent)="77",prob+-sd="77+-6"]:
6.064761e-02[&length_mean=6.41525057e-02,length_median=6.06476100e-0
2,length_95%HPD={1.09028600e-02,1.26437300e-01}],
106[&prob=1.00000000e+00,prob_stddev=0.00000000e+00,prob_range={1.00
000000e+00,1.00000000e+00},prob(percent)="100",prob+-sd="100+-0"]:
1.027605e-01[&length_mean=1.04845628e-01,length_median=1.02760500e-0
1,length_95%HPD={5.10856500e-02,1.56824400e-01}])
[&prob=6.15883739e-01,prob_stddev=4.18588611e-02,prob_range={5.86285
054e-01,6.45482423e-01},prob(percent)="62",prob+-sd="62+-4"]:
2.081945e-02[&length_mean=2.36359110e-02,length_median=2.08194500e-0
2,length_95%HPD={6.79560600e-05,5.34773300e-02}],

((107[&prob=1.00000000e+00,prob_stddev=0.00000000e+00,prob_range={1.00000000e+00,1.00000000e+00},prob(percent)="100",prob+-sd="100+-0"]:
5.207249e-02[&length_mean=5.41328178e-02,length_median=5.20724900e-02,length_95%HPD={2.25530100e-02,8.99854700e-02}],
108[&prob=1.00000000e+00,prob_stddev=0.00000000e+00,prob_range={1.00000000e+00,1.00000000e+00},prob(percent)="100",prob+-sd="100+-0"]:
4.549047e-03[&length_mean=6.40913722e-03,length_median=4.54904700e-03,length_95%HPD={4.15603900e-06,1.87119200e-02}])
[&prob=1.00000000e+00,prob_stddev=0.00000000e+00,prob_range={1.00000000e+00,1.00000000e+00},prob(percent)="100",prob+-sd="100+-0"]:
3.532060e-02[&length_mean=3.73221060e-02,length_median=3.53206000e-02,length_95%HPD={1.02306000e-02,6.72742700e-02}],
109[&prob=1.00000000e+00,prob_stddev=0.00000000e+00,prob_range={1.00000000e+00,1.00000000e+00},prob(percent)="100",prob+-sd="100+-0"]:
6.456354e-02[&length_mean=6.63763968e-02,length_median=6.45635400e-02,length_95%HPD={2.87770600e-02,1.06827600e-01}])
[&prob=9.94778010e-01,prob_stddev=9.42767141e-05,prob_range={9.94711346e-01,9.94844674e-01},prob(percent)="99",prob+-sd="99+-0"]:
4.219512e-02[&length_mean=4.43378617e-02,length_median=4.21951200e-02,length_95%HPD={1.31710500e-02,8.05852400e-02}])
[&prob=1.00000000e+00,prob_stddev=0.00000000e+00,prob_range={1.00000000e+00,1.00000000e+00},prob(percent)="100",prob+-sd="100+-0"]:
1.864797e-01[&length_mean=1.88736515e-01,length_median=1.86479700e-01,length_95%HPD={9.62232100e-02,2.93759100e-01}],
(110[&prob=1.00000000e+00,prob_stddev=0.00000000e+00,prob_range={1.00000000e+00,1.00000000e+00},prob(percent)="100",prob+-sd="100+-0"]:
2.135414e-01[&length_mean=2.15176364e-01,length_median=2.13541400e-01,length_95%HPD={1.35338800e-01,2.99101900e-01}],
(111[&prob=1.00000000e+00,prob_stddev=0.00000000e+00,prob_range={1.00000000e+00,1.00000000e+00},prob(percent)="100",prob+-sd="100+-0"]:
2.117288e-01[&length_mean=2.14031861e-01,length_median=2.11728800e-01,length_95%HPD={1.23232100e-01,3.01928900e-01}],
(112[&prob=1.00000000e+00,prob_stddev=0.00000000e+00,prob_range={1.00000000e+00,1.00000000e+00},prob(percent)="100",prob+-sd="100+-0"]:
1.922235e-01[&length_mean=1.95007835e-01,length_median=1.92223500e-01,length_95%HPD={6.32831900e-02,3.27037200e-01}],
113[&prob=1.00000000e+00,prob_stddev=0.00000000e+00,prob_range={1.00000000e+00,1.00000000e+00},prob(percent)="100",prob+-sd="100+-0"]:
6.297701e-01[&length_mean=6.37977344e-01,length_median=6.29770100e-01,length_95%HPD={4.35478100e-01,8.56146900e-01}])
[&prob=8.61806142e-01,prob_stddev=1.09675244e-02,prob_range={8.54050931e-01,8.69561353e-01},prob(percent)="86",prob+-sd="86+-1"]:
1.117592e-01[&length_mean=1.17540962e-01,length_median=1.11759200e-01,length_95%HPD={5.25801700e-03,2.24089900e-01}])
[&prob=8.93715835e-01,prob_stddev=3.11113156e-03,prob_range={8.91515933e-01,8.95915737e-01},prob(percent)="89",prob+-sd="89+-0"]:
5.690943e-02[&length_mean=6.01971618e-02,length_median=5.69094300e-02,length_95%HPD={9.31219300e-03,1.15791800e-01}])
[&prob=9.94311364e-01,prob_stddev=1.57127857e-03,prob_range={9.93200302e-01,9.95422426e-01},prob(percent)="99",prob+-sd="99+-0"]:
1.529743e-01[&length_mean=1.55182472e-01,length_median=1.52974300e-01,length_95%HPD={6.04883400e-02,2.64960000e-01}])
[&prob=1.00000000e+00,prob_stddev=0.00000000e+00,prob_range={1.00000000e+00,1.00000000e+00},prob(percent)="100",prob+-sd="100+-0"]:

3.495552e-01[&length_mean=3.53850693e-01,length_median=3.49555200e-0
1,length_95%HPD={1.98397000e-01,5.22700500e-01}],
(114[&prob=1.00000000e+00,prob_stddev=0.00000000e+00,prob_range={1.0
0000000e+00,1.00000000e+00},prob(percent)="100",prob+-sd="100+-0"]:
1.942211e-01[&length_mean=2.00545034e-01,length_median=1.94221100e-0
1,length_95%HPD={5.54483200e-02,3.76074400e-01}],
115[&prob=1.00000000e+00,prob_stddev=0.00000000e+00,prob_range={1.00
000000e+00,1.00000000e+00},prob(percent)="100",prob+-sd="100+-0"]:
2.535908e-01[&length_mean=2.61032854e-01,length_median=2.53590800e-0
1,length_95%HPD={1.00454700e-01,4.34655700e-01}])
[&prob=1.00000000e+00,prob_stddev=0.00000000e+00,prob_range={1.00000
000e+00,1.00000000e+00},prob(percent)="100",prob+-sd="100+-0"]:
3.658427e-01[&length_mean=3.72632219e-01,length_median=3.65842700e-0
1,length_95%HPD={1.63054100e-01,5.95723900e-01}])
[&prob=9.49935558e-01,prob_stddev=1.64355738e-02,prob_range={9.38313
853e-01,9.61557264e-01},prob(percent)="95",prob+-sd="95+-2"]:
1.969574e-01[&length_mean=2.02188616e-01,length_median=1.96957400e-0
1,length_95%HPD={6.15901500e-02,3.61820400e-01}],
((((116[&prob=1.00000000e+00,prob_stddev=0.00000000e+00,prob_range={
1.00000000e+00,1.00000000e+00},prob(percent)="100",prob+-
sd="100+-0"]:
3.066236e-01[&length_mean=3.08180399e-01,length_median=3.06623600e-0
1,length_95%HPD={1.79611300e-01,4.51830800e-01}],
117[&prob=1.00000000e+00,prob_stddev=0.00000000e+00,prob_range={1.00
000000e+00,1.00000000e+00},prob(percent)="100",prob+-sd="100+-0"]:
2.171810e-01[&length_mean=2.22583182e-01,length_median=2.17181000e-0
1,length_95%HPD={9.47688300e-02,3.53033000e-01}])
[&prob=1.00000000e+00,prob_stddev=0.00000000e+00,prob_range={1.00000
000e+00,1.00000000e+00},prob(percent)="100",prob+-sd="100+-0"]:
2.176318e-01[&length_mean=2.21774938e-01,length_median=2.17631800e-0
1,length_95%HPD={8.84147500e-02,3.67414700e-01}],
(118[&prob=1.00000000e+00,prob_stddev=0.00000000e+00,prob_range={1.0
0000000e+00,1.00000000e+00},prob(percent)="100",prob+-sd="100+-0"]:
6.686091e-02[&length_mean=7.08051138e-02,length_median=6.68609100e-0
2,length_95%HPD={2.30933600e-02,1.25444700e-01}],
119[&prob=1.00000000e+00,prob_stddev=0.00000000e+00,prob_range={1.00
000000e+00,1.00000000e+00},prob(percent)="100",prob+-sd="100+-0"]:
7.393261e-02[&length_mean=7.72861602e-02,length_median=7.39326100e-0
2,length_95%HPD={2.28844600e-02,1.31525200e-01}])
[&prob=1.00000000e+00,prob_stddev=0.00000000e+00,prob_range={1.00000
000e+00,1.00000000e+00},prob(percent)="100",prob+-sd="100+-0"]:
2.294839e-01[&length_mean=2.33757601e-01,length_median=2.29483900e-0
1,length_95%HPD={1.30469300e-01,3.41097100e-01}])
[&prob=9.88578285e-01,prob_stddev=1.63412971e-03,prob_range={9.87422
781e-01,9.89733790e-01},prob(percent)="99",prob+-sd="99+-0"]:
1.427444e-01[&length_mean=1.47248341e-01,length_median=1.42744400e-0
1,length_95%HPD={4.51515300e-02,2.56757800e-01}],
(120[&prob=1.00000000e+00,prob_stddev=0.00000000e+00,prob_range={1.0
0000000e+00,1.00000000e+00},prob(percent)="100",prob+-sd="100+-0"]:
4.590949e-02[&length_mean=4.74767997e-02,length_median=4.59094900e-0
2,length_95%HPD={1.56727000e-02,8.15976600e-02}],
121[&prob=1.00000000e+00,prob_stddev=0.00000000e+00,prob_range={1.00
000000e+00,1.00000000e+00},prob(percent)="100",prob+-sd="100+-0"]:
3.954104e-02[&length_mean=4.06980036e-02,length_median=3.95410400e-0

2,length_95%HPD={4.36555300e-03,7.55728400e-02}],
(122[&prob=1.00000000e+00,prob_stddev=0.00000000e+00,prob_range={1.0
0000000e+00,1.00000000e+00},prob(percent)="100",prob+-sd="100+-0"]:
4.344049e-02[&length_mean=4.48311794e-02,length_median=4.34404900e-0
2,length_95%HPD={1.72109000e-02,7.54714200e-02}],
123[&prob=1.00000000e+00,prob_stddev=0.00000000e+00,prob_range={1.00
000000e+00,1.00000000e+00},prob(percent)="100",prob+-sd="100+-0"]:
5.658772e-02[&length_mean=5.90802021e-02,length_median=5.65877200e-0
2,length_95%HPD={2.59783000e-02,9.86219400e-02}])
[&prob=5.10488423e-01,prob_stddev=1.55556578e-02,prob_range={4.99488
912e-01,5.21487934e-01},prob(percent)="51",prob+-sd="51+-2"]:
7.925654e-03[&length_mean=1.02211136e-02,length_median=7.92565400e-0
3,length_95%HPD={5.50644100e-06,2.69069900e-02}],
124[&prob=1.00000000e+00,prob_stddev=0.00000000e+00,prob_range={1.00
000000e+00,1.00000000e+00},prob(percent)="100",prob+-sd="100+-0"]:
3.118504e-02[&length_mean=3.32017869e-02,length_median=3.11850400e-0
2,length_95%HPD={6.96627300e-03,6.33313900e-02}],
125[&prob=1.00000000e+00,prob_stddev=0.00000000e+00,prob_range={1.00
000000e+00,1.00000000e+00},prob(percent)="100",prob+-sd="100+-0"]:
3.129359e-02[&length_mean=3.38923231e-02,length_median=3.12935900e-0
2,length_95%HPD={6.45414200e-03,6.56843700e-02}],
126[&prob=1.00000000e+00,prob_stddev=0.00000000e+00,prob_range={1.00
000000e+00,1.00000000e+00},prob(percent)="100",prob+-sd="100+-0"]:
7.407484e-02[&length_mean=7.62338166e-02,length_median=7.40748400e-0
2,length_95%HPD={3.04710300e-02,1.25801200e-01}],
127[&prob=1.00000000e+00,prob_stddev=0.00000000e+00,prob_range={1.00
000000e+00,1.00000000e+00},prob(percent)="100",prob+-sd="100+-0"]:
1.424149e-01[&length_mean=1.43481383e-01,length_median=1.42414900e-0
1,length_95%HPD={5.53262400e-02,2.16789400e-01}])
[&prob=1.00000000e+00,prob_stddev=0.00000000e+00,prob_range={1.00000
000e+00,1.00000000e+00},prob(percent)="100",prob+-sd="100+-0"]:
3.776611e-01[&length_mean=3.81699377e-01,length_median=3.77661100e-0
1,length_95%HPD={2.59245300e-01,5.21185400e-01}])
[&prob=9.90022666e-01,prob_stddev=5.24807042e-03,prob_range={9.86311
719e-01,9.93733612e-01},prob(percent)="99",prob+-sd="99+-1"]:
1.811098e-01[&length_mean=1.82681950e-01,length_median=1.81109800e-0
1,length_95%HPD={6.15699900e-02,3.08671600e-01}],
128[&prob=1.00000000e+00,prob_stddev=0.00000000e+00,prob_range={1.00
000000e+00,1.00000000e+00},prob(percent)="100",prob+-sd="100+-0"]:
4.633780e-01[&length_mean=4.66445066e-01,length_median=4.63378000e-0
1,length_95%HPD={3.09960700e-01,6.23246300e-01}])
[&prob=1.00000000e+00,prob_stddev=0.00000000e+00,prob_range={1.00000
000e+00,1.00000000e+00},prob(percent)="100",prob+-sd="100+-0"]:
3.538797e-01[&length_mean=3.58349137e-01,length_median=3.53879700e-0
1,length_95%HPD={1.89038000e-01,5.24067000e-01}],
((129[&prob=1.00000000e+00,prob_stddev=0.00000000e+00,prob_range={1.
00000000e+00,1.00000000e+00},prob(percent)="100",prob+-sd="100+-0"]:
2.561056e-01[&length_mean=2.57334549e-01,length_median=2.56105600e-0
1,length_95%HPD={1.55120400e-01,3.66905700e-01}],
130[&prob=1.00000000e+00,prob_stddev=0.00000000e+00,prob_range={1.00
000000e+00,1.00000000e+00},prob(percent)="100",prob+-sd="100+-0"]:
1.936609e-01[&length_mean=1.94449144e-01,length_median=1.93660900e-0
1,length_95%HPD={9.57349300e-02,2.96651400e-01}])
[&prob=9.99244478e-01,prob_stddev=1.06846943e-03,prob_range={9.98488

956e-01,1.00000000e+00},prob(percent)="100",prob+-sd="100+-0"]:
2.922047e-01[&length_mean=2.97379343e-01,length_median=2.92204700e-0
1,length_95%HPD={1.33771000e-01,4.75728700e-01}],
131[&prob=1.00000000e+00,prob_stddev=0.00000000e+00,prob_range={1.00
000000e+00,1.00000000e+00},prob(percent)="100",prob+-sd="100+-0"]:
5.387664e-01[&length_mean=5.44343961e-01,length_median=5.38766400e-0
1,length_95%HPD={3.60927700e-01,7.40761300e-01}])
[&prob=1.00000000e+00,prob_stddev=0.00000000e+00,prob_range={1.00000
000e+00,1.00000000e+00},prob(percent)="100",prob+-sd="100+-0"]:
3.417963e-01[&length_mean=3.41702899e-01,length_median=3.41796300e-0
1,length_95%HPD={1.69321100e-01,5.38143500e-01}],
(132[&prob=1.00000000e+00,prob_stddev=0.00000000e+00,prob_range={1.0
0000000e+00,1.00000000e+00},prob(percent)="100",prob+-sd="100+-0"]:
6.217184e-01[&length_mean=6.24426041e-01,length_median=6.21718400e-0
1,length_95%HPD={4.42737600e-01,8.34530800e-01}],
(136[&prob=1.00000000e+00,prob_stddev=0.00000000e+00,prob_range={1.0
0000000e+00,1.00000000e+00},prob(percent)="100",prob+-sd="100+-0"]:
6.326562e-01[&length_mean=6.34740178e-01,length_median=6.32656200e-0
1,length_95%HPD={4.50954500e-01,8.26393800e-01}],
(((137[&prob=1.00000000e+00,prob_stddev=0.00000000e+00,prob_range={1
.00000000e+00,1.00000000e+00},prob(percent)="100",prob+-
sd="100+-0"]:
2.765952e-01[&length_mean=2.80832820e-01,length_median=2.76595200e-0
1,length_95%HPD={1.59497600e-01,4.03766600e-01}],
(138[&prob=1.00000000e+00,prob_stddev=0.00000000e+00,prob_range={1.0
0000000e+00,1.00000000e+00},prob(percent)="100",prob+-sd="100+-0"]:
2.743559e-01[&length_mean=2.77698496e-01,length_median=2.74355900e-0
1,length_95%HPD={1.67887400e-01,3.99071700e-01}],
139[&prob=1.00000000e+00,prob_stddev=0.00000000e+00,prob_range={1.00
000000e+00,1.00000000e+00},prob(percent)="100",prob+-sd="100+-0"]:
2.243652e-01[&length_mean=2.26693874e-01,length_median=2.24365200e-0
1,length_95%HPD={1.19801200e-01,3.32392900e-01}])
[&prob=9.99577797e-01,prob_stddev=3.14255714e-05,prob_range={9.99555
575e-01,9.99600018e-01},prob(percent)="100",prob+-sd="100+-0"]:
1.564765e-01[&length_mean=1.60617963e-01,length_median=1.56476500e-0
1,length_95%HPD={5.55895500e-02,2.67808500e-01}])
[&prob=9.98311186e-01,prob_stddev=1.25702285e-04,prob_range={9.98222
301e-01,9.98400071e-01},prob(percent)="100",prob+-sd="100+-0"]:
1.519233e-01[&length_mean=1.55056790e-01,length_median=1.51923300e-0
1,length_95%HPD={5.22698600e-02,2.58683600e-01}],
140[&prob=1.00000000e+00,prob_stddev=0.00000000e+00,prob_range={1.00
000000e+00,1.00000000e+00},prob(percent)="100",prob+-sd="100+-0"]:
3.117359e-01[&length_mean=3.16227087e-01,length_median=3.11735900e-0
1,length_95%HPD={2.02394300e-01,4.38159300e-01}])
[&prob=9.97177903e-01,prob_stddev=9.11341569e-04,prob_range={9.96533
487e-01,9.97822319e-01},prob(percent)="100",prob+-sd="100+-0"]:
1.935911e-01[&length_mean=1.95002212e-01,length_median=1.93591100e-0
1,length_95%HPD={8.91189700e-02,3.12184700e-01}],
((141[&prob=1.00000000e+00,prob_stddev=0.00000000e+00,prob_range={1.
00000000e+00,1.00000000e+00},prob(percent)="100",prob+-sd="100+-0"]:
3.594599e-01[&length_mean=3.62038443e-01,length_median=3.59459900e-0
1,length_95%HPD={2.12302500e-01,5.10040500e-01}],
142[&prob=1.00000000e+00,prob_stddev=0.00000000e+00,prob_range={1.00
000000e+00,1.00000000e+00},prob(percent)="100",prob+-sd="100+-0"]:

4.412655e-01[&length_mean=4.44803151e-01,length_median=4.41265500e-0
1,length_95%HPD={2.72169500e-01,6.40391500e-01}])
[&prob=7.35500644e-01,prob_stddev=5.49633243e-02,prob_range={6.96635
705e-01,7.74365584e-01},prob(percent)="74",prob+-sd="74+-5"]:
1.111653e-01[&length_mean=1.14689407e-01,length_median=1.11165300e-0
1,length_95%HPD={2.33033800e-02,2.10012600e-01}],
143[&prob=1.00000000e+00,prob_stddev=0.00000000e+00,prob_range={1.00
000000e+00,1.00000000e+00},prob(percent)="100",prob+-sd="100+-0"]:
3.420076e-01[&length_mean=3.43483935e-01,length_median=3.42007600e-0
1,length_95%HPD={2.04299200e-01,4.83026900e-01}])
[&prob=8.56317497e-01,prob_stddev=4.42472045e-02,prob_range={8.25029
999e-01,8.87604995e-01},prob(percent)="86",prob+-sd="86+-4"]:
8.891249e-02[&length_mean=9.33254784e-02,length_median=8.89124900e-0
2,length_95%HPD={5.29382200e-03,1.80747500e-01}],
(144[&prob=1.00000000e+00,prob_stddev=0.00000000e+00,prob_range={1.0
0000000e+00,1.00000000e+00},prob(percent)="100",prob+-sd="100+-0"]:
2.045890e-01[&length_mean=2.08979765e-01,length_median=2.04589000e-0
1,length_95%HPD={6.86119700e-02,3.50871800e-01}],
148[&prob=1.00000000e+00,prob_stddev=0.00000000e+00,prob_range={1.00
000000e+00,1.00000000e+00},prob(percent)="100",prob+-sd="100+-0"]:
5.162545e-01[&length_mean=5.20751938e-01,length_median=5.16254500e-0
1,length_95%HPD={3.42279800e-01,7.05338200e-01}])
[&prob=5.18399182e-01,prob_stddev=1.36701235e-02,prob_range={5.08732
945e-01,5.28065419e-01},prob(percent)="52",prob+-sd="52+-1"]:
9.872384e-02[&length_mean=1.06068688e-01,length_median=9.87238400e-0
2,length_95%HPD={5.41563300e-05,2.17812500e-01}],
(145[&prob=1.00000000e+00,prob_stddev=0.00000000e+00,prob_range={1.0
0000000e+00,1.00000000e+00},prob(percent)="100",prob+-sd="100+-0"]:
3.254679e-01[&length_mean=3.29712237e-01,length_median=3.25467900e-0
1,length_95%HPD={2.10191400e-01,4.47882400e-01}],
(150[&prob=1.00000000e+00,prob_stddev=0.00000000e+00,prob_range={1.0
0000000e+00,1.00000000e+00},prob(percent)="100",prob+-sd="100+-0"]:
4.445469e-01[&length_mean=4.46648947e-01,length_median=4.44546900e-0
1,length_95%HPD={3.00701900e-01,6.15806800e-01}],
151[&prob=1.00000000e+00,prob_stddev=0.00000000e+00,prob_range={1.00
000000e+00,1.00000000e+00},prob(percent)="100",prob+-sd="100+-0"]:
4.181511e-01[&length_mean=4.23815301e-01,length_median=4.18151100e-0
1,length_95%HPD={2.74241800e-01,5.77090600e-01}])
[&prob=9.96044620e-01,prob_stddev=7.54213713e-04,prob_range={9.95511
311e-01,9.96577930e-01},prob(percent)="100",prob+-sd="100+-0"]:
1.763849e-01[&length_mean=1.79882744e-01,length_median=1.76384900e-0
1,length_95%HPD={6.36280100e-02,3.16416300e-01}])
[&prob=5.23443403e-01,prob_stddev=3.26197431e-02,prob_range={5.00377
761e-01,5.46509044e-01},prob(percent)="52",prob+-sd="52+-3"]:
1.032884e-01[&length_mean=1.05978873e-01,length_median=1.03288400e-0
1,length_95%HPD={1.04422800e-05,1.87783000e-01}],
(146[&prob=1.00000000e+00,prob_stddev=0.00000000e+00,prob_range={1.0
0000000e+00,1.00000000e+00},prob(percent)="100",prob+-sd="100+-0"]:
3.206437e-01[&length_mean=3.25287329e-01,length_median=3.20643700e-0
1,length_95%HPD={2.04885700e-01,4.53442400e-01}],
147[&prob=1.00000000e+00,prob_stddev=0.00000000e+00,prob_range={1.00
000000e+00,1.00000000e+00},prob(percent)="100",prob+-sd="100+-0"]:
2.325307e-01[&length_mean=2.37475898e-01,length_median=2.32530700e-0
1,length_95%HPD={1.37661500e-01,3.45658900e-01}])

[&prob=9.99488912e-01,prob_stddev=3.45681285e-04,prob_range={9.99244
478e-01,9.99733345e-01},prob(percent)="100",prob+-sd="100+-0"]:
1.378643e-01[&length_mean=1.41695185e-01,length_median=1.37864300e-0
1,length_95%HPD={4.64577300e-02,2.34695700e-01}],
149[&prob=1.00000000e+00,prob_stddev=0.00000000e+00,prob_range={1.00
000000e+00,1.00000000e+00},prob(percent)="100",prob+-sd="100+-0"]:
6.221405e-01[&length_mean=6.23783733e-01,length_median=6.22140500e-0
1,length_95%HPD={4.22753500e-01,8.10668700e-01}])
[&prob=8.78538732e-01,prob_stddev=2.63974799e-03,prob_range={8.76672
148e-01,8.80405315e-01},prob(percent)="88",prob+-sd="88+-0"]:
1.822384e-01[&length_mean=1.86257629e-01,length_median=1.82238400e-0
1,length_95%HPD={4.73876700e-02,3.24874700e-01}])
[&prob=9.99844451e-01,prob_stddev=9.42767141e-05,prob_range={9.99777
788e-01,9.99911115e-01},prob(percent)="100",prob+-sd="100+-0"]:
3.153187e-01[&length_mean=3.20961153e-01,length_median=3.15318700e-0
1,length_95%HPD={1.35815700e-01,5.28903000e-01}])
[&prob=5.13310520e-01,prob_stddev=7.03932798e-03,prob_range={5.08332
963e-01,5.18288076e-01},prob(percent)="51",prob+-sd="51+-1"]:
1.937648e-01[&length_mean=1.96661541e-01,length_median=1.93764800e-0
1,length_95%HPD={5.31390400e-02,3.32760300e-01}],
((133[&prob=1.00000000e+00,prob_stddev=0.00000000e+00,prob_range={1.
00000000e+00,1.00000000e+00},prob(percent)="100",prob+-sd="100+-0"]:
5.160747e-01[&length_mean=5.20338837e-01,length_median=5.16074700e-0
1,length_95%HPD={3.52950000e-01,6.87556900e-01}],
134[&prob=1.00000000e+00,prob_stddev=0.00000000e+00,prob_range={1.00
000000e+00,1.00000000e+00},prob(percent)="100",prob+-sd="100+-0"]:
3.117634e-01[&length_mean=3.17486205e-01,length_median=3.11763400e-0
1,length_95%HPD={1.83242600e-01,4.69686000e-01}])
[&prob=9.99622239e-01,prob_stddev=9.42767141e-05,prob_range={9.99555
575e-01,9.99688903e-01},prob(percent)="100",prob+-sd="100+-0"]:
2.116046e-01[&length_mean=2.15743921e-01,length_median=2.11604600e-0
1,length_95%HPD={7.35269600e-02,3.60252400e-01}],
135[&prob=1.00000000e+00,prob_stddev=0.00000000e+00,prob_range={1.00
000000e+00,1.00000000e+00},prob(percent)="100",prob+-sd="100+-0"]:
4.563399e-01[&length_mean=4.60799811e-01,length_median=4.56339900e-0
1,length_95%HPD={3.02315000e-01,6.35627200e-01}])
[&prob=9.99844451e-01,prob_stddev=1.57127857e-04,prob_range={9.99733
345e-01,9.99955558e-01},prob(percent)="100",prob+-sd="100+-0"]:
2.812958e-01[&length_mean=2.84742490e-01,length_median=2.81295800e-0
1,length_95%HPD={1.17897200e-01,4.50102100e-01}],
((165[&prob=1.00000000e+00,prob_stddev=0.00000000e+00,prob_range={1.
00000000e+00,1.00000000e+00},prob(percent)="100",prob+-sd="100+-0"]:
1.049361e-01[&length_mean=1.07721047e-01,length_median=1.04936100e-0
1,length_95%HPD={2.74190700e-02,1.96852000e-01}],
166[&prob=1.00000000e+00,prob_stddev=0.00000000e+00,prob_range={1.00
000000e+00,1.00000000e+00},prob(percent)="100",prob+-sd="100+-0"]:
7.672074e-02[&length_mean=8.08392179e-02,length_median=7.67207400e-0
2,length_95%HPD={1.26295600e-02,1.64421300e-01}])
[&prob=1.00000000e+00,prob_stddev=0.00000000e+00,prob_range={1.00000
000e+00,1.00000000e+00},prob(percent)="100",prob+-sd="100+-0"]:
8.016905e-01[&length_mean=8.11032494e-01,length_median=8.01690500e-0
1,length_95%HPD={5.51177900e-01,1.09087600e+00}],
167[&prob=1.00000000e+00,prob_stddev=0.00000000e+00,prob_range={1.00
000000e+00,1.00000000e+00},prob(percent)="100",prob+-sd="100+-0"]:

9.123147e-01[&length_mean=9.20506492e-01,length_median=9.12314700e-0
1,length_95%HPD={6.38255900e-01,1.21935600e+00}])
[&prob=7.79276477e-01,prob_stddev=6.19083756e-03,prob_range={7.74898
893e-01,7.83654060e-01},prob(percent)="78",prob+-sd="78+-1"]:
2.283273e-01[&length_mean=2.41258683e-01,length_median=2.28327300e-0
1,length_95%HPD={5.19012000e-02,4.52209500e-01}])
[&prob=8.71539043e-01,prob_stddev=2.04580470e-02,prob_range={8.57073
019e-01,8.86005066e-01},prob(percent)="87",prob+-sd="87+-2"]:
1.530314e-01[&length_mean=1.59367068e-01,length_median=1.53031400e-0
1,length_95%HPD={2.76843600e-02,2.94694100e-01}],
((((((((152[&prob=1.00000000e+00,prob_stddev=0.00000000e+00,prob_ran
ge={1.00000000e+00,1.00000000e+00},prob(percent)="100",prob+-
sd="100+-0"]:
9.084249e-02[&length_mean=9.31512650e-02,length_median=9.08424900e-0
2,length_95%HPD={4.40494200e-02,1.49992500e-01}],
154[&prob=1.00000000e+00,prob_stddev=0.00000000e+00,prob_range={1.00
000000e+00,1.00000000e+00},prob(percent)="100",prob+-sd="100+-0"]:
6.580062e-02[&length_mean=6.94228963e-02,length_median=6.58006200e-0
2,length_95%HPD={2.03278600e-02,1.25671100e-01}])
[&prob=9.98844496e-01,prob_stddev=1.13132057e-03,prob_range={9.98044
531e-01,9.99644460e-01},prob(percent)="100",prob+-sd="100+-0"]:
4.966893e-02[&length_mean=5.28273040e-02,length_median=4.96689300e-0
2,length_95%HPD={1.13613200e-02,9.70078600e-02}],
153[&prob=1.00000000e+00,prob_stddev=0.00000000e+00,prob_range={1.00
000000e+00,1.00000000e+00},prob(percent)="100",prob+-sd="100+-0"]:
6.030096e-02[&length_mean=6.23847896e-02,length_median=6.03009600e-0
2,length_95%HPD={2.14288100e-02,1.13876600e-01}])
[&prob=9.99355584e-01,prob_stddev=4.08532428e-04,prob_range={9.99066
708e-01,9.99644460e-01},prob(percent)="100",prob+-sd="100+-0"]:
1.042308e-01[&length_mean=1.07509660e-01,length_median=1.04230800e-0
1,length_95%HPD={3.73491400e-02,1.85587100e-01}],
(155[&prob=1.00000000e+00,prob_stddev=0.00000000e+00,prob_range={1.0
0000000e+00,1.00000000e+00},prob(percent)="100",prob+-sd="100+-0"]:
8.357247e-02[&length_mean=8.71807751e-02,length_median=8.35724700e-0
2,length_95%HPD={2.77402500e-02,1.57143600e-01}],
156[&prob=1.00000000e+00,prob_stddev=0.00000000e+00,prob_range={1.00
000000e+00,1.00000000e+00},prob(percent)="100",prob+-sd="100+-0"]:
1.129386e-01[&length_mean=1.16125385e-01,length_median=1.12938600e-0
1,length_95%HPD={5.13863400e-02,1.88207600e-01}])
[&prob=9.99244478e-01,prob_stddev=1.06846943e-03,prob_range={9.98488
956e-01,1.00000000e+00},prob(percent)="100",prob+-sd="100+-0"]:
1.265038e-01[&length_mean=1.29012033e-01,length_median=1.26503800e-0
1,length_95%HPD={4.35586300e-02,2.14969800e-01}])
[&prob=1.00000000e+00,prob_stddev=0.00000000e+00,prob_range={1.00000
000e+00,1.00000000e+00},prob(percent)="100",prob+-sd="100+-0"]:
1.819992e-01[&length_mean=1.85391601e-01,length_median=1.81999200e-0
1,length_95%HPD={7.34444300e-02,2.98106000e-01}],
157[&prob=1.00000000e+00,prob_stddev=0.00000000e+00,prob_range={1.00
000000e+00,1.00000000e+00},prob(percent)="100",prob+-sd="100+-0"]:
3.501850e-01[&length_mean=3.54909071e-01,length_median=3.50185000e-0
1,length_95%HPD={2.26471300e-01,4.91169200e-01}])
[&prob=9.98155638e-01,prob_stddev=2.35691785e-03,prob_range={9.96489
045e-01,9.99822230e-01},prob(percent)="100",prob+-sd="100+-0"]:
1.860749e-01[&length_mean=1.92280041e-01,length_median=1.86074900e-0

1,length_95%HPD={6.20388000e-02,3.24611000e-01}],
(163[&prob=1.00000000e+00,prob_stddev=0.00000000e+00,prob_range={1.0
0000000e+00,1.00000000e+00},prob(percent)="100",prob+-sd="100+-0"]:
4.609671e-01[&length_mean=4.67438739e-01,length_median=4.60967100e-0
1,length_95%HPD={2.71913000e-01,6.81348200e-01}],
164[&prob=1.00000000e+00,prob_stddev=0.00000000e+00,prob_range={1.00
000000e+00,1.00000000e+00},prob(percent)="100",prob+-sd="100+-0"]:
7.166671e-01[&length_mean=7.23236522e-01,length_median=7.16667100e-0
1,length_95%HPD={4.90074000e-01,9.55490400e-01}])
[&prob=8.36185058e-01,prob_stddev=4.71383570e-03,prob_range={8.32851
873e-01,8.39518244e-01},prob(percent)="84",prob+-sd="84+-0"]:
1.617695e-01[&length_mean=1.69359225e-01,length_median=1.61769500e-0
1,length_95%HPD={1.40677200e-02,3.36209800e-01}])
[&prob=6.08906271e-01,prob_stddev=4.33672885e-03,prob_range={6.05839
740e-01,6.11972801e-01},prob(percent)="61",prob+-sd="61+-0"]:
1.597219e-01[&length_mean=1.64607540e-01,length_median=1.59721900e-0
1,length_95%HPD={3.05995500e-02,3.02145600e-01}],
(158[&prob=1.00000000e+00,prob_stddev=0.00000000e+00,prob_range={1.0
0000000e+00,1.00000000e+00},prob(percent)="100",prob+-sd="100+-0"]:
3.413224e-01[&length_mean=3.43620861e-01,length_median=3.41322400e-0
1,length_95%HPD={2.13410400e-01,4.81996700e-01}],
(159[&prob=1.00000000e+00,prob_stddev=0.00000000e+00,prob_range={1.0
0000000e+00,1.00000000e+00},prob(percent)="100",prob+-sd="100+-0"]:
2.120192e-01[&length_mean=2.16195353e-01,length_median=2.12019200e-0
1,length_95%HPD={9.96484800e-02,3.50525200e-01}],
160[&prob=1.00000000e+00,prob_stddev=0.00000000e+00,prob_range={1.00
000000e+00,1.00000000e+00},prob(percent)="100",prob+-sd="100+-0"]:
1.486017e-01[&length_mean=1.54888843e-01,length_median=1.48601700e-0
1,length_95%HPD={4.35118100e-02,2.66993500e-01}])
[&prob=1.00000000e+00,prob_stddev=0.00000000e+00,prob_range={1.00000
000e+00,1.00000000e+00},prob(percent)="100",prob+-sd="100+-0"]:
3.417269e-01[&length_mean=3.46917752e-01,length_median=3.41726900e-0
1,length_95%HPD={1.89265700e-01,5.27638800e-01}])
[&prob=8.82494111e-01,prob_stddev=6.72507227e-03,prob_range={8.77738
767e-01,8.87249456e-01},prob(percent)="88",prob+-sd="88+-1"]:
1.491636e-01[&length_mean=1.54642722e-01,length_median=1.49163600e-0
1,length_95%HPD={3.10060300e-02,2.85076700e-01}])
[&prob=9.99200036e-01,prob_stddev=5.65660284e-04,prob_range={9.98800
053e-01,9.99600018e-01},prob(percent)="100",prob+-sd="100+-0"]:
2.396783e-01[&length_mean=2.45859223e-01,length_median=2.39678300e-0
1,length_95%HPD={8.71455800e-02,3.93935200e-01}],
161[&prob=1.00000000e+00,prob_stddev=0.00000000e+00,prob_range={1.00
000000e+00,1.00000000e+00},prob(percent)="100",prob+-sd="100+-0"]:
3.801495e-01[&length_mean=3.81851768e-01,length_median=3.80149500e-0
1,length_95%HPD={2.32798100e-01,5.54081100e-01}])
[&prob=9.54535354e-01,prob_stddev=2.76545028e-03,prob_range={9.52579
885e-01,9.56490823e-01},prob(percent)="95",prob+-sd="95+-0"]:
2.321508e-01[&length_mean=2.37575355e-01,length_median=2.32150800e-0
1,length_95%HPD={8.13812600e-02,4.27818800e-01}],
162[&prob=1.00000000e+00,prob_stddev=0.00000000e+00,prob_range={1.00
000000e+00,1.00000000e+00},prob(percent)="100",prob+-sd="100+-0"]:
5.671549e-01[&length_mean=5.71612533e-01,length_median=5.67154900e-0
1,length_95%HPD={3.62262700e-01,7.99646800e-01}])
[&prob=1.00000000e+00,prob_stddev=0.00000000e+00,prob_range={1.00000

000e+00,1.00000000e+00},prob(percent)="100",prob+-sd="100+-0"]:
3.833258e-01[&length_mean=3.92202436e-01,length_median=3.83325800e-0
1,length_95%HPD={2.07920100e-01,6.06700000e-01}],
(171[&prob=1.00000000e+00,prob_stddev=0.00000000e+00,prob_range={1.0
0000000e+00,1.00000000e+00},prob(percent)="100",prob+-sd="100+-0"]:
1.470650e+00[&length_mean=1.48553282e+00,length_median=1.47065000e+0
0,length_95%HPD={1.15967200e+00,1.82455100e+00}],
(172[&prob=1.00000000e+00,prob_stddev=0.00000000e+00,prob_range={1.0
0000000e+00,1.00000000e+00},prob(percent)="100",prob+-sd="100+-0"]:
1.068510e+00[&length_mean=1.08150542e+00,length_median=1.06851000e+0
0,length_95%HPD={8.35581900e-01,1.36964200e+00}],
(173[&prob=1.00000000e+00,prob_stddev=0.00000000e+00,prob_range={1.0
0000000e+00,1.00000000e+00},prob(percent)="100",prob+-sd="100+-0"]:
3.726772e-02[&length_mean=3.92850679e-02,length_median=3.72677200e-0
2,length_95%HPD={4.32499900e-03,7.74090800e-02}],
174[&prob=1.00000000e+00,prob_stddev=0.00000000e+00,prob_range={1.00
000000e+00,1.00000000e+00},prob(percent)="100",prob+-sd="100+-0"]:
5.800395e-02[&length_mean=5.82967651e-02,length_median=5.80039500e-0
2,length_95%HPD={1.94387700e-02,9.98537500e-02}])
[&prob=1.00000000e+00,prob_stddev=0.00000000e+00,prob_range={1.00000
000e+00,1.00000000e+00},prob(percent)="100",prob+-sd="100+-0"]:
1.110036e+00[&length_mean=1.11618040e+00,length_median=1.11003600e+0
0,length_95%HPD={8.61430900e-01,1.38965900e+00}])
[&prob=6.20372428e-01,prob_stddev=1.06846943e-03,prob_range={6.19616
906e-01,6.21127950e-01},prob(percent)="62",prob+-sd="62+-0"]:
1.725566e-01[&length_mean=1.87077975e-01,length_median=1.72556600e-0
1,length_95%HPD={8.56308900e-05,4.02930100e-01}])
[&prob=9.70779076e-01,prob_stddev=6.94505127e-03,prob_range={9.65868
184e-01,9.75689969e-01},prob(percent)="97",prob+-sd="97+-1"]:
3.180162e-01[&length_mean=3.22728541e-01,length_median=3.18016200e-0
1,length_95%HPD={8.43893500e-02,5.55760000e-01}])
[&prob=1.00000000e+00,prob_stddev=0.00000000e+00,prob_range={1.00000
000e+00,1.00000000e+00},prob(percent)="100",prob+-sd="100+-0"]:
3.168607e-01[&length_mean=3.18212712e-01,length_median=3.16860700e-0
1,length_95%HPD={1.54629000e-01,4.79711600e-01}])
[&prob=7.40544865e-01,prob_stddev=1.60898925e-02,prob_range={7.29167
593e-01,7.51922137e-01},prob(percent)="74",prob+-sd="74+-2"]:
9.765160e-02[&length_mean=1.00754360e-01,length_median=9.76516000e-0
2,length_95%HPD={6.00282000e-03,1.87820100e-01}])
[&prob=5.58486289e-01,prob_stddev=1.60584670e-02,prob_range={5.47131
239e-01,5.69841340e-01},prob(percent)="56",prob+-sd="56+-2"]:
5.459766e-02[&length_mean=5.94166893e-02,length_median=5.45976600e-0
2,length_95%HPD={3.00758200e-03,1.22615500e-01}])
[&prob=9.84467357e-01,prob_stddev=2.16836442e-03,prob_range={9.82934
092e-01,9.86000622e-01},prob(percent)="98",prob+-sd="98+-0"]:
1.158579e-01[&length_mean=1.18413538e-01,length_median=1.15857900e-0
1,length_95%HPD={4.11512900e-02,1.97729000e-01}],
((43[&prob=1.00000000e+00,prob_stddev=0.00000000e+00,prob_range={1.0
0000000e+00,1.00000000e+00},prob(percent)="100",prob+-sd="100+-0"]:
5.567936e-02[&length_mean=5.75319852e-02,length_median=5.56793600e-0
2,length_95%HPD={1.74105900e-02,1.01798800e-01}],
44[&prob=1.00000000e+00,prob_stddev=0.00000000e+00,prob_range={1.000
00000e+00,1.00000000e+00},prob(percent)="100",prob+-sd="100+-0"]:
2.669043e-02[&length_mean=2.97554745e-02,length_median=2.66904300e-0

2,length_95%HPD={5.79590900e-05,6.32127600e-02}])
[&prob=1.00000000e+00,prob_stddev=0.00000000e+00,prob_range={1.00000
000e+00,1.00000000e+00},prob(percent)="100",prob+-sd="100+-0"]:
2.983404e-01[&length_mean=2.99624104e-01,length_median=2.98340400e-0
1,length_95%HPD={2.02194600e-01,4.01730600e-01}],
(45[&prob=1.00000000e+00,prob_stddev=0.00000000e+00,prob_range={1.00
000000e+00,1.00000000e+00},prob(percent)="100",prob+-sd="100+-0"]:
1.464442e-01[&length_mean=1.48901717e-01,length_median=1.46444200e-0
1,length_95%HPD={8.15819200e-02,2.16994100e-01}],
46[&prob=1.00000000e+00,prob_stddev=0.00000000e+00,prob_range={1.000
00000e+00,1.00000000e+00},prob(percent)="100",prob+-sd="100+-0"]:
1.527634e-01[&length_mean=1.55557942e-01,length_median=1.52763400e-0
1,length_95%HPD={9.06591400e-02,2.30309400e-01}])
[&prob=9.96844585e-01,prob_stddev=1.06846943e-03,prob_range={9.96089
063e-01,9.97600107e-01},prob(percent)="100",prob+-sd="100+-0"]:
8.914773e-02[&length_mean=9.26828884e-02,length_median=8.91477300e-0
2,length_95%HPD={2.93422100e-02,1.61751800e-01}])
[&prob=1.00000000e+00,prob_stddev=0.00000000e+00,prob_range={1.00000
000e+00,1.00000000e+00},prob(percent)="100",prob+-sd="100+-0"]:
2.326017e-01[&length_mean=2.35311099e-01,length_median=2.32601700e-0
1,length_95%HPD={1.25923600e-01,3.31986800e-01}],
(56[&prob=1.00000000e+00,prob_stddev=0.00000000e+00,prob_range={1.00
000000e+00,1.00000000e+00},prob(percent)="100",prob+-sd="100+-0"]:
3.077457e-01[&length_mean=3.08502793e-01,length_median=3.07745700e-0
1,length_95%HPD={1.82216100e-01,4.39195700e-01}],
57[&prob=1.00000000e+00,prob_stddev=0.00000000e+00,prob_range={1.000
00000e+00,1.00000000e+00},prob(percent)="100",prob+-sd="100+-0"]:
4.752886e-01[&length_mean=4.78705054e-01,length_median=4.75288600e-0
1,length_95%HPD={3.37510900e-01,6.14232500e-01}])
[&prob=1.00000000e+00,prob_stddev=0.00000000e+00,prob_range={1.00000
000e+00,1.00000000e+00},prob(percent)="100",prob+-sd="100+-0"]:
2.897643e-01[&length_mean=2.92785792e-01,length_median=2.89764300e-0
1,length_95%HPD={1.69176900e-01,4.24722400e-01}])
[&prob=6.64814897e-01,prob_stddev=1.00561828e-03,prob_range={6.64103
818e-01,6.65525977e-01},prob(percent)="66",prob+-sd="66+-0"]:
5.226948e-02[&length_mean=5.58958068e-02,length_median=5.22694800e-0
2,length_95%HPD={8.48754700e-04,1.12868400e-01}],
((58[&prob=1.00000000e+00,prob_stddev=0.00000000e+00,prob_range={1.0
0000000e+00,1.00000000e+00},prob(percent)="100",prob+-sd="100+-0"]:
3.738516e-02[&length_mean=4.06721630e-02,length_median=3.73851600e-0
2,length_95%HPD={3.47577000e-03,8.28306300e-02}],
59[&prob=1.00000000e+00,prob_stddev=0.00000000e+00,prob_range={1.000
00000e+00,1.00000000e+00},prob(percent)="100",prob+-sd="100+-0"]:
3.278612e-02[&length_mean=3.52256234e-02,length_median=3.27861200e-0
2,length_95%HPD={1.49986100e-03,7.21025000e-02}])
[&prob=1.00000000e+00,prob_stddev=0.00000000e+00,prob_range={1.00000
000e+00,1.00000000e+00},prob(percent)="100",prob+-sd="100+-0"]:
4.974886e-01[&length_mean=4.99152464e-01,length_median=4.97488600e-0
1,length_95%HPD={3.50223600e-01,6.39553300e-01}],
60[&prob=1.00000000e+00,prob_stddev=0.00000000e+00,prob_range={1.000
00000e+00,1.00000000e+00},prob(percent)="100",prob+-sd="100+-0"]:
3.374217e-01[&length_mean=3.41522280e-01,length_median=3.37421700e-0
1,length_95%HPD={2.18295100e-01,4.77490600e-01}])
[&prob=1.00000000e+00,prob_stddev=0.00000000e+00,prob_range={1.00000

000e+00,1.00000000e+00},prob(percent)="100",prob+-sd="100+-0"]:
3.249449e-01[&length_mean=3.28404921e-01,length_median=3.24944900e-0
1,length_95%HPD={1.91991600e-01,4.59995400e-01}])
[&prob=1.00000000e+00,prob_stddev=0.00000000e+00,prob_range={1.00000
000e+00,1.00000000e+00},prob(percent)="100",prob+-sd="100+-0"]:
9.567374e-02[&length_mean=9.94561304e-02,length_median=9.56737400e-0
2,length_95%HPD={3.77915800e-02,1.72020600e-01}])
[&prob=9.96266833e-01,prob_stddev=3.14255714e-03,prob_range={9.94044
709e-01,9.98488956e-01},prob(percent)="100",prob+-sd="100+-0"]:
6.905715e-02[&length_mean=7.14859194e-02,length_median=6.90571500e-0
2,length_95%HPD={2.43094500e-02,1.27554700e-01}])
[&prob=1.00000000e+00,prob_stddev=0.00000000e+00,prob_range={1.00000
000e+00,1.00000000e+00},prob(percent)="100",prob+-sd="100+-0"]:
2.432124e-01[&length_mean=2.45343907e-01,length_median=2.43212400e-0
1,length_95%HPD={1.56754000e-01,3.34335500e-01}])
[&prob=9.86133950e-01,prob_stddev=6.15941199e-03,prob_range={9.81778
588e-01,9.90489312e-01},prob(percent)="99",prob+-sd="99+-1"]:
3.661042e-02[&length_mean=3.95077357e-02,length_median=3.66104200e-0
2,length_95%HPD={6.04864100e-03,7.91299200e-02}],
((16[&prob=1.00000000e+00,prob_stddev=0.00000000e+00,prob_range={1.0
0000000e+00,1.00000000e+00},prob(percent)="100",prob+-sd="100+-0"]:
2.036951e-02[&length_mean=2.25622172e-02,length_median=2.03695100e-0
2,length_95%HPD={1.38925600e-03,4.85109400e-02}],
17[&prob=1.00000000e+00,prob_stddev=0.00000000e+00,prob_range={1.000
00000e+00,1.00000000e+00},prob(percent)="100",prob+-sd="100+-0"]:
7.866907e-02[&length_mean=8.13346072e-02,length_median=7.86690700e-0
2,length_95%HPD={3.82947900e-02,1.34750700e-01}])
[&prob=1.00000000e+00,prob_stddev=0.00000000e+00,prob_range={1.00000
000e+00,1.00000000e+00},prob(percent)="100",prob+-sd="100+-0"]:
8.047446e-02[&length_mean=8.24059527e-02,length_median=8.04744600e-0
2,length_95%HPD={3.49069000e-02,1.30919900e-01}],
(22[&prob=1.00000000e+00,prob_stddev=0.00000000e+00,prob_range={1.00
000000e+00,1.00000000e+00},prob(percent)="100",prob+-sd="100+-0"]:
1.417719e-01[&length_mean=1.43277689e-01,length_median=1.41771900e-0
1,length_95%HPD={8.14510000e-02,2.06831900e-01}],
23[&prob=1.00000000e+00,prob_stddev=0.00000000e+00,prob_range={1.000
00000e+00,1.00000000e+00},prob(percent)="100",prob+-sd="100+-0"]:
1.172685e-01[&length_mean=1.20860191e-01,length_median=1.17268500e-0
1,length_95%HPD={6.45211800e-02,1.82715300e-01}])
[&prob=9.99977779e-01,prob_stddev=3.14255714e-05,prob_range={9.99955
558e-01,1.00000000e+00},prob(percent)="100",prob+-sd="100+-0"]:
1.044067e-01[&length_mean=1.06359636e-01,length_median=1.04406700e-0
1,length_95%HPD={4.00597100e-02,1.70767700e-01}])
[&prob=7.24834452e-01,prob_stddev=1.74003389e-01,prob_range={6.01795
476e-01,8.47873428e-01},prob(percent)="72",prob+-sd="72+-17"]:
6.464360e-02[&length_mean=6.71911580e-02,length_median=6.46436000e-0
2,length_95%HPD={2.51111300e-02,1.14007300e-01}])
[&prob=6.90280432e-01,prob_stddev=1.68063956e-01,prob_range={5.71441
269e-01,8.09119595e-01},prob(percent)="69",prob+-sd="69+-17"]:
3.723377e-02[&length_mean=3.98269733e-02,length_median=3.72337700e-0
2,length_95%HPD={6.79466200e-03,7.62144400e-02}])
[&prob=5.27665437e-01,prob_stddev=5.21664485e-02,prob_range={4.90778
188e-01,5.64552687e-01},prob(percent)="53",prob+-sd="53+-5"]:
4.664262e-02[&length_mean=4.94848137e-02,length_median=4.66426200e-0

```
2,length_95%HPD={7.77324100e-03,9.43124300e-02}],
((26[&prob=1.00000000e+00,prob_stddev=0.00000000e+00,prob_range={1.0
0000000e+00,1.00000000e+00},prob(percent)="100",prob+-sd="100+-0"]:
3.817533e-02[&length_mean=4.30779460e-02,length_median=3.81753300e-0
2,length_95%HPD={1.41709400e-03,9.39195900e-02}],
38[&prob=1.00000000e+00,prob_stddev=0.00000000e+00,prob_range={1.000
00000e+00,1.00000000e+00},prob(percent)="100",prob+-sd="100+-0"]:
3.814918e-01[&length_mean=3.83662717e-01,length_median=3.81491800e-0
1,length_95%HPD={2.71430600e-01,5.06756300e-01}])
[&prob=1.00000000e+00,prob_stddev=0.00000000e+00,prob_range={1.00000
000e+00,1.00000000e+00},prob(percent)="100",prob+-sd="100+-0"]:
1.952763e-01[&length_mean=1.97993097e-01,length_median=1.95276300e-0
1,length_95%HPD={1.16266100e-01,2.88571200e-01}],
(36[&prob=1.00000000e+00,prob_stddev=0.00000000e+00,prob_range={1.00
000000e+00,1.00000000e+00},prob(percent)="100",prob+-sd="100+-0"]:
1.103416e-01[&length_mean=1.14013546e-01,length_median=1.10341600e-0
1,length_95%HPD={5.72813500e-02,1.81169100e-01}],
37[&prob=1.00000000e+00,prob_stddev=0.00000000e+00,prob_range={1.000
00000e+00,1.00000000e+00},prob(percent)="100",prob+-sd="100+-0"]:
1.080384e-01[&length_mean=1.09994408e-01,length_median=1.08038400e-0
1,length_95%HPD={5.08888500e-02,1.67783800e-01}])
[&prob=1.00000000e+00,prob_stddev=0.00000000e+00,prob_range={1.00000
000e+00,1.00000000e+00},prob(percent)="100",prob+-sd="100+-0"]:
2.370300e-01[&length_mean=2.39086034e-01,length_median=2.37030000e-0
1,length_95%HPD={1.56037400e-01,3.32362700e-01}])
[&prob=9.99777788e-01,prob_stddev=1.25702285e-04,prob_range={9.99688
903e-01,9.99866673e-01},prob(percent)="100",prob+-sd="100+-0"]:
1.154583e-01[&length_mean=1.18169119e-01,length_median=1.15458300e-0
1,length_95%HPD={4.25174000e-02,1.89432200e-01}])
[&prob=6.94435803e-01,prob_stddev=1.78340117e-01,prob_range={5.68330
296e-01,8.20541309e-01},prob(percent)="69",prob+-sd="69+-18"]:
9.239179e-02[&length_mean=9.46074570e-02,length_median=9.23917900e-0
2,length_95%HPD={2.96822500e-02,1.59668400e-01}],
(24[&prob=1.00000000e+00,prob_stddev=0.00000000e+00,prob_range={1.00
000000e+00,1.00000000e+00},prob(percent)="100",prob+-sd="100+-0"]:
1.473986e-01[&length_mean=1.49308760e-01,length_median=1.47398600e-0
1,length_95%HPD={8.40044200e-02,2.15462500e-01}],
25[&prob=1.00000000e+00,prob_stddev=0.00000000e+00,prob_range={1.000
00000e+00,1.00000000e+00},prob(percent)="100",prob+-sd="100+-0"]:
8.088721e-02[&length_mean=8.37902545e-02,length_median=8.08872100e-0
2,length_95%HPD={3.15693900e-02,1.34697300e-01}])
[&prob=9.99955558e-01,prob_stddev=6.28511427e-05,prob_range={9.99911
115e-01,1.00000000e+00},prob(percent)="100",prob+-sd="100+-0"]:
1.154794e-01[&length_mean=1.24515495e-01,length_median=1.15479400e-0
1,length_95%HPD={3.71093300e-02,2.30426400e-01}])
[&prob=1.00000000e+00,prob_stddev=0.00000000e+00,prob_range={1.00000
000e+00,1.00000000e+00},prob(percent)="100",prob+-sd="100+-0"]:
2.440359e-01[&length_mean=2.47777319e-01,length_median=2.44035900e-0
1,length_95%HPD={1.53048400e-01,3.57067900e-01}])
[&prob=1.00000000e+00,prob_stddev=0.00000000e+00,prob_range={1.00000
000e+00,1.00000000e+00},prob(percent)="100",prob+-sd="100+-0"]:
3.893880e-01[&length_mean=3.95435146e-01,length_median=3.89388000e-0
1,length_95%HPD={2.75088800e-01,5.34732000e-01}]);
end;
```

Fig4B(Fig_S5)_RAxML_tree_file

(EPHA1_MOUSE:0.00772598212243390259,(((EPHA8_HUMAN:
0.13567512634608897004,Drer_41799.6:0.09003126204337996707):
0.11315331593022195933,((EPHA6_HUMAN:
0.03976811883703817302,Drer_121462.2:0.13987771496885650913):
0.11791442099094674723,
((((((Drer_99872.3:0.12218325030746528259,EPHA10_HUMAN:
0.13268977311773963446):0.11808513386490061825,
(Drer_93563.5:0.07525195284418356723,EPHA7_HUMAN:
0.01461767252936614289):0.07549683356413224022):
0.06437637405183230244,
((Pmar_2234.1:0.09012892720781204292,Pmar_9310.1:0.12869321468033226
297):0.05263445687474544582,
(((Drer_136066.1:0.01351610062463172966,EPHB1_HUMAN:
0.06744051774505480101):0.08921858906098872888,((EPHB2_HUMAN:
0.05876202869538513923,
(Drer_43755.7:0.01761197914892933050,Drer_112928.2:0.055401393965723
77195):0.03277320130870715192):
0.07439422146552604476,Pmar_7951.1:0.09923270076733328726):
0.02644801661082961039):0.02453949027348810083,((((EPHB6_HUMAN:
0.02981400226194756697,EPHB6_MOUSE:0.03263223764702117419):
0.50728250542894726127,Drer_113830.2:0.34670782896637364345):
0.38921825223511363756,(((EPHB4_HUMAN:
0.04846299622940982571,EPHB4_MOUSE:0.02394387619302120601):
0.29277527465369340698,
(Drer_135357.1:0.14790658717067786676,Drer_88414.3:0.141023501697386
58847):0.08384510805919810628):0.25876985016238979176,((CiEphc:
0.33540233852812290083,CiEphe:0.50041609221864424839):
0.29774609828594267302,(((CiEphf:0.96732568465787960310,
((Dpul_79285:0.01664264324741683218,
(Lith_3827:0.00000133037082675727,Lith_21051:0.02959412382269952305)
:0.06572521602280716191):0.43674154297277312242,
((Hpsi_234025:0.91407583365983302581,
(Tpol_7305:0.35688579727520985951,Ctel_198909:0.31215338736090320060
):0.08265181439608576541):
0.03557028650673909470,Lgig_105485:0.43841455661803668820):
0.13171742912193470931):0.19207431470258060080):
0.08463477845198984217,
((Skow_11098:0.40297957042835857999,Spur_027145:0.51850082363992688 8
83):0.19290340602731140263,
(BfEph1:0.12724073675825459118,BfEph2:0.09127709469820742549):
0.21086220638303826425):0.04432901776668858596):
0.08004742537976571037,(((Vmul_37755:0.41760258444592041061,
((Baby_41769:0.43472422810590494180,
(Patl_251869:0.28888995921115911969,
(Edun_188839:0.61661178341253797264,Mlei_02521a-PA:
0.52275790271741928716):0.29669864610850371633):
0.05843388932228175264):
0.28757994847336126876,Cast_22203:0.27512436695481318205):
0.08878565300237152569):0.72463415644400452909,
((((((Cnul_32703:0.06361518390999802708,Cnul_32701:0.109814425693447
01532):1.06448850914361425879,Pper_114995:1.20967806212904860352):

0.25340692917738155687,
((((Kvar_25216:0.32972587047193857579,Cele_137847:0.6106355788318139
6324):0.25007386198575509706,Lapi_88092:0.48880503556865861903):
0.30609503151135947219,(((Cpro_80288.25:0.20064527154442957091,
(Lapi_154083:0.19812515862473115136,
(Cele_48040:0.74342327640409777700,Kvar_222799:0.1662622540317533481
6):0.12989083763178671527):0.05807209194549815129):
0.13418182002661865893,((Cvar_1757:0.32423301474613219764,
((Cvar_19697:0.00000133037082675727,Cvar_19694:0.0490164224991214633
7):0.03646195019752547506,Cvar_19695:0.05254321723919303661):
0.03275088784050826651):0.00808350612989829770,
(Cvar_18894:0.21278402464288034834,Cvar_19692:0.10013819447976354815
):0.01557965261081616613):0.18922981872084007615):
0.31302677231378517320,
(Ifas_36548:0.23930933659211997555,Ifas_26594:0.19973173472617103408
):0.42754037975209230327):0.22566545850042254995):
0.17988831543639463573,(((((Aque_03862:0.04477244833261630197,
(((Aque_37706:0.07606743866222896722,
(Aque_16689:0.13976615886652490883,Aque_41463:0.02174688738096998766
):0.01080932323313750325):
0.00781274354091253745,Aque_37708:0.02930061344863396644):
0.00987425010559365816,
(Aque_32612:0.05084765647675226763,Aque_32613:0.03939356232417224463
):0.00000133037082675727):0.00976245642960047247):
0.02089805419733113678,Aque_12969:0.01100780467969331503):
0.39341264713747053916,
((Pfic_36041:0.23186215552732003520,Niph_54283:0.2985715419428455197
3):0.26270579016534839623,
(Xtes_94993:0.08146525805880208693,Xtes_94992:0.05858601259998975214
):0.20493474389223986787):0.15588344389514194321):
0.17061605052203840382,Aque_37707:0.51641305784647162369):
0.45396172794929573469,((Cpro_78852.34:0.62062110940908099543,
((((Lapi_126388:0.26113973598631695650,Kvar_137296:0.362702185375209
70692):0.15524422507146631856,
(((Cpro_75438.40:0.62245495652542448539,Lapi_159044:0.17619024925508
103285):0.11279642847387560955,(Lapi_108357:0.38632560167123630723,
(Cele_85965:0.36668008906540300318,Kvar_2233:0.46164230806637107651)
:0.11907835940839281430):0.04584846485409903305):
0.06630348453916944107,(Kvar_222743:0.36445469876334635195,
(Psub_14382:0.49213758167829307899,Psub_7666:0.47307514639716063964)
:0.20966647829271412018):0.11804987184482798601):
0.10869971340814890692):0.07755116271631620761,
((Cpro_59066.22:0.30358453331382517781,
(Cele_124391:0.29519405231465045691,Kvar_4564:0.24414448305345623491
):0.16480001769359384212):
0.14713635017547135875,Lapi_96494:0.33185765643388659418):
0.19672703583505884994):
0.09318077272134353550,Cvar_27357:0.81502038282624755805):
0.08511203211585423312):0.40675397580941080600,
(Slac_1485:0.64505077114638997848,
(Niph_32084:0.54883145995481075197,
(Hamb_7684:0.28408329736235460317,Aque_11657:0.17028622294858830610)
:0.36112926397968131420):0.38122622147737406095):
0.22866384150430990596):0.12636353882320577879):

0.07273220793585900845):0.07642813001630174852):
0.21393483991341996631,
((Aele_22617:0.61124280135293529437,Clat_102964:0.81383735110936572532):0.73815988373209695528,
(Tadh_53296:0.94633666734542554178,Tadh_56933:1.03164963651628993624):0.50568699005244488465):0.15308119391497376771):
0.04421300457783618187,
((Ccan_99557:0.71753189083908075485,Ocar_909:0.62981448778183279913):0.49306245335044868172,(Rfib_47059:0.70718623003614378142,
(((((Rfib_2729:0.22975561641252278955,Snux_119736:0.15935144119134839191):0.35903323833364464424,Avas_85009:0.39395425972127928738):
0.21201521746966411897,
(Hpop_15474:0.84664450156415005377,Avas_63694:0.49696704365352467603):0.10461015288042960214):0.13128484559263309261,
(Snux_109225:0.39864131813383413272,
((Avas_93988:0.08103267734178457027,Avas_93993:0.10988890017028488111):0.11737956547863996615,
((Avas_56846:0.08623811359457703163,Avas_56851:0.05786129200920350768):0.04178133599475705034,Avas_56854:0.05812177584724589202):
0.11548999596734774165):0.18217214978614293397):
0.21975422479302922363):
0.31419511619692208626,Avas_82832:0.38431479592989814531):
0.27041754640112181107):0.60759599356633364753):
0.07637078742887118410):0.19672439727125906606,
((Aspe_Peph_158325:1.90876866358455066219,
(Cowc_Peph_08089T0:1.26311954867180742390,
(Mbre_Peph_26435:0.05665985713682027830,Mbre_Peph_9190:0.03371605695170867140):1.45845058014813289127):0.21003209517611051704):
0.41274831565030722924,(((((Adig_04039:0.05884698888948868695,
(((Adig_12429:0.08180711376754246089,
((Adig_03916:0.10818561423138135791,Adig_20930:0.05244115127025471973):0.16808329657415907454,Adig_15481:0.15776565656291105522):
0.10783475060261786538):
0.04740140862671333999,Adig_00805:0.08935439342370034288):
0.03514096884955542066,
(Adig_12482:0.08867022751493232324,Adig_18229:0.11090241318164094364):0.02485621317858108942):0.03639104860130218388):
0.91737319858640331560,
(((Apal_612045:0.40172615831980873491,Apal_455194:0.47621754691336437570):0.31726470913816184893,
((Nvec_39124:0.08592200194167853855,Nvec_39123:0.02010449073831559533):0.69692411170281665100,Btue_32401:0.49070792594286483101):
0.06977766909301157117):0.16865246370878242410,
(((Apal_307048:0.73342847897005991609,Apal_215204:0.15994708114548467082):0.06750121052191401794,((((Apal_25755:0.30574577556084159280,
(Apal_215216:0.03749671388417953610,
(Apal_215213:0.00000133037082675727,Apal_215214:0.12670810520880410044):0.11478659069722273178):0.06407507585786985371):
0.02894642593332294109,(Apal_549316:0.18033611870747170980,
(Apal_215207:0.08588022996301947209,Apal_215220:0.03812673557870652902):0.10436814064554630899):0.03946168261879667155):
0.01972510537697402117,Apal_549317:0.11018680130831522146):
0.01662251638585820340,
(Apal_215219:0.04299272590617918971,Apal_215211:0.104882038521194861

92):0.1359580900928206437 2):0.01472275852445519007):
0.32203143700330172372,Hdig_11359:0.39248660803093676774):
0.17597255442461587349):0.23266048013037166076):
0.12223602636317938697,Pcar_315635:0.86009213662037964365):
0.26242477444755607507,(Adig_13891:0.75983879448685043290,
(Apal_405536:0.65728810358416756010,Nvec_40993:0.6339224615583904931
5):0.19918388669790201839):0.06881075264316560691):
0.17345537528770002544,
(Adig_18762:0.12404821742722470235,Adig_13888:0.15086911669578009776
):0.96739646840631077218):0.03856320139187419116):
0.11391526276618299529):0.15995155461427454369):
0.44795223846605652929,(CiEpha:0.43436490294547569491,(CiEphd:
0.15612877996788071533,CiEphb:0.17910056142620769948):
0.18569596252272341230):0.10226303207793857597):
0.04426879952107732469):0.07796352586174447319):
0.05107091095157275523):0.06283245888768497489):
0.06661419918961652975,(EPHB3_HUMAN:0.06366615184113387504,
(Drer_40208.7:0.11917051632284682283,Drer_140419.1:0.049833027233784
43128):0.03961819607399928722):0.10398963920212582479):
0.06573840167774801235):0.24532977751567408564):
0.03401777119936585936):0.02290182242443699473,
(((Drer_30606.8:0.07902181096504742874,Drer_96552.3:0.09954483188104
476521):0.14420619044168478395,(EPHA4_HUMAN:0.01103049541502670698,
(Drer_3161.9:0.10799543984129912622,Drer_123962.1:0.0309481028410832
2901):0.04390826838670799925):0.05042840787896684523):
0.08959111942012511209,(EPHA5_HUMAN:
0.04950568585096792179,Drer_134983.1:0.11654092792639482445):
0.12418972403729995613):0.03427587863557787079):
0.03365993929140648905,
((Pmar_7260.1:0.10786590868185862158,Pmar_10202.1:0.1026424341830979
1644):0.24884327956700008633,
(Pmar_131.1:0.40684094552372657150,Pmar_9793.1:0.0198882217036687319
9):0.20400713774806381040):0.12339832855355661878):
0.04796337810719879247,(EPHA3_HUMAN:
0.03200178406834831846,Drer_21706.11:0.08531829665922981143):
0.09670877363682674199):0.04670521970282938418):
0.06449197898118683159):0.26972026799171006095,((EPHA2_MOUSE:
0.05262070580498161981,EPHA2_HUMAN:0.00000133037082675727):
0.17308005897801306827,
(Drer_11069.6:0.32554543164346200212,Drer_44917.5:0.2768778308652229
4029):0.25799483224091340761):0.10975431822906887847):
0.38311418511516937002,EPHA1_HUMAN:0.08158879010241648633):0.0;

Fig4C(Fig_S6)_IQtree_tree_file

(EFNA1_HUMAN:0.1056183009,EFNA1_MOUSE:0.1092822581,
((Drer_12577.6:0.3309267704,Drer_50216.6:0.2939942618)100:0.36045997
60,((((EFNA2_HUMAN:0.4632782195,
((Drer_40277.7:0.3466566131,Drer_101723.3:0.2614355669)100:0.1534437
343,(EFNA5_HUMAN:0.1947998705,(Drer_136933.1:0.1802862904,
(Drer_111146.1:0.0000021980,Drer_116412.1:0.1969618676)100:0.2726721
519)100:0.0447104024)100:0.3754319010)99:0.1196569646)100:0.32625561
18,(EFNA3_HUMAN:0.3928015809,
(Drer_119988.2:0.2033309682,Drer_133669.1:0.1374030817)100:0.1888511

185)100:0.3557749499)95:0.1576572507,((EFNA4_HUMAN:
0.0952935528,EFNA4_MOUSE:
0.1802942451)100:0.6726495018,Drer_141804.1:0.8101039571)99:0.377865
4401)97:0.1623295549,((((((((EFNB1_HUMAN:
0.2369450471,Drer_24428.4:0.2902348533)100:0.3149282295,
(EFNB2_MOUSE:0.2021697741,
(Drer_10432.8:0.1921107005,Drer_79638.5:0.3990586296)100:0.187520365
1)100:0.1456283555)100:0.2331312781,((EFNB3_HUMAN:
0.4076605166,Pmar_8071.1:1.1131167291)99:0.2681394411,
(Drer_62002.6:0.1538842204,Drer_73969.3:0.2435664454)99:0.1371436748
)100:0.2727482483)100:0.4874270114,CiEfnB:
1.2424036198)100:0.1537511876,Skow_16956:1.0509011820)99:0.128241397
0,Spur_23757:1.1814700793)79:0.0857269291,
((BfEfn1:0.9912322949,BfEfn2:1.4236392442)100:0.2773431141,Skow_5664
:1.4413588856)92:0.2174249943)98:0.0422515314,
(((((Ctel_225002:0.9297989810,Lgig_171062:1.0156503107)99:0.19681352
18,
(Tpol_19734:0.0964662233,Tpol_19735:0.0741647007)100:0.8491142486)99
:0.1952339978,
(Hpsi_83841:0.0946592557,Hpsi_83847:0.0460168117)100:1.1440762341)93
:0.1904963706,
(Dpul_EFX72793:0.4965107785,Lith_21086:0.2609879907)100:0.7123383779
)75:0.1587082314,
((((((((Atet_66572:0.4409034868,Atet_41310:0.4681214411)100:0.283665
5579,Clat_97078:0.2372997355)100:0.3240551254,
((Aele_151942:0.2029147972,Aele_151940:0.0000021722)100:0.2392795303
,
(Aele_151938:0.2774170752,Nbij_52843:0.2095725524)100:0.2143593385)1
00:0.2830319662)99:0.1934438009,Pphy_29075:0.9288777656)99:0.3055425
132,((Holi_5987:0.1976412139,
(Hvul_1033087.1:0.0055868409,Hvul_1038805.1:0.1702622620)100:0.15583
85835)100:0.4252189344,Hvir_3164:0.2842794144)100:0.9386009334)99:0.
3870396176,
(Aaur_20911:0.7631396470,Pper_55548:0.8660443198)72:0.2900617354)80:
0.3173189639,
((((Ever_204:0.5884285733,Ever_1897:0.9006477473)100:0.8480603338,
((((((((Apal_398190:0.1370417025,Hdig_30557:0.2253011831)100:0.49327
87868,Nvec_EDO36327:0.5352001006)100:0.4231690876,Pcar_235347:0.8231
187357)48:0.0921879166,
((Apal_41158:0.4671021419,Adig_07747:1.1864928082)99:0.1806316129,Pc
ar_395755:1.0824822089)57:0.0923195043)47:0.1170033903,
((Nvec_EDO47804:0.8120295959,Apal_54304:0.9693063419)100:0.312587169
7,Adig_04407:1.3066895479)55:0.1342134203)56:0.0680853917,
(((((Apal_344094:0.1799442497,Hdig_22696:0.2431684418)100:0.21145803
69,
(Btue_29916:0.0079770869,Btue_30708:0.0603740712)100:0.3324133888)10
0:0.1396441624,Nvec_EDO44950:0.5477581595)100:0.3175206142,
((Apal_400465:0.5799355652,Hdig_5881:0.5392753470)99:0.1573743743,Bt
ue_23164:0.9053278633)99:0.4190335563)80:0.1085702647,
(Pcar_189454:0.6854357125,Adig_20275:0.9695015840)100:0.3813285312)7
8:0.1873510815)72:0.0743268027,
(((Apal_344111:0.6205213050,Hdig_22213:0.5618821497)94:0.1274876636,
Btue_12517:1.1118873381)100:0.6889561771,Pcar_215100:1.0928618873)68
:0.2289229934)72:0.1257700612,((Pcar_240721:0.9755914166,

(Adig_03889:0.7863754150,
(((Adig_06508:0.0459295473,Adig_10295:0.2148984453)98:0.0651444679,A
dig_20773:0.1542938875)98:0.0976062224,Adig_19031:0.0406407332)99:0.
2994371164)100:0.6097972962)99:0.4237747824,
((((Apal_288439:0.0956131285,Apal_288431:0.1939594816)100:0.14847032
88,Apal_8979:0.1110481741)100:0.4066611531,Hdig_4952:0.3974715200)99
:0.2157703462,
(Btue_9751:0.1563167574,Btue_9750:0.1144810192)100:0.6021733740)100:
0.5904514240)91:0.2685598945)50:0.0805410539)67:0.1361794840,
((Btue_28719:2.5395304333,(((((((Cvar_2778:0.5195476227,
(Cvar_12230:0.7151670857,
(Cvar_1084:0.6564465965,Cvar_8977:0.8185922846)100:0.1638879717)96:0
.1027263650)100:0.5605343525,
((Cpro_58774.15:0.7950541091,Lapi_57857:0.8576657123)100:0.345959314
8,Slac_8385:1.1224603935)99:0.4728193272)61:0.2226622834,
(Lapi_139996:1.0250560613,Kvar_23578:1.5120704236)73:0.1782308329)99
:0.3953021525,Spyx_Cu_120946:2.3657529280)86:0.1406398352,
(Ifas_6298:0.0509648946,Ifas_6301:0.0815392658)100:1.3041409394)96:0
.
1595090534,Ifas_Cu_19075:2.8607351066)64:0.1276500136,Mlei_03441:2.1
204520402)30:0.0000020532)53:0.0562808123,
((Snux_75316:1.7601881208,Paer_Azurin:1.9780813301)86:0.1600959147,
((Uper_Plastocyanin:1.4817708926,Tfer_Rusticyanin:
2.2459293248)99:0.7000079439,(Atha_Blue_copper:
0.8545251665,Aspe_Cu_472242:2.2701824364)96:0.3689576877)94:0.304465
3751)91:0.4147636509)58:0.1772458281)43:0.0637812467,
((Pper_140147:1.2002480131,
(((((((Atet_141600:0.6445820883,Clat_80728:0.2885787259)100:0.23189
24469,
(((Clat_48253:0.2763845804,Atet_192642:1.0531629948)100:0.2681339056
,
(Clat_28862:0.2911692649,Atet_40821:0.7352223498)100:0.1827475196)10
0:0.2869379112,Pphy_19559:0.7774100431)99:0.2640282494)96:0.06508191
43,
(Aele_79976:0.4147389756,Nbij_20083:0.4741908612)100:0.1532962803)10
0:0.2125940031,
((Aele_124411:0.3615959301,Nbij_143918:0.5330640711)100:0.3134391505
,
(Clat_120953:0.5516879937,Atet_88599:0.6722398896)93:0.0937627462)10
0:0.4196412973)94:0.3344219146,(Pphy_49294:0.9898851925,
((Holi_3973:0.2826565505,
(Hvul_1018918.1:0.0241108946,Hvul_1028415.1:0.1176187400)99:0.133304
3837)100:0.8078940822,(Hvul_1034474.1:1.3468093982,
(Holi_7726:0.1499940811,Hvul_1019657.1:0.4133918750)100:0.4864939259
)99:0.3143751522)99:0.1766354704)55:0.1149759512)98:0.2206004260,
(Pper_105129:0.1808964683,Pper_41140:0.2871963824)100:0.7476104717)9
2:0.1406569826,Pper_103077:1.0817310610)99:0.2123298487,
(((((Aele_7590:0.7656197185,Nbij_23770:0.9266464213)98:0.2860120858,
Clat_22532:0.8791259823)71:0.2046627536,
(Pphy_13023:0.2635338918,Pphy_14332:0.4805689779)100:0.2720478551)97
:0.1713742053,Nbij_57193:1.2382643851)96:0.1413366061,
(Holi_14653:0.1752451841,Hvul_1028689.1:0.1863147771)100:0.674995830
0)100:0.5317764522)79:0.0454830129)92:0.2936835911,
((Apal_89985:0.9605834998,Pcar_179493:0.7708444306)100:0.4886664512,

```
Ever_19722:2.2042632197)83:0.2693395991)80:0.2267493054)37:0.0769645
613)61:0.1060833774,
(((Ever_13730:1.4220057240,Hvul_1029169.1:1.8953137561)82:0.08924960
75,Ccan_Cu_114223:3.3173949992)56:0.0601983000,Aque_39602:1.85139574
52)42:0.1426442874)83:0.2543901467)96:0.1261491697)99:0.3686027273,
((CiEfnAa:1.7021491987,(CiEfnAc:0.6435362191,CiEfnAd:
0.3228414766)100:0.6323963547)91:0.2089611673,CiEfnAb:
1.1128595872)94:0.2447630891)99:0.7769565154)100:0.5191743386)100:0.
4303454634);
```

Fig4C(Fig_S6)_Mrbayes_tree_file

```
#NEXUS
[ID: 0325627600]
begin taxa;
        dimensions ntax=156;
        taxlabels
                EFNA1_HUMAN
                EFNA1_MOUSE
                Drer_12577_6
                Drer_50216_6
                EFNA2_HUMAN
                Drer_40277_7
                Drer_101723_3
                EFNA5_HUMAN
                Drer_136933_1
                Drer_111146_1
                Drer_116412_1
                EFNA3_HUMAN
                Drer_119988_2
                Drer_133669_1
                EFNA4_HUMAN
                EFNA4_MOUSE
                Drer_141804_1
                EFNB1_HUMAN
                Drer_24428_4
                EFNB2_MOUSE
                Drer_10432_8
                Drer_79638_5
                EFNB3_HUMAN
                Drer_62002_6
                Drer_73969_3
                Pmar_8071_1
                Skow_16956
                CiEfnB
                BfEfn1
                Ctel_225002
                Lgig_171062
                Tpol_19734
                Tpol_19735
                Hpsi_83841
                Hpsi_83847
                Dpul_EFX72793
                Lith_21086
```

Spur_23757
Skow_5664
BfEfn2
Atet_66572
Atet_41310
Aele_151942
Aele_151940
Aele_151938
Nbij_52843
Clat_97078
Pphy_29075
Holi_5987
Hvul_1033087_1
Hvul_1038805_1
Hvir_3164
Ever_204
Ever_1897
Apal_398190
Hdig_30557
Nvec_EDO36327
Apal_41158
Apal_344094
Hdig_22696
Btue_29916
Btue_30708
Nvec_EDO44950
Apal_400465
Hdig_5881
Pcar_235347
Btue_23164
Nvec_EDO47804
Pcar_189454
Adig_20275
Apal_344111
Hdig_22213
Btue_12517
Pper_140147
Apal_54304
Adig_04407
Adig_07747
Pcar_215100
Pcar_395755
Pcar_240721
Adig_03889
Adig_06508
Adig_10295
Adig_20773
Adig_19031
Apal_288439
Apal_8979
Apal_288431
Hdig_4952
Btue_9751
Btue_9750

Aaur_20911
Pper_55548
Ever_13730
Atet_141600
Aele_79976
Nbij_20083
Clat_80728
Clat_48253
Clat_28862
Atet_192642
Pphy_49294
Pphy_19559
Aele_124411
Nbij_143918
Clat_120953
Atet_40821
Holi_3973
Hvul_1018918_1
Hvul_1028415_1
Atet_88599
Hvul_1034474_1
Pper_105129
Pper_41140
Holi_7726
Hvul_1019657_1
Pper_103077
Apal_89985
Pcar_179493
Aele_7590
Nbij_23770
Clat_22532
Pphy_13023
Pphy_14332
Holi_14653
Hvul_1028689_1
Nbij_57193
CiEfnAa
CiEfnAb
CiEfnAc
CiEfnAd
Btue_28719
Cvar_2778
Cvar_12230
Cvar_1084
Cvar_8977
Cpro_58774_15
Lapi_57857
Slac_8385
Lapi_139996
Ifas_6298
Ifas_6301
Kvar_23578
Ever_19722
Hvul_1029169_1

```
                 Mlei_03441
                 Aque_39602
                 Snux_75316
                 Spyx_Cu_120946
                 Uper_Plastocyanin
                 Paer_Azurin
                 Tfer_Rusticyanin
                 Atha_Blue_copper
                 Ifas_Cu_19075
                 Aspe_Cu_472242
                 Ccan_Cu_114223
                 ;
end;
begin trees;
        translate
                 1        EFNA1_HUMAN,
                 2        EFNA1_MOUSE,
                 3        Drer_12577_6,
                 4        Drer_50216_6,
                 5        EFNA2_HUMAN,
                 6        Drer_40277_7,
                 7        Drer_101723_3,
                 8        EFNA5_HUMAN,
                 9        Drer_136933_1,
                 10       Drer_111146_1,
                 11       Drer_116412_1,
                 12       EFNA3_HUMAN,
                 13       Drer_119988_2,
                 14       Drer_133669_1,
                 15       EFNA4_HUMAN,
                 16       EFNA4_MOUSE,
                 17       Drer_141804_1,
                 18       EFNB1_HUMAN,
                 19       Drer_24428_4,
                 20       EFNB2_MOUSE,
                 21       Drer_10432_8,
                 22       Drer_79638_5,
                 23       EFNB3_HUMAN,
                 24       Drer_62002_6,
                 25       Drer_73969_3,
                 26       Pmar_8071_1,
                 27       Skow_16956,
                 28       CiEfnB,
                 29       BfEfn1,
                 30       Ctel_225002,
                 31       Lgig_171062,
                 32       Tpol_19734,
                 33       Tpol_19735,
                 34       Hpsi_83841,
                 35       Hpsi_83847,
                 36       Dpul_EFX72793,
                 37       Lith_21086,
                 38       Spur_23757,
                 39       Skow_5664,
```

40 BfEfn2,
41 Atet_66572,
42 Atet_41310,
43 Aele_151942,
44 Aele_151940,
45 Aele_151938,
46 Nbij_52843,
47 Clat_97078,
48 Pphy_29075,
49 Holi_5987,
50 Hvul_1033087_1,
51 Hvul_1038805_1,
52 Hvir_3164,
53 Ever_204,
54 Ever_1897,
55 Apal_398190,
56 Hdig_30557,
57 Nvec_EDO36327,
58 Apal_41158,
59 Apal_344094,
60 Hdig_22696,
61 Btue_29916,
62 Btue_30708,
63 Nvec_EDO44950,
64 Apal_400465,
65 Hdig_5881,
66 Pcar_235347,
67 Btue_23164,
68 Nvec_EDO47804,
69 Pcar_189454,
70 Adig_20275,
71 Apal_344111,
72 Hdig_22213,
73 Btue_12517,
74 Pper_140147,
75 Apal_54304,
76 Adig_04407,
77 Adig_07747,
78 Pcar_215100,
79 Pcar_395755,
80 Pcar_240721,
81 Adig_03889,
82 Adig_06508,
83 Adig_10295,
84 Adig_20773,
85 Adig_19031,
86 Apal_288439,
87 Apal_8979,
88 Apal_288431,
89 Hdig_4952,
90 Btue_9751,
91 Btue_9750,
92 Aaur_20911,
93 Pper_55548,

94 Ever_13730,
95 Atet_141600,
96 Aele_79976,
97 Nbij_20083,
98 Clat_80728,
99 Clat_48253,
100 Clat_28862,
101 Atet_192642,
102 Pphy_49294,
103 Pphy_19559,
104 Aele_124411,
105 Nbij_143918,
106 Clat_120953,
107 Atet_40821,
108 Holi_3973,
109 Hvul_1018918_1,
110 Hvul_1028415_1,
111 Atet_88599,
112 Hvul_1034474_1,
113 Pper_105129,
114 Pper_41140,
115 Holi_7726,
116 Hvul_1019657_1,
117 Pper_103077,
118 Apal_89985,
119 Pcar_179493,
120 Aele_7590,
121 Nbij_23770,
122 Clat_22532,
123 Pphy_13023,
124 Pphy_14332,
125 Holi_14653,
126 Hvul_1028689_1,
127 Nbij_57193,
128 CiEfnAa,
129 CiEfnAb,
130 CiEfnAc,
131 CiEfnAd,
132 Btue_28719,
133 Cvar_2778,
134 Cvar_12230,
135 Cvar_1084,
136 Cvar_8977,
137 Cpro_58774_15,
138 Lapi_57857,
139 Slac_8385,
140 Lapi_139996,
141 Ifas_6298,
142 Ifas_6301,
143 Kvar_23578,
144 Ever_19722,
145 Hvul_1029169_1,
146 Mlei_03441,
147 Aque_39602,

```
                148     Snux_75316,
                149     Spyx_Cu_120946,
                150     Uper_Plastocyanin,
                151     Paer_Azurin,
                152     Tfer_Rusticyanin,
                153     Atha_Blue_copper,
                154     Ifas_Cu_19075,
                155     Aspe_Cu_472242,
                156     Ccan_Cu_114223
                ;
   tree con_50_majrule = [&U]
(1[&prob=1.00000000e+00,prob_stddev=0.00000000e+00,prob_range={1.000
00000e+00,1.00000000e+00},prob(percent)="100",prob+-sd="100+-0"]:
7.605345e-02[&length_mean=7.76887076e-02,length_median=7.60534500e-0
2,length_95%HPD={2.76676800e-02,1.27580700e-01}],
2[&prob=1.00000000e+00,prob_stddev=0.00000000e+00,prob_range={1.0000
0000e+00,1.00000000e+00},prob(percent)="100",prob+-sd="100+-0"]:
8.748495e-02[&length_mean=8.97710180e-02,length_median=8.74849500e-0
2,length_95%HPD={3.75161500e-02,1.43210300e-01}],
((3[&prob=1.00000000e+00,prob_stddev=0.00000000e+00,prob_range={1.00
000000e+00,1.00000000e+00},prob(percent)="100",prob+-sd="100+-0"]:
2.388186e-01[&length_mean=2.42813941e-01,length_median=2.38818600e-0
1,length_95%HPD={1.53301200e-01,3.43264900e-01}],
4[&prob=1.00000000e+00,prob_stddev=0.00000000e+00,prob_range={1.0000
0000e+00,1.00000000e+00},prob(percent)="100",prob+-sd="100+-0"]:
2.278974e-01[&length_mean=2.30137777e-01,length_median=2.27897400e-0
1,length_95%HPD={1.35742100e-01,3.30494300e-01}])
[&prob=1.00000000e+00,prob_stddev=0.00000000e+00,prob_range={1.00000
000e+00,1.00000000e+00},prob(percent)="100",prob+-sd="100+-0"]:
2.465403e-01[&length_mean=2.49819876e-01,length_median=2.46540300e-0
1,length_95%HPD={1.38715100e-01,3.73949300e-01}],
((((5[&prob=1.00000000e+00,prob_stddev=0.00000000e+00,prob_range={1.
00000000e+00,1.00000000e+00},prob(percent)="100",prob+-sd="100+-0"]:
3.238383e-01[&length_mean=3.26563921e-01,length_median=3.23838300e-0
1,length_95%HPD={2.19991400e-01,4.36107700e-01}],
((6[&prob=1.00000000e+00,prob_stddev=0.00000000e+00,prob_range={1.00
000000e+00,1.00000000e+00},prob(percent)="100",prob+-sd="100+-0"]:
2.411887e-01[&length_mean=2.41881289e-01,length_median=2.41188700e-0
1,length_95%HPD={1.51187700e-01,3.34479800e-01}],
7[&prob=1.00000000e+00,prob_stddev=0.00000000e+00,prob_range={1.0000
0000e+00,1.00000000e+00},prob(percent)="100",prob+-sd="100+-0"]:
1.757828e-01[&length_mean=1.77633124e-01,length_median=1.75782800e-0
1,length_95%HPD={9.52227100e-02,2.64393600e-01}])
[&prob=9.86533932e-01,prob_stddev=7.35358370e-03,prob_range={9.81334
163e-01,9.91733701e-01},prob(percent)="99",prob+-sd="99+-1"]:
9.619703e-02[&length_mean=9.90265137e-02,length_median=9.61970300e-0
2,length_95%HPD={2.71213900e-02,1.75268700e-01}],
(8[&prob=1.00000000e+00,prob_stddev=0.00000000e+00,prob_range={1.000
00000e+00,1.00000000e+00},prob(percent)="100",prob+-sd="100+-0"]:
1.552755e-01[&length_mean=1.56692307e-01,length_median=1.55275500e-0
1,length_95%HPD={9.29477500e-02,2.27045200e-01}],
(9[&prob=1.00000000e+00,prob_stddev=0.00000000e+00,prob_range={1.000
00000e+00,1.00000000e+00},prob(percent)="100",prob+-sd="100+-0"]:
1.351043e-01[&length_mean=1.37126036e-01,length_median=1.35104300e-0
```

1,length_95%HPD={7.66312500e-02,2.00664600e-01}],
(10[&prob=1.00000000e+00,prob_stddev=0.00000000e+00,prob_range={1.00
000000e+00,1.00000000e+00},prob(percent)="100",prob+-sd="100+-0"]:
8.983652e-03[&length_mean=1.09458590e-02,length_median=8.98365200e-0
3,length_95%HPD={1.90105000e-05,2.78337300e-02}],
11[&prob=1.00000000e+00,prob_stddev=0.00000000e+00,prob_range={1.000
00000e+00,1.00000000e+00},prob(percent)="100",prob+-sd="100+-0"]:
1.458694e-01[&length_mean=1.47491439e-01,length_median=1.45869400e-0
1,length_95%HPD={9.90863500e-02,1.97591100e-01}])
[&prob=9.99977779e-01,prob_stddev=3.14255714e-05,prob_range={9.99955
558e-01,1.00000000e+00},prob(percent)="100",prob+-sd="100+-0"]:
1.962635e-01[&length_mean=1.98460063e-01,length_median=1.96263500e-0
1,length_95%HPD={1.30323800e-01,2.71400900e-01}])
[&prob=8.94004711e-01,prob_stddev=7.35358370e-03,prob_range={8.88804
942e-01,8.99204480e-01},prob(percent)="89",prob+-sd="89+-1"]:
4.179552e-02[&length_mean=4.53176979e-02,length_median=4.17955200e-0
2,length_95%HPD={2.95515900e-03,9.71393700e-02}])
[&prob=9.99977779e-01,prob_stddev=3.14255714e-05,prob_range={9.99955
558e-01,1.00000000e+00},prob(percent)="100",prob+-sd="100+-0"]:
2.681244e-01[&length_mean=2.70160277e-01,length_median=2.68124400e-0
1,length_95%HPD={1.75448100e-01,3.67738300e-01}])
[&prob=9.70401315e-01,prob_stddev=1.06846943e-03,prob_range={9.69645
794e-01,9.71156837e-01},prob(percent)="97",prob+-sd="97+-0"]:
9.199054e-02[&length_mean=9.59347255e-02,length_median=9.19905400e-0
2,length_95%HPD={1.98221100e-02,1.75706600e-01}])
[&prob=9.99977779e-01,prob_stddev=3.14255714e-05,prob_range={9.99955
558e-01,1.00000000e+00},prob(percent)="100",prob+-sd="100+-0"]:
2.303090e-01[&length_mean=2.34382178e-01,length_median=2.30309000e-0
1,length_95%HPD={1.27629200e-01,3.49231300e-01}],
(12[&prob=1.00000000e+00,prob_stddev=0.00000000e+00,prob_range={1.00
000000e+00,1.00000000e+00},prob(percent)="100",prob+-sd="100+-0"]:
3.079317e-01[&length_mean=3.08644692e-01,length_median=3.07931700e-0
1,length_95%HPD={2.12233200e-01,4.08962400e-01}],
(13[&prob=1.00000000e+00,prob_stddev=0.00000000e+00,prob_range={1.00
000000e+00,1.00000000e+00},prob(percent)="100",prob+-sd="100+-0"]:
1.535150e-01[&length_mean=1.55995304e-01,length_median=1.53515000e-0
1,length_95%HPD={9.67818400e-02,2.25473400e-01}],
14[&prob=1.00000000e+00,prob_stddev=0.00000000e+00,prob_range={1.000
00000e+00,1.00000000e+00},prob(percent)="100",prob+-sd="100+-0"]:
1.064364e-01[&length_mean=1.08373579e-01,length_median=1.06436400e-0
1,length_95%HPD={5.68219200e-02,1.62225400e-01}])
[&prob=9.99044487e-01,prob_stddev=9.74192712e-04,prob_range={9.98355
629e-01,9.99733345e-01},prob(percent)="100",prob+-sd="100+-0"]:
1.219565e-01[&length_mean=1.24927334e-01,length_median=1.21956500e-0
1,length_95%HPD={4.93067400e-02,2.10016200e-01}])
[&prob=1.00000000e+00,prob_stddev=0.00000000e+00,prob_range={1.00000
000e+00,1.00000000e+00},prob(percent)="100",prob+-sd="100+-0"]:
2.588627e-01[&length_mean=2.60346043e-01,length_median=2.58862700e-0
1,length_95%HPD={1.50440900e-01,3.63728500e-01}])
[&prob=7.70210213e-01,prob_stddev=2.19979000e-04,prob_range={7.70054
664e-01,7.70365762e-01},prob(percent)="77",prob+-sd="77+-0"]:
1.102617e-01[&length_mean=1.15595233e-01,length_median=1.10261700e-0
1,length_95%HPD={1.95253400e-02,2.21602900e-01}],
((15[&prob=1.00000000e+00,prob_stddev=0.00000000e+00,prob_range={1.0

0000000e+00,1.00000000e+00},prob(percent)="100",prob+-sd="100+-0"]:
8.447254e-02[&length_mean=8.69599774e-02,length_median=8.44725400e-0
2,length_95%HPD={2.38153800e-02,1.49595500e-01}],
16[&prob=1.00000000e+00,prob_stddev=0.00000000e+00,prob_range={1.000
00000e+00,1.00000000e+00},prob(percent)="100",prob+-sd="100+-0"]:
1.193844e-01[&length_mean=1.21653236e-01,length_median=1.19384400e-0
1,length_95%HPD={5.10772900e-02,1.92438900e-01}])
[&prob=1.00000000e+00,prob_stddev=0.00000000e+00,prob_range={1.00000
000e+00,1.00000000e+00},prob(percent)="100",prob+-sd="100+-0"]:
4.457190e-01[&length_mean=4.48523190e-01,length_median=4.45719000e-0
1,length_95%HPD={2.92095500e-01,6.04740300e-01}],
17[&prob=1.00000000e+00,prob_stddev=0.00000000e+00,prob_range={1.000
00000e+00,1.00000000e+00},prob(percent)="100",prob+-sd="100+-0"]:
5.175293e-01[&length_mean=5.22329674e-01,length_median=5.17529300e-0
1,length_95%HPD={3.55489100e-01,7.00807400e-01}])
[&prob=9.97044576e-01,prob_stddev=1.28844843e-03,prob_range={9.96133
505e-01,9.97955646e-01},prob(percent)="100",prob+-sd="100+-0"]:
2.565524e-01[&length_mean=2.58971187e-01,length_median=2.56552400e-0
1,length_95%HPD={1.18645100e-01,3.97950000e-01}])
[&prob=9.77112128e-01,prob_stddev=4.58813342e-03,prob_range={9.73867
828e-01,9.80356429e-01},prob(percent)="98",prob+-sd="98+-0"]:
1.588202e-01[&length_mean=1.65165166e-01,length_median=1.58820200e-0
1,length_95%HPD={4.86975700e-02,2.90935900e-01}],
(((((((18[&prob=1.00000000e+00,prob_stddev=0.00000000e+00,prob_range
={1.00000000e+00,1.00000000e+00},prob(percent)="100",prob+-
sd="100+-0"]:
1.946758e-01[&length_mean=1.96413592e-01,length_median=1.94675800e-0
1,length_95%HPD={1.36450000e-01,2.65288000e-01}],
19[&prob=1.00000000e+00,prob_stddev=0.00000000e+00,prob_range={1.000
00000e+00,1.00000000e+00},prob(percent)="100",prob+-sd="100+-0"]:
2.255099e-01[&length_mean=2.27201137e-01,length_median=2.25509900e-0
1,length_95%HPD={1.61893600e-01,2.96715900e-01}])
[&prob=1.00000000e+00,prob_stddev=0.00000000e+00,prob_range={1.00000
000e+00,1.00000000e+00},prob(percent)="100",prob+-sd="100+-0"]:
2.449432e-01[&length_mean=2.47893980e-01,length_median=2.44943200e-0
1,length_95%HPD={1.71356600e-01,3.32944500e-01}],
(20[&prob=1.00000000e+00,prob_stddev=0.00000000e+00,prob_range={1.00
000000e+00,1.00000000e+00},prob(percent)="100",prob+-sd="100+-0"]:
1.668977e-01[&length_mean=1.67526535e-01,length_median=1.66897700e-0
1,length_95%HPD={1.10595700e-01,2.26996500e-01}],
(21[&prob=1.00000000e+00,prob_stddev=0.00000000e+00,prob_range={1.00
000000e+00,1.00000000e+00},prob(percent)="100",prob+-sd="100+-0"]:
1.603592e-01[&length_mean=1.61856670e-01,length_median=1.60359200e-0
1,length_95%HPD={1.04727000e-01,2.18237500e-01}],
22[&prob=1.00000000e+00,prob_stddev=0.00000000e+00,prob_range={1.000
00000e+00,1.00000000e+00},prob(percent)="100",prob+-sd="100+-0"]:
3.070944e-01[&length_mean=3.08300962e-01,length_median=3.07094400e-0
1,length_95%HPD={2.34040400e-01,3.80235400e-01}])
[&prob=1.00000000e+00,prob_stddev=0.00000000e+00,prob_range={1.00000
000e+00,1.00000000e+00},prob(percent)="100",prob+-sd="100+-0"]:
1.431347e-01[&length_mean=1.45112267e-01,length_median=1.43134700e-0
1,length_95%HPD={8.74587600e-02,2.03608000e-01}])
[&prob=1.00000000e+00,prob_stddev=0.00000000e+00,prob_range={1.00000
000e+00,1.00000000e+00},prob(percent)="100",prob+-sd="100+-0"]:

1.142940e-01[&length_mean=1.16867482e-01,length_median=1.14294000e-0
1,length_95%HPD={5.77236400e-02,1.83439800e-01}])
[&prob=1.00000000e+00,prob_stddev=0.00000000e+00,prob_range={1.00000
000e+00,1.00000000e+00},prob(percent)="100",prob+-sd="100+-0"]:
1.842150e-01[&length_mean=1.85885303e-01,length_median=1.84215000e-0
1,length_95%HPD={9.74667400e-02,2.65654600e-01}],
((23[&prob=1.00000000e+00,prob_stddev=0.00000000e+00,prob_range={1.0
0000000e+00,1.00000000e+00},prob(percent)="100",prob+-sd="100+-0"]:
3.264486e-01[&length_mean=3.29738763e-01,length_median=3.26448600e-0
1,length_95%HPD={1.61232800e-01,5.09678500e-01}],
26[&prob=1.00000000e+00,prob_stddev=0.00000000e+00,prob_range={1.000
00000e+00,1.00000000e+00},prob(percent)="100",prob+-sd="100+-0"]:
5.634075e-01[&length_mean=5.71157677e-01,length_median=5.63407500e-0
1,length_95%HPD={4.04141800e-01,7.64034600e-01}])
[&prob=9.45713524e-01,prob_stddev=2.68060124e-02,prob_range={9.26758
811e-01,9.64668237e-01},prob(percent)="95",prob+-sd="95+-3"]:
1.931210e-01[&length_mean=1.96897940e-01,length_median=1.93121000e-0
1,length_95%HPD={4.08438600e-02,3.49307500e-01}],
(24[&prob=1.00000000e+00,prob_stddev=0.00000000e+00,prob_range={1.00
000000e+00,1.00000000e+00},prob(percent)="100",prob+-sd="100+-0"]:
1.287164e-01[&length_mean=1.29589920e-01,length_median=1.28716400e-0
1,length_95%HPD={7.90547700e-02,1.85986400e-01}],
25[&prob=1.00000000e+00,prob_stddev=0.00000000e+00,prob_range={1.000
00000e+00,1.00000000e+00},prob(percent)="100",prob+-sd="100+-0"]:
1.982271e-01[&length_mean=1.98882245e-01,length_median=1.98227100e-0
1,length_95%HPD={1.42595000e-01,2.66314800e-01}])
[&prob=9.99466690e-01,prob_stddev=3.14255714e-04,prob_range={9.99244
478e-01,9.99688903e-01},prob(percent)="100",prob+-sd="100+-0"]:
1.180187e-01[&length_mean=1.20743382e-01,length_median=1.18018700e-0
1,length_95%HPD={5.25123200e-02,1.91872200e-01}])
[&prob=9.99822230e-01,prob_stddev=1.25702285e-04,prob_range={9.99733
345e-01,9.99911115e-01},prob(percent)="100",prob+-sd="100+-0"]:
1.971399e-01[&length_mean=1.98296558e-01,length_median=1.97139900e-0
1,length_95%HPD={1.10786300e-01,2.96825900e-01}])
[&prob=1.00000000e+00,prob_stddev=0.00000000e+00,prob_range={1.00000
000e+00,1.00000000e+00},prob(percent)="100",prob+-sd="100+-0"]:
3.717164e-01[&length_mean=3.73730520e-01,length_median=3.71716400e-0
1,length_95%HPD={2.40805600e-01,5.12159100e-01}],
28[&prob=1.00000000e+00,prob_stddev=0.00000000e+00,prob_range={1.000
00000e+00,1.00000000e+00},prob(percent)="100",prob+-sd="100+-0"]:
8.355423e-01[&length_mean=8.41234405e-01,length_median=8.35542300e-0
1,length_95%HPD={6.73595900e-01,1.01883200e+00}])
[&prob=9.00359984e-01,prob_stddev=6.97647684e-03,prob_range={8.95426
870e-01,9.05293098e-01},prob(percent)="90",prob+-sd="90+-1"]:
1.369713e-01[&length_mean=1.41047623e-01,length_median=1.36971300e-0
1,length_95%HPD={9.93230900e-03,2.67649700e-01}],
27[&prob=1.00000000e+00,prob_stddev=0.00000000e+00,prob_range={1.000
00000e+00,1.00000000e+00},prob(percent)="100",prob+-sd="100+-0"]:
5.449869e-01[&length_mean=5.48186641e-01,length_median=5.44986900e-0
1,length_95%HPD={3.76811200e-01,7.21556300e-01}])
[&prob=9.10959513e-01,prob_stddev=3.67993441e-02,prob_range={8.84938
447e-01,9.36980579e-01},prob(percent)="91",prob+-sd="91+-4"]:
1.582349e-01[&length_mean=1.59175369e-01,length_median=1.58234900e-0
1,length_95%HPD={4.06570900e-02,2.77062800e-01}],

((29[&prob=1.00000000e+00,prob_stddev=0.00000000e+00,prob_range={1.0
0000000e+00,1.00000000e+00},prob(percent)="100",prob+-sd="100+-0"]:
6.320214e-01[&length_mean=6.33918318e-01,length_median=6.32021400e-0
1,length_95%HPD={4.52304600e-01,8.20570300e-01}],
40[&prob=1.00000000e+00,prob_stddev=0.00000000e+00,prob_range={1.000
00000e+00,1.00000000e+00},prob(percent)="100",prob+-sd="100+-0"]:
8.434233e-01[&length_mean=8.45600349e-01,length_median=8.43423300e-0
1,length_95%HPD={6.54898300e-01,1.08438800e+00}])
[&prob=9.97444558e-01,prob_stddev=7.85639284e-04,prob_range={9.96889
027e-01,9.98000089e-01},prob(percent)="100",prob+-sd="100+-0"]:
3.059267e-01[&length_mean=3.13142357e-01,length_median=3.05926700e-0
1,length_95%HPD={1.06287800e-01,5.07416100e-01}],
(38[&prob=1.00000000e+00,prob_stddev=0.00000000e+00,prob_range={1.00
000000e+00,1.00000000e+00},prob(percent)="100",prob+-sd="100+-0"]:
6.359610e-01[&length_mean=6.36552606e-01,length_median=6.35961000e-0
1,length_95%HPD={4.75428900e-01,8.15987100e-01}],
39[&prob=1.00000000e+00,prob_stddev=0.00000000e+00,prob_range={1.000
00000e+00,1.00000000e+00},prob(percent)="100",prob+-sd="100+-0"]:
8.187957e-01[&length_mean=8.23834034e-01,length_median=8.18795700e-0
1,length_95%HPD={6.39542600e-01,1.01434500e+00}])
[&prob=6.65770410e-01,prob_stddev=3.88734318e-02,prob_range={6.38282
743e-01,6.93258077e-01},prob(percent)="67",prob+-sd="67+-4"]:
1.410829e-01[&length_mean=1.46221569e-01,length_median=1.41082900e-0
1,length_95%HPD={6.72666400e-03,2.89324200e-01}])
[&prob=7.82031910e-01,prob_stddev=1.03484406e-01,prob_range={7.08857
384e-01,8.55206435e-01},prob(percent)="78",prob+-sd="78+-10"]:
1.700327e-01[&length_mean=1.74001633e-01,length_median=1.70032700e-0
1,length_95%HPD={6.17020100e-02,2.91670500e-01}],
(((((30[&prob=1.00000000e+00,prob_stddev=0.00000000e+00,prob_range={
1.00000000e+00,1.00000000e+00},prob(percent)="100",prob+-
sd="100+-0"]:
6.213937e-01[&length_mean=6.27410923e-01,length_median=6.21393700e-0
1,length_95%HPD={4.59019600e-01,7.98514300e-01}],
31[&prob=1.00000000e+00,prob_stddev=0.00000000e+00,prob_range={1.000
00000e+00,1.00000000e+00},prob(percent)="100",prob+-sd="100+-0"]:
7.045778e-01[&length_mean=7.08796921e-01,length_median=7.04577800e-0
1,length_95%HPD={5.11875900e-01,8.80505900e-01}])
[&prob=8.35251767e-01,prob_stddev=7.16503027e-03,prob_range={8.30185
325e-01,8.40318208e-01},prob(percent)="84",prob+-sd="84+-1"]:
1.464204e-01[&length_mean=1.49371554e-01,length_median=1.46420400e-0
1,length_95%HPD={2.84508600e-02,2.66353100e-01}],
(32[&prob=1.00000000e+00,prob_stddev=0.00000000e+00,prob_range={1.00
000000e+00,1.00000000e+00},prob(percent)="100",prob+-sd="100+-0"]:
7.996655e-02[&length_mean=8.16301848e-02,length_median=7.99665500e-0
2,length_95%HPD={3.45472400e-02,1.30507500e-01}],
33[&prob=1.00000000e+00,prob_stddev=0.00000000e+00,prob_range={1.000
00000e+00,1.00000000e+00},prob(percent)="100",prob+-sd="100+-0"]:
6.018167e-02[&length_mean=6.26936153e-02,length_median=6.01816700e-0
2,length_95%HPD={2.38199100e-02,1.07888700e-01}])
[&prob=1.00000000e+00,prob_stddev=0.00000000e+00,prob_range={1.00000
000e+00,1.00000000e+00},prob(percent)="100",prob+-sd="100+-0"]:
5.073845e-01[&length_mean=5.09479964e-01,length_median=5.07384500e-0
1,length_95%HPD={3.74697300e-01,6.52226100e-01}])
[&prob=9.33025199e-01,prob_stddev=1.11246523e-02,prob_range={9.25158

882e-01,9.40891516e-01},prob(percent)="93",prob+-sd="93+-1"]:
1.643117e-01[&length_mean=1.67239779e-01,length_median=1.64311700e-0
1,length_95%HPD={4.48425100e-02,3.04831600e-01}],
(34[&prob=1.00000000e+00,prob_stddev=0.00000000e+00,prob_range={1.00
000000e+00,1.00000000e+00},prob(percent)="100",prob+-sd="100+-0"]:
7.910078e-02[&length_mean=8.10543550e-02,length_median=7.91007800e-0
2,length_95%HPD={3.96773900e-02,1.29680100e-01}],
35[&prob=1.00000000e+00,prob_stddev=0.00000000e+00,prob_range={1.000
00000e+00,1.00000000e+00},prob(percent)="100",prob+-sd="100+-0"]:
3.828434e-02[&length_mean=4.00362002e-02,length_median=3.82843400e-0
2,length_95%HPD={4.14204000e-03,7.74430700e-02}])
[&prob=1.00000000e+00,prob_stddev=0.00000000e+00,prob_range={1.00000
000e+00,1.00000000e+00},prob(percent)="100",prob+-sd="100+-0"]:
7.071083e-01[&length_mean=6.87573158e-01,length_median=7.07108300e-0
1,length_95%HPD={4.25401400e-01,9.10342700e-01}])
[&prob=7.96809031e-01,prob_stddev=1.75291837e-01,prob_range={6.72858
984e-01,9.20759077e-01},prob(percent)="80",prob+-sd="80+-18"]:
1.550828e-01[&length_mean=1.58677053e-01,length_median=1.55082800e-0
1,length_95%HPD={3.54824000e-02,2.75097400e-01}],
(36[&prob=1.00000000e+00,prob_stddev=0.00000000e+00,prob_range={1.00
000000e+00,1.00000000e+00},prob(percent)="100",prob+-sd="100+-0"]:
3.690509e-01[&length_mean=3.69814152e-01,length_median=3.69050900e-0
1,length_95%HPD={2.61323800e-01,4.78808600e-01}],
37[&prob=1.00000000e+00,prob_stddev=0.00000000e+00,prob_range={1.000
00000e+00,1.00000000e+00},prob(percent)="100",prob+-sd="100+-0"]:
1.917485e-01[&length_mean=1.95620752e-01,length_median=1.91748500e-0
1,length_95%HPD={1.05333300e-01,2.92953400e-01}])
[&prob=1.00000000e+00,prob_stddev=0.00000000e+00,prob_range={1.00000
000e+00,1.00000000e+00},prob(percent)="100",prob+-sd="100+-0"]:
4.666799e-01[&length_mean=4.69286438e-01,length_median=4.66679900e-0
1,length_95%HPD={3.45865000e-01,6.10005000e-01}])
[&prob=7.10012888e-01,prob_stddev=1.63224418e-01,prob_range={5.94595
796e-01,8.25429981e-01},prob(percent)="71",prob+-sd="71+-16"]:
1.329289e-01[&length_mean=1.34534470e-01,length_median=1.32928900e-0
1,length_95%HPD={3.57519100e-02,2.35716900e-01}],
((((((41[&prob=1.00000000e+00,prob_stddev=0.00000000e+00,prob_range
={1.00000000e+00,1.00000000e+00},prob(percent)="100",prob+-
sd="100+-0"]:
3.048138e-01[&length_mean=3.07108075e-01,length_median=3.04813800e-0
1,length_95%HPD={2.17648300e-01,4.06971600e-01}],
42[&prob=1.00000000e+00,prob_stddev=0.00000000e+00,prob_range={1.000
00000e+00,1.00000000e+00},prob(percent)="100",prob+-sd="100+-0"]:
3.573962e-01[&length_mean=3.61350216e-01,length_median=3.57396200e-0
1,length_95%HPD={2.63587500e-01,4.62459700e-01}])
[&prob=1.00000000e+00,prob_stddev=0.00000000e+00,prob_range={1.00000
000e+00,1.00000000e+00},prob(percent)="100",prob+-sd="100+-0"]:
1.925055e-01[&length_mean=1.95443802e-01,length_median=1.92505500e-0
1,length_95%HPD={1.06331400e-01,2.91356200e-01}],
47[&prob=1.00000000e+00,prob_stddev=0.00000000e+00,prob_range={1.000
00000e+00,1.00000000e+00},prob(percent)="100",prob+-sd="100+-0"]:
2.098110e-01[&length_mean=2.08671237e-01,length_median=2.09811000e-0
1,length_95%HPD={1.23308500e-01,2.82426400e-01}])
[&prob=1.00000000e+00,prob_stddev=0.00000000e+00,prob_range={1.00000
000e+00,1.00000000e+00},prob(percent)="100",prob+-sd="100+-0"]:

2.499270e-01[&length_mean=2.51092497e-01,length_median=2.49927000e-0
1,length_95%HPD={1.60291600e-01,3.53177700e-01}],
((43[&prob=1.00000000e+00,prob_stddev=0.00000000e+00,prob_range={1.0
0000000e+00,1.00000000e+00},prob(percent)="100",prob+-sd="100+-0"]:
1.583195e-01[&length_mean=1.61032074e-01,length_median=1.58319500e-0
1,length_95%HPD={1.01028100e-01,2.27376800e-01}],
44[&prob=1.00000000e+00,prob_stddev=0.00000000e+00,prob_range={1.000
00000e+00,1.00000000e+00},prob(percent)="100",prob+-sd="100+-0"]:
7.023006e-03[&length_mean=1.02660107e-02,length_median=7.02300600e-0
3,length_95%HPD={2.02686500e-06,3.07417500e-02}])
[&prob=1.00000000e+00,prob_stddev=0.00000000e+00,prob_range={1.00000
000e+00,1.00000000e+00},prob(percent)="100",prob+-sd="100+-0"]:
2.034113e-01[&length_mean=2.05442242e-01,length_median=2.03411300e-0
1,length_95%HPD={1.15821300e-01,2.93290300e-01}],
(45[&prob=1.00000000e+00,prob_stddev=0.00000000e+00,prob_range={1.00
000000e+00,1.00000000e+00},prob(percent)="100",prob+-sd="100+-0"]:
2.182120e-01[&length_mean=2.21595678e-01,length_median=2.18212000e-0
1,length_95%HPD={1.22322300e-01,3.16158800e-01}],
46[&prob=1.00000000e+00,prob_stddev=0.00000000e+00,prob_range={1.000
00000e+00,1.00000000e+00},prob(percent)="100",prob+-sd="100+-0"]:
1.771446e-01[&length_mean=1.79269848e-01,length_median=1.77144600e-0
1,length_95%HPD={9.78105300e-02,2.66631600e-01}])
[&prob=9.99377805e-01,prob_stddev=4.39957999e-04,prob_range={9.99066
708e-01,9.99688903e-01},prob(percent)="100",prob+-sd="100+-0"]:
1.475648e-01[&length_mean=1.51085102e-01,length_median=1.47564800e-0
1,length_95%HPD={4.55913000e-02,2.58158700e-01}])
[&prob=1.00000000e+00,prob_stddev=0.00000000e+00,prob_range={1.00000
000e+00,1.00000000e+00},prob(percent)="100",prob+-sd="100+-0"]:
2.098868e-01[&length_mean=2.12112725e-01,length_median=2.09886800e-0
1,length_95%HPD={1.19145200e-01,3.09203100e-01}])
[&prob=9.99044487e-01,prob_stddev=5.34234713e-04,prob_range={9.98666
726e-01,9.99422248e-01},prob(percent)="100",prob+-sd="100+-0"]:
1.576373e-01[&length_mean=1.60801690e-01,length_median=1.57637300e-0
1,length_95%HPD={5.80061000e-02,2.68109500e-01}],
48[&prob=1.00000000e+00,prob_stddev=0.00000000e+00,prob_range={1.000
00000e+00,1.00000000e+00},prob(percent)="100",prob+-sd="100+-0"]:
5.816771e-01[&length_mean=5.87096276e-01,length_median=5.81677100e-0
1,length_95%HPD={4.48376900e-01,7.53444300e-01}])
[&prob=9.78467624e-01,prob_stddev=5.62517727e-03,prob_range={9.74490
023e-01,9.82445225e-01},prob(percent)="98",prob+-sd="98+-1"]:
1.990109e-01[&length_mean=2.00841911e-01,length_median=1.99010900e-0
1,length_95%HPD={6.63434600e-02,3.31933100e-01}],
((49[&prob=1.00000000e+00,prob_stddev=0.00000000e+00,prob_range={1.0
0000000e+00,1.00000000e+00},prob(percent)="100",prob+-sd="100+-0"]:
1.617403e-01[&length_mean=1.62234948e-01,length_median=1.61740300e-0
1,length_95%HPD={1.04447500e-01,2.13699300e-01}],
(50[&prob=1.00000000e+00,prob_stddev=0.00000000e+00,prob_range={1.00
000000e+00,1.00000000e+00},prob(percent)="100",prob+-sd="100+-0"]:
6.306027e-03[&length_mean=7.40746990e-03,length_median=6.30602700e-0
3,length_95%HPD={1.22328600e-06,1.81295600e-02}],
51[&prob=1.00000000e+00,prob_stddev=0.00000000e+00,prob_range={1.000
00000e+00,1.00000000e+00},prob(percent)="100",prob+-sd="100+-0"]:
1.476168e-01[&length_mean=1.49193095e-01,length_median=1.47616800e-0
1,length_95%HPD={1.04314700e-01,1.99942500e-01}])

[&prob=1.00000000e+00,prob_stddev=0.00000000e+00,prob_range={1.00000
000e+00,1.00000000e+00},prob(percent)="100",prob+-sd="100+-0"]:
1.315661e-01[&length_mean=1.32941625e-01,length_median=1.31566100e-0
1,length_95%HPD={8.31070200e-02,1.86550000e-01}])
[&prob=1.00000000e+00,prob_stddev=0.00000000e+00,prob_range={1.00000
000e+00,1.00000000e+00},prob(percent)="100",prob+-sd="100+-0"]:
2.975051e-01[&length_mean=2.98925195e-01,length_median=2.97505100e-0
1,length_95%HPD={1.98300400e-01,4.06294600e-01}],
52[&prob=1.00000000e+00,prob_stddev=0.00000000e+00,prob_range={1.000
00000e+00,1.00000000e+00},prob(percent)="100",prob+-sd="100+-0"]:
2.575901e-01[&length_mean=2.59302196e-01,length_median=2.57590100e-0
1,length_95%HPD={1.57725900e-01,3.57064000e-01}])
[&prob=1.00000000e+00,prob_stddev=0.00000000e+00,prob_range={1.00000
000e+00,1.00000000e+00},prob(percent)="100",prob+-sd="100+-0"]:
6.451398e-01[&length_mean=6.46737895e-01,length_median=6.45139800e-0
1,length_95%HPD={4.86026300e-01,8.12350700e-01}])
[&prob=9.38802720e-01,prob_stddev=3.28711476e-02,prob_range={9.15559
308e-01,9.62046131e-01},prob(percent)="94",prob+-sd="94+-3"]:
2.185964e-01[&length_mean=2.21502845e-01,length_median=2.18596400e-0
1,length_95%HPD={7.98407000e-02,3.56852900e-01}],
(92[&prob=1.00000000e+00,prob_stddev=0.00000000e+00,prob_range={1.00
000000e+00,1.00000000e+00},prob(percent)="100",prob+-sd="100+-0"]:
5.327433e-01[&length_mean=5.36117322e-01,length_median=5.32743300e-0
1,length_95%HPD={3.22740400e-01,7.37366300e-01}],
93[&prob=1.00000000e+00,prob_stddev=0.00000000e+00,prob_range={1.000
00000e+00,1.00000000e+00},prob(percent)="100",prob+-sd="100+-0"]:
5.759849e-01[&length_mean=5.81585685e-01,length_median=5.75984900e-0
1,length_95%HPD={3.96231600e-01,7.70738100e-01}])
[&prob=9.57224123e-01,prob_stddev=1.51156998e-02,prob_range={9.46535
710e-01,9.67912537e-01},prob(percent)="96",prob+-sd="96+-2"]:
2.329821e-01[&length_mean=2.34458227e-01,length_median=2.32982100e-0
1,length_95%HPD={7.32137900e-02,3.89835200e-01}])
[&prob=8.19519132e-01,prob_stddev=6.36682076e-02,prob_range={7.74498
911e-01,8.64539354e-01},prob(percent)="82",prob+-sd="82+-6"]:
1.940770e-01[&length_mean=1.91448264e-01,length_median=1.94077000e-0
1,length_95%HPD={3.63809300e-02,3.38418900e-01}],
(53[&prob=1.00000000e+00,prob_stddev=0.00000000e+00,prob_range={1.00
000000e+00,1.00000000e+00},prob(percent)="100",prob+-sd="100+-0"]:
3.526209e-01[&length_mean=3.52458380e-01,length_median=3.52620900e-0
1,length_95%HPD={2.21774000e-01,4.90557200e-01}],
54[&prob=1.00000000e+00,prob_stddev=0.00000000e+00,prob_range={1.000
00000e+00,1.00000000e+00},prob(percent)="100",prob+-sd="100+-0"]:
5.976572e-01[&length_mean=6.00123485e-01,length_median=5.97657200e-0
1,length_95%HPD={4.38652600e-01,7.53419600e-01}])
[&prob=9.99822230e-01,prob_stddev=2.51404571e-04,prob_range={9.99644
460e-01,1.00000000e+00},prob(percent)="100",prob+-sd="100+-0"]:
5.015698e-01[&length_mean=5.00243874e-01,length_median=5.01569800e-0
1,length_95%HPD={3.27104400e-01,6.64256400e-01}],
((((55[&prob=1.00000000e+00,prob_stddev=0.00000000e+00,prob_range={1
.00000000e+00,1.00000000e+00},prob(percent)="100",prob+-
sd="100+-0"]:
1.192585e-01[&length_mean=1.22728550e-01,length_median=1.19258500e-0
1,length_95%HPD={5.07924600e-02,2.06329400e-01}],
56[&prob=1.00000000e+00,prob_stddev=0.00000000e+00,prob_range={1.000

00000e+00,1.00000000e+00},prob(percent)="100",prob+-sd="100+-0"]:
1.359448e-01[&length_mean=1.37409467e-01,length_median=1.35944800e-0
1,length_95%HPD={6.87829000e-02,2.06685000e-01}])
[&prob=1.00000000e+00,prob_stddev=0.00000000e+00,prob_range={1.00000
000e+00,1.00000000e+00},prob(percent)="100",prob+-sd="100+-0"]:
3.340757e-01[&length_mean=3.35953486e-01,length_median=3.34075700e-0
1,length_95%HPD={2.24393300e-01,4.58089500e-01}],
57[&prob=1.00000000e+00,prob_stddev=0.00000000e+00,prob_range={1.000
00000e+00,1.00000000e+00},prob(percent)="100",prob+-sd="100+-0"]:
4.330758e-01[&length_mean=4.34140859e-01,length_median=4.33075800e-0
1,length_95%HPD={3.19835300e-01,5.53173500e-01}])
[&prob=1.00000000e+00,prob_stddev=0.00000000e+00,prob_range={1.00000
000e+00,1.00000000e+00},prob(percent)="100",prob+-sd="100+-0"]:
3.189148e-01[&length_mean=3.20242807e-01,length_median=3.18914800e-0
1,length_95%HPD={1.89161600e-01,4.42096100e-01}],
(58[&prob=1.00000000e+00,prob_stddev=0.00000000e+00,prob_range={1.00
000000e+00,1.00000000e+00},prob(percent)="100",prob+-sd="100+-0"]:
3.731467e-01[&length_mean=3.74481110e-01,length_median=3.73146700e-0
1,length_95%HPD={2.37823800e-01,4.98546800e-01}],
77[&prob=1.00000000e+00,prob_stddev=0.00000000e+00,prob_range={1.000
00000e+00,1.00000000e+00},prob(percent)="100",prob+-sd="100+-0"]:
6.938905e-01[&length_mean=7.01643628e-01,length_median=6.93890500e-0
1,length_95%HPD={5.33508900e-01,8.88154400e-01}])
[&prob=9.27025466e-01,prob_stddev=2.47004991e-02,prob_range={9.09559
575e-01,9.44491356e-01},prob(percent)="93",prob+-sd="93+-2"]:
1.605787e-01[&length_mean=1.63709466e-01,length_median=1.60578700e-0
1,length_95%HPD={4.96957300e-02,2.94542400e-01}],
66[&prob=1.00000000e+00,prob_stddev=0.00000000e+00,prob_range={1.000
00000e+00,1.00000000e+00},prob(percent)="100",prob+-sd="100+-0"]:
5.875766e-01[&length_mean=5.83569939e-01,length_median=5.87576600e-0
1,length_95%HPD={3.95846500e-01,7.69795200e-01}],
(68[&prob=1.00000000e+00,prob_stddev=0.00000000e+00,prob_range={1.00
000000e+00,1.00000000e+00},prob(percent)="100",prob+-sd="100+-0"]:
5.861034e-01[&length_mean=5.90374583e-01,length_median=5.86103400e-0
1,length_95%HPD={4.44881000e-01,7.39878800e-01}],
75[&prob=1.00000000e+00,prob_stddev=0.00000000e+00,prob_range={1.000
00000e+00,1.00000000e+00},prob(percent)="100",prob+-sd="100+-0"]:
6.211296e-01[&length_mean=6.26642386e-01,length_median=6.21129600e-0
1,length_95%HPD={4.80602000e-01,7.90406700e-01}])
[&prob=9.98533399e-01,prob_stddev=3.77106856e-04,prob_range={9.98266
744e-01,9.98800053e-01},prob(percent)="100",prob+-sd="100+-0"]:
2.808273e-01[&length_mean=2.79066696e-01,length_median=2.80827300e-0
1,length_95%HPD={1.15194300e-01,4.25664600e-01}],
76[&prob=1.00000000e+00,prob_stddev=0.00000000e+00,prob_range={1.000
00000e+00,1.00000000e+00},prob(percent)="100",prob+-sd="100+-0"]:
6.216684e-01[&length_mean=5.33923272e-01,length_median=6.21668400e-0
1,length_95%HPD={3.68748500e-04,8.59950600e-01}],
79[&prob=1.00000000e+00,prob_stddev=0.00000000e+00,prob_range={1.000
00000e+00,1.00000000e+00},prob(percent)="100",prob+-sd="100+-0"]:
6.283668e-01[&length_mean=5.66562081e-01,length_median=6.28366800e-0
1,length_95%HPD={3.39303000e-03,9.82284800e-01}])
[&prob=5.83418515e-01,prob_stddev=1.23502495e-02,prob_range={5.74685
570e-01,5.92151460e-01},prob(percent)="58",prob+-sd="58+-1"]:
7.909957e-02[&length_mean=8.20550222e-02,length_median=7.90995700e-0

2,length_95%HPD={9.70483400e-03,1.57295600e-01}],
(((((59[&prob=1.00000000e+00,prob_stddev=0.00000000e+00,prob_range={
1.00000000e+00,1.00000000e+00},prob(percent)="100",prob+-
sd="100+-0"]:
1.565455e-01[&length_mean=1.57649424e-01,length_median=1.56545500e-0
1,length_95%HPD={9.11844800e-02,2.25187700e-01}],
60[&prob=1.00000000e+00,prob_stddev=0.00000000e+00,prob_range={1.000
00000e+00,1.00000000e+00},prob(percent)="100",prob+-sd="100+-0"]:
1.652637e-01[&length_mean=1.68214000e-01,length_median=1.65263700e-0
1,length_95%HPD={1.06663200e-01,2.39356200e-01}])
[&prob=1.00000000e+00,prob_stddev=0.00000000e+00,prob_range={1.00000
000e+00,1.00000000e+00},prob(percent)="100",prob+-sd="100+-0"]:
1.744641e-01[&length_mean=1.77274279e-01,length_median=1.74464100e-0
1,length_95%HPD={9.69868200e-02,2.63605200e-01}],
(61[&prob=1.00000000e+00,prob_stddev=0.00000000e+00,prob_range={1.00
000000e+00,1.00000000e+00},prob(percent)="100",prob+-sd="100+-0"]:
1.298310e-02[&length_mean=1.62177192e-02,length_median=1.29831000e-0
2,length_95%HPD={5.23224700e-06,4.28057400e-02}],
62[&prob=1.00000000e+00,prob_stddev=0.00000000e+00,prob_range={1.000
00000e+00,1.00000000e+00},prob(percent)="100",prob+-sd="100+-0"]:
4.669331e-02[&length_mean=5.00520325e-02,length_median=4.66933100e-0
2,length_95%HPD={1.19874300e-02,9.25073900e-02}])
[&prob=1.00000000e+00,prob_stddev=0.00000000e+00,prob_range={1.00000
000e+00,1.00000000e+00},prob(percent)="100",prob+-sd="100+-0"]:
1.956723e-01[&length_mean=1.99314777e-01,length_median=1.95672300e-0
1,length_95%HPD={1.11110700e-01,2.80057400e-01}])
[&prob=9.81511933e-01,prob_stddev=9.67907598e-03,prob_range={9.74667
793e-01,9.88356073e-01},prob(percent)="98",prob+-sd="98+-1"]:
9.270366e-02[&length_mean=9.58182998e-02,length_median=9.27036600e-0
2,length_95%HPD={2.22446500e-02,1.67145100e-01}],
63[&prob=1.00000000e+00,prob_stddev=0.00000000e+00,prob_range={1.000
00000e+00,1.00000000e+00},prob(percent)="100",prob+-sd="100+-0"]:
4.517784e-01[&length_mean=4.52510485e-01,length_median=4.51778400e-0
1,length_95%HPD={3.52488100e-01,5.52263500e-01}])
[&prob=1.00000000e+00,prob_stddev=0.00000000e+00,prob_range={1.00000
000e+00,1.00000000e+00},prob(percent)="100",prob+-sd="100+-0"]:
2.359846e-01[&length_mean=2.36122586e-01,length_median=2.35984600e-0
1,length_95%HPD={1.33018700e-01,3.33907300e-01}],
((64[&prob=1.00000000e+00,prob_stddev=0.00000000e+00,prob_range={1.0
0000000e+00,1.00000000e+00},prob(percent)="100",prob+-sd="100+-0"]:
4.115557e-01[&length_mean=4.14701901e-01,length_median=4.11555700e-0
1,length_95%HPD={2.87207200e-01,5.53069500e-01}],
65[&prob=1.00000000e+00,prob_stddev=0.00000000e+00,prob_range={1.000
00000e+00,1.00000000e+00},prob(percent)="100",prob+-sd="100+-0"]:
3.569407e-01[&length_mean=3.59763629e-01,length_median=3.56940700e-0
1,length_95%HPD={2.20795700e-01,5.01336200e-01}])
[&prob=7.59210702e-01,prob_stddev=2.51404571e-03,prob_range={7.57433
003e-01,7.60988401e-01},prob(percent)="76",prob+-sd="76+-0"]:
1.218622e-01[&length_mean=1.26601140e-01,length_median=1.21862200e-0
1,length_95%HPD={2.25976900e-02,2.47635500e-01}],
67[&prob=1.00000000e+00,prob_stddev=0.00000000e+00,prob_range={1.000
00000e+00,1.00000000e+00},prob(percent)="100",prob+-sd="100+-0"]:
5.527175e-01[&length_mean=5.60038515e-01,length_median=5.52717500e-0
1,length_95%HPD={4.14675000e-01,7.23220000e-01}])

[&prob=9.97289009e-01,prob_stddev=3.45681285e-03,prob_range={9.94844
674e-01,9.99733345e-01},prob(percent)="100",prob+-sd="100+-0"]:
2.949335e-01[&length_mean=2.95443884e-01,length_median=2.94933500e-0
1,length_95%HPD={1.55216900e-01,4.27645200e-01}])
[&prob=9.31158615e-01,prob_stddev=2.40091365e-02,prob_range={9.14181
592e-01,9.48135638e-01},prob(percent)="93",prob+-sd="93+-2"]:
1.109040e-01[&length_mean=1.13583900e-01,length_median=1.10904000e-0
1,length_95%HPD={2.77121900e-02,1.97081800e-01}],
(69[&prob=1.00000000e+00,prob_stddev=0.00000000e+00,prob_range={1.00
000000e+00,1.00000000e+00},prob(percent)="100",prob+-sd="100+-0"]:
4.965194e-01[&length_mean=4.98703210e-01,length_median=4.96519400e-0
1,length_95%HPD={3.60783000e-01,6.30661200e-01}],
70[&prob=1.00000000e+00,prob_stddev=0.00000000e+00,prob_range={1.000
00000e+00,1.00000000e+00},prob(percent)="100",prob+-sd="100+-0"]:
6.606284e-01[&length_mean=6.61797707e-01,length_median=6.60628400e-0
1,length_95%HPD={5.20779000e-01,8.17733400e-01}])
[&prob=1.00000000e+00,prob_stddev=0.00000000e+00,prob_range={1.00000
000e+00,1.00000000e+00},prob(percent)="100",prob+-sd="100+-0"]:
2.544018e-01[&length_mean=2.57059725e-01,length_median=2.54401800e-0
1,length_95%HPD={1.32636100e-01,3.75607100e-01}])
[&prob=9.66690369e-01,prob_stddev=2.86286955e-02,prob_range={9.46446
825e-01,9.86933914e-01},prob(percent)="97",prob+-sd="97+-3"]:
1.438109e-01[&length_mean=1.45646316e-01,length_median=1.43810900e-0
1,length_95%HPD={6.33585400e-02,2.41846700e-01}])
[&prob=8.31651927e-01,prob_stddev=4.02247313e-03,prob_range={8.28807
609e-01,8.34496245e-01},prob(percent)="83",prob+-sd="83+-0"]:
1.177081e-01[&length_mean=1.20214244e-01,length_median=1.17708100e-0
1,length_95%HPD={3.86403700e-02,2.04586600e-01}],
(((71[&prob=1.00000000e+00,prob_stddev=0.00000000e+00,prob_range={1.
00000000e+00,1.00000000e+00},prob(percent)="100",prob+-sd="100+-0"]:
4.466662e-01[&length_mean=4.49451188e-01,length_median=4.46666200e-0
1,length_95%HPD={3.25408500e-01,5.87297600e-01}],
72[&prob=1.00000000e+00,prob_stddev=0.00000000e+00,prob_range={1.000
00000e+00,1.00000000e+00},prob(percent)="100",prob+-sd="100+-0"]:
3.527512e-01[&length_mean=3.55151967e-01,length_median=3.52751200e-0
1,length_95%HPD={2.49305300e-01,4.84056300e-01}])
[&prob=6.93858051e-01,prob_stddev=9.71050155e-03,prob_range={6.86991
689e-01,7.00724412e-01},prob(percent)="69",prob+-sd="69+-1"]:
9.833443e-02[&length_mean=1.08021933e-01,length_median=9.83344300e-0
2,length_95%HPD={8.38611100e-05,2.32204500e-01}],
73[&prob=1.00000000e+00,prob_stddev=0.00000000e+00,prob_range={1.000
00000e+00,1.00000000e+00},prob(percent)="100",prob+-sd="100+-0"]:
5.903296e-01[&length_mean=5.99261729e-01,length_median=5.90329600e-0
1,length_95%HPD={4.09860500e-01,8.17940600e-01}])
[&prob=9.99400027e-01,prob_stddev=8.48490427e-04,prob_range={9.98800
053e-01,1.00000000e+00},prob(percent)="100",prob+-sd="100+-0"]:
4.015023e-01[&length_mean=4.01124359e-01,length_median=4.01502300e-0
1,length_95%HPD={2.12721900e-01,5.75130300e-01}],
78[&prob=1.00000000e+00,prob_stddev=0.00000000e+00,prob_range={1.000
00000e+00,1.00000000e+00},prob(percent)="100",prob+-sd="100+-0"]:
7.369143e-01[&length_mean=7.37263201e-01,length_median=7.36914300e-0
1,length_95%HPD={5.54109800e-01,9.29787200e-01}])
[&prob=8.65183770e-01,prob_stddev=5.26378320e-02,prob_range={8.27963
202e-01,9.02404338e-01},prob(percent)="87",prob+-sd="87+-5"]:

1.495162e-01[&length_mean=1.50842602e-01,length_median=1.49516200e-01,length_95%HPD={2.87085700e-02,2.65358800e-01}],
((74[&prob=1.00000000e+00,prob_stddev=0.00000000e+00,prob_range={1.00000000e+00,1.00000000e+00},prob(percent)="100",prob+-sd="100+-0"]:
5.981440e-01[&length_mean=6.03397329e-01,length_median=5.98144000e-01,length_95%HPD={2.71486200e-01,9.34023000e-01}],
(((((((95[&prob=1.00000000e+00,prob_stddev=0.00000000e+00,prob_range={1.00000000e+00,1.00000000e+00},prob(percent)="100",prob+-sd="100+-0"]:
5.184459e-01[&length_mean=5.19728201e-01,length_median=5.18445900e-01,length_95%HPD={4.16866600e-01,6.24076300e-01}],
98[&prob=1.00000000e+00,prob_stddev=0.00000000e+00,prob_range={1.00000000e+00,1.00000000e+00},prob(percent)="100",prob+-sd="100+-0"]:
2.599443e-01[&length_mean=2.61246571e-01,length_median=2.59944300e-01,length_95%HPD={1.78755700e-01,3.49979800e-01}])
[&prob=1.00000000e+00,prob_stddev=0.00000000e+00,prob_range={1.00000000e+00,1.00000000e+00},prob(percent)="100",prob+-sd="100+-0"]:
1.683449e-01[&length_mean=1.71283045e-01,length_median=1.68344900e-01,length_95%HPD={9.22509200e-02,2.54166800e-01}],
(96[&prob=1.00000000e+00,prob_stddev=0.00000000e+00,prob_range={1.00000000e+00,1.00000000e+00},prob(percent)="100",prob+-sd="100+-0"]:
3.201468e-01[&length_mean=3.20777424e-01,length_median=3.20146800e-01,length_95%HPD={2.21429200e-01,4.18459800e-01}],
97[&prob=1.00000000e+00,prob_stddev=0.00000000e+00,prob_range={1.00000000e+00,1.00000000e+00},prob(percent)="100",prob+-sd="100+-0"]:
3.238213e-01[&length_mean=3.25860604e-01,length_median=3.23821300e-01,length_95%HPD={2.34232800e-01,4.31684000e-01}])
[&prob=9.85845074e-01,prob_stddev=1.03704385e-03,prob_range={9.85111773e-01,9.86578374e-01},prob(percent)="99",prob+-sd="99+-0"]:
1.259995e-01[&length_mean=1.28738834e-01,length_median=1.25999500e-01,length_95%HPD={5.19016300e-02,2.14620400e-01}])
[&prob=9.68356962e-01,prob_stddev=7.35358370e-03,prob_range={9.63157193e-01,9.73556731e-01},prob(percent)="97",prob+-sd="97+-1"]:
1.269681e-01[&length_mean=1.28521793e-01,length_median=1.26968100e-01,length_95%HPD={4.13771200e-02,2.14067300e-01}],
(((99[&prob=1.00000000e+00,prob_stddev=0.00000000e+00,prob_range={1.00000000e+00,1.00000000e+00},prob(percent)="100",prob+-sd="100+-0"]:
2.168029e-01[&length_mean=2.19301400e-01,length_median=2.16802900e-01,length_95%HPD={9.58011200e-02,3.52221600e-01}],
101[&prob=1.00000000e+00,prob_stddev=0.00000000e+00,prob_range={1.00000000e+00,1.00000000e+00},prob(percent)="100",prob+-sd="100+-0"]:
6.217349e-01[&length_mean=6.27256380e-01,length_median=6.21734900e-01,length_95%HPD={4.45158000e-01,8.48249400e-01}])
[&prob=9.88489400e-01,prob_stddev=1.06846943e-03,prob_range={9.87733878e-01,9.89244922e-01},prob(percent)="99",prob+-sd="99+-0"]:
1.773950e-01[&length_mean=1.80524101e-01,length_median=1.77395000e-01,length_95%HPD={5.87878700e-02,3.12666000e-01}],
(100[&prob=1.00000000e+00,prob_stddev=0.00000000e+00,prob_range={1.00000000e+00,1.00000000e+00},prob(percent)="100",prob+-sd="100+-0"]:
2.372133e-01[&length_mean=2.39218541e-01,length_median=2.37213300e-01,length_95%HPD={1.31531800e-01,3.54745200e-01}],
107[&prob=1.00000000e+00,prob_stddev=0.00000000e+00,prob_range={1.00000000e+00,1.00000000e+00},prob(percent)="100",prob+-sd="100+-0"]:
5.293450e-01[&length_mean=5.29640397e-01,length_median=5.29345000e-0

1,length_95%HPD={3.66296800e-01,6.90419600e-01}])
[&prob=9.56935247e-01,prob_stddev=1.04332897e-02,prob_range={9.49557
797e-01,9.64312697e-01},prob(percent)="96",prob+-sd="96+-1"]:
1.328175e-01[&length_mean=1.36933734e-01,length_median=1.32817500e-0
1,length_95%HPD={4.03314600e-02,2.44117800e-01}])
[&prob=9.99866673e-01,prob_stddev=1.88553428e-04,prob_range={9.99733
345e-01,1.00000000e+00},prob(percent)="100",prob+-sd="100+-0"]:
1.966756e-01[&length_mean=1.97911607e-01,length_median=1.96675600e-0
1,length_95%HPD={9.74248000e-02,2.91801000e-01}],
103[&prob=1.00000000e+00,prob_stddev=0.00000000e+00,prob_range={1.00
000000e+00,1.00000000e+00},prob(percent)="100",prob+-sd="100+-0"]:
5.529869e-01[&length_mean=5.53563606e-01,length_median=5.52986900e-0
1,length_95%HPD={4.22532100e-01,6.76597100e-01}])
[&prob=9.97089018e-01,prob_stddev=4.11674985e-03,prob_range={9.94178
037e-01,1.00000000e+00},prob(percent)="100",prob+-sd="100+-0"]:
1.795799e-01[&length_mean=1.81504343e-01,length_median=1.79579900e-0
1,length_95%HPD={8.57741200e-02,2.75973400e-01}])
[&prob=9.95800187e-01,prob_stddev=1.16274614e-03,prob_range={9.94978
001e-01,9.96622372e-01},prob(percent)="100",prob+-sd="100+-0"]:
1.490722e-01[&length_mean=1.51143693e-01,length_median=1.49072200e-0
1,length_95%HPD={6.22032800e-02,2.46685000e-01}],
((104[&prob=1.00000000e+00,prob_stddev=0.00000000e+00,prob_range={1.
00000000e+00,1.00000000e+00},prob(percent)="100",prob+-sd="100+-0"]:
3.050439e-01[&length_mean=3.05233256e-01,length_median=3.05043900e-0
1,length_95%HPD={2.01280600e-01,4.00632400e-01}],
105[&prob=1.00000000e+00,prob_stddev=0.00000000e+00,prob_range={1.00
000000e+00,1.00000000e+00},prob(percent)="100",prob+-sd="100+-0"]:
3.889269e-01[&length_mean=3.89069680e-01,length_median=3.88926900e-0
1,length_95%HPD={2.85406500e-01,4.86035100e-01}])
[&prob=1.00000000e+00,prob_stddev=0.00000000e+00,prob_range={1.00000
000e+00,1.00000000e+00},prob(percent)="100",prob+-sd="100+-0"]:
2.454988e-01[&length_mean=2.47087541e-01,length_median=2.45498800e-0
1,length_95%HPD={1.52019200e-01,3.55197900e-01}],
(106[&prob=1.00000000e+00,prob_stddev=0.00000000e+00,prob_range={1.0
0000000e+00,1.00000000e+00},prob(percent)="100",prob+-sd="100+-0"]:
4.253821e-01[&length_mean=4.27677441e-01,length_median=4.25382100e-0
1,length_95%HPD={3.20943000e-01,5.40542600e-01}],
111[&prob=1.00000000e+00,prob_stddev=0.00000000e+00,prob_range={1.00
000000e+00,1.00000000e+00},prob(percent)="100",prob+-sd="100+-0"]:
5.592507e-01[&length_mean=5.51051308e-01,length_median=5.59250700e-0
1,length_95%HPD={3.86120900e-01,7.94690900e-01}])
[&prob=6.83258522e-01,prob_stddev=4.72640593e-02,prob_range={6.49837
785e-01,7.16679259e-01},prob(percent)="68",prob+-sd="68+-5"]:
8.862755e-02[&length_mean=9.10953229e-02,length_median=8.86275500e-0
2,length_95%HPD={1.48689000e-02,1.72200100e-01}])
[&prob=9.50957735e-01,prob_stddev=6.93562360e-02,prob_range={9.01915
470e-01,1.00000000e+00},prob(percent)="95",prob+-sd="95+-7"]:
2.811854e-01[&length_mean=2.83677346e-01,length_median=2.81185400e-0
1,length_95%HPD={1.74288600e-01,3.92986900e-01}])
[&prob=9.45846851e-01,prob_stddev=7.12417703e-02,prob_range={8.95471
312e-01,9.96222390e-01},prob(percent)="95",prob+-sd="95+-7"]:
2.596231e-01[&length_mean=2.59238631e-01,length_median=2.59623100e-0
1,length_95%HPD={1.47853500e-01,3.83518700e-01}],
(102[&prob=1.00000000e+00,prob_stddev=0.00000000e+00,prob_range={1.0

0000000e+00,1.00000000e+00},prob(percent)="100",prob+-sd="100+-0"]:
5.058733e-01[&length_mean=5.15096741e-01,length_median=5.05873300e-0
1,length_95%HPD={3.28559800e-01,7.37490000e-01}],
((108[&prob=1.00000000e+00,prob_stddev=0.00000000e+00,prob_range={1.
00000000e+00,1.00000000e+00},prob(percent)="100",prob+-sd="100+-0"]:
1.698088e-01[&length_mean=1.73145821e-01,length_median=1.69808800e-0
1,length_95%HPD={8.86122900e-02,2.73219500e-01}],
(109[&prob=1.00000000e+00,prob_stddev=0.00000000e+00,prob_range={1.0
0000000e+00,1.00000000e+00},prob(percent)="100",prob+-sd="100+-0"]:
3.643632e-02[&length_mean=3.86472454e-02,length_median=3.64363200e-0
2,length_95%HPD={2.63972200e-04,7.43254900e-02}],
110[&prob=1.00000000e+00,prob_stddev=0.00000000e+00,prob_range={1.00
000000e+00,1.00000000e+00},prob(percent)="100",prob+-sd="100+-0"]:
8.890716e-02[&length_mean=8.96846726e-02,length_median=8.89071600e-0
2,length_95%HPD={4.93581300e-02,1.42471200e-01}])
[&prob=9.97644549e-01,prob_stddev=1.44557628e-03,prob_range={9.96622
372e-01,9.98666726e-01},prob(percent)="100",prob+-sd="100+-0"]:
1.289865e-01[&length_mean=1.32237359e-01,length_median=1.28986500e-0
1,length_95%HPD={4.24392100e-02,2.18009400e-01}])
[&prob=1.00000000e+00,prob_stddev=0.00000000e+00,prob_range={1.00000
000e+00,1.00000000e+00},prob(percent)="100",prob+-sd="100+-0"]:
5.262030e-01[&length_mean=5.31296247e-01,length_median=5.26203000e-0
1,length_95%HPD={3.66189300e-01,6.87422600e-01}],
(112[&prob=1.00000000e+00,prob_stddev=0.00000000e+00,prob_range={1.0
0000000e+00,1.00000000e+00},prob(percent)="100",prob+-sd="100+-0"]:
7.016275e-01[&length_mean=7.05327588e-01,length_median=7.01627500e-0
1,length_95%HPD={4.68138200e-01,9.59712300e-01}],
(115[&prob=1.00000000e+00,prob_stddev=0.00000000e+00,prob_range={1.0
0000000e+00,1.00000000e+00},prob(percent)="100",prob+-sd="100+-0"]:
1.594156e-01[&length_mean=1.62939486e-01,length_median=1.59415600e-0
1,length_95%HPD={8.16538500e-02,2.51674100e-01}],
116[&prob=1.00000000e+00,prob_stddev=0.00000000e+00,prob_range={1.00
000000e+00,1.00000000e+00},prob(percent)="100",prob+-sd="100+-0"]:
3.206327e-01[&length_mean=3.23154914e-01,length_median=3.20632700e-0
1,length_95%HPD={2.33126700e-01,4.27820400e-01}])
[&prob=1.00000000e+00,prob_stddev=0.00000000e+00,prob_range={1.00000
000e+00,1.00000000e+00},prob(percent)="100",prob+-sd="100+-0"]:
3.509815e-01[&length_mean=3.57183517e-01,length_median=3.50981500e-0
1,length_95%HPD={1.60531200e-01,5.64866200e-01}])
[&prob=9.94578019e-01,prob_stddev=9.42767141e-04,prob_range={9.93911
382e-01,9.95244656e-01},prob(percent)="99",prob+-sd="99+-0"]:
2.559388e-01[&length_mean=2.58923082e-01,length_median=2.55938800e-0
1,length_95%HPD={8.89206500e-02,4.42185200e-01}])
[&prob=7.90764855e-01,prob_stddev=1.28844843e-02,prob_range={7.81654
149e-01,7.99875561e-01},prob(percent)="79",prob+-sd="79+-1"]:
1.316235e-01[&length_mean=1.36813387e-01,length_median=1.31623500e-0
1,length_95%HPD={4.95169700e-04,2.61089400e-01}])
[&prob=8.05875294e-01,prob_stddev=2.09922817e-02,prob_range={7.91031
510e-01,8.20719079e-01},prob(percent)="81",prob+-sd="81+-2"]:
1.198578e-01[&length_mean=1.24385280e-01,length_median=1.19857800e-0
1,length_95%HPD={2.09680300e-02,2.41241600e-01}])
[&prob=9.41024843e-01,prob_stddev=6.69993181e-02,prob_range={8.93649
171e-01,9.88400516e-01},prob(percent)="94",prob+-sd="94+-7"]:
1.687506e-01[&length_mean=1.71192973e-01,length_median=1.68750600e-0

1,length_95%HPD={6.97806600e-02,2.83434000e-01}],
((113[&prob=1.00000000e+00,prob_stddev=0.00000000e+00,prob_range={1.
00000000e+00,1.00000000e+00},prob(percent)="100",prob+-sd="100+-0"]:
1.554930e-01[&length_mean=1.59653159e-01,length_median=1.55493000e-0
1,length_95%HPD={3.87075800e-02,2.86107800e-01}],
114[&prob=1.00000000e+00,prob_stddev=0.00000000e+00,prob_range={1.00
000000e+00,1.00000000e+00},prob(percent)="100",prob+-sd="100+-0"]:
1.962084e-01[&length_mean=2.01541444e-01,length_median=1.96208400e-0
1,length_95%HPD={5.24095900e-02,3.55129800e-01}])
[&prob=9.99755566e-01,prob_stddev=3.45681285e-04,prob_range={9.99511
133e-01,1.00000000e+00},prob(percent)="100",prob+-sd="100+-0"]:
4.365899e-01[&length_mean=4.39630481e-01,length_median=4.36589900e-0
1,length_95%HPD={2.37720200e-01,6.50650800e-01}],
117[&prob=1.00000000e+00,prob_stddev=0.00000000e+00,prob_range={1.00
000000e+00,1.00000000e+00},prob(percent)="100",prob+-sd="100+-0"]:
6.617263e-01[&length_mean=6.65075326e-01,length_median=6.61726300e-0
1,length_95%HPD={4.85791800e-01,8.69064400e-01}])
[&prob=8.31896360e-01,prob_stddev=1.00876084e-02,prob_range={8.24763
344e-01,8.39029376e-01},prob(percent)="83",prob+-sd="83+-1"]:
1.954251e-01[&length_mean=1.96337738e-01,length_median=1.95425100e-0
1,length_95%HPD={3.86575000e-02,3.37396500e-01}])
[&prob=8.65517088e-01,prob_stddev=7.52956690e-02,prob_range={8.12275
010e-01,9.18759166e-01},prob(percent)="87",prob+-sd="87+-8"]:
1.803477e-01[&length_mean=1.83701581e-01,length_median=1.80347700e-0
1,length_95%HPD={6.37337400e-02,3.16974000e-01}],
(((120[&prob=1.00000000e+00,prob_stddev=0.00000000e+00,prob_range={1
.00000000e+00,1.00000000e+00},prob(percent)="100",prob+-
sd="100+-0"]:
3.939902e-01[&length_mean=4.02960822e-01,length_median=3.93990200e-0
1,length_95%HPD={1.78359500e-01,6.50239800e-01}],
121[&prob=1.00000000e+00,prob_stddev=0.00000000e+00,prob_range={1.00
000000e+00,1.00000000e+00},prob(percent)="100",prob+-sd="100+-0"]:
5.704619e-01[&length_mean=5.77067472e-01,length_median=5.70461900e-0
1,length_95%HPD={3.46601200e-01,7.94294600e-01}])
[&prob=7.12123906e-01,prob_stddev=3.29968499e-03,prob_range={7.09790
676e-01,7.14457135e-01},prob(percent)="71",prob+-sd="71+-0"]:
1.395518e-01[&length_mean=1.48953265e-01,length_median=1.39551800e-0
1,length_95%HPD={5.82678900e-03,2.96027500e-01}],
(122[&prob=1.00000000e+00,prob_stddev=0.00000000e+00,prob_range={1.0
0000000e+00,1.00000000e+00},prob(percent)="100",prob+-sd="100+-0"]:
5.084761e-01[&length_mean=5.12393517e-01,length_median=5.08476100e-0
1,length_95%HPD={3.64979500e-01,6.90474800e-01}],
(123[&prob=1.00000000e+00,prob_stddev=0.00000000e+00,prob_range={1.0
0000000e+00,1.00000000e+00},prob(percent)="100",prob+-sd="100+-0"]:
2.386505e-01[&length_mean=2.38927321e-01,length_median=2.38650500e-0
1,length_95%HPD={1.40372700e-01,3.40283400e-01}],
124[&prob=1.00000000e+00,prob_stddev=0.00000000e+00,prob_range={1.00
000000e+00,1.00000000e+00},prob(percent)="100",prob+-sd="100+-0"]:
3.225843e-01[&length_mean=3.24524346e-01,length_median=3.22584300e-0
1,length_95%HPD={2.13803700e-01,4.44981500e-01}])
[&prob=9.83556286e-01,prob_stddev=1.63412971e-03,prob_range={9.82400
782e-01,9.84711791e-01},prob(percent)="98",prob+-sd="98+-0"]:
1.683431e-01[&length_mean=1.72335973e-01,length_median=1.68343100e-0
1,length_95%HPD={2.82290100e-02,3.04500700e-01}])

[&prob=8.37807209e-01,prob_stddev=7.76211613e-03,prob_range={8.32318
564e-01,8.43295854e-01},prob(percent)="84",prob+-sd="84+-1"]:
1.322701e-01[&length_mean=1.36043751e-01,length_median=1.32270100e-0
1,length_95%HPD={1.74773700e-02,2.58943700e-01}],
127[&prob=1.00000000e+00,prob_stddev=0.00000000e+00,prob_range={1.00
000000e+00,1.00000000e+00},prob(percent)="100",prob+-sd="100+-0"]:
9.256889e-01[&length_mean=9.20181765e-01,length_median=9.25688900e-0
1,length_95%HPD={6.73629400e-01,1.15402800e+00}])
[&prob=6.23594507e-01,prob_stddev=6.00228413e-03,prob_range={6.19350
251e-01,6.27838763e-01},prob(percent)="62",prob+-sd="62+-1"]:
1.157381e-01[&length_mean=1.18131269e-01,length_median=1.15738100e-0
1,length_95%HPD={1.09365100e-02,2.24161100e-01}],
(125[&prob=1.00000000e+00,prob_stddev=0.00000000e+00,prob_range={1.0
0000000e+00,1.00000000e+00},prob(percent)="100",prob+-sd="100+-0"]:
1.380714e-01[&length_mean=1.43456151e-01,length_median=1.38071400e-0
1,length_95%HPD={4.65451800e-02,2.45753100e-01}],
126[&prob=1.00000000e+00,prob_stddev=0.00000000e+00,prob_range={1.00
000000e+00,1.00000000e+00},prob(percent)="100",prob+-sd="100+-0"]:
1.359575e-01[&length_mean=1.37887025e-01,length_median=1.35957500e-0
1,length_95%HPD={5.19818400e-02,2.34663400e-01}])
[&prob=1.00000000e+00,prob_stddev=0.00000000e+00,prob_range={1.00000
000e+00,1.00000000e+00},prob(percent)="100",prob+-sd="100+-0"]:
5.680697e-01[&length_mean=5.70423511e-01,length_median=5.68069700e-0
1,length_95%HPD={4.27024000e-01,7.11556200e-01}])
[&prob=9.98733390e-01,prob_stddev=1.28844843e-03,prob_range={9.97822
319e-01,9.99644460e-01},prob(percent)="100",prob+-sd="100+-0"]:
3.704748e-01[&length_mean=3.72712209e-01,length_median=3.70474800e-0
1,length_95%HPD={2.22717600e-01,5.16668900e-01}])
[&prob=6.14950447e-01,prob_stddev=6.29768450e-02,prob_range={5.70419
092e-01,6.59481801e-01},prob(percent)="61",prob+-sd="61+-6"]:
1.819967e-01[&length_mean=1.75584455e-01,length_median=1.81996700e-0
1,length_95%HPD={7.56636600e-03,3.26945100e-01}])
[&prob=6.52504333e-01,prob_stddev=7.96952490e-02,prob_range={5.96151
282e-01,7.08857384e-01},prob(percent)="65",prob+-sd="65+-8"]:
2.142621e-01[&length_mean=2.04575128e-01,length_median=2.14262100e-0
1,length_95%HPD={2.31933000e-02,3.54798100e-01}],
((118[&prob=1.00000000e+00,prob_stddev=0.00000000e+00,prob_range={1.
00000000e+00,1.00000000e+00},prob(percent)="100",prob+-sd="100+-0"]:
6.628179e-01[&length_mean=6.63806114e-01,length_median=6.62817900e-0
1,length_95%HPD={4.91778200e-01,8.19988700e-01}],
119[&prob=1.00000000e+00,prob_stddev=0.00000000e+00,prob_range={1.00
000000e+00,1.00000000e+00},prob(percent)="100",prob+-sd="100+-0"]:
5.859685e-01[&length_mean=5.86687828e-01,length_median=5.85968500e-0
1,length_95%HPD={4.48420700e-01,7.28537600e-01}])
[&prob=9.96444602e-01,prob_stddev=2.19979000e-03,prob_range={9.94889
116e-01,9.98000089e-01},prob(percent)="100",prob+-sd="100+-0"]:
3.079129e-01[&length_mean=3.13230982e-01,length_median=3.07912900e-0
1,length_95%HPD={1.20686500e-01,4.96131200e-01}],
132[&prob=1.00000000e+00,prob_stddev=0.00000000e+00,prob_range={1.00
000000e+00,1.00000000e+00},prob(percent)="100",prob+-sd="100+-0"]:
1.112771e+00[&length_mean=1.11625991e+00,length_median=1.11277100e+0
0,length_95%HPD={7.19467400e-01,1.47346500e+00}],
144[&prob=1.00000000e+00,prob_stddev=0.00000000e+00,prob_range={1.00
000000e+00,1.00000000e+00},prob(percent)="100",prob+-sd="100+-0"]:

```
1.280834e+00[&length_mean=1.27345432e+00,length_median=1.28083400e+0
0,length_95%HPD={9.38539500e-01,1.59713900e+00}])
[&prob=5.87196125e-01,prob_stddev=4.99352329e-02,prob_range={5.51886
583e-01,6.22505666e-01},prob(percent)="59",prob+-sd="59+-5"]:
1.421162e-01[&length_mean=1.48130802e-01,length_median=1.42116200e-0
1,length_95%HPD={1.29825800e-02,2.84133200e-01}])
[&prob=7.71899027e-01,prob_stddev=8.89029414e-02,prob_range={7.09035
154e-01,8.34762899e-01},prob(percent)="77",prob+-sd="77+-9"]:
1.593774e-01[&length_mean=1.57299189e-01,length_median=1.59377400e-0
1,length_95%HPD={5.59529600e-02,2.64131700e-01}],
((80[&prob=1.00000000e+00,prob_stddev=0.00000000e+00,prob_range={1.0
0000000e+00,1.00000000e+00},prob(percent)="100",prob+-sd="100+-0"]:
6.828860e-01[&length_mean=6.88453364e-01,length_median=6.82886000e-0
1,length_95%HPD={5.44014800e-01,8.49426600e-01}],
(81[&prob=1.00000000e+00,prob_stddev=0.00000000e+00,prob_range={1.00
000000e+00,1.00000000e+00},prob(percent)="100",prob+-sd="100+-0"]:
5.394663e-01[&length_mean=5.40650073e-01,length_median=5.39466300e-0
1,length_95%HPD={3.88376400e-01,6.96740700e-01}],
(((82[&prob=1.00000000e+00,prob_stddev=0.00000000e+00,prob_range={1.
00000000e+00,1.00000000e+00},prob(percent)="100",prob+-sd="100+-0"]:
4.261865e-02[&length_mean=4.42617585e-02,length_median=4.26186500e-0
2,length_95%HPD={1.44383900e-02,7.63752900e-02}],
83[&prob=1.00000000e+00,prob_stddev=0.00000000e+00,prob_range={1.000
00000e+00,1.00000000e+00},prob(percent)="100",prob+-sd="100+-0"]:
1.803833e-01[&length_mean=1.81353280e-01,length_median=1.80383300e-0
1,length_95%HPD={1.17504300e-01,2.45447400e-01}])
[&prob=9.99355584e-01,prob_stddev=4.08532428e-04,prob_range={9.99066
708e-01,9.99644460e-01},prob(percent)="100",prob+-sd="100+-0"]:
6.044188e-02[&length_mean=6.25161694e-02,length_median=6.04418800e-0
2,length_95%HPD={1.86415800e-02,1.07219700e-01}],
84[&prob=1.00000000e+00,prob_stddev=0.00000000e+00,prob_range={1.000
00000e+00,1.00000000e+00},prob(percent)="100",prob+-sd="100+-0"]:
1.294179e-01[&length_mean=1.30091794e-01,length_median=1.29417900e-0
1,length_95%HPD={8.52018000e-02,1.75139000e-01}])
[&prob=9.98000089e-01,prob_stddev=9.42767141e-04,prob_range={9.97333
452e-01,9.98666726e-01},prob(percent)="100",prob+-sd="100+-0"]:
7.722143e-02[&length_mean=7.82553979e-02,length_median=7.72214300e-0
2,length_95%HPD={2.95276400e-02,1.24181400e-01}],
85[&prob=1.00000000e+00,prob_stddev=0.00000000e+00,prob_range={1.000
00000e+00,1.00000000e+00},prob(percent)="100",prob+-sd="100+-0"]:
5.302415e-02[&length_mean=5.47972778e-02,length_median=5.30241500e-0
2,length_95%HPD={1.54718000e-02,9.76853000e-02}])
[&prob=1.00000000e+00,prob_stddev=0.00000000e+00,prob_range={1.00000
000e+00,1.00000000e+00},prob(percent)="100",prob+-sd="100+-0"]:
2.285987e-01[&length_mean=2.31033757e-01,length_median=2.28598700e-0
1,length_95%HPD={1.00407000e-01,3.63515500e-01}])
[&prob=1.00000000e+00,prob_stddev=0.00000000e+00,prob_range={1.00000
000e+00,1.00000000e+00},prob(percent)="100",prob+-sd="100+-0"]:
4.631172e-01[&length_mean=4.62820893e-01,length_median=4.63117200e-0
1,length_95%HPD={3.02311000e-01,6.26510100e-01}])
[&prob=9.99711124e-01,prob_stddev=3.45681285e-04,prob_range={9.99466
690e-01,9.99955558e-01},prob(percent)="100",prob+-sd="100+-0"]:
2.465240e-01[&length_mean=2.49868024e-01,length_median=2.46524000e-0
1,length_95%HPD={1.25146700e-01,3.75604500e-01}],
```

((((86[&prob=1.00000000e+00,prob_stddev=0.00000000e+00,prob_range={1
.00000000e+00,1.00000000e+00},prob(percent)="100",prob+-
sd="100+-0"]:
8.859441e-02[&length_mean=9.07470126e-02,length_median=8.85944100e-0
2,length_95%HPD={2.93522100e-02,1.49527800e-01}],
88[&prob=1.00000000e+00,prob_stddev=0.00000000e+00,prob_range={1.000
00000e+00,1.00000000e+00},prob(percent)="100",prob+-sd="100+-0"]:
1.403057e-01[&length_mean=1.42819904e-01,length_median=1.40305700e-0
1,length_95%HPD={8.54608600e-02,2.05818900e-01}])
[&prob=9.99933336e-01,prob_stddev=9.42767141e-05,prob_range={9.99866
673e-01,1.00000000e+00},prob(percent)="100",prob+-sd="100+-0"]:
1.274296e-01[&length_mean=1.31453829e-01,length_median=1.27429600e-0
1,length_95%HPD={4.51184700e-02,2.21665900e-01}],
87[&prob=1.00000000e+00,prob_stddev=0.00000000e+00,prob_range={1.000
00000e+00,1.00000000e+00},prob(percent)="100",prob+-sd="100+-0"]:
8.878818e-02[&length_mean=9.39457941e-02,length_median=8.87881800e-0
2,length_95%HPD={2.55677700e-02,1.71129000e-01}])
[&prob=1.00000000e+00,prob_stddev=0.00000000e+00,prob_range={1.00000
000e+00,1.00000000e+00},prob(percent)="100",prob+-sd="100+-0"]:
2.909567e-01[&length_mean=2.92878429e-01,length_median=2.90956700e-0
1,length_95%HPD={1.73661200e-01,4.08106300e-01}],
89[&prob=1.00000000e+00,prob_stddev=0.00000000e+00,prob_range={1.000
00000e+00,1.00000000e+00},prob(percent)="100",prob+-sd="100+-0"]:
3.278853e-01[&length_mean=3.29788496e-01,length_median=3.27885300e-0
1,length_95%HPD={2.37846800e-01,4.15413100e-01}])
[&prob=9.94666904e-01,prob_stddev=2.82830142e-03,prob_range={9.92666
993e-01,9.96666815e-01},prob(percent)="99",prob+-sd="99+-0"]:
1.777637e-01[&length_mean=1.80398799e-01,length_median=1.77763700e-0
1,length_95%HPD={9.11522100e-02,2.75330300e-01}],
(90[&prob=1.00000000e+00,prob_stddev=0.00000000e+00,prob_range={1.00
000000e+00,1.00000000e+00},prob(percent)="100",prob+-sd="100+-0"]:
1.205075e-01[&length_mean=1.21484285e-01,length_median=1.20507500e-0
1,length_95%HPD={6.75472200e-02,1.71430800e-01}],
91[&prob=1.00000000e+00,prob_stddev=0.00000000e+00,prob_range={1.000
00000e+00,1.00000000e+00},prob(percent)="100",prob+-sd="100+-0"]:
1.019359e-01[&length_mean=1.03205449e-01,length_median=1.01935900e-0
1,length_95%HPD={4.51061700e-02,1.56468100e-01}])
[&prob=1.00000000e+00,prob_stddev=0.00000000e+00,prob_range={1.00000
000e+00,1.00000000e+00},prob(percent)="100",prob+-sd="100+-0"]:
4.248079e-01[&length_mean=4.28145639e-01,length_median=4.24807900e-0
1,length_95%HPD={3.21702900e-01,5.54521300e-01}])
[&prob=9.99911115e-01,prob_stddev=1.25702285e-04,prob_range={9.99822
230e-01,1.00000000e+00},prob(percent)="100",prob+-sd="100+-0"]:
4.199921e-01[&length_mean=4.21967572e-01,length_median=4.19992100e-0
1,length_95%HPD={2.91265400e-01,5.52118100e-01}])
[&prob=9.61512822e-01,prob_stddev=5.15379370e-03,prob_range={9.57868
539e-01,9.65157104e-01},prob(percent)="96",prob+-sd="96+-1"]:
1.881115e-01[&length_mean=1.87863015e-01,length_median=1.88111500e-0
1,length_95%HPD={8.05817500e-02,3.02816800e-01}],
(94[&prob=1.00000000e+00,prob_stddev=0.00000000e+00,prob_range={1.00
000000e+00,1.00000000e+00},prob(percent)="100",prob+-sd="100+-0"]:
9.497957e-01[&length_mean=9.62682721e-01,length_median=9.49795700e-0
1,length_95%HPD={7.17038600e-01,1.24835800e+00}],
145[&prob=1.00000000e+00,prob_stddev=0.00000000e+00,prob_range={1.00

000000e+00,1.00000000e+00},prob(percent)="100",prob+-sd="100+-0"]:
1.087400e+00[&length_mean=1.09442218e+00,length_median=1.08740000e+0
0,length_95%HPD={8.57491700e-01,1.36944100e+00}])
[&prob=7.18123639e-01,prob_stddev=4.33987140e-02,prob_range={6.87436
114e-01,7.48811164e-01},prob(percent)="72",prob+-sd="72+-4"]:
1.946085e-01[&length_mean=1.99247333e-01,length_median=1.94608500e-0
1,length_95%HPD={2.79063200e-03,3.69588900e-01}],
(((((133[&prob=1.00000000e+00,prob_stddev=0.00000000e+00,prob_range=
{1.00000000e+00,1.00000000e+00},prob(percent)="100",prob+-
sd="100+-0"]:
3.748362e-01[&length_mean=3.77755390e-01,length_median=3.74836200e-0
1,length_95%HPD={2.46109300e-01,4.96453100e-01}],
(134[&prob=1.00000000e+00,prob_stddev=0.00000000e+00,prob_range={1.0
0000000e+00,1.00000000e+00},prob(percent)="100",prob+-sd="100+-0"]:
5.006010e-01[&length_mean=5.01596094e-01,length_median=5.00601000e-0
1,length_95%HPD={3.62141000e-01,6.35512200e-01}],
(135[&prob=1.00000000e+00,prob_stddev=0.00000000e+00,prob_range={1.0
0000000e+00,1.00000000e+00},prob(percent)="100",prob+-sd="100+-0"]:
4.893471e-01[&length_mean=4.92282892e-01,length_median=4.89347100e-0
1,length_95%HPD={3.58003700e-01,6.27357900e-01}],
136[&prob=1.00000000e+00,prob_stddev=0.00000000e+00,prob_range={1.00
000000e+00,1.00000000e+00},prob(percent)="100",prob+-sd="100+-0"]:
5.311388e-01[&length_mean=5.36882126e-01,length_median=5.31138800e-0
1,length_95%HPD={4.01107400e-01,6.75926700e-01}])
[&prob=5.71885694e-01,prob_stddev=9.67907598e-03,prob_range={5.65041
554e-01,5.78729834e-01},prob(percent)="57",prob+-sd="57+-1"]:
1.115897e-01[&length_mean=1.14908143e-01,length_median=1.11589700e-0
1,length_95%HPD={1.05184800e-02,2.15532400e-01}])
[&prob=8.24474468e-01,prob_stddev=3.22112106e-02,prob_range={8.01697
702e-01,8.47251233e-01},prob(percent)="82",prob+-sd="82+-3"]:
1.039030e-01[&length_mean=1.07579279e-01,length_median=1.03903000e-0
1,length_95%HPD={1.44832500e-02,2.06790500e-01}])
[&prob=1.00000000e+00,prob_stddev=0.00000000e+00,prob_range={1.00000
000e+00,1.00000000e+00},prob(percent)="100",prob+-sd="100+-0"]:
3.655434e-01[&length_mean=3.69360403e-01,length_median=3.65543400e-0
1,length_95%HPD={2.15822600e-01,5.25841500e-01}],
(140[&prob=1.00000000e+00,prob_stddev=0.00000000e+00,prob_range={1.0
0000000e+00,1.00000000e+00},prob(percent)="100",prob+-sd="100+-0"]:
6.659628e-01[&length_mean=6.73181900e-01,length_median=6.65962800e-0
1,length_95%HPD={4.80768400e-01,8.83938500e-01}],
143[&prob=1.00000000e+00,prob_stddev=0.00000000e+00,prob_range={1.00
000000e+00,1.00000000e+00},prob(percent)="100",prob+-sd="100+-0"]:
8.781525e-01[&length_mean=8.84530586e-01,length_median=8.78152500e-0
1,length_95%HPD={6.70520100e-01,1.13578500e+00}])
[&prob=9.29669792e-01,prob_stddev=1.55556578e-02,prob_range={9.18670
281e-01,9.40669304e-01},prob(percent)="93",prob+-sd="93+-2"]:
1.988073e-01[&length_mean=2.03715715e-01,length_median=1.98807300e-0
1,length_95%HPD={4.08760800e-02,3.60406000e-01}])
[&prob=5.13288298e-01,prob_stddev=3.56680235e-02,prob_range={4.88067
197e-01,5.38509400e-01},prob(percent)="51",prob+-sd="51+-4"]:
9.950894e-02[&length_mean=1.06665270e-01,length_median=9.95089400e-0
2,length_95%HPD={1.12848700e-03,2.19839800e-01}],
((137[&prob=1.00000000e+00,prob_stddev=0.00000000e+00,prob_range={1.
00000000e+00,1.00000000e+00},prob(percent)="100",prob+-sd="100+-0"]:

4.962364e-01[&length_mean=4.99235287e-01,length_median=4.96236400e-0
1,length_95%HPD={3.36663300e-01,6.57088500e-01}],
138[&prob=1.00000000e+00,prob_stddev=0.00000000e+00,prob_range={1.00
000000e+00,1.00000000e+00},prob(percent)="100",prob+-sd="100+-0"]:
5.704643e-01[&length_mean=5.74461204e-01,length_median=5.70464300e-0
1,length_95%HPD={4.09492300e-01,7.52417200e-01}])
[&prob=9.95866850e-01,prob_stddev=2.26264114e-03,prob_range={9.94266
921e-01,9.97466779e-01},prob(percent)="100",prob+-sd="100+-0"]:
2.346618e-01[&length_mean=2.36752251e-01,length_median=2.34661800e-0
1,length_95%HPD={6.61354900e-02,3.95847400e-01}],
139[&prob=1.00000000e+00,prob_stddev=0.00000000e+00,prob_range={1.00
000000e+00,1.00000000e+00},prob(percent)="100",prob+-sd="100+-0"]:
5.036984e-01[&length_mean=5.10753582e-01,length_median=5.03698400e-0
1,length_95%HPD={3.12097300e-01,7.09136500e-01}])
[&prob=9.95711302e-01,prob_stddev=9.42767141e-05,prob_range={9.95644
638e-01,9.95777965e-01},prob(percent)="100",prob+-sd="100+-0"]:
2.431754e-01[&length_mean=2.50277144e-01,length_median=2.43175400e-0
1,length_95%HPD={8.63957000e-02,4.34191800e-01}])
[&prob=9.99555575e-01,prob_stddev=0.00000000e+00,prob_range={9.99555
575e-01,9.99555575e-01},prob(percent)="100",prob+-sd="100+-0"]:
3.469451e-01[&length_mean=3.47829015e-01,length_median=3.46945100e-0
1,length_95%HPD={1.44821000e-01,5.31780600e-01}],
(141[&prob=1.00000000e+00,prob_stddev=0.00000000e+00,prob_range={1.0
0000000e+00,1.00000000e+00},prob(percent)="100",prob+-sd="100+-0"]:
5.717585e-02[&length_mean=5.94520516e-02,length_median=5.71758500e-0
2,length_95%HPD={1.12255700e-02,1.05166700e-01}],
142[&prob=1.00000000e+00,prob_stddev=0.00000000e+00,prob_range={1.00
000000e+00,1.00000000e+00},prob(percent)="100",prob+-sd="100+-0"]:
5.361614e-02[&length_mean=5.51284611e-02,length_median=5.36161400e-0
2,length_95%HPD={1.03188500e-02,1.01418000e-01}])
[&prob=1.00000000e+00,prob_stddev=0.00000000e+00,prob_range={1.00000
000e+00,1.00000000e+00},prob(percent)="100",prob+-sd="100+-0"]:
8.376124e-01[&length_mean=8.37753072e-01,length_median=8.37612400e-0
1,length_95%HPD={6.16815400e-01,1.03533300e+00}],
149[&prob=1.00000000e+00,prob_stddev=0.00000000e+00,prob_range={1.00
000000e+00,1.00000000e+00},prob(percent)="100",prob+-sd="100+-0"]:
1.134152e+00[&length_mean=1.13238661e+00,length_median=1.13415200e+0
0,length_95%HPD={6.85192800e-01,1.53266700e+00}])
[&prob=6.19839118e-01,prob_stddev=5.13493836e-02,prob_range={5.83529
621e-01,6.56148616e-01},prob(percent)="62",prob+-sd="62+-5"]:
1.436425e-01[&length_mean=1.50662580e-01,length_median=1.43642500e-0
1,length_95%HPD={1.75634200e-02,2.76010100e-01}],
(146[&prob=1.00000000e+00,prob_stddev=0.00000000e+00,prob_range={1.0
0000000e+00,1.00000000e+00},prob(percent)="100",prob+-sd="100+-0"]:
1.099846e+00[&length_mean=1.08880911e+00,length_median=1.09984600e+0
0,length_95%HPD={6.51525000e-01,1.50854800e+00}],
(148[&prob=1.00000000e+00,prob_stddev=0.00000000e+00,prob_range={1.0
0000000e+00,1.00000000e+00},prob(percent)="100",prob+-sd="100+-0"]:
9.326915e-01[&length_mean=9.40655843e-01,length_median=9.32691500e-0
1,length_95%HPD={7.05446400e-01,1.26454900e+00}],
(154[&prob=1.00000000e+00,prob_stddev=0.00000000e+00,prob_range={1.0
0000000e+00,1.00000000e+00},prob(percent)="100",prob+-sd="100+-0"]:
1.201335e+00[&length_mean=1.20156981e+00,length_median=1.20133500e+0
0,length_95%HPD={7.89352100e-01,1.57269400e+00}],

156[&prob=1.00000000e+00,prob_stddev=0.00000000e+00,prob_range={1.00
000000e+00,1.00000000e+00},prob(percent)="100",prob+-sd="100+-0"]:
1.381498e+00[&length_mean=1.38892905e+00,length_median=1.38149800e+0
0,length_95%HPD={9.74470100e-01,1.81354200e+00}])
[&prob=6.59637349e-01,prob_stddev=4.43100556e-03,prob_range={6.56504
155e-01,6.62770544e-01},prob(percent)="66",prob+-sd="66+-0"]:
2.682752e-01[&length_mean=2.85019583e-01,length_median=2.68275200e-0
1,length_95%HPD={1.48059100e-03,5.87812700e-01}])
[&prob=5.90418204e-01,prob_stddev=1.99238122e-02,prob_range={5.76329
941e-01,6.04506466e-01},prob(percent)="59",prob+-sd="59+-2"]:
1.730041e-01[&length_mean=1.79611189e-01,length_median=1.73004100e-0
1,length_95%HPD={9.36553900e-03,3.44667400e-01}],
(150[&prob=1.00000000e+00,prob_stddev=0.00000000e+00,prob_range={1.0
0000000e+00,1.00000000e+00},prob(percent)="100",prob+-sd="100+-0"]:
6.709300e-01[&length_mean=7.09252491e-01,length_median=6.70930000e-0
1,length_95%HPD={2.42614200e-01,1.25078300e+00}],
152[&prob=1.00000000e+00,prob_stddev=0.00000000e+00,prob_range={1.00
000000e+00,1.00000000e+00},prob(percent)="100",prob+-sd="100+-0"]:
1.047165e+00[&length_mean=1.06214303e+00,length_median=1.04716500e+0
0,length_95%HPD={6.57228400e-01,1.51527300e+00}])
[&prob=8.14563797e-01,prob_stddev=7.01104497e-02,prob_range={7.64988
223e-01,8.64139372e-01},prob(percent)="81",prob+-sd="81+-7"]:
3.975011e-01[&length_mean=3.99789800e-01,length_median=3.97501100e-0
1,length_95%HPD={5.07248500e-02,7.65402800e-01}],
151[&prob=1.00000000e+00,prob_stddev=0.00000000e+00,prob_range={1.00
000000e+00,1.00000000e+00},prob(percent)="100",prob+-sd="100+-0"]:
9.133542e-01[&length_mean=9.23219110e-01,length_median=9.13354200e-0
1,length_95%HPD={6.25234100e-01,1.25414500e+00}],
(153[&prob=1.00000000e+00,prob_stddev=0.00000000e+00,prob_range={1.0
0000000e+00,1.00000000e+00},prob(percent)="100",prob+-sd="100+-0"]:
6.046426e-01[&length_mean=6.09570924e-01,length_median=6.04642600e-0
1,length_95%HPD={3.46648300e-01,8.90151100e-01}],
155[&prob=1.00000000e+00,prob_stddev=0.00000000e+00,prob_range={1.00
000000e+00,1.00000000e+00},prob(percent)="100",prob+-sd="100+-0"]:
1.312487e+00[&length_mean=1.32382713e+00,length_median=1.31248700e+0
0,length_95%HPD={9.81531000e-01,1.68389800e+00}])
[&prob=8.01319941e-01,prob_stddev=2.06466004e-02,prob_range={7.86720
590e-01,8.15919292e-01},prob(percent)="80",prob+-sd="80+-2"]:
2.508030e-01[&length_mean=2.60449281e-01,length_median=2.50803000e-0
1,length_95%HPD={1.61040900e-02,4.95066000e-01}])
[&prob=6.19483579e-01,prob_stddev=1.59830456e-01,prob_range={5.06466
379e-01,7.32500778e-01},prob(percent)="62",prob+-sd="62+-16"]:
2.210565e-01[&length_mean=2.25268645e-01,length_median=2.21056500e-0
1,length_95%HPD={7.33210300e-02,3.87478700e-01}])
[&prob=5.79285365e-01,prob_stddev=2.93829092e-02,prob_range={5.58508
511e-01,6.00062219e-01},prob(percent)="58",prob+-sd="58+-3"]:
1.568041e-01[&length_mean=1.59283692e-01,length_median=1.56804100e-0
1,length_95%HPD={4.95441200e-02,2.66684500e-01}],
147[&prob=1.00000000e+00,prob_stddev=0.00000000e+00,prob_range={1.00
000000e+00,1.00000000e+00},prob(percent)="100",prob+-sd="100+-0"]:
1.107353e+00[&length_mean=1.10551177e+00,length_median=1.10735300e+0
0,length_95%HPD={8.52640900e-01,1.36529600e+00}])
[&prob=8.24341140e-01,prob_stddev=1.87453533e-01,prob_range={6.91791
476e-01,9.56890805e-01},prob(percent)="82",prob+-sd="82+-19"]:

1.762198e-01[&length_mean=1.79520072e-01,length_median=1.76219800e-0
1,length_95%HPD={6.86477900e-02,2.96684900e-01}])
[&prob=6.30127550e-01,prob_stddev=4.11989241e-02,prob_range={6.00995
511e-01,6.59259588e-01},prob(percent)="63",prob+-sd="63+-4"]:
1.123241e-01[&length_mean=1.13892966e-01,length_median=1.12324100e-0
1,length_95%HPD={2.46462900e-02,1.97513000e-01}])
[&prob=9.99977779e-01,prob_stddev=3.14255714e-05,prob_range={9.99955
558e-01,1.00000000e+00},prob(percent)="100",prob+-sd="100+-0"]:
2.464715e-01[&length_mean=2.49927574e-01,length_median=2.46471500e-0
1,length_95%HPD={1.23371000e-01,3.72099500e-01}],
((128[&prob=1.00000000e+00,prob_stddev=0.00000000e+00,prob_range={1.
00000000e+00,1.00000000e+00},prob(percent)="100",prob+-sd="100+-0"]:
1.051305e+00[&length_mean=1.05436527e+00,length_median=1.05130500e+0
0,length_95%HPD={8.06979300e-01,1.30978900e+00}],
129[&prob=1.00000000e+00,prob_stddev=0.00000000e+00,prob_range={1.00
000000e+00,1.00000000e+00},prob(percent)="100",prob+-sd="100+-0"]:
6.836072e-01[&length_mean=6.81826812e-01,length_median=6.83607200e-0
1,length_95%HPD={4.79897300e-01,8.82478100e-01}])
[&prob=6.04217590e-01,prob_stddev=1.61213181e-02,prob_range={5.92818
097e-01,6.15617084e-01},prob(percent)="60",prob+-sd="60+-2"]:
1.558441e-01[&length_mean=1.60085686e-01,length_median=1.55844100e-0
1,length_95%HPD={2.10003500e-02,3.01447300e-01}],
(130[&prob=1.00000000e+00,prob_stddev=0.00000000e+00,prob_range={1.0
0000000e+00,1.00000000e+00},prob(percent)="100",prob+-sd="100+-0"]:
4.413555e-01[&length_mean=4.42708600e-01,length_median=4.41355500e-0
1,length_95%HPD={3.12155900e-01,5.65107100e-01}],
131[&prob=1.00000000e+00,prob_stddev=0.00000000e+00,prob_range={1.00
000000e+00,1.00000000e+00},prob(percent)="100",prob+-sd="100+-0"]:
2.582015e-01[&length_mean=2.60978245e-01,length_median=2.58201500e-0
1,length_95%HPD={1.52025200e-01,3.69729800e-01}])
[&prob=1.00000000e+00,prob_stddev=0.00000000e+00,prob_range={1.00000
000e+00,1.00000000e+00},prob(percent)="100",prob+-sd="100+-0"]:
4.876773e-01[&length_mean=4.88990841e-01,length_median=4.87677300e-0
1,length_95%HPD={2.80437900e-01,6.66864300e-01}])
[&prob=9.49424470e-01,prob_stddev=3.77106856e-04,prob_range={9.49157
815e-01,9.49691125e-01},prob(percent)="95",prob+-sd="95+-0"]:
1.463799e-01[&length_mean=1.50983164e-01,length_median=1.46379900e-0
1,length_95%HPD={1.72645200e-02,2.74987100e-01}])
[&prob=9.99977779e-01,prob_stddev=3.14255714e-05,prob_range={9.99955
558e-01,1.00000000e+00},prob(percent)="100",prob+-sd="100+-0"]:
4.940437e-01[&length_mean=5.00254149e-01,length_median=4.94043700e-0
1,length_95%HPD={3.28630300e-01,6.70907900e-01}])
[&prob=1.00000000e+00,prob_stddev=0.00000000e+00,prob_range={1.00000
000e+00,1.00000000e+00},prob(percent)="100",prob+-sd="100+-0"]:
3.383230e-01[&length_mean=3.41849323e-01,length_median=3.38323000e-0
1,length_95%HPD={1.92713700e-01,4.84063200e-01}])
[&prob=1.00000000e+00,prob_stddev=0.00000000e+00,prob_range={1.00000
000e+00,1.00000000e+00},prob(percent)="100",prob+-sd="100+-0"]:
3.058360e-01[&length_mean=3.08591559e-01,length_median=3.05836000e-0
1,length_95%HPD={1.85042000e-01,4.27536500e-01}]);
end;

Fig4C(Fig_S6)_RAxML_tree_file

```
#NEXUS
begin taxa;
	dimensions ntax=156;
	taxlabels
	Aaur_20911[&!color=#cc6600]
	Adig_03889[&!color=#cc6600]
	Adig_04407[&!color=#cc6600]
	Adig_06508[&!color=#cc6600]
	Adig_07747[&!color=#cc6600]
	Adig_10295[&!color=#cc6600]
	Adig_19031[&!color=#cc6600]
	Adig_20275[&!color=#cc6600]
	Adig_20773[&!color=#cc6600]
	Aele_124411[&!color=#cc6600]
	Aele_151938[&!color=#cc6600]
	Aele_151940[&!color=#cc6600]
	Aele_151942[&!color=#cc6600]
	Aele_7590[&!color=#cc6600]
	Aele_79976[&!color=#cc6600]
	Apal_288431[&!color=#cc6600]
	Apal_288439[&!color=#cc6600]
	Apal_344094[&!color=#cc6600]
	Apal_344111[&!color=#cc6600]
	Apal_398190[&!color=#cc6600]
	Apal_400465[&!color=#cc6600]
	Apal_41158[&!color=#cc6600]
	Apal_54304[&!color=#cc6600]
	Apal_8979[&!color=#cc6600]
	Apal_89985[&!color=#cc6600]
	Aque_39602[&!color=#0000ff]
	Aspe_Cu_472242
	Atet_141600[&!color=#cc6600]
	Atet_192642[&!color=#cc6600]
	Atet_40821[&!color=#cc6600]
	Atet_41310[&!color=#cc6600]
	Atet_66572[&!color=#cc6600]
	Atet_88599[&!color=#cc6600]
	Atha_Blue_copper
	BfEfn1[&!color=#6666ff]
	BfEfn2[&!color=#6666ff]
	Btue_12517[&!color=#cc6600]
	Btue_23164[&!color=#cc6600]
	Btue_28719[&!color=#cc6600]
	Btue_29916[&!color=#cc6600]
	Btue_30708[&!color=#cc6600]
	Btue_9750[&!color=#cc6600]
	Btue_9751[&!color=#cc6600]
	Ccan_Cu_114223
	CiEfnAa[&!color=#6666ff]
	CiEfnAb[&!color=#6666ff]
	CiEfnAc[&!color=#6666ff]
	CiEfnAd[&!color=#6666ff]
	CiEfnB[&!color=#6666ff]
	Clat_120953[&!color=#cc6600]
```

```
Clat_22532[&!color=#cc6600]
Clat_28862[&!color=#cc6600]
Clat_48253[&!color=#cc6600]
Clat_80728[&!color=#cc6600]
Clat_97078[&!color=#cc6600]
'Cpro_58774.15'[&!color=#0000ff]
Ctel_225002[&!color=#6666ff]
Cvar_1084[&!color=#0000ff]
Cvar_12230[&!color=#0000ff]
Cvar_2778[&!color=#0000ff]
Cvar_8977[&!color=#0000ff]
Dpul_EFX72793[&!color=#6666ff]
'Drer_101723.3'[&!color=#990000]
'Drer_10432.8'[&!color=#990000]
'Drer_111146.1'[&!color=#990000]
'Drer_116412.1'[&!color=#990000]
'Drer_119988.2'[&!color=#990000]
'Drer_12577.6'[&!color=#990000]
'Drer_133669.1'[&!color=#990000]
'Drer_136933.1'[&!color=#990000]
'Drer_141804.1'[&!color=#990000]
'Drer_24428.4'[&!color=#990000]
'Drer_40277.7'[&!color=#990000]
'Drer_50216.6'[&!color=#990000]
'Drer_62002.6'[&!color=#990000]
'Drer_73969.3'[&!color=#990000]
'Drer_79638.5'[&!color=#990000]
EFNA1_HUMAN[&!color=#990000]
EFNA1_MOUSE[&!color=#990000]
EFNA2_HUMAN[&!color=#990000]
EFNA3_HUMAN[&!color=#990000]
EFNA4_HUMAN[&!color=#990000]
EFNA4_MOUSE[&!color=#990000]
EFNA5_HUMAN[&!color=#990000]
EFNB1_HUMAN[&!color=#990000]
EFNB2_MOUSE[&!color=#990000]
EFNB3_HUMAN[&!color=#990000]
Ever_13730[&!color=#cc3300]
Ever_1897[&!color=#cc6600]
Ever_19722[&!color=#cc6600]
Ever_204[&!color=#cc6600]
Hdig_22213[&!color=#cc6600]
Hdig_22696[&!color=#cc6600]
Hdig_30557[&!color=#cc6600]
Hdig_4952[&!color=#cc6600]
Hdig_5881[&!color=#cc6600]
Holi_14653[&!color=#cc6600]
Holi_3973[&!color=#cc6600]
Holi_5987[&!color=#cc6600]
Holi_7726[&!color=#cc6600]
Hpsi_83841[&!color=#6666ff]
Hpsi_83847[&!color=#6666ff]
Hvir_3164[&!color=#cc6600]
'Hvul_1018918.1'[&!color=#cc6600]
```

```
		'Hvul_1019657.1'[&!color=#cc6600]
		'Hvul_1028415.1'[&!color=#cc6600]
		'Hvul_1028689.1'[&!color=#cc6600]
		'Hvul_1029169.1'[&!color=#cc3300]
		'Hvul_1033087.1'[&!color=#cc6600]
		'Hvul_1034474.1'[&!color=#cc6600]
		'Hvul_1038805.1'[&!color=#cc6600]
		Ifas_6298[&!color=#0000ff]
		Ifas_6301[&!color=#0000ff]
		Ifas_Cu_19075
		Kvar_23578[&!color=#0000ff]
		Lapi_139996[&!color=#0000ff]
		Lapi_57857[&!color=#0000ff]
		Lgig_171062[&!color=#6666ff]
		Lith_21086[&!color=#6666ff]
		Mlei_03441[&!color=#cc00ff]
		Nbij_143918[&!color=#cc6600]
		Nbij_20083[&!color=#cc6600]
		Nbij_23770[&!color=#cc6600]
		Nbij_52843[&!color=#cc6600]
		Nbij_57193[&!color=#cc6600]
		Nvec_EDO36327[&!color=#cc6600]
		Nvec_EDO44950[&!color=#cc6600]
		Nvec_EDO47804[&!color=#cc6600]
		Paer_Azurin
		Pcar_179493[&!color=#cc6600]
		Pcar_189454[&!color=#cc6600]
		Pcar_215100[&!color=#cc6600]
		Pcar_235347[&!color=#cc6600]
		Pcar_240721[&!color=#cc6600]
		Pcar_395755[&!color=#cc6600]
		'Pmar_8071.1'[&!color=#990000]
		Pper_103077[&!color=#cc6600]
		Pper_105129[&!color=#cc6600]
		Pper_140147[&!color=#cc6600]
		Pper_41140[&!color=#cc6600]
		Pper_55548[&!color=#cc6600]
		Pphy_13023[&!color=#cc6600]
		Pphy_14332[&!color=#cc6600]
		Pphy_19559[&!color=#cc6600]
		Pphy_29075[&!color=#cc6600]
		Pphy_49294[&!color=#cc6600]
		Skow_16956[&!color=#6666ff]
		Skow_5664[&!color=#6666ff]
		Slac_8385[&!color=#0000ff]
		Snux_75316[&!color=#0000ff]
		Spur_23757[&!color=#6666ff]
		Spyx_Cu_120946
		Tfer_Rusticyanin
		Tpol_19734[&!color=#6666ff]
		Tpol_19735[&!color=#6666ff]
		Uper_Plastocyanin
;
end;
```

```
begin trees;
        tree tree_1 = [&R] ((CiEfnAa[&!color=#6666ff]:1.382333,
(CiEfnAd[&!color=#6666ff]:0.279324,CiEfnAc[&!color=#6666ff]:
0.520955)[&label=100,!color=#6666ff]:0.512761)[&label=46,!
color=#6666ff]:0.163031,((((Skow_16956[&!color=#6666ff]:0.829304,
(CiEfnB[&!color=#6666ff]:1.021176,((('Drer_73969.3'[&!
color=#990000]:0.215272,'Drer_62002.6'[&!color=#990000]:0.13134)
[&label=70,!color=#990000]:0.106932,('Pmar_8071.1'[&!color=#990000]:
0.891119,EFNB3_HUMAN[&!color=#990000]:0.340966)[&label=71,!
color=#990000]:0.233924)[&label=98,!color=#990000]:0.253943,
((EFNB2_MOUSE[&!color=#990000]:0.175971,('Drer_79638.5'[&!
color=#990000]:0.348146,'Drer_10432.8'[&!color=#990000]:0.171034)
[&label=99,!color=#990000]:0.163732)[&label=96,!color=#990000]:
0.120849,('Drer_24428.4'[&!color=#990000]:0.242575,EFNB1_HUMAN[&!
color=#990000]:0.216035)[&label=100,!color=#990000]:0.285957)
[&label=93,!color=#990000]:0.194847)[&label=99,!color=#990000]:
0.432415)[&label=75,!color=#6666ff]:0.127504)[&label=62,!
color=#6666ff]:0.171652,((BfEfn2[&!color=#6666ff]:1.158855,BfEfn1[&!
color=#6666ff]:0.816963)[&label=82,!color=#6666ff]:0.279654,
(Spur_23757[&!color=#6666ff]:0.786628,Skow_5664[&!color=#6666ff]:
1.056756)[&label=23,!color=#6666ff]:0.145142)[&label=27,!
color=#6666ff]:0.185847)[&label=24,!color=#6666ff]:0.025867,
(((((((Atha_Blue_copper:0.773978,Aspe_Cu_472242:1.917022)
[&label=29]:0.229951,(Uper_Plastocyanin:1.240025,Tfer_Rusticyanin:
1.669615)[&label=30]:0.613785)[&label=13]:0.258903,(Snux_75316[&!
color=#0000ff]:1.277662,Paer_Azurin:1.484735)[&label=18]:0.202989)
[&label=6]:0.323141,(Mlei_03441[&!color=#cc00ff]:1.722087,
(Ifas_Cu_19075:2.289539,((Ifas_6301[&!color=#0000ff]:
0.061118,Ifas_6298[&!color=#0000ff]:0.055747)[&label=100,!
color=#0000ff]:1.060106,(Spyx_Cu_120946:1.890789,((Kvar_23578[&!
color=#0000ff]:1.226442,((Lapi_57857[&!color=#0000ff]:
0.75637,'Cpro_58774.15'[&!color=#0000ff]:0.61735)[&label=94,!
color=#0000ff]:0.3608,Slac_8385[&!color=#0000ff]:0.880842)
[&label=74,!color=#0000ff]:0.221035)[&label=32,!color=#0000ff]:
0.09734,(Lapi_139996[&!color=#0000ff]:0.920457,(Cvar_2778[&!
color=#0000ff]:0.432484,((Cvar_8977[&!color=#0000ff]:
0.664288,Cvar_1084[&!color=#0000ff]:0.562815)[&label=53,!
color=#0000ff]:0.13443,Cvar_12230[&!color=#0000ff]:0.584814)
[&label=64,!color=#0000ff]:0.095265)[&label=100,!color=#0000ff]:
0.49089)[&label=32,!color=#0000ff]:0.123283)[&label=76,!
color=#0000ff]:0.275744)[&label=41,!color=#0000ff]:0.166207)
[&label=27,!color=#0000ff]:0.155087)[&label=9]:0.001392)[&label=2]:
0.115909)[&label=0,!color=#cc6600]:0.144933,(((Ever_204[&!
color=#cc6600]:0.467953,Ever_1897[&!color=#cc6600]:0.773048)
[&label=95,!color=#cc6600]:0.568886,(Aaur_20911[&!color=#cc6600]:
0.728255,(Pper_55548[&!color=#cc6600]:0.839379,((Hvir_3164[&!
color=#cc6600]:0.26181,(Holi_5987[&!color=#cc6600]:0.17726,
('Hvul_1038805.1'[&!color=#cc6600]:0.152892,'Hvul_1033087.1'[&!
color=#cc6600]:0.004601)[&label=100,!color=#cc6600]:0.13935)
[&label=100,!color=#cc6600]:0.368617)[&label=100,!color=#cc6600]:
0.814227,((((Aele_151940[&!color=#cc6600]:1.0E-6,Aele_151942[&!
color=#cc6600]:0.177832)[&label=100,!color=#cc6600]:0.219237,
(Aele_151938[&!color=#cc6600]:0.23439,Nbij_52843[&!color=#cc6600]:
```

0.179069)[&label=98,!color=#cc6600]:0.175242)[&label=96,!
color=#cc6600]:0.238294,(Clat_97078[&!color=#cc6600]:0.201814,
(Atet_66572[&!color=#cc6600]:0.386482,Atet_41310[&!color=#cc6600]:
0.395933)[&label=98,!color=#cc6600]:0.241691)[&label=100,!
color=#cc6600]:0.298275)[&label=72,!color=#cc6600]:
0.164557,Pphy_29075[&!color=#cc6600]:0.784111)[&label=71,!
color=#cc6600]:0.24)[&label=89,!color=#cc6600]:0.272846)[&label=45,!
color=#cc6600]:0.163403)[&label=40,!color=#cc6600]:0.18025)
[&label=9,!color=#cc6600]:0.093766,((((((Adig_20773[&!
color=#cc6600]:0.13721,(Adig_06508[&!color=#cc6600]:
0.041371,Adig_10295[&!color=#cc6600]:0.183257)[&label=70,!
color=#cc6600]:0.055436)[&label=77,!color=#cc6600]:
0.082958,Adig_19031[&!color=#cc6600]:0.039642)[&label=84,!
color=#cc6600]:0.251952,Adig_03889[&!color=#cc6600]:0.635112)
[&label=96,!color=#cc6600]:0.513994,Pcar_240721[&!color=#cc6600]:
0.789239)[&label=79,!color=#cc6600]:0.341552,((Btue_9751[&!
color=#cc6600]:0.130144,Btue_9750[&!color=#cc6600]:0.107109)
[&label=100,!color=#cc6600]:0.495465,(Hdig_4952[&!color=#cc6600]:
0.343487,(Apal_8979[&!color=#cc6600]:0.094226,(Apal_288439[&!
color=#cc6600]:0.085017,Apal_288431[&!color=#cc6600]:0.169809)
[&label=89,!color=#cc6600]:0.137536)[&label=99,!color=#cc6600]:
0.353609)[&label=65,!color=#cc6600]:0.168359)[&label=98,!
color=#cc6600]:0.498766)[&label=37,!color=#cc6600]:0.215611,
(Pcar_215100[&!color=#cc6600]:0.93396,(Btue_12517[&!color=#cc6600]:
0.9127,(Apal_344111[&!color=#cc6600]:0.530889,Hdig_22213[&!
color=#cc6600]:0.475074)[&label=80,!color=#cc6600]:0.131955)
[&label=91,!color=#cc6600]:0.523319)[&label=30,!color=#cc6600]:
0.162367)[&label=3,!color=#cc6600]:0.078944,((((Btue_23164[&!
color=#cc6600]:0.762586,(Apal_400465[&!color=#cc6600]:
0.495167,Hdig_5881[&!color=#cc6600]:0.439335)[&label=65,!
color=#cc6600]:0.119595)[&label=75,!color=#cc6600]:0.371741,
(((Hdig_22696[&!color=#cc6600]:0.208583,Apal_344094[&!
color=#cc6600]:0.162523)[&label=100,!color=#cc6600]:0.175573,
(Btue_29916[&!color=#cc6600]:0.002579,Btue_30708[&!color=#cc6600]:
0.054919)[&label=100,!color=#cc6600]:0.293821)[&label=93,!
color=#cc6600]:0.118282,Nvec_EDO44950[&!color=#cc6600]:0.481252)
[&label=90,!color=#cc6600]:0.251051)[&label=22,!color=#cc6600]:
0.100855,(Pcar_189454[&!color=#cc6600]:0.580097,Adig_20275[&!
color=#cc6600]:0.809641)[&label=90,!color=#cc6600]:0.319234)
[&label=11,!color=#cc6600]:0.148591,(((Pcar_235347[&!color=#cc6600]:
0.701291,((Hdig_30557[&!color=#cc6600]:0.186081,Apal_398190[&!
color=#cc6600]:0.128631)[&label=100,!color=#cc6600]:
0.433031,Nvec_EDO36327[&!color=#cc6600]:0.451943)[&label=99,!
color=#cc6600]:0.347697)[&label=40,!color=#cc6600]:0.090681,
(Pcar_395755[&!color=#cc6600]:0.926678,(Adig_07747[&!color=#cc6600]:
0.952091,Apal_41158[&!color=#cc6600]:0.401307)[&label=65,!
color=#cc6600]:0.178826)[&label=12,!color=#cc6600]:0.032319)
[&label=12,!color=#cc6600]:0.111335,(Adig_04407[&!color=#cc6600]:
1.067152,(Apal_54304[&!color=#cc6600]:0.782392,Nvec_EDO47804[&!
color=#cc6600]:0.695301)[&label=70,!color=#cc6600]:0.232862)
[&label=29,!color=#cc6600]:0.109014)[&label=1,!color=#cc6600]:
0.06466)[&label=2,!color=#cc6600]:0.121908)[&label=2,!
color=#cc6600]:0.118597)[&label=2,!color=#cc6600]:0.14586)
[&label=0,!color=#cc6600]:0.059583,(Btue_28719[&!color=#cc6600]:

2.010931,(((Pper_140147[&!color=#cc6600]:0.914387,(((Pper_41140[&!
color=#cc6600]:0.218487,Pper_105129[&!color=#cc6600]:0.161093)
[&label=100,!color=#cc6600]:0.631585,((Pphy_49294[&!color=#cc6600]:
0.850279,((('Hvul_1028415.1'[&!color=#cc6600]:
0.105433,'Hvul_1018918.1'[&!color=#cc6600]:0.019419)[&label=100,!
color=#cc6600]:0.125702,Holi_3973[&!color=#cc6600]:0.229267)
[&label=98,!color=#cc6600]:0.636325,('Hvul_1034474.1'[&!
color=#cc6600]:1.061165,('Hvul_1019657.1'[&!color=#cc6600]:
0.371073,Holi_7726[&!color=#cc6600]:0.123003)[&label=94,!
color=#cc6600]:0.40787)[&label=73,!color=#cc6600]:0.29847)
[&label=52,!color=#cc6600]:0.130797)[&label=15,!color=#cc6600]:
0.094852,(((Nbij_20083[&!color=#cc6600]:0.390075,Aele_79976[&!
color=#cc6600]:0.370181)[&label=64,!color=#cc6600]:0.139623,
((Atet_141600[&!color=#cc6600]:0.564521,Clat_80728[&!color=#cc6600]:
0.259418)[&label=92,!color=#cc6600]:0.190978,(Pphy_19559[&!
color=#cc6600]:0.659524,((Atet_192642[&!color=#cc6600]:
0.861285,Clat_48253[&!color=#cc6600]:0.235521)[&label=95,!
color=#cc6600]:0.239531,(Atet_40821[&!color=#cc6600]:
0.625442,Clat_28862[&!color=#cc6600]:0.244887)[&label=88,!
color=#cc6600]:0.151059)[&label=85,!color=#cc6600]:0.244829)
[&label=59,!color=#cc6600]:0.218534)[&label=19,!color=#cc6600]:
0.051428)[&label=52,!color=#cc6600]:0.168123,(Atet_88599[&!
color=#cc6600]:0.54098,(Clat_120953[&!color=#cc6600]:0.487343,
(Nbij_143918[&!color=#cc6600]:0.466772,Aele_124411[&!color=#cc6600]:
0.310674)[&label=97,!color=#cc6600]:0.296287)[&label=52,!
color=#cc6600]:0.091392)[&label=96,!color=#cc6600]:0.370198)
[&label=45,!color=#cc6600]:0.305397)[&label=56,!color=#cc6600]:
0.195308)[&label=50,!color=#cc6600]:0.125441,Pper_103077[&!
color=#cc6600]:0.867627)[&label=66,!color=#cc6600]:0.161865)
[&label=29,!color=#cc6600]:0.056775,((Holi_14653[&!color=#cc6600]:
0.20765,'Hvul_1028689.1'[&!color=#cc6600]:0.121362)[&label=100,!
color=#cc6600]:0.619574,(Nbij_57193[&!color=#cc6600]:1.076611,
((Aele_7590[&!color=#cc6600]:0.587764,Nbij_23770[&!color=#cc6600]:
0.790268)[&label=57,!color=#cc6600]:0.25037,(Clat_22532[&!
color=#cc6600]:0.821908,(Pphy_13023[&!color=#cc6600]:
0.238658,Pphy_14332[&!color=#cc6600]:0.412756)[&label=78,!
color=#cc6600]:0.192556)[&label=28,!color=#cc6600]:0.12248)
[&label=52,!color=#cc6600]:0.059616)[&label=33,!color=#cc6600]:
0.12378)[&label=73,!color=#cc6600]:0.444923)[&label=33,!
color=#cc6600]:0.269102,(Ever_19722[&!color=#cc6600]:1.86376,
(Apal_89985[&!color=#cc6600]:0.746023,Pcar_179493[&!color=#cc6600]:
0.669958)[&label=71,!color=#cc6600]:0.36463)[&label=23,!
color=#cc6600]:0.214403)[&label=11,!color=#cc6600]:0.160152)
[&label=1,!color=#cc6600]:0.04773)[&label=3]:0.10585,((Aque_39602[&!
color=#0000ff]:1.434008,Ccan_Cu_114223:2.578826)[&label=1]:0.103108,
('Hvul_1029169.1'[&!color=#cc3300]:1.617122,Ever_13730[&!
color=#cc3300]:1.191037)[&label=14,!color=#cc3300]:0.109047)
[&label=1]:0.102065)[&label=22,!color=#6666ff]:0.169631,
(((Hpsi_83847[&!color=#6666ff]:0.040475,Hpsi_83841[&!color=#6666ff]:
0.082964)[&label=100,!color=#6666ff]:0.911594,((Tpol_19734[&!
color=#6666ff]:0.079762,Tpol_19735[&!color=#6666ff]:0.068695)
[&label=99,!color=#6666ff]:0.676052,(Lgig_171062[&!color=#6666ff]:
0.855687,Ctel_225002[&!color=#6666ff]:0.744551)[&label=69,!
color=#6666ff]:0.171354)[&label=65,!color=#6666ff]:0.207052)

```
[&label=54,!color=#6666ff]:0.154001,(Dpul_EFX72793[&!color=#6666ff]:
0.408484,Lith_21086[&!color=#6666ff]:0.239148)[&label=100,!
color=#6666ff]:0.562762)[&label=23,!color=#6666ff]:0.121171)
[&label=13,!color=#6666ff]:0.131099)[&label=61,!color=#6666ff]:
0.273132,((('Drer_141804.1'[&!color=#990000]:0.645809,
(EFNA4_MOUSE[&!color=#990000]:0.151291,EFNA4_HUMAN[&!color=#990000]:
0.085189)[&label=94,!color=#990000]:0.581984)[&label=72,!
color=#990000]:0.313681,((EFNA3_HUMAN[&!color=#990000]:0.343577,
('Drer_133669.1'[&!color=#990000]:0.114842,'Drer_119988.2'[&!
color=#990000]:0.182216)[&label=99,!color=#990000]:0.15804)
[&label=100,!color=#990000]:0.313047,(EFNA2_HUMAN[&!color=#990000]:
0.408971,(('Drer_40277.7'[&!color=#990000]:
0.290193,'Drer_101723.3'[&!color=#990000]:0.222945)[&label=96,!
color=#990000]:0.133505,(EFNA5_HUMAN[&!color=#990000]:0.1726,
('Drer_136933.1'[&!color=#990000]:0.155238,('Drer_116412.1'[&!
color=#990000]:0.175913,'Drer_111146.1'[&!color=#990000]:1.06E-4)
[&label=100,!color=#990000]:0.233338)[&label=78,!color=#990000]:
0.043302)[&label=100,!color=#990000]:0.334009)[&label=52,!
color=#990000]:0.100946)[&label=95,!color=#990000]:0.291116)
[&label=48,!color=#990000]:0.137297)[&label=43,!color=#990000]:
0.142998,(('Drer_12577.6'[&!color=#990000]:
0.288249,'Drer_50216.6'[&!color=#990000]:0.260504)[&label=100,!
color=#990000]:0.307275,(EFNA1_HUMAN[&!color=#990000]:
0.089399,EFNA1_MOUSE[&!color=#990000]:0.102772)[&label=100,!
color=#990000]:0.372129)[&label=98,!color=#990000]:0.447262)
[&label=87,!color=#990000]:0.628712)[&label=51,!color=#6666ff]:
0.174969,CiEfnAb[&!color=#6666ff]:0.920954);
end;

begin figtree;
	set appearance.backgroundColorAttribute="Default";
	set appearance.backgroundColour=#ffffff;
	set appearance.branchColorAttribute="User selection";
	set appearance.branchColorGradient=false;
	set appearance.branchLineWidth=1.0;
	set appearance.branchMinLineWidth=0.0;
	set appearance.branchWidthAttribute="Fixed";
	set appearance.foregroundColour=#000000;
	set appearance.hilightingGradient=false;
	set appearance.selectionColour=#2d3680;
	set branchLabels.colorAttribute="User selection";
	set branchLabels.displayAttribute="Branch times";
	set branchLabels.fontName="sansserif";
	set branchLabels.fontSize=8;
	set branchLabels.fontStyle=0;
	set branchLabels.isShown=false;
	set branchLabels.significantDigits=4;
	set layout.expansion=655;
	set layout.layoutType="RECTILINEAR";
	set layout.zoom=0;
	set legend.attribute="label";
	set legend.fontSize=10.0;
	set legend.isShown=false;
	set legend.significantDigits=4;
```

```
set nodeBars.barWidth=4.0;
set nodeBars.displayAttribute=null;
set nodeBars.isShown=false;
set nodeLabels.colorAttribute="User selection";
set nodeLabels.displayAttribute="label";
set nodeLabels.fontName="sansserif";
set nodeLabels.fontSize=8;
set nodeLabels.fontStyle=0;
set nodeLabels.isShown=true;
set nodeLabels.significantDigits=4;
set nodeShapeExternal.colourAttribute="User selection";
set nodeShapeExternal.isShown=false;
set nodeShapeExternal.minSize=10.0;
set nodeShapeExternal.scaleType=Width;
set nodeShapeExternal.shapeType=Circle;
set nodeShapeExternal.size=4.0;
set nodeShapeExternal.sizeAttribute="Fixed";
set nodeShapeInternal.colourAttribute="User selection";
set nodeShapeInternal.isShown=false;
set nodeShapeInternal.minSize=10.0;
set nodeShapeInternal.scaleType=Width;
set nodeShapeInternal.shapeType=Circle;
set nodeShapeInternal.size=4.0;
set nodeShapeInternal.sizeAttribute="Fixed";
set polarLayout.alignTipLabels=false;
set polarLayout.angularRange=0;
set polarLayout.rootAngle=0;
set polarLayout.rootLength=100;
set polarLayout.showRoot=true;
set radialLayout.spread=0.0;
set rectilinearLayout.alignTipLabels=false;
set rectilinearLayout.curvature=0;
set rectilinearLayout.rootLength=100;
set scale.offsetAge=0.0;
set scale.rootAge=1.0;
set scale.scaleFactor=1.0;
set scale.scaleRoot=false;
set scaleAxis.automaticScale=true;
set scaleAxis.fontSize=8.0;
set scaleAxis.isShown=false;
set scaleAxis.lineWidth=1.0;
set scaleAxis.majorTicks=1.0;
set scaleAxis.minorTicks=0.5;
set scaleAxis.origin=0.0;
set scaleAxis.reverseAxis=false;
set scaleAxis.showGrid=true;
set scaleBar.automaticScale=true;
set scaleBar.fontSize=10.0;
set scaleBar.isShown=true;
set scaleBar.lineWidth=1.0;
set scaleBar.scaleRange=0.0;
set tipLabels.colorAttribute="User selection";
set tipLabels.displayAttribute="Names";
set tipLabels.fontName="Arial";
```

```
        set tipLabels.fontSize=12;
        set tipLabels.fontStyle=1;
        set tipLabels.isShown=true;
        set tipLabels.significantDigits=4;
        set trees.order=true;
        set trees.orderType="decreasing";
        set trees.rooting=true;
        set trees.rootingType="User Selection";
        set trees.transform=false;
        set trees.transformType="cladogram";
end;
```

Fig5C(Fig_S9_FastTree_tree_file

(EFNA1_HUMAN_3CZU_B:0.0831255712,((((EFNA2_HUMAN:
0.0416686816,EFNA2_MOUSE:0.0464178591)97.5/100:0.1991771635,
(EFNA5_HUMAN:0.0868613421,
(Drer_111146.1:0.0000025127,Drer_116412.1:0.1193975234)95.8/100:0.09
00737426)88.6/100:0.1340641560)97.6/100:0.2244522269,(EFNA3_HUMAN:
0.2945088180,(EFNA4_HUMAN:0.0801557281,EFNA4_MOUSE:
0.0604336194)100/100:0.4332334285)57.1/98:0.1187237233)71.4/99:0.068
5623103,(((((((EFNB1_HUMAN:
0.0843966698,Drer_24428.4:0.1821519373)90.7/100:0.0919027999,
(EFNB3_HUMAN:0.1603355346,
(Drer_62002.6:0.1276065593,Drer_73969.3:0.2077594734)61.2/100:0.0868
903618)99.7/100:0.3751165538)53.4/81:0.1213725816,(EFNB2_MOUSE:
0.1226066268,
(Drer_10432.8:0.0745919865,Drer_79638.5:0.2601173777)97.2/100:0.1574
958517)66.4/96:0.0906218412)97.4/96:0.3024467108,CiEfnB:
0.6174670294)92.9/96:0.2051255982,
((BfEfn1:0.4668190449,BfEfn2:0.8351545441)77.3/99:0.1728131984,
((((((((((Cvar_2778:0.1591610425,
(((Cvar_8977:0.3648028685,Aque_39602:2.0737814073)0/24:0.0005296282,
Cvar_1084:0.2631332282)92/81:0.1486485923,Cvar_12230:0.2904438914)75
.5/80:0.1152477946)98.9/89:0.6587549306,
((Cpro_58774.15:0.7251440333,Lapi_57857:0.3436124146)89.1/100:0.4565
821245,
(Ifas_6298:0.1736454667,Ifas_6301:0.0488270993)99.7/100:1.3879351430
)51/80:0.2801312731)90.4/97:0.5100570251,((m.
393972_Acanthoeca_spectabilis:0.9078667525,m.
338217_Stephanoeca_diplocostata_aus:
1.1860184409)86.9/99:0.5504682676,(m.134676_Salpingoeca_urceolata:
1.1790946517,m.130281_Salpingoeca_infusionum:
1.2349206527)79.7/98:0.3546113246)84.5/97:0.3357805655)61.9/94:0.331
2654687,Mbre_Cu_33299:2.6743575088)0/21:0.0633421319,
(((((((((KFH05385_1_SRS12D_Tgon:
0.5196957947,XP_008888179_1_SRS19A_Hham:
0.7095919939)98.7/100:0.7032982192,(CEL72442_1_SRS16E_Tgon:
1.0933098562,ESS32458_1_SRS40E_Tgon:
0.7844807354)14.1/92:0.0793371192)87.6/99:0.2519753160,
(XP_003880697_1_SRSl_Ncan:1.6837718895,XP_003883724_1_SRSl_Ncan:
1.4098394130)67.2/52:0.1800202536)65.9/39:0.1612050162,
((XP_002364752_1_SRS54_Tgon:0.7990269529,XP_008888917_1_SRS38D_Hham:
0.8000813600)77.8/100:0.2677637095,XP_003883689_1_SRSl_Ncan:

1.4506649115)85.5/99:0.5162975250)0/38:0.0407316195,
((XP_002368205_2_SRS29B_Tgon:0.0000026072,1KZQ_B:
0.0000021420)97.5/100:0.7520590902,(XP_003883788_1_SRSl_Ncan:
0.7044279006,XP_008889467_1_SRS57_Hham:
1.6327997429)92.3/98:0.5880768271)83.1/74:0.2807696878)87.6/94:0.219
4392654,(XP_003879773_1_SRSl_Ncan:1.3946790089,
(XP_003882257_1_SRSl_Ncan:1.1446107127,XP_003885673_1_SRSl_Ncan:
1.4919794017)6.8/87:0.2450210023)80.2/97:0.1769082016)81.9/94:0.2072
188628,(XP_002372001_1_SRS49D_Tgon:1.4451349218,
(XP_003883569_1_SRSl_Ncan:1.7201400005,XP_003884629_1_SAG2D_Ncan:
1.8258554816)35.6/89:0.3055099961)0/64:0.0875128057)79.3/70:0.137240
1639,(((((((KFH12913_1_SRS55A_Tgon:
0.8373313385,XP_018635616_1_SRS55N_Tgon:
0.5199748139)86.2/100:0.3061570454,KYF47445_1_SRS55F_Tgon:
1.0945945181)84.9/80:0.2726808069,(XP_003884898_1_SRSl_Ncan:
1.0638600829,(EPR60006_1_SRS48K_Tgon:
0.9407561990,EPR60025_1_SRS47C_Tgon:
0.7071059047)63.7/100:0.1714925770)49.5/71:0.2892279853)0/76:0.05066
84735,((KYF49211_1_SRS56_Tgon:1.5226410213,
(XP_003881691_1_SRSl_Ncan:0.6289209860,XP_008884156_1_SRS20C_Hham:
0.8426749278)54.3/98:0.2512942112)66/99:0.2744875614,XP_003880400_1_
SRS14_Ncan:
1.1157918297)92.7/97:0.7790832264)72/96:0.2523746363,XP_003884896_1_
SRSl_Ncan:1.5011279683)22.2/97:0.0817184964,CEL77162_1_SRSl_Tgon:
1.1242836979)78.8/98:0.3250035993,XP_003881503_1_SRSl_Ncan:
2.0192358694)87.9/79:0.4536718839,(XP_003881326_1_SRSl_Ncan:
2.0167991403,(XP_003881327_1_SRSl_Ncan:1.6759124399,
(((XP_003883768_1_SRSl_Ncan:1.8409391210,EPR62843_1_SRS26B_Tgon:
0.6594915185)36.4/97:0.1463014494,XP_018635103_1_SRS59K_Tgon:
1.0993709761)89.3/100:0.4154310411,XP_003884879_1_SRSl_Ncan:
1.2872715361)67.2/100:0.2015585589)79.4/97:0.2513972981)74.9/76:0.20
59284506)63.1/74:0.1096263270)98.9/88:0.6312441451,
(((((((((Cele_Cu_86692:0.3734539963,Aspe_Cu_91753:0.3297787483)59.5
/
82:0.0453507734,Aspe_Cu_147913:0.1660023834)98.2/100:1.0680792010,As
pe_Cu_220103:1.6360490479)0/25:0.0960962182,
(((Xtes_Cu_53246:0.1221754696,Xtes_Cu_142493:0.1832370477)94.9/100:0
.6141334438,
(Aspe_Cu_63219:0.0000020608,Spyx_Cu_614696:0.0247099850)99.8/100:1.7
135242290)62.7/93:0.2773732234,Aspe_Cu_437501:1.1696132786)27.6/73:0
.
3237910892)66.8/39:0.3434015310,Cele_Cu_55819:2.0572188589)0/33:0.03
30849988,((Xtes_Cu_27655:1.5893345870,
(Clat_Cu_35282:0.7781804538,Nbij_Cu_650:1.5914092005)81.3/99:0.74165
00647)88.2/96:0.6578238277,
((Spyx_Cu_588060:0.7466668979,1JZG_A_Paer_Azurin:
1.9312551472)0/64:0.0000026995,
(Aspe_Cu_335984:0.3471598700,Spyx_Cu_215319:0.3261532215)98.9/99:0.9
225109208)92.6/98:0.7178127516)52.6/90:0.1566978457)76.5/41:0.191378
8637,
(Cvar_Cu_11918:0.1233938141,Aspe_Cu_4486:0.2476434068)99.9/100:1.268
1899950)85/79:0.3682968707,
(((Xtes_Cu_36302:1.6509790371,Xtes_Cu_6593:1.5227741056)83.5/31:0.65
09764317,

((Aspe_Cu_55573:0.2650104823,Spyx_Cu_676302:0.2777854957)32.2/86:0.0
624580958,Aspe_Cu_16005:0.1641007342)96.2/98:1.2534209260)42.4/13:0.
2544367662,
(((Xtes_Cu_32872:0.0228421347,Xtes_Cu_32871:0.0718101322)76.3/95:0.8
251439287,Hpop_Cu_32908:2.5032281128)59.3/97:0.4953117861,Tfer_Rusti
cyanin:
1.6951984346)18.5/49:0.2587482669)83.6/32:0.4318651892)78.9/20:0.199
4074842,
(((((((((Hpop_Cu_30906:0.2512658582,Xtes_Cu_54960:0.3171551207)69.8
/
99:0.0995439014,Pfic_Cu_52139:0.1337148922)92.8/99:0.3487726431,Xtes
_Cu_102830:0.7585980564)69.5/95:0.1376203617,
(Xtes_Cu_17449:0.8529014160,Xtes_Cu_180680:1.3268125643)19.5/93:0.13
36348844)78/94:0.1758537178,(Hpop_Cu_54677:1.1506098291,
(Xtes_Cu_123795:1.5097559553,
((Aspe_Cu_128312:0.4747836273,Aspe_Cu_94040:0.7904431785)80.5/96:0.3
163197584,Aspe_Cu_182790:0.3016002326)86.7/100:0.5117812088)68.3/86:
0.2492856672)29.5/62:0.0591600279)89.3/78:0.3334635605,
(((Mory_Cu_03946:0.6563768586,Mory_Cu_13261:0.7140628943)80.2/100:0.
2679990836,(Mory_Cu_12247:1.2106841866,
(Mory_Cu_8593:0.1321170871,Ncra_Cu_27669:0.1911567700)66.9/100:0.264
1359606)73.5/80:0.6521304132)85.2/79:0.3434364040,Afum_Cu_1558:1.239
1059441)94.1/97:0.5671067552)0/73:0.0169676286,
(Aspe_Cu_470184:1.2715052088,Hvul_Cu_13222.1:1.4759374694)75.2/93:0.
2963589755)46.8/69:0.2306918384,
(Mver_Cu_01248:1.0554387567,Xtes_Cu_67971:1.3998512515)63.6/87:0.458
6616341)49.5/83:0.1292339116,Pcar_Cu_62093:1.9827889431)0/28:0.00000
28705,
(((Xtes_Cu_40331:0.1654332423,Xtes_Cu_107017:0.1650789504)0/97:0.024
4964333,Xtes_Cu_52934:0.2207229891)99.6/100:1.3842499770,
((Ccan_Cu_153316:1.7204131786,Xtes_Cu_47395:0.9949407586)86.2/100:0.
5999528902,(Aspe_Cu_489355:0.0751410440,
(Aspe_Cu_772:0.4473706461,Aspe_Cu_445213:0.0000026430)99.4/100:2.239
5694680)96.4/96:1.3885345630)46.8/83:0.2890877684)77.7/72:0.38245003
45)82.9/58:0.1929962864)79.9/23:0.1195775450,
((((Xtes_Cu_52242:0.6292033155,
(Spyx_Cu_120943:0.0000010062,Spyx_Cu_120946:0.0000010062)99.3/100:1.
6758929070)90.2/99:0.8362337517,(Aspe_Cu_56717:0.7708863004,
(Aspe_Cu_459874:0.3618200308,Apal_Cu_7673:1.9735833581)21.1/57:0.142
2119841)78.5/63:0.2662383730)82.2/62:0.4409767936,
((Aspe_Cu_276485:1.7809895309,Uper_Plastocyanin:
1.4636429200)0/31:0.0000025834,Mory_Cu_08291:2.1511457959)39.6/52:0.
2321877177)91.7/82:0.6639904441,
((((((Aspe_Cu_136914:0.3524742118,Aspe_Cu_298288:0.4644935070)99.8/1
00:2.2532086540,Cnuc_Cu_19759:1.2267870938)52.2/96:0.3361226028,
(Spyx_Cu_152634:1.3675035708,Atha_Blue_copper:
0.9522328369)90.7/98:0.7206894707)55.1/91:0.3318785397,
(Apar_Cu_52294:0.4910512250,Apar_Cu_154092:1.4652887036)57.3/84:0.26
05822245)27.7/47:0.2353315763,
((Apar_Cu_235353:1.5904991555,Sros_Cu_10601:1.2026914083)60.1/93:0.2
584049757,Pphy_Cu_8558:1.2944527660)80.6/91:0.5055822444)0/66:0.0000
020061,Spyx_Cu_70505:3.2048694756)18.5/19:0.2497385116)84.4/21:0.240
6205202)47.2/82:0.0777388845)36.4/71:0.0909271705,AKQ22481_1_P48_45_
Plasmodium:

2.8833652357)74.7/74:0.1118253888)0/18:0.0112639205,Snux_75319:1.770
8492893)75/86:0.2545770624,
(((((((Atet_141600:0.2233459466,Clat_80728:0.1555803226)91.2/100:0.1
058317188,
((((Aele_124411:0.0000010062,Aele_124437:0.0000010062)96.8/100:0.105
4793046,Nbij_143918:0.2306111770)37.3/99:0.0546676344,Clat_120953:0.
2023675260)100/100:0.2930070927,
(Aele_79976:0.2216294415,Nbij_20083:0.3147791006)82/100:0.0783964711
)35.7/98:0.0584177214)92.9/99:0.1619297588,Clat_48253:0.3748967801)6
4.1/97:0.0644033779,Pphy_19559:0.3493633288)93.9/99:0.2143079385,
(Hvul_1018918.1:0.0000010062,Hvul_1028415.1:0.0000028865)99.9/100:0.
5916825982)87.1/99:0.1608788746,
(Pper_105129:0.4443192920,Pper_103076:0.4457578113)86.5/99:0.2343860
087)85.9/100:0.1309409109,
((Hvul_1028689.1:0.5833077616,Pphy_13023:0.5890381809)73.9/100:0.138
5864002,ALQ43976_1_6cysteine_Plasmodium:
2.2519394059)59.5/89:0.1355736351)95.3/89:0.2640295730)86.7/65:0.133
3000009,
(Ever_19722:1.0800014898,Hvul_1029169.1:1.0173179025)91.4/59:0.30065
38026)0/38:0.0000020445,
(Apal_89985:0.7297109472,Pcar_179493:0.6982585299)45.1/87:0.18320902
98)56.2/47:0.0837557620,
((((((Atet_66572:0.2246852480,Atet_41310:0.1702307250)25.3/89:0.0617
116973,
(Clat_97078:0.0000010062,Clat_97081:0.0000010062)92.4/100:0.10249106
31)93/95:0.1535569864,
(Nbij_52832:0.0000010062,Nbij_52843:0.0000010062)100/100:0.432603597
7)87.9/89:0.1888346192,Pphy_29075:0.4709095169)77.5/91:0.1820853949,
((Holi_5987:0.0483913553,Hvul_1033087.1:0.0878620626)91.8/100:0.1462
071817,Hvir_3164:0.0877742507)100/100:0.6904034749)98.5/99:0.4282479
803,(((((Apal_398190:0.4043990457,((Apal_41158:0.3089285826,
(Apal_344094:0.4879410106,Nvec_EDO44950:0.3022240724)97.3/100:0.2404
543979)57.6/91:0.0476251645,
(Apal_54304:0.5910140110,Nvec_EDO47804:0.4116094272)96.1/91:0.251911
9325)75.2/30:0.0461842675)83.1/34:0.0727335320,
(((Apal_344111:0.7556047193,Pcar_215100:0.4907012098)83.3/95:0.16441
59383,Adig_07747:0.6971515566)84.3/79:0.0839822291,Pcar_235347:0.503
2531458)23.9/13:0.0282996894)97.8/93:0.2271471212,
(Ever_1897:0.9845328994,
(Pcar_189454:0.3004226971,Adig_20275:0.5621371075)87.7/100:0.1688807
156)82.2/27:0.1552767300)91.3/23:0.1074070979,
(Pcar_240721:0.4531605134,
((Adig_10295:0.0385978535,Adig_20773:0.0313453924)99.7/100:0.1862548
515,Adig_19031:0.0000021550)98.5/100:0.3526320758)97.9/99:0.34195595
41)63.6/27:0.0233765269,(Apal_400465:0.4159630225,
((Apal_288439:0.0000010062,Apal_288406:0.0000010062)90.8/100:0.15845
26635,
(Btue_9751:0.3496584589,Hdig_4952:0.1021383679)84.4/98:0.1480815717)
100/100:0.7645453726)83.4/38:0.1352614960)80.6/54:0.0808706780)79.8/
58:0.1328451181)98.2/95:0.3199659768,
(Spur_23757:0.5691696509,Skow_5664:0.6298075998)88.2/97:0.3472541717
)75.1/96:0.1318680991)63.5/95:0.1463440314)27.7/64:0.0519002581,
(Lgig_171062:0.7287381248,
((Hpsi_83841:0.1001414792,Hpsi_83847:0.0508047214)100/100:0.63905916

```
82,
(Tpol_19734:0.0532519749,Tpol_19735:0.0942860280)97.1/100:0.27733775
82)55.6/99:0.0744694060)94.7/100:0.2473286148)68.2/88:0.1919127573,
(Dpul_EFX72793:0.1711969553,Lith_21086:0.0414988080)98.5/100:0.40459
79009)92.3/99:0.2758008324,((CiEfnAa:0.5353343577,(CiEfnAc:
0.1612733621,CiEfnAd:
0.2307606537)99.5/100:0.6051302331)70.3/99:0.2214638383,CiEfnAb:
0.8160437270)89.7/100:0.3652674725)99.1/100:0.5122170619)95.5/100:0.
2127167186,
(Drer_12577.6:0.1069440044,Drer_50216.6:0.1290950561)97.9/100:0.2301
131362)96.3/100:0.2036134548,EFNA1_MOUSE:0.0984841005);
```

Fig5C(Fig_S9_IQtree_tree_file

```
#NEXUS
begin taxa;
	dimensions ntax=221;
	taxlabels
	1JZG_A_Paer_Azurin
	1KZQ_B[&!color=#996600]
	AKQ22481_1_P48_45_Plasmodium[&!color=#996600]
	ALQ43976_1_6cysteine_Plasmodium[&!color=#996600]
	Adig_07747[&!color=#cccc00]
	Adig_10295[&!color=#cccc00]
	Adig_19031[&!color=#cccc00]
	Adig_20275[&!color=#cccc00]
	Adig_20773[&!color=#cccc00]
	Aele_124411[&!color=#cccc00]
	Aele_124437[&!color=#cccc00]
	Aele_79976[&!color=#cccc00]
	Afum_Cu_1558[&!color=#cc00cc]
	Apal_288406[&!color=#cccc00]
	Apal_288439[&!color=#cccc00]
	Apal_344094[&!color=#cccc00]
	Apal_344111[&!color=#cccc00]
	Apal_398190[&!color=#cccc00]
	Apal_400465[&!color=#cccc00]
	Apal_41158[&!color=#cccc00]
	Apal_54304[&!color=#cccc00]
	Apal_89985[&!color=#cccc00]
	Apal_Cu_7673[&!color=#cccc00]
	Apar_Cu_154092[&!color=#cccc00]
	Apar_Cu_235353[&!color=#cccc00]
	Apar_Cu_52294[&!color=#cccc00]
	Aque_39602[&!color=#0033cc]
	Aspe_Cu_128312[&!color=#ffcc00]
	Aspe_Cu_136914[&!color=#ffcc00]
	Aspe_Cu_147913[&!color=#ffcc00]
	Aspe_Cu_16005[&!color=#ffcc00]
	Aspe_Cu_182790[&!color=#ffcc00]
	Aspe_Cu_220103[&!color=#ffcc00]
	Aspe_Cu_276485[&!color=#ffcc00]
	Aspe_Cu_298288[&!color=#ffcc00]
	Aspe_Cu_335984[&!color=#ffcc00]
```

Aspe_Cu_437501[&!color=#ffcc00]
Aspe_Cu_445213[&!color=#ffcc00]
Aspe_Cu_4486[&!color=#ffcc00]
Aspe_Cu_459874[&!color=#ffcc00]
Aspe_Cu_470184[&!color=#ffcc00]
Aspe_Cu_489355[&!color=#ffcc00]
Aspe_Cu_55573[&!color=#ffcc00]
Aspe_Cu_56717[&!color=#ffcc00]
Aspe_Cu_63219[&!color=#ffcc00]
Aspe_Cu_772[&!color=#ffcc00]
Aspe_Cu_91753[&!color=#ffcc00]
Aspe_Cu_94040[&!color=#ffcc00]
Atet_141600[&!color=#cccc00]
Atet_41310[&!color=#cccc00]
Atet_66572[&!color=#cccc00]
Atha_Blue_copper[&!color=#cccc00]
BfEfn1[&!color=#339900]
BfEfn2[&!color=#339900]
Btue_9751[&!color=#cccc00]
CEL72442_1_SRS16E_Tgon[&!color=#996600]
CEL77162_1_SRSl_Tgon[&!color=#996600]
Ccan_Cu_153316[&!color=#0033cc]
Cele_Cu_55819[&!color=#0033cc]
Cele_Cu_86692[&!color=#0033cc]
CiEfnAa[&!color=#339900]
CiEfnAb[&!color=#339900]
CiEfnAc[&!color=#339900]
CiEfnAd[&!color=#339900]
CiEfnB[&!color=#339900]
Clat_120953[&!color=#cccc00]
Clat_48253[&!color=#cccc00]
Clat_80728[&!color=#cccc00]
Clat_97078[&!color=#cccc00]
Clat_97081[&!color=#cccc00]
Clat_Cu_35282[&!color=#cccc00]
Cnuc_Cu_19759[&!color=#0033cc]
'Cpro_58774.15'[&!color=#0033cc]
Cvar_1084[&!color=#0033cc]
Cvar_12230[&!color=#0033cc]
Cvar_2778[&!color=#0033cc]
Cvar_8977[&!color=#0033cc]
Cvar_Cu_11918[&!color=#0033cc]
Dpul_EFX72793[&!color=#339900]
'Drer_10432.8'[&!color=#990033]
'Drer_111146.1'[&!color=#990033]
'Drer_116412.1'[&!color=#990033]
'Drer_12577.6'[&!color=#990033]
'Drer_24428.4'[&!color=#990033]
'Drer_50216.6'[&!color=#990033]
'Drer_62002.6'[&!color=#990033]
'Drer_73969.3'[&!color=#990033]
'Drer_79638.5'[&!color=#990033]
EFNA1_HUMAN_3CZU_B[&!color=#990033]
EFNA1_MOUSE[&!color=#990033]

EFNA2_HUMAN[&!color=#990033]
EFNA2_MOUSE[&!color=#990033]
EFNA3_HUMAN[&!color=#990033]
EFNA4_HUMAN[&!color=#990033]
EFNA4_MOUSE[&!color=#990033]
EFNA5_HUMAN[&!color=#990033]
EFNB1_HUMAN[&!color=#990033]
EFNB2_MOUSE[&!color=#990033]
EFNB3_HUMAN[&!color=#990033]
EPR60006_1_SRS48K_Tgon[&!color=#996600]
EPR60025_1_SRS47C_Tgon[&!color=#996600]
EPR62843_1_SRS26B_Tgon[&!color=#996600]
ESS32458_1_SRS40E_Tgon[&!color=#996600]
Ever_1897[&!color=#cccc00]
Ever_19722[&!color=#cccc00]
Hdig_4952[&!color=#cccc00]
Holi_5987[&!color=#cccc00]
Hpop_Cu_30906[&!color=#0033cc]
Hpop_Cu_32908[&!color=#0033cc]
Hpop_Cu_54677[&!color=#0033cc]
Hpsi_83841[&!color=#339900]
Hpsi_83847[&!color=#339900]
Hvir_3164[&!color=#cccc00]
'Hvul_1018918.1'[&!color=#cccc00]
'Hvul_1028415.1'[&!color=#cccc00]
'Hvul_1028689.1'[&!color=#cccc00]
'Hvul_1029169.1'[&!color=#cccc00]
'Hvul_1033087.1'[&!color=#cccc00]
'Hvul_Cu_13222.1'[&!color=#cccc00]
Ifas_6298[&!color=#0033cc]
Ifas_6301[&!color=#0033cc]
KFH05385_1_SRS12D_Tgon[&!color=#996600]
KFH12913_1_SRS55A_Tgon[&!color=#996600]
KYF47445_1_SRS55F_Tgon[&!color=#996600]
KYF49211_1_SRS56_Tgon[&!color=#996600]
Lapi_57857[&!color=#0033cc]
Lgig_171062[&!color=#339900]
Lith_21086[&!color=#339900]
Mbre_Cu_33299[&!color=#ff9900]
Mory_Cu_03946[&!color=#cc00cc]
Mory_Cu_08291[&!color=#cc00cc]
Mory_Cu_12247[&!color=#cc00cc]
Mory_Cu_13261[&!color=#cc00cc]
Mory_Cu_8593[&!color=#cc00cc]
Mver_Cu_01248[&!color=#cc00cc]
Nbij_143918[&!color=#cccc00]
Nbij_20083[&!color=#cccc00]
Nbij_52832[&!color=#cccc00]
Nbij_52843[&!color=#cccc00]
Nbij_Cu_650[&!color=#cccc00]
Ncra_Cu_27669[&!color=#cc00cc]
Nvec_EDO44950[&!color=#cccc00]
Nvec_EDO47804[&!color=#cccc00]
Pcar_179493[&!color=#cccc00]

Pcar_189454[&!color=#cccc00]
Pcar_215100[&!color=#cccc00]
Pcar_235347[&!color=#cccc00]
Pcar_240721[&!color=#cccc00]
Pcar_Cu_62093[&!color=#cccc00]
Pfic_Cu_52139[&!color=#0033cc]
Pper_103076[&!color=#cccc00]
Pper_105129[&!color=#cccc00]
Pphy_13023[&!color=#cccc00]
Pphy_19559[&!color=#cccc00]
Pphy_29075[&!color=#cccc00]
Pphy_Cu_8558[&!color=#cccc00]
Skow_5664[&!color=#339900]
Snux_75319[&!color=#0033cc]
Spur_23757[&!color=#339900]
Spyx_Cu_120943[&!color=#ffcc00]
Spyx_Cu_120946[&!color=#ffcc00]
Spyx_Cu_152634[&!color=#ffcc00]
Spyx_Cu_215319[&!color=#ffcc00]
Spyx_Cu_588060[&!color=#ffcc00]
Spyx_Cu_614696[&!color=#ffcc00]
Spyx_Cu_676302[&!color=#ffcc00]
Spyx_Cu_70505[&!color=#ffcc00]
Sros_Cu_10601[&!color=#cccc00]
Tfer_Rusticyanin
Tpol_19734[&!color=#339900]
Tpol_19735[&!color=#339900]
Uper_Plastocyanin
XP_002364752_1_SRS54_Tgon[&!color=#996600]
XP_002368205_2_SRS29B_Tgon[&!color=#996600]
XP_002372001_1_SRS49D_Tgon[&!color=#996600]
XP_003879773_1_SRSl_Ncan[&!color=#996600]
XP_003880400_1_SRS14_Ncan[&!color=#996600]
XP_003880697_1_SRSl_Ncan[&!color=#996600]
XP_003881326_1_SRSl_Ncan[&!color=#996600]
XP_003881327_1_SRSl_Ncan[&!color=#996600]
XP_003881503_1_SRSl_Ncan[&!color=#996600]
XP_003881691_1_SRSl_Ncan[&!color=#996600]
XP_003882257_1_SRSl_Ncan[&!color=#996600]
XP_003883569_1_SRSl_Ncan[&!color=#996600]
XP_003883689_1_SRSl_Ncan[&!color=#996600]
XP_003883724_1_SRSl_Ncan[&!color=#996600]
XP_003883768_1_SRSl_Ncan[&!color=#996600]
XP_003883788_1_SRSl_Ncan[&!color=#996600]
XP_003884629_1_SAG2D_Ncan[&!color=#996600]
XP_003884879_1_SRSl_Ncan[&!color=#996600]
XP_003884896_1_SRSl_Ncan[&!color=#996600]
XP_003884898_1_SRSl_Ncan[&!color=#996600]
XP_003885673_1_SRSl_Ncan[&!color=#996600]
XP_008884156_1_SRS20C_Hham[&!color=#996600]
XP_008888179_1_SRS19A_Hham[&!color=#996600]
XP_008888917_1_SRS38D_Hham[&!color=#996600]
XP_008889467_1_SRS57_Hham[&!color=#996600]
XP_018635103_1_SRS59K_Tgon[&!color=#996600]

```
		XP_018635616_1_SRS55N_Tgon[&!color=#996600]
		Xtes_Cu_102830[&!color=#0033cc]
		Xtes_Cu_107017[&!color=#0033cc]
		Xtes_Cu_123795[&!color=#0033cc]
		Xtes_Cu_142493[&!color=#0033cc]
		Xtes_Cu_17449[&!color=#0033cc]
		Xtes_Cu_180680[&!color=#0033cc]
		Xtes_Cu_27655[&!color=#0033cc]
		Xtes_Cu_32871[&!color=#0033cc]
		Xtes_Cu_32872[&!color=#0033cc]
		Xtes_Cu_36302[&!color=#0033cc]
		Xtes_Cu_40331[&!color=#0033cc]
		Xtes_Cu_47395[&!color=#0033cc]
		Xtes_Cu_52242[&!color=#0033cc]
		Xtes_Cu_52934[&!color=#0033cc]
		Xtes_Cu_53246[&!color=#0033cc]
		Xtes_Cu_54960[&!color=#0033cc]
		Xtes_Cu_6593[&!color=#0033cc]
		Xtes_Cu_67971[&!color=#0033cc]
		'm.130281_Salpingoeca_infusionum'[&!color=#ff9900]
		'm.134676_Salpingoeca_urceolata'[&!color=#ff9900]
		'm.338217_Stephanoeca_diplocostata_aus'[&!color=#ff9900]
		'm.393972_Acanthoeca_spectabilis'[&!color=#ff9900]
;
end;

begin trees;
		tree tree_1 = [&R] (EFNA1_HUMAN_3CZU_B[&!color=#990033]:
0.083253,(((((EFNA2_HUMAN[&!color=#990033]:0.041617,EFNA2_MOUSE[&!
color=#990033]:0.046609)[&label=100,!color=#990033]:0.199543,
(EFNA5_HUMAN[&!color=#990033]:0.087094,('Drer_111146.1'[&!
color=#990033]:3.0E-6,'Drer_116412.1'[&!color=#990033]:0.119645)
[&label=100,!color=#990033]:0.090267)[&label=100,!color=#990033]:
0.134257)[&label=100,!color=#990033]:0.225096,(EFNA3_HUMAN[&!
color=#990033]:0.294725,(EFNA4_HUMAN[&!color=#990033]:
0.080367,EFNA4_MOUSE[&!color=#990033]:0.060433)[&label=100,!
color=#990033]:0.43414)[&label=98,!color=#990033]:0.119141)
[&label=99,!color=#990033]:0.06886,(((((((EFNB1_HUMAN[&!
color=#990033]:0.084464,'Drer_24428.4'[&!color=#990033]:0.182227)
[&label=100,!color=#990033]:0.091012,(EFNB3_HUMAN[&!color=#990033]:
0.160792,('Drer_62002.6'[&!color=#990033]:0.127901,'Drer_73969.3'[&!
color=#990033]:0.207968)[&label=100,!color=#990033]:0.086656)
[&label=100,!color=#990033]:0.37622)[&label=81,!color=#990033]:
0.121285,(EFNB2_MOUSE[&!color=#990033]:0.122523,('Drer_10432.8'[&!
color=#990033]:0.075209,'Drer_79638.5'[&!color=#990033]:0.260125)
[&label=100,!color=#990033]:0.157403)[&label=96,!color=#990033]:
0.091832)[&label=96,!color=#990033]:0.30076,CiEfnB[&!color=#339900]:
0.617525)[&label=96,!color=#339900]:0.209742,((BfEfn1[&!
color=#339900]:0.464253,BfEfn2[&!color=#339900]:0.835564)
[&label=99,!color=#339900]:0.180267,((((((((Cvar_2778[&!
color=#0033cc]:0.163091,((Cvar_8977[&!color=#0033cc]:0.365965,
(Cvar_1084[&!color=#0033cc]:0.260239,Aque_39602[&!color=#0033cc]:
2.076422)[&label=54,!color=#0033cc]:0.003046)[&label=81,!
color=#0033cc]:0.148291,Cvar_12230[&!color=#0033cc]:0.290824)
```

[&label=80,!color=#0033cc]:0.111909)[&label=89,!color=#0033cc]:
0.667872,(('Cpro_58774.15'[&!color=#0033cc]:0.723849,Lapi_57857[&!
color=#0033cc]:0.346781)[&label=100,!color=#0033cc]:0.457286,
(Ifas_6298[&!color=#0033cc]:0.174944,Ifas_6301[&!color=#0033cc]:
0.047782)[&label=100,!color=#0033cc]:1.384108)[&label=80,!
color=#0033cc]:0.270279)[&label=97,!color=#0033cc]:0.50961,(('m.
393972_Acanthoeca_spectabilis'[&!color=#ff9900]:0.901654,'m.
338217_Stephanoeca_diplocostata_aus'[&!color=#ff9900]:1.192361)
[&label=99,!color=#ff9900]:0.554503,('m.
134676_Salpingoeca_urceolata'[&!color=#ff9900]:1.186283,'m.
130281_Salpingoeca_infusionum'[&!color=#ff9900]:1.227883)
[&label=98,!color=#ff9900]:0.359252)[&label=97,!color=#ff9900]:
0.33731)[&label=94]:0.379877,((Snux_75319[&!color=#0033cc]:
1.745467,Mbre_Cu_33299[&!color=#ff9900]:2.717045)[&label=30]:3.0E-6,
(((((((((KFH05385_1_SRS12D_Tgon[&!color=#996600]:
0.522787,XP_008888179_1_SRS19A_Hham[&!color=#996600]:0.708454)
[&label=100,!color=#996600]:0.703612,(CEL72442_1_SRS16E_Tgon[&!
color=#996600]:1.093307,ESS32458_1_SRS40E_Tgon[&!color=#996600]:
0.786683)[&label=92,!color=#996600]:0.078727)[&label=99,!
color=#996600]:0.254144,(XP_003880697_1_SRSl_Ncan[&!color=#996600]:
1.689334,XP_003883724_1_SRSl_Ncan[&!color=#996600]:1.405939)
[&label=52,!color=#996600]:0.179537)[&label=39,!color=#996600]:
0.158818,((XP_002364752_1_SRS54_Tgon[&!color=#996600]:
0.796434,XP_008888917_1_SRS38D_Hham[&!color=#996600]:0.803123)
[&label=100,!color=#996600]:0.269826,XP_003883689_1_SRSl_Ncan[&!
color=#996600]:1.448674)[&label=99,!color=#996600]:0.521732)
[&label=38,!color=#996600]:0.047144,((XP_002368205_2_SRS29B_Tgon[&!
color=#996600]:3.0E-6,1KZQ_B[&!color=#996600]:3.0E-6)[&label=100,!
color=#996600]:0.753388,(XP_003883788_1_SRSl_Ncan[&!color=#996600]:
0.705535,XP_008889467_1_SRS57_Hham[&!color=#996600]:1.638441)
[&label=98,!color=#996600]:0.58861)[&label=74,!color=#996600]:
0.271825)[&label=94,!color=#996600]:0.219471,
(XP_003879773_1_SRSl_Ncan[&!color=#996600]:1.395216,
(XP_003882257_1_SRSl_Ncan[&!color=#996600]:
1.149873,XP_003885673_1_SRSl_Ncan[&!color=#996600]:1.494801)
[&label=87,!color=#996600]:0.24845)[&label=97,!color=#996600]:
0.17912)[&label=94,!color=#996600]:0.211002,
(XP_002372001_1_SRS49D_Tgon[&!color=#996600]:1.448333,
(XP_003883569_1_SRSl_Ncan[&!color=#996600]:
1.71088,XP_003884629_1_SAG2D_Ncan[&!color=#996600]:1.832957)
[&label=89,!color=#996600]:0.299188)[&label=64,!color=#996600]:
0.084402)[&label=70,!color=#996600]:0.138518,
(((((((KFH12913_1_SRS55A_Tgon[&!color=#996600]:
0.837972,XP_018635616_1_SRS55N_Tgon[&!color=#996600]:0.523544)
[&label=100,!color=#996600]:0.306653,KYF47445_1_SRS55F_Tgon[&!
color=#996600]:1.095095)[&label=80,!color=#996600]:0.272609,
(XP_003884898_1_SRSl_Ncan[&!color=#996600]:1.069641,
(EPR60006_1_SRS48K_Tgon[&!color=#996600]:
0.940352,EPR60025_1_SRS47C_Tgon[&!color=#996600]:0.711303)
[&label=100,!color=#996600]:0.169878)[&label=71,!color=#996600]:
0.289774)[&label=76,!color=#996600]:0.052414,
((KYF49211_1_SRS56_Tgon[&!color=#996600]:1.521454,
(XP_003881691_1_SRSl_Ncan[&!color=#996600]:
0.629768,XP_008884156_1_SRS20C_Hham[&!color=#996600]:0.843129)

[&label=98,!color=#996600]:0.253607)[&label=99,!color=#996600]:
0.274587,XP_003880400_1_SRS14_Ncan[&!color=#996600]:1.114329)
[&label=97,!color=#996600]:0.782214)[&label=96,!color=#996600]:
0.255993,XP_003884896_1_SRSl_Ncan[&!color=#996600]:1.493291)
[&label=97,!color=#996600]:0.080654,CEL77162_1_SRSl_Tgon[&!
color=#996600]:1.124338)[&label=98,!color=#996600]:
0.325772,XP_003881503_1_SRSl_Ncan[&!color=#996600]:2.0235)
[&label=79,!color=#996600]:0.461541,(XP_003881326_1_SRSl_Ncan[&!
color=#996600]:2.009568,(XP_003881327_1_SRSl_Ncan[&!color=#996600]:
1.684377,(((XP_003883768_1_SRSl_Ncan[&!color=#996600]:
1.854208,EPR62843_1_SRS26B_Tgon[&!color=#996600]:0.658536)
[&label=97,!color=#996600]:0.133103,XP_018635103_1_SRS59K_Tgon[&!
color=#996600]:1.10883)[&label=100,!color=#996600]:
0.418605,XP_003884879_1_SRSl_Ncan[&!color=#996600]:1.295273)
[&label=100,!color=#996600]:0.193857)[&label=97,!color=#996600]:
0.260723)[&label=76,!color=#996600]:0.202659)[&label=74,!
color=#996600]:0.103932)[&label=88,!color=#996600]:0.622886,
(((((((((Cele_Cu_86692[&!color=#0033cc]:0.373269,Aspe_Cu_91753[&!
color=#ffcc00]:0.329733)[&label=82,!color=#ffcc00]:
0.052374,Aspe_Cu_147913[&!color=#ffcc00]:0.160077)[&label=100,!
color=#ffcc00]:1.134962,(((Xtes_Cu_53246[&!color=#0033cc]:
0.123332,Xtes_Cu_142493[&!color=#0033cc]:0.182365)[&label=100,!
color=#0033cc]:0.610334,(Aspe_Cu_63219[&!color=#ffcc00]:
2.0E-6,Spyx_Cu_614696[&!color=#ffcc00]:0.024748)[&label=100,!
color=#ffcc00]:1.720483)[&label=93,!color=#ffcc00]:
0.279934,Aspe_Cu_437501[&!color=#ffcc00]:1.158248)[&label=73,!
color=#ffcc00]:0.328586)[&label=56,!color=#ffcc00]:
0.043144,Aspe_Cu_220103[&!color=#ffcc00]:1.673858)[&label=39,!
color=#ffcc00]:0.334287,Cele_Cu_55819[&!color=#0033cc]:2.073238)
[&label=33,!color=#0033cc]:0.026951,((Xtes_Cu_27655[&!
color=#0033cc]:1.560255,(Clat_Cu_35282[&!color=#cccc00]:
0.790564,Nbij_Cu_650[&!color=#cccc00]:1.585889)[&label=99,!
color=#cccc00]:0.757017)[&label=96,!color=#0033cc]:0.667381,
((Spyx_Cu_588060[&!color=#ffcc00]:0.754707,1JZG_A_Paer_Azurin[&!
color=#ffcc00]:1.925327)[&label=64,!color=#ffcc00]:3.0E-6,
(Aspe_Cu_335984[&!color=#ffcc00]:0.34653,Spyx_Cu_215319[&!
color=#ffcc00]:0.328308)[&label=99,!color=#ffcc00]:0.927516)
[&label=98,!color=#0033cc]:0.718376)[&label=90,!color=#0033cc]:
0.14255)[&label=41,!color=#0033cc]:0.18989,(Cvar_Cu_11918[&!
color=#0033cc]:0.123929,Aspe_Cu_4486[&!color=#ffcc00]:0.247437)
[&label=100,!color=#0033cc]:1.261026)[&label=79,!color=#0033cc]:
0.407443,((((Xtes_Cu_36302[&!color=#0033cc]:
1.279918,Spyx_Cu_70505[&!color=#ffcc00]:2.254004)[&label=66,!
color=#0033cc]:0.429642,Xtes_Cu_6593[&!color=#0033cc]:1.550634)
[&label=65,!color=#0033cc]:0.617446,((Aspe_Cu_55573[&!
color=#ffcc00]:0.265366,Spyx_Cu_676302[&!color=#ffcc00]:0.278038)
[&label=86,!color=#ffcc00]:0.058349,Aspe_Cu_16005[&!color=#ffcc00]:
0.168249)[&label=98,!color=#ffcc00]:1.309195)[&label=20,!
color=#0033cc]:0.264231,(((Xtes_Cu_32872[&!color=#0033cc]:
0.02636,Xtes_Cu_32871[&!color=#0033cc]:0.068488)[&label=95,!
color=#0033cc]:0.851784,Hpop_Cu_32908[&!color=#0033cc]:2.466624)
[&label=97,!color=#0033cc]:0.45068,Tfer_Rusticyanin:1.704966)
[&label=49,!color=#0033cc]:0.29291)[&label=59,!color=#0033cc]:
0.422723)[&label=50,!color=#0033cc]:0.176712,

(((((((((Hpop_Cu_30906[&!color=#0033cc]:0.251625,Xtes_Cu_54960[&!
color=#0033cc]:0.317428)[&label=99,!color=#0033cc]:
0.099794,Pfic_Cu_52139[&!color=#0033cc]:0.133752)[&label=99,!
color=#0033cc]:0.348608,Xtes_Cu_102830[&!color=#0033cc]:0.759233)
[&label=95,!color=#0033cc]:0.137702,(Xtes_Cu_17449[&!color=#0033cc]:
0.85422,Xtes_Cu_180680[&!color=#0033cc]:1.327252)[&label=93,!
color=#0033cc]:0.136083)[&label=94,!color=#0033cc]:0.17648,
(Hpop_Cu_54677[&!color=#0033cc]:1.149122,(Xtes_Cu_123795[&!
color=#0033cc]:1.512851,((Aspe_Cu_128312[&!color=#ffcc00]:
0.477459,Aspe_Cu_94040[&!color=#ffcc00]:0.790846)[&label=96,!
color=#ffcc00]:0.313943,Aspe_Cu_182790[&!color=#ffcc00]:0.304446)
[&label=100,!color=#ffcc00]:0.514929)[&label=86,!color=#0033cc]:
0.249462)[&label=62,!color=#0033cc]:0.059079)[&label=78,!
color=#0033cc]:0.337101,(((Mory_Cu_03946[&!color=#cc00cc]:
0.65581,Mory_Cu_13261[&!color=#cc00cc]:0.716135)[&label=100,!
color=#cc00cc]:0.269123,(Mory_Cu_12247[&!color=#cc00cc]:1.215437,
(Mory_Cu_8593[&!color=#cc00cc]:0.132711,Ncra_Cu_27669[&!
color=#cc00cc]:0.190926)[&label=100,!color=#cc00cc]:0.262295)
[&label=80,!color=#cc00cc]:0.654243)[&label=79,!color=#cc00cc]:
0.351628,Afum_Cu_1558[&!color=#cc00cc]:1.23072)[&label=97,!
color=#cc00cc]:0.565203)[&label=73]:0.004166,(Aspe_Cu_470184[&!
color=#ffcc00]:1.278223,'Hvul_Cu_13222.1'[&!color=#cccc00]:1.480768)
[&label=93]:0.30886)[&label=69]:0.240014,(Mver_Cu_01248[&!
color=#cc00cc]:1.049115,Xtes_Cu_67971[&!color=#0033cc]:1.395261)
[&label=87]:0.453774)[&label=83]:0.126788,Pcar_Cu_62093[&!
color=#cccc00]:2.00246)[&label=28,!color=#0033cc]:2.0E-6,
(((Xtes_Cu_40331[&!color=#0033cc]:0.165629,Xtes_Cu_107017[&!
color=#0033cc]:0.165705)[&label=97,!color=#0033cc]:
0.026586,Xtes_Cu_52934[&!color=#0033cc]:0.219461)[&label=100,!
color=#0033cc]:1.387297,((Ccan_Cu_153316[&!color=#0033cc]:
1.724332,Xtes_Cu_47395[&!color=#0033cc]:0.994932)[&label=100,!
color=#0033cc]:0.594715,(Aspe_Cu_489355[&!color=#ffcc00]:0.072564,
(Aspe_Cu_772[&!color=#ffcc00]:0.447692,Aspe_Cu_445213[&!
color=#ffcc00]:2.0E-6)[&label=100,!color=#ffcc00]:2.246609)
[&label=96,!color=#ffcc00]:1.402766)[&label=83,!color=#0033cc]:
0.298281)[&label=72,!color=#0033cc]:0.38143)[&label=58,!
color=#0033cc]:0.199652)[&label=53]:0.108766,((((Xtes_Cu_52242[&!
color=#0033cc]:0.631516,(Spyx_Cu_120943[&!color=#ffcc00]:
3.0E-6,Spyx_Cu_120946[&!color=#ffcc00]:3.0E-6)[&label=100,!
color=#ffcc00]:1.675303)[&label=99,!color=#ffcc00]:0.831154,
(Aspe_Cu_56717[&!color=#ffcc00]:0.771691,(Aspe_Cu_459874[&!
color=#ffcc00]:0.373395,Apal_Cu_7673[&!color=#cccc00]:1.953999)
[&label=57,!color=#ffcc00]:0.136222)[&label=63,!color=#ffcc00]:
0.271827)[&label=62,!color=#ffcc00]:0.403924,((Aspe_Cu_276485[&!
color=#ffcc00]:1.781915,Uper_Plastocyanin:1.450276)[&label=31]:
3.0E-6,Mory_Cu_08291[&!color=#cc00cc]:2.180644)[&label=52,!
color=#ffcc00]:0.256877)[&label=82,!color=#ffcc00]:0.663182,
(((((Aspe_Cu_136914[&!color=#ffcc00]:0.350229,Aspe_Cu_298288[&!
color=#ffcc00]:0.468751)[&label=100,!color=#ffcc00]:
2.196745,Cnuc_Cu_19759[&!color=#0033cc]:1.251777)[&label=96,!
color=#ffcc00]:0.336051,(Spyx_Cu_152634[&!color=#ffcc00]:
1.356989,Atha_Blue_copper[&!color=#cccc00]:0.975261)[&label=98,!
color=#ffcc00]:0.712552)[&label=91,!color=#ffcc00]:0.302035,
(Apar_Cu_52294[&!color=#cccc00]:0.509684,Apar_Cu_154092[&!

color=#cccc00]:1.435872)[&label=84,!color=#cccc00]:0.287881)
[&label=47,!color=#cccc00]:0.199467,((Apar_Cu_235353[&!
color=#cccc00]:1.572096,Sros_Cu_10601[&!color=#cccc00]:1.188082)
[&label=93,!color=#cccc00]:0.208364,Pphy_Cu_8558[&!color=#cccc00]:
1.328407)[&label=91,!color=#cccc00]:0.566476)[&label=66,!
color=#cccc00]:0.242976)[&label=58]:0.24831)[&label=82,!
color=#996600]:0.095234)[&label=71,!color=#996600]:
0.059237,AKQ22481_1_P48_45_Plasmodium[&!color=#996600]:2.906704)
[&label=74]:0.104775)[&label=60]:0.035659)[&label=86,!
color=#cccc00]:0.252872,(((((((Atet_141600[&!color=#cccc00]:
0.223715,Clat_80728[&!color=#cccc00]:0.15562)[&label=100,!
color=#cccc00]:0.105742,((((Aele_124411[&!color=#cccc00]:
3.0E-6,Aele_124437[&!color=#cccc00]:3.0E-6)[&label=100,!
color=#cccc00]:0.105638,Nbij_143918[&!color=#cccc00]:0.230877)
[&label=99,!color=#cccc00]:0.054683,Clat_120953[&!color=#cccc00]:
0.20269)[&label=100,!color=#cccc00]:0.293193,(Aele_79976[&!
color=#cccc00]:0.221844,Nbij_20083[&!color=#cccc00]:0.31484)
[&label=100,!color=#cccc00]:0.078222)[&label=98,!color=#cccc00]:
0.058977)[&label=99,!color=#cccc00]:0.161931,Clat_48253[&!
color=#cccc00]:0.375024)[&label=97,!color=#cccc00]:
0.06345,Pphy_19559[&!color=#cccc00]:0.351151)[&label=99,!
color=#cccc00]:0.214351,('Hvul_1018918.1'[&!color=#cccc00]:
3.0E-6,'Hvul_1028415.1'[&!color=#cccc00]:3.0E-6)[&label=100,!
color=#cccc00]:0.592849)[&label=99,!color=#cccc00]:0.161654,
(Pper_105129[&!color=#cccc00]:0.446786,Pper_103076[&!color=#cccc00]:
0.444622)[&label=99,!color=#cccc00]:0.234615)[&label=100,!
color=#cccc00]:0.130472,(('Hvul_1028689.1'[&!color=#cccc00]:
0.590123,Pphy_13023[&!color=#cccc00]:0.58392)[&label=100,!
color=#cccc00]:0.137678,ALQ43976_1_6cysteine_Plasmodium[&!
color=#996600]:2.253107)[&label=89,!color=#cccc00]:0.137323)
[&label=89,!color=#cccc00]:0.268835)[&label=65,!color=#cccc00]:
0.137729,(Ever_19722[&!color=#cccc00]:1.058544,'Hvul_1029169.1'[&!
color=#cccc00]:1.029235)[&label=59,!color=#cccc00]:0.296931)
[&label=38,!color=#cccc00]:2.0E-6,(Apal_89985[&!color=#cccc00]:
0.727368,Pcar_179493[&!color=#cccc00]:0.698387)[&label=87,!
color=#cccc00]:0.18588)[&label=47,!color=#cccc00]:0.075417,
((((((Atet_66572[&!color=#cccc00]:0.22489,Atet_41310[&!
color=#cccc00]:0.170402)[&label=89,!color=#cccc00]:0.064016,
(Clat_97078[&!color=#cccc00]:3.0E-6,Clat_97081[&!color=#cccc00]:
3.0E-6)[&label=100,!color=#cccc00]:0.100929)[&label=95,!
color=#cccc00]:0.159745,(Nbij_52832[&!color=#cccc00]:
3.0E-6,Nbij_52843[&!color=#cccc00]:3.0E-6)[&label=100,!
color=#cccc00]:0.427479)[&label=89,!color=#cccc00]:
0.194983,Pphy_29075[&!color=#cccc00]:0.467487)[&label=91,!
color=#cccc00]:0.163336,((Holi_5987[&!color=#cccc00]:
0.048453,'Hvul_1033087.1'[&!color=#cccc00]:0.088064)[&label=100,!
color=#cccc00]:0.145472,Hvir_3164[&!color=#cccc00]:0.089046)
[&label=100,!color=#cccc00]:0.703028)[&label=99,!color=#cccc00]:
0.430578,((((((Apal_398190[&!color=#cccc00]:0.386897,(Apal_41158[&!
color=#cccc00]:0.278529,(Apal_344094[&!color=#cccc00]:
0.478211,Nvec_EDO44950[&!color=#cccc00]:0.319543)[&label=100,!
color=#cccc00]:0.255989)[&label=91,!color=#cccc00]:0.08263)
[&label=57,!color=#cccc00]:0.061687,Pcar_235347[&!color=#cccc00]:
0.519463)[&label=58,!color=#cccc00]:0.090622,(Apal_54304[&!

```
color=#cccc00]:0.543878,Nvec_ED047804[&!color=#cccc00]:0.448309)
[&label=91,!color=#cccc00]:0.213985)[&label=59,!color=#cccc00]:
0.038811,((Apal_344111[&!color=#cccc00]:0.726668,Pcar_215100[&!
color=#cccc00]:0.509264)[&label=95,!color=#cccc00]:
0.12582,Adig_07747[&!color=#cccc00]:0.769632)[&label=79,!
color=#cccc00]:0.103754)[&label=93,!color=#cccc00]:0.170403,
(Pcar_189454[&!color=#cccc00]:0.332884,Adig_20275[&!color=#cccc00]:
0.536095)[&label=100,!color=#cccc00]:0.324056)[&label=68,!
color=#cccc00]:0.101355,(Apal_400465[&!color=#cccc00]:0.509231,
(Pcar_240721[&!color=#cccc00]:0.474582,((Adig_10295[&!
color=#cccc00]:0.038324,Adig_20773[&!color=#cccc00]:0.031564)
[&label=100,!color=#cccc00]:0.187707,Adig_19031[&!color=#cccc00]:
2.0E-6)[&label=100,!color=#cccc00]:0.331196)[&label=99,!
color=#cccc00]:0.347946)[&label=23,!color=#cccc00]:0.035321)
[&label=48,!color=#cccc00]:0.06564,(((Apal_288439[&!color=#cccc00]:
3.0E-6,Apal_288406[&!color=#cccc00]:3.0E-6)[&label=100,!
color=#cccc00]:0.212931,(Btue_9751[&!color=#cccc00]:
0.352912,Hdig_4952[&!color=#cccc00]:0.096699)[&label=98,!
color=#cccc00]:0.101995)[&label=100,!color=#cccc00]:
0.630271,Ever_1897[&!color=#cccc00]:0.884489)[&label=22,!
color=#cccc00]:0.21893)[&label=54,!color=#cccc00]:0.054642)
[&label=58,!color=#cccc00]:0.135397)[&label=95,!color=#339900]:
0.320496,(Spur_23757[&!color=#339900]:0.563875,Skow_5664[&!
color=#339900]:0.630337)[&label=97,!color=#339900]:0.345087)
[&label=96,!color=#339900]:0.124749)[&label=95,!color=#339900]:
0.149961)[&label=64,!color=#339900]:0.046126,(Lgig_171062[&!
color=#339900]:0.732384,((Hpsi_83841[&!color=#339900]:
0.100288,Hpsi_83847[&!color=#339900]:0.05075)[&label=100,!
color=#339900]:0.639266,(Tpol_19734[&!color=#339900]:
0.053377,Tpol_19735[&!color=#339900]:0.094343)[&label=100,!
color=#339900]:0.276686)[&label=99,!color=#339900]:0.074077)
[&label=100,!color=#339900]:0.247837)[&label=88,!color=#339900]:
0.192044,(Dpul_EFX72793[&!color=#339900]:0.171527,Lith_21086[&!
color=#339900]:0.04151)[&label=100,!color=#339900]:0.403905)
[&label=99,!color=#339900]:0.273443,((CiEfnAa[&!color=#339900]:
0.53522,(CiEfnAc[&!color=#339900]:0.161309,CiEfnAd[&!color=#339900]:
0.231174)[&label=100,!color=#339900]:0.607604)[&label=99,!
color=#339900]:0.22143,CiEfnAb[&!color=#339900]:0.817398)
[&label=100,!color=#339900]:0.367367)[&label=100,!color=#990033]:
0.510523)[&label=100,!color=#990033]:0.212717,('Drer_12577.6'[&!
color=#990033]:0.106835,'Drer_50216.6'[&!color=#990033]:0.129652)
[&label=100,!color=#990033]:0.230627)[&label=100,!color=#990033]:
0.203958,EFNA1_MOUSE[&!color=#990033]:0.098754);
end;

begin figtree;
	set appearance.backgroundColorAttribute="Default";
	set appearance.backgroundColour=#ffffff;
	set appearance.branchColorAttribute="User selection";
	set appearance.branchColorGradient=false;
	set appearance.branchLineWidth=1.0;
	set appearance.branchMinLineWidth=0.0;
	set appearance.branchWidthAttribute="Fixed";
	set appearance.foregroundColour=#000000;
```

```
set appearance.hilightingGradient=false;
set appearance.selectionColour=#2d3680;
set branchLabels.colorAttribute="User selection";
set branchLabels.displayAttribute="Branch times";
set branchLabels.fontName="sansserif";
set branchLabels.fontSize=8;
set branchLabels.fontStyle=0;
set branchLabels.isShown=false;
set branchLabels.significantDigits=4;
set layout.expansion=366;
set layout.layoutType="RECTILINEAR";
set layout.zoom=0;
set legend.attribute="label";
set legend.fontSize=10.0;
set legend.isShown=false;
set legend.significantDigits=4;
set nodeBars.barWidth=4.0;
set nodeBars.displayAttribute=null;
set nodeBars.isShown=false;
set nodeLabels.colorAttribute="User selection";
set nodeLabels.displayAttribute="label";
set nodeLabels.fontName="sansserif";
set nodeLabels.fontSize=8;
set nodeLabels.fontStyle=0;
set nodeLabels.isShown=true;
set nodeLabels.significantDigits=4;
set nodeShapeExternal.colourAttribute="User selection";
set nodeShapeExternal.isShown=false;
set nodeShapeExternal.minSize=10.0;
set nodeShapeExternal.scaleType=Width;
set nodeShapeExternal.shapeType=Circle;
set nodeShapeExternal.size=4.0;
set nodeShapeExternal.sizeAttribute="Fixed";
set nodeShapeInternal.colourAttribute="User selection";
set nodeShapeInternal.isShown=true;
set nodeShapeInternal.minSize=10.0;
set nodeShapeInternal.scaleType=Width;
set nodeShapeInternal.shapeType=Circle;
set nodeShapeInternal.size=4.0;
set nodeShapeInternal.sizeAttribute="Fixed";
set polarLayout.alignTipLabels=false;
set polarLayout.angularRange=0;
set polarLayout.rootAngle=0;
set polarLayout.rootLength=100;
set polarLayout.showRoot=true;
set radialLayout.spread=0.0;
set rectilinearLayout.alignTipLabels=false;
set rectilinearLayout.curvature=0;
set rectilinearLayout.rootLength=10000;
set scale.offsetAge=0.0;
set scale.rootAge=1.0;
set scale.scaleFactor=1.0;
set scale.scaleRoot=false;
set scaleAxis.automaticScale=true;
```

```
        set scaleAxis.fontSize=8.0;
        set scaleAxis.isShown=false;
        set scaleAxis.lineWidth=1.0;
        set scaleAxis.majorTicks=1.0;
        set scaleAxis.minorTicks=0.5;
        set scaleAxis.origin=0.0;
        set scaleAxis.reverseAxis=false;
        set scaleAxis.showGrid=true;
        set scaleBar.automaticScale=true;
        set scaleBar.fontSize=10.0;
        set scaleBar.isShown=true;
        set scaleBar.lineWidth=1.0;
        set scaleBar.scaleRange=0.0;
        set tipLabels.colorAttribute="User selection";
        set tipLabels.displayAttribute="Names";
        set tipLabels.fontName="sansserif";
        set tipLabels.fontSize=8;
        set tipLabels.fontStyle=0;
        set tipLabels.isShown=true;
        set tipLabels.significantDigits=4;
        set trees.order=true;
        set trees.orderType="decreasing";
        set trees.rooting=true;
        set trees.rootingType="User Selection";
        set trees.transform=false;
        set trees.transformType="cladogram";
end;
```

Supp_tree_additional_Eph_LBD_complete_mining_tree_file

```
(Ifas_39595:0.9028843134,
(((Xtes_94993:0.1094569785,Xtes_94992:0.0881723367)100:0.3016546523,
(((((((Smp_124210.1_pep:
2.0061448194,Pper_114995:1.2529197659)95:0.3437180998,Ifas_36548:0.6
673514437)88:0.3159742490,
(((((Cele_75907:0.1568112995,Kvar_222799:0.1683330088)100:0.0831012
317,Cele_133760:0.4486776678)86:0.1105038202,Lapi_154083:0.241449769
3)83:0.0670895234,Cpro_80288.25:0.2545127081)96:0.1366419038,
((Cvar_1757:0.3996699801,
((Cvar_19694:0.0618771742,Cvar_19697:0.0000021625)100:0.0471521912,C
var_19695:0.0632397590)100:0.0370566561)85:0.0087031642,
(Cvar_18894:0.2543509724,Cvar_19692:0.1254081851)85:0.0248053367)100
:
0.2366976991)96:0.3143538264,Cele_48040:1.1646912577)95:0.4023189691
)76:0.1851041562,((Cvar_27357:1.2374277506,
((((((((Cpro_75438.40:0.9402807007,Psub_7666:0.9262209040)98:0.07808
73320,Cele_85965:0.6935115559)78:0.1213505741,Psub_14382:1.160819418
2)89:0.0564389273,Kvar_222743:0.5802915553)66:0.0766210463,
((Kvar_2233:0.8609301535,Lapi_108357:0.2075459278)97:0.2614280639,La
pi_159044:0.2793159126)94:0.0546761432)67:0.1131063287,
(Kvar_137296:0.3755669982,Lapi_126388:0.3572763454)100:0.1447871730)
99:0.2914982098,
(((Cele_124391:0.3813845375,Kvar_4564:0.2642352719)100:0.2024029828,
Cpro_59066.22:0.3714694309)100:0.1245308338,Lapi_96494:0.3661004136)
```

100:0.2387365246)86:0.2005898641,Cpro_78852.34:0.9047333856)100:0.17
42408413)100:0.3759681754,Slac_1485:1.0203850273)85:0.2352909212)49:
0.0730862865,((((((((((((((SSCA007952-PA:
0.0970829366,tetur38g00020.1:0.1135472489)96:0.0109708644,KFM62390:0
.0635774898)96:0.0160656808,ISCW022934-PA:
0.2223889726)99:0.0471898339,(((Lith_3827:0.0252706815,SMAR002974-
PA:
0.0225250379)99:0.0210145239,Lith_21051:0.0111232437)100:0.066759053
8,
(Dpul_79285:0.0000021608,EFX79285:0.0000021608)100:0.0535639635)95:0
.0320577589)58:0.0000023336,
((((ENN72307:0.0673081206,TC033957_001:0.0000021655)75:0.0000021224,
ENN72308:0.7304802203)98:0.0620078273,PHUM576020-PA:
0.1996761434)66:0.0093814090,(((((BGIBMGA002079-TA:
0.3607036697,HMEL008812-PA:0.0887007875)99:0.0268989740,ACYPI064034-
PA:0.2061965798)67:0.0271171596,(((AGAP000489-PA:
0.0000021608,ADAC004174-PA:0.0000021608)70:0.0000021608,CPIJ014812-
PA:0.1830032193)98:0.0123449611,
((((((FBpp0414687:0.0000021608,FBpp0126032:0.0000021608)72:0.0000021
608,
(FBpp0325417:0.0066515847,FBpp0182284:0.0066699062)99:0.0066337965)1
00:0.0063345674,
(FBpp0228658:0.0000021608,FBpp0163312:0.0000021608)96:0.0007532498)1
00:0.0061533549,(KNC34063:0.0000021608,TDAL011489-PA:
0.0000021608)99:0.0133496006)100:0.0337543436,MESCA003372-PA:
0.0203002769)50:0.0000021608,Mdes004538-RA_cds:
0.0132780655)97:0.0078281167)99:0.0908892655)60:0.0235978675,RPRC012
412-PA:0.1083034924)88:0.0000021066,(((BIMP12060-PA:
0.0000000000,XP_003400363.1:0.0000000000):
0.0000021608,XP_020722019.1:0.0000021608)100:0.0598342255,
(XP_011157324.1:0.0000021608,XP_011157319.1:0.0000021608)100:0.03538
78588)100:0.0767062769)89:0.0127569395)99:0.0721519247)88:0.09873193
09,EMLSAP00000000364:0.5594846338)100:0.2497126754,
(((OVOC11316:0.1058344682,Bm5071:0.1087627086)100:0.6869319336,SRAE_
X000163000:1.7920468604)84:0.0927248276,EFV53427:1.1036725954)83:0.0
942577455)84:0.1302749719,
(((Hpsi_234025:1.1314674943,g1515.t1:0.4416228874)75:0.1128785735,
(Ocbimv22021576m.p:0.4423536418,(EKC33758:0.5684846706,
(Lgig_105485:0.0000021608,LotgiP105485:0.0000021608)100:0.2814955013
)99:0.0998270204)100:0.2879263987)65:0.1017016919,
(Tpol_7305:0.3924596144,
(Ctel_198909:0.0000021608,CapteP198909:0.0000020330)100:0.5644897418
)94:0.1561246256)98:0.2822809200)93:0.1073423021,
(GSADVT00007809001:0.0640665578,
(GSADVT00000371001:0.0000021608,GSADVT00013698001:0.0061938817)100:0
.1078523689)100:0.8913286666)82:0.0950597191,
((((((((((((Pmar_131.1:0.5546312154,Pmar_9793.1:0.0286393520)100:0.
2326508752,
(Pmar_7260.1:0.1146799531,Pmar_10202.1:0.1428672455)100:0.3250973662
)99:0.1552920313,(((Drer_121462.2:0.1750640471,(EPHA6_MOUSE:
0.0271771383,EPHA6_HUMAN:
0.0135903234)100:0.0389865693)100:0.1282318783,(((EPHA8_MOUSE:
0.0168118831,EPHA8_HUMAN:
0.0177809038)100:0.1573590413,Drer_41799.6:0.0997604926)100:0.146114

8941,((EPHA1_MOUSE:0.0084434972,EPHA1_HUMAN:
0.1001711091)100:0.4738439158,((EPHA2_MOUSE:
0.0647972116,EPHA2_HUMAN:0.0000029847)100:0.1680166413,
(Drer_44917.5:0.3240070032,Drer_11069.6:0.3489758155)100:0.369202284
3)99:0.1556967043)100:0.3469257577)56:0.0655037328)58:0.0663348184,
(Drer_21706.11:0.1017959183,(EPHA3_MOUSE:0.0000021608,EPHA3_HUMAN:
0.0000021608)100:0.0415577776)100:0.1158775822)61:0.0605568432)74:0.
0405980223,((Drer_134983.1:0.1394922189,(EPHA5_MOUSE:
0.0067916279,EPHA5_HUMAN:
0.0067237536)100:0.0519647075)100:0.1510258978,
((Drer_30606.8:0.0903614469,Drer_96552.3:0.1095974949)100:0.16299305
14,
(((Drer_123962.1:0.0000021608,Drer_134275.1:0.0000021608)100:0.03754
56523,Drer_3161.9:0.1303547913)100:0.0522061950,(EPHA4_MOUSE:
0.0199656063,EPHA4_HUMAN:
0.0000021608)93:0.0135722305)93:0.0534766888)99:0.1171242537)81:0.04
55929794)74:0.0265445816,
(((Drer_131364.1:0.0000021608,Drer_93563.5:0.0000021608)100:0.091393
8661,(EPHA7_HUMAN:0.0000022115,EPHA7_MOUSE:
0.0067765069)65:0.0167946354)100:0.0877391918,((EPHA10_HUMAN:
0.0150406725,EPHA10_MOUSE:
0.0269301303)100:0.1545223705,Drer_99872.3:0.1446188487)100:0.146997
3020)86:0.0766948843)96:0.0419446559,
(Pmar_9310.1:0.1583039476,Pmar_2234.1:0.1148965637)100:0.0645876388)
100:0.2887912546,(((((EPHB6_HUMAN:0.0369355860,EPHB6_MOUSE:
0.0377669135)100:0.5312286474,Drer_113830.2:0.4872188646)100:0.52300
92087,((EPHB2_HUMAN:0.0081048329,EPHB2_MOUSE:
0.0055219878)100:0.0572026191,
(Drer_112928.2:0.0707344936,Drer_43755.7:0.0195087367)100:0.04547371
08)60:0.0126056822)56:0.0835557583,Pmar_7951.1:0.1208893186)55:0.032
5556129,((EPHB1_HUMAN:0.0000021608,EPHB1_MOUSE:
0.0000021608)100:0.0772225652,Drer_136066.1:0.0219849376)100:0.10805
03745)56:0.0364278431)54:0.0650832618,((EPHB3_HUMAN:
0.0000021608,EPHB3_MOUSE:0.0065887630)100:0.0845627661,
(Drer_140419.1:0.0611641561,Drer_40208.7:0.1420714539)95:0.045470705
9)100:0.1256282801)99:0.1425149146,
((Drer_88414.3:0.1708457144,Drer_135357.1:0.1799283348)100:0.0747377
150,(EPHB4_HUMAN:0.0652112906,EPHB4_MOUSE:
0.0215225451)100:0.3824889399)100:0.3062109966)85:0.0755464178,
(CiEphe:0.5892104217,CiEphc:
0.3650382906)100:0.4536926653)92:0.1065098025,(CiEphf:1.0662459609,
((CiEphb:0.1917238962,CiEphd:0.1893476844)100:0.2227922932,CiEpha:
0.5217608132)93:0.1271135182)90:0.0729051592)95:0.1203637810,
(Skow_11098:0.6650099499,
(BfEph2:0.1277829402,BfEph1:0.1646358308)100:0.2629404274)95:0.15425
11360)89:0.0558321374,(SPU_027145-tr:
0.0000021608,Spur_027145:0.0000021608)100:0.8121222224)98:0.20905482
75)82:0.0962959743,
((((((((((((Apal_549317:0.1469989793,Apal_25755:0.3705865137)50:0.
0166344226,(Apal_549316:0.1998112513,
(Apal_215207:0.0746106006,Apal_215220:0.0355849980)100:0.1662975549)
81:0.0414695857)28:0.0202216760,
((Apal_215214:0.1695376287,Apal_215213:0.0000021625)99:0.1294765005,
Apal_215216:0.0463223660)85:0.0709182536)58:0.0060068351,

(Apal_215211:0.1216640525,Apal_215219:0.0479507370)100:0.1971510421)
81:0.0570334212,Apal_215204:0.2191826814)96:0.1685730321,Apal_307048
:
0.7606890445)100:0.2881285330,Hdig_11359:0.6167442338)100:0.30215666
01,((Btue_32401:0.7392317004,
(Apal_455194:0.4641606366,Apal_612045:0.6267566547)99:0.3271321582)5
6:0.1668807187,
((EDO39123:0.0000021608,Nvec_39123:0.0000021608)100:0.0677511884,
(Nvec_39124:0.0000021608,EDO39124:0.0000021608)100:0.0538948648)100:
1.1656686212)95:0.2327037721)99:0.3625643450,
((Adig_12482:0.1122430187,Adig_18229:0.1353756762)37:0.0196748747,
((((Adig_15481:0.1935597756,
(Adig_20930:0.0617924384,Adig_03916:0.1280653120)100:0.2053479575)98
:
0.1400072793,Adig_12429:0.1013808579)79:0.0578459858,Adig_00805:0.10
48513821)80:0.0508960575,Adig_04039:0.1119050468)80:0.0133770471)100
:
1.0997166340)96:0.1048368902,Pcar_315635:0.9125993413)99:0.119068420
3,
((Apal_405536:1.0457090937,Btue_32074:0.3495626005)100:0.1773447130,
(EDO40993:0.0000021655,Nvec_40993:0.0000021608)100:0.7582307667)99:0
.3404067274)82:0.1274833141,
((Adig_13888:0.0267898436,Adig_13889:0.0130943334)100:0.1877153715,A
dig_18762:0.1376960539)100:1.2834258296)79:0.2100674638,
(((Ccan_99557:0.7560088362,Ocar_909:1.0544886083)96:0.4299844617,Adi
g_13891:0.9958635001)63:0.1054971743,
(g4371.t1:0.8952206523,g27553.t1:1.1953463869)100:0.6872673936)43:0.
1579224244)28:0.0714059197,((((CBG13447:0.2249302807,
(CBN17142:0.0000021608,CBN08102:0.0000021608)100:0.0522136518)96:0.0
176360530,CJA06042:0.1021580420)92:0.0120505882,CRE17286:0.036070631
6)91:0.0451059176,M03A1.1b:
0.0000026170)100:1.2717866408)27:0.1097587354)24:0.0775759888,
((((Edun_188839:1.0443473333,Mlei_02521a-PA:
0.6462223310)90:0.2832596726,Baby_41769:0.4597962613)87:0.1656143342
,Patl_251869:0.3002546904)99:0.3164900889,Cast_22203:0.4029618701)10
0:1.3133773676)76:0.1514310627,
(((((Snux_119736:0.2163594028,Rfib_2729:0.2822950645)100:0.514779644
4,Avas_85009:0.4653007915)100:0.6337991190,
((((Avas_93993:0.1395824781,Avas_93988:0.1066834180)100:0.1667377446
,
((Avas_56846:0.1019491535,Avas_56851:0.0722124021)100:0.0341068388,A
vas_56854:0.0910048011)100:0.1159192532)100:0.2914838770,Snux_109225
:0.7038389436)100:0.3526917445,
(Rfib_47059:1.4867600095,Avas_82832:0.4952869502)70:0.2895705400)71:
0.2450651279)41:0.1117752921,Avas_63694:0.9499064323)71:0.1877947356
,Hpop_15474:1.1490071798)99:0.5844936400)82:0.1495210290,
(((TriadP53296:0.0000021608,Tadh_53296:0.0000021608)100:1.0717925583
,(TriadP56934:2.3670868339,
(Tadh_56933:0.0000021608,TriadP56933:0.0000021608)100:0.7588557486)9
4:0.1848915117)99:0.7585880543,
(Aele_22617:1.1772931451,Clat_102964:1.0941752463)99:0.6727319628)88
:0.1224080786)93:0.1368789778,
((Cnul_32703:0.0000024087,Cnul_32701:0.2369300769)100:1.6806243679,
((Kvar_25216:0.7198912846,Cele_137847:0.5446070517)99:0.2917731416,L

api_88092:0.8160754386)99:0.2566101841)87:0.1735799396)84:0.14829100
97,
((Hamb_7684:0.2246655715,Aque_11657:0.3326798553)100:0.4321519676,Ni
ph_32084:0.9098560418)100:0.8086727538)67:0.0612599397)99:0.43388440
84,Aque_37707:0.9300044472)100:0.5002957738,Pfic_36041:0.2196636540)
99:0.1321241156,
(Niph_54283:0.0210982242,Niph_54284:0.0392361685)100:0.3686111514)99
:0.4759900102)99:0.1341955947,
((((Aque_05648:0.0410393064,Aque_32613:0.0000022543)99:0.0504542647
,Aque_32612:0.0662282510)92:0.0000027460,
(Aque_03862:0.0583836894,Aque_12969:0.0444591552)57:0.0137708394)57:
0.0144097019,(Aque_37706:0.0977445097,
(Aque_41463:0.0274312815,Aque_16689:0.1894808665)95:0.0151648805)50:
0.0106627168)82:0.0428907759,Aque_37708:0.0000026631)99:0.5896836358
)100:0.7271069510,Vmul_37755:1.6427769788);

Supp_tree_additional_Eph_tyrosine_kinase_domain_complete_mining_tree
_file

(HMEL008812-PA:8.4800988640,((Smp_124210.1_pep:8.5795018559,
((g27553.t1:9.9999984272,g4371.t1:9.9999987885)96:4.3905617273,Triad
P53296:9.9999988825)72:2.0459522685)76:0.7382312263,
(((((((((((Aspe_Peph_146412:2.3595731446,Aspe_Peph_158325:2.386959
6798)99:0.3942920313,
(Mbre_Peph_25247:1.7533478568,Sros_Peph_EGD72856:1.3396406888)100:0.
6838762115)99:0.2794837503,Mbre_Peph_26435:2.8782202922)89:0.2007330
011,(((EDO39123:0.4414940245,EDO39124:0.0017914829)100:4.5788123324,
((Cowc_Peph_01676T0:1.6658347030,Cowc_Peph_09852T0:1.2296213461)100:
1.4006794719,Mvib_Peph_25039:2.1548145480)98:0.4697528388)77:0.30995
67055,
(Apar_Peph_165057:2.3861147258,Sarc_Peph_10364T0:2.6373633608)99:1.3
585724869)47:0.3550090667)60:0.2711818945,Aque_32612:6.5790417057)50
:
0.1157933363,SRAE_X000163000:2.5410909978)38:0.1219431719,Sros_Peph_
EGD75115:1.9313066403)69:0.2520547405,
(((Baby_41769:0.4211674994,Patl_251869:1.5875326268)98:0.1461439544,
Mlei_35913a-PA:
0.3811493717)100:1.9272463412,Tadh_12344:1.9927174208)83:0.217195028
4)58:0.2304356953,
((((((Nbij_150435:0.2157528114,Aele_129846:0.0560209919)100:0.07719
69930,
(Clat_102964:0.0560982052,Atet_45096:0.0892186725)100:0.1665176674)1
00:0.3580841713,
(Clat_76838:0.1480860816,Aele_55285:0.2168719504)100:0.2516181170)10
0:0.2150690938,
((((Hvul_13820:0.4103248667,Hvul_31223:0.4347613582)99:0.1229095881,
Hvul_37681:0.1024496619)99:0.1469500282,
(Hvul_37741:1.4277681824,Hvul_13823:0.3138672782)100:0.1742040825)98
:
0.1829673633,Clat_108627:0.9363100629)99:0.1700736693)97:0.164900473
3,Hvul_18252:0.5982559980)100:0.5773593520,
((((((Pcar_152322:0.1688151195,Pcar_152315:0.0000021579)100:0.292081
5087,Adig_00802:1.5373943460)99:0.5006599397,
((((Apal_271591:0.4816344591,Apal_306140:0.0666898851)94:0.073332978

1,Apal_306157:0.0165492623)93:0.1385809818,
(Apal_455194:0.7382902669,Hdig_16446:0.3712734000)73:0.0977633952)76
:
0.0448837543,Apal_306159:0.2167821415)97:0.1972752087)76:0.094951542
3,Nvec_33736:0.4833286542)99:0.2183323177,
((Adig_12429:0.0928832009,Adig_04039:0.0747791146)100:0.1140016915,A
dig_15481:0.0980623628)100:0.8531178915)96:0.2191956425,
(((((Adig_18762:0.5644578878,Pcar_328883:0.3549393700)100:0.15271131
72,
(Apal_103658:0.3084122981,Nvec_40994:0.2493340247)100:0.1822880021)9
9:0.1903823900,Ever_3805:1.0426584352)69:0.1009386082,
((Ever_2431:0.8760295948,Ever_12042:0.9914606718)87:0.2628902016,Eve
r_14808:0.9583842169)71:0.2150088440)41:0.0352909563,
(Ever_20101:1.1925790676,Ever_3065:1.1351995804)74:0.2760389957)54:0
.0808554175)83:0.1683059572)81:0.1355736558,
(Scoa_1400:1.5061364203,Ocar_5890:1.2523281720)92:0.5543320444)67:0.
1164959415)41:0.1710732694,(((Avas_85009:1.1056981330,
(Snux_6723:0.2440066868,Rfib_47059:0.2873424659)100:0.5581711820)100
:0.7480378735,(((Avas_63694:0.8965916872,
(Rfib_55118:0.2264992334,Snux_99202:0.0911901132)100:0.3727852075)10
0:0.5537108086,((Avas_85324:0.5422109853,
(Snux_109225:0.2709333303,Rfib_49817:0.2873143677)100:0.3372311640)9
7:0.1886516381,Avas_93988:0.4695141198)100:0.3047713161)98:0.1795557
344,
(Avas_82832:0.5724475452,Rfib_47064:0.6567875000)100:0.6607608131)10
0:0.1758137172)100:0.8205339016,(Cnul_9016:1.2003427864,
(((Cpro_79320.39:0.1412797503,Lapi_154083:0.1831084041)100:0.0833566
893,Kvar_222799:0.2973462603)100:0.7210010887,
((Niph_54284:0.3110804183,Pfic_10202:0.4067495556)100:0.1957626656,
(Xtes_54061:0.4010486147,Aque_41463:0.5127360471)100:0.0367710273)10
0:0.9836183698)100:0.4965319506)97:0.1980235685)97:0.2948618930)68:0
.1952585835,
((TriadP56933:8.7570157400,Bm5071:0.3260378065)74:0.1687092514,
((((M03A1.1b:0.0000020787,M03A1.1a:
0.0000020787)100:0.0413049929,CJA06042:0.1022882679)96:0.0286337933,
CRE17286:0.0777824639)74:0.0072674760,CBG13447:0.0573364952)71:0.015
0803843,
(CBN17142:0.0000020787,CBN08102:0.0000020787)100:0.0145225179)98:0.6
948309232)76:0.8050346607)66:0.1162972008,((((((((((((((SMAR002974-
PA:9.9999981944,(Pmar_5131.1:0.0602920619,
(Drer_43755.7:0.0263246292,(EPHB2_HUMAN:
0.0288277044,Drer_112928.2:0.0603524569)42:0.0138462777)49:0.0421244
106)50:0.0000023622)50:0.0721089623,Pmar_7951.1:0.1842247182)49:0.04
18237165,((((Drer_113830.2:0.8611574073,EPHB6_HUMAN:
1.2585045945)97:0.4764299604,
((Drer_135357.1:0.0778832076,Drer_88414.3:0.0868914188)100:0.1111586
476,EPHB4_HUMAN:0.1250915083)100:0.1557298386)77:0.0532085644,
((Drer_121980.1:0.0000020787,Drer_111570.2:0.0000020787)100:0.062889
7197,EPHB1_HUMAN:0.0312628193)100:0.0804306012)77:0.0555511926,
((Drer_40208.7:0.3195254083,Drer_140419.1:0.0075540001)85:0.03007890
92,EPHB3_HUMAN:
0.0699337678)86:0.0749118647)90:0.0393357454)50:0.1576266998,
((((((EPHA10_HUMAN:
1.1132702792,Drer_141023.1:0.7066702467)100:0.6726851657,

((Drer_93563.5:0.0000020787,Drer_131364.1:0.0000020787)100:0.0454203
352,EPHA7_HUMAN:0.0612658467)100:0.1445277602)83:0.0851896858,
((EPHA8_HUMAN:
0.3725700921,Drer_41799.6:0.2408260335)100:0.2011509063,
(EPHA6_HUMAN:
0.0705025079,Drer_121462.2:0.1821421535)100:0.2232170476)98:0.045954
9383)81:0.0292850903,((Drer_21706.11:0.0688089854,EPHA3_HUMAN:
0.0362179705)100:0.1361889774,
(Drer_75583.4:0.1628842672,EPHA5_HUMAN:
0.1212193382)100:0.0753035221)99:0.0723971064)80:0.0401475990,
(((Drer_30606.8:0.0317284484,Drer_96552.3:0.1353684983)100:0.1674695
428,
((Drer_3161.9:0.1592695099,Drer_123962.1:0.0609693230)100:0.05755994
14,EPHA4_HUMAN:
0.0283798050)100:0.1227532809)58:0.0275896280,Pmar_10282.1:0.5793620
457)97:0.0600220746)73:0.0223226572,((Pmar_9793.1:0.2253414809,
(Pmar_10202.1:0.0611613115,Pmar_7260.1:0.0370832680)100:0.0739420963
)100:0.3764008904,((EPHA1_HUMAN:1.4597041879,
((Drer_44917.5:0.1220292935,Drer_11069.6:0.2443823192)100:0.17225806
54,EPHA2_HUMAN:0.3145119018)100:0.2111445880)98:0.0651412569,CiEphf:
2.2182703113)91:0.1353136892)71:0.0636476163)81:0.0401382157,Pmar_22
34.1:0.7036199355)82:0.0404277418)46:0.0629519173,(CiEpha:
1.0225886911,(CiEphd:0.2182782229,CiEphb:
0.1928655251)100:0.1473299081)90:0.2327296437)72:0.1090566229,
((CiEphc:
0.9659008752,TriadP56934:9.9999982850)78:2.3460869659,CiEphe:
0.9094921697)75:0.7591347632)77:0.1344215719,
(BfEph2:0.4220608331,BfEph1:0.1045539035)91:0.4527827621)82:0.142420
5306,(Spur_027145:0.0000020787,SPU_027145-tr:
0.0000026655)100:0.5616824085)80:0.1525239271,((((((((AAEL008301-
PA:0.0147123094,(((FBpp0341564:0.0348590423,
(FBpp0414687:0.1357441482,
(((FBpp0228658:0.0000000000,FBpp0391235:0.0000000000):
0.0000020787,FBpp0395578:0.0000020787)100:0.0067714217,
(FBpp0387469:0.0000020787,FBpp0163312:0.0000020787)99:0.0010523214)7
0:0.0081385177)52:0.0053745053)59:0.0077491283,
(((((((FBpp0341768:0.0000000000,FBpp0350759:0.0000000000):
0.0000020787,FBpp0126032:0.0000020787)100:0.0333344460,
(FBpp0134941:0.0000020787,FBpp0371063:0.0000020787)78:0.0000020787)7
7:0.0036791312,
((((((FBpp0317218:0.0000000000,FBpp0325417:0.0000000000):
0.0000000000,FBpp0326453:0.0000000000):
0.0000000000,FBpp0328187:0.0000000000):
0.0000000000,FBpp0313042:0.0000000000):
0.0000000000,FBpp0314589:0.0000000000):
0.0000020787,FBpp0325148:0.0000020787)69:0.0000020787,
((((FBpp0088151:0.0000000000,FBpp0088153:0.0000000000):
0.0000000000,FBpp0088155:0.0000000000):
0.0000020787,FBpp0088152:0.0000020787)83:0.0000020787,FBpp0088154:0.
0803832416)78:0.0025979883,FBpp0290500:0.0000020787)98:0.0010791320)
100:0.0073977980)76:0.0034973607,
((FBpp0363121:0.0000000000,FBpp0259565:0.0000000000):
0.0000020787,FBpp0357757:0.0000020787)100:0.0037515119)93:0.01803039
10,FBpp0182284:0.0059200652)92:0.0054436296,FBpp0242839:0.0038809764

)85:0.0078147922,TDAL011489-PA:
0.0269556162)70:0.0038480520)77:0.0064145397,KNC34063:0.0230683154)9
9:0.0525481768)80:0.0214602070,(AGAP000489-PA:
0.1054927704,ADAC004174-PA:
0.0757530695)99:0.0443341751)100:0.5251907888,(RPRC012412-PA:
0.0546718762,(((XP_012064306.1:0.0000020787,
((XP_011157326.1:0.0000000000,XP_011157327.1:0.0000000000):
0.0000020787,XP_011157322.1:0.0000020787)100:0.0035262152)99:0.00356
23478,
((((((((XP_012171130.1:0.0000000000,XP_012171139.1:0.0000000000):
0.0000000000,XP_012171135.1:0.0000000000):
0.0000000000,XP_012171136.1:0.0000000000):
0.0000000000,XP_012171140.1:0.0000000000):
0.0000020787,XP_012171133.1:0.0000020787)95:0.0000023525,BIMP12060-
PA:0.0000020787)99:0.0035594268,GB41629-PA:
0.0000020787)94:0.0095792144,
((((XP_011157325.1:0.0000000000,XP_011157320.1:0.0000000000):
0.0000000000,XP_011157321.1:0.0000000000):
0.0000000000,XP_011157319.1:0.0000000000):
0.0000000000,XP_011157323.1:0.0000000000):
0.0000020787,XP_011157324.1:0.0000020787)100:0.0441257236)100:0.4565
462572,((XP_012171141.1:0.0000000000,XP_003400363.1:0.0000000000):
0.0000020787,XP_020722019.1:0.0000020787)100:0.0070000424)64:0.00000
20205)99:0.0091306424,NV11238-PA:
0.0016782405)100:0.0987235844)89:0.0154400886)61:0.0127190148,
(PHUM576020-PA:0.0629185313,(BGIBMGA002079-TA:0.1807891704,
(TC033957_001:0.4743986769,AGLA005888-RA:
0.0206767039)100:0.0731191909)100:0.0491049994)97:0.0170101980)91:0.
0287751501,ACYPI064034-PA:0.0994456479)99:0.0624292984,
(Lith_3827:0.0326501872,
(Dpul_79285:0.0000020787,EFX79285:0.0000020297)100:0.1613820570)99:0
.
0557718931)99:0.0634337939,KFM62390:0.1042672557)67:0.0295238104,ISC
W022934-PA:
0.0611520421)68:0.0312552959,tetur38g00020.1:0.2937843025)99:0.18514
24282)81:0.0766151812,g1515.t1:0.2276681107)45:0.0004361310,
((Ctel_198909:0.0000020787,CapteP198909:0.0000025975)99:0.1284865903
,EDO40993:9.9999985162)86:0.1964214328)75:0.0663388101,
((Lgig_105485:0.0000020787,LotgiP105485:0.0000020798)100:0.138552256
9,Hpsi_102387:0.2328041742)82:0.0420610264)67:0.0470944411,EKC33758:
0.3186397820)76:0.0997271538,Tpol_63971:0.2383362013)87:0.2653959927
,
((GSADVT00007809001:0.0000020787,GSADVT00027792001:0.0000020787)100:
0.1728471814,
(GSADVT00013698001:0.0005443111,GSADVT00000371001:0.2012428998)100:0
.
1774647795)100:0.6496395379)78:0.2681599873)34:0.0000024531,EFV53427
:1.3294332503)77:1.7657726260)87:6.4436717091,MESCA003372-PA:
9.9999986387);

Supp_tree_additional_Ephrins_complete_mining_tree_file

(SPU_023757-tr:1.6564279644,((((((LotgiP171062:1.4751780867,
(Ocbimv22026748m.p:0.5718394946,PHUM090120-PA:

5.5108633438)96:0.7046160496)97:0.5368475988,
((CapteP225002:0.9033965817,HelroP194703:2.2014111489)100:0.39615318
70,EKC32286:2.1842920273)91:0.2721266312)67:0.1602112820,
((HelroP188210:3.3312366557,
(Hpsi_83841:0.1095975596,Hpsi_83847:0.0755891394)100:1.3612185694)53
:0.1360459963,
(Tpol_19734:0.1263022225,Tpol_19735:0.0920558869)100:1.3135025517)54
:0.0793149562)86:0.4596887563,(((((((((((EDO47804:1.4718546660,
(EDO36327:0.5642318205,
(Apal_398190:0.1429862836,Hdig_30557:0.3452314165)100:0.5228090937)1
00:0.4140082411)43:0.0598323736,Pcar_235347:1.0399448118)75:0.197691
7422,
(Apal_41158:0.8504215787,Adig_07747:1.5378894649)94:0.3076557077)100
:0.2119909188,((((EDO44950:0.7264743883,
((Apal_344094:0.2017537872,Hdig_22696:0.3283788068)100:0.2415359064,
(Btue_29916:0.0082121747,Btue_30708:0.0908069534)100:0.4759642443)10
0:0.1708116976)100:0.3391963236,
((Apal_400465:0.7456556162,Hdig_5881:0.9968154995)99:0.1943353630,Bt
ue_23164:1.1914290189)99:0.4991297733)79:0.2122904661,
((Ever_204:0.6390731303,Ever_1897:1.2315262627)100:0.9174682098,
(Pcar_189454:0.8814067174,Adig_20275:1.2042610155)100:0.5046027011)6
3:0.1594542525)48:0.1137393512,Aaur_20911:1.5243952338)35:0.03607003
16)62:0.3816826810,
(Apal_54304:1.3892518805,Adig_04407:2.0683446431)88:0.2008768255)76:
0.1481417640,
(((((Apal_288439:0.1235165179,Apal_288431:0.2487389469)100:0.1718304
614,Apal_8979:0.1267303550)100:0.4680100184,
((Btue_9751:0.2214897896,Btue_9750:0.1699132766)99:0.7415984615,Hdig
_4952:0.5156488902)96:0.2948719415)99:1.0483553525,
((Pcar_240721:1.3014542910,(Adig_03889:1.5349797253,
((Adig_06508:0.1045652650,
(Adig_10295:0.3635608423,Adig_20773:0.1225962181)95:0.1243907967)91:
0.0426322199,Adig_19031:0.1283560073)94:0.2517052462)100:0.631412803
6)99:0.4700317124,Aspe_Cu_472242:4.9871526599)93:0.3753545690)96:0.1
807981998,
((((Apal_344111:0.8021713583,Btue_12517:2.2299258010)83:0.2095782637
,Hdig_22213:0.8196196849)100:0.9103109983,Pcar_215100:1.5938742906)9
8:0.2616972938,Ifas_Cu_19075:4.1365733066)79:0.1249673022)91:0.20293
50818)72:0.1547611123,
(Apal_89985:2.3657205159,Pcar_179493:1.5326952202)84:0.4371479758)63
:0.1151391305,(((((Smp_133090.1_pep:
3.8633150825,Ever_19722:1.6679021817)96:0.8185812907,Hvul_1029169.1:
1.9948825863)84:0.1685351492,Pper_140147:1.7870893186)88:0.222446072
9,
((((((Atet_66572:0.5406145544,Atet_41310:0.5792583550)100:0.31170023
54,Clat_97078:0.2482325997)100:0.3620319603,
((Aele_151942:0.2370974234,Aele_151940:0.0000021919)100:0.1968275994
,
(Aele_151938:0.3754145218,Nbij_52843:0.2735897309)100:0.3302555709)1
00:0.3516105799)100:0.3875605157,Pphy_29075:1.5060608148)99:0.326318
5489,((Holi_5987:0.2453792346,
(Hvul_1033087.1:0.0026462113,Hvul_1038805.1:0.2423450663)100:0.14233
91784)100:0.4098321692,Hvir_3164:0.3038355199)100:1.0205287563)99:0.
2347954352,Pper_55548:1.8219897326)100:0.5402142637)83:0.2996175545,

Ever_13730:1.7529931970)60:0.2243192928)32:0.0691538799,
(((((((((((Atet_141600:0.6799317494,Clat_80728:0.2586003673)100:0.46
94624799,
(((Atet_40821:1.0415813694,Clat_28862:0.4786821008)100:0.2030985118,
Clat_48253:0.6682994924)100:0.3492223098,Pphy_19559:0.9786665116)100
:0.3735423135)100:0.1647385991,
(Aele_79976:0.4961428525,Nbij_20083:0.6110830867)100:0.1690690302)10
0:0.3115416140,(Atet_88599:0.5219527768,
((Aele_124411:0.3919526530,Nbij_143918:0.6590247363)100:0.2354092813
,Clat_120953:0.7077386398)100:0.3255064390)100:0.6298409457)99:0.347
5470712,Pphy_49294:1.3502084939)48:0.1056628675,
((Holi_3973:0.3454071874,
(Hvul_1018918.1:0.0742871853,Hvul_1028415.1:0.1054477453)100:0.19078
62238)100:1.0146547450,
((Holi_7726:0.1396027641,Hvul_1019657.1:0.5789930866)100:0.580171521
0,Hvul_1034474.1:2.1500298932)98:0.6283103606)95:0.4099121175)100:0.
4398736320,Pper_105129:1.3791723579)99:0.1923761675,Pper_103077:1.92
62980972)100:0.1815120043,(Atet_192642:2.0461107031,
(((Aele_7590:0.9105407728,Nbij_23770:1.0782724367)100:0.3329989167,C
lat_22532:1.3478278651)100:0.4592124451,
((Holi_14653:0.3443557309,Hvul_1028689.1:0.1470022476)100:1.00003763
13,
(Pphy_13023:0.2594768594,Pphy_14332:0.6697629208)100:0.4001404594)10
0:0.4384511019)100:0.3843431824)100:0.5362158670)97:0.3644707298,Cca
n_Cu_114223:5.4917830061)80:0.2287596975,Btue_28719:3.6712059437)50:
0.1118860930)89:0.4328808998,(((((CRE14007:7.9346159666,CiEfnAd:
0.2025788605)100:0.6213481381,CiEfnAc:
0.8412988762)100:0.6380314782,CiEfnAb:
2.2274737988)100:0.5707261139,CiEfnAa:1.8217660434)99:0.8606827680,
(((((EFNB1_HUMAN:
0.2610233586,Drer_24428.4:0.3326733409)100:0.3475157004,
(EFNB2_MOUSE:0.3007759695,
(Drer_10432.8:0.2486387581,Drer_79638.5:0.4510320203)100:0.166570363
9)100:0.2558987227)100:0.2983489521,(EFNB3_HUMAN:0.8102684939,
(Drer_62002.6:0.1512585443,Drer_73969.3:0.2976952883)88:0.0860161340
)91:0.3427148378)73:0.4234104033,Pmar_8071.1:1.7564142050)100:0.9917
877846,CiEfnB:
1.3529970737)78:0.3021237545)48:0.1276461881)47:0.1456434040,
((((EFNA1_HUMAN:0.1238998337,EFNA1_MOUSE:
0.1356220483)100:0.5287090128,
(Drer_12577.6:0.4107736088,Drer_50216.6:0.3281210942)100:0.544334893
7)100:0.5288621722,(((EFNA2_HUMAN:0.6333239184,((EFNA5_HUMAN:
0.2605739862,
((Drer_111146.1:0.0000026770,Drer_116412.1:0.1986878224)100:0.326261
3441,Drer_136933.1:0.2154043493)100:0.0350571990)100:0.5919733402,
(Drer_40277.7:0.4967443185,Drer_101723.3:0.3522474337)100:0.20772014
94)100:0.1859508123)100:0.3832989037,(EFNA3_HUMAN:0.4576518715,
(Drer_119988.2:0.2329456167,Drer_133669.1:0.1604465294)100:0.2312403
102)100:0.7389923884)100:0.3874895410,((EFNA4_HUMAN:
0.1608615018,EFNA4_MOUSE:
0.1743474610)100:0.6966501968,Drer_141804.1:1.0288290034)100:0.54489
29248)90:0.0764671231)100:1.5624970056,
((BfEfn1:1.5148782679,BfEfn2:2.0370989200)85:0.1078107110,Mlei_03441
:2.8438765357)46:0.2279570066)35:0.1864135083)51:0.0628931539,

((Spyx_Cu_120946:2.8734725768,Tfer_Rusticyanin:
3.5273822419)91:0.7862602858,Paer_Azurin:
3.8811332210)77:0.4160034485)56:0.1003539706,
(((((((((Bm17311:0.0356568764,
(EJD76147.1:0.0707042029,OVOC9135:0.2316592454)100:0.1387321021)100:
0.5997931314,SRAE_X000008200:1.5245270674)90:0.3399913376,
((C43F9.8:0.1235998172,
(CBN29472:0.2918898056,CBG04418:0.0966678270)75:0.0255550046)97:0.12
16646253,CJA07765b:
0.0690100586)99:0.8736583586)53:0.0000023605,Uper_Plastocyanin:
5.5509597052)84:0.7024168274,
(EFV61089:1.8265207786,EFV49446:1.1344978019)98:0.3685640621)72:0.23
35218770,
((EFV61082:1.1755062977,EFV61087:1.4882907778)99:0.4267833677,
((EJD76078.1:0.1229985592,OVOC4451:0.2089896666)98:0.0780550660,Bm27
26:0.1480324364)99:1.9504349209)88:0.3943262344)82:0.2704621545,
(((((CRE10194:0.2063997555,CBG01610:0.2460442538)100:0.1207650619,CB
N08149:0.4670684298)100:0.1025649993,F56A11.3:0.1612680253)99:0.0655
818995,CJA14032:0.2252686437)100:1.4394146924,
(((Bm11673:0.2415073269,OVOC4018:0.2611916188)100:0.1604412843,EFO17
917.2:0.0000023903)99:1.9667334708,(SRAE_X000209500:2.4615909182,
(((Smp_129000.1_pep:1.2982430049,Smp_133080.1_pep:
2.4364299177)100:1.2854927946,HelroP177176:1.7895390804)83:0.0000020
406,Nbij_57193:3.6748228387)40:0.2471728968)26:0.2705306367)25:0.148
2023876)60:0.3799388508)48:0.1923358121,
((((CBG10748:0.1049529149,CRE10731:0.2008571624)99:0.0328286519,
(Y37E11AR.6a:
0.0899107494,CJA19195:0.2723913627)100:0.0936054887)99:0.1095532362,
CBN21225:0.0197243960)99:0.4039646122,PPA28315:1.2808351762)100:1.12
72878540)43:0.1402676025,
(((CBG07443:0.6646818630,CRE22399:0.9819246146)99:0.2441862861,F15A2
.5:0.8529405762)100:1.4293456047,
(((SRAE_X000065200:2.5688377973,PPA18651:2.6746846369)91:0.704134719
6,(((((Cvar_2778:0.5129350963,
((Cvar_8977:0.9364996278,Cvar_1084:0.9895190132)100:0.3713515966,Cva
r_12230:0.8068408422)99:0.2740036398)100:1.0344128751,Kvar_23578:2.2
252692906)85:0.2574583039,
(Lapi_139996:1.5538647296,Slac_8385:2.0328612677)75:0.1381525358)91:
0.4343848390,
((Cpro_58774.15:1.2898544794,Lapi_57857:1.1397806725)100:0.844462199
5,
(Ifas_6298:0.1359848563,Ifas_6301:0.0327800353)100:1.6420493278)90:0
.
2697073286)99:0.3361696572,Aque_39602:1.7198204857)88:0.0650586593)8
2:0.1311889243,Atha_Blue_copper:
2.7247921374)84:0.0953840647)87:0.3669579625)61:0.1837007669,
((((((((DPOGS203864-PA:0.4780464972,
(((TC008794_001:0.1043601858,AGLA008721-RA:
0.4643790410)64:0.1301191286,ENN77761:0.0607061035)64:0.0380256425,E
NN77760:0.6139486809)100:0.4001485404)99:0.1769696666,TC004127_001:0
.4488138681)98:0.1167099788,((((AGAP009426-PA:
0.0734913267,ADAC006258-PA:0.1541136635)100:0.3392401819,
(AAEL004488-PA:0.0278667348,CPIJ001112-PA:
0.1396209954)100:0.1661742120)100:0.5642775701,(Mdes004544-RA_cds:

0.5347708113,
((((FBpp0405637:0.1887323386,FBpp0163267:0.2350751877)98:0.060254310
9,FBpp0394582:0.2056495975)100:0.1652234153,
((FBpp0122928:0.3228444231,
(FBpp0088144:0.0619210485,FBpp0363337:0.0776592770)100:0.1587988322)
98:0.0593832420,
(FBpp0182251:0.1068691918,FBpp0242833:0.2455532867)98:0.1116927818)9
8:0.0975428864)98:0.0673838854,(KNC32890:0.3909234307,(MESCA003657-
PA:1.4352660504,TDAL010693-PA:
0.2291484550)90:0.2754283149)89:0.0803083890)98:0.3198665346)97:0.16
95268404)97:0.2520871658,(((BIMP16439-PA:
1.7017122588,XP_003394061.1:0.0000029759)100:0.1212889706,XP_0120550
56.1:0.1120108494)100:0.0665684093,NV11883-PA:
0.2398509096)100:0.4893705687)94:0.1022707621)95:0.1219779344,ACYPI0
02895-PA:0.4948013471)95:0.1442548817,((RPRC001731-PA:
0.3481108470,tetur15g03500.1:0.9807697751)96:0.2031636868,
(PHUM089410-PA:
0.4826237365,KZS17355:0.6118190679)94:0.1380741676)95:0.1747842373)9
0:0.0726468047,(((ISCW018996-PA:
0.0000020455,KFM67366:0.6640155574)100:0.3703305321,(SMAR011358-PA:
1.2732368323,Lith_21086:0.2843028227)99:0.3705302398)96:0.1347539285
,(EMLSAP00000001309:1.5127961045,
((((EMLSAP00000004326:0.4751255484,EMLSAP00000004327:0.7406085354)10
0:0.2471350877,EMLSAP00000009373:1.0592457807)87:0.2081334108,EMLSAP
00000004379:1.0769339196)86:0.1915742600,
(EMLSAP00000004329:0.7779350088,EMLSAP00000007655:0.8495947472)100:0
.
1999720444)85:0.3307679918)85:0.3353260270)67:0.1413971855)61:0.1142
700781,tetur15g03520.1:1.5106515898)42:0.3312978791,
(GSADVT00010381001:0.4794863967,GSADVT00033567001:0.3243308757)100:1
.
9635876314)49:0.1588005959,Pcar_395755:7.7547384592)44:0.1261448036)
34:0.0505678618)40:0.2165018525)50:0.2702810365,HelroP169042:2.81583
58087)78:0.0807284177,Skow_16956:1.8127006722)66:0.2241057666,Skow_5
664:1.5207710787);
